# Microbial Similarity and Preference for Specific Sites in Healthy Oral Cavity and Esophagus

**DOI:** 10.3389/fmicb.2018.01603

**Published:** 2018-07-17

**Authors:** Li Dong, Jian Yin, Jing Zhao, Shan-rui Ma, Hai-rui Wang, Meng Wang, Wen Chen, Wen-qiang Wei

**Affiliations:** ^1^Department of Epidemiology, National Cancer Center/National Clinical Research Center for Cancer/Cancer Hospital, Chinese Academy of Medical Sciences and Peking Union Medical College, Beijing, China; ^2^Institutes of Biomedical Sciences, Shanxi University, Taiyuan, China

**Keywords:** microbial similarity, microbial preference, oral cavity, esophagus, 16S rRNA gene sequencing

## Abstract

Human microbial communities are highly complex ecosystems, but it remains unclear if microbial compositions have any similarity in distinct sites of the oral cavity and esophagus in particular. Clinical samples were collected from three niches (saliva, tongue dorsum and supragingival plaque) of the oral cavity and three segments (upper, middle, and lower) of the esophagus in 27 healthy individuals. Bacterial V3-V4 region of 16S rRNA gene in these samples was amplified and sequenced on Illumina sequencing platform, followed by data analysis using QIIME and LEfSe softwares. Highly diverse bacterial flora with 365 genera belonging to 29 phyla resided in the oral cavity and 594 genera belonging to 29 phyla in the esophagus. The phyla Proteobacteria, Firmicutes, Bacteroidetes, Actinobacteria, Fusobacteria, and TM7 were most abundant in both the oral cavity and the esophagus, but the phyla Actinobacteria and Bacteroidetes were preferable in the oral cavity and Firmicutes in the esophagus. The genera *Streptococcus, Neisseria, Prevotella, Actinobacillus*, and *Veillonella* were most abundant in both oral cavity and esophagus, but *Neisser*ia was preferable in the oral cavity and *Streptococcus* in the esophagus. Different niche-specific bacterial signatures were found in the oral cavity, e.g., the class Flavobacteria in the supragingival plaque, class Bacteroides in the saliva and the class Clostridia in the tongue dorsum. By contrast, no site specific bacteria for three different segments of esophagus were found. However, high variability of microbial compositions between individuals was observed. In conclusion, this study confirmed microbial diversity at different taxonomic levels in healthy oral cavity and esophagus, and identified the site-preferable bacterial signatures in six niches of the upper digestive tract. These findings provide a critical baseline for future studies interpreting microbiome-related diseases.

## Introduction

Human microbiome in the upper digestive tract is a very complex and highly diverse ecosystem (Bashan et al., 2016). Homeostasis of microbial diversity is vital for the development of mucosal barrier functions and immune responses to the invasion of pathogens leading to the diseases (Frank et al., 2011; Tlaskalova-Hogenova et al., 2011; Segata et al., 2012). Oral cavity, as the initial gateway of the integral digestive tract, is constantly exposed to both the inhaled and ingested microbes with more than 700 bacterial species or phylotypes (Aas et al., 2005; Nasidze et al., 2009), part of which have been reported to be associated with the cancer and other systemic diseases (Abusleme et al., 2013; Kerr, 2015; Fan et al., 2016). It has been estimated that about 10^11^ bacterial cells flow from the mouth to the stomach per day (Socransky and Haffajee, 2002; Segata et al., 2012), and microbial compositions are overlapped along the oral, pharyngeal, esophageal and intestinal locations (Segata et al., 2012; Norder Grusell et al., 2013; Gall et al., 2015). However, it remains unclear to date if microbial compositions have any similarity in distinct sites of the upper digestive tract, oral cavity and esophagus in particular.

Esophagus plays a critical anatomic role in transferring the alimentary bolus from the mouth to the stomach and also in receiving the reflux from the stomach. Therefore, these varied microenvironments in the esophagus, e.g., the exposure to oral-like environment in the proximal esophagus and a sudden lowering of pH values in the distal esophagus might lead to microbial diversity in different segments (Di Pilato et al., 2016). Meanwhile, the esophagus is characterized with three physiological stenosis where residual food and microbes are more prone to retain and the risk of invasive cancer or esophageal lesions might be increased (Rice et al., 2009). Microbial diversity in the distal esophagus in patients with esophagitis has been reported (Pei et al., 2005). However, few studies analyze microbial compositions that resided in different esophageal segments, particularly in healthy individuals. This is in part due to the difficulty in procuring the esophageal samples that always requires an invasive method or limited capability of traditionally culture-based methods to identify the unknown, uncultivable and unclassified species (Pozhitkov et al., 2011). Not until recently, Pei et al. (2004) firstly found the most six prevalent phyla in four healthy individuals using 16S rDNA sequencing and widened our knowledge about microbial spectrum in distal esophagus. Similar investigations have been performed in different countries (Yu et al., 2014; Gall et al., 2015; Feng et al., 2017). Nevertheless, some limitations such as only distal or proximal esophagus sampled, biopsy-based rather than mucosal brushes sampling, culture-based method rather than sequencing in these studies precluded us from revealing a detailed spectrum of microbiome along healthy esophageal tract (Gall et al., 2015). In addition, fewer studies reported the similarity and specificity of microbial communities that reside in the oral cavity and the esophagus.

Understanding microbial spectrum in healthy population is of great value to demonstrate the bacteria-associated diseases affecting human health. The focus of this present study is to measure and compare the composition and relative abundance of the bacterial population inhabiting: (1) the oral cavity and esophagus; (2) three distinct oral niches including the saliva, tongue dorsum and supragingival plaque; (3) three distinct esophagus segments including upper, middle and lower esophagus, in a population with high-risk of esophagus cancer.

## Materials and Methods

### Study Population

Participants were recruited for the upper digestive tract cancer screening in June 2015 in Linzhou county, China, a region with high incidence of esophageal cancer. The inclusion criteria were as follows: aged from 40 to 69 years old, local residents living in Linzhou county for at least 5 years, no contraindications for endoscopic examinations (e.g., history of allergy to iodine or lidocaine), mentally and physically competent to provide written informed consent, and no consumption of any food or beverage at least 6 hours prior to sample collection. For explorative reasons, a total of 27 dental and esophageal disease-free individuals (4 men and 23 women) were included in final analysis (Supplementary Table S1). Those dental disease-free individuals were those dentist-confirmed free of periodontal diseases and no incident caries at the time of sampling. Among some people who had a past history of caries, only those people whose caries had been filled at the time of sampling were eligible for this study. Those esophageal disease-free individuals were confirmed by endoscopic examination and then biopsy-based pathological diagnosis if necessary. This study was performed in accordance with the recommendations of the Declaration of Helsinki, Institutional Review Board approval of Cancer Hospital, Chinese Academy of Medical Sciences.

### Clinical Procedures and Sample Collection

With the signed consent from each participant, we collected the information related with socio-demographics, lifestyle, eating habits and history of antibiotics use. Subsequently, visual inspection for oral health was conducted by the dentists and then the samples were collected from the oral cavity in the order of the tongue dorsum using sterile swab, the supragingival plaque using sterile forceps and the saliva by drooling. Thereafter, each participant underwent general anesthesia prior to the endoscopic examination of esophagus. The samples were collected from the upper third, middle third and lower third along esophageal tract surface in order with new sterile head-covered brushes, respectively. Biopsy would be taken at the suspicious locations and then pathological diagnosis was made. All samples were preserved in PreservCyt solution (Hologic, Bedford, MA, United States), transported with dry ice and stored in -70°C for use.

### Quality Control

Minimization of the contamination from handling environment and adjacent tract sites was essential to accurately determine site-specific microbial compositions. Three important measures were deployed besides meticulous items such as disposable sampling devices and sterile equipment: Firstly, a covered esophageal sampling brush by a protective sheath was used so that it was threaded through the endoscope channel, deployed at the site of sampling and then re-sheathed before being retracted through the endoscope. Secondly, sample collection began with the upper third of the esophagus, followed with middle third using a new brush and ended at lower third with a new brush to avoid cross contamination along the surface of endoscope channel. Once being retracted the head of the brush enriched with bacterial cells was re-sheathed, cut by a sterile scissor, immersed into the preservation solution and sealed immediately. Finally, three brushes without samples as the negative control were exposed at the same sampling room and concurrently processed with the samples in the same batch. The amount of DNA extracted from the negative control was beyond the detection limitation of Qubit (<<0.01 ng/μL).

### DNA Extraction and 16S rRNA Gene Sequencing

Genomic DNA was extracted from each sample using a series of phenol and chloroform method. Each sample was subjected to a bead beating prior to DNA extraction using Lysing Matrix-B (MP Bio) to maximize the release of the microbial genomic DNA. The V3-V4 hypervariable region of 16S rRNA gene was amplified using the forward primer (5′-GTACTCCTACGGGAGGCAGCA-3′) and reverse primer (5′-GTGGACTACHVGGGTWTCTAAT-3′) with eight base pair barcodes (Caporaso et al., 2011a,b; Fierer et al., 2012). PCR reactions were performed using TransStart Fast PfuDNA Polymerase (TransStart^®^, TransGenBiotech, Beijing, China) with the following cycling parameters: 94°C for 3 min, followed by 23 cycles of 94°C for 30 s, 60°C for 40 s, 72°C for 60 s, and a final elongation at 72°C for 10 min. Three 16S gene amplicons for each sample were pooled and their resulting bands with a correct size on a 1% agarose gel were excised. Amplicons were further purified using Gel Extraction Kit (Omega Bio-tek, United States) and quantified with Qubit. All samples were pooled together with equal molar amount from each sample. The sequencing library was constructed using TruSeq DNA kit according to the manufacturer’s instruction (Illumina, San Diego, CA, United States). The purified library was diluted, denatured, re-diluted, mixed with PhiX (equal to 30% of final DNA amount) as described in the Illumina library preparation protocols, and then applied to an Illumina Miseq system for sequencing with the Reagent Kit v3 600 cycles (Illumina, San Diego, CA, United States) as described in the manufacturer’s manual.

### OTU Picking

Raw sequencing data were demultiplexed, quality filtered, denoised, and then clear overlap paired end reads were joined together using fastq-join program. The 16S rRNA operational taxonomic units (OTUs) were clustered using “open-reference OTU” of QIIME (Caporaso et al., 2010). In this open-reference OTU picking process, reads were firstly clustered with reference to the Greengenes database (Release 13.8) using closed-reference OTUs picking. Subsequently, 0.1% of the reads which failed to hit the reference sequence collection were randomly subsampled and clustered de novo using UCLUST (v1.2.22q), with an OTU cluster defined by a sequence similarity of 97%. Chimeric sequences were removed using PYNAST (v1.2.2). OTUs were rarefied at a depth of 5,598 sequences. The operational taxonomic units (OTUs) were assigned to taxa (domain, kingdom, phylum, class, order, family, genus and species) by matching to the Greengenes database.

### Phylogenetic Diversity Analysis and Statistical Analysis

Bacterial compositions and relative abundances at each taxonomic level in each specimen, were measured. Alpha diversity was estimated for each sample using the Chao1 richness (representing the community richness), Shannon’s Diversity and Simpson Index (representing the community diversity) and then plotted using Origin 7.5. Statistical difference of the alpha diversity between two groups was determined by Student’s *t*-tests and that among three or more groups by one-way ANOVA tests followed by Dunnet’s test for multiple comparisons. Beta diversity was estimated by the Bray-Curtis distance and visualized by box plot. The difference of microbial spectrum between the oral cavity and the esophagus was revealed by the principal coordinate analysis (PCoA). PCoA was generated with QIIME platform based on the Bray-Curtis distance of OTU profile to ordinate dissimilarity matrices, in which complex dimensionality of the database indicating the beta diversity was reduced to its compositions of greatest variation. All statistical tests were performed using SPSS 18.0 with two-sided 0.05 as the significance level.

Linear discriminant effect size analysis (LEfSe) (Segata et al., 2011) based on the non-parametric factorial Kruskal–Wallis test was performed using the default parameters at any taxonomic level to find microbial biomarkers for the oral cavity and the esophagus. The threshold on the linear discriminant analysis (LDA) score for discriminative biomarkers was 2.0. All statistical analyses were conducted using R 3.1.1.

## Results

### Composition of Microbial Community in the Oral Cavity and the Esophagus

**FIGURE 1:**
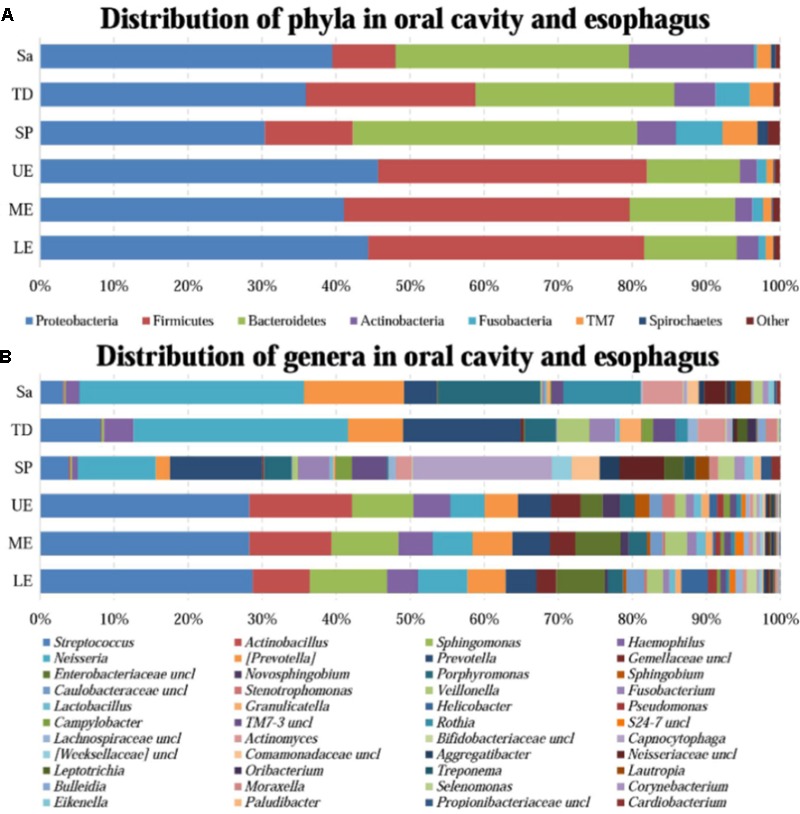
The relative abundance of human microbiome in the oral cavity and esophagus. **(A)** At the phylum level. **(B)** At the genus level. Sa, saliva; TD, tongue dorsum; SP, supragingival plaque; UE, upper esophagus; ME, middle esophagus; LE, lower esophagus.

A great diversity of microbial community was observed in the oral cavity and the esophagus (**Figure 1** and Supplementary Table S2). A total of 365 genera belonging to 29 phyla of bacteria in the oral cavity and 594 genera belonging to 29 phyla of bacteria in the esophagus were found. The most abundant microbiome at the phylum level predominant in both the oral cavity and esophagus were: Proteobacteria, Firmicutes, Bacteroidetes, Actinobacteria, Fusobacteria and TM7. Compared with the oral cavity, the esophagus had an increased relative abundance of Proteobacteria (43.6 ± 22.7% vs. 35.3 ± 15.4%, *p* < 0.001) and Firmicutes (37.4 ± 20.8% vs. 14.5 ± 10.3%, *p* < 0.001), and decreased abundance of Bacteroidetes (13.2 ± 8.6% vs. 32.2 ± 11.3%, *p* < 0.01), Actinobacteria (2.5 ± 2.0% vs. 9.3 ± 9.3%, *p* < 0.01), Fusobacteria (1.2 ± 0.9% vs. 3.8 ± 3.3%, *p* < 0.05) and TM7 (1.1 ± 1.4% vs. 3.3 ± 3.4%, *p* < 0.01).

At the genus level, the bacteria in both the oral cavity and the esopagus were characterized by high relative abundances of *Streptococcus, Neisseria, Prevotella, Actinobacillus*, and *Veillonella* (**Figure 1B** and Supplementary Table S2). *Neisseria* was predominant in the oral cavity, while *Streptococcus* in the esophagus. In addition, the bacteria with each relative abundance of over 1% were *Prevotella, Porphyromonas, Capnocytophaga, Streptococcus*, and *Rothia* in the oral cavity, and *Actinobacillus, Sphingomonas, Neisseria, Haemophilus*, and *Prevotella* in the esophagus.

An interesting finding was that the phyla TM7 and Spirochaetes had low relative abundance but high prevalence in both the oral cavity and the esophagus. TM7 was detected in at least one of three sampling sites of the oral cavity of all subjects and in the esophagus of 96.7% subjects. Spirochaetes were detected in at least one of three sampling sites in the oral cavity of 95.0% subjects and in the esophagus of 85.0% subjects (Supplementary Table S3).

### Distinct Microbial Preference in the Oral Cavity and the Esophagus

**FIGURE 2:**
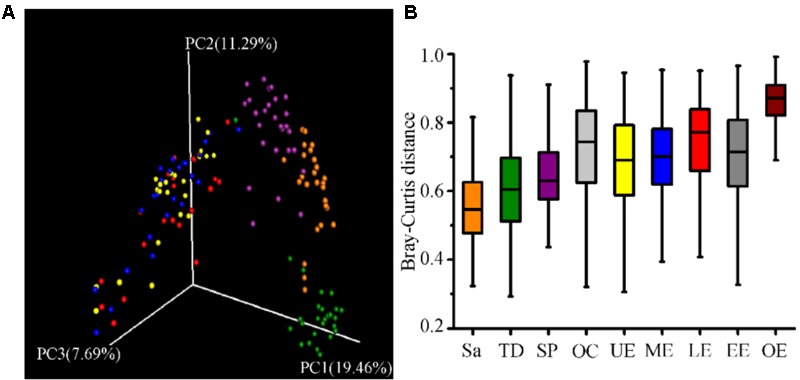
The similarity of community structure within and between the oral cavity and esophagus. **(A)** Three-dimensional ordination of human microbial profiles by principal coordinate analysis (PCoA) of average Bray-Curtis index in different body sites. Red indicating LE, Blue indicating ME, Brown indicating Sa, Green indicating SP, Purple indicating TD, and Yellow indicating UE. **(B)** The similarity of microbial diversity in three sites of the esophagus estimated by Bray-Curtis index. Values expressed as the median and quartitle of Bray-Curtis index. Sa, saliva; TD, tongue dorsum; SP, supragingival plaque; OC, oral cavity; UE, upper esophagus; ME, middle esophagus; LE, lower esophagus; EE, entire esophagus; OE, oral cavity and esophagus.

**FIGURE 3:**
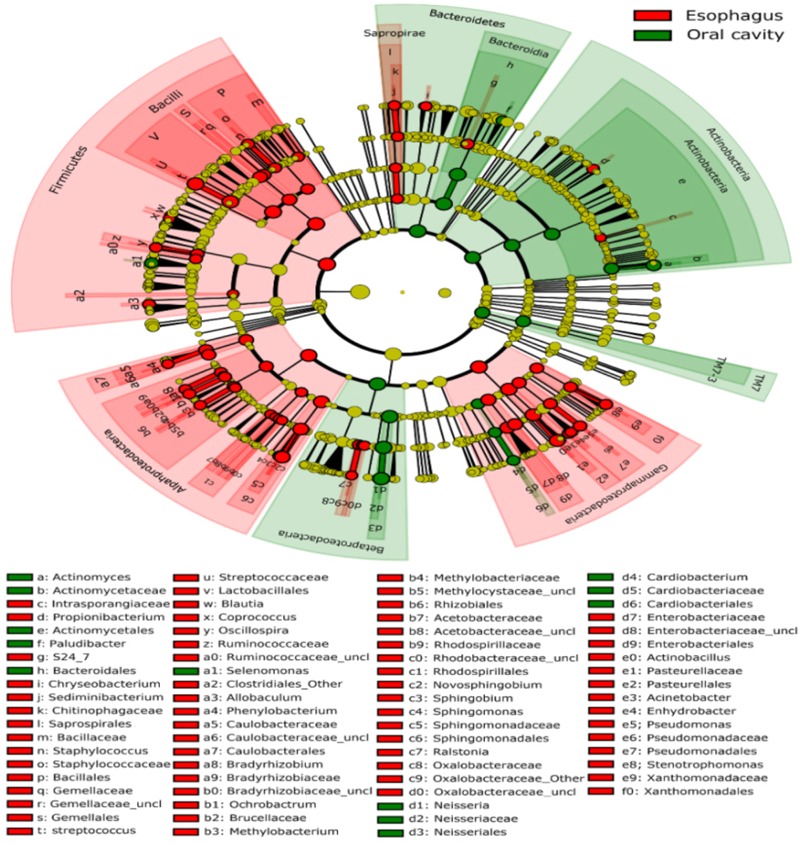
Circular cladogram for niche specialization of microbial compositions in the oral cavity and esophagus using the linear discriminant analysis effect size (LEfSe) analysis of the abundance patterns of bacterial taxa. The circles used in this diagram represent the taxonomic categories of organisms from the phylum level as the outermost circle to genus (or species) level as the innermost cycle. Within each given taxon, each small cycle represents its lower taxon. The yellow nodes indicate no statistically significant differences of specific taxa between the samples from the oral cavity and the esophagus, the red nodes indicate significantly higher relative abundance in the esophagus than in the oral cavity, and the green nodes indicate significantly higher relative abundance in the oral cavity than the esophagus. The size of the node is in proportion to the linear discriminant analysis (LDA) score (detailed in Supplementary Figure S1). The links (lines) between the nodes mean hypothetically phylogenetic relationships among organisms, which can be traced back to where the lines branch off (hypothetical ancestor).

All samples from the oral cavity were significantly clustered together in PCoA plots based on the Bray-Curtis distance of microbial composition, but not the case for those from the esophagus, indicating lower variability of microbial composition in the oral cavity than that in the esophagus (**Figure 2A,B**, *p* < 0.001). Many microbial taxa significantly differed between the oral cavity and esophagus with LDA score >2 using LEfse analysis (**Figure 3** and Supplementary Figure S1). Both the phyla Actinobacteria and Bacteroidetes were more abundant in the oral cavity than those in the esophagus. By contrast, most taxa within the phylum Firmicutes such as the class of Bacilli and Alpha proteobacteria and most taxa within the phylum Proteobacteria such as the class Gamma proteobacteria were consistently more abundant in the esophagus than that in the oral cavity (**Figure 1**, all *p* < 0.05). Notably, the phylum TM7, originally thought to be exclusively environmental was present in any site of the oral cavity at greatly higher relative abundance than that in the esophagus (3.3 ± 3.6% vs. 1.1 ± 1.4%, *p* < 0.05) (**Figures 1, 3** and Supplementary Table S2).

**FIGURE 4:**
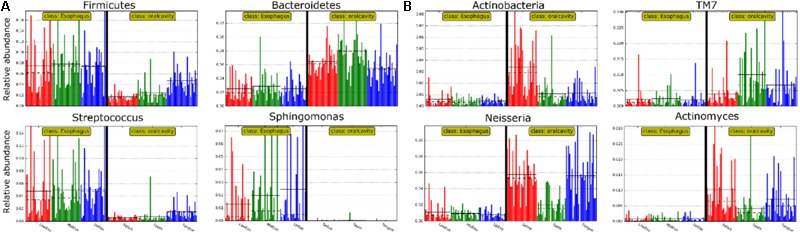
Relative abundance of most predominant discriminative microbiota between the oral cavity and the esophagus in terms of the phylum level **(A)** and the genus level **(B)**.

LEfSe analysis identified 37 genus-level signatures whose relative abundance significantly differed between the samples from the oral cavity and esophagus (**Figures 1, 4**). Genus *Neisseria* of the phylum Proteobacteria was apparently more abundant in the oral cavity than that in the esophagus (21.9 ± 15.0% vs. 4.8 ± 4.1%, *p* < 0.001), so did *Actinomyces* of the phylum Actinobacteria (3.5 ± 3.4% vs. 0.5 ± 0.5%, *p* < 0.001). The genus *Streptococcus* of the phylum Firmicutes and the genera *Actinobacillus* and *Sphingomonas* belonging to the phylum Proteobacteria could be considered as the genus-level biomarkers with significantly higher abundance in the esophagus than that in the oral cavity (*p* < 0.001). Another genus-level biomarker for the esophagus detected at >1% on the relative abundance was *Novosphingobium*, but its relative abundance much less in the oral cavity (**Figures 3, 4** and Supplementary Table S2).

### Microbial Ecological Diversity in the Oral Cavity and the Esophagus

Phylogenetic difference of microbiome between the oral cavity and esophagus extended to several ecological statistics including alpha diversity estimated by Chao1 richness, Shannon’s Diversity and Simpson Index (**Table 1**) and beta diversity estimated by the Bray-Curtis distances (**Figure 2A**). Microbial profiles from esophageal specimens were statistically less diverse than those from oral specimens estimated by Shannon Diversity and Simpson Index (*p* < 0.001). However, no similar trends were found when the Chao1 richness was used (**Table 1**). These distinctions of alpha diversity in the level of OTU between the oral cavity and the esophagus reflected their physiological anatomic characteristics. Notably, after adjusting the beta diversity for each pair of samples using Bray-Curtis distances, within-group distance in each site was significantly lower than between-group distance between the oral cavity and the esophagus (**Figure 2B**, both *p* < 0.05).

**Table 1:** Within-community alpha diversity of microbiome in the oral cavity and the esophagus.

Body habitats	Chao1 richness				Shannon Diversity				Simpson Index			
	Min	Max	Mean	*SD*	Min	Max	Mean	*SD*	Min	Max	Mean	*SD*
Oral cavity	291.18	1404.04	593.90	261.99	4.16	7.50	5.89	0.74	0.87	0.99	0.95	0.03
Sa	320.87	1355.30	645.79	289.93	4.16	6.92	5.69	0.63	0.87	0.98	0.94	0.03
TD	291.18	1085.69	535.37	264.48	4.44	6.19	5.49	0.53	0.87	0.97	0.94	0.03
SP	335.58	1404.04	600.79	223.76	4.98	7.50	6.53	0.59	0.88	0.99	0.97	0.02
Esophagus	237.50	1830.54	636.79	400.65	2.70	7.42	5.30	1.12	0.53	0.99	0.88	0.10
UE	247.24	1409.90	643.83	341.39	2.74	6.67	5.24	1.05	0.56	0.97	0.88	0.10
ME	237.50	1830.54	622.39	409.65	3.55	7.33	5.33	1.01	0.61	0.98	0.89	0.09
LE	238.91	1638.12	646.03	458.71	2.70	7.42	5.33	1.30	0.53	0.99	0.88	0.12

*Min, minimal; Max, maximal; SD, standard deviation; Sa, saliva; TD, tongue dorsum; SP, supragingival plaque; UE, upper esophagus; ME, middle esophagus; LE, lower esophagus.*

### Notable Preference of Microbial Communities for Three Sites of the Oral Cavity

**FIGURE 5:**
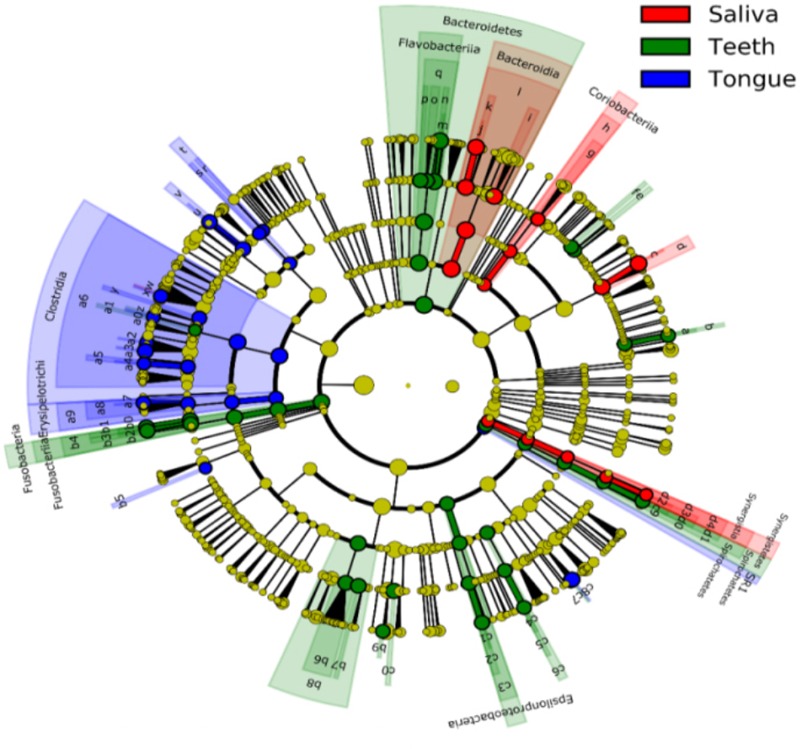
Circular cladogram for niche specialization of microbial compositions in three sites in the oral cavity using the linear discriminant analysis effect size (LEfSe) analysis of the abundance patterns of bacterial taxa. The circles used in this diagram represent the taxonomic categories of organisms from the phylum level as the outermost circle to genus (or species) level as the innermost cycle. Within each given taxon, each small cycle represents its lower clade. The yellow nodes indicate no statistically significant differences of a given taxon between the samples of three sites, the red nodes indicate significantly higher relative abundance in saliva than other two sites, the green nodes indicate significantly higher relative abundance in the supragingival plagues than other two sites, and the blue nodes indicate significantly higher relative abundance in tongue dorsum than other two sites in oral cavity. The size of the node is in proportion to the LDA score. The links (lines) between the nodes mean hypothetically phylogenetic relationships among organisms, which can be traced back to where the lines branch off (hypothetical ancestor). Sa, saliva; TD, tongue dorsum; SP, supragingival plaque.

Bacterial communities differed by the sites of the oral cavity (**Figure 5** and Supplementary Table S2). The class Bacteroides of the phylum Bacteroidetes appeared to be more abundant in the saliva. The class Clostridia from the phylum Firmicutes was preferable in the tongue dorsum. All taxa of the phylum TM7 and the class Flavobacteria of the phylum Bacteroidetes were prone to reside in the supragingival plaque.

At the genus level, *Neisseria* was more represented in the saliva and the tongue dorsum (28.4 ± 11.6% and 27.3 ± 16.5% respectively) than the supragingival plaque (9.7 ± 7.7%). *Porphyromonas, Rothia*, and *Actinomyces* were considerably abundant in the saliva. *Streptococcus, Veillonella, Haemophilus, Granulicatella, Oribacterium*, and *Bulleidia* dominated the tongue dorsum. The genus *Capnocytophaga* was well represented in the supragingival plaque but undetectable in the saliva and tongue dorsum. In contrast, the genus *Fusobacterium* accounted for higher relative abundance in the tongue dorsum and supragingival plaque than that in the saliva.

Regarding alpha diversity, Shannon Diversity and Simpson Index revealed that microbial profiles in the specimens from both the saliva and the tongue dorsum were statistically less diverse compared to those from the supragingival plaque, but no apparent differences among three sites measured by the Chao1 richness (*p* < 0.001, **Table 1**).

### No Distinct Microbial Preference for Three Segments of the Esophagus

Microbial compositions from three anatomically separated segments along the esophageal tract were examined to identify segment-specific bacteria. LEfSe analysis did not discern any microbial difference among the upper third, middle third and lower third of esophagus at any clade level (Supplementary Table S2, S4). Ecological summary statistics of both alpha diversity and beta diversity were employed to further analyze the difference of microbial abundance (**Figure 2B** and **Table 1**). No apparent clustering pattern was observed for the samples from three esophagus segments weighted by the Bray-Curtis distance of beta diversity (**Figure 2A**), and also no statistical differences of alpha diversity estimated by Shannon Diversity and Simpson Index (**Table 1**). Furthermore, within-group distance was comparable to between-group distance for all three habitats, indicating that the inter-individual variability of microbial diversity in the esophagus was so wide that the between-group diversity of microbes was covered.

## Discussion

We conducted a baseline study in a healthy population from a high-incidence region of esophageal cancer in China to demonstrate the contiguity and preference of microbiota in three niches of the oral cavity and three segments of the esophagus by 16S rRNA gene sequencing. Highly diverse bacterial flora with hundreds of genera belonging to 29 phyla resided in the oral cavity and the esophagus. Six most abundant phyla in both upper digestive tracts were Proteobacteria, Firmicutes, Bacteroidetes, Actinobacteria, Fusobacteria, and TM7. Meanwhile, several site-specific bacterial biomarkers both at the phylum and genus levels for the oral cavity and the esophagus were identified.

This investigation corroborated the diversity and complexity of bacterial profiles in the oral cavity reported by previous findings (Aas et al., 2005; Keijser et al., 2008; Bik et al., 2010; Segata et al., 2012; Norder Grusell et al., 2013), in which the phyla Proteobacteria, Firmicutes, Bacteroidetes, Actinobacteria, Fusobacteria, and TM7 were frequently detected and the genera *Neisseria, Prevotella, Porphyromonas, Capnocytophaga, Streptococcus, Rothia*, and *Actinomyces* were most predominant. The similarity of microbial compositions among three niches in the oral cavity coincided with their spatial continuity. The saliva is directly exposed to the ingested and inhaled substances, the tongue dorsum provides soft tissues medium for the dynamic bacterial flora and the supragingival plaque bathed in the saliva offers a hard tissue medium for the transitory reservoir of bacterial flora. These specific microenvironments in three oral niches might lead to microbial preference for the colonizing medium (Nasidze et al., 2009; Segata et al., 2012). Our results showed that the phylum Bacteroidetes apparently preferred to colonizing on the tooth surfaces than colonizing in the saliva or the supragingival plague, consistent with Segas’s findings (Segata et al., 2012). The phylum TM7, originally thought to be exclusively environmental, was detected in healthy oral cavity (Kelsen et al., 2015; Ren et al., 2016; Xiao et al., 2016). In our study, TM7 apparently has a predilection for the supragingival plaque, indicating that ‘environmental’ phyla once resided in the oral cavity would select suitable anatomic niches and create microbial environment to conversely influence the ecology of these habitats (Aas et al., 2005). Microbial differences from three oral habitats in healthy individuals at the genus level were further analyzed in our study. The genus *Neisseria* appeared to be transient because it was more abundant in the tongue dorsum and the saliva than that in the supragingival plaque. The genus *Capnocytophaga* was a predominant composition of biofilm in the supragingival plaque, but undetectable in the saliva and the tongue dorsum, indicating the possibility of its involvement in the periodontal and systematic diseases in the immunocompromised and immunocompetent hosts (Bonatti et al., 2003; Piau et al., 2013). The findings of site-specific bacteria in the oral cavity have been reported by Aas et al. (2005) who utilized 16S PCR cloning molecular technique to analyze microbial compositions in nine sites of oral cavity from five clinically healthy subjects. Meanwhile, the US HMP study has re-categorized nine distinct mouth surfaces into three distinct community groups based on phylogenetic relationships of bacterial communities using 16S rRNA gene sequencing (Segata et al., 2012). Microbial preference to the oral niches might be a result of specific adhesions of bacterial surface binding to complementary specific receptors on a given oral surface (Gibbons et al., 1976; Gibbons, 1989).

Healthy esophagus was colonized by its own residential bacteria (Pei et al., 2004; Norder Grusell et al., 2013). Six predominant phylum-level bacteria in the esophagus identified by our study are generally in consistent with the findings in the distal esophagus using the same 16S rRNA gene sequencing technique (Pei et al., 2004), more diverse than those found in the studies using culture-based technique (Norder Grusell et al., 2013). However, our findings in esophageal microbiota at the genus level diverge from previous studies. Apart from the predominance of *Streptococcus*, other twelve members of genera found by Pei et al. (2004) were of different abundances. For instances, the top two common bacteria in the esophagus were *Prevotella* and *Veillonella*, in combination, accounting for 30% of the bacterial taxa in their studies compared with approximately 5% in our study. *Gemella* and *Clostridium* presented in all subjects in their studies while in 30% of subjects in our study. *Actinobacillus* was not the predominant bacteria in their study but the second most common in our study. It might be explained by the populations residing in different geographical regions, having different life styles and different sampling methods. Besides, sex proportions could be also an impact factor. Four subjects including three males and one females, were recruited by Pei et al. (2005). while 27 subjects including four males and 23 females in our study. The divergence in gut microbiota in relation to age, gender, and geographic regions has been evaluated (Mueller et al., 2006; Haro et al., 2016). The last, more microbial diversity identified in brushes samples than the biopsies could also explain the difference between our study and Pei Z’s study. Whatever, it is still uncertain which method or technique is suitable for specimen collection of the upper digestive tract.

Another intriguing finding is that *Sphingomonas* was present at least one site of the oral cavity and esophageal segments. The presence of this bacterium in human samples is normally related to contamination during the handling or DNA extraction process in samples with low bacterial load, where the contribution of water-associated or environmental bacteria is potentially higher. Although we did not sequence the negative controls, the small DNA amounts in these controls (<0.01 ng/μL) in relation to the DNA amounts in our samples (2–30 ng/μL) makes us think that the relative contribution of contaminants is low. In addition, recent studies also reported that it is an opportunistic pathogen of concern in drinking water and might cause the Bacteremia in patient with sickle cell disease and in a patient with cancer (Angelakis et al., 2009; Garcia-Lozano et al., 2015; Gulati et al., 2016). Therefore, its role in healthy upper digestive tract remains to be determined in future studies.

As reported by global esophageal cancer collaboration groups, the occurrence rates of esophageal cancer varied by specific esophageal segments (Rice et al., 2009). Considering the exposure of the reflux acid contents from the stomach in distal esophagus, we tried to investigate the differences among indigenous biota from three distinct esophageal segments. However, this study did not identify any segment-specific microbiota of healthy esophagus, which suggests that the samples from any one of esophagus segments are representative for investigating the microbiome. This finding might simplify the procedure of sampling esophagus and the resulting reduction of costs, especially for esophageal cancer screening. Nevertheless, given the bacterial compositions in abnormal esophagus different from those in healthy esophagus (Pei et al., 2005; Yang et al., 2009; Liu et al., 2013), the conclusion of random sampling at any segment should be generalized to the population with high-risk of esophagus cancer or other patients cautiously.

The esophagus may not only be colonized by the bacteria from the oral cavity but also its own specific residential bacteria. Our study confirmed the overall similarity of microbial biota at each clade level between the oral cavity and the esophagus, indicating that the oral microbiota might significantly contribute to the microbiota of down-stream digestive tract including intestine (Dal Bello and Hertel, 2006; Maukonen et al., 2008; Segata et al., 2012). However, certain bacteria, such as the genera *Neisseria, Prevotella, Capnocytophaga*, and *Porphyromonas*, were detected highly abundant in the oral cavity but moderately abundant in the esophagus where genus *Streptococcus* had pronounced predominance. These divergences could be explained by either selective passage of bacteria from the oropharynx or the selective retention of particular oral bacteria by the esophagus (Segata et al., 2012). Furthermore, similar bacteria from the samples between the esophagus and the stomach were also found if microbial compositions in the esophagus in our study were linked with those in the stomach reported by other studies (Hsieh et al., 2018), despite no direct comparison of the microbial communities between the samples of esophagus and stomach at the same individual to date.

The present study has several strengths in stringent inclusion criteria of the participants confirmed by the dentists and physicians aided with esophageal endoscopy to avoid the bias of disease misclassification, and a series of quality control methods for the minimization of the contamination of microbiota from handling environment and adjacent tracts. However, a few of drawbacks still need to be addressed. Firstly, small sample size of healthy individuals limited the evaluation of the effects of social-demographic characteristics on microbial diversity. Secondly, microbiome diversity at the specie-level in high phylogenetic resolution couldn’t be reached by 16S rRNA gene sequencing (Sundquist et al., 2007), compared to whole genome shotgun sequencing. Thirdly, most participants enrolled in our study were women, which might lead to microbial bias in term of sex-relevance. Finally, since this study just focused on healthy subjects, not on both patients of esophageal cancer and healthy subjects correspondingly, the association between microbial microbiome and esophageal cancer needs to be investigated in future studies.

## Conclusion

Our study demonstrated microbial diversity at different taxonomic level in healthy oral cavity and esophagus, and identified site-preferable bacterial signatures in six niches of the upper digestive tract. We also found microbial differences between individuals caused by different genetic background and lifestyles. Further investigations with consideration of demographics variation including gender, age, health condition and life-styles are likely to comprehensively deepen our understanding of microbial characteristics in healthy population and to elucidate the disease-associated microbiota in local and systemic diseases affecting human health.

## Author Contributions

W-QW, WC, and LD designed this study. LD and JY wrote the manuscript. JY, WC, LD, and JZ cleaned, analyzed, and interpreted the data. W-QW, LD, S-RM, H-RW, and MW collected and detected clinical samples. All authors revised the manuscript and approved the final manuscript.

## Conflict of Interest Statement

The authors declare that the research was conducted in the absence of any commercial or financial relationships that could be construed as a potential conflict of interest.
