# Supplementary material for: Microbial Similarity and Preference for Specific Sites in Healthy Oral Cavity and Esophagus
Source: Front Microbiol. 2018 Jul 17;9:1603. doi: 10.3389/fmicb.2018.01603 (PMC6056649; doi:10.3389/fmicb.2018.01603)
Supplement: Supplementary file 1 [file Table_1.pdf]

Table S1 Socio-demographic characteristics of 27 healthy individual

Characteristics	N	%
**Total**	27	
**Age (years)**		55.63 ± 1.725
**Sex**		
Male	4	14.8
Female	23	85.2
**Family history of esophageal cancer**		
No	10	37
Yes	17	63
**Marital status**		
Merried	23	85.2
divorced	0	0
widowed	4	14.8
**Education level**		
Not attend school	2	7.4
Elementary school	15	55.6
Middle school	8	29.6
High school	2	7.4
**Medication History**		
Never	15	55.6
Antibiotics	2	7.4
Others	10	37
**Current smoking**		
No	26	96.3
Yes	1	3.7
**Alcohol drinking**		
No	27	100
Yes	0	0
**Tea drinking**		
No	27	100
Yes	0	0
**Lifestyle**		
Deep well water	27	100
**Fresh vegtables**		
never	0	0
rarely	0	0
often	27	100
**Fresh fruits**		
never	0	0
rarely	24	88.9
often	3	11.1
**Meet egg and milk**		
never	0	0
rarely	18	66.7
often	9	33.3
**Soy food**		
never	0	0
rarely	25	92.6
often	2	7.4
**Pickled food**		
never	0	0
rarely	27	100
often	0	0
**Fried food**		
never	0	0
rarely	27	100
often	0	0
**Hot food**		
never	22	81.5
rarely	0	0
often	5	18.5
**Mouldy food**		
never	24	88.9
rarely	3	11.1
often	0	0
