# Supplementary material for: Microbial Similarity and Preference for Specific Sites in Healthy Oral Cavity and Esophagus
Source: Front Microbiol. 2018 Jul 17;9:1603. doi: 10.3389/fmicb.2018.01603 (PMC6056649; doi:10.3389/fmicb.2018.01603)
Supplement: Supplementary file 2 [file Table_2.pdf]

Table S2 Average relative abundance, expressed in percentage of all baterial clades detected by 16S rRNA gene sequencing in body habitats from the oral cavity and esophagus

Microbial clades	Oral Cavity								Esophagus							
	All sites		Sa		TD		SP		All sites		UE		ME		LE	
	Ave	SD	Ave	SD	Ave	SD	Ave	SD	Ave	SD	Ave	SD	Ave	SD	Ave	SD
Archaea	0.00	0.00	0.00	0.00	0.00	0.00	0.00	0.00	0.00	0.00	0.00	0.00	0.00	0.00	0.00	0.00
Bacteria	100.00	100.00	100.00	100.00	100.00	100.00	100.00	100.00	100.00	100.00	100.00	100.00	100.00	100.00	####	####
unclassified	0.24	0.41	0.27	0.40	0.22	0.49	0.22	0.33	0.26	0.46	0.20	0.35	0.28	0.54	0.29	0.47
Bacteria|[Thermi]	0.00	0.00	0.00	0.00	0.00	0.00	0.00	0.00	0.03	0.09	0.02	0.05	0.05	0.15	0.02	0.03
Bacteria|Acidobacteria	0.00	0.00	0.00	0.00	0.00	0.00	0.00	0.00	0.04	0.08	0.04	0.07	0.03	0.07	0.05	0.09
Bacteria|Actinobacteria	9.26	9.27	16.85	10.94	5.54	4.47	5.24	5.66	2.53	2.04	2.35	1.37	2.32	1.60	2.96	2.90
Bacteria|Armatimonadetes	0.00	0.00	0.00	0.00	0.00	0.00	0.00	0.00	0.02	0.04	0.02	0.04	0.01	0.03	0.03	0.04
Bacteria|Bacteroidetes	32.20	11.43	31.56	8.39	26.86	10.64	38.41	12.28	13.17	8.63	12.59	7.86	14.26	9.68	12.51	8.37
Bacteria|BRC1	0.00	0.00	0.00	0.00	0.00	0.00	0.00	0.00	0.00	0.00	0.00	0.00	0.00	0.00	0.00	0.00
Bacteria|Chlamydiae	0.00	0.00	0.00	0.00	0.00	0.00	0.00	0.00	0.00	0.00	0.00	0.00	0.00	0.01	0.00	0.00
Bacteria|Chlorobi	0.00	0.01	0.00	0.00	0.00	0.00	0.00	0.02	0.00	0.00	0.00	0.00	0.00	0.00	0.00	0.00
Bacteria|Chloroflexi	0.00	0.00	0.00	0.00	0.00	0.00	0.00	0.00	0.01	0.07	0.00	0.01	0.02	0.11	0.00	0.02
Bacteria|Cyanobacteria	0.00	0.01	0.00	0.00	0.00	0.00	0.01	0.02	0.02	0.03	0.01	0.03	0.01	0.03	0.02	0.02
Bacteria|Elusimicrobia	0.01	0.02	0.00	0.01	0.00	0.00	0.02	0.04	0.00	0.00	0.00	0.00	0.00	0.00	0.00	0.00
Bacteria|Fibrobacteres	0.00	0.00	0.00	0.00	0.00	0.00	0.00	0.00	0.00	0.00	0.00	0.00	0.00	0.00	0.00	0.00
Bacteria|Firmicutes	14.48	10.26	8.53	3.76	22.95	10.66	11.87	8.76	37.42	20.83	36.27	19.93	38.59	19.83	37.25	23.53
Bacteria|Fusobacteria	3.76	3.36	0.42	0.39	4.65	2.77	6.31	2.81	1.22	0.90	1.25	0.85	1.43	1.16	0.95	0.48
Bacteria|Gemmatimonadetes	0.00	0.00	0.00	0.00	0.00	0.00	0.00	0.00	0.00	0.00	0.00	0.01	0.00	0.00	0.00	0.00
Bacteria|GN02	0.30	0.81	0.03	0.07	0.13	0.41	0.78	1.24	0.08	0.18	0.15	0.28	0.04	0.09	0.06	0.08
Bacteria|Lentisphaerae	0.00	0.00	0.00	0.00	0.00	0.00	0.00	0.00	0.00	0.00	0.00	0.00	0.00	0.00	0.00	0.00
Bacteria|Planctomycetes	0.00	0.00	0.00	0.00	0.00	0.00	0.00	0.00	0.00	0.00	0.00	0.00	0.00	0.00	0.00	0.00
Bacteria|Proteobacteria	35.34	15.36	39.51	12.49	35.93	18.71	30.41	13.22	43.61	22.66	45.70	23.49	41.09	21.66	44.37	23.65
Bacteria|Spirochaetes	0.70	1.10	0.62	0.45	0.13	0.12	1.38	1.67	0.17	0.19	0.17	0.23	0.18	0.19	0.14	0.15
Bacteria|SR1	0.17	0.44	0.02	0.02	0.30	0.73	0.18	0.12	0.08	0.24	0.07	0.13	0.05	0.10	0.13	0.40
Bacteria|Synergistetes	0.12	0.22	0.25	0.31	0.00	0.01	0.10	0.13	0.01	0.01	0.01	0.01	0.01	0.01	0.01	0.02

Microbial clades	Oral Cavity								Esophagus							
	All sites		Sa		TD		SP		All sites		UE		ME		LE	
	Ave	SD	Ave	SD	Ave	SD	Ave	SD	Ave	SD	Ave	SD	Ave	SD	Ave	SD
Bacteria|Tenericutes	0.15	0.33	0.04	0.07	0.10	0.11	0.33	0.52	0.17	0.33	0.11	0.21	0.26	0.47	0.12	0.21
Bacteria|Thermotogae	0.00	0.00	0.00	0.00	0.00	0.00	0.00	0.00	0.00	0.01	0.00	0.00	0.00	0.00	0.00	0.02
Bacteria|TM6	0.00	0.00	0.00	0.00	0.00	0.00	0.00	0.00	0.00	0.01	0.00	0.00	0.00	0.00	0.00	0.01
Bacteria|TM7	3.25	3.59	1.88	1.96	3.20	3.43	4.73	4.49	1.06	1.35	0.95	1.34	1.18	1.18	1.05	1.57
Bacteria|Verrucomicrobia	0.00	0.00	0.00	0.00	0.00	0.00	0.00	0.00	0.08	0.30	0.07	0.13	0.15	0.47	0.03	0.12
Bacteria|WPS-2	0.00	0.00	0.00	0.00	0.00	0.00	0.00	0.00	0.02	0.13	0.00	0.01	0.04	0.21	0.00	0.00
Bacteria|WS6	0.01	0.03	0.00	0.00	0.00	0.00	0.02	0.06	0.00	0.01	0.00	0.00	0.00	0.00	0.00	0.01
Bacteria|[Thermi]|Deinococci	0.00	0.00	0.00	0.00	0.00	0.00	0.00	0.00	0.03	0.09	0.02	0.05	0.05	0.15	0.02	0.03
Bacteria|Acidobacteria|[Chloracidobacteria	0.00	0.00	0.00	0.00	0.00	0.00	0.00	0.00	0.00	0.00	0.00	0.00	0.00	0.00	0.00	0.00
Bacteria|Acidobacteria|Acidobacteria-6	0.00	0.00	0.00	0.00	0.00	0.00	0.00	0.00	0.00	0.01	0.00	0.01	0.00	0.00	0.00	0.01
Bacteria|Acidobacteria|Acidobacteriia	0.00	0.00	0.00	0.00	0.00	0.00	0.00	0.00	0.00	0.00	0.00	0.00	0.00	0.01	0.00	0.01
Bacteria|Acidobacteria|Holophagae	0.00	0.00	0.00	0.00	0.00	0.00	0.00	0.00	0.00	0.00	0.00	0.00	0.00	0.00	0.00	0.00
Bacteria|Acidobacteria|Solibacteres	0.00	0.00	0.00	0.00	0.00	0.00	0.00	0.00	0.04	0.08	0.04	0.07	0.03	0.07	0.05	0.09
Bacteria|Acidobacteria|Sva0725	0.00	0.00	0.00	0.00	0.00	0.00	0.00	0.00	0.00	0.00	0.00	0.00	0.00	0.00	0.00	0.00
Bacteria|Actinobacteria|Acidimicrobiia	0.00	0.00	0.00	0.00	0.00	0.01	0.00	0.00	0.01	0.04	0.00	0.00	0.02	0.07	0.00	0.00
Bacteria|Actinobacteria|Actinobacteria	8.92	8.93	16.22	10.51	5.23	4.24	5.19	5.65	2.43	1.91	2.26	1.30	2.22	1.52	2.83	2.67
Bacteria|Actinobacteria|Coriobacteriia	0.34	0.49	0.64	0.66	0.31	0.34	0.05	0.11	0.10	0.19	0.09	0.15	0.09	0.15	0.13	0.26
Bacteria|Actinobacteria|Nitriliruptoria	0.00	0.00	0.00	0.00	0.00	0.00	0.00	0.00	0.00	0.00	0.00	0.00	0.00	0.00	0.00	0.00
Bacteria|Actinobacteria|Rubrobacteria	0.00	0.00	0.00	0.00	0.00	0.00	0.00	0.00	0.00	0.00	0.00	0.00	0.00	0.00	0.00	0.00
Bacteria|Actinobacteria|Thermoleophilia	0.00	0.00	0.00	0.00	0.00	0.01	0.00	0.00	0.00	0.01	0.00	0.00	0.00	0.00	0.00	0.01
Bacteria|Armatimonadetes|[Fimbriimonadi	0.00	0.00	0.00	0.00	0.00	0.00	0.00	0.00	0.02	0.04	0.02	0.04	0.01	0.03	0.03	0.04
Bacteria|Armatimonadetes|Armatimonadia	0.00	0.00	0.00	0.00	0.00	0.00	0.00	0.00	0.00	0.01	0.00	0.02	0.00	0.00	0.00	0.00
Bacteria|Bacteroidetes|[Saprospirae]	0.01	0.02	0.01	0.04	0.00	0.01	0.00	0.01	0.33	0.94	0.23	0.18	0.25	0.32	0.53	1.62
Bacteria|Bacteroidetes|Bacteroidia	25.40	11.47	31.05	8.24	26.09	10.69	18.80	12.09	11.61	8.67	10.67	7.98	13.03	9.74	10.96	8.20
Bacteria|Bacteroidetes|Cytophagia	0.00	0.00	0.00	0.00	0.00	0.00	0.00	0.01	0.02	0.05	0.03	0.06	0.01	0.04	0.01	0.03
Bacteria|Bacteroidetes|Flavobacteriia	6.79	11.41	0.50	0.45	0.75	0.68	19.59	12.58	0.97	0.81	0.95	0.68	0.96	0.64	1.00	1.08
Bacteria|Bacteroidetes|Sphingobacteriia	0.01	0.04	0.00	0.01	0.02	0.06	0.01	0.03	0.23	1.21	0.70	2.10	0.02	0.05	0.01	0.03
Bacteria|BRC1|PRR-11	0.00	0.00	0.00	0.00	0.00	0.00	0.00	0.00	0.00	0.00	0.00	0.00	0.00	0.00	0.00	0.00
Bacteria|Chlamydiae|Chlamydiia	0.00	0.00	0.00	0.00	0.00	0.00	0.00	0.00	0.00	0.00	0.00	0.00	0.00	0.01	0.00	0.00

Microbial clades	Oral Cavity								Esophagus							
	All sites		Sa		TD		SP		All sites		UE		ME		LE	
	Ave	SD	Ave	SD	Ave	SD	Ave	SD	Ave	SD	Ave	SD	Ave	SD	Ave	SD
Bacteria|Chlorobi|Ignavibacteria	0.00	0.00	0.00	0.00	0.00	0.00	0.00	0.00	0.00	0.00	0.00	0.00	0.00	0.00	0.00	0.00
Bacteria|Chlorobi|OPB56	0.00	0.01	0.00	0.00	0.00	0.00	0.00	0.02	0.00	0.00	0.00	0.00	0.00	0.00	0.00	0.00
Bacteria|Chloroflexi|Anaerolineae	0.00	0.00	0.00	0.00	0.00	0.00	0.00	0.00	0.00	0.01	0.00	0.00	0.00	0.00	0.00	0.02
Bacteria|Chloroflexi|C0119	0.00	0.00	0.00	0.00	0.00	0.00	0.00	0.00	0.01	0.07	0.00	0.00	0.02	0.11	0.00	0.00
Bacteria|Chloroflexi|Ellin6529	0.00	0.00	0.00	0.00	0.00	0.00	0.00	0.00	0.00	0.00	0.00	0.00	0.00	0.00	0.00	0.00
Bacteria|Chloroflexi|S085	0.00	0.00	0.00	0.00	0.00	0.00	0.00	0.00	0.00	0.00	0.00	0.00	0.00	0.00	0.00	0.00
Bacteria|Chloroflexi|Thermomicrobia	0.00	0.00	0.00	0.00	0.00	0.00	0.00	0.00	0.00	0.01	0.00	0.01	0.00	0.00	0.00	0.00
Bacteria|Chloroflexi|TK17	0.00	0.00	0.00	0.00	0.00	0.00	0.00	0.00	0.00	0.00	0.00	0.00	0.00	0.00	0.00	0.00
Bacteria|Cyanobacteria|4C0d-2	0.00	0.00	0.00	0.00	0.00	0.00	0.00	0.00	0.00	0.01	0.00	0.01	0.00	0.01	0.01	0.01
Bacteria|Cyanobacteria|Chloroplast	0.00	0.01	0.00	0.00	0.00	0.00	0.01	0.02	0.01	0.02	0.01	0.03	0.00	0.01	0.01	0.02
Bacteria|Cyanobacteria|ML635J-21	0.00	0.00	0.00	0.00	0.00	0.00	0.00	0.00	0.00	0.00	0.00	0.00	0.00	0.00	0.00	0.00
Bacteria|Cyanobacteria|Oscillatoriophycide	0.00	0.00	0.00	0.00	0.00	0.00	0.00	0.00	0.00	0.02	0.00	0.00	0.01	0.03	0.00	0.01
Bacteria|Elusimicrobia|Elusimicrobia	0.00	0.02	0.00	0.01	0.00	0.00	0.01	0.03	0.00	0.00	0.00	0.00	0.00	0.00	0.00	0.00
Bacteria|Elusimicrobia|Endomicrobia	0.00	0.01	0.00	0.00	0.00	0.00	0.01	0.02	0.00	0.00	0.00	0.00	0.00	0.00	0.00	0.00
Bacteria|Fibrobacteres|Fibrobacteria	0.00	0.00	0.00	0.00	0.00	0.00	0.00	0.00	0.00	0.00	0.00	0.00	0.00	0.00	0.00	0.00
Bacteria|Firmicutes|Bacilli	6.77	6.80	3.85	1.59	11.68	6.85	4.71	7.43	30.58	20.61	31.29	19.58	31.12	19.33	29.27	23.68
Bacteria|Firmicutes|Clostridia	7.26	5.23	4.43	2.43	10.26	5.52	7.08	5.49	6.33	8.49	4.72	3.46	7.00	9.80	7.19	10.36
Bacteria|Firmicutes|Erysipelotrichi	0.45	0.62	0.25	0.23	1.01	0.79	0.08	0.09	0.50	1.16	0.25	0.25	0.47	0.82	0.79	1.83
Bacteria|Fusobacteria|Fusobacteriia	3.76	3.36	0.42	0.39	4.65	2.77	6.31	2.81	1.22	0.90	1.25	0.85	1.43	1.16	0.95	0.48
Bacteria|Gemmatimonadetes|Gemmatimon	0.00	0.00	0.00	0.00	0.00	0.00	0.00	0.00	0.00	0.00	0.00	0.01	0.00	0.00	0.00	0.00
Bacteria|GN02|BD1-5	0.30	0.81	0.03	0.07	0.13	0.41	0.78	1.24	0.08	0.18	0.15	0.28	0.04	0.09	0.06	0.08
Bacteria|Lentisphaerae|[Lentisphaeria]	0.00	0.00	0.00	0.00	0.00	0.00	0.00	0.00	0.00	0.00	0.00	0.00	0.00	0.00	0.00	0.00
Bacteria|Planctomycetes|OM190	0.00	0.00	0.00	0.00	0.00	0.00	0.00	0.00	0.00	0.00	0.00	0.00	0.00	0.00	0.00	0.00
Bacteria|Planctomycetes|Phycisphaerae	0.00	0.00	0.00	0.00	0.00	0.00	0.00	0.00	0.00	0.00	0.00	0.00	0.00	0.00	0.00	0.00
Bacteria|Planctomycetes|Planctomycetia	0.00	0.00	0.00	0.00	0.00	0.00	0.00	0.00	0.00	0.00	0.00	0.00	0.00	0.00	0.00	0.00
Bacteria|Proteobacteria|Alphaproteobacteri	0.37	0.79	0.24	0.63	0.55	1.06	0.32	0.61	13.87	20.48	14.71	22.73	12.82	19.11	14.21	20.49
Bacteria|Proteobacteria|Betaproteobacteria	28.68	14.51	35.63	12.06	28.09	16.55	22.09	11.48	6.74	4.82	6.01	3.27	6.10	3.40	8.20	6.92
Bacteria|Proteobacteria|Deltaproteobacteri	0.11	0.21	0.17	0.24	0.01	0.01	0.17	0.25	0.08	0.13	0.09	0.16	0.06	0.08	0.08	0.16
Bacteria|Proteobacteria|Epsilonproteobacte	1.20	1.24	0.14	0.07	1.45	1.31	2.05	1.01	1.90	4.70	1.66	3.44	0.80	0.91	3.38	7.40

Microbial clades	Oral Cavity								Esophagus							
	All sites		Sa		TD		SP		All sites		UE		ME		LE	
	Ave	SD	Ave	SD	Ave	SD	Ave	SD	Ave	SD	Ave	SD	Ave	SD	Ave	SD
Bacteria|Proteobacteria|Gammaproteobacte	4.97	4.65	3.35	2.08	5.84	6.43	5.77	4.06	21.02	15.83	23.22	19.87	21.30	15.02	18.49	12.09
Bacteria|Proteobacteria|TA18	0.00	0.00	0.00	0.00	0.00	0.00	0.00	0.00	0.00	0.00	0.00	0.00	0.00	0.00	0.00	0.00
Bacteria|Spirochaetes|GN05	0.00	0.00	0.00	0.00	0.00	0.00	0.00	0.01	0.00	0.00	0.00	0.00	0.00	0.00	0.00	0.00
Bacteria|Spirochaetes|Spirochaetes	0.70	1.10	0.62	0.45	0.13	0.12	1.38	1.67	0.17	0.19	0.17	0.23	0.18	0.19	0.14	0.15
Bacteria|Synergistetes|Synergistia	0.12	0.22	0.25	0.31	0.00	0.01	0.10	0.13	0.01	0.01	0.01	0.01	0.01	0.01	0.01	0.02
Bacteria|Tenericutes|CK-1C4-19	0.00	0.00	0.00	0.00	0.00	0.00	0.00	0.00	0.03	0.28	0.00	0.00	0.09	0.46	0.00	0.00
Bacteria|Tenericutes|Mollicutes	0.13	0.27	0.04	0.06	0.10	0.11	0.25	0.43	0.13	0.20	0.11	0.21	0.17	0.19	0.12	0.21
Bacteria|Tenericutes|RF3	0.03	0.11	0.00	0.01	0.00	0.01	0.08	0.17	0.00	0.01	0.00	0.00	0.00	0.01	0.00	0.01
Bacteria|Thermotogae|Thermotogae	0.00	0.00	0.00	0.00	0.00	0.00	0.00	0.00	0.00	0.01	0.00	0.00	0.00	0.00	0.00	0.02
Bacteria|TM6|SBRH58	0.00	0.00	0.00	0.00	0.00	0.00	0.00	0.00	0.00	0.01	0.00	0.00	0.00	0.00	0.00	0.01
Bacteria|TM6|SJA-4	0.00	0.00	0.00	0.00	0.00	0.00	0.00	0.00	0.00	0.00	0.00	0.00	0.00	0.00	0.00	0.01
Bacteria|TM7|SC3	0.00	0.00	0.00	0.00	0.00	0.00	0.00	0.00	0.00	0.00	0.00	0.00	0.00	0.00	0.00	0.00
Bacteria|TM7|TM7-1	0.00	0.00	0.00	0.00	0.00	0.00	0.00	0.00	0.00	0.00	0.00	0.01	0.00	0.00	0.00	0.00
Bacteria|TM7|TM7-3	3.25	3.59	1.88	1.96	3.20	3.43	4.73	4.49	1.06	1.35	0.95	1.34	1.18	1.18	1.05	1.57
Bacteria|Verrucomicrobia|Verruco-5	0.00	0.00	0.00	0.00	0.00	0.00	0.00	0.00	0.00	0.00	0.00	0.00	0.00	0.00	0.00	0.00
Bacteria|Verrucomicrobia|Verrucomicrobia	0.00	0.00	0.00	0.00	0.00	0.00	0.00	0.00	0.08	0.30	0.07	0.13	0.15	0.47	0.03	0.12
Bacteria|WS6|SC72	0.01	0.03	0.00	0.00	0.00	0.00	0.02	0.06	0.00	0.01	0.00	0.00	0.00	0.00	0.00	0.01
Bacteria|[Thermi]|Deinococci|Deinococcal	0.00	0.00	0.00	0.00	0.00	0.00	0.00	0.00	0.03	0.09	0.02	0.05	0.04	0.15	0.02	0.03
Bacteria|[Thermi]|Deinococci|Thermales	0.00	0.00	0.00	0.00	0.00	0.00	0.00	0.00	0.00	0.01	0.00	0.00	0.00	0.00	0.00	0.02
Bacteria|Acidobacteria|[Chloracidobacteria	0.00	0.00	0.00	0.00	0.00	0.00	0.00	0.00	0.00	0.00	0.00	0.00	0.00	0.00	0.00	0.00
Bacteria|Acidobacteria|Acidobacteria-6|iii1	0.00	0.00	0.00	0.00	0.00	0.00	0.00	0.00	0.00	0.01	0.00	0.01	0.00	0.00	0.00	0.01
Bacteria|Acidobacteria|Acidobacteriia|Acic	0.00	0.00	0.00	0.00	0.00	0.00	0.00	0.00	0.00	0.00	0.00	0.00	0.00	0.01	0.00	0.01
Bacteria|Acidobacteria|Holophagae|Holopl	0.00	0.00	0.00	0.00	0.00	0.00	0.00	0.00	0.00	0.00	0.00	0.00	0.00	0.00	0.00	0.00
Bacteria|Acidobacteria|Solibacteres|Soliba	0.00	0.00	0.00	0.00	0.00	0.00	0.00	0.00	0.04	0.08	0.04	0.07	0.03	0.07	0.05	0.09
Bacteria|Acidobacteria|Sva0725|Sva0725	0.00	0.00	0.00	0.00	0.00	0.00	0.00	0.00	0.00	0.00	0.00	0.00	0.00	0.00	0.00	0.00
Bacteria|Actinobacteria|Acidimicrobiia|Aci	0.00	0.00	0.00	0.00	0.00	0.01	0.00	0.00	0.01	0.04	0.00	0.00	0.02	0.07	0.00	0.00
Bacteria|Actinobacteria|Actinobacteria|Act	8.65	8.91	15.96	10.45	5.01	4.30	4.84	5.57	1.69	1.25	1.69	1.23	1.72	1.28	1.65	1.31
Bacteria|Actinobacteria|Actinobacteria|Bifi	0.27	0.67	0.25	0.41	0.22	0.38	0.35	1.03	0.74	1.66	0.57	1.26	0.50	1.03	1.19	2.39
Bacteria|Actinobacteria|Actinobacteria|Mic	0.00	0.00	0.00	0.00	0.00	0.00	0.00	0.00	0.00	0.00	0.00	0.00	0.00	0.00	0.00	0.00

Microbial clades	Oral Cavity								Esophagus							
	All sites		Sa		TD		SP		All sites		UE		ME		LE	
	Ave	SD	Ave	SD	Ave	SD	Ave	SD	Ave	SD	Ave	SD	Ave	SD	Ave	SD
Bacteria|Actinobacteria|Coriobacteriia|Cori	0.34	0.49	0.64	0.66	0.31	0.34	0.05	0.11	0.10	0.19	0.09	0.15	0.09	0.15	0.13	0.26
Bacteria|Actinobacteria|Nitriliruptoria|Nitri	0.00	0.00	0.00	0.00	0.00	0.00	0.00	0.00	0.00	0.00	0.00	0.00	0.00	0.00	0.00	0.00
Bacteria|Actinobacteria|Rubrobacteria|Rub	0.00	0.00	0.00	0.00	0.00	0.00	0.00	0.00	0.00	0.00	0.00	0.00	0.00	0.00	0.00	0.00
Bacteria|Actinobacteria|Thermoleophilia|G	0.00	0.00	0.00	0.00	0.00	0.00	0.00	0.00	0.00	0.00	0.00	0.00	0.00	0.00	0.00	0.00
Bacteria|Actinobacteria|Thermoleophilia|S	0.00	0.00	0.00	0.00	0.00	0.01	0.00	0.00	0.00	0.01	0.00	0.00	0.00	0.00	0.00	0.01
Bacteria|Armatimonadetes|[Fimbriimonadi	0.00	0.00	0.00	0.00	0.00	0.00	0.00	0.00	0.02	0.04	0.02	0.04	0.01	0.03	0.03	0.04
Bacteria|Armatimonadetes|Armatimonadia	0.00	0.00	0.00	0.00	0.00	0.00	0.00	0.00	0.00	0.01	0.00	0.02	0.00	0.00	0.00	0.00
Bacteria|Bacteroidetes|[Saprospirae]|[Sapro	0.01	0.02	0.01	0.04	0.00	0.01	0.00	0.01	0.33	0.94	0.23	0.18	0.25	0.32	0.53	1.62
Bacteria|Bacteroidetes|Bacteroidia|Bactero	25.40	11.47	31.05	8.24	26.09	10.69	18.80	12.09	11.61	8.67	10.67	7.98	13.03	9.74	10.96	8.20
Bacteria|Bacteroidetes|Cytophagia|Cytopha	0.00	0.00	0.00	0.00	0.00	0.00	0.00	0.01	0.02	0.05	0.03	0.06	0.01	0.04	0.01	0.03
Bacteria|Bacteroidetes|Flavobacteriia|Flavo	6.79	11.41	0.50	0.45	0.75	0.68	19.59	12.58	0.97	0.81	0.95	0.68	0.96	0.64	1.00	1.08
Bacteria|Bacteroidetes|Sphingobacteriia|Sp	0.01	0.04	0.00	0.01	0.02	0.06	0.01	0.03	0.23	1.21	0.70	2.10	0.02	0.05	0.01	0.03
Bacteria|Chlamydiae|Chlamydiia|Chlamyd	0.00	0.00	0.00	0.00	0.00	0.00	0.00	0.00	0.00	0.00	0.00	0.00	0.00	0.01	0.00	0.00
Bacteria|Chlorobi|Ignavibacteria|Ignavibac	0.00	0.00	0.00	0.00	0.00	0.00	0.00	0.00	0.00	0.00	0.00	0.00	0.00	0.00	0.00	0.00
Bacteria|Chloroflexi|Anaerolineae|Anaerol	0.00	0.00	0.00	0.00	0.00	0.00	0.00	0.00	0.00	0.00	0.00	0.00	0.00	0.00	0.00	0.00
Bacteria|Chloroflexi|Anaerolineae|Caldilin	0.00	0.00	0.00	0.00	0.00	0.00	0.00	0.00	0.00	0.00	0.00	0.00	0.00	0.00	0.00	0.00
Bacteria|Chloroflexi|Anaerolineae|SBR103	0.00	0.00	0.00	0.00	0.00	0.00	0.00	0.00	0.00	0.01	0.00	0.00	0.00	0.00	0.00	0.02
Bacteria|Chloroflexi|Thermomicrobia|JG30	0.00	0.00	0.00	0.00	0.00	0.00	0.00	0.00	0.00	0.01	0.00	0.01	0.00	0.00	0.00	0.00
Bacteria|Chloroflexi|TK17|mle1-48	0.00	0.00	0.00	0.00	0.00	0.00	0.00	0.00	0.00	0.00	0.00	0.00	0.00	0.00	0.00	0.00
Bacteria|Cyanobacteria|4C0d-2|MLE1-12	0.00	0.00	0.00	0.00	0.00	0.00	0.00	0.00	0.00	0.01	0.00	0.01	0.00	0.01	0.01	0.01
Bacteria|Cyanobacteria|4C0d-2|SM2F09	0.00	0.00	0.00	0.00	0.00	0.00	0.00	0.00	0.00	0.00	0.00	0.00	0.00	0.00	0.00	0.00
Bacteria|Cyanobacteria|4C0d-2|YS2	0.00	0.00	0.00	0.00	0.00	0.00	0.00	0.00	0.00	0.00	0.00	0.00	0.00	0.00	0.00	0.00
Bacteria|Cyanobacteria|Chloroplast|Strame	0.00	0.00	0.00	0.00	0.00	0.00	0.00	0.00	0.00	0.00	0.00	0.00	0.00	0.00	0.00	0.00
Bacteria|Cyanobacteria|Chloroplast|Strepto	0.00	0.01	0.00	0.00	0.00	0.00	0.01	0.02	0.01	0.02	0.01	0.03	0.00	0.01	0.01	0.02
Bacteria|Cyanobacteria|Oscillatoriophycide	0.00	0.00	0.00	0.00	0.00	0.00	0.00	0.00	0.00	0.01	0.00	0.00	0.00	0.01	0.00	0.00
Bacteria|Cyanobacteria|Oscillatoriophycide	0.00	0.00	0.00	0.00	0.00	0.00	0.00	0.00	0.00	0.02	0.00	0.00	0.01	0.03	0.00	0.01
Bacteria|Elusimicrobia|Elusimicrobia|Elusi	0.00	0.02	0.00	0.01	0.00	0.00	0.01	0.03	0.00	0.00	0.00	0.00	0.00	0.00	0.00	0.00
Bacteria|Fibrobacteres|Fibrobacteria|Fibrob	0.00	0.00	0.00	0.00	0.00	0.00	0.00	0.00	0.00	0.00	0.00	0.00	0.00	0.00	0.00	0.00
Bacteria|Firmicutes|Bacilli|Bacillales	0.03	0.08	0.01	0.02	0.03	0.06	0.04	0.13	1.04	2.54	0.68	1.01	0.89	2.24	1.57	3.69

Microbial clades	Oral Cavity								Esophagus							
	All sites		Sa		TD		SP		All sites		UE		ME		LE	
	Ave	SD	Ave	SD	Ave	SD	Ave	SD	Ave	SD	Ave	SD	Ave	SD	Ave	SD
Bacteria|Firmicutes|Bacilli|Gemellales	0.23	0.33	0.06	0.05	0.40	0.32	0.23	0.41	2.98	3.44	3.60	4.52	3.07	3.06	2.26	2.47
Bacteria|Firmicutes|Bacilli|Lactobacillales	6.52	6.56	3.78	1.58	11.25	6.76	4.44	7.02	26.56	19.98	27.01	18.17	27.16	19.18	25.43	23.19
Bacteria|Firmicutes|Bacilli|Turicibacterales	0.00	0.00	0.00	0.00	0.00	0.00	0.00	0.00	0.01	0.02	0.01	0.02	0.00	0.02	0.01	0.03
Bacteria|Firmicutes|Clostridia|Clostridiales	7.26	5.23	4.43	2.43	10.26	5.52	7.08	5.49	6.33	8.49	4.72	3.46	7.00	9.80	7.19	10.36
Bacteria|Firmicutes|Clostridia|Halanaerobi	0.00	0.00	0.00	0.00	0.00	0.00	0.00	0.00	0.00	0.00	0.00	0.00	0.00	0.00	0.00	0.00
Bacteria|Firmicutes|Clostridia|Thermoanae	0.00	0.00	0.00	0.00	0.00	0.00	0.00	0.00	0.00	0.01	0.00	0.00	0.00	0.00	0.00	0.02
Bacteria|Firmicutes|Erysipelotrichi|Erysipe	0.45	0.62	0.25	0.23	1.01	0.79	0.08	0.09	0.50	1.16	0.25	0.25	0.47	0.82	0.79	1.83
Bacteria|Fusobacteria|Fusobacteriia|Fusoba	3.76	3.36	0.42	0.39	4.65	2.77	6.31	2.81	1.22	0.90	1.25	0.85	1.43	1.16	0.95	0.48
Bacteria|Gemmatimonadetes|Gemmatimon	0.00	0.00	0.00	0.00	0.00	0.00	0.00	0.00	0.00	0.00	0.00	0.01	0.00	0.00	0.00	0.00
Bacteria|Lentisphaerae|[Lentisphaeria]|Z20	0.00	0.00	0.00	0.00	0.00	0.00	0.00	0.00	0.00	0.00	0.00	0.00	0.00	0.00	0.00	0.00
Bacteria|Planctomycetes|OM190|agg27	0.00	0.00	0.00	0.00	0.00	0.00	0.00	0.00	0.00	0.00	0.00	0.00	0.00	0.00	0.00	0.00
Bacteria|Planctomycetes|Phycisphaerae|Phy	0.00	0.00	0.00	0.00	0.00	0.00	0.00	0.00	0.00	0.00	0.00	0.00	0.00	0.00	0.00	0.00
Bacteria|Planctomycetes|Planctomycetia|G	0.00	0.00	0.00	0.00	0.00	0.00	0.00	0.00	0.00	0.00	0.00	0.00	0.00	0.00	0.00	0.00
Bacteria|Proteobacteria|Alphaproteobacteri	0.00	0.00	0.00	0.00	0.00	0.00	0.00	0.00	0.01	0.06	0.02	0.09	0.00	0.00	0.01	0.05
Bacteria|Proteobacteria|Alphaproteobacteri	0.09	0.17	0.02	0.05	0.15	0.16	0.11	0.22	2.16	3.59	1.74	1.23	1.75	1.32	3.05	6.07
Bacteria|Proteobacteria|Alphaproteobacteri	0.00	0.00	0.00	0.00	0.00	0.00	0.00	0.00	0.00	0.01	0.00	0.01	0.00	0.00	0.00	0.00
Bacteria|Proteobacteria|Alphaproteobacteri	0.00	0.00	0.00	0.00	0.00	0.00	0.00	0.00	0.00	0.00	0.00	0.00	0.00	0.00	0.00	0.00
Bacteria|Proteobacteria|Alphaproteobacteri	0.03	0.08	0.02	0.05	0.05	0.13	0.02	0.03	1.41	1.36	1.38	1.47	1.37	1.10	1.50	1.56
Bacteria|Proteobacteria|Alphaproteobacteri	0.00	0.01	0.00	0.00	0.00	0.02	0.00	0.00	0.06	0.13	0.10	0.19	0.05	0.10	0.02	0.04
Bacteria|Proteobacteria|Alphaproteobacteri	0.02	0.11	0.02	0.09	0.03	0.16	0.00	0.00	0.20	0.33	0.25	0.46	0.19	0.31	0.15	0.17
Bacteria|Proteobacteria|Alphaproteobacteri	0.00	0.00	0.00	0.00	0.00	0.00	0.00	0.00	0.00	0.01	0.00	0.01	0.00	0.01	0.01	0.01
Bacteria|Proteobacteria|Alphaproteobacteri	0.22	0.73	0.17	0.56	0.31	0.98	0.18	0.57	10.02	19.26	11.21	21.44	9.45	18.21	9.46	18.94
Bacteria|Proteobacteria|Betaproteobacteria	0.00	0.00	0.00	0.00	0.00	0.00	0.00	0.00	0.00	0.00	0.00	0.00	0.00	0.00	0.00	0.00
Bacteria|Proteobacteria|Betaproteobacteria	3.12	4.02	3.64	3.60	0.20	0.21	5.60	4.65	1.56	2.46	1.60	1.54	1.01	0.88	2.14	3.94
Bacteria|Proteobacteria|Betaproteobacteria	0.00	0.00	0.00	0.00	0.00	0.00	0.00	0.00	0.01	0.06	0.00	0.01	0.03	0.09	0.01	0.03
Bacteria|Proteobacteria|Betaproteobacteria	0.00	0.00	0.00	0.00	0.00	0.00	0.00	0.00	0.00	0.00	0.00	0.00	0.00	0.00	0.00	0.00
Bacteria|Proteobacteria|Betaproteobacteria	25.55	14.60	31.97	12.10	27.88	16.59	16.47	9.94	5.14	4.47	4.39	3.02	5.04	3.09	6.01	6.57
Bacteria|Proteobacteria|Betaproteobacteria	0.01	0.03	0.02	0.04	0.00	0.00	0.02	0.04	0.03	0.05	0.02	0.03	0.02	0.03	0.05	0.08
Bacteria|Proteobacteria|Betaproteobacteria	0.00	0.00	0.00	0.00	0.00	0.00	0.00	0.00	0.00	0.00	0.00	0.00	0.00	0.00	0.00	0.00

Microbial clades	Oral Cavity								Esophagus							
	All sites		Sa		TD		SP		All sites		UE		ME		LE	
	Ave	SD	Ave	SD	Ave	SD	Ave	SD	Ave	SD	Ave	SD	Ave	SD	Ave	SD
Bacteria|Proteobacteria|Deltaproteobacteria	0.00	0.00	0.00	0.00	0.00	0.00	0.00	0.00	0.00	0.00	0.00	0.00	0.00	0.00	0.00	0.00
Bacteria|Proteobacteria|Deltaproteobacteria	0.00	0.00	0.00	0.00	0.00	0.00	0.00	0.00	0.01	0.03	0.01	0.05	0.00	0.00	0.00	0.01
Bacteria|Proteobacteria|Deltaproteobacteria	0.03	0.08	0.04	0.04	0.00	0.01	0.06	0.12	0.00	0.02	0.00	0.00	0.01	0.01	0.01	0.03
Bacteria|Proteobacteria|Deltaproteobacteria	0.07	0.17	0.12	0.24	0.00	0.01	0.08	0.16	0.05	0.12	0.06	0.13	0.03	0.05	0.06	0.16
Bacteria|Proteobacteria|Deltaproteobacteria	0.00	0.00	0.00	0.00	0.00	0.00	0.00	0.00	0.00	0.00	0.00	0.00	0.00	0.00	0.00	0.00
Bacteria|Proteobacteria|Deltaproteobacteria	0.01	0.04	0.00	0.01	0.00	0.01	0.03	0.07	0.01	0.02	0.01	0.02	0.01	0.03	0.00	0.01
Bacteria|Proteobacteria|Deltaproteobacteria	0.00	0.00	0.00	0.00	0.00	0.00	0.00	0.00	0.00	0.00	0.00	0.00	0.00	0.00	0.00	0.00
Bacteria|Proteobacteria|Deltaproteobacteria	0.00	0.02	0.00	0.01	0.00	0.00	0.01	0.03	0.01	0.04	0.01	0.02	0.02	0.06	0.01	0.01
Bacteria|Proteobacteria|Deltaproteobacteria	0.00	0.00	0.00	0.00	0.00	0.00	0.00	0.00	0.00	0.00	0.00	0.00	0.00	0.00	0.00	0.00
Bacteria|Proteobacteria|Deltaproteobacteria	0.00	0.00	0.00	0.00	0.00	0.00	0.00	0.00	0.00	0.00	0.00	0.00	0.00	0.00	0.00	0.00
Bacteria|Proteobacteria|Epsilonproteobacte	1.20	1.24	0.14	0.07	1.45	1.31	2.05	1.01	1.90	4.70	1.66	3.44	0.80	0.91	3.38	7.40
Bacteria|Proteobacteria|Gammaproteobacte	0.01	0.03	0.01	0.04	0.00	0.01	0.00	0.01	0.02	0.04	0.02	0.05	0.01	0.02	0.02	0.05
Bacteria|Proteobacteria|Gammaproteobacte	0.00	0.00	0.00	0.00	0.00	0.00	0.00	0.00	0.01	0.06	0.00	0.01	0.02	0.08	0.02	0.05
Bacteria|Proteobacteria|Gammaproteobacte	0.61	0.94	0.55	0.50	0.03	0.04	1.28	1.30	0.02	0.06	0.02	0.04	0.01	0.02	0.03	0.09
Bacteria|Proteobacteria|Gammaproteobacte	0.12	0.35	0.11	0.22	0.17	0.55	0.07	0.13	4.58	7.26	2.62	3.14	5.46	9.16	5.53	7.68
Bacteria|Proteobacteria|Gammaproteobacte	0.00	0.00	0.00	0.00	0.00	0.00	0.00	0.00	0.02	0.04	0.00	0.01	0.02	0.05	0.02	0.05
Bacteria|Proteobacteria|Gammaproteobacte	0.00	0.00	0.00	0.00	0.00	0.00	0.00	0.00	0.02	0.12	0.00	0.01	0.04	0.19	0.03	0.09
Bacteria|Proteobacteria|Gammaproteobacte	3.31	2.94	2.54	2.07	4.08	3.33	3.32	3.16	13.77	15.88	16.98	21.12	14.08	14.37	10.23	10.44
Bacteria|Proteobacteria|Gammaproteobacte	0.91	3.45	0.12	0.17	1.55	5.67	1.07	1.75	1.63	1.94	1.49	1.62	1.29	1.04	2.16	2.80
Bacteria|Proteobacteria|Gammaproteobacte	0.00	0.00	0.00	0.00	0.00	0.00	0.00	0.00	0.00	0.00	0.00	0.00	0.00	0.00	0.00	0.00
Bacteria|Proteobacteria|Gammaproteobacte	0.00	0.00	0.00	0.00	0.00	0.00	0.00	0.00	0.00	0.00	0.00	0.00	0.00	0.00	0.00	0.00
Bacteria|Proteobacteria|Gammaproteobacte	0.02	0.05	0.01	0.03	0.01	0.02	0.03	0.08	0.95	3.44	2.09	5.98	0.37	0.36	0.45	0.43
Bacteria|Proteobacteria|TA18|CV90	0.00	0.00	0.00	0.00	0.00	0.00	0.00	0.00	0.00	0.00	0.00	0.00	0.00	0.00	0.00	0.00
Bacteria|Spirochaetes|GN05|SBYZ_6080	0.00	0.00	0.00	0.00	0.00	0.00	0.00	0.01	0.00	0.00	0.00	0.00	0.00	0.00	0.00	0.00
Bacteria|Spirochaetes|Spirochaetes|Sphaero	0.00	0.01	0.00	0.01	0.00	0.00	0.01	0.02	0.00	0.00	0.00	0.00	0.00	0.00	0.00	0.00
Bacteria|Spirochaetes|Spirochaetes|Spiroch	0.70	1.10	0.62	0.45	0.13	0.12	1.37	1.66	0.17	0.19	0.17	0.23	0.18	0.19	0.14	0.15
Bacteria|Synergistetes|Synergistia|Synergis	0.12	0.22	0.25	0.31	0.00	0.01	0.10	0.13	0.01	0.01	0.01	0.01	0.01	0.01	0.01	0.02
Bacteria|Tenericutes|Mollicutes|Acholeplas	0.05	0.20	0.00	0.01	0.01	0.03	0.13	0.34	0.03	0.13	0.01	0.01	0.05	0.14	0.04	0.17
Bacteria|Tenericutes|Mollicutes|Mycoplasm	0.05	0.10	0.03	0.06	0.03	0.05	0.09	0.16	0.09	0.16	0.09	0.21	0.10	0.13	0.06	0.13

Microbial clades	Oral Cavity								Esophagus							
	All sites		Sa		TD		SP		All sites		UE		ME		LE	
	Ave	SD	Ave	SD	Ave	SD	Ave	SD	Ave	SD	Ave	SD	Ave	SD	Ave	SD
Bacteria|Tenericutes|Mollicutes|RF39	0.03	0.06	0.00	0.01	0.05	0.08	0.02	0.04	0.02	0.04	0.01	0.02	0.03	0.05	0.01	0.02
Bacteria|Tenericutes|RF3|ML615J-28	0.03	0.11	0.00	0.01	0.00	0.01	0.08	0.17	0.00	0.01	0.00	0.00	0.00	0.01	0.00	0.01
Bacteria|Thermotogae|Thermotogae|Therm	0.00	0.00	0.00	0.00	0.00	0.00	0.00	0.00	0.00	0.01	0.00	0.00	0.00	0.00	0.00	0.02
Bacteria|TM7|TM7-3|Blgi18	0.00	0.00	0.00	0.00	0.00	0.00	0.00	0.00	0.00	0.00	0.00	0.00	0.00	0.00	0.00	0.00
Bacteria|TM7|TM7-3|CW040	0.16	0.32	0.03	0.06	0.21	0.25	0.24	0.48	0.15	0.29	0.10	0.13	0.19	0.34	0.15	0.36
Bacteria|TM7|TM7-3|EW055	0.00	0.00	0.00	0.00	0.00	0.00	0.00	0.00	0.02	0.06	0.02	0.08	0.01	0.04	0.01	0.05
Bacteria|TM7|TM7-3|I025	0.20	0.33	0.35	0.36	0.09	0.26	0.17	0.34	0.18	0.55	0.11	0.32	0.20	0.60	0.23	0.68
Bacteria|Verrucomicrobia|Verruco-5|WCH	0.00	0.00	0.00	0.00	0.00	0.00	0.00	0.00	0.00	0.00	0.00	0.00	0.00	0.00	0.00	0.00
Bacteria|Verrucomicrobia|Verrucomicrobia	0.00	0.00	0.00	0.00	0.00	0.00	0.00	0.00	0.08	0.30	0.07	0.13	0.15	0.47	0.03	0.12
Bacteria|WS6|SC72|WCHB1-15	0.01	0.03	0.00	0.00	0.00	0.00	0.02	0.06	0.00	0.01	0.00	0.00	0.00	0.00	0.00	0.01
Bacteria|[Thermi]|Deinococci|Deinococcal	0.00	0.00	0.00	0.00	0.00	0.00	0.00	0.00	0.03	0.09	0.02	0.03	0.04	0.15	0.01	0.03
Bacteria|[Thermi]|Deinococci|Deinococcal	0.00	0.00	0.00	0.00	0.00	0.00	0.00	0.00	0.00	0.01	0.01	0.02	0.00	0.00	0.00	0.01
Bacteria|[Thermi]|Deinococci|Thermales|T	0.00	0.00	0.00	0.00	0.00	0.00	0.00	0.00	0.00	0.01	0.00	0.00	0.00	0.00	0.00	0.02
Bacteria|Acidobacteria|[Chloracidobacteria	0.00	0.00	0.00	0.00	0.00	0.00	0.00	0.00	0.00	0.00	0.00	0.00	0.00	0.00	0.00	0.00
Bacteria|Acidobacteria|Acidobacteria-6|iii1	0.00	0.00	0.00	0.00	0.00	0.00	0.00	0.00	0.00	0.00	0.00	0.01	0.00	0.00	0.00	0.00
Bacteria|Acidobacteria|Acidobacteriia|Acid	0.00	0.00	0.00	0.00	0.00	0.00	0.00	0.00	0.00	0.00	0.00	0.00	0.00	0.01	0.00	0.01
Bacteria|Acidobacteria|Holophagae|Holoph	0.00	0.00	0.00	0.00	0.00	0.00	0.00	0.00	0.00	0.00	0.00	0.00	0.00	0.00	0.00	0.00
Bacteria|Actinobacteria|Acidimicrobiia|Aci	0.00	0.00	0.00	0.00	0.00	0.01	0.00	0.00	0.00	0.00	0.00	0.00	0.00	0.00	0.00	0.00
Bacteria|Actinobacteria|Actinobacteria|Act	0.00	0.00	0.00	0.00	0.00	0.00	0.00	0.00	0.00	0.00	0.00	0.00	0.00	0.00	0.00	0.01
Bacteria|Actinobacteria|Actinobacteria|Act	3.58	3.76	5.13	4.51	3.48	3.10	2.09	2.96	0.44	0.47	0.44	0.49	0.45	0.46	0.45	0.48
Bacteria|Actinobacteria|Actinobacteria|Act	0.00	0.00	0.00	0.00	0.00	0.00	0.00	0.00	0.00	0.00	0.00	0.01	0.00	0.00	0.00	0.00
Bacteria|Actinobacteria|Actinobacteria|Act	0.00	0.00	0.00	0.00	0.00	0.00	0.00	0.00	0.00	0.02	0.00	0.01	0.01	0.03	0.00	0.00
Bacteria|Actinobacteria|Actinobacteria|Act	0.65	0.91	0.72	0.67	0.02	0.03	1.25	1.15	0.11	0.17	0.09	0.11	0.11	0.21	0.12	0.16
Bacteria|Actinobacteria|Actinobacteria|Act	0.00	0.00	0.00	0.00	0.00	0.00	0.00	0.00	0.00	0.00	0.00	0.00	0.00	0.00	0.00	0.00
Bacteria|Actinobacteria|Actinobacteria|Act	0.00	0.00	0.00	0.00	0.00	0.00	0.00	0.00	0.00	0.00	0.00	0.00	0.00	0.00	0.00	0.00
Bacteria|Actinobacteria|Actinobacteria|Act	0.00	0.00	0.00	0.00	0.00	0.00	0.00	0.00	0.00	0.00	0.00	0.00	0.00	0.00	0.00	0.00
Bacteria|Actinobacteria|Actinobacteria|Act	0.00	0.00	0.00	0.00	0.00	0.00	0.00	0.00	0.00	0.00	0.00	0.00	0.00	0.00	0.00	0.00
Bacteria|Actinobacteria|Actinobacteria|Act	0.00	0.00	0.00	0.00	0.00	0.00	0.00	0.00	0.00	0.01	0.01	0.02	0.00	0.00	0.00	0.00
Bacteria|Actinobacteria|Actinobacteria|Act	0.00	0.00	0.00	0.00	0.00	0.00	0.00	0.00	0.01	0.04	0.02	0.07	0.01	0.04	0.00	0.01

Microbial clades	Oral Cavity								Esophagus							
	All sites		Sa		TD		SP		All sites		UE		ME		LE	
	Ave	SD	Ave	SD	Ave	SD	Ave	SD	Ave	SD	Ave	SD	Ave	SD	Ave	SD
Bacteria|Actinobacteria|Actinobacteria|Act	0.00	0.00	0.00	0.00	0.00	0.00	0.00	0.00	0.00	0.01	0.00	0.01	0.00	0.00	0.00	0.00
Bacteria|Actinobacteria|Actinobacteria|Act	0.00	0.00	0.00	0.00	0.00	0.00	0.00	0.00	0.06	0.16	0.03	0.05	0.09	0.18	0.06	0.19
Bacteria|Actinobacteria|Actinobacteria|Act	0.00	0.00	0.00	0.00	0.00	0.00	0.00	0.00	0.00	0.00	0.00	0.00	0.00	0.00	0.00	0.00
Bacteria|Actinobacteria|Actinobacteria|Act	0.00	0.00	0.00	0.00	0.00	0.00	0.00	0.00	0.00	0.00	0.00	0.00	0.00	0.00	0.00	0.00
Bacteria|Actinobacteria|Actinobacteria|Act	0.00	0.01	0.00	0.00	0.00	0.01	0.00	0.00	0.03	0.04	0.03	0.05	0.02	0.03	0.02	0.04
Bacteria|Actinobacteria|Actinobacteria|Act	3.87	6.18	9.77	7.50	1.49	2.05	0.21	0.27	0.55	0.67	0.71	0.94	0.51	0.47	0.44	0.52
Bacteria|Actinobacteria|Actinobacteria|Act	0.00	0.00	0.00	0.00	0.00	0.00	0.00	0.00	0.01	0.03	0.00	0.01	0.00	0.00	0.02	0.04
Bacteria|Actinobacteria|Actinobacteria|Act	0.00	0.00	0.00	0.00	0.00	0.00	0.00	0.00	0.00	0.01	0.00	0.00	0.00	0.02	0.00	0.00
Bacteria|Actinobacteria|Actinobacteria|Act	0.00	0.00	0.00	0.00	0.00	0.00	0.00	0.00	0.03	0.05	0.02	0.03	0.02	0.04	0.04	0.08
Bacteria|Actinobacteria|Actinobacteria|Act	0.00	0.01	0.00	0.00	0.00	0.01	0.01	0.02	0.03	0.11	0.06	0.19	0.01	0.02	0.01	0.03
Bacteria|Actinobacteria|Actinobacteria|Act	0.00	0.00	0.00	0.00	0.00	0.00	0.00	0.00	0.00	0.01	0.00	0.01	0.00	0.01	0.00	0.02
Bacteria|Actinobacteria|Actinobacteria|Act	0.00	0.00	0.00	0.00	0.00	0.00	0.00	0.00	0.00	0.00	0.00	0.00	0.00	0.00	0.00	0.01
Bacteria|Actinobacteria|Actinobacteria|Act	0.54	1.39	0.35	0.37	0.01	0.01	1.28	2.26	0.28	0.46	0.20	0.25	0.33	0.49	0.30	0.58
Bacteria|Actinobacteria|Actinobacteria|Act	0.00	0.00	0.00	0.00	0.00	0.00	0.00	0.00	0.01	0.04	0.00	0.00	0.02	0.06	0.01	0.03
Bacteria|Actinobacteria|Actinobacteria|Act	0.00	0.00	0.00	0.00	0.00	0.00	0.00	0.00	0.00	0.02	0.01	0.04	0.00	0.00	0.00	0.00
Bacteria|Actinobacteria|Actinobacteria|Act	0.00	0.00	0.00	0.00	0.00	0.00	0.00	0.00	0.00	0.00	0.00	0.00	0.00	0.00	0.00	0.00
Bacteria|Actinobacteria|Actinobacteria|Act	0.00	0.00	0.00	0.00	0.00	0.00	0.00	0.00	0.11	0.27	0.04	0.10	0.14	0.29	0.15	0.36
Bacteria|Actinobacteria|Actinobacteria|Act	0.00	0.00	0.00	0.00	0.00	0.00	0.00	0.00	0.00	0.00	0.00	0.00	0.00	0.00	0.00	0.00
Bacteria|Actinobacteria|Actinobacteria|Act	0.00	0.00	0.00	0.00	0.00	0.00	0.00	0.00	0.00	0.01	0.00	0.00	0.00	0.00	0.00	0.01
Bacteria|Actinobacteria|Actinobacteria|Bifi	0.27	0.67	0.25	0.41	0.22	0.38	0.35	1.03	0.74	1.66	0.57	1.26	0.50	1.03	1.19	2.39
Bacteria|Actinobacteria|Coriobacteriia|Cori	0.34	0.49	0.64	0.66	0.31	0.34	0.05	0.11	0.10	0.19	0.09	0.15	0.09	0.15	0.13	0.26
Bacteria|Actinobacteria|Nitriliruptoria|Nitri	0.00	0.00	0.00	0.00	0.00	0.00	0.00	0.00	0.00	0.00	0.00	0.00	0.00	0.00	0.00	0.00
Bacteria|Actinobacteria|Rubrobacteria|Rub	0.00	0.00	0.00	0.00	0.00	0.00	0.00	0.00	0.00	0.00	0.00	0.00	0.00	0.00	0.00	0.00
Bacteria|Actinobacteria|Thermoleophilia|So	0.00	0.00	0.00	0.00	0.00	0.00	0.00	0.00	0.00	0.01	0.00	0.00	0.00	0.00	0.00	0.01
Bacteria|Actinobacteria|Thermoleophilia|So	0.00	0.00	0.00	0.00	0.00	0.00	0.00	0.00	0.00	0.00	0.00	0.00	0.00	0.00	0.00	0.00
Bacteria|Armatimonadetes|[Fimbriimonadi	0.00	0.00	0.00	0.00	0.00	0.00	0.00	0.00	0.02	0.04	0.02	0.04	0.01	0.03	0.03	0.04
Bacteria|Armatimonadetes|Armatimonadia	0.00	0.00	0.00	0.00	0.00	0.00	0.00	0.00	0.00	0.01	0.00	0.02	0.00	0.00	0.00	0.00
Bacteria|Bacteroidetes|[Saprospirae]|[Sapro	0.01	0.02	0.01	0.04	0.00	0.01	0.00	0.01	0.33	0.92	0.23	0.18	0.25	0.32	0.52	1.59
Bacteria|Bacteroidetes|[Saprospirae]|[Sapro	0.00	0.00	0.00	0.00	0.00	0.00	0.00	0.00	0.00	0.02	0.00	0.00	0.00	0.00	0.01	0.03

Microbial clades	Oral Cavity								Esophagus							
	All sites		Sa		TD		SP		All sites		UE		ME		LE	
	Ave	SD	Ave	SD	Ave	SD	Ave	SD	Ave	SD	Ave	SD	Ave	SD	Ave	SD
Bacteria|Bacteroidetes|Bacteroidia|Bactero	0.00	0.00	0.00	0.00	0.00	0.00	0.00	0.00	0.00	0.00	0.00	0.00	0.00	0.00	0.00	0.00
Bacteria|Bacteroidetes|Bacteroidia|Bactero	0.01	0.04	0.00	0.01	0.00	0.00	0.01	0.07	0.01	0.06	0.00	0.01	0.02	0.07	0.02	0.07
Bacteria|Bacteroidetes|Bacteroidia|Bactero	7.20	5.66	12.65	4.64	6.99	3.63	1.75	1.52	4.35	5.29	3.99	3.62	4.73	6.83	4.28	4.90
Bacteria|Bacteroidetes|Bacteroidia|Bactero	0.07	0.20	0.12	0.31	0.01	0.02	0.08	0.16	0.41	0.81	0.27	0.47	0.40	0.70	0.56	1.14
Bacteria|Bacteroidetes|Bacteroidia|Bactero	0.02	0.09	0.05	0.11	0.01	0.01	0.02	0.09	0.02	0.07	0.02	0.09	0.02	0.09	0.01	0.02
Bacteria|Bacteroidetes|Bacteroidia|Bactero	0.00	0.01	0.00	0.00	0.00	0.01	0.00	0.00	0.00	0.00	0.00	0.00	0.00	0.01	0.00	0.00
Bacteria|Bacteroidetes|Bacteroidia|Bactero	7.54	6.88	13.46	8.06	4.05	4.22	5.02	2.40	2.02	1.77	1.89	1.73	2.25	2.07	1.88	1.46
Bacteria|Bacteroidetes|Bacteroidia|Bactero	10.16	9.75	4.16	2.72	14.99	10.84	11.37	10.25	3.99	4.72	3.93	4.91	4.50	5.11	3.47	4.20
Bacteria|Bacteroidetes|Bacteroidia|Bactero	0.00	0.01	0.00	0.00	0.00	0.00	0.00	0.02	0.00	0.01	0.00	0.00	0.00	0.01	0.00	0.00
Bacteria|Bacteroidetes|Bacteroidia|Bactero	0.00	0.02	0.01	0.03	0.00	0.01	0.00	0.01	0.03	0.08	0.03	0.07	0.03	0.10	0.04	0.07
Bacteria|Bacteroidetes|Bacteroidia|Bactero	0.01	0.01	0.01	0.01	0.01	0.01	0.01	0.01	0.74	1.95	0.50	0.71	1.03	2.75	0.65	1.72
Bacteria|Bacteroidetes|Cytophagia|Cytopha	0.00	0.00	0.00	0.00	0.00	0.00	0.00	0.00	0.00	0.00	0.00	0.00	0.00	0.00	0.00	0.00
Bacteria|Bacteroidetes|Cytophagia|Cytopha	0.00	0.00	0.00	0.00	0.00	0.00	0.00	0.01	0.02	0.04	0.03	0.06	0.01	0.04	0.00	0.01
Bacteria|Bacteroidetes|Flavobacteriia|Flavo	0.86	2.19	0.07	0.16	0.11	0.11	2.46	3.34	0.56	0.68	0.55	0.50	0.46	0.37	0.68	1.03
Bacteria|Bacteroidetes|Flavobacteriia|Flavo	0.00	0.00	0.00	0.00	0.00	0.00	0.00	0.00	0.00	0.00	0.00	0.00	0.00	0.00	0.00	0.00
Bacteria|Bacteroidetes|Flavobacteriia|Flavo	5.92	10.35	0.43	0.34	0.64	0.63	17.12	12.02	0.41	0.43	0.40	0.37	0.50	0.56	0.32	0.31
Bacteria|Bacteroidetes|Sphingobacteriia|Sp	0.01	0.04	0.00	0.01	0.02	0.06	0.01	0.03	0.23	1.21	0.70	2.10	0.01	0.01	0.01	0.03
Bacteria|Chlamydiae|Chlamydiia|Chlamyd	0.00	0.00	0.00	0.00	0.00	0.00	0.00	0.00	0.00	0.00	0.00	0.00	0.00	0.01	0.00	0.00
Bacteria|Chlamydiae|Chlamydiia|Chlamyd	0.00	0.00	0.00	0.00	0.00	0.00	0.00	0.00	0.00	0.00	0.00	0.00	0.00	0.00	0.00	0.00
Bacteria|Chloroflexi|Anaerolineae|Anaerol	0.00	0.00	0.00	0.00	0.00	0.00	0.00	0.00	0.00	0.00	0.00	0.00	0.00	0.00	0.00	0.00
Bacteria|Chloroflexi|Anaerolineae|Caldilin	0.00	0.00	0.00	0.00	0.00	0.00	0.00	0.00	0.00	0.00	0.00	0.00	0.00	0.00	0.00	0.00
Bacteria|Chloroflexi|Anaerolineae|SBR103	0.00	0.00	0.00	0.00	0.00	0.00	0.00	0.00	0.00	0.01	0.00	0.00	0.00	0.00	0.00	0.02
Bacteria|Cyanobacteria|Oscillatoriophycide	0.00	0.00	0.00	0.00	0.00	0.00	0.00	0.00	0.00	0.01	0.00	0.00	0.00	0.01	0.00	0.00
Bacteria|Cyanobacteria|Oscillatoriophycide	0.00	0.00	0.00	0.00	0.00	0.00	0.00	0.00	0.00	0.02	0.00	0.00	0.01	0.03	0.00	0.01
Bacteria|Fibrobacteres|Fibrobacteria|Fibrob	0.00	0.00	0.00	0.00	0.00	0.00	0.00	0.00	0.00	0.00	0.00	0.00	0.00	0.00	0.00	0.00
Bacteria|Firmicutes|Bacilli|Bacillales|[Exig	0.00	0.00	0.00	0.00	0.00	0.00	0.00	0.00	0.00	0.01	0.00	0.02	0.00	0.01	0.00	0.00
Bacteria|Firmicutes|Bacilli|Bacillales|[Ther	0.00	0.00	0.00	0.00	0.00	0.00	0.00	0.00	0.00	0.00	0.00	0.00	0.00	0.00	0.00	0.00
Bacteria|Firmicutes|Bacilli|Bacillales|Alicy	0.00	0.00	0.00	0.00	0.00	0.00	0.00	0.00	0.00	0.00	0.00	0.00	0.00	0.00	0.00	0.00
Bacteria|Firmicutes|Bacilli|Bacillales|Bacil	0.02	0.04	0.01	0.02	0.02	0.05	0.02	0.04	0.27	0.60	0.36	0.89	0.18	0.29	0.30	0.51

Microbial clades	Oral Cavity								Esophagus							
	All sites		Sa		TD		SP		All sites		UE		ME		LE	
	Ave	SD	Ave	SD	Ave	SD	Ave	SD	Ave	SD	Ave	SD	Ave	SD	Ave	SD
Bacteria|Firmicutes|Bacilli|Bacillales|Lister	0.00	0.00	0.00	0.00	0.00	0.00	0.00	0.00	0.00	0.01	0.00	0.00	0.00	0.01	0.00	0.00
Bacteria|Firmicutes|Bacilli|Bacillales|Paeni	0.00	0.00	0.00	0.00	0.00	0.00	0.00	0.00	0.27	1.96	0.04	0.12	0.04	0.15	0.75	3.46
Bacteria|Firmicutes|Bacilli|Bacillales|Planc	0.00	0.00	0.00	0.00	0.00	0.00	0.00	0.00	0.01	0.02	0.01	0.01	0.01	0.02	0.01	0.03
Bacteria|Firmicutes|Bacilli|Bacillales|Sporo	0.00	0.00	0.00	0.00	0.00	0.00	0.00	0.00	0.00	0.00	0.00	0.00	0.00	0.00	0.00	0.00
Bacteria|Firmicutes|Bacilli|Bacillales|Staph	0.01	0.05	0.00	0.00	0.00	0.00	0.02	0.09	0.48	1.45	0.26	0.33	0.65	2.22	0.50	0.98
Bacteria|Firmicutes|Bacilli|Bacillales|Therr	0.00	0.00	0.00	0.00	0.00	0.00	0.00	0.00	0.00	0.00	0.00	0.00	0.00	0.00	0.00	0.00
Bacteria|Firmicutes|Bacilli|Gemellales|Gen	0.23	0.33	0.06	0.05	0.40	0.32	0.23	0.41	2.97	3.44	3.60	4.52	3.07	3.05	2.24	2.48
Bacteria|Firmicutes|Bacilli|Lactobacillales|	0.14	0.20	0.12	0.18	0.13	0.24	0.17	0.18	0.17	0.40	0.14	0.28	0.25	0.59	0.12	0.18
Bacteria|Firmicutes|Bacilli|Lactobacillales|	1.18	1.59	0.45	0.28	2.76	1.85	0.29	0.51	0.81	0.71	0.93	0.84	0.84	0.65	0.66	0.62
Bacteria|Firmicutes|Bacilli|Lactobacillales|	0.00	0.01	0.00	0.01	0.00	0.01	0.00	0.00	0.05	0.11	0.02	0.03	0.06	0.13	0.07	0.14
Bacteria|Firmicutes|Bacilli|Lactobacillales|	0.42	0.88	0.28	0.55	0.57	1.16	0.40	0.84	0.91	1.59	0.94	1.70	1.10	1.86	0.68	1.09
Bacteria|Firmicutes|Bacilli|Lactobacillales|	0.00	0.00	0.00	0.00	0.00	0.01	0.00	0.00	0.01	0.03	0.00	0.00	0.01	0.05	0.00	0.01
Bacteria|Firmicutes|Bacilli|Lactobacillales|	4.77	5.40	2.93	1.51	7.77	5.60	3.58	6.52	24.59	19.70	24.97	17.93	24.89	18.96	23.88	22.83
Bacteria|Firmicutes|Bacilli|Turicibacterales	0.00	0.00	0.00	0.00	0.00	0.00	0.00	0.00	0.01	0.02	0.01	0.02	0.00	0.02	0.01	0.03
Bacteria|Firmicutes|Clostridia|Clostridiales	0.00	0.00	0.00	0.00	0.00	0.00	0.00	0.00	0.00	0.00	0.00	0.00	0.00	0.00	0.00	0.00
Bacteria|Firmicutes|Clostridia|Clostridiales	0.60	0.66	0.22	0.13	0.99	0.66	0.60	0.77	0.30	0.29	0.29	0.26	0.33	0.33	0.28	0.27
Bacteria|Firmicutes|Clostridia|Clostridiales	0.16	0.33	0.06	0.08	0.24	0.44	0.18	0.32	0.13	0.20	0.11	0.13	0.12	0.15	0.16	0.28
Bacteria|Firmicutes|Clostridia|Clostridiales	0.00	0.00	0.00	0.00	0.00	0.00	0.00	0.00	0.11	0.36	0.07	0.18	0.13	0.46	0.13	0.38
Bacteria|Firmicutes|Clostridia|Clostridiales	0.02	0.06	0.01	0.04	0.03	0.08	0.02	0.06	0.15	0.39	0.08	0.09	0.10	0.21	0.26	0.65
Bacteria|Firmicutes|Clostridia|Clostridiales	0.00	0.00	0.00	0.00	0.00	0.00	0.00	0.00	0.00	0.01	0.00	0.01	0.00	0.00	0.00	0.00
Bacteria|Firmicutes|Clostridia|Clostridiales	0.05	0.36	0.01	0.02	0.01	0.04	0.13	0.64	0.00	0.01	0.01	0.02	0.00	0.00	0.00	0.01
Bacteria|Firmicutes|Clostridia|Clostridiales	1.81	1.57	1.13	0.66	2.81	2.08	1.48	1.04	1.82	3.11	1.10	0.83	1.93	2.83	2.42	4.54
Bacteria|Firmicutes|Clostridia|Clostridiales	0.04	0.07	0.02	0.01	0.07	0.11	0.02	0.02	0.02	0.03	0.02	0.04	0.01	0.03	0.01	0.02
Bacteria|Firmicutes|Clostridia|Clostridiales	0.39	0.47	0.08	0.07	0.45	0.35	0.66	0.63	0.28	0.31	0.26	0.25	0.29	0.40	0.28	0.27
Bacteria|Firmicutes|Clostridia|Clostridiales	0.06	0.26	0.11	0.41	0.04	0.12	0.02	0.09	0.80	1.41	0.68	1.04	0.74	1.30	0.98	1.82
Bacteria|Firmicutes|Clostridia|Clostridiales	0.01	0.02	0.00	0.01	0.00	0.00	0.02	0.04	0.00	0.00	0.00	0.00	0.00	0.00	0.00	0.00
Bacteria|Firmicutes|Clostridia|Clostridiales	3.45	3.42	1.93	1.51	5.08	4.06	3.33	3.47	2.21	6.08	1.56	1.74	2.83	8.32	2.17	6.06
Bacteria|Firmicutes|Clostridia|Thermoanae	0.00	0.00	0.00	0.00	0.00	0.00	0.00	0.00	0.00	0.01	0.00	0.00	0.00	0.00	0.00	0.02
Bacteria|Firmicutes|Erysipelotrichi|Erysipe	0.45	0.62	0.25	0.23	1.01	0.79	0.08	0.09	0.50	1.16	0.25	0.25	0.47	0.82	0.79	1.83

Microbial clades	Oral Cavity								Esophagus							
	All sites		Sa		TD		SP		All sites		UE		ME		LE	
	Ave	SD	Ave	SD	Ave	SD	Ave	SD	Ave	SD	Ave	SD	Ave	SD	Ave	SD
Bacteria|Fusobacteria|Fusobacteriia|Fusoba	2.49	2.42	0.28	0.27	3.32	2.62	3.93	1.77	0.95	0.79	0.96	0.68	1.13	1.03	0.73	0.50
Bacteria|Fusobacteria|Fusobacteriia|Fusoba	1.27	1.53	0.14	0.17	1.33	0.97	2.38	1.94	0.27	0.31	0.29	0.37	0.30	0.32	0.22	0.21
Bacteria|Lentisphaerae|[Lentisphaeria]|Z20	0.00	0.00	0.00	0.00	0.00	0.00	0.00	0.00	0.00	0.00	0.00	0.00	0.00	0.00	0.00	0.00
Bacteria|Planctomycetes|Planctomycetia|G	0.00	0.00	0.00	0.00	0.00	0.00	0.00	0.00	0.00	0.00	0.00	0.00	0.00	0.00	0.00	0.00
Bacteria|Proteobacteria|Alphaproteobacteri	0.09	0.17	0.02	0.05	0.15	0.16	0.11	0.22	2.16	3.59	1.74	1.23	1.75	1.32	3.05	6.07
Bacteria|Proteobacteria|Alphaproteobacteri	0.00	0.00	0.00	0.00	0.00	0.00	0.00	0.00	0.00	0.00	0.00	0.00	0.00	0.00	0.00	0.00
Bacteria|Proteobacteria|Alphaproteobacteri	0.00	0.00	0.00	0.00	0.00	0.00	0.00	0.00	0.00	0.03	0.00	0.00	0.00	0.00	0.01	0.05
Bacteria|Proteobacteria|Alphaproteobacteri	0.00	0.00	0.00	0.00	0.00	0.00	0.00	0.00	0.00	0.01	0.00	0.01	0.00	0.00	0.00	0.01
Bacteria|Proteobacteria|Alphaproteobacteri	0.01	0.02	0.00	0.01	0.01	0.02	0.00	0.01	0.32	0.34	0.23	0.21	0.37	0.34	0.37	0.42
Bacteria|Proteobacteria|Alphaproteobacteri	0.01	0.02	0.01	0.03	0.01	0.01	0.01	0.02	0.47	0.61	0.35	0.47	0.51	0.51	0.54	0.81
Bacteria|Proteobacteria|Alphaproteobacteri	0.00	0.02	0.00	0.01	0.01	0.04	0.00	0.00	0.02	0.04	0.03	0.05	0.01	0.02	0.02	0.04
Bacteria|Proteobacteria|Alphaproteobacteri	0.00	0.01	0.00	0.00	0.00	0.00	0.00	0.01	0.46	0.80	0.62	1.24	0.39	0.50	0.38	0.42
Bacteria|Proteobacteria|Alphaproteobacteri	0.00	0.00	0.00	0.00	0.00	0.00	0.00	0.00	0.00	0.02	0.01	0.04	0.00	0.00	0.00	0.00
Bacteria|Proteobacteria|Alphaproteobacteri	0.00	0.02	0.00	0.01	0.01	0.02	0.00	0.01	0.05	0.12	0.08	0.14	0.06	0.14	0.02	0.03
Bacteria|Proteobacteria|Alphaproteobacteri	0.00	0.01	0.00	0.00	0.01	0.02	0.00	0.00	0.05	0.18	0.01	0.03	0.02	0.04	0.12	0.31
Bacteria|Proteobacteria|Alphaproteobacteri	0.00	0.00	0.00	0.00	0.00	0.00	0.00	0.00	0.00	0.02	0.00	0.02	0.00	0.00	0.01	0.04
Bacteria|Proteobacteria|Alphaproteobacteri	0.00	0.00	0.00	0.00	0.00	0.00	0.00	0.00	0.00	0.00	0.00	0.00	0.00	0.00	0.00	0.00
Bacteria|Proteobacteria|Alphaproteobacteri	0.00	0.01	0.00	0.00	0.00	0.02	0.00	0.00	0.06	0.13	0.10	0.19	0.05	0.10	0.02	0.04
Bacteria|Proteobacteria|Alphaproteobacteri	0.02	0.11	0.02	0.09	0.03	0.16	0.00	0.00	0.09	0.18	0.13	0.23	0.10	0.17	0.06	0.10
Bacteria|Proteobacteria|Alphaproteobacteri	0.00	0.01	0.00	0.01	0.00	0.00	0.00	0.00	0.10	0.21	0.12	0.30	0.10	0.16	0.10	0.15
Bacteria|Proteobacteria|Alphaproteobacteri	0.00	0.00	0.00	0.00	0.00	0.00	0.00	0.00	0.00	0.01	0.00	0.00	0.00	0.00	0.00	0.01
Bacteria|Proteobacteria|Alphaproteobacteri	0.00	0.00	0.00	0.00	0.00	0.00	0.00	0.00	0.00	0.00	0.00	0.00	0.00	0.00	0.00	0.00
Bacteria|Proteobacteria|Alphaproteobacteri	0.00	0.00	0.00	0.00	0.00	0.00	0.00	0.00	0.00	0.00	0.00	0.00	0.00	0.00	0.00	0.00
Bacteria|Proteobacteria|Alphaproteobacteri	0.00	0.00	0.00	0.00	0.00	0.00	0.00	0.00	0.01	0.02	0.02	0.03	0.01	0.02	0.01	0.02
Bacteria|Proteobacteria|Alphaproteobacteri	0.22	0.73	0.17	0.56	0.30	0.98	0.18	0.57	9.99	19.27	11.19	21.44	9.42	18.22	9.45	18.94
Bacteria|Proteobacteria|Betaproteobacteria	0.01	0.02	0.01	0.03	0.01	0.01	0.00	0.01	0.20	0.71	0.07	0.13	0.12	0.44	0.42	1.15
Bacteria|Proteobacteria|Betaproteobacteria	1.27	1.80	1.97	1.75	0.11	0.11	1.75	2.21	0.16	0.15	0.13	0.15	0.16	0.15	0.17	0.16
Bacteria|Proteobacteria|Betaproteobacteria	1.83	2.93	1.66	2.74	0.08	0.13	3.84	3.44	0.67	2.20	0.63	1.00	0.35	0.35	1.07	3.77
Bacteria|Proteobacteria|Betaproteobacteria	0.00	0.01	0.00	0.01	0.01	0.01	0.00	0.01	0.53	0.73	0.75	0.97	0.38	0.50	0.47	0.66

Microbial clades	Oral Cavity								Esophagus							
	All sites		Sa		TD		SP		All sites		UE		ME		LE	
	Ave	SD	Ave	SD	Ave	SD	Ave	SD	Ave	SD	Ave	SD	Ave	SD	Ave	SD
Bacteria|Proteobacteria|Betaproteobacteria	0.00	0.00	0.00	0.00	0.00	0.00	0.00	0.00	0.01	0.06	0.00	0.01	0.03	0.09	0.01	0.03
Bacteria|Proteobacteria|Betaproteobacteria	25.55	14.60	31.97	12.10	27.88	16.59	16.47	9.94	5.14	4.47	4.39	3.02	5.04	3.09	6.01	6.57
Bacteria|Proteobacteria|Betaproteobacteria	0.01	0.03	0.02	0.04	0.00	0.00	0.02	0.04	0.03	0.05	0.02	0.03	0.02	0.03	0.05	0.08
Bacteria|Proteobacteria|Deltaproteobacteria	0.00	0.00	0.00	0.00	0.00	0.00	0.00	0.00	0.00	0.01	0.00	0.01	0.00	0.00	0.00	0.00
Bacteria|Proteobacteria|Deltaproteobacteria	0.00	0.00	0.00	0.00	0.00	0.00	0.00	0.00	0.00	0.02	0.01	0.04	0.00	0.00	0.00	0.00
Bacteria|Proteobacteria|Deltaproteobacteria	0.03	0.08	0.04	0.04	0.00	0.01	0.06	0.12	0.00	0.02	0.00	0.00	0.01	0.01	0.01	0.03
Bacteria|Proteobacteria|Deltaproteobacteria	0.01	0.03	0.01	0.03	0.00	0.00	0.01	0.05	0.00	0.02	0.00	0.00	0.00	0.03	0.00	0.00
Bacteria|Proteobacteria|Deltaproteobacteria	0.06	0.17	0.11	0.24	0.00	0.01	0.06	0.14	0.05	0.12	0.06	0.13	0.02	0.05	0.06	0.16
Bacteria|Proteobacteria|Deltaproteobacteria	0.00	0.00	0.00	0.00	0.00	0.00	0.00	0.00	0.00	0.01	0.00	0.00	0.00	0.00	0.00	0.01
Bacteria|Proteobacteria|Deltaproteobacteria	0.00	0.00	0.00	0.00	0.00	0.00	0.00	0.00	0.00	0.04	0.00	0.00	0.01	0.06	0.00	0.00
Bacteria|Proteobacteria|Deltaproteobacteria	0.00	0.00	0.00	0.00	0.00	0.00	0.00	0.00	0.00	0.00	0.00	0.00	0.00	0.00	0.00	0.00
Bacteria|Proteobacteria|Deltaproteobacteria	0.00	0.00	0.00	0.00	0.00	0.00	0.00	0.00	0.00	0.00	0.00	0.00	0.00	0.00	0.00	0.00
Bacteria|Proteobacteria|Epsilonproteobacte	1.19	1.23	0.13	0.07	1.44	1.31	2.02	1.00	0.53	0.74	0.76	1.08	0.47	0.51	0.37	0.46
Bacteria|Proteobacteria|Epsilonproteobacte	0.01	0.06	0.01	0.02	0.00	0.01	0.03	0.11	1.37	4.74	0.90	3.45	0.34	0.68	2.99	7.48
Bacteria|Proteobacteria|Gammaproteobacte	0.00	0.00	0.00	0.00	0.00	0.00	0.00	0.00	0.01	0.04	0.01	0.05	0.01	0.02	0.01	0.05
Bacteria|Proteobacteria|Gammaproteobacte	0.01	0.03	0.01	0.04	0.00	0.01	0.00	0.01	0.01	0.02	0.00	0.01	0.00	0.01	0.01	0.02
Bacteria|Proteobacteria|Gammaproteobacte	0.00	0.00	0.00	0.00	0.00	0.00	0.00	0.00	0.00	0.01	0.00	0.00	0.00	0.00	0.00	0.01
Bacteria|Proteobacteria|Gammaproteobacte	0.00	0.00	0.00	0.00	0.00	0.00	0.00	0.00	0.01	0.05	0.00	0.00	0.02	0.08	0.01	0.04
Bacteria|Proteobacteria|Gammaproteobacte	0.00	0.00	0.00	0.00	0.00	0.00	0.00	0.00	0.00	0.02	0.00	0.00	0.00	0.00	0.01	0.04
Bacteria|Proteobacteria|Gammaproteobacte	0.59	0.90	0.55	0.50	0.03	0.04	1.22	1.25	0.02	0.06	0.02	0.04	0.01	0.02	0.03	0.09
Bacteria|Proteobacteria|Gammaproteobacte	0.12	0.35	0.11	0.22	0.17	0.55	0.07	0.13	4.58	7.26	2.62	3.14	5.46	9.16	5.53	7.68
Bacteria|Proteobacteria|Gammaproteobacte	0.00	0.00	0.00	0.00	0.00	0.00	0.00	0.00	0.00	0.00	0.00	0.00	0.00	0.00	0.00	0.00
Bacteria|Proteobacteria|Gammaproteobacte	0.00	0.00	0.00	0.00	0.00	0.00	0.00	0.00	0.02	0.04	0.00	0.01	0.02	0.05	0.02	0.05
Bacteria|Proteobacteria|Gammaproteobacte	0.00	0.00	0.00	0.00	0.00	0.00	0.00	0.00	0.02	0.12	0.00	0.01	0.04	0.19	0.02	0.09
Bacteria|Proteobacteria|Gammaproteobacte	0.00	0.00	0.00	0.00	0.00	0.00	0.00	0.00	0.00	0.00	0.00	0.00	0.00	0.00	0.00	0.00
Bacteria|Proteobacteria|Gammaproteobacte	3.31	2.94	2.54	2.07	4.08	3.33	3.32	3.16	13.77	15.88	16.97	21.12	14.08	14.37	10.23	10.44
Bacteria|Proteobacteria|Gammaproteobacte	0.88	3.45	0.10	0.15	1.53	5.67	1.03	1.76	0.75	1.47	0.46	0.53	0.67	0.83	1.11	2.39
Bacteria|Proteobacteria|Gammaproteobacte	0.03	0.06	0.03	0.07	0.02	0.04	0.04	0.07	0.89	1.28	1.03	1.41	0.62	0.69	1.05	1.61
Bacteria|Proteobacteria|Gammaproteobacte	0.00	0.00	0.00	0.00	0.00	0.00	0.00	0.00	0.00	0.00	0.00	0.00	0.00	0.00	0.00	0.00

Microbial clades	Oral Cavity								Esophagus							
	All sites		Sa		TD		SP		All sites		UE		ME		LE	
	Ave	SD	Ave	SD	Ave	SD	Ave	SD	Ave	SD	Ave	SD	Ave	SD	Ave	SD
Bacteria|Proteobacteria|Gammaproteobacte	0.00	0.00	0.00	0.00	0.00	0.00	0.00	0.00	0.00	0.00	0.00	0.00	0.00	0.00	0.00	0.00
Bacteria|Proteobacteria|Gammaproteobacte	0.00	0.00	0.00	0.00	0.00	0.00	0.00	0.00	0.00	0.00	0.00	0.00	0.00	0.00	0.00	0.00
Bacteria|Proteobacteria|Gammaproteobacte	0.00	0.00	0.00	0.00	0.00	0.00	0.00	0.00	0.03	0.06	0.03	0.04	0.02	0.04	0.05	0.09
Bacteria|Proteobacteria|Gammaproteobacte	0.02	0.05	0.01	0.03	0.01	0.01	0.03	0.08	0.91	3.44	2.06	5.99	0.35	0.35	0.40	0.38
Bacteria|Spirochaetes|Spirochaetes|Sphaer	0.00	0.01	0.00	0.01	0.00	0.00	0.01	0.02	0.00	0.00	0.00	0.00	0.00	0.00	0.00	0.00
Bacteria|Spirochaetes|Spirochaetes|Spiroch	0.70	1.10	0.62	0.45	0.13	0.12	1.37	1.66	0.17	0.19	0.17	0.23	0.18	0.19	0.14	0.15
Bacteria|Synergistetes|Synergistia|Synergis	0.12	0.22	0.24	0.31	0.00	0.01	0.10	0.13	0.01	0.01	0.01	0.01	0.01	0.01	0.01	0.02
Bacteria|Synergistetes|Synergistia|Synergis	0.00	0.00	0.00	0.00	0.00	0.00	0.00	0.00	0.00	0.00	0.00	0.00	0.00	0.00	0.00	0.00
Bacteria|Tenericutes|Mollicutes|Acholeplas	0.05	0.20	0.00	0.01	0.01	0.03	0.13	0.34	0.03	0.13	0.01	0.01	0.05	0.14	0.04	0.17
Bacteria|Tenericutes|Mollicutes|Mycoplasr	0.05	0.10	0.03	0.06	0.03	0.05	0.09	0.16	0.09	0.16	0.09	0.21	0.10	0.13	0.06	0.13
Bacteria|Thermotogae|Thermotogae|Therm	0.00	0.00	0.00	0.00	0.00	0.00	0.00	0.00	0.00	0.01	0.00	0.00	0.00	0.00	0.00	0.02
Bacteria|TM7|TM7-3|CW040|F16	0.03	0.05	0.01	0.01	0.05	0.06	0.04	0.06	0.03	0.08	0.02	0.03	0.04	0.12	0.02	0.05
Bacteria|TM7|TM7-3|I025|Rs-045	0.20	0.33	0.35	0.36	0.09	0.26	0.17	0.34	0.18	0.55	0.10	0.32	0.20	0.60	0.23	0.68
Bacteria|Verrucomicrobia|Verruco-5|WCH	0.00	0.00	0.00	0.00	0.00	0.00	0.00	0.00	0.00	0.00	0.00	0.00	0.00	0.00	0.00	0.00
Bacteria|Verrucomicrobia|Verrucomicrobia	0.00	0.00	0.00	0.00	0.00	0.00	0.00	0.00	0.08	0.30	0.07	0.13	0.15	0.47	0.03	0.12
Bacteria|[Thermi]|Deinococci|Deinococcal	0.00	0.00	0.00	0.00	0.00	0.00	0.00	0.00	0.03	0.09	0.02	0.03	0.04	0.15	0.01	0.03
Bacteria|[Thermi]|Deinococci|Deinococcal	0.00	0.00	0.00	0.00	0.00	0.00	0.00	0.00	0.00	0.01	0.01	0.02	0.00	0.00	0.00	0.01
Bacteria|[Thermi]|Deinococci|Thermales|T	0.00	0.00	0.00	0.00	0.00	0.00	0.00	0.00	0.00	0.01	0.00	0.00	0.00	0.00	0.00	0.02
Bacteria|Acidobacteria|[Chloracidobacteria	0.00	0.00	0.00	0.00	0.00	0.00	0.00	0.00	0.00	0.00	0.00	0.00	0.00	0.00	0.00	0.00
Bacteria|Acidobacteria|Acidobacteria-6|iii1	0.00	0.00	0.00	0.00	0.00	0.00	0.00	0.00	0.00	0.00	0.00	0.01	0.00	0.00	0.00	0.00
Bacteria|Acidobacteria|Acidobacteriia|Acic	0.00	0.00	0.00	0.00	0.00	0.00	0.00	0.00	0.00	0.00	0.00	0.00	0.00	0.01	0.00	0.01
Bacteria|Acidobacteria|Holophagae|Holoph	0.00	0.00	0.00	0.00	0.00	0.00	0.00	0.00	0.00	0.00	0.00	0.00	0.00	0.00	0.00	0.00
Bacteria|Actinobacteria|Acidimicrobiia|Aci	0.00	0.00	0.00	0.00	0.00	0.01	0.00	0.00	0.00	0.00	0.00	0.00	0.00	0.00	0.00	0.00
Bacteria|Actinobacteria|Actinobacteria|Act	0.00	0.00	0.00	0.00	0.00	0.00	0.00	0.00	0.00	0.00	0.00	0.00	0.00	0.00	0.00	0.01
Bacteria|Actinobacteria|Actinobacteria|Act	3.51	3.68	5.06	4.47	3.47	3.09	1.96	2.65	0.43	0.46	0.44	0.49	0.44	0.45	0.42	0.45
Bacteria|Actinobacteria|Actinobacteria|Act	0.00	0.01	0.00	0.01	0.00	0.00	0.00	0.00	0.00	0.01	0.00	0.00	0.00	0.00	0.00	0.02
Bacteria|Actinobacteria|Actinobacteria|Act	0.00	0.00	0.00	0.00	0.00	0.00	0.00	0.00	0.00	0.00	0.00	0.00	0.00	0.00	0.00	0.00
Bacteria|Actinobacteria|Actinobacteria|Act	0.00	0.00	0.00	0.00	0.00	0.00	0.00	0.00	0.00	0.00	0.00	0.00	0.00	0.00	0.00	0.01
Bacteria|Actinobacteria|Actinobacteria|Act	0.07	0.20	0.06	0.10	0.01	0.02	0.13	0.33	0.01	0.04	0.00	0.01	0.01	0.02	0.02	0.06

Microbial clades	Oral Cavity								Esophagus							
	All sites		Sa		TD		SP		All sites		UE		ME		LE	
	Ave	SD	Ave	SD	Ave	SD	Ave	SD	Ave	SD	Ave	SD	Ave	SD	Ave	SD
Bacteria|Actinobacteria|Actinobacteria|Act	0.00	0.00	0.00	0.00	0.00	0.00	0.00	0.00	0.00	0.00	0.00	0.01	0.00	0.00	0.00	0.00
Bacteria|Actinobacteria|Actinobacteria|Act	0.00	0.00	0.00	0.00	0.00	0.00	0.00	0.00	0.00	0.02	0.00	0.01	0.01	0.03	0.00	0.00
Bacteria|Actinobacteria|Actinobacteria|Act	0.65	0.91	0.72	0.67	0.02	0.03	1.25	1.15	0.11	0.17	0.09	0.11	0.11	0.21	0.12	0.16
Bacteria|Actinobacteria|Actinobacteria|Act	0.00	0.00	0.00	0.00	0.00	0.00	0.00	0.00	0.00	0.00	0.00	0.00	0.00	0.00	0.00	0.00
Bacteria|Actinobacteria|Actinobacteria|Act	0.00	0.00	0.00	0.00	0.00	0.00	0.00	0.00	0.00	0.00	0.00	0.00	0.00	0.00	0.00	0.00
Bacteria|Actinobacteria|Actinobacteria|Act	0.00	0.00	0.00	0.00	0.00	0.00	0.00	0.00	0.00	0.00	0.00	0.00	0.00	0.00	0.00	0.00
Bacteria|Actinobacteria|Actinobacteria|Act	0.00	0.00	0.00	0.00	0.00	0.00	0.00	0.00	0.00	0.00	0.00	0.00	0.00	0.00	0.00	0.00
Bacteria|Actinobacteria|Actinobacteria|Act	0.00	0.00	0.00	0.00	0.00	0.00	0.00	0.00	0.00	0.01	0.00	0.01	0.00	0.00	0.00	0.00
Bacteria|Actinobacteria|Actinobacteria|Act	0.00	0.00	0.00	0.00	0.00	0.00	0.00	0.00	0.00	0.00	0.00	0.00	0.00	0.00	0.00	0.00
Bacteria|Actinobacteria|Actinobacteria|Act	0.00	0.00	0.00	0.00	0.00	0.00	0.00	0.00	0.00	0.01	0.00	0.01	0.00	0.00	0.00	0.00
Bacteria|Actinobacteria|Actinobacteria|Act	0.00	0.00	0.00	0.00	0.00	0.00	0.00	0.00	0.01	0.03	0.01	0.03	0.01	0.04	0.00	0.00
Bacteria|Actinobacteria|Actinobacteria|Act	0.00	0.00	0.00	0.00	0.00	0.00	0.00	0.00	0.00	0.03	0.01	0.06	0.00	0.00	0.00	0.00
Bacteria|Actinobacteria|Actinobacteria|Act	0.00	0.00	0.00	0.00	0.00	0.00	0.00	0.00	0.00	0.01	0.00	0.01	0.00	0.00	0.00	0.00
Bacteria|Actinobacteria|Actinobacteria|Act	0.00	0.00	0.00	0.00	0.00	0.00	0.00	0.00	0.00	0.00	0.00	0.00	0.00	0.00	0.00	0.00
Bacteria|Actinobacteria|Actinobacteria|Act	0.00	0.00	0.00	0.00	0.00	0.00	0.00	0.00	0.00	0.01	0.00	0.01	0.00	0.00	0.00	0.00
Bacteria|Actinobacteria|Actinobacteria|Act	0.00	0.00	0.00	0.00	0.00	0.00	0.00	0.00	0.00	0.00	0.00	0.00	0.00	0.00	0.00	0.00
Bacteria|Actinobacteria|Actinobacteria|Act	0.00	0.00	0.00	0.00	0.00	0.00	0.00	0.00	0.06	0.15	0.02	0.03	0.09	0.18	0.06	0.19
Bacteria|Actinobacteria|Actinobacteria|Act	0.00	0.00	0.00	0.00	0.00	0.00	0.00	0.00	0.00	0.03	0.01	0.04	0.00	0.02	0.00	0.00
Bacteria|Actinobacteria|Actinobacteria|Act	0.00	0.00	0.00	0.00	0.00	0.00	0.00	0.00	0.00	0.00	0.00	0.00	0.00	0.00	0.00	0.00
Bacteria|Actinobacteria|Actinobacteria|Act	0.00	0.00	0.00	0.00	0.00	0.00	0.00	0.00	0.00	0.00	0.00	0.00	0.00	0.00	0.00	0.00
Bacteria|Actinobacteria|Actinobacteria|Act	0.00	0.00	0.00	0.00	0.00	0.00	0.00	0.00	0.00	0.01	0.00	0.01	0.00	0.01	0.00	0.00
Bacteria|Actinobacteria|Actinobacteria|Act	0.00	0.00	0.00	0.00	0.00	0.00	0.00	0.00	0.00	0.00	0.00	0.00	0.00	0.00	0.00	0.00
Bacteria|Actinobacteria|Actinobacteria|Act	0.00	0.00	0.00	0.00	0.00	0.00	0.00	0.00	0.00	0.00	0.00	0.00	0.00	0.00	0.00	0.00
Bacteria|Actinobacteria|Actinobacteria|Act	0.00	0.00	0.00	0.00	0.00	0.00	0.00	0.00	0.00	0.01	0.00	0.00	0.00	0.02	0.00	0.00
Bacteria|Actinobacteria|Actinobacteria|Act	0.00	0.00	0.00	0.00	0.00	0.00	0.00	0.00	0.01	0.03	0.02	0.02	0.01	0.02	0.02	0.04
Bacteria|Actinobacteria|Actinobacteria|Act	0.00	0.00	0.00	0.00	0.00	0.00	0.00	0.00	0.00	0.00	0.00	0.00	0.00	0.00	0.00	0.00
Bacteria|Actinobacteria|Actinobacteria|Act	0.00	0.00	0.00	0.00	0.00	0.00	0.00	0.00	0.00	0.00	0.00	0.00	0.00	0.00	0.00	0.00
Bacteria|Actinobacteria|Actinobacteria|Act	0.00	0.00	0.00	0.00	0.00	0.00	0.00	0.00	0.00	0.00	0.00	0.00	0.00	0.00	0.00	0.00
Bacteria|Actinobacteria|Actinobacteria|Act	0.00	0.00	0.00	0.00	0.00	0.00	0.00	0.00	0.01	0.02	0.01	0.03	0.01	0.01	0.01	0.01

Microbial clades	Oral Cavity								Esophagus							
	All sites		Sa		TD		SP		All sites		UE		ME		LE	
	Ave	SD	Ave	SD	Ave	SD	Ave	SD	Ave	SD	Ave	SD	Ave	SD	Ave	SD
Bacteria|Actinobacteria|Actinobacteria|Act	0.00	0.00	0.00	0.00	0.00	0.00	0.00	0.00	0.00	0.01	0.00	0.00	0.00	0.00	0.00	0.02
Bacteria|Actinobacteria|Actinobacteria|Act	0.00	0.00	0.00	0.00	0.00	0.00	0.00	0.00	0.01	0.02	0.00	0.00	0.00	0.00	0.01	0.04
Bacteria|Actinobacteria|Actinobacteria|Act	0.00	0.00	0.00	0.00	0.00	0.00	0.00	0.00	0.00	0.00	0.00	0.00	0.00	0.00	0.00	0.00
Bacteria|Actinobacteria|Actinobacteria|Act	0.00	0.00	0.00	0.00	0.00	0.00	0.00	0.00	0.01	0.04	0.01	0.05	0.01	0.03	0.01	0.04
Bacteria|Actinobacteria|Actinobacteria|Act	0.00	0.00	0.00	0.00	0.00	0.00	0.00	0.00	0.00	0.01	0.00	0.00	0.00	0.02	0.00	0.00
Bacteria|Actinobacteria|Actinobacteria|Act	0.00	0.01	0.00	0.00	0.00	0.00	0.00	0.01	0.02	0.07	0.04	0.12	0.01	0.04	0.00	0.00
Bacteria|Actinobacteria|Actinobacteria|Act	3.86	6.18	9.77	7.50	1.49	2.05	0.20	0.26	0.51	0.66	0.64	0.92	0.48	0.46	0.41	0.54
Bacteria|Actinobacteria|Actinobacteria|Act	0.00	0.00	0.00	0.00	0.00	0.00	0.00	0.00	0.00	0.00	0.00	0.00	0.00	0.00	0.00	0.00
Bacteria|Actinobacteria|Actinobacteria|Act	0.00	0.00	0.00	0.00	0.00	0.00	0.00	0.00	0.01	0.02	0.01	0.03	0.01	0.02	0.00	0.02
Bacteria|Actinobacteria|Actinobacteria|Act	0.00	0.00	0.00	0.00	0.00	0.00	0.00	0.00	0.01	0.03	0.00	0.01	0.00	0.00	0.02	0.04
Bacteria|Actinobacteria|Actinobacteria|Act	0.00	0.00	0.00	0.00	0.00	0.00	0.00	0.00	0.00	0.01	0.00	0.00	0.00	0.02	0.00	0.00
Bacteria|Actinobacteria|Actinobacteria|Act	0.00	0.00	0.00	0.00	0.00	0.00	0.00	0.00	0.00	0.00	0.00	0.00	0.00	0.00	0.00	0.00
Bacteria|Actinobacteria|Actinobacteria|Act	0.00	0.00	0.00	0.00	0.00	0.00	0.00	0.00	0.02	0.03	0.02	0.03	0.02	0.04	0.02	0.03
Bacteria|Actinobacteria|Actinobacteria|Act	0.00	0.00	0.00	0.00	0.00	0.00	0.00	0.00	0.01	0.04	0.00	0.00	0.00	0.00	0.02	0.08
Bacteria|Actinobacteria|Actinobacteria|Act	0.00	0.00	0.00	0.00	0.00	0.00	0.00	0.00	0.00	0.01	0.00	0.00	0.00	0.00	0.00	0.01
Bacteria|Actinobacteria|Actinobacteria|Act	0.00	0.00	0.00	0.00	0.00	0.00	0.00	0.00	0.00	0.00	0.00	0.00	0.00	0.00	0.00	0.00
Bacteria|Actinobacteria|Actinobacteria|Act	0.00	0.00	0.00	0.00	0.00	0.00	0.00	0.00	0.00	0.02	0.00	0.02	0.00	0.00	0.01	0.03
Bacteria|Actinobacteria|Actinobacteria|Act	0.00	0.00	0.00	0.00	0.00	0.00	0.00	0.00	0.00	0.00	0.00	0.01	0.00	0.00	0.00	0.00
Bacteria|Actinobacteria|Actinobacteria|Act	0.00	0.01	0.00	0.00	0.00	0.00	0.01	0.02	0.01	0.06	0.02	0.10	0.00	0.00	0.00	0.00
Bacteria|Actinobacteria|Actinobacteria|Act	0.00	0.00	0.00	0.00	0.00	0.00	0.00	0.00	0.00	0.00	0.00	0.00	0.00	0.00	0.00	0.00
Bacteria|Actinobacteria|Actinobacteria|Act	0.00	0.00	0.00	0.00	0.00	0.01	0.00	0.00	0.01	0.07	0.03	0.12	0.01	0.02	0.00	0.01
Bacteria|Actinobacteria|Actinobacteria|Act	0.00	0.00	0.00	0.00	0.00	0.00	0.00	0.00	0.00	0.01	0.00	0.00	0.00	0.01	0.00	0.02
Bacteria|Actinobacteria|Actinobacteria|Act	0.00	0.00	0.00	0.00	0.00	0.00	0.00	0.00	0.00	0.00	0.00	0.00	0.00	0.00	0.00	0.00
Bacteria|Actinobacteria|Actinobacteria|Act	0.00	0.00	0.00	0.00	0.00	0.00	0.00	0.00	0.00	0.00	0.00	0.00	0.00	0.00	0.00	0.00
Bacteria|Actinobacteria|Actinobacteria|Act	0.00	0.00	0.00	0.00	0.00	0.00	0.00	0.00	0.00	0.00	0.00	0.00	0.00	0.00	0.00	0.01
Bacteria|Actinobacteria|Actinobacteria|Act	0.00	0.00	0.00	0.00	0.00	0.00	0.00	0.00	0.00	0.01	0.01	0.02	0.00	0.00	0.00	0.00
Bacteria|Actinobacteria|Actinobacteria|Act	0.00	0.00	0.00	0.00	0.00	0.00	0.00	0.00	0.25	0.44	0.17	0.24	0.29	0.47	0.28	0.57
Bacteria|Actinobacteria|Actinobacteria|Act	0.00	0.00	0.00	0.00	0.00	0.00	0.00	0.00	0.00	0.00	0.00	0.00	0.00	0.00	0.00	0.00
Bacteria|Actinobacteria|Actinobacteria|Act	0.53	1.39	0.35	0.37	0.01	0.01	1.28	2.26	0.03	0.07	0.03	0.04	0.04	0.11	0.03	0.05

Microbial clades	Oral Cavity								Esophagus							
	All sites		Sa		TD		SP		All sites		UE		ME		LE	
	Ave	SD	Ave	SD	Ave	SD	Ave	SD	Ave	SD	Ave	SD	Ave	SD	Ave	SD
Bacteria|Actinobacteria|Actinobacteria|Act	0.00	0.00	0.00	0.00	0.00	0.00	0.00	0.00	0.01	0.03	0.00	0.00	0.01	0.04	0.01	0.03
Bacteria|Actinobacteria|Actinobacteria|Act	0.00	0.00	0.00	0.00	0.00	0.00	0.00	0.00	0.00	0.01	0.00	0.00	0.00	0.02	0.00	0.00
Bacteria|Actinobacteria|Actinobacteria|Act	0.00	0.00	0.00	0.00	0.00	0.00	0.00	0.00	0.00	0.01	0.00	0.00	0.00	0.02	0.00	0.00
Bacteria|Actinobacteria|Actinobacteria|Act	0.00	0.00	0.00	0.00	0.00	0.00	0.00	0.00	0.00	0.00	0.00	0.00	0.00	0.00	0.00	0.00
Bacteria|Actinobacteria|Actinobacteria|Act	0.00	0.00	0.00	0.00	0.00	0.00	0.00	0.00	0.00	0.02	0.01	0.04	0.00	0.00	0.00	0.00
Bacteria|Actinobacteria|Actinobacteria|Act	0.00	0.00	0.00	0.00	0.00	0.00	0.00	0.00	0.00	0.00	0.00	0.00	0.00	0.00	0.00	0.00
Bacteria|Actinobacteria|Actinobacteria|Act	0.00	0.00	0.00	0.00	0.00	0.00	0.00	0.00	0.11	0.27	0.04	0.10	0.14	0.29	0.15	0.36
Bacteria|Actinobacteria|Actinobacteria|Act	0.00	0.00	0.00	0.00	0.00	0.00	0.00	0.00	0.00	0.00	0.00	0.00	0.00	0.00	0.00	0.00
Bacteria|Actinobacteria|Actinobacteria|Act	0.00	0.00	0.00	0.00	0.00	0.00	0.00	0.00	0.00	0.01	0.00	0.00	0.00	0.00	0.00	0.01
Bacteria|Actinobacteria|Actinobacteria|Act	0.00	0.00	0.00	0.00	0.00	0.00	0.00	0.00	0.00	0.00	0.00	0.00	0.00	0.00	0.00	0.00
Bacteria|Actinobacteria|Actinobacteria|Bifi	0.11	0.59	0.11	0.35	0.03	0.08	0.20	0.98	0.09	0.35	0.15	0.61	0.05	0.10	0.07	0.12
Bacteria|Actinobacteria|Actinobacteria|Bifi	0.00	0.00	0.00	0.00	0.00	0.00	0.00	0.00	0.00	0.00	0.00	0.00	0.00	0.00	0.00	0.00
Bacteria|Actinobacteria|Actinobacteria|Bifi	0.01	0.02	0.01	0.02	0.01	0.01	0.01	0.02	0.00	0.01	0.00	0.01	0.00	0.01	0.00	0.00
Bacteria|Actinobacteria|Actinobacteria|Bifi	0.00	0.01	0.00	0.00	0.00	0.01	0.00	0.01	0.01	0.01	0.00	0.01	0.00	0.01	0.01	0.02
Bacteria|Actinobacteria|Actinobacteria|Bifi	0.15	0.35	0.13	0.27	0.18	0.37	0.14	0.40	0.64	1.55	0.41	0.98	0.44	1.00	1.10	2.32
Bacteria|Actinobacteria|Coriobacteriia|Cori	0.00	0.00	0.00	0.00	0.00	0.00	0.00	0.00	0.00	0.02	0.00	0.01	0.00	0.00	0.01	0.03
Bacteria|Actinobacteria|Coriobacteriia|Cori	0.28	0.48	0.55	0.66	0.28	0.35	0.01	0.02	0.02	0.05	0.03	0.07	0.02	0.04	0.01	0.02
Bacteria|Actinobacteria|Coriobacteriia|Cori	0.00	0.00	0.00	0.00	0.00	0.00	0.00	0.00	0.00	0.00	0.00	0.00	0.00	0.00	0.00	0.00
Bacteria|Actinobacteria|Coriobacteriia|Cori	0.00	0.00	0.00	0.00	0.00	0.00	0.00	0.00	0.00	0.00	0.00	0.00	0.00	0.00	0.00	0.00
Bacteria|Actinobacteria|Coriobacteriia|Cori	0.01	0.01	0.01	0.02	0.00	0.01	0.00	0.01	0.00	0.00	0.00	0.00	0.00	0.00	0.00	0.00
Bacteria|Actinobacteria|Coriobacteriia|Cori	0.00	0.02	0.01	0.02	0.00	0.00	0.00	0.01	0.00	0.00	0.00	0.00	0.00	0.00	0.00	0.00
Bacteria|Actinobacteria|Coriobacteriia|Cori	0.04	0.08	0.06	0.10	0.02	0.06	0.03	0.09	0.07	0.17	0.06	0.13	0.06	0.13	0.11	0.24
Bacteria|Actinobacteria|Nitriliruptoria|Nitri	0.00	0.00	0.00	0.00	0.00	0.00	0.00	0.00	0.00	0.00	0.00	0.00	0.00	0.00	0.00	0.00
Bacteria|Actinobacteria|Rubrobacteria|Rub	0.00	0.00	0.00	0.00	0.00	0.00	0.00	0.00	0.00	0.00	0.00	0.00	0.00	0.00	0.00	0.00
Bacteria|Actinobacteria|Thermoleophilia|So	0.00	0.00	0.00	0.00	0.00	0.00	0.00	0.00	0.00	0.01	0.00	0.00	0.00	0.00	0.00	0.01
Bacteria|Actinobacteria|Thermoleophilia|So	0.00	0.00	0.00	0.00	0.00	0.00	0.00	0.00	0.00	0.00	0.00	0.00	0.00	0.00	0.00	0.00
Bacteria|Armatimonadetes|[Fimbriimonadi	0.00	0.00	0.00	0.00	0.00	0.00	0.00	0.00	0.02	0.04	0.02	0.04	0.01	0.03	0.03	0.04
Bacteria|Armatimonadetes|Armatimonadia	0.00	0.00	0.00	0.00	0.00	0.00	0.00	0.00	0.00	0.01	0.00	0.02	0.00	0.00	0.00	0.00
Bacteria|Bacteroidetes|[Saprospirae]|[Sapro	0.00	0.00	0.00	0.00	0.00	0.00	0.00	0.00	0.00	0.00	0.00	0.00	0.00	0.00	0.00	0.00

Microbial clades	Oral Cavity								Esophagus							
	All sites		Sa		TD		SP		All sites		UE		ME		LE	
	Ave	SD	Ave	SD	Ave	SD	Ave	SD	Ave	SD	Ave	SD	Ave	SD	Ave	SD
Bacteria|Bacteroidetes|[Saprospirae]|[Sapro	0.00	0.00	0.00	0.00	0.00	0.00	0.00	0.00	0.00	0.01	0.00	0.02	0.00	0.00	0.00	0.00
Bacteria|Bacteroidetes|[Saprospirae]|[Sapro	0.00	0.00	0.00	0.00	0.00	0.00	0.00	0.00	0.00	0.00	0.00	0.00	0.00	0.00	0.00	0.00
Bacteria|Bacteroidetes|[Saprospirae]|[Sapro	0.00	0.02	0.01	0.04	0.00	0.01	0.00	0.01	0.30	0.91	0.15	0.10	0.25	0.31	0.51	1.58
Bacteria|Bacteroidetes|[Saprospirae]|[Sapro	0.00	0.01	0.00	0.00	0.00	0.01	0.00	0.00	0.03	0.09	0.07	0.15	0.00	0.01	0.01	0.02
Bacteria|Bacteroidetes|[Saprospirae]|[Sapro	0.00	0.00	0.00	0.00	0.00	0.00	0.00	0.00	0.00	0.00	0.00	0.00	0.00	0.00	0.00	0.00
Bacteria|Bacteroidetes|[Saprospirae]|[Sapro	0.00	0.00	0.00	0.00	0.00	0.00	0.00	0.00	0.00	0.02	0.00	0.00	0.00	0.00	0.01	0.03
Bacteria|Bacteroidetes|Bacteroidia|Bactero	0.00	0.00	0.00	0.00	0.00	0.00	0.00	0.00	0.00	0.00	0.00	0.00	0.00	0.00	0.00	0.00
Bacteria|Bacteroidetes|Bacteroidia|Bactero	0.00	0.00	0.00	0.01	0.00	0.00	0.00	0.00	0.01	0.06	0.00	0.01	0.02	0.07	0.01	0.07
Bacteria|Bacteroidetes|Bacteroidia|Bactero	0.01	0.04	0.00	0.01	0.00	0.00	0.01	0.07	0.00	0.01	0.00	0.00	0.00	0.01	0.01	0.02
Bacteria|Bacteroidetes|Bacteroidia|Bactero	7.19	5.67	12.65	4.64	6.99	3.63	1.73	1.53	4.35	5.29	3.99	3.62	4.73	6.82	4.28	4.90
Bacteria|Bacteroidetes|Bacteroidia|Bactero	0.00	0.00	0.00	0.00	0.00	0.00	0.00	0.00	0.00	0.00	0.00	0.00	0.00	0.00	0.00	0.00
Bacteria|Bacteroidetes|Bacteroidia|Bactero	0.00	0.00	0.00	0.00	0.00	0.00	0.00	0.00	0.00	0.00	0.00	0.00	0.00	0.00	0.00	0.00
Bacteria|Bacteroidetes|Bacteroidia|Bactero	0.01	0.03	0.00	0.00	0.00	0.00	0.01	0.04	0.00	0.00	0.00	0.00	0.00	0.00	0.00	0.00
Bacteria|Bacteroidetes|Bacteroidia|Bactero	0.07	0.20	0.12	0.31	0.01	0.02	0.08	0.16	0.41	0.81	0.27	0.47	0.40	0.70	0.56	1.14
Bacteria|Bacteroidetes|Bacteroidia|Bactero	0.02	0.09	0.05	0.11	0.01	0.01	0.02	0.09	0.02	0.07	0.02	0.09	0.02	0.09	0.01	0.02
Bacteria|Bacteroidetes|Bacteroidia|Bactero	0.00	0.01	0.00	0.00	0.00	0.01	0.00	0.00	0.00	0.00	0.00	0.00	0.00	0.01	0.00	0.00
Bacteria|Bacteroidetes|Bacteroidia|Bactero	0.00	0.00	0.00	0.00	0.00	0.00	0.00	0.00	0.00	0.00	0.00	0.00	0.00	0.00	0.00	0.01
Bacteria|Bacteroidetes|Bacteroidia|Bactero	0.33	0.63	0.04	0.08	0.01	0.02	0.95	0.79	0.02	0.06	0.04	0.10	0.02	0.04	0.01	0.03
Bacteria|Bacteroidetes|Bacteroidia|Bactero	0.00	0.01	0.00	0.02	0.00	0.00	0.00	0.01	0.04	0.12	0.01	0.01	0.02	0.05	0.08	0.20
Bacteria|Bacteroidetes|Bacteroidia|Bactero	6.78	6.79	12.91	7.75	3.94	4.20	3.36	1.88	1.89	1.79	1.79	1.73	2.15	2.08	1.70	1.54
Bacteria|Bacteroidetes|Bacteroidia|Bactero	0.44	0.50	0.52	0.45	0.10	0.14	0.71	0.59	0.07	0.11	0.05	0.08	0.06	0.09	0.09	0.16
Bacteria|Bacteroidetes|Bacteroidia|Bactero	0.00	0.00	0.00	0.00	0.00	0.00	0.00	0.00	0.00	0.01	0.00	0.01	0.00	0.00	0.00	0.01
Bacteria|Bacteroidetes|Bacteroidia|Bactero	10.16	9.75	4.16	2.72	14.99	10.84	11.37	10.25	3.99	4.72	3.93	4.91	4.50	5.11	3.47	4.20
Bacteria|Bacteroidetes|Bacteroidia|Bactero	0.00	0.01	0.00	0.00	0.00	0.00	0.00	0.02	0.00	0.01	0.00	0.00	0.00	0.01	0.00	0.00
Bacteria|Bacteroidetes|Bacteroidia|Bactero	0.00	0.00	0.00	0.00	0.00	0.00	0.00	0.00	0.00	0.00	0.00	0.00	0.00	0.00	0.00	0.00
Bacteria|Bacteroidetes|Bacteroidia|Bactero	0.00	0.00	0.00	0.00	0.00	0.00	0.00	0.00	0.00	0.00	0.00	0.00	0.00	0.00	0.00	0.00
Bacteria|Bacteroidetes|Bacteroidia|Bactero	0.00	0.00	0.00	0.00	0.00	0.00	0.00	0.00	0.00	0.02	0.01	0.03	0.00	0.00	0.00	0.00
Bacteria|Bacteroidetes|Bacteroidia|Bactero	0.00	0.02	0.01	0.03	0.00	0.01	0.00	0.01	0.03	0.08	0.02	0.06	0.03	0.10	0.04	0.07
Bacteria|Bacteroidetes|Bacteroidia|Bactero	0.01	0.01	0.01	0.01	0.01	0.01	0.01	0.01	0.74	1.95	0.50	0.71	1.03	2.75	0.65	1.72

Microbial clades	Oral Cavity								Esophagus							
	All sites		Sa		TD		SP		All sites		UE		ME		LE	
	Ave	SD	Ave	SD	Ave	SD	Ave	SD	Ave	SD	Ave	SD	Ave	SD	Ave	SD
Bacteria|Bacteroidetes|Cytophagia|Cytopha	0.00	0.00	0.00	0.00	0.00	0.00	0.00	0.00	0.00	0.00	0.00	0.00	0.00	0.00	0.00	0.00
Bacteria|Bacteroidetes|Cytophagia|Cytopha	0.00	0.00	0.00	0.00	0.00	0.00	0.00	0.00	0.00	0.00	0.00	0.00	0.00	0.00	0.00	0.00
Bacteria|Bacteroidetes|Cytophagia|Cytopha	0.00	0.00	0.00	0.00	0.00	0.00	0.00	0.00	0.00	0.00	0.00	0.00	0.00	0.00	0.00	0.00
Bacteria|Bacteroidetes|Cytophagia|Cytopha	0.00	0.00	0.00	0.00	0.00	0.00	0.00	0.00	0.00	0.02	0.01	0.03	0.00	0.00	0.00	0.00
Bacteria|Bacteroidetes|Cytophagia|Cytopha	0.00	0.00	0.00	0.00	0.00	0.00	0.00	0.00	0.00	0.00	0.00	0.00	0.00	0.00	0.00	0.00
Bacteria|Bacteroidetes|Cytophagia|Cytopha	0.00	0.00	0.00	0.00	0.00	0.00	0.00	0.01	0.01	0.02	0.00	0.01	0.01	0.04	0.00	0.01
Bacteria|Bacteroidetes|Cytophagia|Cytopha	0.00	0.00	0.00	0.00	0.00	0.00	0.00	0.00	0.00	0.02	0.01	0.04	0.00	0.00	0.00	0.00
Bacteria|Bacteroidetes|Cytophagia|Cytopha	0.00	0.00	0.00	0.00	0.00	0.00	0.00	0.00	0.00	0.01	0.00	0.02	0.00	0.00	0.00	0.00
Bacteria|Bacteroidetes|Cytophagia|Cytopha	0.00	0.00	0.00	0.00	0.00	0.00	0.00	0.00	0.00	0.00	0.00	0.00	0.00	0.00	0.00	0.00
Bacteria|Bacteroidetes|Cytophagia|Cytopha	0.00	0.00	0.00	0.00	0.00	0.00	0.00	0.00	0.00	0.00	0.00	0.00	0.00	0.00	0.00	0.00
Bacteria|Bacteroidetes|Cytophagia|Cytopha	0.00	0.00	0.00	0.00	0.00	0.00	0.00	0.00	0.00	0.00	0.00	0.00	0.00	0.00	0.00	0.00
Bacteria|Bacteroidetes|Flavobacteriia|Flavo	0.00	0.01	0.00	0.01	0.00	0.01	0.00	0.00	0.19	0.57	0.19	0.42	0.08	0.22	0.31	0.89
Bacteria|Bacteroidetes|Flavobacteriia|Flavo	0.00	0.00	0.00	0.00	0.00	0.00	0.00	0.00	0.01	0.07	0.00	0.01	0.02	0.07	0.03	0.10
Bacteria|Bacteroidetes|Flavobacteriia|Flavo	0.00	0.00	0.00	0.00	0.00	0.00	0.00	0.00	0.03	0.06	0.02	0.04	0.03	0.09	0.03	0.05
Bacteria|Bacteroidetes|Flavobacteriia|Flavo	0.00	0.00	0.00	0.00	0.00	0.00	0.00	0.00	0.00	0.00	0.00	0.00	0.00	0.00	0.00	0.01
Bacteria|Bacteroidetes|Flavobacteriia|Flavo	0.00	0.00	0.00	0.00	0.00	0.00	0.00	0.00	0.00	0.00	0.00	0.00	0.00	0.00	0.00	0.00
Bacteria|Bacteroidetes|Flavobacteriia|Flavo	0.86	2.19	0.07	0.16	0.11	0.11	2.46	3.34	0.33	0.40	0.34	0.41	0.33	0.32	0.31	0.47
Bacteria|Bacteroidetes|Flavobacteriia|Flavo	0.00	0.00	0.00	0.00	0.00	0.00	0.00	0.00	0.00	0.00	0.00	0.00	0.00	0.00	0.00	0.00
Bacteria|Bacteroidetes|Flavobacteriia|Flavo	5.92	10.35	0.42	0.34	0.63	0.63	17.12	12.02	0.39	0.43	0.38	0.36	0.48	0.55	0.29	0.31
Bacteria|Bacteroidetes|Flavobacteriia|Flavo	0.00	0.01	0.00	0.00	0.00	0.01	0.00	0.00	0.02	0.06	0.02	0.06	0.02	0.04	0.04	0.09
Bacteria|Bacteroidetes|Flavobacteriia|Flavo	0.00	0.00	0.00	0.00	0.00	0.00	0.00	0.00	0.00	0.00	0.00	0.00	0.00	0.00	0.00	0.00
Bacteria|Bacteroidetes|Flavobacteriia|Flavo	0.00	0.01	0.00	0.00	0.00	0.00	0.00	0.01	0.00	0.00	0.00	0.00	0.00	0.00	0.00	0.00
Bacteria|Bacteroidetes|Sphingobacteriia|Sp	0.01	0.04	0.00	0.01	0.02	0.06	0.01	0.03	0.22	1.20	0.69	2.08	0.00	0.00	0.01	0.01
Bacteria|Bacteroidetes|Sphingobacteriia|Sp	0.00	0.00	0.00	0.00	0.00	0.00	0.00	0.00	0.01	0.01	0.01	0.02	0.00	0.01	0.00	0.01
Bacteria|Bacteroidetes|Sphingobacteriia|Sp	0.00	0.00	0.00	0.00	0.00	0.01	0.00	0.00	0.00	0.01	0.00	0.00	0.00	0.00	0.00	0.01
Bacteria|Chlamydiae|Chlamydiia|Chlamyd	0.00	0.00	0.00	0.00	0.00	0.00	0.00	0.00	0.00	0.00	0.00	0.00	0.00	0.01	0.00	0.00
Bacteria|Chlamydiae|Chlamydiia|Chlamyd	0.00	0.00	0.00	0.00	0.00	0.00	0.00	0.00	0.00	0.00	0.00	0.00	0.00	0.00	0.00	0.00
Bacteria|Chloroflexi|Anaerolineae|Anaerol	0.00	0.00	0.00	0.00	0.00	0.00	0.00	0.00	0.00	0.00	0.00	0.00	0.00	0.00	0.00	0.00
Bacteria|Chloroflexi|Anaerolineae|Caldilin	0.00	0.00	0.00	0.00	0.00	0.00	0.00	0.00	0.00	0.00	0.00	0.00	0.00	0.00	0.00	0.00

Microbial clades	Oral Cavity								Esophagus							
	All sites		Sa		TD		SP		All sites		UE		ME		LE	
	Ave	SD	Ave	SD	Ave	SD	Ave	SD	Ave	SD	Ave	SD	Ave	SD	Ave	SD
Bacteria|Chloroflexi|Anaerolineae|Caldilin	0.00	0.00	0.00	0.00	0.00	0.00	0.00	0.00	0.00	0.00	0.00	0.00	0.00	0.00	0.00	0.00
Bacteria|Chloroflexi|Anaerolineae|SBR103	0.00	0.00	0.00	0.00	0.00	0.00	0.00	0.00	0.00	0.01	0.00	0.00	0.00	0.00	0.00	0.02
Bacteria|Cyanobacteria|Oscillatoriophycide	0.00	0.00	0.00	0.00	0.00	0.00	0.00	0.00	0.00	0.01	0.00	0.00	0.00	0.01	0.00	0.00
Bacteria|Cyanobacteria|Oscillatoriophycide	0.00	0.00	0.00	0.00	0.00	0.00	0.00	0.00	0.00	0.02	0.00	0.00	0.01	0.03	0.00	0.01
Bacteria|Fibrobacteres|Fibrobacteria|Fibrob	0.00	0.00	0.00	0.00	0.00	0.00	0.00	0.00	0.00	0.00	0.00	0.00	0.00	0.00	0.00	0.00
Bacteria|Firmicutes|Bacilli|Bacillales|[Exig	0.00	0.00	0.00	0.00	0.00	0.00	0.00	0.00	0.00	0.01	0.00	0.02	0.00	0.01	0.00	0.00
Bacteria|Firmicutes|Bacilli|Bacillales|[Ther	0.00	0.00	0.00	0.00	0.00	0.00	0.00	0.00	0.00	0.00	0.00	0.00	0.00	0.00	0.00	0.00
Bacteria|Firmicutes|Bacilli|Bacillales|Alicy	0.00	0.00	0.00	0.00	0.00	0.00	0.00	0.00	0.00	0.00	0.00	0.00	0.00	0.00	0.00	0.00
Bacteria|Firmicutes|Bacilli|Bacillales|Bacil	0.00	0.00	0.00	0.00	0.00	0.00	0.00	0.00	0.01	0.10	0.00	0.01	0.03	0.16	0.01	0.04
Bacteria|Firmicutes|Bacilli|Bacillales|Bacil	0.02	0.04	0.01	0.02	0.02	0.05	0.02	0.04	0.24	0.59	0.36	0.89	0.13	0.24	0.24	0.50
Bacteria|Firmicutes|Bacilli|Bacillales|Bacil	0.00	0.00	0.00	0.00	0.00	0.00	0.00	0.00	0.02	0.11	0.00	0.00	0.01	0.04	0.04	0.18
Bacteria|Firmicutes|Bacilli|Bacillales|Bacil	0.00	0.00	0.00	0.00	0.00	0.00	0.00	0.00	0.00	0.00	0.00	0.00	0.00	0.00	0.00	0.00
Bacteria|Firmicutes|Bacilli|Bacillales|Bacil	0.00	0.00	0.00	0.00	0.00	0.00	0.00	0.00	0.00	0.00	0.00	0.00	0.00	0.00	0.00	0.00
Bacteria|Firmicutes|Bacilli|Bacillales|Bacil	0.00	0.00	0.00	0.00	0.00	0.00	0.00	0.00	0.00	0.02	0.00	0.00	0.01	0.02	0.01	0.04
Bacteria|Firmicutes|Bacilli|Bacillales|Lister	0.00	0.00	0.00	0.00	0.00	0.00	0.00	0.00	0.00	0.01	0.00	0.00	0.00	0.01	0.00	0.00
Bacteria|Firmicutes|Bacilli|Bacillales|Paeni	0.00	0.00	0.00	0.00	0.00	0.00	0.00	0.00	0.00	0.01	0.00	0.00	0.00	0.01	0.00	0.00
Bacteria|Firmicutes|Bacilli|Bacillales|Paeni	0.00	0.00	0.00	0.00	0.00	0.00	0.00	0.00	0.27	1.96	0.04	0.12	0.04	0.15	0.75	3.46
Bacteria|Firmicutes|Bacilli|Bacillales|Paeni	0.00	0.00	0.00	0.00	0.00	0.00	0.00	0.00	0.00	0.00	0.00	0.00	0.00	0.01	0.00	0.00
Bacteria|Firmicutes|Bacilli|Bacillales|Planc	0.00	0.00	0.00	0.00	0.00	0.00	0.00	0.00	0.00	0.00	0.00	0.00	0.00	0.00	0.00	0.00
Bacteria|Firmicutes|Bacilli|Bacillales|Planc	0.00	0.00	0.00	0.00	0.00	0.00	0.00	0.00	0.00	0.00	0.00	0.00	0.00	0.00	0.00	0.00
Bacteria|Firmicutes|Bacilli|Bacillales|Planc	0.00	0.00	0.00	0.00	0.00	0.00	0.00	0.00	0.00	0.00	0.00	0.00	0.00	0.00	0.00	0.00
Bacteria|Firmicutes|Bacilli|Bacillales|Planc	0.00	0.00	0.00	0.00	0.00	0.00	0.00	0.00	0.01	0.02	0.00	0.01	0.01	0.02	0.01	0.03
Bacteria|Firmicutes|Bacilli|Bacillales|Sporo	0.00	0.00	0.00	0.00	0.00	0.00	0.00	0.00	0.00	0.00	0.00	0.00	0.00	0.00	0.00	0.00
Bacteria|Firmicutes|Bacilli|Bacillales|Staph	0.00	0.00	0.00	0.00	0.00	0.00	0.00	0.00	0.00	0.01	0.00	0.00	0.00	0.01	0.00	0.01
Bacteria|Firmicutes|Bacilli|Bacillales|Staph	0.00	0.00	0.00	0.00	0.00	0.00	0.00	0.00	0.00	0.01	0.00	0.00	0.00	0.01	0.00	0.00
Bacteria|Firmicutes|Bacilli|Bacillales|Staph	0.00	0.00	0.00	0.00	0.00	0.00	0.00	0.00	0.00	0.00	0.00	0.00	0.00	0.00	0.00	0.00
Bacteria|Firmicutes|Bacilli|Bacillales|Staph	0.01	0.05	0.00	0.00	0.00	0.00	0.02	0.09	0.48	1.44	0.26	0.33	0.64	2.22	0.50	0.97
Bacteria|Firmicutes|Bacilli|Bacillales|Therr	0.00	0.00	0.00	0.00	0.00	0.00	0.00	0.00	0.00	0.00	0.00	0.00	0.00	0.00	0.00	0.00
Bacteria|Firmicutes|Bacilli|Bacillales_Othe	0.00	0.00	0.00	0.00	0.00	0.00	0.00	0.00	0.01	0.01	0.00	0.01	0.01	0.01	0.01	0.02

<table>
<tr><th rowspan="3">Microbial clades</th><th colspan="8">Oral Cavity</th><th colspan="8">Esophagus</th></tr>
<tr><th colspan="2">All sites</th><th colspan="2">Sa</th><th colspan="2">TD</th><th colspan="2">SP</th><th colspan="2">All sites</th><th colspan="2">UE</th><th colspan="2">ME</th><th colspan="2">LE</th></tr>
<tr><th>Ave</th><th>SD</th><th>Ave</th><th>SD</th><th>Ave</th><th>SD</th><th>Ave</th><th>SD</th><th>Ave</th><th>SD</th><th>Ave</th><th>SD</th><th>Ave</th><th>SD</th><th>Ave</th><th>SD</th></tr>
<tr><td>Bacteria|Firmicutes|Bacilli|Gemellales|Gen</td><td>0.02</td><td>0.07</td><td>0.01</td><td>0.02</td><td>0.03</td><td>0.10</td><td>0.02</td><td>0.06</td><td>0.04</td><td>0.11</td><td>0.03</td><td>0.07</td><td>0.06</td><td>0.16</td><td>0.03</td><td>0.06</td></tr>
<tr><td>Bacteria|Firmicutes|Bacilli|Gemellales|Gen</td><td>0.00</td><td>0.00</td><td>0.00</td><td>0.00</td><td>0.00</td><td>0.00</td><td>0.00</td><td>0.00</td><td>0.00</td><td>0.00</td><td>0.00</td><td>0.01</td><td>0.00</td><td>0.00</td><td>0.00</td><td>0.00</td></tr>
<tr><td>Bacteria|Firmicutes|Bacilli|Gemellales|Gen</td><td>0.21</td><td>0.33</td><td>0.05</td><td>0.04</td><td>0.37</td><td>0.31</td><td>0.21</td><td>0.42</td><td>2.93</td><td>3.45</td><td>3.57</td><td>4.53</td><td>3.00</td><td>3.08</td><td>2.22</td><td>2.49</td></tr>
<tr><td>Bacteria|Firmicutes|Bacilli|Gemellales_Oth</td><td>0.00</td><td>0.00</td><td>0.00</td><td>0.00</td><td>0.00</td><td>0.00</td><td>0.00</td><td>0.00</td><td>0.00</td><td>0.00</td><td>0.00</td><td>0.00</td><td>0.00</td><td>0.00</td><td>0.00</td><td>0.00</td></tr>
<tr><td>Bacteria|Firmicutes|Bacilli|Lactobacillales|</td><td>0.09</td><td>0.11</td><td>0.07</td><td>0.07</td><td>0.07</td><td>0.14</td><td>0.11</td><td>0.12</td><td>0.04</td><td>0.09</td><td>0.03</td><td>0.06</td><td>0.04</td><td>0.08</td><td>0.06</td><td>0.13</td></tr>
<tr><td>Bacteria|Firmicutes|Bacilli|Lactobacillales|</td><td>0.05</td><td>0.16</td><td>0.05</td><td>0.17</td><td>0.05</td><td>0.13</td><td>0.06</td><td>0.17</td><td>0.10</td><td>0.36</td><td>0.06</td><td>0.14</td><td>0.20</td><td>0.57</td><td>0.04</td><td>0.08</td></tr>
<tr><td>Bacteria|Firmicutes|Bacilli|Lactobacillales|</td><td>0.00</td><td>0.00</td><td>0.00</td><td>0.00</td><td>0.00</td><td>0.00</td><td>0.00</td><td>0.00</td><td>0.00</td><td>0.00</td><td>0.00</td><td>0.00</td><td>0.00</td><td>0.00</td><td>0.00</td><td>0.00</td></tr>
<tr><td>Bacteria|Firmicutes|Bacilli|Lactobacillales|</td><td>0.00</td><td>0.00</td><td>0.00</td><td>0.00</td><td>0.00</td><td>0.00</td><td>0.00</td><td>0.00</td><td>0.00</td><td>0.02</td><td>0.00</td><td>0.00</td><td>0.00</td><td>0.02</td><td>0.01</td><td>0.02</td></tr>
<tr><td>Bacteria|Firmicutes|Bacilli|Lactobacillales|</td><td>0.00</td><td>0.00</td><td>0.00</td><td>0.00</td><td>0.00</td><td>0.00</td><td>0.00</td><td>0.00</td><td>0.01</td><td>0.07</td><td>0.03</td><td>0.12</td><td>0.00</td><td>0.01</td><td>0.00</td><td>0.00</td></tr>
<tr><td>Bacteria|Firmicutes|Bacilli|Lactobacillales|</td><td>0.00</td><td>0.00</td><td>0.00</td><td>0.00</td><td>0.00</td><td>0.00</td><td>0.00</td><td>0.00</td><td>0.00</td><td>0.00</td><td>0.00</td><td>0.01</td><td>0.00</td><td>0.00</td><td>0.00</td><td>0.00</td></tr>
<tr><td>Bacteria|Firmicutes|Bacilli|Lactobacillales|</td><td>0.00</td><td>0.00</td><td>0.00</td><td>0.00</td><td>0.00</td><td>0.01</td><td>0.00</td><td>0.00</td><td>0.00</td><td>0.00</td><td>0.00</td><td>0.00</td><td>0.00</td><td>0.00</td><td>0.00</td><td>0.00</td></tr>
<tr><td>Bacteria|Firmicutes|Bacilli|Lactobacillales|</td><td>0.00</td><td>0.00</td><td>0.00</td><td>0.00</td><td>0.00</td><td>0.00</td><td>0.00</td><td>0.00</td><td>0.01</td><td>0.03</td><td>0.01</td><td>0.04</td><td>0.01</td><td>0.01</td><td>0.01</td><td>0.02</td></tr>
<tr><td>Bacteria|Firmicutes|Bacilli|Lactobacillales|</td><td>0.00</td><td>0.00</td><td>0.00</td><td>0.00</td><td>0.00</td><td>0.00</td><td>0.00</td><td>0.00</td><td>0.00</td><td>0.01</td><td>0.00</td><td>0.01</td><td>0.00</td><td>0.01</td><td>0.00</td><td>0.01</td></tr>
<tr><td>Bacteria|Firmicutes|Bacilli|Lactobacillales|</td><td>0.00</td><td>0.00</td><td>0.00</td><td>0.00</td><td>0.00</td><td>0.00</td><td>0.00</td><td>0.00</td><td>0.00</td><td>0.00</td><td>0.00</td><td>0.00</td><td>0.00</td><td>0.00</td><td>0.00</td><td>0.00</td></tr>
<tr><td>Bacteria|Firmicutes|Bacilli|Lactobacillales|</td><td>1.18</td><td>1.59</td><td>0.45</td><td>0.28</td><td>2.76</td><td>1.85</td><td>0.29</td><td>0.51</td><td>0.80</td><td>0.70</td><td>0.93</td><td>0.84</td><td>0.83</td><td>0.64</td><td>0.64</td><td>0.61</td></tr>
<tr><td>Bacteria|Firmicutes|Bacilli|Lactobacillales|</td><td>0.00</td><td>0.00</td><td>0.00</td><td>0.00</td><td>0.00</td><td>0.00</td><td>0.00</td><td>0.00</td><td>0.00</td><td>0.01</td><td>0.00</td><td>0.00</td><td>0.00</td><td>0.00</td><td>0.00</td><td>0.02</td></tr>
<tr><td>Bacteria|Firmicutes|Bacilli|Lactobacillales|</td><td>0.00</td><td>0.00</td><td>0.00</td><td>0.00</td><td>0.00</td><td>0.00</td><td>0.00</td><td>0.00</td><td>0.00</td><td>0.00</td><td>0.00</td><td>0.00</td><td>0.00</td><td>0.00</td><td>0.00</td><td>0.00</td></tr>
<tr><td>Bacteria|Firmicutes|Bacilli|Lactobacillales|</td><td>0.00</td><td>0.00</td><td>0.00</td><td>0.00</td><td>0.00</td><td>0.00</td><td>0.00</td><td>0.00</td><td>0.00</td><td>0.01</td><td>0.00</td><td>0.00</td><td>0.00</td><td>0.00</td><td>0.00</td><td>0.02</td></tr>
<tr><td>Bacteria|Firmicutes|Bacilli|Lactobacillales|</td><td>0.00</td><td>0.00</td><td>0.00</td><td>0.00</td><td>0.00</td><td>0.01</td><td>0.00</td><td>0.00</td><td>0.02</td><td>0.08</td><td>0.00</td><td>0.01</td><td>0.04</td><td>0.12</td><td>0.01</td><td>0.04</td></tr>
<tr><td>Bacteria|Firmicutes|Bacilli|Lactobacillales|</td><td>0.00</td><td>0.00</td><td>0.00</td><td>0.00</td><td>0.00</td><td>0.00</td><td>0.00</td><td>0.00</td><td>0.00</td><td>0.00</td><td>0.00</td><td>0.00</td><td>0.00</td><td>0.00</td><td>0.00</td><td>0.00</td></tr>
<tr><td>Bacteria|Firmicutes|Bacilli|Lactobacillales|</td><td>0.00</td><td>0.00</td><td>0.00</td><td>0.01</td><td>0.00</td><td>0.00</td><td>0.00</td><td>0.00</td><td>0.01</td><td>0.03</td><td>0.01</td><td>0.01</td><td>0.01</td><td>0.01</td><td>0.02</td><td>0.05</td></tr>
<tr><td>Bacteria|Firmicutes|Bacilli|Lactobacillales|</td><td>0.00</td><td>0.00</td><td>0.00</td><td>0.00</td><td>0.00</td><td>0.00</td><td>0.00</td><td>0.00</td><td>0.02</td><td>0.06</td><td>0.01</td><td>0.02</td><td>0.01</td><td>0.03</td><td>0.04</td><td>0.09</td></tr>
<tr><td>Bacteria|Firmicutes|Bacilli|Lactobacillales|</td><td>0.42</td><td>0.88</td><td>0.28</td><td>0.55</td><td>0.57</td><td>1.16</td><td>0.40</td><td>0.84</td><td>0.91</td><td>1.59</td><td>0.94</td><td>1.70</td><td>1.10</td><td>1.86</td><td>0.68</td><td>1.09</td></tr>
<tr><td>Bacteria|Firmicutes|Bacilli|Lactobacillales|</td><td>0.00</td><td>0.00</td><td>0.00</td><td>0.00</td><td>0.00</td><td>0.01</td><td>0.00</td><td>0.00</td><td>0.01</td><td>0.03</td><td>0.00</td><td>0.00</td><td>0.01</td><td>0.05</td><td>0.00</td><td>0.01</td></tr>
<tr><td>Bacteria|Firmicutes|Bacilli|Lactobacillales|</td><td>0.00</td><td>0.00</td><td>0.00</td><td>0.00</td><td>0.00</td><td>0.00</td><td>0.00</td><td>0.00</td><td>0.00</td><td>0.00</td><td>0.00</td><td>0.00</td><td>0.00</td><td>0.00</td><td>0.00</td><td>0.01</td></tr>
<tr><td>Bacteria|Firmicutes|Bacilli|Lactobacillales|</td><td>4.77</td><td>5.40</td><td>2.93</td><td>1.51</td><td>7.76</td><td>5.60</td><td>3.58</td><td>6.52</td><td>24.59</td><td>19.70</td><td>24.97</td><td>17.93</td><td>24.89</td><td>18.96</td><td>23.88</td><td>22.83</td></tr>
<tr><td>Bacteria|Firmicutes|Bacilli|Lactobacillales|</td><td>0.00</td><td>0.00</td><td>0.00</td><td>0.00</td><td>0.00</td><td>0.00</td><td>0.00</td><td>0.00</td><td>0.00</td><td>0.00</td><td>0.00</td><td>0.00</td><td>0.00</td><td>0.00</td><td>0.00</td><td>0.00</td></tr>
<tr><td>Bacteria|Firmicutes|Bacilli|Lactobacillales_</td><td>0.01</td><td>0.02</td><td>0.01</td><td>0.01</td><td>0.02</td><td>0.02</td><td>0.00</td><td>0.01</td><td>0.01</td><td>0.02</td><td>0.00</td><td>0.01</td><td>0.01</td><td>0.03</td><td>0.01</td><td>0.02</td></tr>
<tr><td>Bacteria|Firmicutes|Bacilli|Turicibacterales</td><td>0.00</td><td>0.00</td><td>0.00</td><td>0.00</td><td>0.00</td><td>0.00</td><td>0.00</td><td>0.00</td><td>0.01</td><td>0.02</td><td>0.01</td><td>0.02</td><td>0.00</td><td>0.02</td><td>0.01</td><td>0.03</td></tr>
</table>

Microbial clades	Oral Cavity								Esophagus							
	All sites		Sa		TD		SP		All sites		UE		ME		LE	
	Ave	SD	Ave	SD	Ave	SD	Ave	SD	Ave	SD	Ave	SD	Ave	SD	Ave	SD
Bacteria|Firmicutes|Bacilli_Other	0.00	0.01	0.00	0.01	0.00	0.00	0.00	0.00	0.00	0.00	0.00	0.00	0.00	0.00	0.00	0.00
Bacteria|Firmicutes|Clostridia|Clostridiales	0.00	0.00	0.00	0.00	0.00	0.00	0.00	0.00	0.00	0.00	0.00	0.00	0.00	0.00	0.00	0.00
Bacteria|Firmicutes|Clostridia|Clostridiales	0.05	0.13	0.01	0.01	0.11	0.22	0.03	0.04	0.01	0.04	0.02	0.05	0.02	0.06	0.00	0.01
Bacteria|Firmicutes|Clostridia|Clostridiales	0.14	0.38	0.01	0.01	0.36	0.60	0.03	0.05	0.09	0.11	0.08	0.10	0.11	0.15	0.06	0.07
Bacteria|Firmicutes|Clostridia|Clostridiales	0.02	0.06	0.00	0.01	0.00	0.00	0.06	0.09	0.01	0.03	0.02	0.06	0.01	0.01	0.00	0.01
Bacteria|Firmicutes|Clostridia|Clostridiales	0.40	0.46	0.20	0.12	0.51	0.34	0.48	0.68	0.19	0.22	0.17	0.19	0.19	0.21	0.22	0.25
Bacteria|Firmicutes|Clostridia|Clostridiales	0.00	0.00	0.00	0.00	0.00	0.00	0.00	0.00	0.00	0.00	0.00	0.00	0.00	0.00	0.00	0.00
Bacteria|Firmicutes|Clostridia|Clostridiales	0.01	0.03	0.01	0.03	0.01	0.02	0.02	0.05	0.02	0.05	0.02	0.04	0.03	0.06	0.02	0.05
Bacteria|Firmicutes|Clostridia|Clostridiales	0.00	0.02	0.01	0.02	0.00	0.00	0.01	0.02	0.01	0.04	0.01	0.02	0.01	0.02	0.02	0.05
Bacteria|Firmicutes|Clostridia|Clostridiales	0.00	0.00	0.00	0.00	0.00	0.00	0.00	0.00	0.00	0.00	0.00	0.00	0.00	0.00	0.00	0.00
Bacteria|Firmicutes|Clostridia|Clostridiales	0.13	0.29	0.03	0.05	0.23	0.41	0.13	0.25	0.06	0.10	0.06	0.07	0.06	0.08	0.07	0.14
Bacteria|Firmicutes|Clostridia|Clostridiales	0.01	0.02	0.01	0.01	0.01	0.02	0.01	0.03	0.03	0.10	0.02	0.05	0.02	0.05	0.05	0.17
Bacteria|Firmicutes|Clostridia|Clostridiales	0.00	0.00	0.00	0.00	0.00	0.00	0.00	0.00	0.00	0.00	0.00	0.00	0.00	0.00	0.00	0.00
Bacteria|Firmicutes|Clostridia|Clostridiales	0.00	0.00	0.00	0.00	0.00	0.00	0.00	0.00	0.00	0.00	0.00	0.00	0.00	0.00	0.00	0.00
Bacteria|Firmicutes|Clostridia|Clostridiales	0.00	0.00	0.00	0.00	0.00	0.00	0.00	0.00	0.00	0.00	0.00	0.00	0.00	0.00	0.00	0.00
Bacteria|Firmicutes|Clostridia|Clostridiales	0.01	0.02	0.00	0.01	0.00	0.00	0.02	0.04	0.00	0.00	0.00	0.00	0.00	0.00	0.00	0.00
Bacteria|Firmicutes|Clostridia|Clostridiales	0.00	0.00	0.00	0.00	0.00	0.00	0.00	0.00	0.00	0.00	0.00	0.00	0.00	0.00	0.00	0.00
Bacteria|Firmicutes|Clostridia|Clostridiales	0.00	0.00	0.00	0.00	0.00	0.00	0.00	0.00	0.11	0.36	0.07	0.18	0.13	0.46	0.13	0.38
Bacteria|Firmicutes|Clostridia|Clostridiales	0.00	0.00	0.00	0.00	0.00	0.00	0.00	0.00	0.00	0.01	0.00	0.00	0.00	0.01	0.00	0.00
Bacteria|Firmicutes|Clostridia|Clostridiales	0.02	0.06	0.01	0.04	0.03	0.08	0.02	0.06	0.07	0.23	0.03	0.05	0.04	0.10	0.14	0.39
Bacteria|Firmicutes|Clostridia|Clostridiales	0.00	0.00	0.00	0.00	0.00	0.00	0.00	0.00	0.00	0.00	0.00	0.00	0.00	0.00	0.00	0.00
Bacteria|Firmicutes|Clostridia|Clostridiales	0.00	0.00	0.00	0.00	0.00	0.00	0.00	0.00	0.00	0.00	0.00	0.00	0.00	0.00	0.00	0.00
Bacteria|Firmicutes|Clostridia|Clostridiales	0.00	0.00	0.00	0.00	0.00	0.00	0.00	0.00	0.00	0.00	0.00	0.01	0.00	0.01	0.00	0.00
Bacteria|Firmicutes|Clostridia|Clostridiales	0.00	0.00	0.00	0.00	0.00	0.01	0.00	0.00	0.08	0.20	0.05	0.08	0.06	0.15	0.12	0.30
Bacteria|Firmicutes|Clostridia|Clostridiales	0.00	0.00	0.00	0.00	0.00	0.00	0.00	0.00	0.00	0.01	0.00	0.01	0.00	0.00	0.00	0.00
Bacteria|Firmicutes|Clostridia|Clostridiales	0.00	0.00	0.00	0.00	0.00	0.00	0.00	0.00	0.00	0.00	0.00	0.00	0.00	0.00	0.00	0.00
Bacteria|Firmicutes|Clostridia|Clostridiales	0.00	0.00	0.00	0.00	0.00	0.00	0.00	0.00	0.00	0.00	0.00	0.00	0.00	0.00	0.00	0.00
Bacteria|Firmicutes|Clostridia|Clostridiales	0.05	0.36	0.01	0.02	0.01	0.04	0.13	0.64	0.00	0.01	0.01	0.02	0.00	0.00	0.00	0.01
Bacteria|Firmicutes|Clostridia|Clostridiales	0.00	0.01	0.00	0.01	0.00	0.00	0.00	0.00	0.02	0.04	0.01	0.01	0.02	0.04	0.02	0.04

Microbial clades	Oral Cavity								Esophagus							
	All sites		Sa		TD		SP		All sites		UE		ME		LE	
	Ave	SD	Ave	SD	Ave	SD	Ave	SD	Ave	SD	Ave	SD	Ave	SD	Ave	SD
Bacteria|Firmicutes|Clostridia|Clostridiales	0.00	0.00	0.00	0.00	0.00	0.00	0.00	0.00	0.01	0.06	0.00	0.00	0.02	0.09	0.01	0.02
Bacteria|Firmicutes|Clostridia|Clostridiales	0.00	0.01	0.01	0.02	0.01	0.01	0.00	0.01	0.30	0.78	0.12	0.11	0.31	0.71	0.48	1.15
Bacteria|Firmicutes|Clostridia|Clostridiales	0.05	0.07	0.09	0.07	0.06	0.06	0.01	0.01	0.03	0.09	0.03	0.08	0.04	0.09	0.03	0.11
Bacteria|Firmicutes|Clostridia|Clostridiales	0.23	0.32	0.03	0.03	0.27	0.18	0.39	0.45	0.04	0.06	0.04	0.05	0.05	0.08	0.02	0.03
Bacteria|Firmicutes|Clostridia|Clostridiales	0.00	0.02	0.01	0.03	0.00	0.01	0.00	0.00	0.12	0.28	0.09	0.19	0.12	0.24	0.15	0.40
Bacteria|Firmicutes|Clostridia|Clostridiales	0.00	0.01	0.00	0.01	0.00	0.01	0.00	0.01	0.09	0.36	0.03	0.05	0.13	0.57	0.10	0.19
Bacteria|Firmicutes|Clostridia|Clostridiales	0.00	0.00	0.00	0.00	0.00	0.00	0.00	0.00	0.00	0.00	0.00	0.00	0.00	0.00	0.00	0.00
Bacteria|Firmicutes|Clostridia|Clostridiales	0.00	0.01	0.00	0.01	0.00	0.01	0.00	0.00	0.02	0.14	0.00	0.00	0.05	0.24	0.01	0.02
Bacteria|Firmicutes|Clostridia|Clostridiales	0.00	0.01	0.00	0.02	0.00	0.00	0.00	0.01	0.02	0.04	0.01	0.02	0.02	0.06	0.02	0.05
Bacteria|Firmicutes|Clostridia|Clostridiales	0.00	0.01	0.00	0.01	0.00	0.00	0.00	0.00	0.04	0.26	0.00	0.01	0.01	0.03	0.10	0.47
Bacteria|Firmicutes|Clostridia|Clostridiales	0.56	1.07	0.49	0.52	1.07	1.64	0.09	0.20	0.23	0.74	0.20	0.41	0.29	1.04	0.20	0.61
Bacteria|Firmicutes|Clostridia|Clostridiales	0.01	0.02	0.01	0.04	0.01	0.01	0.00	0.01	0.06	0.18	0.01	0.02	0.05	0.18	0.11	0.24
Bacteria|Firmicutes|Clostridia|Clostridiales	0.15	0.27	0.24	0.28	0.09	0.16	0.10	0.31	0.11	0.31	0.04	0.05	0.12	0.38	0.17	0.38
Bacteria|Firmicutes|Clostridia|Clostridiales	0.00	0.01	0.01	0.01	0.00	0.00	0.00	0.01	0.07	0.17	0.05	0.08	0.05	0.11	0.13	0.27
Bacteria|Firmicutes|Clostridia|Clostridiales	0.80	0.71	0.23	0.19	1.29	0.80	0.87	0.53	0.66	1.13	0.47	0.30	0.64	1.08	0.86	1.63
Bacteria|Firmicutes|Clostridia|Clostridiales	0.00	0.00	0.00	0.00	0.00	0.00	0.00	0.00	0.00	0.00	0.00	0.00	0.00	0.00	0.00	0.00
Bacteria|Firmicutes|Clostridia|Clostridiales	0.04	0.07	0.02	0.01	0.07	0.11	0.02	0.02	0.01	0.03	0.02	0.04	0.01	0.03	0.01	0.02
Bacteria|Firmicutes|Clostridia|Clostridiales	0.00	0.00	0.00	0.00	0.00	0.00	0.00	0.00	0.00	0.00	0.00	0.00	0.00	0.00	0.00	0.00
Bacteria|Firmicutes|Clostridia|Clostridiales	0.00	0.00	0.00	0.00	0.00	0.00	0.00	0.00	0.00	0.01	0.00	0.00	0.00	0.00	0.00	0.01
Bacteria|Firmicutes|Clostridia|Clostridiales	0.14	0.28	0.03	0.03	0.11	0.19	0.31	0.40	0.07	0.10	0.06	0.08	0.08	0.13	0.06	0.09
Bacteria|Firmicutes|Clostridia|Clostridiales	0.18	0.29	0.04	0.04	0.29	0.21	0.20	0.42	0.19	0.26	0.19	0.20	0.20	0.35	0.19	0.19
Bacteria|Firmicutes|Clostridia|Clostridiales	0.07	0.13	0.01	0.01	0.05	0.11	0.15	0.17	0.02	0.05	0.02	0.04	0.02	0.05	0.03	0.06
Bacteria|Firmicutes|Clostridia|Clostridiales	0.00	0.00	0.00	0.00	0.00	0.00	0.00	0.00	0.00	0.00	0.00	0.00	0.00	0.00	0.00	0.00
Bacteria|Firmicutes|Clostridia|Clostridiales	0.00	0.00	0.00	0.00	0.00	0.00	0.00	0.00	0.00	0.00	0.00	0.00	0.00	0.00	0.00	0.00
Bacteria|Firmicutes|Clostridia|Clostridiales	0.02	0.11	0.04	0.18	0.02	0.05	0.01	0.03	0.05	0.14	0.02	0.04	0.05	0.09	0.09	0.22
Bacteria|Firmicutes|Clostridia|Clostridiales	0.01	0.02	0.01	0.03	0.01	0.02	0.00	0.01	0.34	0.75	0.29	0.50	0.33	0.80	0.41	0.92
Bacteria|Firmicutes|Clostridia|Clostridiales	0.00	0.01	0.00	0.02	0.00	0.00	0.00	0.00	0.06	0.13	0.08	0.17	0.04	0.10	0.05	0.10
Bacteria|Firmicutes|Clostridia|Clostridiales	0.00	0.00	0.00	0.00	0.00	0.00	0.00	0.00	0.00	0.00	0.00	0.00	0.00	0.00	0.00	0.00
Bacteria|Firmicutes|Clostridia|Clostridiales	0.03	0.11	0.05	0.18	0.02	0.05	0.01	0.05	0.34	0.64	0.28	0.50	0.32	0.54	0.43	0.85

Microbial clades	Oral Cavity								Esophagus							
	All sites		Sa		TD		SP		All sites		UE		ME		LE	
	Ave	SD	Ave	SD	Ave	SD	Ave	SD	Ave	SD	Ave	SD	Ave	SD	Ave	SD
Bacteria|Firmicutes|Clostridia|Clostridiales	0.01	0.02	0.00	0.01	0.00	0.00	0.02	0.04	0.00	0.00	0.00	0.00	0.00	0.00	0.00	0.00
Bacteria|Firmicutes|Clostridia|Clostridiales	0.00	0.00	0.00	0.00	0.00	0.00	0.00	0.00	0.00	0.00	0.00	0.00	0.00	0.00	0.00	0.00
Bacteria|Firmicutes|Clostridia|Clostridiales	0.24	0.46	0.16	0.23	0.15	0.32	0.41	0.68	0.06	0.07	0.07	0.09	0.05	0.06	0.04	0.06
Bacteria|Firmicutes|Clostridia|Clostridiales	0.00	0.00	0.00	0.00	0.00	0.00	0.00	0.00	0.00	0.00	0.00	0.00	0.00	0.00	0.00	0.00
Bacteria|Firmicutes|Clostridia|Clostridiales	0.30	0.49	0.24	0.35	0.56	0.67	0.08	0.14	0.14	0.40	0.09	0.09	0.09	0.12	0.24	0.69
Bacteria|Firmicutes|Clostridia|Clostridiales	0.00	0.00	0.00	0.00	0.00	0.00	0.00	0.00	0.00	0.00	0.00	0.00	0.00	0.00	0.00	0.00
Bacteria|Firmicutes|Clostridia|Clostridiales	0.06	0.11	0.09	0.14	0.00	0.00	0.09	0.12	0.00	0.01	0.00	0.01	0.01	0.02	0.00	0.00
Bacteria|Firmicutes|Clostridia|Clostridiales	1.13	1.51	1.17	0.91	0.21	0.25	2.03	2.13	0.09	0.16	0.10	0.21	0.08	0.10	0.09	0.15
Bacteria|Firmicutes|Clostridia|Clostridiales	0.00	0.00	0.00	0.00	0.00	0.00	0.00	0.01	0.00	0.00	0.00	0.00	0.00	0.00	0.00	0.00
Bacteria|Firmicutes|Clostridia|Clostridiales	0.00	0.00	0.00	0.00	0.00	0.00	0.00	0.00	0.00	0.01	0.00	0.00	0.00	0.00	0.00	0.02
Bacteria|Firmicutes|Clostridia|Clostridiales	1.66	2.59	0.19	0.20	4.11	3.20	0.64	0.74	1.92	5.97	1.27	1.63	2.60	8.19	1.79	5.93
Bacteria|Firmicutes|Clostridia|Clostridiales	0.00	0.00	0.00	0.00	0.00	0.00	0.00	0.00	0.00	0.00	0.00	0.00	0.00	0.00	0.00	0.00
Bacteria|Firmicutes|Clostridia|Clostridiales	0.06	0.18	0.08	0.14	0.04	0.16	0.08	0.22	0.01	0.03	0.02	0.04	0.01	0.02	0.01	0.02
Bacteria|Firmicutes|Clostridia|Clostridiales	0.00	0.01	0.01	0.02	0.00	0.00	0.00	0.01	0.08	0.18	0.14	0.29	0.05	0.09	0.06	0.09
Bacteria|Firmicutes|Clostridia|Thermoanae	0.00	0.00	0.00	0.00	0.00	0.00	0.00	0.00	0.00	0.01	0.00	0.00	0.00	0.00	0.00	0.02
Bacteria|Firmicutes|Erysipelotrichi|Erysipe	0.00	0.01	0.00	0.01	0.00	0.00	0.00	0.00	0.01	0.04	0.00	0.01	0.00	0.01	0.03	0.08
Bacteria|Firmicutes|Erysipelotrichi|Erysipe	0.00	0.01	0.00	0.00	0.00	0.00	0.00	0.01	0.40	1.16	0.12	0.15	0.38	0.82	0.70	1.83
Bacteria|Firmicutes|Erysipelotrichi|Erysipe	0.42	0.63	0.22	0.22	0.99	0.79	0.04	0.05	0.08	0.10	0.12	0.14	0.07	0.08	0.06	0.06
Bacteria|Firmicutes|Erysipelotrichi|Erysipe	0.00	0.00	0.00	0.00	0.00	0.00	0.00	0.00	0.00	0.01	0.00	0.00	0.00	0.00	0.00	0.01
Bacteria|Firmicutes|Erysipelotrichi|Erysipe	0.00	0.00	0.00	0.00	0.00	0.00	0.00	0.00	0.00	0.00	0.00	0.00	0.00	0.00	0.00	0.00
Bacteria|Firmicutes|Erysipelotrichi|Erysipe	0.00	0.00	0.00	0.00	0.00	0.00	0.00	0.00	0.00	0.00	0.00	0.00	0.00	0.00	0.00	0.00
Bacteria|Firmicutes|Erysipelotrichi|Erysipe	0.00	0.00	0.00	0.00	0.00	0.00	0.00	0.00	0.00	0.00	0.00	0.00	0.00	0.00	0.00	0.00
Bacteria|Firmicutes|Erysipelotrichi|Erysipe	0.00	0.02	0.00	0.00	0.00	0.00	0.01	0.03	0.00	0.00	0.00	0.00	0.00	0.00	0.00	0.00
Bacteria|Firmicutes|Erysipelotrichi|Erysipe	0.01	0.02	0.00	0.01	0.01	0.01	0.02	0.03	0.00	0.00	0.00	0.00	0.00	0.01	0.00	0.00
Bacteria|Firmicutes|Erysipelotrichi|Erysipe	0.00	0.00	0.00	0.00	0.00	0.00	0.00	0.00	0.00	0.00	0.00	0.00	0.00	0.00	0.00	0.00
Bacteria|Firmicutes|Erysipelotrichi|Erysipe	0.01	0.05	0.02	0.08	0.00	0.01	0.01	0.03	0.01	0.02	0.00	0.01	0.01	0.02	0.00	0.01
Bacteria|Firmicutes_Other	0.00	0.00	0.00	0.00	0.00	0.00	0.00	0.00	0.00	0.00	0.00	0.00	0.00	0.00	0.00	0.00
Bacteria|Fusobacteria|Fusobacteriia|Fusoba	2.49	2.42	0.28	0.27	3.32	2.62	3.93	1.77	0.95	0.79	0.96	0.68	1.13	1.03	0.73	0.50
Bacteria|Fusobacteria|Fusobacteriia|Fusoba	1.24	1.54	0.12	0.14	1.29	0.97	2.36	1.95	0.19	0.19	0.21	0.18	0.21	0.22	0.15	0.17

Microbial clades	Oral Cavity								Esophagus							
	All sites		Sa		TD		SP		All sites		UE		ME		LE	
	Ave	SD	Ave	SD	Ave	SD	Ave	SD	Ave	SD	Ave	SD	Ave	SD	Ave	SD
Bacteria|Fusobacteria|Fusobacteriia|Fusoba	0.02	0.06	0.02	0.05	0.03	0.07	0.02	0.07	0.03	0.09	0.02	0.04	0.03	0.08	0.05	0.12
Bacteria|Fusobacteria|Fusobacteriia|Fusoba	0.00	0.00	0.00	0.00	0.00	0.00	0.00	0.00	0.00	0.00	0.00	0.00	0.00	0.00	0.00	0.00
Bacteria|Fusobacteria|Fusobacteriia|Fusoba	0.00	0.01	0.00	0.00	0.01	0.02	0.00	0.01	0.05	0.19	0.06	0.27	0.06	0.19	0.02	0.07
Bacteria|Lentisphaerae|[Lentisphaeria]|Z20	0.00	0.00	0.00	0.00	0.00	0.00	0.00	0.00	0.00	0.00	0.00	0.00	0.00	0.00	0.00	0.00
Bacteria|Planctomycetes|Planctomycetia|G	0.00	0.00	0.00	0.00	0.00	0.00	0.00	0.00	0.00	0.00	0.00	0.00	0.00	0.00	0.00	0.00
Bacteria|Proteobacteria|Alphaproteobacteri	0.00	0.00	0.00	0.00	0.00	0.00	0.00	0.00	0.00	0.00	0.00	0.00	0.00	0.00	0.00	0.00
Bacteria|Proteobacteria|Alphaproteobacteri	0.00	0.00	0.00	0.00	0.00	0.00	0.00	0.00	0.00	0.00	0.00	0.00	0.00	0.00	0.00	0.00
Bacteria|Proteobacteria|Alphaproteobacteri	0.00	0.00	0.00	0.00	0.00	0.00	0.00	0.00	0.30	2.01	0.04	0.06	0.06	0.09	0.82	3.55
Bacteria|Proteobacteria|Alphaproteobacteri	0.00	0.00	0.00	0.00	0.00	0.00	0.00	0.00	0.02	0.03	0.02	0.03	0.02	0.03	0.02	0.04
Bacteria|Proteobacteria|Alphaproteobacteri	0.00	0.00	0.00	0.00	0.00	0.00	0.00	0.00	0.01	0.02	0.00	0.01	0.00	0.01	0.02	0.04
Bacteria|Proteobacteria|Alphaproteobacteri	0.01	0.02	0.00	0.01	0.00	0.00	0.01	0.04	0.18	0.38	0.14	0.22	0.22	0.50	0.18	0.37
Bacteria|Proteobacteria|Alphaproteobacteri	0.00	0.00	0.00	0.00	0.00	0.00	0.00	0.00	0.00	0.00	0.00	0.00	0.00	0.01	0.00	0.00
Bacteria|Proteobacteria|Alphaproteobacteri	0.09	0.16	0.02	0.04	0.15	0.16	0.10	0.22	1.66	1.79	1.54	1.20	1.45	1.11	2.02	2.71
Bacteria|Proteobacteria|Alphaproteobacteri	0.00	0.00	0.00	0.00	0.00	0.00	0.00	0.00	0.00	0.00	0.00	0.00	0.00	0.00	0.00	0.00
Bacteria|Proteobacteria|Alphaproteobacteri	0.00	0.00	0.00	0.00	0.00	0.00	0.00	0.00	0.00	0.00	0.00	0.00	0.00	0.00	0.00	0.00
Bacteria|Proteobacteria|Alphaproteobacteri	0.00	0.00	0.00	0.00	0.00	0.00	0.00	0.00	0.00	0.03	0.00	0.00	0.00	0.00	0.01	0.05
Bacteria|Proteobacteria|Alphaproteobacteri	0.00	0.00	0.00	0.00	0.00	0.00	0.00	0.00	0.00	0.01	0.00	0.01	0.00	0.00	0.00	0.00
Bacteria|Proteobacteria|Alphaproteobacteri	0.00	0.00	0.00	0.00	0.00	0.00	0.00	0.00	0.00	0.00	0.00	0.00	0.00	0.00	0.00	0.00
Bacteria|Proteobacteria|Alphaproteobacteri	0.00	0.00	0.00	0.00	0.00	0.00	0.00	0.00	0.00	0.00	0.00	0.00	0.00	0.00	0.00	0.01
Bacteria|Proteobacteria|Alphaproteobacteri	0.00	0.00	0.00	0.00	0.00	0.00	0.00	0.00	0.00	0.01	0.00	0.01	0.00	0.00	0.00	0.00
Bacteria|Proteobacteria|Alphaproteobacteri	0.00	0.00	0.00	0.00	0.00	0.00	0.00	0.00	0.00	0.01	0.00	0.00	0.00	0.00	0.01	0.02
Bacteria|Proteobacteria|Alphaproteobacteri	0.00	0.01	0.00	0.01	0.00	0.01	0.00	0.00	0.28	0.30	0.20	0.17	0.33	0.32	0.32	0.38
Bacteria|Proteobacteria|Alphaproteobacteri	0.00	0.00	0.00	0.00	0.00	0.00	0.00	0.00	0.00	0.00	0.00	0.00	0.00	0.00	0.00	0.00
Bacteria|Proteobacteria|Alphaproteobacteri	0.00	0.00	0.00	0.00	0.00	0.01	0.00	0.00	0.04	0.06	0.03	0.06	0.03	0.05	0.05	0.08
Bacteria|Proteobacteria|Alphaproteobacteri	0.01	0.02	0.01	0.02	0.01	0.01	0.00	0.01	0.46	0.60	0.34	0.46	0.51	0.50	0.54	0.81
Bacteria|Proteobacteria|Alphaproteobacteri	0.00	0.00	0.00	0.00	0.00	0.00	0.00	0.00	0.01	0.02	0.01	0.02	0.01	0.01	0.01	0.02
Bacteria|Proteobacteria|Alphaproteobacteri	0.00	0.02	0.00	0.00	0.01	0.03	0.00	0.00	0.01	0.02	0.01	0.01	0.01	0.01	0.01	0.04
Bacteria|Proteobacteria|Alphaproteobacteri	0.00	0.00	0.00	0.00	0.00	0.00	0.00	0.00	0.01	0.02	0.01	0.03	0.00	0.01	0.01	0.03
Bacteria|Proteobacteria|Alphaproteobacteri	0.00	0.00	0.00	0.00	0.00	0.00	0.00	0.00	0.00	0.00	0.00	0.00	0.00	0.00	0.00	0.00

Microbial clades	Oral Cavity								Esophagus							
	All sites		Sa		TD		SP		All sites		UE		ME		LE	
	Ave	SD	Ave	SD	Ave	SD	Ave	SD	Ave	SD	Ave	SD	Ave	SD	Ave	SD
Bacteria|Proteobacteria|Alphaproteobacteri	0.00	0.00	0.00	0.00	0.00	0.00	0.00	0.00	0.01	0.02	0.01	0.04	0.00	0.01	0.00	0.00
Bacteria|Proteobacteria|Alphaproteobacteri	0.00	0.00	0.00	0.00	0.00	0.00	0.00	0.00	0.00	0.00	0.00	0.00	0.00	0.00	0.00	0.00
Bacteria|Proteobacteria|Alphaproteobacteri	0.00	0.00	0.00	0.00	0.00	0.00	0.00	0.00	0.00	0.00	0.00	0.00	0.00	0.00	0.00	0.00
Bacteria|Proteobacteria|Alphaproteobacteri	0.00	0.00	0.00	0.00	0.00	0.00	0.00	0.01	0.28	0.43	0.29	0.52	0.29	0.45	0.25	0.33
Bacteria|Proteobacteria|Alphaproteobacteri	0.00	0.00	0.00	0.00	0.00	0.00	0.00	0.00	0.18	0.48	0.33	0.80	0.10	0.17	0.13	0.20
Bacteria|Proteobacteria|Alphaproteobacteri	0.00	0.00	0.00	0.00	0.00	0.00	0.00	0.00	0.00	0.00	0.00	0.00	0.00	0.00	0.00	0.00
Bacteria|Proteobacteria|Alphaproteobacteri	0.00	0.00	0.00	0.00	0.00	0.00	0.00	0.00	0.00	0.00	0.00	0.00	0.00	0.00	0.00	0.00
Bacteria|Proteobacteria|Alphaproteobacteri	0.00	0.00	0.00	0.00	0.00	0.00	0.00	0.00	0.00	0.02	0.01	0.04	0.00	0.00	0.00	0.00
Bacteria|Proteobacteria|Alphaproteobacteri	0.00	0.00	0.00	0.00	0.00	0.00	0.00	0.00	0.00	0.00	0.00	0.00	0.00	0.00	0.00	0.00
Bacteria|Proteobacteria|Alphaproteobacteri	0.00	0.00	0.00	0.00	0.00	0.00	0.00	0.00	0.00	0.00	0.00	0.00	0.00	0.00	0.00	0.00
Bacteria|Proteobacteria|Alphaproteobacteri	0.00	0.02	0.00	0.01	0.01	0.02	0.00	0.01	0.05	0.12	0.08	0.14	0.05	0.14	0.02	0.02
Bacteria|Proteobacteria|Alphaproteobacteri	0.00	0.00	0.00	0.00	0.00	0.00	0.00	0.00	0.00	0.01	0.00	0.00	0.00	0.00	0.00	0.02
Bacteria|Proteobacteria|Alphaproteobacteri	0.00	0.00	0.00	0.00	0.00	0.00	0.00	0.00	0.03	0.14	0.01	0.03	0.00	0.01	0.09	0.25
Bacteria|Proteobacteria|Alphaproteobacteri	0.00	0.00	0.00	0.00	0.00	0.00	0.00	0.00	0.00	0.02	0.00	0.00	0.01	0.03	0.00	0.00
Bacteria|Proteobacteria|Alphaproteobacteri	0.00	0.00	0.00	0.00	0.00	0.01	0.00	0.00	0.00	0.01	0.00	0.01	0.00	0.00	0.00	0.00
Bacteria|Proteobacteria|Alphaproteobacteri	0.00	0.01	0.00	0.00	0.01	0.02	0.00	0.00	0.01	0.06	0.00	0.01	0.01	0.02	0.03	0.10
Bacteria|Proteobacteria|Alphaproteobacteri	0.00	0.00	0.00	0.00	0.00	0.00	0.00	0.00	0.00	0.00	0.00	0.00	0.00	0.00	0.00	0.00
Bacteria|Proteobacteria|Alphaproteobacteri	0.00	0.00	0.00	0.00	0.00	0.00	0.00	0.00	0.00	0.02	0.00	0.02	0.00	0.00	0.01	0.04
Bacteria|Proteobacteria|Alphaproteobacteri	0.00	0.00	0.00	0.00	0.00	0.00	0.00	0.00	0.00	0.00	0.00	0.00	0.00	0.00	0.00	0.00
Bacteria|Proteobacteria|Alphaproteobacteri	0.00	0.00	0.00	0.00	0.00	0.00	0.00	0.00	0.00	0.00	0.00	0.01	0.00	0.00	0.00	0.00
Bacteria|Proteobacteria|Alphaproteobacteri	0.00	0.00	0.00	0.00	0.00	0.00	0.00	0.00	0.00	0.00	0.00	0.00	0.00	0.00	0.00	0.00
Bacteria|Proteobacteria|Alphaproteobacteri	0.00	0.00	0.00	0.00	0.00	0.00	0.00	0.00	0.00	0.00	0.00	0.01	0.00	0.00	0.00	0.00
Bacteria|Proteobacteria|Alphaproteobacteri	0.00	0.00	0.00	0.00	0.00	0.00	0.00	0.00	0.02	0.06	0.04	0.10	0.01	0.03	0.01	0.02
Bacteria|Proteobacteria|Alphaproteobacteri	0.00	0.01	0.00	0.00	0.00	0.01	0.00	0.00	0.00	0.01	0.01	0.02	0.00	0.01	0.00	0.00
Bacteria|Proteobacteria|Alphaproteobacteri	0.00	0.00	0.00	0.00	0.00	0.00	0.00	0.00	0.01	0.05	0.02	0.06	0.01	0.07	0.00	0.01
Bacteria|Proteobacteria|Alphaproteobacteri	0.00	0.00	0.00	0.00	0.00	0.00	0.00	0.00	0.00	0.00	0.00	0.00	0.00	0.00	0.00	0.00
Bacteria|Proteobacteria|Alphaproteobacteri	0.00	0.00	0.00	0.00	0.00	0.00	0.00	0.00	0.02	0.06	0.04	0.08	0.03	0.07	0.01	0.02
Bacteria|Proteobacteria|Alphaproteobacteri	0.01	0.09	0.02	0.09	0.03	0.14	0.00	0.00	0.01	0.06	0.01	0.04	0.02	0.09	0.00	0.01
Bacteria|Proteobacteria|Alphaproteobacteri	0.00	0.00	0.00	0.00	0.00	0.00	0.00	0.00	0.00	0.01	0.00	0.00	0.00	0.02	0.00	0.00

Microbial clades	Oral Cavity								Esophagus							
	All sites		Sa		TD		SP		All sites		UE		ME		LE	
	Ave	SD	Ave	SD	Ave	SD	Ave	SD	Ave	SD	Ave	SD	Ave	SD	Ave	SD
Bacteria|Proteobacteria|Alphaproteobacteri	0.00	0.00	0.00	0.00	0.00	0.00	0.00	0.00	0.00	0.01	0.00	0.02	0.00	0.00	0.00	0.00
Bacteria|Proteobacteria|Alphaproteobacteri	0.00	0.00	0.00	0.00	0.00	0.00	0.00	0.00	0.02	0.10	0.03	0.17	0.00	0.00	0.02	0.08
Bacteria|Proteobacteria|Alphaproteobacteri	0.00	0.01	0.00	0.00	0.00	0.02	0.00	0.00	0.01	0.06	0.01	0.02	0.02	0.10	0.00	0.01
Bacteria|Proteobacteria|Alphaproteobacteri	0.00	0.00	0.00	0.00	0.00	0.01	0.00	0.00	0.05	0.10	0.07	0.14	0.05	0.10	0.03	0.05
Bacteria|Proteobacteria|Alphaproteobacteri	0.00	0.00	0.00	0.00	0.00	0.00	0.00	0.00	0.00	0.00	0.00	0.00	0.00	0.00	0.00	0.00
Bacteria|Proteobacteria|Alphaproteobacteri	0.00	0.00	0.00	0.00	0.00	0.00	0.00	0.00	0.00	0.01	0.00	0.00	0.00	0.01	0.00	0.01
Bacteria|Proteobacteria|Alphaproteobacteri	0.00	0.00	0.00	0.00	0.00	0.00	0.00	0.00	0.00	0.00	0.00	0.00	0.00	0.00	0.00	0.00
Bacteria|Proteobacteria|Alphaproteobacteri	0.00	0.00	0.00	0.00	0.00	0.00	0.00	0.00	0.02	0.16	0.06	0.29	0.00	0.00	0.00	0.00
Bacteria|Proteobacteria|Alphaproteobacteri	0.00	0.00	0.00	0.00	0.00	0.00	0.00	0.00	0.08	0.13	0.06	0.07	0.09	0.16	0.09	0.15
Bacteria|Proteobacteria|Alphaproteobacteri	0.00	0.00	0.00	0.00	0.00	0.00	0.00	0.00	0.00	0.01	0.00	0.00	0.00	0.00	0.00	0.01
Bacteria|Proteobacteria|Alphaproteobacteri	0.00	0.00	0.00	0.00	0.00	0.00	0.00	0.00	0.00	0.00	0.00	0.00	0.00	0.00	0.00	0.00
Bacteria|Proteobacteria|Alphaproteobacteri	0.00	0.00	0.00	0.00	0.00	0.00	0.00	0.00	0.00	0.00	0.00	0.00	0.00	0.00	0.00	0.00
Bacteria|Proteobacteria|Alphaproteobacteri	0.00	0.00	0.00	0.00	0.00	0.00	0.00	0.00	0.00	0.00	0.00	0.00	0.00	0.00	0.00	0.00
Bacteria|Proteobacteria|Alphaproteobacteri	0.00	0.00	0.00	0.00	0.00	0.00	0.00	0.00	0.01	0.02	0.02	0.03	0.01	0.02	0.01	0.02
Bacteria|Proteobacteria|Alphaproteobacteri	0.00	0.00	0.00	0.00	0.00	0.00	0.00	0.00	0.01	0.03	0.00	0.01	0.00	0.01	0.02	0.06
Bacteria|Proteobacteria|Alphaproteobacteri	0.00	0.01	0.00	0.00	0.00	0.01	0.00	0.00	0.02	0.10	0.05	0.17	0.00	0.00	0.00	0.00
Bacteria|Proteobacteria|Alphaproteobacteri	0.01	0.01	0.01	0.02	0.00	0.01	0.00	0.01	1.11	4.27	2.07	6.75	0.95	3.18	0.34	0.54
Bacteria|Proteobacteria|Alphaproteobacteri	0.00	0.01	0.00	0.01	0.00	0.01	0.00	0.01	0.82	3.42	1.72	5.78	0.43	1.46	0.36	0.78
Bacteria|Proteobacteria|Alphaproteobacteri	0.21	0.71	0.16	0.55	0.30	0.97	0.18	0.57	7.98	17.03	7.28	15.84	7.99	17.29	8.68	18.56
Bacteria|Proteobacteria|Alphaproteobacteri	0.00	0.00	0.00	0.00	0.00	0.00	0.00	0.00	0.01	0.02	0.00	0.01	0.00	0.01	0.01	0.03
Bacteria|Proteobacteria|Alphaproteobacteri	0.00	0.00	0.00	0.00	0.00	0.00	0.00	0.00	0.02	0.06	0.03	0.06	0.02	0.07	0.02	0.04
Bacteria|Proteobacteria|Alphaproteobacteri	0.00	0.00	0.00	0.00	0.00	0.00	0.00	0.00	0.02	0.06	0.03	0.06	0.03	0.07	0.01	0.03
Bacteria|Proteobacteria|Alphaproteobacteri	0.00	0.00	0.00	0.00	0.00	0.00	0.00	0.00	0.00	0.00	0.00	0.00	0.00	0.00	0.00	0.00
Bacteria|Proteobacteria|Alphaproteobacteri	0.00	0.00	0.00	0.00	0.00	0.00	0.00	0.00	0.00	0.00	0.00	0.00	0.00	0.00	0.00	0.00
Bacteria|Proteobacteria|Betaproteobacteria	0.00	0.00	0.00	0.00	0.00	0.00	0.00	0.00	0.01	0.02	0.01	0.02	0.00	0.01	0.01	0.02
Bacteria|Proteobacteria|Betaproteobacteria	0.00	0.02	0.01	0.03	0.00	0.01	0.00	0.01	0.18	0.71	0.04	0.11	0.10	0.45	0.40	1.15
Bacteria|Proteobacteria|Betaproteobacteria	0.00	0.00	0.00	0.00	0.00	0.00	0.00	0.00	0.00	0.00	0.00	0.00	0.00	0.00	0.00	0.00
Bacteria|Proteobacteria|Betaproteobacteria	0.00	0.00	0.00	0.00	0.00	0.00	0.00	0.00	0.02	0.03	0.02	0.04	0.02	0.03	0.01	0.02
Bacteria|Proteobacteria|Betaproteobacteria	0.00	0.00	0.00	0.00	0.00	0.00	0.00	0.00	0.00	0.00	0.00	0.00	0.00	0.01	0.00	0.00

Microbial clades	Oral Cavity								Esophagus							
	All sites		Sa		TD		SP		All sites		UE		ME		LE	
	Ave	SD	Ave	SD	Ave	SD	Ave	SD	Ave	SD	Ave	SD	Ave	SD	Ave	SD
Bacteria|Proteobacteria|Betaproteobacteria	1.27	1.80	1.97	1.75	0.11	0.11	1.75	2.21	0.15	0.15	0.13	0.15	0.16	0.15	0.16	0.15
Bacteria|Proteobacteria|Betaproteobacteria	0.00	0.00	0.00	0.00	0.00	0.00	0.00	0.00	0.00	0.00	0.00	0.00	0.00	0.00	0.00	0.00
Bacteria|Proteobacteria|Betaproteobacteria	0.00	0.00	0.00	0.00	0.00	0.00	0.00	0.00	0.00	0.02	0.00	0.01	0.00	0.01	0.01	0.02
Bacteria|Proteobacteria|Betaproteobacteria	0.00	0.00	0.00	0.00	0.00	0.01	0.00	0.00	0.03	0.06	0.04	0.10	0.02	0.02	0.02	0.04
Bacteria|Proteobacteria|Betaproteobacteria	0.00	0.00	0.00	0.00	0.00	0.00	0.00	0.00	0.26	1.99	0.02	0.03	0.03	0.05	0.78	3.52
Bacteria|Proteobacteria|Betaproteobacteria	0.00	0.00	0.00	0.00	0.00	0.00	0.00	0.00	0.00	0.00	0.00	0.00	0.00	0.00	0.00	0.00
Bacteria|Proteobacteria|Betaproteobacteria	0.00	0.00	0.00	0.00	0.00	0.00	0.00	0.00	0.01	0.02	0.01	0.02	0.01	0.02	0.01	0.02
Bacteria|Proteobacteria|Betaproteobacteria	0.00	0.00	0.00	0.00	0.00	0.00	0.00	0.00	0.00	0.00	0.00	0.00	0.00	0.00	0.00	0.00
Bacteria|Proteobacteria|Betaproteobacteria	0.00	0.00	0.00	0.00	0.00	0.00	0.00	0.00	0.00	0.00	0.00	0.00	0.00	0.00	0.00	0.00
Bacteria|Proteobacteria|Betaproteobacteria	0.00	0.00	0.00	0.00	0.00	0.00	0.00	0.00	0.00	0.03	0.00	0.00	0.01	0.04	0.00	0.01
Bacteria|Proteobacteria|Betaproteobacteria	0.00	0.00	0.00	0.00	0.00	0.00	0.00	0.00	0.00	0.00	0.00	0.00	0.00	0.00	0.00	0.00
Bacteria|Proteobacteria|Betaproteobacteria	0.00	0.01	0.00	0.00	0.00	0.01	0.00	0.00	0.01	0.02	0.01	0.04	0.00	0.01	0.00	0.01
Bacteria|Proteobacteria|Betaproteobacteria	0.00	0.00	0.00	0.00	0.00	0.00	0.00	0.00	0.01	0.02	0.02	0.03	0.01	0.02	0.00	0.01
Bacteria|Proteobacteria|Betaproteobacteria	0.00	0.00	0.00	0.00	0.00	0.00	0.00	0.00	0.00	0.00	0.00	0.00	0.00	0.00	0.00	0.00
Bacteria|Proteobacteria|Betaproteobacteria	0.00	0.00	0.00	0.00	0.00	0.00	0.00	0.00	0.00	0.01	0.00	0.00	0.00	0.00	0.00	0.01
Bacteria|Proteobacteria|Betaproteobacteria	0.00	0.00	0.00	0.00	0.00	0.00	0.00	0.00	0.00	0.00	0.00	0.00	0.00	0.00	0.00	0.00
Bacteria|Proteobacteria|Betaproteobacteria	0.00	0.00	0.00	0.00	0.00	0.00	0.00	0.00	0.00	0.00	0.00	0.00	0.00	0.00	0.00	0.00
Bacteria|Proteobacteria|Betaproteobacteria	0.00	0.00	0.00	0.00	0.00	0.01	0.00	0.00	0.00	0.01	0.00	0.01	0.01	0.02	0.00	0.00
Bacteria|Proteobacteria|Betaproteobacteria	0.20	0.64	0.22	0.68	0.01	0.02	0.38	0.86	0.09	0.32	0.18	0.53	0.05	0.14	0.05	0.10
Bacteria|Proteobacteria|Betaproteobacteria	1.63	2.53	1.43	2.11	0.06	0.09	3.46	3.08	0.25	0.32	0.34	0.47	0.22	0.23	0.18	0.21
Bacteria|Proteobacteria|Betaproteobacteria	0.00	0.00	0.00	0.00	0.00	0.00	0.00	0.00	0.10	0.24	0.21	0.38	0.05	0.10	0.06	0.10
Bacteria|Proteobacteria|Betaproteobacteria	0.00	0.00	0.00	0.00	0.00	0.01	0.00	0.00	0.00	0.01	0.01	0.02	0.00	0.01	0.00	0.00
Bacteria|Proteobacteria|Betaproteobacteria	0.00	0.00	0.00	0.00	0.00	0.00	0.00	0.00	0.13	0.28	0.16	0.36	0.10	0.19	0.13	0.29
Bacteria|Proteobacteria|Betaproteobacteria	0.00	0.00	0.00	0.00	0.00	0.00	0.00	0.00	0.07	0.12	0.07	0.11	0.08	0.17	0.06	0.06
Bacteria|Proteobacteria|Betaproteobacteria	0.00	0.00	0.00	0.00	0.00	0.01	0.00	0.00	0.22	0.53	0.30	0.61	0.15	0.46	0.23	0.54
Bacteria|Proteobacteria|Betaproteobacteria	0.00	0.00	0.00	0.00	0.00	0.00	0.00	0.00	0.00	0.01	0.01	0.02	0.00	0.00	0.00	0.00
Bacteria|Proteobacteria|Betaproteobacteria	0.00	0.00	0.00	0.00	0.00	0.00	0.00	0.00	0.01	0.05	0.00	0.01	0.02	0.08	0.00	0.00
Bacteria|Proteobacteria|Betaproteobacteria	0.00	0.00	0.00	0.00	0.00	0.00	0.00	0.00	0.00	0.00	0.00	0.00	0.00	0.00	0.00	0.00
Bacteria|Proteobacteria|Betaproteobacteria	0.00	0.00	0.00	0.00	0.00	0.00	0.00	0.00	0.00	0.02	0.00	0.00	0.00	0.02	0.01	0.02

Microbial clades	Oral Cavity								Esophagus							
	All sites		Sa		TD		SP		All sites		UE		ME		LE	
	Ave	SD	Ave	SD	Ave	SD	Ave	SD	Ave	SD	Ave	SD	Ave	SD	Ave	SD
Bacteria|Proteobacteria|Betaproteobacteria	0.60	0.65	0.66	0.41	0.08	0.11	1.09	0.78	0.05	0.08	0.04	0.05	0.04	0.05	0.07	0.13
Bacteria|Proteobacteria|Betaproteobacteria	0.07	0.11	0.10	0.08	0.03	0.03	0.09	0.17	0.04	0.12	0.01	0.02	0.03	0.08	0.07	0.20
Bacteria|Proteobacteria|Betaproteobacteria	21.93	15.05	28.35	11.60	27.34	16.49	9.66	7.73	4.78	4.14	4.10	2.86	4.70	2.88	5.56	6.06
Bacteria|Proteobacteria|Betaproteobacteria	0.11	0.13	0.14	0.13	0.06	0.08	0.13	0.15	0.01	0.03	0.02	0.03	0.01	0.02	0.02	0.03
Bacteria|Proteobacteria|Betaproteobacteria	2.83	3.06	2.72	1.80	0.37	0.34	5.51	3.48	0.26	0.32	0.22	0.22	0.26	0.25	0.29	0.45
Bacteria|Proteobacteria|Betaproteobacteria	0.00	0.00	0.00	0.00	0.00	0.00	0.00	0.00	0.00	0.00	0.00	0.00	0.00	0.00	0.00	0.00
Bacteria|Proteobacteria|Betaproteobacteria	0.00	0.00	0.00	0.00	0.00	0.00	0.00	0.00	0.00	0.00	0.00	0.00	0.00	0.00	0.00	0.00
Bacteria|Proteobacteria|Betaproteobacteria	0.00	0.00	0.00	0.00	0.00	0.00	0.00	0.00	0.01	0.04	0.01	0.01	0.01	0.02	0.03	0.06
Bacteria|Proteobacteria|Betaproteobacteria	0.01	0.03	0.02	0.04	0.00	0.00	0.02	0.04	0.00	0.00	0.00	0.00	0.00	0.00	0.00	0.00
Bacteria|Proteobacteria|Betaproteobacteria	0.00	0.00	0.00	0.00	0.00	0.00	0.00	0.00	0.00	0.01	0.00	0.00	0.00	0.00	0.00	0.01
Bacteria|Proteobacteria|Betaproteobacteria	0.00	0.00	0.00	0.00	0.00	0.00	0.00	0.00	0.01	0.03	0.01	0.02	0.01	0.03	0.02	0.05
Bacteria|Proteobacteria|Betaproteobacteria	0.00	0.01	0.00	0.00	0.00	0.01	0.00	0.00	0.00	0.01	0.00	0.02	0.00	0.01	0.00	0.01
Bacteria|Proteobacteria|Deltaproteobacteria	0.00	0.00	0.00	0.00	0.00	0.00	0.00	0.00	0.00	0.01	0.00	0.01	0.00	0.00	0.00	0.00
Bacteria|Proteobacteria|Deltaproteobacteria	0.00	0.00	0.00	0.00	0.00	0.00	0.00	0.00	0.00	0.02	0.01	0.04	0.00	0.00	0.00	0.00
Bacteria|Proteobacteria|Deltaproteobacteria	0.03	0.08	0.04	0.04	0.00	0.01	0.06	0.12	0.00	0.02	0.00	0.00	0.01	0.01	0.01	0.03
Bacteria|Proteobacteria|Deltaproteobacteria	0.01	0.03	0.01	0.03	0.00	0.00	0.01	0.05	0.00	0.02	0.00	0.00	0.00	0.03	0.00	0.00
Bacteria|Proteobacteria|Deltaproteobacteria	0.00	0.00	0.00	0.00	0.00	0.00	0.00	0.00	0.00	0.00	0.00	0.00	0.00	0.00	0.00	0.01
Bacteria|Proteobacteria|Deltaproteobacteria	0.06	0.17	0.11	0.24	0.00	0.01	0.06	0.14	0.02	0.05	0.02	0.05	0.01	0.01	0.03	0.07
Bacteria|Proteobacteria|Deltaproteobacteria	0.00	0.00	0.00	0.00	0.00	0.00	0.00	0.00	0.03	0.08	0.03	0.11	0.01	0.05	0.03	0.09
Bacteria|Proteobacteria|Deltaproteobacteria	0.00	0.00	0.00	0.00	0.00	0.00	0.00	0.00	0.00	0.01	0.00	0.00	0.00	0.00	0.00	0.01
Bacteria|Proteobacteria|Deltaproteobacteria	0.00	0.00	0.00	0.00	0.00	0.00	0.00	0.00	0.00	0.04	0.00	0.00	0.01	0.06	0.00	0.00
Bacteria|Proteobacteria|Deltaproteobacteria	0.00	0.00	0.00	0.00	0.00	0.00	0.00	0.00	0.00	0.00	0.00	0.00	0.00	0.00	0.00	0.00
Bacteria|Proteobacteria|Deltaproteobacteria	0.00	0.00	0.00	0.00	0.00	0.00	0.00	0.00	0.00	0.00	0.00	0.00	0.00	0.00	0.00	0.00
Bacteria|Proteobacteria|Deltaproteobacteria	0.00	0.00	0.00	0.00	0.00	0.00	0.00	0.00	0.00	0.00	0.00	0.00	0.00	0.00	0.00	0.00
Bacteria|Proteobacteria|Epsilonproteobacte	0.00	0.00	0.00	0.00	0.00	0.00	0.00	0.00	0.00	0.00	0.00	0.00	0.00	0.00	0.00	0.00
Bacteria|Proteobacteria|Epsilonproteobacte	1.19	1.23	0.13	0.07	1.44	1.31	2.02	1.00	0.53	0.74	0.76	1.08	0.47	0.51	0.37	0.46
Bacteria|Proteobacteria|Epsilonproteobacte	0.01	0.06	0.01	0.02	0.00	0.00	0.03	0.11	0.00	0.01	0.00	0.01	0.01	0.02	0.00	0.00
Bacteria|Proteobacteria|Epsilonproteobacte	0.00	0.00	0.00	0.00	0.00	0.00	0.00	0.01	1.36	4.75	0.90	3.45	0.33	0.68	2.99	7.48
Bacteria|Proteobacteria|Epsilonproteobacte	0.00	0.00	0.00	0.00	0.00	0.00	0.00	0.00	0.00	0.00	0.00	0.00	0.00	0.00	0.00	0.00

Microbial clades	Oral Cavity								Esophagus							
	All sites		Sa		TD		SP		All sites		UE		ME		LE	
	Ave	SD	Ave	SD	Ave	SD	Ave	SD	Ave	SD	Ave	SD	Ave	SD	Ave	SD
Bacteria|Proteobacteria|Gammaproteobacte	0.00	0.00	0.00	0.00	0.00	0.00	0.00	0.00	0.00	0.01	0.00	0.01	0.00	0.00	0.00	0.00
Bacteria|Proteobacteria|Gammaproteobacte	0.00	0.00	0.00	0.00	0.00	0.00	0.00	0.00	0.00	0.00	0.00	0.00	0.00	0.00	0.00	0.00
Bacteria|Proteobacteria|Gammaproteobacte	0.00	0.00	0.00	0.00	0.00	0.00	0.00	0.00	0.01	0.04	0.01	0.05	0.01	0.02	0.01	0.04
Bacteria|Proteobacteria|Gammaproteobacte	0.01	0.03	0.01	0.04	0.00	0.01	0.00	0.01	0.01	0.02	0.00	0.01	0.00	0.01	0.01	0.02
Bacteria|Proteobacteria|Gammaproteobacte	0.00	0.00	0.00	0.00	0.00	0.00	0.00	0.00	0.00	0.00	0.00	0.00	0.00	0.00	0.00	0.00
Bacteria|Proteobacteria|Gammaproteobacte	0.00	0.00	0.00	0.00	0.00	0.00	0.00	0.00	0.00	0.00	0.00	0.00	0.00	0.00	0.00	0.00
Bacteria|Proteobacteria|Gammaproteobacte	0.00	0.00	0.00	0.00	0.00	0.00	0.00	0.00	0.00	0.00	0.00	0.00	0.00	0.00	0.00	0.01
Bacteria|Proteobacteria|Gammaproteobacte	0.00	0.00	0.00	0.00	0.00	0.00	0.00	0.00	0.00	0.00	0.00	0.00	0.00	0.00	0.00	0.00
Bacteria|Proteobacteria|Gammaproteobacte	0.00	0.00	0.00	0.00	0.00	0.00	0.00	0.00	0.01	0.05	0.00	0.00	0.02	0.08	0.01	0.04
Bacteria|Proteobacteria|Gammaproteobacte	0.00	0.00	0.00	0.00	0.00	0.00	0.00	0.00	0.00	0.00	0.00	0.00	0.00	0.00	0.00	0.00
Bacteria|Proteobacteria|Gammaproteobacte	0.00	0.00	0.00	0.00	0.00	0.00	0.00	0.00	0.00	0.02	0.00	0.00	0.00	0.00	0.01	0.04
Bacteria|Proteobacteria|Gammaproteobacte	0.52	0.83	0.47	0.42	0.02	0.02	1.08	1.18	0.02	0.06	0.02	0.04	0.01	0.02	0.03	0.09
Bacteria|Proteobacteria|Gammaproteobacte	0.07	0.19	0.08	0.14	0.01	0.02	0.14	0.30	0.00	0.01	0.00	0.00	0.00	0.00	0.01	0.02
Bacteria|Proteobacteria|Gammaproteobacte	0.02	0.09	0.01	0.01	0.00	0.01	0.06	0.16	0.00	0.01	0.00	0.00	0.00	0.00	0.00	0.01
Bacteria|Proteobacteria|Gammaproteobacte	0.00	0.00	0.00	0.00	0.00	0.00	0.00	0.00	0.00	0.00	0.00	0.01	0.00	0.00	0.00	0.00
Bacteria|Proteobacteria|Gammaproteobacte	0.00	0.00	0.00	0.00	0.00	0.00	0.00	0.00	0.01	0.02	0.01	0.01	0.01	0.03	0.01	0.02
Bacteria|Proteobacteria|Gammaproteobacte	0.00	0.00	0.00	0.00	0.00	0.00	0.00	0.00	0.01	0.01	0.00	0.01	0.01	0.01	0.01	0.01
Bacteria|Proteobacteria|Gammaproteobacte	0.00	0.00	0.00	0.00	0.00	0.00	0.00	0.00	0.00	0.00	0.00	0.00	0.00	0.00	0.00	0.00
Bacteria|Proteobacteria|Gammaproteobacte	0.00	0.00	0.00	0.00	0.00	0.00	0.00	0.00	0.00	0.00	0.00	0.00	0.00	0.00	0.00	0.00
Bacteria|Proteobacteria|Gammaproteobacte	0.00	0.00	0.00	0.00	0.00	0.00	0.00	0.00	0.00	0.00	0.00	0.00	0.00	0.00	0.00	0.00
Bacteria|Proteobacteria|Gammaproteobacte	0.00	0.00	0.00	0.00	0.00	0.00	0.00	0.00	0.01	0.05	0.00	0.00	0.02	0.08	0.00	0.02
Bacteria|Proteobacteria|Gammaproteobacte	0.00	0.00	0.00	0.00	0.00	0.00	0.00	0.00	0.00	0.00	0.00	0.00	0.00	0.00	0.00	0.00
Bacteria|Proteobacteria|Gammaproteobacte	0.00	0.00	0.00	0.01	0.00	0.00	0.00	0.00	0.00	0.01	0.00	0.00	0.00	0.00	0.00	0.02
Bacteria|Proteobacteria|Gammaproteobacte	0.00	0.02	0.00	0.01	0.01	0.03	0.00	0.00	0.03	0.06	0.01	0.01	0.02	0.03	0.05	0.09
Bacteria|Proteobacteria|Gammaproteobacte	0.11	0.35	0.10	0.21	0.16	0.55	0.07	0.12	4.52	7.24	2.60	3.14	5.40	9.16	5.46	7.62
Bacteria|Proteobacteria|Gammaproteobacte	0.00	0.00	0.00	0.00	0.00	0.00	0.00	0.00	0.00	0.00	0.00	0.00	0.00	0.00	0.00	0.00
Bacteria|Proteobacteria|Gammaproteobacte	0.00	0.00	0.00	0.00	0.00	0.00	0.00	0.00	0.02	0.04	0.00	0.01	0.02	0.05	0.02	0.05
Bacteria|Proteobacteria|Gammaproteobacte	0.00	0.00	0.00	0.00	0.00	0.00	0.00	0.00	0.00	0.00	0.00	0.00	0.00	0.00	0.00	0.00
Bacteria|Proteobacteria|Gammaproteobacte	0.00	0.00	0.00	0.00	0.00	0.00	0.00	0.00	0.00	0.00	0.00	0.00	0.00	0.00	0.00	0.00

Microbial clades	Oral Cavity								Esophagus							
	All sites		Sa		TD		SP		All sites		UE		ME		LE	
	Ave	SD	Ave	SD	Ave	SD	Ave	SD	Ave	SD	Ave	SD	Ave	SD	Ave	SD
Bacteria|Proteobacteria|Gammaproteobacte	0.00	0.00	0.00	0.00	0.00	0.00	0.00	0.00	0.02	0.12	0.00	0.01	0.04	0.19	0.02	0.09
Bacteria|Proteobacteria|Gammaproteobacte	0.00	0.00	0.00	0.00	0.00	0.00	0.00	0.00	0.00	0.00	0.00	0.00	0.00	0.00	0.00	0.00
Bacteria|Proteobacteria|Gammaproteobacte	0.14	0.40	0.16	0.25	0.08	0.17	0.18	0.63	9.49	15.41	12.29	21.13	9.75	13.77	6.39	9.26
Bacteria|Proteobacteria|Gammaproteobacte	1.09	1.88	0.67	0.83	0.22	0.20	2.44	2.73	0.28	0.35	0.28	0.31	0.22	0.24	0.35	0.46
Bacteria|Proteobacteria|Gammaproteobacte	2.07	2.49	1.70	1.57	3.76	3.25	0.70	0.90	3.99	4.20	4.40	5.30	4.09	4.03	3.47	3.14
Bacteria|Proteobacteria|Gammaproteobacte	0.00	0.00	0.00	0.00	0.00	0.00	0.00	0.00	0.00	0.00	0.00	0.00	0.00	0.00	0.00	0.00
Bacteria|Proteobacteria|Gammaproteobacte	0.00	0.00	0.00	0.00	0.00	0.00	0.00	0.00	0.01	0.05	0.01	0.05	0.01	0.04	0.01	0.06
Bacteria|Proteobacteria|Gammaproteobacte	0.01	0.02	0.01	0.02	0.01	0.01	0.00	0.01	0.01	0.01	0.00	0.01	0.01	0.02	0.00	0.01
Bacteria|Proteobacteria|Gammaproteobacte	0.00	0.00	0.00	0.00	0.00	0.00	0.00	0.00	0.00	0.00	0.00	0.00	0.00	0.00	0.00	0.00
Bacteria|Proteobacteria|Gammaproteobacte	0.00	0.01	0.00	0.01	0.00	0.01	0.00	0.00	0.44	1.36	0.21	0.40	0.40	0.64	0.72	2.29
Bacteria|Proteobacteria|Gammaproteobacte	0.00	0.00	0.00	0.00	0.00	0.00	0.00	0.00	0.00	0.01	0.00	0.02	0.00	0.00	0.00	0.00
Bacteria|Proteobacteria|Gammaproteobacte	0.01	0.01	0.01	0.01	0.01	0.02	0.00	0.01	0.14	0.26	0.12	0.23	0.15	0.22	0.16	0.33
Bacteria|Proteobacteria|Gammaproteobacte	0.88	3.45	0.09	0.15	1.52	5.67	1.03	1.76	0.14	0.33	0.12	0.16	0.12	0.22	0.18	0.51
Bacteria|Proteobacteria|Gammaproteobacte	0.00	0.00	0.00	0.00	0.00	0.00	0.00	0.00	0.00	0.01	0.00	0.00	0.00	0.00	0.00	0.01
Bacteria|Proteobacteria|Gammaproteobacte	0.00	0.00	0.00	0.00	0.00	0.00	0.00	0.00	0.00	0.01	0.00	0.01	0.00	0.00	0.00	0.01
Bacteria|Proteobacteria|Gammaproteobacte	0.00	0.00	0.00	0.00	0.00	0.00	0.00	0.00	0.00	0.00	0.00	0.00	0.00	0.00	0.00	0.00
Bacteria|Proteobacteria|Gammaproteobacte	0.00	0.00	0.00	0.00	0.00	0.00	0.00	0.00	0.02	0.07	0.01	0.02	0.01	0.04	0.04	0.12
Bacteria|Proteobacteria|Gammaproteobacte	0.03	0.06	0.02	0.07	0.02	0.04	0.03	0.06	0.80	1.15	0.82	1.09	0.59	0.66	1.01	1.58
Bacteria|Proteobacteria|Gammaproteobacte	0.00	0.00	0.00	0.00	0.00	0.00	0.00	0.00	0.01	0.02	0.01	0.02	0.00	0.01	0.01	0.02
Bacteria|Proteobacteria|Gammaproteobacte	0.00	0.00	0.00	0.00	0.00	0.00	0.00	0.01	0.08	0.31	0.20	0.52	0.03	0.09	0.03	0.04
Bacteria|Proteobacteria|Gammaproteobacte	0.00	0.00	0.00	0.00	0.00	0.00	0.00	0.00	0.00	0.00	0.00	0.00	0.00	0.00	0.00	0.00
Bacteria|Proteobacteria|Gammaproteobacte	0.00	0.00	0.00	0.00	0.00	0.00	0.00	0.00	0.00	0.00	0.00	0.00	0.00	0.00	0.00	0.00
Bacteria|Proteobacteria|Gammaproteobacte	0.00	0.00	0.00	0.00	0.00	0.00	0.00	0.00	0.00	0.00	0.00	0.00	0.00	0.00	0.00	0.00
Bacteria|Proteobacteria|Gammaproteobacte	0.00	0.00	0.00	0.00	0.00	0.00	0.00	0.00	0.00	0.00	0.00	0.00	0.00	0.00	0.00	0.00
Bacteria|Proteobacteria|Gammaproteobacte	0.00	0.00	0.00	0.00	0.00	0.00	0.00	0.00	0.00	0.00	0.00	0.00	0.00	0.00	0.00	0.00
Bacteria|Proteobacteria|Gammaproteobacte	0.00	0.00	0.00	0.00	0.00	0.00	0.00	0.00	0.02	0.04	0.01	0.03	0.01	0.04	0.02	0.04
Bacteria|Proteobacteria|Gammaproteobacte	0.00	0.00	0.00	0.00	0.00	0.00	0.00	0.00	0.01	0.05	0.01	0.02	0.01	0.01	0.03	0.08
Bacteria|Proteobacteria|Gammaproteobacte	0.00	0.00	0.00	0.00	0.00	0.00	0.00	0.00	0.00	0.01	0.00	0.01	0.00	0.00	0.00	0.00
Bacteria|Proteobacteria|Gammaproteobacte	0.00	0.00	0.00	0.00	0.00	0.00	0.00	0.00	0.02	0.08	0.01	0.02	0.03	0.12	0.02	0.06

Microbial clades	Oral Cavity								Esophagus							
	All sites		Sa		TD		SP		All sites		UE		ME		LE	
	Ave	SD	Ave	SD	Ave	SD	Ave	SD	Ave	SD	Ave	SD	Ave	SD	Ave	SD
Bacteria|Proteobacteria|Gammaproteobacte	0.00	0.00	0.00	0.00	0.00	0.00	0.00	0.00	0.00	0.00	0.00	0.00	0.00	0.00	0.00	0.00
Bacteria|Proteobacteria|Gammaproteobacte	0.00	0.00	0.00	0.00	0.00	0.00	0.00	0.00	0.00	0.01	0.00	0.01	0.00	0.01	0.00	0.00
Bacteria|Proteobacteria|Gammaproteobacte	0.00	0.00	0.00	0.00	0.00	0.00	0.00	0.00	0.00	0.01	0.00	0.02	0.00	0.00	0.00	0.00
Bacteria|Proteobacteria|Gammaproteobacte	0.01	0.04	0.01	0.02	0.01	0.01	0.02	0.06	0.69	2.48	1.50	4.32	0.30	0.31	0.30	0.29
Bacteria|Proteobacteria|Gammaproteobacte	0.00	0.00	0.00	0.00	0.00	0.00	0.00	0.00	0.00	0.01	0.00	0.00	0.00	0.00	0.01	0.02
Bacteria|Proteobacteria|Gammaproteobacte	0.00	0.00	0.00	0.00	0.00	0.00	0.00	0.00	0.01	0.04	0.01	0.02	0.01	0.02	0.03	0.06
Bacteria|Proteobacteria|Gammaproteobacte	0.00	0.01	0.00	0.00	0.00	0.00	0.00	0.02	0.19	0.96	0.53	1.66	0.01	0.02	0.04	0.11
Bacteria|Proteobacteria|Gammaproteobacte	0.00	0.00	0.00	0.00	0.00	0.00	0.00	0.00	0.00	0.00	0.00	0.00	0.00	0.00	0.00	0.00
Bacteria|Proteobacteria_Other	0.00	0.00	0.00	0.00	0.00	0.00	0.00	0.00	0.00	0.00	0.00	0.00	0.00	0.00	0.00	0.00
Bacteria|Spirochaetes|Spirochaetes|Sphaer	0.00	0.01	0.00	0.01	0.00	0.00	0.01	0.02	0.00	0.00	0.00	0.00	0.00	0.00	0.00	0.00
Bacteria|Spirochaetes|Spirochaetes|Spiroch	0.70	1.10	0.62	0.45	0.13	0.12	1.37	1.66	0.17	0.19	0.17	0.23	0.18	0.19	0.14	0.15
Bacteria|Synergistetes|Synergistia|Synergis	0.00	0.02	0.01	0.01	0.00	0.00	0.01	0.03	0.00	0.00	0.00	0.00	0.00	0.00	0.00	0.00
Bacteria|Synergistetes|Synergistia|Synergis	0.11	0.21	0.24	0.31	0.00	0.01	0.09	0.12	0.01	0.01	0.01	0.01	0.01	0.01	0.01	0.02
Bacteria|Synergistetes|Synergistia|Synergis	0.00	0.00	0.00	0.00	0.00	0.00	0.00	0.00	0.00	0.00	0.00	0.00	0.00	0.00	0.00	0.00
Bacteria|Synergistetes|Synergistia|Synergis	0.00	0.00	0.00	0.00	0.00	0.00	0.00	0.00	0.00	0.00	0.00	0.00	0.00	0.00	0.00	0.00
Bacteria|Tenericutes|Mollicutes|Acholepla	0.05	0.20	0.00	0.01	0.01	0.03	0.13	0.34	0.03	0.13	0.01	0.01	0.05	0.14	0.04	0.17
Bacteria|Tenericutes|Mollicutes|Mycoplasr	0.05	0.10	0.03	0.06	0.03	0.04	0.09	0.16	0.08	0.15	0.09	0.21	0.09	0.13	0.06	0.13
Bacteria|Tenericutes|Mollicutes|Mycoplasr	0.00	0.01	0.00	0.00	0.01	0.01	0.00	0.00	0.00	0.01	0.00	0.01	0.01	0.01	0.00	0.01
Bacteria|Thermotogae|Thermotogae|Therm	0.00	0.00	0.00	0.00	0.00	0.00	0.00	0.00	0.00	0.00	0.00	0.00	0.00	0.00	0.00	0.00
Bacteria|Thermotogae|Thermotogae|Therm	0.00	0.00	0.00	0.00	0.00	0.00	0.00	0.00	0.00	0.01	0.00	0.00	0.00	0.00	0.00	0.02
Bacteria|TM7|TM7-3|CW040|F16_unclass	0.03	0.05	0.01	0.01	0.05	0.06	0.04	0.06	0.03	0.08	0.02	0.03	0.04	0.12	0.02	0.05
Bacteria|TM7|TM7-3|I025|Rs-045_unclass	0.20	0.33	0.35	0.36	0.09	0.26	0.17	0.34	0.18	0.55	0.10	0.32	0.20	0.60	0.23	0.68
Bacteria|Verrucomicrobia|Verruco-5|WCH	0.00	0.00	0.00	0.00	0.00	0.00	0.00	0.00	0.00	0.00	0.00	0.00	0.00	0.00	0.00	0.00
Bacteria|Verrucomicrobia|Verrucomicrobia	0.00	0.00	0.00	0.00	0.00	0.00	0.00	0.00	0.08	0.30	0.07	0.13	0.15	0.47	0.03	0.12

ıbrreviation: Sa, saliva; TD, tongue dorsum; SP, supragingival plaque; UE, upper esophagus; ME, middle esophagus; LE, lower esophagus; Ave, Average; SD, standard deviatio
