# Supplementary material for: Microbial Similarity and Preference for Specific Sites in Healthy Oral Cavity and Esophagus
Source: Front Microbiol. 2018 Jul 17;9:1603. doi: 10.3389/fmicb.2018.01603 (PMC6056649; doi:10.3389/fmicb.2018.01603)
Supplement: Supplementary file 3 [file Table_3.pdf]

Table S3 Percentages of subjects for each taxon detected in oral cavity and esophagus

Taxa	Present in at least one oral cavity and esophagus	Present in at least one oral cavity site	Present in at least one esophagus site
Bacteria|Actinobacteria	100.0	100.0	100.0
Bacteria|Actinobacteria|Actinobacteria	100.0	100.0	100.0
Bacteria|Actinobacteria|Actinobacteria|Actinomycetales	100.0	100.0	100.0
Bacteria|Actinobacteria|Actinobacteria|Actinomycetales|Actinomycetaceae	98.3	100.0	96.7
Bacteria|Actinobacteria|Actinobacteria|Actinomycetales|Actinomycetaceae|Actinomyces	98.3	100.0	96.7
Bacteria|Bacteroidetes	100.0	100.0	100.0
Bacteria|Bacteroidetes|Bacteroidia	100.0	100.0	100.0
Bacteria|Bacteroidetes|Bacteroidia|Bacteroidales	100.0	100.0	100.0
Bacteria|Bacteroidetes|Bacteroidia|Bacteroidales|[Paraprevotellaceae]	100.0	100.0	100.0
Bacteria|Bacteroidetes|Bacteroidia|Bacteroidales|[Paraprevotellaceae]|[Prevotella]	100.0	100.0	100.0
Bacteria|Bacteroidetes|Bacteroidia|Bacteroidales|Porphyromonadaceae	100.0	100.0	100.0
Bacteria|Bacteroidetes|Bacteroidia|Bacteroidales|Porphyromonadaceae|Porphyromonas	99.2	100.0	98.3
Bacteria|Bacteroidetes|Bacteroidia|Bacteroidales|Porphyromonadaceae|Tannerella	83.3	100.0	66.7
Bacteria|Bacteroidetes|Bacteroidia|Bacteroidales|Prevotellaceae	100.0	100.0	100.0
Bacteria|Bacteroidetes|Bacteroidia|Bacteroidales|Prevotellaceae|Prevotella	100.0	100.0	100.0
Bacteria|Bacteroidetes|Flavobacteriia	99.2	100.0	98.3
Bacteria|Bacteroidetes|Flavobacteriia|Flavobacteriales	99.2	100.0	98.3
Bacteria|Bacteroidetes|Flavobacteriia|Flavobacteriales|Flavobacteriaceae	95.8	100.0	91.7
Bacteria|Bacteroidetes|Flavobacteriia|Flavobacteriales|Flavobacteriaceae|Capnocytop	94.2	100.0	88.3
Bacteria|Firmicutes	100.0	100.0	100.0
Bacteria|Firmicutes|Bacilli	100.0	100.0	100.0
Bacteria|Firmicutes|Bacilli|Lactobacillales	100.0	100.0	100.0
Bacteria|Firmicutes|Bacilli|Lactobacillales|Carnobacteriaceae	98.3	100.0	96.7
Bacteria|Firmicutes|Bacilli|Lactobacillales|Carnobacteriaceae|Granulicatella	98.3	100.0	96.7
Bacteria|Firmicutes|Bacilli|Lactobacillales|Streptococcaceae	100.0	100.0	100.0
Bacteria|Firmicutes|Bacilli|Lactobacillales|Streptococcaceae|Streptococcus	100.0	100.0	100.0
Bacteria|Firmicutes|Clostridia	100.0	100.0	100.0
Bacteria|Firmicutes|Clostridia|Clostridiales	100.0	100.0	100.0
Bacteria|Firmicutes|Clostridia|Clostridiales|[Mogibacteriaceae]	95.8	100.0	91.7
Bacteria|Firmicutes|Clostridia|Clostridiales|Lachnospiraceae	97.5	100.0	95.0

Taxa	Present in at least one oral cavity and esophagus	Present in at least one oral cavity site	Present in at least one esophagus site
Bacteria|Firmicutes|Clostridia|Clostridiales|Lachnospiraceae_unclassified	95.8	100.0	91.7
Bacteria|Firmicutes|Clostridia|Clostridiales|Peptostreptococcaceae	92.5	100.0	85.0
Bacteria|Firmicutes|Clostridia|Clostridiales|Veillonellaceae	100.0	100.0	100.0
Bacteria|Firmicutes|Clostridia|Clostridiales|Veillonellaceae|Selenomonas	90.8	100.0	81.7
Bacteria|Firmicutes|Clostridia|Clostridiales|Veillonellaceae|Veillonella	100.0	100.0	100.0
Bacteria|Fusobacteria	99.2	100.0	98.3
Bacteria|Fusobacteria|Fusobacteriia	99.2	100.0	98.3
Bacteria|Fusobacteria|Fusobacteriia|Fusobacteriales	99.2	100.0	98.3
Bacteria|Fusobacteria|Fusobacteriia|Fusobacteriales|Fusobacteriaceae	99.2	100.0	98.3
Bacteria|Fusobacteria|Fusobacteriia|Fusobacteriales|Fusobacteriaceae|Fusobacterium	99.2	100.0	98.3
Bacteria|Fusobacteria|Fusobacteriia|Fusobacteriales|Leptotrichiaceae	95.8	100.0	91.7
Bacteria|Fusobacteria|Fusobacteriia|Fusobacteriales|Leptotrichiaceae|Leptotrichia	95.8	100.0	91.7
Bacteria|Proteobacteria	100.0	100.0	100.0
Bacteria|Proteobacteria|Betaproteobacteria	100.0	100.0	100.0
Bacteria|Proteobacteria|Betaproteobacteria|Burkholderiales	100.0	100.0	100.0
Bacteria|Proteobacteria|Betaproteobacteria|Neisseriales	100.0	100.0	100.0
Bacteria|Proteobacteria|Betaproteobacteria|Neisseriales|Neisseriaceae	100.0	100.0	100.0
Bacteria|Proteobacteria|Betaproteobacteria|Neisseriales|Neisseriaceae|Neisseria	100.0	100.0	100.0
Bacteria|Proteobacteria|Epsilonproteobacteria	95.0	100.0	90.0
Bacteria|Proteobacteria|Epsilonproteobacteria|Campylobacterales	95.0	100.0	90.0
Bacteria|Proteobacteria|Epsilonproteobacteria|Campylobacterales|Campylobacteraceae	94.2	100.0	88.3
Bacteria|Proteobacteria|Epsilonproteobacteria|Campylobacterales|Campylobacteraceae|	94.2	100.0	88.3
Bacteria|Proteobacteria|Gammaproteobacteria	100.0	100.0	100.0
Bacteria|Proteobacteria|Gammaproteobacteria|Pasteurellales	100.0	100.0	100.0
Bacteria|Proteobacteria|Gammaproteobacteria|Pasteurellales|Pasteurellaceae	100.0	100.0	100.0
Bacteria|Proteobacteria|Gammaproteobacteria|Pasteurellales|Pasteurellaceae|Haemophi	100.0	100.0	100.0
Bacteria|TM7	98.3	100.0	96.7
Bacteria|TM7|TM7-3	98.3	100.0	96.7
Bacteria|Actinobacteria|Actinobacteria|Actinomycetales|Micrococcaceae	98.3	98.3	98.3
Bacteria|Actinobacteria|Actinobacteria|Actinomycetales|Micrococcaceae|Rothia	97.5	98.3	96.7
Bacteria|Firmicutes|Bacilli|Gemellales	98.3	98.3	98.3
Bacteria|Firmicutes|Bacilli|Gemellales|Gemellaceae	98.3	98.3	98.3

Taxa	Present in at least one oral cavity and esophagus	Present in at least one oral cavity site	Present in at least one esophagus site
Bacteria|Firmicutes|Clostridia|Clostridiales|[Mogibacteriaceae]_unclassified	92.5	98.3	86.7
Bacteria|Proteobacteria|Betaproteobacteria|Burkholderiales|Burkholderiaceae	88.3	98.3	78.3
Bacteria|Proteobacteria|Betaproteobacteria|Burkholderiales|Burkholderiaceae|Lautrop	86.7	98.3	75.0
Bacteria|Proteobacteria|Betaproteobacteria|Neisseriales|Neisseriaceae_unclassified	90.8	98.3	83.3
Bacteria|Proteobacteria|Betaproteobacteria|Neisseriales|Neisseriaceae|Eikenella	80.0	98.3	61.7
Bacteria|Bacteroidetes|Flavobacteriia|Flavobacteriales|[Weeksellaceae]	94.2	96.7	91.7
Bacteria|Bacteroidetes|Flavobacteriia|Flavobacteriales|[Weeksellaceae]_unclassified	91.7	96.7	86.7
Bacteria|Firmicutes|Bacilli|Gemellales|Gemellaceae_unclassified	97.5	96.7	98.3
Bacteria|Firmicutes|Clostridia|Clostridiales|Veillonellaceae|Dialister	80.8	96.7	65.0
Bacteria|Firmicutes|Erysipelotrichi	90.0	96.7	83.3
Bacteria|Firmicutes|Erysipelotrichi|Erysipelotrichales	90.0	96.7	83.3
Bacteria|Firmicutes|Erysipelotrichi|Erysipelotrichales|Erysipelotrichaceae	90.0	96.7	83.3
Bacteria|Proteobacteria|Gammaproteobacteria|Pasteurellales|Pasteurellaceae|Aggregat	89.2	96.7	81.7
Bacteria|Firmicutes|Clostridia|Clostridiales|Peptostreptococcaceae|Filifactor	78.3	95.0	61.7
Bacteria|Proteobacteria|Betaproteobacteria|Burkholderiales|Comamonadaceae	95.8	95.0	96.7
Bacteria|Proteobacteria|Betaproteobacteria|Burkholderiales|Comamonadaceae_unclassif	94.2	95.0	93.3
Bacteria|Spirochaetes	90.0	95.0	85.0
Bacteria|Spirochaetes|Spirochaetes	90.0	95.0	85.0
Bacteria|Spirochaetes|Spirochaetes|Spirochaetales	90.0	95.0	85.0
Bacteria|Spirochaetes|Spirochaetes|Spirochaetales|Spirochaetaceae	90.0	95.0	85.0
Bacteria|Spirochaetes|Spirochaetes|Spirochaetales|Spirochaetaceae|Treponema	90.0	95.0	85.0
Bacteria|Actinobacteria|Actinobacteria|Actinomycetales|Corynebacteriaceae	85.8	93.3	78.3
Bacteria|Actinobacteria|Actinobacteria|Actinomycetales|Corynebacteriaceae|Corynebac	85.8	93.3	78.3
Bacteria|Actinobacteria|Coriobacteriia	80.0	93.3	66.7
Bacteria|Actinobacteria|Coriobacteriia|Coriobacteriales	80.0	93.3	66.7
Bacteria|Actinobacteria|Coriobacteriia|Coriobacteriales|Coriobacteriaceae	80.0	93.3	66.7
Bacteria|Firmicutes|Clostridia|Clostridiales|Lachnospiraceae|Catonella	73.3	93.3	53.3
Bacteria|Firmicutes|Clostridia|Clostridiales|Peptostreptococcaceae|Peptostreptococc	81.7	93.3	70.0
Bacteria|Proteobacteria|Betaproteobacteria|Neisseriales|Neisseriaceae_Other	67.5	93.3	41.7
Bacteria|Proteobacteria|Gammaproteobacteria|Cardiobacteriales	65.0	93.3	36.7
Bacteria|Proteobacteria|Gammaproteobacteria|Cardiobacteriales|Cardiobacteriaceae	65.0	93.3	36.7
Bacteria|Proteobacteria|Gammaproteobacteria|Cardiobacteriales|Cardiobacteriaceae|Ca	62.5	93.3	31.7

Taxa	Present in at least one oral cavity and esophagus	Present in at least one oral cavity site	Present in at least one esophagus site
Bacteria|Firmicutes|Erysipelotrichi|Erysipelotrichales|Erysipelotrichaceae|Bulleidi	81.7	91.7	71.7
Bacteria|Firmicutes|Bacilli|Lactobacillales|Aerococcaceae	80.8	90.0	71.7
Bacteria|Firmicutes|Clostridia|Clostridiales|[Tissierellaceae]	75.8	90.0	61.7
Bacteria|Firmicutes|Clostridia|Clostridiales|Veillonellaceae|Megasphaera	80.8	90.0	71.7
Bacteria|TM7|TM7-3|CW040	82.5	90.0	75.0
Bacteria|Actinobacteria|Actinobacteria|Actinomycetales|Propionibacteriaceae	89.2	88.3	90.0
Bacteria|Bacteroidetes|Bacteroidia|Bacteroidales|Porphyromonadaceae|Paludibacter	62.5	86.7	38.3
Bacteria|Firmicutes|Clostridia|Clostridiales|[Tissierellaceae]|Parvimonas	73.3	86.7	60.0
Bacteria|Firmicutes|Clostridia|Clostridiales|Lachnospiraceae|Oribacterium	77.5	86.7	68.3
Bacteria|TM7|TM7-3|I025	70.8	86.7	55.0
Bacteria|TM7|TM7-3|I025|Rs-045	70.8	86.7	55.0
Bacteria|TM7|TM7-3|I025|Rs-045_unclassified	70.8	86.7	55.0
Bacteria|Actinobacteria|Actinobacteria|Actinomycetales|Propionibacteriaceae_unclass	66.7	85.0	48.3
Bacteria|Proteobacteria|Alphaproteobacteria	92.5	85.0	100.0
Bacteria|Proteobacteria|Deltaproteobacteria	78.3	85.0	71.7
Bacteria|Proteobacteria|Gammaproteobacteria|Pasteurellales|Pasteurellaceae|Actinoba	88.3	85.0	91.7
unclassified	79.2	85.0	73.3
Bacteria|Firmicutes|Bacilli|Lactobacillales|Aerococcaceae|Abiotrophia	69.2	83.3	55.0
Bacteria|Proteobacteria|Gammaproteobacteria|Pseudomonadales	91.7	83.3	100.0
Bacteria|Tenericutes	73.3	83.3	63.3
Bacteria|SR1	70.0	83.3	56.7
Bacteria|Tenericutes|Mollicutes	71.7	81.7	61.7
Bacteria|Actinobacteria|Coriobacteriia|Coriobacteriales|Coriobacteriaceae_unclassif	61.7	80.0	43.3
Bacteria|Firmicutes|Clostridia|Clostridiales|Lachnospiraceae|Butyrivibrio	59.2	80.0	38.3
Bacteria|Firmicutes|Clostridia|Clostridiales|Lachnospiraceae|Shuttleworthia	65.0	80.0	50.0
Bacteria|Synergistetes	58.3	80.0	36.7
Bacteria|Synergistetes|Synergistia	58.3	80.0	36.7
Bacteria|Synergistetes|Synergistia|Synergistales	58.3	80.0	36.7
Bacteria|Synergistetes|Synergistia|Synergistales|Dethiosulfovibrionaceae	58.3	80.0	36.7
Bacteria|Firmicutes|Clostridia|Clostridiales|Peptostreptococcaceae_unclassified	60.8	78.3	43.3
Bacteria|Proteobacteria|Alphaproteobacteria|Caulobacterales	89.2	78.3	100.0
Bacteria|Proteobacteria|Alphaproteobacteria|Caulobacterales|Caulobacteraceae	89.2	78.3	100.0

Taxa	Present in at least one oral cavity and esophagus	Present in at least one oral cavity site	Present in at least one esophagus site
Bacteria|Proteobacteria|Betaproteobacteria|Neisseriales|Neisseriaceae|Kingella	52.5	78.3	26.7
Bacteria|Synergistetes|Synergistia|Synergistales|Dethiosulfovibrionaceae|TG5	56.7	78.3	35.0
Bacteria|Actinobacteria|Actinobacteria|Actinomycetales|Actinomycetaceae_unclassifie	47.5	76.7	18.3
Bacteria|Actinobacteria|Actinobacteria|Bifidobacteriales	67.5	76.7	58.3
Bacteria|Actinobacteria|Actinobacteria|Bifidobacteriales|Bifidobacteriaceae	67.5	76.7	58.3
Bacteria|Actinobacteria|Coriobacteriia|Coriobacteriales|Coriobacteriaceae|Atopobium	64.2	76.7	51.7
Bacteria|Bacteroidetes|Bacteroidia|Bacteroidales|Bacteroidaceae	77.5	76.7	78.3
Bacteria|Bacteroidetes|Bacteroidia|Bacteroidales|Bacteroidaceae|Bacteroides	77.5	76.7	78.3
Bacteria|Firmicutes|Clostridia|Clostridiales|Peptococcaceae	58.3	76.7	40.0
Bacteria|Firmicutes|Clostridia|Clostridiales|Peptococcaceae|Peptococcus	54.2	76.7	31.7
Bacteria|Proteobacteria|Gammaproteobacteria|Pseudomonadales|Moraxellaceae	87.5	76.7	98.3
Bacteria|Proteobacteria|Gammaproteobacteria|Pseudomonadales|Moraxellaceae|Moraxella	65.8	76.7	55.0
Bacteria|Proteobacteria|Alphaproteobacteria|Caulobacterales|Caulobacteraceae_unclas	87.5	75.0	100.0
Bacteria|GN02	63.3	73.3	53.3
Bacteria|GN02|BD1-5	63.3	73.3	53.3
Bacteria|Tenericutes|Mollicutes|Mycoplasmatales	63.3	73.3	53.3
Bacteria|Tenericutes|Mollicutes|Mycoplasmatales|Mycoplasmataceae	63.3	73.3	53.3
Bacteria|Tenericutes|Mollicutes|Mycoplasmatales|Mycoplasmataceae|Mycoplasma	63.3	73.3	53.3
Bacteria|Firmicutes|Clostridia|Clostridiales|[Mogibacteriaceae]|Mogibacterium	69.2	71.7	66.7
Bacteria|Firmicutes|Clostridia|Clostridiales|Veillonellaceae|Schwartzia	46.7	71.7	21.7
Bacteria|TM7|TM7-3|CW040|F16	60.0	71.7	48.3
Bacteria|TM7|TM7-3|CW040|F16_unclassified	60.0	71.7	48.3
Bacteria|Proteobacteria|Gammaproteobacteria|Enterobacteriales	83.3	66.7	100.0
Bacteria|Proteobacteria|Gammaproteobacteria|Enterobacteriales|Enterobacteriaceae	83.3	66.7	100.0
Bacteria|Proteobacteria|Gammaproteobacteria|Enterobacteriales|Enterobacteriaceae_un	83.3	66.7	100.0
Bacteria|Firmicutes|Clostridia|Clostridiales|[Mogibacteriaceae]_Other	40.0	56.7	23.3
Bacteria|Proteobacteria|Deltaproteobacteria|Desulfobacterales	34.2	56.7	11.7
Bacteria|Proteobacteria|Deltaproteobacteria|Desulfobacterales|Desulfobulbaceae	34.2	56.7	11.7
Bacteria|Proteobacteria|Deltaproteobacteria|Desulfobacterales|Desulfobulbaceae|Desu	34.2	56.7	11.7
Bacteria|Proteobacteria|Deltaproteobacteria|Desulfovibrionales	52.5	55.0	50.0
Bacteria|Firmicutes|Bacilli|Lactobacillales|Lactobacillaceae	64.2	53.3	75.0
Bacteria|Firmicutes|Bacilli|Lactobacillales|Lactobacillaceae|Lactobacillus	64.2	53.3	75.0

Taxa	Present in at least one oral cavity and esophagus	Present in at least one oral cavity site	Present in at least one esophagus site
Bacteria|Firmicutes|Clostridia|Clostridiales|Veillonellaceae_unclassified	36.7	53.3	20.0
Bacteria|Proteobacteria|Alphaproteobacteria|Rhizobiales	76.7	53.3	100.0
Bacteria|Proteobacteria|Betaproteobacteria|Burkholderiales|Comamonadaceae_Other	50.0	51.7	48.3
Bacteria|Firmicutes|Clostridia|Clostridiales|Ruminococcaceae	66.7	50.0	83.3
Bacteria|Proteobacteria|Deltaproteobacteria|Desulfovibrionales|Desulfovibrionaceae	49.2	50.0	48.3
Bacteria|Proteobacteria|Deltaproteobacteria|Desulfovibrionales|Desulfovibrionaceae|	43.3	50.0	36.7
Bacteria|Firmicutes|Clostridia|Clostridiales|[Mogibacteriaceae]|Anaerovorax	35.8	48.3	23.3
Bacteria|Firmicutes|Erysipelotrichi|Erysipelotrichales|Erysipelotrichaceae|Sharpea	32.5	48.3	16.7
Bacteria|Proteobacteria|Betaproteobacteria|Rhodocyclales	55.0	48.3	61.7
Bacteria|Proteobacteria|Betaproteobacteria|Rhodocyclales|Rhodocyclaceae	55.0	48.3	61.7
Bacteria|Actinobacteria|Actinobacteria|Bifidobacteriales|Bifidobacteriaceae_unclass	43.3	46.7	40.0
Bacteria|Proteobacteria|Alphaproteobacteria|Sphingomonadales	73.3	46.7	100.0
Bacteria|Proteobacteria|Alphaproteobacteria|Sphingomonadales|Sphingomonadaceae	73.3	46.7	100.0
Bacteria|Actinobacteria|Actinobacteria|Bifidobacteriales|Bifidobacteriaceae|Bifidob	47.5	45.0	50.0
Bacteria|Tenericutes|Mollicutes|RF39	36.7	43.3	30.0
Bacteria|Firmicutes|Bacilli|Lactobacillales_Other	34.2	41.7	26.7
Bacteria|Proteobacteria|Gammaproteobacteria|Pseudomonadales|Pseudomonadaceae	65.0	41.7	88.3
Bacteria|Proteobacteria|Gammaproteobacteria|Pseudomonadales|Pseudomonadaceae|Pseudo	64.2	41.7	86.7
Bacteria|Actinobacteria|Actinobacteria|Bifidobacteriales|Bifidobacteriaceae|Scardov	28.3	40.0	16.7
Bacteria|Firmicutes|Bacilli|Bacillales	64.2	38.3	90.0
Bacteria|Firmicutes|Clostridia|Clostridiales|Clostridiaceae	46.7	38.3	55.0
Bacteria|Firmicutes|Clostridia|Clostridiales|Ruminococcaceae_unclassified	58.3	38.3	78.3
Bacteria|Firmicutes|Clostridia|Clostridiales|Eubacteriaceae	25.8	36.7	15.0
Bacteria|Firmicutes|Clostridia|Clostridiales|Eubacteriaceae|Pseudoramibacter_Eubact	25.0	36.7	13.3
Bacteria|Proteobacteria|Epsilonproteobacteria|Campylobacterales|Helicobacteraceae	43.3	36.7	50.0
Bacteria|Proteobacteria|Gammaproteobacteria|Xanthomonadales	68.3	36.7	100.0
Bacteria|Proteobacteria|Gammaproteobacteria|Xanthomonadales|Xanthomonadaceae	66.7	36.7	96.7
Bacteria|Firmicutes|Clostridia|Clostridiales|Clostridiaceae|Clostridium	35.8	35.0	36.7
Bacteria|Proteobacteria|Alphaproteobacteria|Sphingomonadales|Sphingomonadaceae|Sphi	67.5	35.0	100.0
Bacteria|Tenericutes|Mollicutes|Acholeplasmatales	29.2	35.0	23.3
Bacteria|Tenericutes|Mollicutes|Acholeplasmatales|Acholeplasmataceae	29.2	35.0	23.3
Bacteria|Tenericutes|Mollicutes|Acholeplasmatales|Acholeplasmataceae|Acholeplasma	29.2	35.0	23.3

Taxa	Present in at least one oral cavity and esophagus	Present in at least one oral cavity site	Present in at least one esophagus site
Bacteria|Tenericutes|RF3	22.5	33.3	11.7
Bacteria|Tenericutes|RF3|ML615J-28	22.5	33.3	11.7
Bacteria|Proteobacteria|Gammaproteobacteria|Xanthomonadales|Xanthomonadaceae|Stenot	63.3	31.7	95.0
Bacteria|Bacteroidetes|Bacteroidia|Bacteroidales|S24-7	60.0	30.0	90.0
Bacteria|Bacteroidetes|Bacteroidia|Bacteroidales|S24-7_unclassified	60.0	30.0	90.0
Bacteria|Firmicutes|Bacilli|Bacillales|Bacillaceae	41.7	30.0	53.3
Bacteria|Firmicutes|Bacilli|Bacillales|Bacillaceae|Bacillus	38.3	30.0	46.7
Bacteria|Proteobacteria|Alphaproteobacteria|Rhizobiales|Bradyrhizobiaceae	65.0	30.0	100.0
Bacteria|Firmicutes|Clostridia|Clostridiales|Lachnospiraceae_Other	40.0	28.3	51.7
Bacteria|Proteobacteria|Alphaproteobacteria|Sphingomonadales|Sphingomonadaceae|Novo	56.7	28.3	85.0
Bacteria|Proteobacteria|Betaproteobacteria|Burkholderiales|Oxalobacteraceae	63.3	28.3	98.3
Bacteria|Proteobacteria|Alphaproteobacteria|Rhizobiales|Bradyrhizobiaceae|Bradyrhiz	63.3	26.7	100.0
Bacteria|Proteobacteria|Gammaproteobacteria|Pasteurellales|Pasteurellaceae_Other	25.0	26.7	23.3
Bacteria|Firmicutes|Bacilli|Lactobacillales|Aerococcaceae|Aerococcus	33.3	25.0	41.7
Bacteria|Firmicutes|Clostridia|Clostridiales|[Tissierellaceae]|Peptoniphilus	27.5	25.0	30.0
Bacteria|Firmicutes|Erysipelotrichi|Erysipelotrichales|Erysipelotrichaceae_unclassi	21.7	25.0	18.3
Bacteria|Proteobacteria|Alphaproteobacteria|Rhizobiales|Brucellaceae	57.5	25.0	90.0
Bacteria|Bacteroidetes|Bacteroidia|Bacteroidales|BS11	18.3	23.3	13.3
Bacteria|Bacteroidetes|Bacteroidia|Bacteroidales|BS11_unclassified	18.3	23.3	13.3
Bacteria|Firmicutes|Clostridia|Clostridiales|Lachnospiraceae|Blautia	40.8	23.3	58.3
Bacteria|Proteobacteria|Alphaproteobacteria|Rhizobiales|Brucellaceae|Ochrobactrum	56.7	23.3	90.0
Bacteria|Firmicutes|Bacilli|Gemellales|Gemellaceae|Gemella	25.0	21.7	28.3
Bacteria|Firmicutes|Clostridia|Clostridiales|[Tissierellaceae]|Finegoldia	26.7	21.7	31.7
Bacteria|Proteobacteria|Betaproteobacteria|Burkholderiales|Alcaligenaceae	35.0	21.7	48.3
Bacteria|Bacteroidetes|[Saprospirae]	53.3	20.0	86.7
Bacteria|Bacteroidetes|[Saprospirae]|[Saprospirales]	53.3	20.0	86.7
Bacteria|Bacteroidetes|[Saprospirae]|[Saprospirales]|Chitinophagaceae	53.3	20.0	86.7
Bacteria|Bacteroidetes|Flavobacteriia|Flavobacteriales|[Weeksellaceae]|Chryseobacte	38.3	20.0	56.7
Bacteria|Firmicutes|Clostridia|Clostridiales|[Tissierellaceae]|Anaerococcus	25.0	20.0	30.0
Bacteria|Fusobacteria|Fusobacteriia|Fusobacteriales|Leptotrichiaceae_unclassified	26.7	20.0	33.3
Bacteria|Fusobacteria|Fusobacteriia|Fusobacteriales|Leptotrichiaceae|Sneathia	23.3	20.0	26.7
Bacteria|Firmicutes|Bacilli|Bacillales|Staphylococcaceae	52.5	18.3	86.7

Taxa	Present in at least one oral cavity and esophagus	Present in at least one oral cavity site	Present in at least one esophagus site
Bacteria|Firmicutes|Bacilli|Bacillales|Staphylococcaceae|Staphylococcus	52.5	18.3	86.7
Bacteria|Firmicutes|Clostridia|Clostridiales|Lachnospiraceae|Roseburia	26.7	18.3	35.0
Bacteria|Firmicutes|Clostridia|Clostridiales|Ruminococcaceae|Faecalibacterium	25.8	18.3	33.3
Bacteria|Firmicutes|Clostridia|Clostridiales|Ruminococcaceae|Oscillospira	45.0	18.3	71.7
Bacteria|Proteobacteria|Alphaproteobacteria|Rhizobiales|Methylobacteriaceae	58.3	18.3	98.3
Bacteria|Proteobacteria|Betaproteobacteria|Burkholderiales|Oxalobacteraceae|Cupriav	34.2	18.3	50.0
Bacteria|Bacteroidetes|[Saprospirae]|[Saprospirales]|Chitinophagaceae|Sediminibacte	50.8	16.7	85.0
Bacteria|Bacteroidetes|Sphingobacteriia	29.2	16.7	41.7
Bacteria|Bacteroidetes|Sphingobacteriia|Sphingobacteriales	29.2	16.7	41.7
Bacteria|Bacteroidetes|Sphingobacteriia|Sphingobacteriales|Sphingobacteriaceae	28.3	16.7	40.0
Bacteria|Bacteroidetes|Sphingobacteriia|Sphingobacteriales|Sphingobacteriaceae|Pedo	23.3	16.7	30.0
Bacteria|Chloroflexi	15.0	16.7	13.3
Bacteria|Firmicutes|Clostridia|Clostridiales_Other	35.8	16.7	55.0
Bacteria|Firmicutes|Clostridia|Clostridiales|Lachnospiraceae|Coprococcus	35.8	16.7	55.0
Bacteria|Proteobacteria|Alphaproteobacteria|Caulobacterales|Caulobacteraceae|Phenyl	33.3	16.7	50.0
Bacteria|Proteobacteria|Alphaproteobacteria|Rhizobiales|Methylobacteriaceae|Methylo	56.7	16.7	96.7
Bacteria|Proteobacteria|Alphaproteobacteria|Rhodospirillales	55.0	16.7	93.3
Bacteria|Proteobacteria|Betaproteobacteria|Burkholderiales|Oxalobacteraceae_unclass	39.2	16.7	61.7
Bacteria|Proteobacteria|Deltaproteobacteria|GMD14H09	13.3	16.7	10.0
Bacteria|Proteobacteria|Gammaproteobacteria|Aeromonadales	20.8	16.7	25.0
Bacteria|Actinobacteria|Actinobacteria|Actinomycetales|Microbacteriaceae	32.5	15.0	50.0
Bacteria|Cyanobacteria	30.0	15.0	45.0
Bacteria|Firmicutes|Clostridia|Clostridiales|Clostridiaceae_unclassified	32.5	15.0	50.0
Bacteria|Firmicutes|Clostridia|Clostridiales|Lachnospiraceae|Moryella	14.2	15.0	13.3
Bacteria|Proteobacteria|Betaproteobacteria|Burkholderiales|Alcaligenaceae|Sutterell	25.8	15.0	36.7
Bacteria|Proteobacteria|Gammaproteobacteria|Enterobacteriales|Enterobacteriaceae_Ot	32.5	15.0	50.0
Bacteria|Proteobacteria|Gammaproteobacteria|Pseudomonadales|Moraxellaceae|Acinetoba	51.7	15.0	88.3
Bacteria|Proteobacteria|Gammaproteobacteria|Pseudomonadales|Moraxellaceae|Enhydroba	40.8	15.0	66.7
Bacteria|Actinobacteria|Actinobacteria|Actinomycetales|Micrococcaceae|Renibacterium	15.8	13.3	18.3
Bacteria|Actinobacteria|Actinobacteria|Actinomycetales|Nocardioidaceae	18.3	13.3	23.3
Bacteria|Firmicutes|Clostridia|Clostridiales|Ruminococcaceae|Ruminococcus	26.7	13.3	40.0
Bacteria|Proteobacteria|Gammaproteobacteria|Aeromonadales|Succinivibrionaceae	13.3	13.3	13.3

Taxa	Present in at least one oral cavity and esophagus	Present in at least one oral cavity site	Present in at least one esophagus site
Bacteria|Proteobacteria|Gammaproteobacteria|Aeromonadales|Succinivibrionaceae|Succi	13.3	13.3	13.3
Bacteria|Tenericutes|Mollicutes|Mycoplasmatales|Mycoplasmataceae|Ureaplasma	18.3	13.3	23.3
Bacteria|Cyanobacteria|Chloroplast	18.3	11.7	25.0
Bacteria|Cyanobacteria|Chloroplast|Streptophyta	18.3	11.7	25.0
Bacteria|Firmicutes|Erysipelotrichi|Erysipelotrichales|Erysipelotrichaceae|Allobacu	38.3	11.7	65.0
Bacteria|Proteobacteria|Alphaproteobacteria|Rhizobiales|Hyphomicrobiaceae	24.2	11.7	36.7
Bacteria|Proteobacteria|Alphaproteobacteria|Rhizobiales|Phyllobacteriaceae	31.7	11.7	51.7
Bacteria|Proteobacteria|Alphaproteobacteria|Rhizobiales|Phyllobacteriaceae|Phylloba	31.7	11.7	51.7
Bacteria|Proteobacteria|Alphaproteobacteria|Rhodospirillales|Acetobacteraceae	46.7	11.7	81.7
Bacteria|Proteobacteria|Betaproteobacteria|Burkholderiales|Comamonadaceae|Acidovora	30.0	11.7	48.3
Bacteria|Proteobacteria|Gammaproteobacteria|Xanthomonadales|Xanthomonadaceae_unclas	29.2	11.7	46.7
Bacteria|Actinobacteria|Actinobacteria|Actinomycetales|Propionibacteriaceae|Propion	45.8	10.0	81.7
Bacteria|Actinobacteria|Actinobacteria|Bifidobacteriales|Bifidobacteriaceae_Other	15.8	10.0	21.7
Bacteria|Bacteroidetes|Bacteroidia|Bacteroidales|Rikenellaceae	20.0	10.0	30.0
Bacteria|Firmicutes|Clostridia|Clostridiales|Christensenellaceae	25.0	10.0	40.0
Bacteria|Firmicutes|Clostridia|Clostridiales|Lachnospiraceae|Dorea	26.7	10.0	43.3
Bacteria|Proteobacteria|Gammaproteobacteria|Pseudomonadales|Pseudomonadaceae_unclas	30.0	10.0	50.0
