# Supplementary material for: Microbial Similarity and Preference for Specific Sites in Healthy Oral Cavity and Esophagus
Source: Front Microbiol. 2018 Jul 17;9:1603. doi: 10.3389/fmicb.2018.01603 (PMC6056649; doi:10.3389/fmicb.2018.01603)
Supplement: Supplementary file 4 [file Table_4.pdf]

Table S4: Read counts for all digestive tract samples for each microbial clade

	Sample ID	C1.002	C1.003	C1.006	C1.009	C1.010	C1.012	C1.013	C1.014	C1.016	C1.017	C1.018	C1.019	C1.020	C1.021	C1.024	C1.025
Microbial Clade	Group	Oral Ca	Oral Ca	Oral Ca	Oral Ca	Oral Ca	Oral Ca	Oral Ca	Oral Ca	Oral Ca	Oral Ca	Oral Ca	Oral Ca	Oral Ca	Oral Ca	Oral Ca	Oral Ca
	Body Site	Sa	Sa	Sa	Sa	Sa	Sa	Sa	Sa	Sa	Sa	Sa	Sa	Sa	Sa	Sa	Sa
Archaea		0	0	0	0	0	0	0	0	0	0	0	0	0	0	0	0
Bacteria		18144	19797	25218	21783	10889	6757	17043	19376	17903	13503	19265	19521	24414	14616	22088	11425
unclassified		4	97	0	120	16	74	2	0	0	6	149	0	8	5	69	38
Bacteria|[Thermi]		0	0	0	0	0	0	0	0	0	0	0	0	0	0	0	0
Bacteria|Acidobacteria		0	0	0	0	0	0	0	0	0	0	0	0	0	0	1	0
Bacteria|Actinobacteria		2398	2859	2173	5474	527	564	6899	5178	1741	3542	3966	6643	8082	3547	1003	958
Bacteria|Armatimonadetes		0	0	0	0	0	1	0	0	0	0	0	0	0	0	0	0
Bacteria|Bacteroidetes		4193	7297	5545	6957	3519	2051	4030	5067	3888	3816	5903	3739	6798	5026	6534	3761
Bacteria|BRC1		0	0	0	0	0	0	0	0	0	0	0	0	0	0	0	0
Bacteria|Chlamydiae		0	0	0	0	0	0	0	0	0	0	0	0	0	0	0	0
Bacteria|Chlorobi		0	0	0	0	0	0	0	0	0	0	0	0	0	0	0	0
Bacteria|Chloroflexi		0	0	0	0	1	0	0	0	0	0	0	0	0	0	0	0
Bacteria|Cyanobacteria		0	0	0	1	0	0	1	0	0	0	0	0	0	2	0	0
Bacteria|Elusimicrobia		0	0	0	0	0	0	0	0	0	0	6	0	0	0	0	0
Bacteria|Fibrobacteres		0	0	0	2	0	0	0	0	0	0	0	0	0	0	0	0
Bacteria|Firmicutes		1422	1345	884	2368	721	507	3414	1300	574	1745	2816	1636	3346	1029	1946	757
Bacteria|Fusobacteria		32	76	58	73	113	44	40	27	9	27	273	32	49	13	251	99
Bacteria|Gemmatimonadetes		0	0	0	0	0	0	0	0	0	0	0	0	0	0	0	0
Bacteria|GN02		3	2	2	4	15	2	0	0	0	1	0	2	0	0	74	10
Bacteria|Lentisphaerae		0	0	0	1	0	0	0	0	0	0	0	0	0	0	0	0
Bacteria|Planctomycetes		0	0	0	0	0	0	0	0	0	0	0	0	0	0	0	0
Bacteria|Proteobacteria		9826	7715	16121	6479	4989	3475	2021	7608	11544	4257	5268	7173	5909	4883	10927	5509
Bacteria|Spirochaetes		100	156	59	114	131	0	217	5	15	36	259	2	89	23	163	21
Bacteria|SR1		1	2	8	0	13	0	2	0	3	0	3	1	0	1	11	2
Bacteria|Synergistetes		22	43	15	51	167	2	77	4	6	3	108	0	24	15	70	11
Bacteria|Tenericutes		4	1	0	13	33	0	6	1	0	1	33	2	1	0	44	11
Bacteria|Thermotogae		0	0	0	0	0	0	0	0	0	0	0	0	0	0	0	0
Bacteria|TM6		0	0	0	0	0	0	0	0	0	0	0	0	0	0	0	0
Bacteria|TM7		139	204	353	125	644	37	334	186	123	69	481	291	108	72	995	248
Bacteria|Verrucomicrobia		0	0	0	1	0	0	0	0	0	0	0	0	0	0	0	0
Bacteria|WPS-2		0	0	0	0	0	0	0	0	0	0	0	0	0	0	0	0
Bacteria|WS6		0	0	0	0	0	0	0	0	0	0	0	0	0	0	0	0
Bacteria|[Thermi]|Deinococci		0	0	0	0	0	0	0	0	0	0	0	0	0	0	0	0
Bacteria|Acidobacteria|[Chloracidobacteria]		0	0	0	0	0	0	0	0	0	0	0	0	0	0	0	0
Bacteria|Acidobacteria|Acidobacteria-6		0	0	0	0	0	0	0	0	0	0	0	0	0	0	0	0
Bacteria|Acidobacteria|Acidobacteriia		0	0	0	0	0	0	0	0	0	0	0	0	0	0	0	0

Microbial Clade	Sample ID	C1.002	C1.003	C1.006	C1.009	C1.010	C1.012	C1.013	C1.014	C1.016	C1.017	C1.018	C1.019	C1.020	C1.021	C1.024	C1.025
	Group	Oral Ca	Oral Ca	Oral Ca	Oral Ca	Oral Ca	Oral Ca	Oral Ca	Oral Ca	Oral Ca	Oral Ca	Oral Ca	Oral Ca	Oral Ca	Oral Ca	Oral Ca	Oral Ca
	Body Site	Sa	Sa	Sa	Sa	Sa	Sa	Sa	Sa	Sa	Sa	Sa	Sa	Sa	Sa	Sa	Sa
Bacteria|Acidobacteria|Holophagae		0	0	0	0	0	0	0	0	0	0	0	0	0	0	0	0
Bacteria|Acidobacteria|Solibacteres		0	0	0	0	0	0	0	0	0	0	0	0	0	0	1	0
Bacteria|Acidobacteria|Sva0725		0	0	0	0	0	0	0	0	0	0	0	0	0	0	0	0
Bacteria|Actinobacteria|Acidimicrobiia		0	0	0	0	0	0	0	0	0	0	0	0	0	0	0	0
Bacteria|Actinobacteria|Actinobacteria		2275	2787	2139	5446	502	553	6713	4824	1708	3220	3777	6520	7864	3266	983	930
Bacteria|Actinobacteria|Coriobacteriia		123	72	34	28	25	11	186	354	33	322	189	123	218	281	20	28
Bacteria|Actinobacteria|Nitriliruptoria		0	0	0	0	0	0	0	0	0	0	0	0	0	0	0	0
Bacteria|Actinobacteria|Rubrobacteria		0	0	0	0	0	0	0	0	0	0	0	0	0	0	0	0
Bacteria|Actinobacteria|Thermoleophilia		0	0	0	0	0	0	0	0	0	0	0	0	0	0	0	0
Bacteria|Armatimonadetes|[Fimbriimonadia]		0	0	0	0	0	1	0	0	0	0	0	0	0	0	0	0
Bacteria|Armatimonadetes|Armatimonadia		0	0	0	0	0	0	0	0	0	0	0	0	0	0	0	0
Bacteria|Bacteroidetes|[Saprospirae]		0	0	0	1	1	0	0	0	0	0	0	1	0	0	1	0
Bacteria|Bacteroidetes|Bacteroidia		4137	7254	5372	6825	3337	1987	3950	5007	3865	3793	5854	3718	6752	4982	6180	3708
Bacteria|Bacteroidetes|Cytophagia		0	0	0	0	0	0	0	0	0	0	0	0	0	0	0	0
Bacteria|Bacteroidetes|Flavobacteriia		56	43	173	131	181	64	80	60	23	23	46	20	46	44	353	46
Bacteria|Bacteroidetes|Sphingobacteriia		0	0	0	0	0	0	0	0	0	0	3	0	0	0	0	7
Bacteria|BRC1|PRR-11		0	0	0	0	0	0	0	0	0	0	0	0	0	0	0	0
Bacteria|Chlamydiae|Chlamydiia		0	0	0	0	0	0	0	0	0	0	0	0	0	0	0	0
Bacteria|Chlorobi|Ignavibacteria		0	0	0	0	0	0	0	0	0	0	0	0	0	0	0	0
Bacteria|Chlorobi|OPB56		0	0	0	0	0	0	0	0	0	0	0	0	0	0	0	0
Bacteria|Chloroflexi|Anaerolineae		0	0	0	0	1	0	0	0	0	0	0	0	0	0	0	0
Bacteria|Chloroflexi|C0119		0	0	0	0	0	0	0	0	0	0	0	0	0	0	0	0
Bacteria|Chloroflexi|Ellin6529		0	0	0	0	0	0	0	0	0	0	0	0	0	0	0	0
Bacteria|Chloroflexi|S085		0	0	0	0	0	0	0	0	0	0	0	0	0	0	0	0
Bacteria|Chloroflexi|Thermomicrobia		0	0	0	0	0	0	0	0	0	0	0	0	0	0	0	0
Bacteria|Chloroflexi|TK17		0	0	0	0	0	0	0	0	0	0	0	0	0	0	0	0
Bacteria|Cyanobacteria|4C0d-2		0	0	0	0	0	0	0	0	0	0	0	0	0	0	0	0
Bacteria|Cyanobacteria|Chloroplast		0	0	0	1	0	0	1	0	0	0	0	0	0	2	0	0
Bacteria|Cyanobacteria|ML635J-21		0	0	0	0	0	0	0	0	0	0	0	0	0	0	0	0
Bacteria|Cyanobacteria|Oscillatoriophycideae		0	0	0	0	0	0	0	0	0	0	0	0	0	0	0	0
Bacteria|Elusimicrobia|Elusimicrobia		0	0	0	0	0	0	0	0	0	0	6	0	0	0	0	0
Bacteria|Elusimicrobia|Endomicrobia		0	0	0	0	0	0	0	0	0	0	0	0	0	0	0	0
Bacteria|Fibrobacteres|Fibrobacteria		0	0	0	2	0	0	0	0	0	0	0	0	0	0	0	0
Bacteria|Firmicutes|Bacilli		724	645	521	820	293	287	1432	573	207	778	1020	862	1396	417	996	376
Bacteria|Firmicutes|Clostridia		652	670	350	1476	416	216	1939	648	363	897	1678	691	1883	589	921	260
Bacteria|Firmicutes|Erysipelotrichi		46	30	13	72	12	4	43	79	4	70	118	83	67	23	29	121
Bacteria|Fusobacteria|Fusobacteriia		32	76	58	73	113	44	40	27	9	27	273	32	49	13	251	99

Microbial Clade																	
	Sample ID	C1.002	C1.003	C1.006	C1.009	C1.010	C1.012	C1.013	C1.014	C1.016	C1.017	C1.018	C1.019	C1.020	C1.021	C1.024	C1.025
	Group	Oral Ca	Oral Ca	Oral Ca	Oral Ca	Oral Ca	Oral Ca	Oral Ca	Oral Ca	Oral Ca	Oral Ca	Oral Ca	Oral Ca	Oral Ca	Oral Ca	Oral Ca	Oral Ca
	Body Site	Sa	Sa	Sa	Sa	Sa	Sa	Sa	Sa	Sa	Sa	Sa	Sa	Sa	Sa	Sa	Sa
Bacteria|Gemmatimonadetes|Gemmatimonadetes		0	0	0	0	0	0	0	0	0	0	0	0	0	0	0	0
Bacteria|GN02|BD1-5		3	2	2	4	15	2	0	0	0	1	0	2	0	0	74	10
Bacteria|Lentisphaerae|[Lentisphaeria]		0	0	0	1	0	0	0	0	0	0	0	0	0	0	0	0
Bacteria|Planctomycetes|OM190		0	0	0	0	0	0	0	0	0	0	0	0	0	0	0	0
Bacteria|Planctomycetes|Phycisphaerae		0	0	0	0	0	0	0	0	0	0	0	0	0	0	0	0
Bacteria|Planctomycetes|Planctomycetia		0	0	0	0	0	0	0	0	0	0	0	0	0	0	0	0
Bacteria|Proteobacteria|Alphaproteobacteria		0	3	2	5	29	37	1	1	1	0	1	1	3	0	659	17
Bacteria|Proteobacteria|Betaproteobacteria		9074	6975	14460	5872	4450	3284	1663	6771	11084	3702	4886	6764	3826	4386	9252	5215
Bacteria|Proteobacteria|Deltaproteobacteria		11	24	0	38	62	2	82	1	5	3	63	0	46	23	12	5
Bacteria|Proteobacteria|Epsilonproteobacteria		20	15	33	39	15	10	16	8	5	32	54	23	32	11	61	13
Bacteria|Proteobacteria|Gammaproteobacteria		721	698	1626	525	433	142	259	827	449	520	264	385	2002	463	942	259
Bacteria|Proteobacteria|TA18		0	0	0	0	0	0	0	0	0	0	0	0	0	0	0	0
Bacteria|Spirochaetes|GN05		0	0	0	0	0	0	0	0	0	0	0	0	0	0	0	0
Bacteria|Spirochaetes|Spirochaetes		100	156	59	114	131	0	217	5	15	36	259	2	89	23	163	21
Bacteria|Synergistetes|Synergistia		22	43	15	51	167	2	77	4	6	3	108	0	24	15	70	11
Bacteria|Tenericutes|CK-1C4-19		0	0	0	0	0	0	0	0	0	0	0	0	0	0	0	0
Bacteria|Tenericutes|Mollicutes		4	0	0	13	27	0	4	1	0	1	30	2	1	0	38	11
Bacteria|Tenericutes|RF3		0	1	0	0	6	0	2	0	0	0	3	0	0	0	6	0
Bacteria|Thermotogae|Thermotogae		0	0	0	0	0	0	0	0	0	0	0	0	0	0	0	0
Bacteria|TM6|SBRH58		0	0	0	0	0	0	0	0	0	0	0	0	0	0	0	0
Bacteria|TM6|SJA-4		0	0	0	0	0	0	0	0	0	0	0	0	0	0	0	0
Bacteria|TM7|SC3		0	0	0	0	0	0	0	0	0	0	0	0	0	0	0	0
Bacteria|TM7|TM7-1		0	0	0	0	0	0	0	0	0	0	0	0	0	0	0	0
Bacteria|TM7|TM7-3		139	204	353	125	644	37	334	186	123	69	481	291	108	72	995	248
Bacteria|Verrucomicrobia|Verruco-5		0	0	0	1	0	0	0	0	0	0	0	0	0	0	0	0
Bacteria|Verrucomicrobia|Verrucomicrobiae		0	0	0	0	0	0	0	0	0	0	0	0	0	0	0	0
Bacteria|WS6|SC72		0	0	0	0	0	0	0	0	0	0	0	0	0	0	0	0
Bacteria|[Thermi]|Deinococci|Deinococcales		0	0	0	0	0	0	0	0	0	0	0	0	0	0	0	0
Bacteria|[Thermi]|Deinococci|Thermales		0	0	0	0	0	0	0	0	0	0	0	0	0	0	0	0
Bacteria|Acidobacteria|[Chloracidobacteria]|RB41		0	0	0	0	0	0	0	0	0	0	0	0	0	0	0	0
Bacteria|Acidobacteria|Acidobacteria-6|iii1-15		0	0	0	0	0	0	0	0	0	0	0	0	0	0	0	0
Bacteria|Acidobacteria|Acidobacteriia|Acidobacteriales		0	0	0	0	0	0	0	0	0	0	0	0	0	0	0	0
Bacteria|Acidobacteria|Holophagae|Holophagales		0	0	0	0	0	0	0	0	0	0	0	0	0	0	0	0
Bacteria|Acidobacteria|Solibacteres|Solibacterales		0	0	0	0	0	0	0	0	0	0	0	0	0	0	1	0
Bacteria|Acidobacteria|Sva0725|Sva0725		0	0	0	0	0	0	0	0	0	0	0	0	0	0	0	0
Bacteria|Actinobacteria|Acidimicrobiia|Acidimicrobiales		0	0	0	0	0	0	0	0	0	0	0	0	0	0	0	0
Bacteria|Actinobacteria|Actinobacteria|Actinomycetales		2269	2786	2139	5307	400	523	6412	4817	1705	3209	3757	6509	7805	3263	863	907

	Sample ID	C1.002	C1.003	C1.006	C1.009	C1.010	C1.012	C1.013	C1.014	C1.016	C1.017	C1.018	C1.019	C1.020	C1.021	C1.024	C1.025
Microbial Clade	Group	Oral Ca	Oral Ca	Oral Ca	Oral Ca	Oral Ca	Oral Ca	Oral Ca	Oral Ca	Oral Ca	Oral Ca	Oral Ca	Oral Ca	Oral Ca	Oral Ca	Oral Ca	Oral Ca
	Body Site	Sa	Sa	Sa	Sa	Sa	Sa	Sa	Sa	Sa	Sa	Sa	Sa	Sa	Sa	Sa	Sa
Bacteria|Actinobacteria|Actinobacteria|Bifidobacteriales		6	1	0	139	102	30	301	7	3	11	20	11	59	3	120	23
Bacteria|Actinobacteria|Actinobacteria|Micrococcales		0	0	0	0	0	0	0	0	0	0	0	0	0	0	0	0
Bacteria|Actinobacteria|Coriobacteriia|Coriobacteriales		123	72	34	28	25	11	186	354	33	322	189	123	218	281	20	28
Bacteria|Actinobacteria|Nitriliruptoria|Nitriliruptorales		0	0	0	0	0	0	0	0	0	0	0	0	0	0	0	0
Bacteria|Actinobacteria|Rubrobacteria|Rubrobacterales		0	0	0	0	0	0	0	0	0	0	0	0	0	0	0	0
Bacteria|Actinobacteria|Thermoleophilia|Gaiellales		0	0	0	0	0	0	0	0	0	0	0	0	0	0	0	0
Bacteria|Actinobacteria|Thermoleophilia|Solirubrobacterales		0	0	0	0	0	0	0	0	0	0	0	0	0	0	0	0
Bacteria|Armatimonadetes|[Fimbriimonadia]|[Fimbriimonadales]		0	0	0	0	0	1	0	0	0	0	0	0	0	0	0	0
Bacteria|Armatimonadetes|Armatimonadia|Armatimonadales		0	0	0	0	0	0	0	0	0	0	0	0	0	0	0	0
Bacteria|Bacteroidetes|[Saprospirae]|[Saprospirales]		0	0	0	1	1	0	0	0	0	0	0	1	0	0	1	0
Bacteria|Bacteroidetes|Bacteroidia|Bacteroidales		4137	7254	5372	6825	3337	1987	3950	5007	3865	3793	5854	3718	6752	4982	6180	3708
Bacteria|Bacteroidetes|Cytophagia|Cytophagales		0	0	0	0	0	0	0	0	0	0	0	0	0	0	0	0
Bacteria|Bacteroidetes|Flavobacteriia|Flavobacteriales		56	43	173	131	181	64	80	60	23	23	46	20	46	44	353	46
Bacteria|Bacteroidetes|Sphingobacteriia|Sphingobacteriales		0	0	0	0	0	0	0	0	0	0	3	0	0	0	0	7
Bacteria|Chlamydiae|Chlamydiia|Chlamydiales		0	0	0	0	0	0	0	0	0	0	0	0	0	0	0	0
Bacteria|Chlorobi|Ignavibacteria|Ignavibacteriales		0	0	0	0	0	0	0	0	0	0	0	0	0	0	0	0
Bacteria|Chloroflexi|Anaerolineae|Anaerolineales		0	0	0	0	1	0	0	0	0	0	0	0	0	0	0	0
Bacteria|Chloroflexi|Anaerolineae|Caldilineales		0	0	0	0	0	0	0	0	0	0	0	0	0	0	0	0
Bacteria|Chloroflexi|Anaerolineae|SBR1031		0	0	0	0	0	0	0	0	0	0	0	0	0	0	0	0
Bacteria|Chloroflexi|Thermomicrobia|JG30-KF-CM45		0	0	0	0	0	0	0	0	0	0	0	0	0	0	0	0
Bacteria|Chloroflexi|TK17|mle1-48		0	0	0	0	0	0	0	0	0	0	0	0	0	0	0	0
Bacteria|Cyanobacteria|4C0d-2|MLE1-12		0	0	0	0	0	0	0	0	0	0	0	0	0	0	0	0
Bacteria|Cyanobacteria|4C0d-2|SM2F09		0	0	0	0	0	0	0	0	0	0	0	0	0	0	0	0
Bacteria|Cyanobacteria|4C0d-2|YS2		0	0	0	0	0	0	0	0	0	0	0	0	0	0	0	0
Bacteria|Cyanobacteria|Chloroplast|Stramenopiles		0	0	0	0	0	0	0	0	0	0	0	0	0	0	0	0
Bacteria|Cyanobacteria|Chloroplast|Streptophyta		0	0	0	1	0	0	1	0	0	0	0	0	0	2	0	0
Bacteria|Cyanobacteria|Oscillatoriophycideae|Chroococcales		0	0	0	0	0	0	0	0	0	0	0	0	0	0	0	0
Bacteria|Cyanobacteria|Oscillatoriophycideae|Oscillatoriales		0	0	0	0	0	0	0	0	0	0	0	0	0	0	0	0
Bacteria|Elusimicrobia|Elusimicrobia|Elusimicrobiales		0	0	0	0	0	0	0	0	0	0	6	0	0	0	0	0
Bacteria|Fibrobacteres|Fibrobacteria|Fibrobacterales		0	0	0	2	0	0	0	0	0	0	0	0	0	0	0	0
Bacteria|Firmicutes|Bacilli|Bacillales		0	0	0	0	2	5	0	0	0	0	1	0	0	0	6	6
Bacteria|Firmicutes|Bacilli|Gemellales		4	12	5	12	5	8	13	1	0	4	28	5	10	4	49	2
Bacteria|Firmicutes|Bacilli|Lactobacillales		720	633	516	803	286	273	1419	572	207	774	985	857	1386	413	941	368
Bacteria|Firmicutes|Bacilli|Turicibacterales		0	0	0	2	0	0	0	0	0	0	0	0	0	0	0	0
Bacteria|Firmicutes|Clostridia|Clostridiales		652	670	350	1476	416	216	1939	648	363	897	1678	691	1883	589	921	260
Bacteria|Firmicutes|Clostridia|Halanaerobiales		0	0	0	0	0	0	0	0	0	0	0	0	0	0	0	0
Bacteria|Firmicutes|Clostridia|Thermoanaerobacterales		0	0	0	0	0	0	0	0	0	0	0	0	0	0	0	0

	Sample ID	C1.002	C1.003	C1.006	C1.009	C1.010	C1.012	C1.013	C1.014	C1.016	C1.017	C1.018	C1.019	C1.020	C1.021	C1.024	C1.025
Microbial Clade	Group	Oral Ca	Oral Ca	Oral Ca	Oral Ca	Oral Ca	Oral Ca	Oral Ca	Oral Ca	Oral Ca	Oral Ca	Oral Ca	Oral Ca	Oral Ca	Oral Ca	Oral Ca	Oral Ca
	Body Site	Sa	Sa	Sa	Sa	Sa	Sa	Sa	Sa	Sa	Sa	Sa	Sa	Sa	Sa	Sa	Sa
Bacteria|Firmicutes|Erysipelotrichi|Erysipelotrichales		46	30	13	72	12	4	43	79	4	70	118	83	67	23	29	121
Bacteria|Fusobacteria|Fusobacteriia|Fusobacteriales		32	76	58	73	113	44	40	27	9	27	273	32	49	13	251	99
Bacteria|Gemmatimonadetes|Gemmatimonadetes|Gemmatimonada		0	0	0	0	0	0	0	0	0	0	0	0	0	0	0	0
Bacteria|Lentisphaerae|[Lentisphaeria]|Z20		0	0	0	1	0	0	0	0	0	0	0	0	0	0	0	0
Bacteria|Planctomycetes|OM190|agg27		0	0	0	0	0	0	0	0	0	0	0	0	0	0	0	0
Bacteria|Planctomycetes|Phycisphaerae|Phycisphaerales		0	0	0	0	0	0	0	0	0	0	0	0	0	0	0	0
Bacteria|Planctomycetes|Planctomycetia|Gemmatales		0	0	0	0	0	0	0	0	0	0	0	0	0	0	0	0
Bacteria|Proteobacteria|Alphaproteobacteria|BD7-3		0	0	0	0	0	0	0	0	0	0	0	0	0	0	0	0
Bacteria|Proteobacteria|Alphaproteobacteria|Caulobacterales		0	1	0	1	4	3	1	0	1	0	1	0	2	0	14	1
Bacteria|Proteobacteria|Alphaproteobacteria|Ellin329		0	0	0	0	0	0	0	0	0	0	0	0	0	0	0	0
Bacteria|Proteobacteria|Alphaproteobacteria|RF32		0	0	0	1	0	0	0	0	0	0	0	0	0	0	0	0
Bacteria|Proteobacteria|Alphaproteobacteria|Rhizobiales		0	1	1	1	4	1	0	0	0	0	0	1	1	0	14	8
Bacteria|Proteobacteria|Alphaproteobacteria|Rhodobacterales		0	0	1	0	0	0	0	0	0	0	0	0	0	0	1	0
Bacteria|Proteobacteria|Alphaproteobacteria|Rhodospirillales		0	0	0	1	1	32	0	0	0	0	0	0	0	0	0	0
Bacteria|Proteobacteria|Alphaproteobacteria|Rickettsiales		0	0	0	0	1	0	0	0	0	0	0	0	0	0	2	0
Bacteria|Proteobacteria|Alphaproteobacteria|Sphingomonadales		0	1	0	0	19	1	0	1	0	0	0	0	0	0	628	8
Bacteria|Proteobacteria|Betaproteobacteria|ASSO-13		0	0	0	0	0	0	0	0	0	0	0	0	0	0	0	0
Bacteria|Proteobacteria|Betaproteobacteria|Burkholderiales		102	718	601	549	1729	179	10	1132	951	267	106	58	1507	463	2670	355
Bacteria|Proteobacteria|Betaproteobacteria|Methylophilales		0	0	0	0	0	0	0	0	0	0	0	0	0	0	0	0
Bacteria|Proteobacteria|Betaproteobacteria|MND1		0	0	0	0	0	0	0	0	0	0	0	0	0	0	0	0
Bacteria|Proteobacteria|Betaproteobacteria|Neisseriales		8972	6253	13842	5320	2719	3105	1652	5639	10133	3435	4780	6706	2315	3922	6568	4860
Bacteria|Proteobacteria|Betaproteobacteria|Rhodocyclales		0	4	17	2	2	0	1	0	0	0	0	0	4	1	14	0
Bacteria|Proteobacteria|Betaproteobacteria|SC-I-84		0	0	0	0	0	0	0	0	0	0	0	0	0	0	0	0
Bacteria|Proteobacteria|Deltaproteobacteria|AF420338		0	0	0	0	0	0	0	0	0	0	0	0	0	0	0	0
Bacteria|Proteobacteria|Deltaproteobacteria|Bdellovibrionales		0	0	0	0	0	0	0	0	0	0	0	0	0	0	0	0
Bacteria|Proteobacteria|Deltaproteobacteria|Desulfobacterales		11	12	0	21	0	2	8	1	0	1	4	0	35	5	12	2
Bacteria|Proteobacteria|Deltaproteobacteria|Desulfovibrionales		0	5	0	13	61	0	74	0	5	2	59	0	11	18	0	3
Bacteria|Proteobacteria|Deltaproteobacteria|FAC87		0	0	0	0	0	0	0	0	0	0	0	0	0	0	0	0
Bacteria|Proteobacteria|Deltaproteobacteria|GMD14H09		0	7	0	4	1	0	0	0	0	0	0	0	0	0	0	0
Bacteria|Proteobacteria|Deltaproteobacteria|MIZ46		0	0	0	0	0	0	0	0	0	0	0	0	0	0	0	0
Bacteria|Proteobacteria|Deltaproteobacteria|Myxococcales		0	0	0	0	0	0	0	0	0	0	0	0	0	0	0	0
Bacteria|Proteobacteria|Deltaproteobacteria|Spirobacillales		0	0	0	0	0	0	0	0	0	0	0	0	0	0	0	0
Bacteria|Proteobacteria|Deltaproteobacteria|Syntrophobacterales		0	0	0	0	0	0	0	0	0	0	0	0	0	0	0	0
Bacteria|Proteobacteria|Epsilonproteobacteria|Campylobacterales		20	15	33	39	15	10	16	8	5	32	54	23	32	11	61	13
Bacteria|Proteobacteria|Gammaproteobacteria|Aeromonadales		0	0	0	47	4	3	1	0	0	0	0	0	0	0	0	1
Bacteria|Proteobacteria|Gammaproteobacteria|Alteromonadales		0	0	0	0	0	0	0	0	0	0	0	0	0	0	0	0
Bacteria|Proteobacteria|Gammaproteobacteria|Cardiobacteriales		19	122	273	228	80	29	8	314	105	35	5	14	147	89	480	102

Microbial Clade	Sample ID	C1.002	C1.003	C1.006	C1.009	C1.010	C1.012	C1.013	C1.014	C1.016	C1.017	C1.018	C1.019	C1.020	C1.021	C1.024	C1.025
	Group	Oral Ca	Oral Ca	Oral Ca	Oral Ca	Oral Ca	Oral Ca	Oral Ca	Oral Ca	Oral Ca	Oral Ca	Oral Ca	Oral Ca	Oral Ca	Oral Ca	Oral Ca	Oral Ca
	Body Site	Sa	Sa	Sa	Sa	Sa	Sa	Sa	Sa	Sa	Sa	Sa	Sa	Sa	Sa	Sa	Sa
Bacteria|Proteobacteria|Gammaproteobacteria|Enterobacteriales		0	3	0	34	8	56	3	0	0	1	7	0	14	0	49	11
Bacteria|Proteobacteria|Gammaproteobacteria|Legionellales		0	0	0	0	0	0	0	0	0	0	0	0	0	0	0	0
Bacteria|Proteobacteria|Gammaproteobacteria|Oceanospirillales		0	0	0	0	0	0	0	0	0	0	0	0	0	0	0	0
Bacteria|Proteobacteria|Gammaproteobacteria|Pasteurellales		686	568	1269	208	267	52	247	512	340	484	226	368	1836	374	307	113
Bacteria|Proteobacteria|Gammaproteobacteria|Pseudomonadales		16	5	83	6	74	0	0	1	4	0	24	3	5	0	96	31
Bacteria|Proteobacteria|Gammaproteobacteria|Salinisphaerales		0	0	0	0	0	0	0	0	0	0	0	0	0	0	0	0
Bacteria|Proteobacteria|Gammaproteobacteria|Vibrionales		0	0	0	0	0	0	0	0	0	0	0	0	0	0	0	0
Bacteria|Proteobacteria|Gammaproteobacteria|Xanthomonadales		0	0	1	2	0	2	0	0	0	0	2	0	0	0	10	1
Bacteria|Proteobacteria|TA18|CV90		0	0	0	0	0	0	0	0	0	0	0	0	0	0	0	0
Bacteria|Spirochaetes|GN05|SBYZ_6080		0	0	0	0	0	0	0	0	0	0	0	0	0	0	0	0
Bacteria|Spirochaetes|Spirochaetes|Sphaerochaetales		0	0	0	0	0	0	2	0	0	0	5	0	0	2	0	0
Bacteria|Spirochaetes|Spirochaetes|Spirochaetales		100	156	59	114	131	0	215	5	15	36	254	2	89	21	163	21
Bacteria|Synergistetes|Synergistia|Synergistales		22	43	15	51	167	2	77	4	6	3	108	0	24	15	70	11
Bacteria|Tenericutes|Mollicutes|Acholeplasmatales		0	0	0	6	0	0	0	0	0	1	0	0	0	0	0	0
Bacteria|Tenericutes|Mollicutes|Mycoplasmatales		4	0	0	1	26	0	4	1	0	0	29	0	1	0	34	8
Bacteria|Tenericutes|Mollicutes|RF39		0	0	0	6	1	0	0	0	0	0	1	2	0	0	4	3
Bacteria|Tenericutes|RF3|ML615J-28		0	1	0	0	6	0	2	0	0	0	3	0	0	0	6	0
Bacteria|Thermotogae|Thermotogae|Thermotogales		0	0	0	0	0	0	0	0	0	0	0	0	0	0	0	0
Bacteria|TM7|TM7-3|Blgi18		0	0	0	0	0	0	0	0	0	0	0	0	0	0	0	0
Bacteria|TM7|TM7-3|CW040		3	6	2	1	8	1	1	1	1	4	4	13	1	3	6	34
Bacteria|TM7|TM7-3|EW055		0	0	0	0	0	0	0	0	0	0	0	0	0	0	0	0
Bacteria|TM7|TM7-3|I025		5	15	13	55	147	15	105	59	1	31	242	0	24	17	191	30
Bacteria|Verrucomicrobia|Verruco-5|WCHB1-41		0	0	0	1	0	0	0	0	0	0	0	0	0	0	0	0
Bacteria|Verrucomicrobia|Verrucomicrobiae|Verrucomicrobiales		0	0	0	0	0	0	0	0	0	0	0	0	0	0	0	0
Bacteria|WS6|SC72|WCHB1-15		0	0	0	0	0	0	0	0	0	0	0	0	0	0	0	0
Bacteria|[Thermi]|Deinococci|Deinococcales|Deinococcaceae		0	0	0	0	0	0	0	0	0	0	0	0	0	0	0	0
Bacteria|[Thermi]|Deinococci|Deinococcales|Trueperaceae		0	0	0	0	0	0	0	0	0	0	0	0	0	0	0	0
Bacteria|[Thermi]|Deinococci|Thermales|Thermaceae		0	0	0	0	0	0	0	0	0	0	0	0	0	0	0	0
Bacteria|Acidobacteria|[Chloracidobacteria]|RB41|Ellin6075		0	0	0	0	0	0	0	0	0	0	0	0	0	0	0	0
Bacteria|Acidobacteria|Acidobacteria-6|iii1-15|mb2424		0	0	0	0	0	0	0	0	0	0	0	0	0	0	0	0
Bacteria|Acidobacteria|Acidobacteriia|Acidobacteriales|Acidobacte		0	0	0	0	0	0	0	0	0	0	0	0	0	0	0	0
Bacteria|Acidobacteria|Holophagae|Holophagales|Holophagaceae		0	0	0	0	0	0	0	0	0	0	0	0	0	0	0	0
Bacteria|Actinobacteria|Acidimicrobiia|Acidimicrobiales|C111		0	0	0	0	0	0	0	0	0	0	0	0	0	0	0	0
Bacteria|Actinobacteria|Actinobacteria|Actinomycetales|ACK-M1		0	0	0	0	0	0	0	0	0	0	0	0	0	0	0	0
Bacteria|Actinobacteria|Actinobacteria|Actinomycetales|Actinomy		978	939	258	963	92	220	2084	3600	421	1210	536	1076	2687	1739	405	242
Bacteria|Actinobacteria|Actinobacteria|Actinomycetales|Bogoriella		0	0	0	0	0	0	0	0	0	0	0	0	0	0	0	0
Bacteria|Actinobacteria|Actinobacteria|Actinomycetales|Brevibacte		0	0	0	0	0	0	0	0	0	0	0	0	0	0	0	0

Microbial Clade	Sample ID	C1.002	C1.003	C1.006	C1.009	C1.010	C1.012	C1.013	C1.014	C1.016	C1.017	C1.018	C1.019	C1.020	C1.021	C1.024	C1.025
	Group	Oral Ca	Oral Ca	Oral Ca	Oral Ca	Oral Ca	Oral Ca	Oral Ca	Oral Ca	Oral Ca	Oral Ca	Oral Ca	Oral Ca	Oral Ca	Oral Ca	Oral Ca	Oral Ca
	Body Site	Sa	Sa	Sa	Sa	Sa	Sa	Sa	Sa	Sa	Sa	Sa	Sa	Sa	Sa	Sa	Sa
Bacteria|Actinobacteria|Actinobacteria|Actinomycetales|Corynebac		60	249	612	94	135	63	221	160	208	37	51	523	10	86	259	1
Bacteria|Actinobacteria|Actinobacteria|Actinomycetales|Dermabac		0	0	0	1	0	0	0	0	0	0	0	0	0	0	0	0
Bacteria|Actinobacteria|Actinobacteria|Actinomycetales|Dermacoc		0	0	0	0	0	0	0	0	0	0	0	0	0	0	0	0
Bacteria|Actinobacteria|Actinobacteria|Actinomycetales|Dermatopl		0	0	0	0	0	0	0	0	0	0	0	0	0	0	0	0
Bacteria|Actinobacteria|Actinobacteria|Actinomycetales|Dietziacea		0	0	0	0	0	0	0	0	0	0	0	0	0	0	0	0
Bacteria|Actinobacteria|Actinobacteria|Actinomycetales|Frankiacea		0	0	0	0	0	0	0	0	0	0	0	0	0	0	0	0
Bacteria|Actinobacteria|Actinobacteria|Actinomycetales|Geoderma		0	0	0	0	0	0	0	0	0	0	0	0	0	0	0	0
Bacteria|Actinobacteria|Actinobacteria|Actinomycetales|Gordoniac		0	0	0	0	0	0	0	0	0	0	0	0	0	0	0	0
Bacteria|Actinobacteria|Actinobacteria|Actinomycetales|Intraspora		0	0	0	0	0	0	0	0	0	0	0	0	0	0	0	0
Bacteria|Actinobacteria|Actinobacteria|Actinomycetales|Jonesiacea		0	0	0	0	0	0	0	0	0	0	0	0	0	0	0	0
Bacteria|Actinobacteria|Actinobacteria|Actinomycetales|Kineospor		0	0	0	0	0	0	0	0	0	0	0	0	0	0	0	0
Bacteria|Actinobacteria|Actinobacteria|Actinomycetales|Microbact		0	0	0	1	1	1	0	0	0	1	2	0	0	0	1	0
Bacteria|Actinobacteria|Actinobacteria|Actinomycetales|Micrococc		1213	1324	1084	4179	158	226	4097	881	940	1955	3136	4891	4829	1376	183	662
Bacteria|Actinobacteria|Actinobacteria|Actinomycetales|Mycobact		0	0	0	0	0	0	0	0	0	0	0	0	0	0	0	0
Bacteria|Actinobacteria|Actinobacteria|Actinomycetales|Nakamure		0	0	0	0	0	0	0	0	0	0	0	0	0	0	0	0
Bacteria|Actinobacteria|Actinobacteria|Actinomycetales|Nocardiac		0	0	0	0	0	0	0	0	0	0	0	0	0	0	1	0
Bacteria|Actinobacteria|Actinobacteria|Actinomycetales|Nocardioi		0	0	0	2	0	0	0	0	0	0	0	0	0	0	0	0
Bacteria|Actinobacteria|Actinobacteria|Actinomycetales|Nocardiop		0	0	0	0	0	0	0	0	0	0	0	0	0	0	0	0
Bacteria|Actinobacteria|Actinobacteria|Actinomycetales|Promicron		0	0	0	0	0	0	0	0	0	0	0	0	0	0	0	0
Bacteria|Actinobacteria|Actinobacteria|Actinomycetales|Propioniba		18	274	185	67	14	13	10	176	136	6	32	19	279	62	14	2
Bacteria|Actinobacteria|Actinobacteria|Actinomycetales|Pseudonoc		0	0	0	0	0	0	0	0	0	0	0	0	0	0	0	0
Bacteria|Actinobacteria|Actinobacteria|Actinomycetales|Sporichthy		0	0	0	0	0	0	0	0	0	0	0	0	0	0	0	0
Bacteria|Actinobacteria|Actinobacteria|Actinomycetales|Streptomy		0	0	0	0	0	0	0	0	0	0	0	0	0	0	0	0
Bacteria|Actinobacteria|Actinobacteria|Actinomycetales|Tsukamure		0	0	0	0	0	0	0	0	0	0	0	0	0	0	0	0
Bacteria|Actinobacteria|Actinobacteria|Actinomycetales|Williamsia		0	0	0	0	0	0	0	0	0	0	0	0	0	0	0	0
Bacteria|Actinobacteria|Actinobacteria|Actinomycetales|Yaniellace		0	0	0	0	0	0	0	0	0	0	0	0	0	0	0	0
Bacteria|Actinobacteria|Actinobacteria|Bifidobacteriales|Bifidobac		6	1	0	139	102	30	301	7	3	11	20	11	59	3	120	23
Bacteria|Actinobacteria|Coriobacteriia|Coriobacteriales|Coriobacte		123	72	34	28	25	11	186	354	33	322	189	123	218	281	20	28
Bacteria|Actinobacteria|Nitriliruptoria|Nitriliruptorales|Nitrilirupto		0	0	0	0	0	0	0	0	0	0	0	0	0	0	0	0
Bacteria|Actinobacteria|Rubrobacteria|Rubrobacterales|Rubrobacte		0	0	0	0	0	0	0	0	0	0	0	0	0	0	0	0
Bacteria|Actinobacteria|Thermoleophilia|Solirubrobacterales|Patul		0	0	0	0	0	0	0	0	0	0	0	0	0	0	0	0
Bacteria|Actinobacteria|Thermoleophilia|Solirubrobacterales|Soliru		0	0	0	0	0	0	0	0	0	0	0	0	0	0	0	0
Bacteria|Armatimonadetes|[Fimbriimonadia]|[Fimbriimonadales]|[		0	0	0	0	0	1	0	0	0	0	0	0	0	0	0	0
Bacteria|Armatimonadetes|Armatimonadia|Armatimonadales|Arma		0	0	0	0	0	0	0	0	0	0	0	0	0	0	0	0
Bacteria|Bacteroidetes|[Saprospirae]|[Saprospirales]|Chitinophaga		0	0	0	1	1	0	0	0	0	0	0	1	0	0	1	0
Bacteria|Bacteroidetes|[Saprospirae]|[Saprospirales]|Saprospiracea		0	0	0	0	0	0	0	0	0	0	0	0	0	0	0	0
Bacteria|Bacteroidetes|Bacteroidia|Bacteroidales|[Barnesiellaceae]		0	0	0	0	0	0	0	0	0	0	0	0	0	0	0	0

Microbial Clade	Sample ID<br>Group<br>Body Site	C1.002<br>Oral Ca<br>Sa	C1.003<br>Oral Ca<br>Sa	C1.006<br>Oral Ca<br>Sa	C1.009<br>Oral Ca<br>Sa	C1.010<br>Oral Ca<br>Sa	C1.012<br>Oral Ca<br>Sa	C1.013<br>Oral Ca<br>Sa	C1.014<br>Oral Ca<br>Sa	C1.016<br>Oral Ca<br>Sa	C1.017<br>Oral Ca<br>Sa	C1.018<br>Oral Ca<br>Sa	C1.019<br>Oral Ca<br>Sa	C1.020<br>Oral Ca<br>Sa	C1.021<br>Oral Ca<br>Sa	C1.024<br>Oral Ca<br>Sa	C1.025<br>Oral Ca<br>Sa
Bacteria|Bacteroidetes|Bacteroidia|Bacteroidales|[Odoribacteracea		0	0	0	9	0	0	0	0	0	0	0	0	0	0	0	0
Bacteria|Bacteroidetes|Bacteroidia|Bacteroidales|[Paraprevotellace		2118	2306	1522	2972	1223	641	940	4152	2050	2582	1839	2478	3792	2645	1311	1826
Bacteria|Bacteroidetes|Bacteroidia|Bacteroidales|Bacteroidaceae		5	2	0	346	6	1	20	1	2	0	77	0	8	5	2	5
Bacteria|Bacteroidetes|Bacteroidia|Bacteroidales|BS11		14	16	0	2	0	0	72	0	0	1	0	0	0	0	0	0
Bacteria|Bacteroidetes|Bacteroidia|Bacteroidales|p-2534-18B5		0	0	0	4	1	1	0	0	0	0	0	0	0	0	0	0
Bacteria|Bacteroidetes|Bacteroidia|Bacteroidales|Porphyromonada		1185	4446	3268	2796	1664	1172	1478	288	1609	213	1419	819	892	1537	4110	1504
Bacteria|Bacteroidetes|Bacteroidia|Bacteroidales|Prevotellaceae		792	435	522	475	407	166	1403	545	190	981	2457	419	2059	785	704	331
Bacteria|Bacteroidetes|Bacteroidia|Bacteroidales|RF16		0	0	0	1	0	0	1	0	0	0	0	0	0	0	0	0
Bacteria|Bacteroidetes|Bacteroidia|Bacteroidales|Rikenellaceae		0	0	0	33	0	0	0	0	0	1	0	0	0	0	2	0
Bacteria|Bacteroidetes|Bacteroidia|Bacteroidales|S24-7		0	0	1	10	2	2	1	0	0	0	1	0	0	0	4	0
Bacteria|Bacteroidetes|Cytophagia|Cytophagales|Cyclobacteriacea		0	0	0	0	0	0	0	0	0	0	0	0	0	0	0	0
Bacteria|Bacteroidetes|Cytophagia|Cytophagales|Cytophagaceae		0	0	0	0	0	0	0	0	0	0	0	0	0	0	0	0
Bacteria|Bacteroidetes|Flavobacteriia|Flavobacteriales|[Weeksellac		5	2	15	1	80	1	1	2	0	1	2	1	1	4	93	10
Bacteria|Bacteroidetes|Flavobacteriia|Flavobacteriales|Cryomorpha		0	0	0	0	0	0	0	0	0	0	0	0	0	0	0	0
Bacteria|Bacteroidetes|Flavobacteriia|Flavobacteriales|Flavobacter		51	41	158	130	101	63	79	58	23	22	44	19	45	40	260	36
Bacteria|Bacteroidetes|Sphingobacteriia|Sphingobacteriales|Sphing		0	0	0	0	0	0	0	0	0	0	3	0	0	0	0	7
Bacteria|Chlamydiae|Chlamydiia|Chlamydiales|Rhabdochlamydiac		0	0	0	0	0	0	0	0	0	0	0	0	0	0	0	0
Bacteria|Chlamydiae|Chlamydiia|Chlamydiales|Waddliaceae		0	0	0	0	0	0	0	0	0	0	0	0	0	0	0	0
Bacteria|Chloroflexi|Anaerolineae|Anaerolineales|Anaerolinaceae		0	0	0	0	1	0	0	0	0	0	0	0	0	0	0	0
Bacteria|Chloroflexi|Anaerolineae|Caldilineales|Caldilineaceae		0	0	0	0	0	0	0	0	0	0	0	0	0	0	0	0
Bacteria|Chloroflexi|Anaerolineae|SBR1031|A4b		0	0	0	0	0	0	0	0	0	0	0	0	0	0	0	0
Bacteria|Cyanobacteria|Oscillatoriophycideae|Chroococcales|Xeno		0	0	0	0	0	0	0	0	0	0	0	0	0	0	0	0
Bacteria|Cyanobacteria|Oscillatoriophycideae|Oscillatoriales|Phorr		0	0	0	0	0	0	0	0	0	0	0	0	0	0	0	0
Bacteria|Fibrobacteres|Fibrobacteria|Fibrobacterales|Fibrobacterac		0	0	0	2	0	0	0	0	0	0	0	0	0	0	0	0
Bacteria|Firmicutes|Bacilli|Bacillales|[Exiguobacteraceae]		0	0	0	0	0	0	0	0	0	0	0	0	0	0	0	0
Bacteria|Firmicutes|Bacilli|Bacillales|[Thermicanaceae]		0	0	0	0	0	0	0	0	0	0	0	0	0	0	0	0
Bacteria|Firmicutes|Bacilli|Bacillales|Alicyclobacillaceae		0	0	0	0	0	0	0	0	0	0	0	0	0	0	0	0
Bacteria|Firmicutes|Bacilli|Bacillales|Bacillaceae		0	0	0	0	0	5	0	0	0	0	0	0	0	0	3	4
Bacteria|Firmicutes|Bacilli|Bacillales|Listeriaceae		0	0	0	0	0	0	0	0	0	0	0	0	0	0	0	0
Bacteria|Firmicutes|Bacilli|Bacillales|Paenibacillaceae		0	0	0	0	0	0	0	0	0	0	0	0	0	0	1	0
Bacteria|Firmicutes|Bacilli|Bacillales|Planococcaceae		0	0	0	0	1	0	0	0	0	0	0	0	0	0	0	1
Bacteria|Firmicutes|Bacilli|Bacillales|Sporolactobacillaceae		0	0	0	0	0	0	0	0	0	0	0	0	0	0	0	0
Bacteria|Firmicutes|Bacilli|Bacillales|Staphylococcaceae		0	0	0	0	0	0	0	0	0	0	1	0	0	0	2	1
Bacteria|Firmicutes|Bacilli|Bacillales|Thermoactinomycetaceae		0	0	0	0	0	0	0	0	0	0	0	0	0	0	0	0
Bacteria|Firmicutes|Bacilli|Gemellales|Gemellaceae		4	12	5	12	5	8	13	1	0	4	28	5	10	4	49	2
Bacteria|Firmicutes|Bacilli|Lactobacillales|Aerococcaceae		5	28	24	9	100	6	3	37	4	0	43	5	13	0	61	12
Bacteria|Firmicutes|Bacilli|Lactobacillales|Carnobacteriaceae		88	25	103	96	17	36	180	28	30	112	127	172	267	73	56	29

	Sample ID	C1.002	C1.003	C1.006	C1.009	C1.010	C1.012	C1.013	C1.014	C1.016	C1.017	C1.018	C1.019	C1.020	C1.021	C1.024	C1.025
Microbial Clade	Group	Oral Ca	Oral Ca	Oral Ca	Oral Ca	Oral Ca	Oral Ca	Oral Ca	Oral Ca	Oral Ca	Oral Ca	Oral Ca	Oral Ca	Oral Ca	Oral Ca	Oral Ca	Oral Ca
	Body Site	Sa	Sa	Sa	Sa	Sa	Sa	Sa	Sa	Sa	Sa	Sa	Sa	Sa	Sa	Sa	Sa
Bacteria|Firmicutes|Bacilli|Lactobacillales|Enterococcaceae		0	0	0	1	0	0	0	0	0	0	0	0	0	0	0	0
Bacteria|Firmicutes|Bacilli|Lactobacillales|Lactobacillaceae		0	0	0	59	31	58	22	1	0	0	3	2	0	0	398	55
Bacteria|Firmicutes|Bacilli|Lactobacillales|Leuconostocaceae		0	0	0	0	0	0	0	0	0	0	0	0	0	0	0	0
Bacteria|Firmicutes|Bacilli|Lactobacillales|Streptococcaceae		627	578	389	633	138	173	1214	505	173	662	810	678	1105	340	424	272
Bacteria|Firmicutes|Bacilli|Turicibacterales|Turicibacteraceae		0	0	0	2	0	0	0	0	0	0	0	0	0	0	0	0
Bacteria|Firmicutes|Clostridia|Clostridiales|[Acidaminobacteraceae		0	0	0	0	0	0	0	0	0	0	0	0	0	0	0	0
Bacteria|Firmicutes|Clostridia|Clostridiales|[Mogibacteriaceae]		57	28	17	32	29	9	44	17	6	71	58	56	106	54	40	31
Bacteria|Firmicutes|Clostridia|Clostridiales|[Tissierellaceae]		4	9	5	1	35	2	4	0	0	0	19	0	0	1	28	24
Bacteria|Firmicutes|Clostridia|Clostridiales|Christensenellaceae		0	0	0	4	1	0	0	0	1	0	0	1	1	0	0	0
Bacteria|Firmicutes|Clostridia|Clostridiales|Clostridiaceae		0	0	0	6	3	2	1	0	0	0	1	0	0	0	1	2
Bacteria|Firmicutes|Clostridia|Clostridiales|Dehalobacteriaceae		0	0	0	0	0	0	0	0	0	0	0	0	0	0	0	0
Bacteria|Firmicutes|Clostridia|Clostridiales|Eubacteriaceae		0	1	0	1	0	0	16	1	0	1	1	0	0	0	1	0
Bacteria|Firmicutes|Clostridia|Clostridiales|Lachnospiraceae		175	106	116	452	79	46	350	144	94	176	277	287	636	112	155	55
Bacteria|Firmicutes|Clostridia|Clostridiales|Peptococcaceae		4	3	4	4	2	0	2	0	2	0	4	4	4	0	5	0
Bacteria|Firmicutes|Clostridia|Clostridiales|Peptostreptococcaceae		8	11	12	18	32	4	12	9	5	2	35	10	2	3	43	13
Bacteria|Firmicutes|Clostridia|Clostridiales|Ruminococcaceae		3	0	2	471	29	8	2	3	1	1	1	1	3	0	3	2
Bacteria|Firmicutes|Clostridia|Clostridiales|Syntrophomonadaceae		1	0	0	0	0	0	0	0	0	0	2	0	0	0	0	0
Bacteria|Firmicutes|Clostridia|Clostridiales|Veillonellaceae		286	232	142	273	176	61	998	438	89	607	1100	239	292	340	554	93
Bacteria|Firmicutes|Clostridia|Thermoanaerobacterales|Thermoana		0	0	0	0	0	0	0	0	0	0	0	0	0	0	0	0
Bacteria|Firmicutes|Erysipelotrichi|Erysipelotrichales|Erysipelotric		46	30	13	72	12	4	43	79	4	70	118	83	67	23	29	121
Bacteria|Fusobacteria|Fusobacteriia|Fusobacteriales|Fusobacteriace		27	54	28	49	56	37	28	17	7	12	215	18	44	8	115	74
Bacteria|Fusobacteria|Fusobacteriia|Fusobacteriales|Leptotrichiace		5	22	30	24	57	7	12	10	2	15	58	14	5	5	136	25
Bacteria|Lentisphaerae|[Lentisphaeria]|Z20|R4-45B		0	0	0	1	0	0	0	0	0	0	0	0	0	0	0	0
Bacteria|Planctomycetes|Planctomycetia|Gemmatales|Gemmatacea		0	0	0	0	0	0	0	0	0	0	0	0	0	0	0	0
Bacteria|Proteobacteria|Alphaproteobacteria|Caulobacterales|Caulc		0	1	0	1	4	3	1	0	1	0	1	0	2	0	14	1
Bacteria|Proteobacteria|Alphaproteobacteria|Rhizobiales|Aurantim		0	0	0	0	0	0	0	0	0	0	0	0	0	0	0	0
Bacteria|Proteobacteria|Alphaproteobacteria|Rhizobiales|Bartonell		0	0	0	0	0	0	0	0	0	0	0	0	0	0	0	0
Bacteria|Proteobacteria|Alphaproteobacteria|Rhizobiales|Beijerincl		0	0	0	0	0	0	0	0	0	0	0	0	0	0	0	0
Bacteria|Proteobacteria|Alphaproteobacteria|Rhizobiales|Bradyrhiz		0	1	0	0	0	0	0	0	0	0	0	0	1	0	2	0
Bacteria|Proteobacteria|Alphaproteobacteria|Rhizobiales|Brucellac		0	0	0	1	2	1	0	0	0	0	0	0	0	0	5	0
Bacteria|Proteobacteria|Alphaproteobacteria|Rhizobiales|Hyphomi		0	0	1	0	0	0	0	0	0	0	0	0	0	0	3	3
Bacteria|Proteobacteria|Alphaproteobacteria|Rhizobiales|Methylob		0	0	0	0	1	0	0	0	0	0	0	1	0	0	3	0
Bacteria|Proteobacteria|Alphaproteobacteria|Rhizobiales|Methyloc		0	0	0	0	0	0	0	0	0	0	0	0	0	0	0	0
Bacteria|Proteobacteria|Alphaproteobacteria|Rhizobiales|Phyllobac		0	0	0	0	0	0	0	0	0	0	0	0	0	0	0	3
Bacteria|Proteobacteria|Alphaproteobacteria|Rhizobiales|Rhizobiac		0	0	0	0	0	0	0	0	0	0	0	0	0	0	0	2
Bacteria|Proteobacteria|Alphaproteobacteria|Rhizobiales|Xanthoba		0	0	0	0	0	0	0	0	0	0	0	0	0	0	0	0
Bacteria|Proteobacteria|Alphaproteobacteria|Rhodobacterales|Hypl		0	0	0	0	0	0	0	0	0	0	0	0	0	0	0	0

Microbial Clade	Sample ID	C1.002	C1.003	C1.006	C1.009	C1.010	C1.012	C1.013	C1.014	C1.016	C1.017	C1.018	C1.019	C1.020	C1.021	C1.024	C1.025
	Group	Oral Ca	Oral Ca	Oral Ca	Oral Ca	Oral Ca	Oral Ca	Oral Ca	Oral Ca	Oral Ca	Oral Ca	Oral Ca	Oral Ca	Oral Ca	Oral Ca	Oral Ca	Oral Ca
	Body Site	Sa	Sa	Sa	Sa	Sa	Sa	Sa	Sa	Sa	Sa	Sa	Sa	Sa	Sa	Sa	Sa
Bacteria|Proteobacteria|Alphaproteobacteria|Rhodobacterales|Rhod		0	0	1	0	0	0	0	0	0	0	0	0	0	0	1	0
Bacteria|Proteobacteria|Alphaproteobacteria|Rhodospirillales|Acet		0	0	0	0	1	32	0	0	0	0	0	0	0	0	0	0
Bacteria|Proteobacteria|Alphaproteobacteria|Rhodospirillales|Rhod		0	0	0	1	0	0	0	0	0	0	0	0	0	0	0	0
Bacteria|Proteobacteria|Alphaproteobacteria|Rickettsiales|mitocho		0	0	0	0	0	0	0	0	0	0	0	0	0	0	0	0
Bacteria|Proteobacteria|Alphaproteobacteria|Rickettsiales|Pelagiba		0	0	0	0	0	0	0	0	0	0	0	0	0	0	0	0
Bacteria|Proteobacteria|Alphaproteobacteria|Rickettsiales|Rickettsi		0	0	0	0	0	0	0	0	0	0	0	0	0	0	0	0
Bacteria|Proteobacteria|Alphaproteobacteria|Sphingomonadales|Er		0	0	0	0	1	0	0	0	0	0	0	0	0	0	0	0
Bacteria|Proteobacteria|Alphaproteobacteria|Sphingomonadales|Sp		0	1	0	0	18	1	0	1	0	0	0	0	0	0	628	8
Bacteria|Proteobacteria|Betaproteobacteria|Burkholderiales|Alcalig		0	0	0	32	0	1	0	0	0	0	0	0	0	0	2	0
Bacteria|Proteobacteria|Betaproteobacteria|Burkholderiales|Burkho		79	521	411	362	292	81	7	1088	572	122	80	48	1264	243	1613	287
Bacteria|Proteobacteria|Betaproteobacteria|Burkholderiales|Coman		23	196	190	153	1435	96	3	44	379	144	26	10	242	220	1051	66
Bacteria|Proteobacteria|Betaproteobacteria|Burkholderiales|Oxalob		0	0	0	1	2	0	0	0	0	0	0	0	1	0	4	1
Bacteria|Proteobacteria|Betaproteobacteria|Methylophilales|Methy		0	0	0	0	0	0	0	0	0	0	0	0	0	0	0	0
Bacteria|Proteobacteria|Betaproteobacteria|Neisseriales|Neisseriace		8972	6253	13842	5320	2719	3105	1652	5639	10133	3435	4780	6706	2315	3922	6568	4860
Bacteria|Proteobacteria|Betaproteobacteria|Rhodocyclales|Rhodocy		0	4	17	2	2	0	1	0	0	0	0	0	4	1	14	0
Bacteria|Proteobacteria|Deltaproteobacteria|Bdellovibrionales|Bact		0	0	0	0	0	0	0	0	0	0	0	0	0	0	0	0
Bacteria|Proteobacteria|Deltaproteobacteria|Bdellovibrionales|Bde		0	0	0	0	0	0	0	0	0	0	0	0	0	0	0	0
Bacteria|Proteobacteria|Deltaproteobacteria|Desulfobacterales|Desu		11	12	0	21	0	2	8	1	0	1	4	0	35	5	12	2
Bacteria|Proteobacteria|Deltaproteobacteria|Desulfovibrionales|De		0	0	0	0	0	0	0	0	0	2	21	0	0	0	0	0
Bacteria|Proteobacteria|Deltaproteobacteria|Desulfovibrionales|De		0	5	0	13	61	0	74	0	5	0	38	0	11	18	0	3
Bacteria|Proteobacteria|Deltaproteobacteria|Myxococcales|0319-6(		0	0	0	0	0	0	0	0	0	0	0	0	0	0	0	0
Bacteria|Proteobacteria|Deltaproteobacteria|Myxococcales|Haliang		0	0	0	0	0	0	0	0	0	0	0	0	0	0	0	0
Bacteria|Proteobacteria|Deltaproteobacteria|Myxococcales|Myxoco		0	0	0	0	0	0	0	0	0	0	0	0	0	0	0	0
Bacteria|Proteobacteria|Deltaproteobacteria|Syntrophobacterales|Sy		0	0	0	0	0	0	0	0	0	0	0	0	0	0	0	0
Bacteria|Proteobacteria|Epsilonproteobacteria|Campylobacterales|C		20	15	33	29	15	10	16	8	5	31	54	23	32	11	61	12
Bacteria|Proteobacteria|Epsilonproteobacteria|Campylobacterales|I		0	0	0	10	0	0	0	0	0	1	0	0	0	0	0	1
Bacteria|Proteobacteria|Gammaproteobacteria|Aeromonadales|Aero		0	0	0	0	0	1	0	0	0	0	0	0	0	0	0	1
Bacteria|Proteobacteria|Gammaproteobacteria|Aeromonadales|Succ		0	0	0	47	4	2	1	0	0	0	0	0	0	0	0	0
Bacteria|Proteobacteria|Gammaproteobacteria|Alteromonadales|[C		0	0	0	0	0	0	0	0	0	0	0	0	0	0	0	0
Bacteria|Proteobacteria|Gammaproteobacteria|Alteromonadales|Al		0	0	0	0	0	0	0	0	0	0	0	0	0	0	0	0
Bacteria|Proteobacteria|Gammaproteobacteria|Alteromonadales|Sh		0	0	0	0	0	0	0	0	0	0	0	0	0	0	0	0
Bacteria|Proteobacteria|Gammaproteobacteria|Cardiobacteriales|Ca		19	122	273	224	80	29	8	314	102	35	5	14	147	86	479	94
Bacteria|Proteobacteria|Gammaproteobacteria|Enterobacteriales|En		0	3	0	34	8	56	3	0	0	1	7	0	14	0	49	11
Bacteria|Proteobacteria|Gammaproteobacteria|Legionellales|Coxiel		0	0	0	0	0	0	0	0	0	0	0	0	0	0	0	0
Bacteria|Proteobacteria|Gammaproteobacteria|Legionellales|Legion		0	0	0	0	0	0	0	0	0	0	0	0	0	0	0	0
Bacteria|Proteobacteria|Gammaproteobacteria|Oceanospirillales|Al		0	0	0	0	0	0	0	0	0	0	0	0	0	0	0	0
Bacteria|Proteobacteria|Gammaproteobacteria|Oceanospirillales|Ha		0	0	0	0	0	0	0	0	0	0	0	0	0	0	0	0

Microbial Clade	Sample ID / Group / Body Site	C1.002	C1.003	C1.006	C1.009	C1.010	C1.012	C1.013	C1.014	C1.016	C1.017	C1.018	C1.019	C1.020	C1.021	C1.024	C1.025
	Group	Oral Ca	Oral Ca	Oral Ca	Oral Ca	Oral Ca	Oral Ca	Oral Ca	Oral Ca	Oral Ca	Oral Ca	Oral Ca	Oral Ca	Oral Ca	Oral Ca	Oral Ca	Oral Ca
	Body Site	Sa	Sa	Sa	Sa	Sa	Sa	Sa	Sa	Sa	Sa	Sa	Sa	Sa	Sa	Sa	Sa
Bacteria|Proteobacteria|Gammaproteobacteria|Pasteurellales|Pasteu		686	568	1269	208	267	52	247	512	340	484	226	368	1836	374	307	113
Bacteria|Proteobacteria|Gammaproteobacteria|Pseudomonadales|M		16	5	83	6	71	0	0	0	3	0	24	3	5	0	75	24
Bacteria|Proteobacteria|Gammaproteobacteria|Pseudomonadales|Ps		0	0	0	0	3	0	0	1	1	0	0	0	0	0	21	7
Bacteria|Proteobacteria|Gammaproteobacteria|Salinisphaerales|Sal		0	0	0	0	0	0	0	0	0	0	0	0	0	0	0	0
Bacteria|Proteobacteria|Gammaproteobacteria|Vibrionales|Pseudoa		0	0	0	0	0	0	0	0	0	0	0	0	0	0	0	0
Bacteria|Proteobacteria|Gammaproteobacteria|Vibrionales|Vibrion		0	0	0	0	0	0	0	0	0	0	0	0	0	0	0	0
Bacteria|Proteobacteria|Gammaproteobacteria|Xanthomonadales|S		0	0	0	1	0	0	0	0	0	0	0	0	0	0	0	0
Bacteria|Proteobacteria|Gammaproteobacteria|Xanthomonadales|X		0	0	1	1	0	2	0	0	0	0	2	0	0	0	10	1
Bacteria|Spirochaetes|Spirochaetes|Sphaerochaetales|Sphaerochaet		0	0	0	0	0	0	2	0	0	0	5	0	0	2	0	0
Bacteria|Spirochaetes|Spirochaetes|Spirochaetales|Spirochaetaceae		100	156	59	114	131	0	215	5	15	36	254	2	89	21	163	21
Bacteria|Synergistetes|Synergistia|Synergistales|Dethiosulfovibrior		22	43	15	51	167	2	77	4	6	3	108	0	24	15	70	11
Bacteria|Synergistetes|Synergistia|Synergistales|Synergistaceae		0	0	0	0	0	0	0	0	0	0	0	0	0	0	0	0
Bacteria|Tenericutes|Mollicutes|Acholeplasmatales|Acholeplasmat		0	0	0	6	0	0	0	0	0	1	0	0	0	0	0	0
Bacteria|Tenericutes|Mollicutes|Mycoplasmatales|Mycoplasmatace		4	0	0	1	26	0	4	1	0	0	29	0	1	0	34	8
Bacteria|Thermotogae|Thermotogae|Thermotogales|Thermotogacea		0	0	0	0	0	0	0	0	0	0	0	0	0	0	0	0
Bacteria|TM7|TM7-3|CW040|F16		1	2	1	0	1	1	1	1	0	4	3	4	1	2	0	0
Bacteria|TM7|TM7-3|I025|Rs-045		5	15	13	55	147	15	105	59	1	31	242	0	24	17	191	30
Bacteria|Verrucomicrobia|Verruco-5|WCHB1-41|RFP12		0	0	0	1	0	0	0	0	0	0	0	0	0	0	0	0
Bacteria|Verrucomicrobia|Verrucomicrobiae|Verrucomicrobiales|V		0	0	0	0	0	0	0	0	0	0	0	0	0	0	0	0
Bacteria|[Thermi]|Deinococci|Deinococcales|Deinococcaceae|Dein		0	0	0	0	0	0	0	0	0	0	0	0	0	0	0	0
Bacteria|[Thermi]|Deinococci|Deinococcales|Trueperaceae|Trueper		0	0	0	0	0	0	0	0	0	0	0	0	0	0	0	0
Bacteria|[Thermi]|Deinococci|Thermales|Thermaceae|Thermus		0	0	0	0	0	0	0	0	0	0	0	0	0	0	0	0
Bacteria|Acidobacteria|[Chloracidobacteria]|RB41|Ellin6075_uncl		0	0	0	0	0	0	0	0	0	0	0	0	0	0	0	0
Bacteria|Acidobacteria|Acidobacteria-6|iii1-15|mb2424_unclassifie		0	0	0	0	0	0	0	0	0	0	0	0	0	0	0	0
Bacteria|Acidobacteria|Acidobacteriia|Acidobacteriales|Acidobacte		0	0	0	0	0	0	0	0	0	0	0	0	0	0	0	0
Bacteria|Acidobacteria|Holophagae|Holophagales|Holophagaceae|(		0	0	0	0	0	0	0	0	0	0	0	0	0	0	0	0
Bacteria|Actinobacteria|Acidimicrobiia|Acidimicrobiales|C111_un		0	0	0	0	0	0	0	0	0	0	0	0	0	0	0	0
Bacteria|Actinobacteria|Actinobacteria|Actinomycetales|ACK-M1_		0	0	0	0	0	0	0	0	0	0	0	0	0	0	0	0
Bacteria|Actinobacteria|Actinobacteria|Actinomycetales|Actinomy		970	843	256	953	92	215	2077	3569	412	1205	528	1066	2614	1736	394	240
Bacteria|Actinobacteria|Actinobacteria|Actinomycetales|Actinomy		2	0	0	0	0	0	5	2	1	0	0	2	0	0	0	1
Bacteria|Actinobacteria|Actinobacteria|Actinomycetales|Actinomy		0	0	0	0	0	0	0	0	0	0	0	0	0	0	0	0
Bacteria|Actinobacteria|Actinobacteria|Actinomycetales|Actinomy		0	0	0	0	0	0	0	0	0	0	0	0	0	0	0	0
Bacteria|Actinobacteria|Actinobacteria|Actinomycetales|Actinomy		6	96	2	10	0	5	2	29	8	5	8	8	73	3	11	1
Bacteria|Actinobacteria|Actinobacteria|Actinomycetales|Bogoriella		0	0	0	0	0	0	0	0	0	0	0	0	0	0	0	0
Bacteria|Actinobacteria|Actinobacteria|Actinomycetales|Brevibacte		0	0	0	0	0	0	0	0	0	0	0	0	0	0	0	0
Bacteria|Actinobacteria|Actinobacteria|Actinomycetales|Corynebac		60	249	612	94	135	63	221	160	208	37	51	523	10	86	259	1
Bacteria|Actinobacteria|Actinobacteria|Actinomycetales|Dermabac		0	0	0	1	0	0	0	0	0	0	0	0	0	0	0	0

Microbial Clade		C1.002	C1.003	C1.006	C1.009	C1.010	C1.012	C1.013	C1.014	C1.016	C1.017	C1.018	C1.019	C1.020	C1.021	C1.024	C1.025
	Sample ID	C1.002	C1.003	C1.006	C1.009	C1.010	C1.012	C1.013	C1.014	C1.016	C1.017	C1.018	C1.019	C1.020	C1.021	C1.024	C1.025
	Group	Oral Ca	Oral Ca	Oral Ca	Oral Ca	Oral Ca	Oral Ca	Oral Ca	Oral Ca	Oral Ca	Oral Ca	Oral Ca	Oral Ca	Oral Ca	Oral Ca	Oral Ca	Oral Ca
	Body Site	Sa	Sa	Sa	Sa	Sa	Sa	Sa	Sa	Sa	Sa	Sa	Sa	Sa	Sa	Sa	Sa
Bacteria|Actinobacteria|Actinobacteria|Actinomycetales|Dermacoc		0	0	0	0	0	0	0	0	0	0	0	0	0	0	0	0
Bacteria|Actinobacteria|Actinobacteria|Actinomycetales|Dermatopl		0	0	0	0	0	0	0	0	0	0	0	0	0	0	0	0
Bacteria|Actinobacteria|Actinobacteria|Actinomycetales|Dietziacea		0	0	0	0	0	0	0	0	0	0	0	0	0	0	0	0
Bacteria|Actinobacteria|Actinobacteria|Actinomycetales|Frankiacea		0	0	0	0	0	0	0	0	0	0	0	0	0	0	0	0
Bacteria|Actinobacteria|Actinobacteria|Actinomycetales|Frankiacea		0	0	0	0	0	0	0	0	0	0	0	0	0	0	0	0
Bacteria|Actinobacteria|Actinobacteria|Actinomycetales|Frankiacea		0	0	0	0	0	0	0	0	0	0	0	0	0	0	0	0
Bacteria|Actinobacteria|Actinobacteria|Actinomycetales|Geoderma		0	0	0	0	0	0	0	0	0	0	0	0	0	0	0	0
Bacteria|Actinobacteria|Actinobacteria|Actinomycetales|Geoderma		0	0	0	0	0	0	0	0	0	0	0	0	0	0	0	0
Bacteria|Actinobacteria|Actinobacteria|Actinomycetales|Geoderma		0	0	0	0	0	0	0	0	0	0	0	0	0	0	0	0
Bacteria|Actinobacteria|Actinobacteria|Actinomycetales|Geoderma		0	0	0	0	0	0	0	0	0	0	0	0	0	0	0	0
Bacteria|Actinobacteria|Actinobacteria|Actinomycetales|Gordoniac		0	0	0	0	0	0	0	0	0	0	0	0	0	0	0	0
Bacteria|Actinobacteria|Actinobacteria|Actinomycetales|Intraspora		0	0	0	0	0	0	0	0	0	0	0	0	0	0	0	0
Bacteria|Actinobacteria|Actinobacteria|Actinomycetales|Intraspora		0	0	0	0	0	0	0	0	0	0	0	0	0	0	0	0
Bacteria|Actinobacteria|Actinobacteria|Actinomycetales|Intraspora		0	0	0	0	0	0	0	0	0	0	0	0	0	0	0	0
Bacteria|Actinobacteria|Actinobacteria|Actinomycetales|Jonesiacea		0	0	0	0	0	0	0	0	0	0	0	0	0	0	0	0
Bacteria|Actinobacteria|Actinobacteria|Actinomycetales|Kineospor		0	0	0	0	0	0	0	0	0	0	0	0	0	0	0	0
Bacteria|Actinobacteria|Actinobacteria|Actinomycetales|Microbact		0	0	0	0	0	0	0	0	0	0	0	0	0	0	0	0
Bacteria|Actinobacteria|Actinobacteria|Actinomycetales|Microbact		0	0	0	0	0	0	0	0	0	0	0	0	0	0	0	0
Bacteria|Actinobacteria|Actinobacteria|Actinomycetales|Microbact		0	0	0	0	0	0	0	0	0	0	0	0	0	0	0	0
Bacteria|Actinobacteria|Actinobacteria|Actinomycetales|Microbact		0	0	0	0	0	0	0	0	0	0	0	0	0	0	0	0
Bacteria|Actinobacteria|Actinobacteria|Actinomycetales|Microbact		0	0	0	0	1	0	0	0	0	0	0	0	0	0	1	0
Bacteria|Actinobacteria|Actinobacteria|Actinomycetales|Microbact		0	0	0	0	0	0	0	0	0	0	0	0	0	0	0	0
Bacteria|Actinobacteria|Actinobacteria|Actinomycetales|Microbact		0	0	0	0	0	0	0	0	0	0	0	0	0	0	0	0
Bacteria|Actinobacteria|Actinobacteria|Actinomycetales|Microbact		0	0	0	1	0	1	0	0	0	1	2	0	0	0	0	0
Bacteria|Actinobacteria|Actinobacteria|Actinomycetales|Microbact		0	0	0	0	0	0	0	0	0	0	0	0	0	0	0	0
Bacteria|Actinobacteria|Actinobacteria|Actinomycetales|Micrococc		0	0	0	0	0	0	0	0	0	0	0	0	0	0	0	0
Bacteria|Actinobacteria|Actinobacteria|Actinomycetales|Micrococc		0	0	0	0	0	0	0	0	0	0	0	0	0	0	0	0
Bacteria|Actinobacteria|Actinobacteria|Actinomycetales|Micrococc		0	0	0	0	0	0	0	0	0	0	0	0	0	0	0	0
Bacteria|Actinobacteria|Actinobacteria|Actinomycetales|Micrococc		0	0	0	0	0	0	0	0	0	0	0	0	0	0	0	0
Bacteria|Actinobacteria|Actinobacteria|Actinomycetales|Micrococc		0	0	0	0	0	0	0	0	0	0	0	0	0	0	0	0
Bacteria|Actinobacteria|Actinobacteria|Actinomycetales|Micrococc		0	1	1	0	0	0	1	0	0	0	1	0	0	0	0	0
Bacteria|Actinobacteria|Actinobacteria|Actinomycetales|Micrococc		1213	1323	1083	4179	158	226	4096	881	940	1955	3135	4891	4829	1376	183	662
Bacteria|Actinobacteria|Actinobacteria|Actinomycetales|Micrococc		0	0	0	0	0	0	0	0	0	0	0	0	0	0	0	0
Bacteria|Actinobacteria|Actinobacteria|Actinomycetales|Micrococc		0	0	0	0	0	0	0	0	0	0	0	0	0	0	0	0
Bacteria|Actinobacteria|Actinobacteria|Actinomycetales|Mycobact		0	0	0	0	0	0	0	0	0	0	0	0	0	0	0	0
Bacteria|Actinobacteria|Actinobacteria|Actinomycetales|Nakamure		0	0	0	0	0	0	0	0	0	0	0	0	0	0	0	0
Bacteria|Actinobacteria|Actinobacteria|Actinomycetales|Nocardiac		0	0	0	0	0	0	0	0	0	0	0	0	0	0	0	0

Microbial Clade	Sample ID<br>Group<br>Body Site	C1.002<br>Oral Ca<br>Sa	C1.003<br>Oral Ca<br>Sa	C1.006<br>Oral Ca<br>Sa	C1.009<br>Oral Ca<br>Sa	C1.010<br>Oral Ca<br>Sa	C1.012<br>Oral Ca<br>Sa	C1.013<br>Oral Ca<br>Sa	C1.014<br>Oral Ca<br>Sa	C1.016<br>Oral Ca<br>Sa	C1.017<br>Oral Ca<br>Sa	C1.018<br>Oral Ca<br>Sa	C1.019<br>Oral Ca<br>Sa	C1.020<br>Oral Ca<br>Sa	C1.021<br>Oral Ca<br>Sa	C1.024<br>Oral Ca<br>Sa	C1.025<br>Oral Ca<br>Sa
Bacteria|Actinobacteria|Actinobacteria|Actinomycetales|Nocardiac		0	0	0	0	0	0	0	0	0	0	0	0	0	0	1	0
Bacteria|Actinobacteria|Actinobacteria|Actinomycetales|Nocardiac		0	0	0	0	0	0	0	0	0	0	0	0	0	0	0	0
Bacteria|Actinobacteria|Actinobacteria|Actinomycetales|Nocardiac		0	0	0	0	0	0	0	0	0	0	0	0	0	0	0	0
Bacteria|Actinobacteria|Actinobacteria|Actinomycetales|Nocardioi		0	0	0	0	0	0	0	0	0	0	0	0	0	0	0	0
Bacteria|Actinobacteria|Actinobacteria|Actinomycetales|Nocardioi		0	0	0	0	0	0	0	0	0	0	0	0	0	0	0	0
Bacteria|Actinobacteria|Actinobacteria|Actinomycetales|Nocardioi		0	0	0	0	0	0	0	0	0	0	0	0	0	0	0	0
Bacteria|Actinobacteria|Actinobacteria|Actinomycetales|Nocardioi		0	0	0	2	0	0	0	0	0	0	0	0	0	0	0	0
Bacteria|Actinobacteria|Actinobacteria|Actinomycetales|Nocardioi		0	0	0	0	0	0	0	0	0	0	0	0	0	0	0	0
Bacteria|Actinobacteria|Actinobacteria|Actinomycetales|Nocardioi		0	0	0	0	0	0	0	0	0	0	0	0	0	0	0	0
Bacteria|Actinobacteria|Actinobacteria|Actinomycetales|Nocardiop		0	0	0	0	0	0	0	0	0	0	0	0	0	0	0	0
Bacteria|Actinobacteria|Actinobacteria|Actinomycetales|Nocardiop		0	0	0	0	0	0	0	0	0	0	0	0	0	0	0	0
Bacteria|Actinobacteria|Actinobacteria|Actinomycetales|Promicron		0	0	0	0	0	0	0	0	0	0	0	0	0	0	0	0
Bacteria|Actinobacteria|Actinobacteria|Actinomycetales|Promicron		0	0	0	0	0	0	0	0	0	0	0	0	0	0	0	0
Bacteria|Actinobacteria|Actinobacteria|Actinomycetales|Propionib		0	0	0	0	0	0	0	0	0	0	0	0	0	0	0	0
Bacteria|Actinobacteria|Actinobacteria|Actinomycetales|Propionib		0	0	0	0	0	0	0	0	0	0	1	0	1	0	1	0
Bacteria|Actinobacteria|Actinobacteria|Actinomycetales|Propionib		0	1	0	1	0	0	0	0	0	0	0	0	0	0	0	0
Bacteria|Actinobacteria|Actinobacteria|Actinomycetales|Propionib		18	273	185	66	14	13	10	176	136	6	31	19	278	62	13	2
Bacteria|Actinobacteria|Actinobacteria|Actinomycetales|Pseudono		0	0	0	0	0	0	0	0	0	0	0	0	0	0	0	0
Bacteria|Actinobacteria|Actinobacteria|Actinomycetales|Pseudono		0	0	0	0	0	0	0	0	0	0	0	0	0	0	0	0
Bacteria|Actinobacteria|Actinobacteria|Actinomycetales|Pseudono		0	0	0	0	0	0	0	0	0	0	0	0	0	0	0	0
Bacteria|Actinobacteria|Actinobacteria|Actinomycetales|Pseudono		0	0	0	0	0	0	0	0	0	0	0	0	0	0	0	0
Bacteria|Actinobacteria|Actinobacteria|Actinomycetales|Sporichthy		0	0	0	0	0	0	0	0	0	0	0	0	0	0	0	0
Bacteria|Actinobacteria|Actinobacteria|Actinomycetales|Streptomy		0	0	0	0	0	0	0	0	0	0	0	0	0	0	0	0
Bacteria|Actinobacteria|Actinobacteria|Actinomycetales|Tsukamur		0	0	0	0	0	0	0	0	0	0	0	0	0	0	0	0
Bacteria|Actinobacteria|Actinobacteria|Actinomycetales|Williamsia		0	0	0	0	0	0	0	0	0	0	0	0	0	0	0	0
Bacteria|Actinobacteria|Actinobacteria|Actinomycetales|Yaniellace		0	0	0	0	0	0	0	0	0	0	0	0	0	0	0	0
Bacteria|Actinobacteria|Actinobacteria|Actinomycetales_Other		0	0	0	0	0	0	0	0	0	0	0	0	0	0	0	0
Bacteria|Actinobacteria|Actinobacteria|Bifidobacteriales|Bifidobac		0	1	0	137	2	2	297	7	2	5	10	0	57	0	3	2
Bacteria|Actinobacteria|Actinobacteria|Bifidobacteriales|Bifidobac		0	0	0	0	0	0	0	0	0	0	0	0	0	0	0	0
Bacteria|Actinobacteria|Actinobacteria|Bifidobacteriales|Bifidobac		1	0	0	1	2	0	4	0	1	0	10	0	2	3	1	0
Bacteria|Actinobacteria|Actinobacteria|Bifidobacteriales|Bifidobac		0	0	0	0	0	1	0	0	0	0	0	0	0	0	0	0
Bacteria|Actinobacteria|Actinobacteria|Bifidobacteriales|Bifidobac		5	0	0	1	98	27	0	0	0	6	0	11	0	0	116	21
Bacteria|Actinobacteria|Coriobacteriia|Coriobacteriales|Coriobacte		0	0	0	0	0	0	0	0	0	0	0	1	0	0	0	0
Bacteria|Actinobacteria|Coriobacteriia|Coriobacteriales|Coriobacte		116	65	33	22	3	5	130	346	30	310	80	118	210	269	6	25
Bacteria|Actinobacteria|Coriobacteriia|Coriobacteriales|Coriobacte		0	0	0	1	0	0	0	0	0	0	0	0	0	0	0	0
Bacteria|Actinobacteria|Coriobacteriia|Coriobacteriales|Coriobacte		0	0	0	1	0	0	0	0	0	0	0	0	0	0	0	0
Bacteria|Actinobacteria|Coriobacteriia|Coriobacteriales|Coriobacte		4	1	1	0	0	0	7	3	1	8	5	2	3	10	0	0

Sample ID	C1.002	C1.003	C1.006	C1.009	C1.010	C1.012	C1.013	C1.014	C1.016	C1.017	C1.018	C1.019	C1.020	C1.021	C1.024	C1.025
Microbial Clade Group	Oral Ca	Oral Ca	Oral Ca	Oral Ca	Oral Ca	Oral Ca	Oral Ca	Oral Ca	Oral Ca	Oral Ca	Oral Ca	Oral Ca	Oral Ca	Oral Ca	Oral Ca	Oral Ca
Body Site	Sa	Sa	Sa	Sa	Sa	Sa	Sa	Sa	Sa	Sa	Sa	Sa	Sa	Sa	Sa	Sa
Bacteria|Actinobacteria|Coriobacteriia|Coriobacteriales|Coriobacte	1	0	0	0	0	0	18	0	0	1	13	0	1	1	0	0
Bacteria|Actinobacteria|Coriobacteriia|Coriobacteriales|Coriobacte	2	6	0	4	22	6	31	5	2	3	91	2	4	1	14	3
Bacteria|Actinobacteria|Nitriliruptoria|Nitriliruptorales|Nitrilirupto	0	0	0	0	0	0	0	0	0	0	0	0	0	0	0	0
Bacteria|Actinobacteria|Rubrobacteria|Rubrobacterales|Rubrobacte	0	0	0	0	0	0	0	0	0	0	0	0	0	0	0	0
Bacteria|Actinobacteria|Thermoleophilia|Solirubrobacterales|Patul	0	0	0	0	0	0	0	0	0	0	0	0	0	0	0	0
Bacteria|Actinobacteria|Thermoleophilia|Solirubrobacterales|Solirı	0	0	0	0	0	0	0	0	0	0	0	0	0	0	0	0
Bacteria|Armatimonadetes|[Fimbriimonadia]|[Fimbriimonadales]|[	0	0	0	0	0	1	0	0	0	0	0	0	0	0	0	0
Bacteria|Armatimonadetes|Armatimonadia|Armatimonadales|Arma	0	0	0	0	0	0	0	0	0	0	0	0	0	0	0	0
Bacteria|Bacteroidetes|[Saprospirae]|[Saprospirales]|Chitinophaga	0	0	0	0	0	0	0	0	0	0	0	0	0	0	0	0
Bacteria|Bacteroidetes|[Saprospirae]|[Saprospirales]|Chitinophaga	0	0	0	0	0	0	0	0	0	0	0	0	0	0	0	0
Bacteria|Bacteroidetes|[Saprospirae]|[Saprospirales]|Chitinophaga	0	0	0	0	0	0	0	0	0	0	0	0	0	0	0	0
Bacteria|Bacteroidetes|[Saprospirae]|[Saprospirales]|Chitinophaga	0	0	0	1	1	0	0	0	0	0	0	1	0	0	1	0
Bacteria|Bacteroidetes|[Saprospirae]|[Saprospirales]|Chitinophaga	0	0	0	0	0	0	0	0	0	0	0	0	0	0	0	0
Bacteria|Bacteroidetes|[Saprospirae]|[Saprospirales]|Saprospiracea	0	0	0	0	0	0	0	0	0	0	0	0	0	0	0	0
Bacteria|Bacteroidetes|[Saprospirae]|[Saprospirales]|Saprospiracea	0	0	0	0	0	0	0	0	0	0	0	0	0	0	0	0
Bacteria|Bacteroidetes|Bacteroidia|Bacteroidales|[Barnesiellaceae]	0	0	0	0	0	0	0	0	0	0	0	0	0	0	0	0
Bacteria|Bacteroidetes|Bacteroidia|Bacteroidales|[Odoribacteracea	0	0	0	6	0	0	0	0	0	0	0	0	0	0	0	0
Bacteria|Bacteroidetes|Bacteroidia|Bacteroidales|[Odoribacteracea	0	0	0	3	0	0	0	0	0	0	0	0	0	0	0	0
Bacteria|Bacteroidetes|Bacteroidia|Bacteroidales|[Paraprevotellace	2118	2306	1522	2966	1223	641	938	4152	2050	2582	1838	2478	3792	2644	1311	1826
Bacteria|Bacteroidetes|Bacteroidia|Bacteroidales|[Paraprevotellace	0	0	0	4	0	0	0	0	0	0	0	0	0	1	0	0
Bacteria|Bacteroidetes|Bacteroidia|Bacteroidales|[Paraprevotellace	0	0	0	2	0	0	0	0	0	0	0	0	0	0	0	0
Bacteria|Bacteroidetes|Bacteroidia|Bacteroidales|[Paraprevotellace	0	0	0	0	0	0	2	0	0	0	1	0	0	0	0	0
Bacteria|Bacteroidetes|Bacteroidia|Bacteroidales|Bacteroidaceae|B	5	2	0	346	6	1	20	1	2	0	77	0	8	5	2	5
Bacteria|Bacteroidetes|Bacteroidia|Bacteroidales|BS11_unclassifie	14	16	0	2	0	0	72	0	0	1	0	0	0	0	0	0
Bacteria|Bacteroidetes|Bacteroidia|Bacteroidales|p-2534-18B5_un	0	0	0	4	1	1	0	0	0	0	0	0	0	0	0	0
Bacteria|Bacteroidetes|Bacteroidia|Bacteroidales|Porphyromonada	0	0	0	0	0	0	0	0	0	0	0	0	0	0	0	0
Bacteria|Bacteroidetes|Bacteroidia|Bacteroidales|Porphyromonada	1	1	17	0	4	2	0	1	0	0	3	0	8	1	19	1
Bacteria|Bacteroidetes|Bacteroidia|Bacteroidales|Porphyromonada	0	0	0	21	0	1	0	0	0	0	0	0	0	0	0	0
Bacteria|Bacteroidetes|Bacteroidia|Bacteroidales|Porphyromonada	1137	4225	3136	2692	1608	1095	1320	274	1487	201	1392	811	830	1516	3723	1495
Bacteria|Bacteroidetes|Bacteroidia|Bacteroidales|Porphyromonada	47	220	115	83	52	74	158	13	122	12	24	8	54	20	368	8
Bacteria|Bacteroidetes|Bacteroidia|Bacteroidales|Porphyromonada	0	0	0	0	0	0	0	0	0	0	0	0	0	0	0	0
Bacteria|Bacteroidetes|Bacteroidia|Bacteroidales|Prevotellaceae|Pr	792	435	522	475	407	166	1403	545	190	981	2457	419	2059	785	704	331
Bacteria|Bacteroidetes|Bacteroidia|Bacteroidales|RF16_unclassifie	0	0	0	1	0	0	1	0	0	0	0	0	0	0	0	0
Bacteria|Bacteroidetes|Bacteroidia|Bacteroidales|Rikenellaceae|AF	0	0	0	0	0	0	0	0	0	0	0	0	0	0	0	0
Bacteria|Bacteroidetes|Bacteroidia|Bacteroidales|Rikenellaceae|Bl	0	0	0	0	0	0	0	0	0	1	0	0	0	0	2	0
Bacteria|Bacteroidetes|Bacteroidia|Bacteroidales|Rikenellaceae|Rik	0	0	0	0	0	0	0	0	0	0	0	0	0	0	0	0
Bacteria|Bacteroidetes|Bacteroidia|Bacteroidales|Rikenellaceae_un	0	0	0	33	0	0	0	0	0	0	0	0	0	0	0	0

Microbial Clade	Sample ID<br>Group<br>Body Site	C1.002<br>Oral Ca<br>Sa	C1.003<br>Oral Ca<br>Sa	C1.006<br>Oral Ca<br>Sa	C1.009<br>Oral Ca<br>Sa	C1.010<br>Oral Ca<br>Sa	C1.012<br>Oral Ca<br>Sa	C1.013<br>Oral Ca<br>Sa	C1.014<br>Oral Ca<br>Sa	C1.016<br>Oral Ca<br>Sa	C1.017<br>Oral Ca<br>Sa	C1.018<br>Oral Ca<br>Sa	C1.019<br>Oral Ca<br>Sa	C1.020<br>Oral Ca<br>Sa	C1.021<br>Oral Ca<br>Sa	C1.024<br>Oral Ca<br>Sa	C1.025<br>Oral Ca<br>Sa
Bacteria|Bacteroidetes|Bacteroidia|Bacteroidales|S24-7_unclassifie		0	0	1	10	2	2	1	0	0	0	1	0	0	0	4	0
Bacteria|Bacteroidetes|Cytophagia|Cytophagales|Cyclobacteriaceae		0	0	0	0	0	0	0	0	0	0	0	0	0	0	0	0
Bacteria|Bacteroidetes|Cytophagia|Cytophagales|Cytophagaceae|A		0	0	0	0	0	0	0	0	0	0	0	0	0	0	0	0
Bacteria|Bacteroidetes|Cytophagia|Cytophagales|Cytophagaceae|C		0	0	0	0	0	0	0	0	0	0	0	0	0	0	0	0
Bacteria|Bacteroidetes|Cytophagia|Cytophagales|Cytophagaceae|D		0	0	0	0	0	0	0	0	0	0	0	0	0	0	0	0
Bacteria|Bacteroidetes|Cytophagia|Cytophagales|Cytophagaceae|E		0	0	0	0	0	0	0	0	0	0	0	0	0	0	0	0
Bacteria|Bacteroidetes|Cytophagia|Cytophagales|Cytophagaceae|H		0	0	0	0	0	0	0	0	0	0	0	0	0	0	0	0
Bacteria|Bacteroidetes|Cytophagia|Cytophagales|Cytophagaceae|P		0	0	0	0	0	0	0	0	0	0	0	0	0	0	0	0
Bacteria|Bacteroidetes|Cytophagia|Cytophagales|Cytophagaceae|R		0	0	0	0	0	0	0	0	0	0	0	0	0	0	0	0
Bacteria|Bacteroidetes|Cytophagia|Cytophagales|Cytophagaceae|S		0	0	0	0	0	0	0	0	0	0	0	0	0	0	0	0
Bacteria|Bacteroidetes|Cytophagia|Cytophagales|Cytophagaceae|S		0	0	0	0	0	0	0	0	0	0	0	0	0	0	0	0
Bacteria|Bacteroidetes|Cytophagia|Cytophagales|Cytophagaceae_u		0	0	0	0	0	0	0	0	0	0	0	0	0	0	0	0
Bacteria|Bacteroidetes|Flavobacteriia|Flavobacteriales|[Weeksellac		0	0	0	0	0	0	0	0	0	0	0	0	0	0	2	3
Bacteria|Bacteroidetes|Flavobacteriia|Flavobacteriales|[Weeksellac		0	0	0	0	0	0	0	0	0	0	0	0	0	0	0	0
Bacteria|Bacteroidetes|Flavobacteriia|Flavobacteriales|[Weeksellac		0	0	0	0	0	0	0	0	0	0	0	0	0	0	1	0
Bacteria|Bacteroidetes|Flavobacteriia|Flavobacteriales|[Weeksellac		0	0	0	0	0	0	0	0	0	0	0	0	0	0	0	0
Bacteria|Bacteroidetes|Flavobacteriia|Flavobacteriales|[Weeksellac		0	0	0	0	0	0	0	0	0	0	0	0	0	0	2	0
Bacteria|Bacteroidetes|Flavobacteriia|Flavobacteriales|[Weeksellac		5	2	15	1	80	1	1	2	0	1	2	1	1	4	88	7
Bacteria|Bacteroidetes|Flavobacteriia|Flavobacteriales|Cryomorpha		0	0	0	0	0	0	0	0	0	0	0	0	0	0	0	0
Bacteria|Bacteroidetes|Flavobacteriia|Flavobacteriales|Flavobacter		51	41	158	130	101	63	79	58	23	22	44	19	45	40	259	36
Bacteria|Bacteroidetes|Flavobacteriia|Flavobacteriales|Flavobacter		0	0	0	0	0	0	0	0	0	0	0	0	0	0	1	0
Bacteria|Bacteroidetes|Flavobacteriia|Flavobacteriales|Flavobacter		0	0	0	0	0	0	0	0	0	0	0	0	0	0	0	0
Bacteria|Bacteroidetes|Flavobacteriia|Flavobacteriales_Other		0	0	0	0	0	0	0	0	0	0	0	0	0	0	0	0
Bacteria|Bacteroidetes|Sphingobacteriia|Sphingobacteriales|Sphing		0	0	0	0	0	0	0	0	0	0	3	0	0	0	0	5
Bacteria|Bacteroidetes|Sphingobacteriia|Sphingobacteriales|Sphing		0	0	0	0	0	0	0	0	0	0	0	0	0	0	0	0
Bacteria|Bacteroidetes|Sphingobacteriia|Sphingobacteriales|Sphing		0	0	0	0	0	0	0	0	0	0	0	0	0	0	0	2
Bacteria|Chlamydiae|Chlamydiia|Chlamydiales|Rhabdochlamydiac		0	0	0	0	0	0	0	0	0	0	0	0	0	0	0	0
Bacteria|Chlamydiae|Chlamydiia|Chlamydiales|Waddliaceae_uncla		0	0	0	0	0	0	0	0	0	0	0	0	0	0	0	0
Bacteria|Chloroflexi|Anaerolineae|Anaerolineales|Anaerolinaceae|		0	0	0	0	1	0	0	0	0	0	0	0	0	0	0	0
Bacteria|Chloroflexi|Anaerolineae|Caldilineales|Caldilineaceae|Cal		0	0	0	0	0	0	0	0	0	0	0	0	0	0	0	0
Bacteria|Chloroflexi|Anaerolineae|Caldilineales|Caldilineaceae_un		0	0	0	0	0	0	0	0	0	0	0	0	0	0	0	0
Bacteria|Chloroflexi|Anaerolineae|SBR1031|A4b_unclassified		0	0	0	0	0	0	0	0	0	0	0	0	0	0	0	0
Bacteria|Cyanobacteria|Oscillatoriophycideae|Chroococcales|Xeno		0	0	0	0	0	0	0	0	0	0	0	0	0	0	0	0
Bacteria|Cyanobacteria|Oscillatoriophycideae|Oscillatoriales|Phorr		0	0	0	0	0	0	0	0	0	0	0	0	0	0	0	0
Bacteria|Fibrobacteres|Fibrobacteria|Fibrobacterales|Fibrobacterac		0	0	0	2	0	0	0	0	0	0	0	0	0	0	0	0
Bacteria|Firmicutes|Bacilli|Bacillales|[Exiguobacteraceae]|Exiguob		0	0	0	0	0	0	0	0	0	0	0	0	0	0	0	0
Bacteria|Firmicutes|Bacilli|Bacillales|[Thermicanaceae]|Thermican		0	0	0	0	0	0	0	0	0	0	0	0	0	0	0	0

Microbial Clade	Sample ID	C1.002	C1.003	C1.006	C1.009	C1.010	C1.012	C1.013	C1.014	C1.016	C1.017	C1.018	C1.019	C1.020	C1.021	C1.024	C1.025
	Group	Oral Ca	Oral Ca	Oral Ca	Oral Ca	Oral Ca	Oral Ca	Oral Ca	Oral Ca	Oral Ca	Oral Ca	Oral Ca	Oral Ca	Oral Ca	Oral Ca	Oral Ca	Oral Ca
	Body Site	Sa	Sa	Sa	Sa	Sa	Sa	Sa	Sa	Sa	Sa	Sa	Sa	Sa	Sa	Sa	Sa
Bacteria|Firmicutes|Bacilli|Bacillales|Alicyclobacillaceae|Alicyclob		0	0	0	0	0	0	0	0	0	0	0	0	0	0	0	0
Bacteria|Firmicutes|Bacilli|Bacillales|Bacillaceae|Anoxybacillus		0	0	0	0	0	0	0	0	0	0	0	0	0	0	0	0
Bacteria|Firmicutes|Bacilli|Bacillales|Bacillaceae|Bacillus		0	0	0	0	0	5	0	0	0	0	0	0	0	0	3	4
Bacteria|Firmicutes|Bacilli|Bacillales|Bacillaceae|Geobacillus		0	0	0	0	0	0	0	0	0	0	0	0	0	0	0	0
Bacteria|Firmicutes|Bacilli|Bacillales|Bacillaceae|Virgibacillus		0	0	0	0	0	0	0	0	0	0	0	0	0	0	0	0
Bacteria|Firmicutes|Bacilli|Bacillales|Bacillaceae_Other		0	0	0	0	0	0	0	0	0	0	0	0	0	0	0	0
Bacteria|Firmicutes|Bacilli|Bacillales|Bacillaceae_unclassified		0	0	0	0	0	0	0	0	0	0	0	0	0	0	0	0
Bacteria|Firmicutes|Bacilli|Bacillales|Listeriaceae|Brochothrix		0	0	0	0	0	0	0	0	0	0	0	0	0	0	0	0
Bacteria|Firmicutes|Bacilli|Bacillales|Paenibacillaceae|Ammoniphi		0	0	0	0	0	0	0	0	0	0	0	0	0	0	0	0
Bacteria|Firmicutes|Bacilli|Bacillales|Paenibacillaceae|Brevibacillu		0	0	0	0	0	0	0	0	0	0	0	0	0	0	1	0
Bacteria|Firmicutes|Bacilli|Bacillales|Paenibacillaceae|Paenibacillu		0	0	0	0	0	0	0	0	0	0	0	0	0	0	0	0
Bacteria|Firmicutes|Bacilli|Bacillales|Planococcaceae|Lysinibacillu		0	0	0	0	0	0	0	0	0	0	0	0	0	0	0	0
Bacteria|Firmicutes|Bacilli|Bacillales|Planococcaceae|Planococcus		0	0	0	0	0	0	0	0	0	0	0	0	0	0	0	0
Bacteria|Firmicutes|Bacilli|Bacillales|Planococcaceae|Planomicrob		0	0	0	0	0	0	0	0	0	0	0	0	0	0	0	0
Bacteria|Firmicutes|Bacilli|Bacillales|Planococcaceae_unclassified		0	0	0	0	1	0	0	0	0	0	0	0	0	0	0	1
Bacteria|Firmicutes|Bacilli|Bacillales|Sporolactobacillaceae|Sporol		0	0	0	0	0	0	0	0	0	0	0	0	0	0	0	0
Bacteria|Firmicutes|Bacilli|Bacillales|Staphylococcaceae|Jeotgalicc		0	0	0	0	0	0	0	0	0	0	0	0	0	0	0	0
Bacteria|Firmicutes|Bacilli|Bacillales|Staphylococcaceae|Macrococ		0	0	0	0	0	0	0	0	0	0	0	0	0	0	0	0
Bacteria|Firmicutes|Bacilli|Bacillales|Staphylococcaceae|Salinicoc		0	0	0	0	0	0	0	0	0	0	0	0	0	0	0	0
Bacteria|Firmicutes|Bacilli|Bacillales|Staphylococcaceae|Staphyloc		0	0	0	0	0	0	0	0	0	0	1	0	0	0	2	1
Bacteria|Firmicutes|Bacilli|Bacillales|Thermoactinomycetaceae|Pla		0	0	0	0	0	0	0	0	0	0	0	0	0	0	0	0
Bacteria|Firmicutes|Bacilli|Bacillales_Other		0	0	0	0	1	0	0	0	0	0	0	0	0	0	0	0
Bacteria|Firmicutes|Bacilli|Gemellales|Gemellaceae|Gemella		0	0	0	0	2	0	0	0	0	0	0	0	0	0	21	1
Bacteria|Firmicutes|Bacilli|Gemellales|Gemellaceae_Other		0	0	0	0	0	0	0	0	0	0	1	0	0	0	1	0
Bacteria|Firmicutes|Bacilli|Gemellales|Gemellaceae_unclassified		4	12	5	12	3	8	13	1	0	4	27	5	10	4	27	1
Bacteria|Firmicutes|Bacilli|Gemellales_Other		0	0	0	0	0	0	0	0	0	0	0	0	0	0	0	0
Bacteria|Firmicutes|Bacilli|Lactobacillales|Aerococcaceae|Abiotrop		5	28	24	9	2	5	3	37	4	0	43	5	13	0	30	4
Bacteria|Firmicutes|Bacilli|Lactobacillales|Aerococcaceae|Aerococ		0	0	0	0	98	1	0	0	0	0	0	0	0	0	31	8
Bacteria|Firmicutes|Bacilli|Lactobacillales|Aerococcaceae|Alkaliba		0	0	0	0	0	0	0	0	0	0	0	0	0	0	0	0
Bacteria|Firmicutes|Bacilli|Lactobacillales|Aerococcaceae|Alloiocc		0	0	0	0	0	0	0	0	0	0	0	0	0	0	0	0
Bacteria|Firmicutes|Bacilli|Lactobacillales|Aerococcaceae|Facklam		0	0	0	0	0	0	0	0	0	0	0	0	0	0	0	0
Bacteria|Firmicutes|Bacilli|Lactobacillales|Aerococcaceae|Marinila		0	0	0	0	0	0	0	0	0	0	0	0	0	0	0	0
Bacteria|Firmicutes|Bacilli|Lactobacillales|Aerococcaceae_Other		0	0	0	0	0	0	0	0	0	0	0	0	0	0	0	0
Bacteria|Firmicutes|Bacilli|Lactobacillales|Aerococcaceae_unclass		0	0	0	0	0	0	0	0	0	0	0	0	0	0	0	0
Bacteria|Firmicutes|Bacilli|Lactobacillales|Carnobacteriaceae|Carn		0	0	0	0	0	0	0	0	0	0	0	0	0	0	0	0
Bacteria|Firmicutes|Bacilli|Lactobacillales|Carnobacteriaceae|Dese		0	0	0	0	0	0	0	0	0	0	0	0	0	0	0	0
Bacteria|Firmicutes|Bacilli|Lactobacillales|Carnobacteriaceae|Gran		88	25	103	96	17	36	180	28	30	112	127	172	267	73	56	29

Microbial Clade																
	Sample ID	C1.002	C1.003	C1.006	C1.009	C1.010	C1.012	C1.013	C1.014	C1.016	C1.017	C1.018	C1.019	C1.020	C1.021	C1.024	C1.025
	Group	Oral Ca	Oral Ca	Oral Ca	Oral Ca	Oral Ca	Oral Ca	Oral Ca	Oral Ca	Oral Ca	Oral Ca	Oral Ca	Oral Ca	Oral Ca	Oral Ca	Oral Ca	Oral Ca
	Body Site	Sa	Sa	Sa	Sa	Sa	Sa	Sa	Sa	Sa	Sa	Sa	Sa	Sa	Sa	Sa	Sa
Bacteria|Firmicutes|Bacilli|Lactobacillales|Carnobacteriaceae|Trich		0	0	0	0	0	0	0	0	0	0	0	0	0	0	0	0
Bacteria|Firmicutes|Bacilli|Lactobacillales|Carnobacteriaceae_Othe		0	0	0	0	0	0	0	0	0	0	0	0	0	0	0	0
Bacteria|Firmicutes|Bacilli|Lactobacillales|Carnobacteriaceae_uncl		0	0	0	0	0	0	0	0	0	0	0	0	0	0	0	0
Bacteria|Firmicutes|Bacilli|Lactobacillales|Enterococcaceae|Entero		0	0	0	0	0	0	0	0	0	0	0	0	0	0	0	0
Bacteria|Firmicutes|Bacilli|Lactobacillales|Enterococcaceae|Vagoc		0	0	0	0	0	0	0	0	0	0	0	0	0	0	0	0
Bacteria|Firmicutes|Bacilli|Lactobacillales|Enterococcaceae_Other		0	0	0	1	0	0	0	0	0	0	0	0	0	0	0	0
Bacteria|Firmicutes|Bacilli|Lactobacillales|Enterococcaceae_unclas		0	0	0	0	0	0	0	0	0	0	0	0	0	0	0	0
Bacteria|Firmicutes|Bacilli|Lactobacillales|Lactobacillaceae|Lactob		0	0	0	59	31	58	22	1	0	0	3	2	0	0	398	55
Bacteria|Firmicutes|Bacilli|Lactobacillales|Leuconostocaceae_uncl		0	0	0	0	0	0	0	0	0	0	0	0	0	0	0	0
Bacteria|Firmicutes|Bacilli|Lactobacillales|Streptococcaceae|Lactoc		0	0	0	0	0	0	0	0	0	0	0	0	0	0	0	0
Bacteria|Firmicutes|Bacilli|Lactobacillales|Streptococcaceae|Strept		627	578	389	633	138	173	1214	505	173	662	810	678	1105	340	424	272
Bacteria|Firmicutes|Bacilli|Lactobacillales|Streptococcaceae_uncla		0	0	0	0	0	0	0	0	0	0	0	0	0	0	0	0
Bacteria|Firmicutes|Bacilli|Lactobacillales_Other		0	2	0	5	0	0	0	1	0	0	2	0	1	0	2	0
Bacteria|Firmicutes|Bacilli|Turicibacterales|Turicibacteraceae|Turic		0	0	0	2	0	0	0	0	0	0	0	0	0	0	0	0
Bacteria|Firmicutes|Bacilli_Other		0	0	0	3	0	1	0	0	0	0	6	0	0	0	0	0
Bacteria|Firmicutes|Clostridia|Clostridiales|[Acidaminobacteraceae		0	0	0	0	0	0	0	0	0	0	0	0	0	0	0	0
Bacteria|Firmicutes|Clostridia|Clostridiales|[Mogibacteriaceae]|An		0	1	2	0	5	0	0	0	0	0	1	0	0	0	0	3
Bacteria|Firmicutes|Clostridia|Clostridiales|[Mogibacteriaceae]|Mo		1	1	0	4	0	0	1	0	0	2	3	1	1	0	2	0
Bacteria|Firmicutes|Clostridia|Clostridiales|[Mogibacteriaceae]_Ot		0	1	0	1	0	1	2	0	0	1	3	0	0	0	0	1
Bacteria|Firmicutes|Clostridia|Clostridiales|[Mogibacteriaceae]_un		56	25	15	27	24	8	41	17	6	68	51	55	105	54	38	27
Bacteria|Firmicutes|Clostridia|Clostridiales|[Tissierellaceae]|1-68		0	0	0	0	1	0	0	0	0	0	0	0	0	0	0	0
Bacteria|Firmicutes|Clostridia|Clostridiales|[Tissierellaceae]|Anaer		0	0	0	0	14	0	0	0	0	0	0	0	0	0	1	4
Bacteria|Firmicutes|Clostridia|Clostridiales|[Tissierellaceae]|Finego		0	0	0	0	5	0	0	0	0	0	0	0	0	0	5	1
Bacteria|Firmicutes|Clostridia|Clostridiales|[Tissierellaceae]|GW-3		0	0	0	0	0	0	0	0	0	0	0	0	0	0	0	0
Bacteria|Firmicutes|Clostridia|Clostridiales|[Tissierellaceae]|Parvir		1	7	5	1	10	1	3	0	0	0	14	0	0	1	10	17
Bacteria|Firmicutes|Clostridia|Clostridiales|[Tissierellaceae]|Peptor		0	0	0	0	4	1	0	0	0	0	0	0	0	0	12	1
Bacteria|Firmicutes|Clostridia|Clostridiales|[Tissierellaceae]|ph2		0	0	0	0	1	0	0	0	0	0	1	0	0	0	0	0
Bacteria|Firmicutes|Clostridia|Clostridiales|[Tissierellaceae]|Sedim		0	0	0	0	0	0	0	0	0	0	0	0	0	0	0	0
Bacteria|Firmicutes|Clostridia|Clostridiales|[Tissierellaceae]|WAL_		0	0	0	0	0	0	0	0	0	0	0	0	0	0	0	0
Bacteria|Firmicutes|Clostridia|Clostridiales|[Tissierellaceae]_uncla		3	2	0	0	0	0	1	0	0	0	4	0	0	0	0	1
Bacteria|Firmicutes|Clostridia|Clostridiales|Christensenellaceae|Ch		0	0	0	0	0	0	0	0	0	0	0	0	0	0	0	0
Bacteria|Firmicutes|Clostridia|Clostridiales|Christensenellaceae_ur		0	0	0	4	1	0	0	0	1	0	0	1	1	0	0	0
Bacteria|Firmicutes|Clostridia|Clostridiales|Clostridiaceae|Caloram		0	0	0	0	0	0	0	0	0	0	0	0	0	0	0	0
Bacteria|Firmicutes|Clostridia|Clostridiales|Clostridiaceae|Clostrid		0	0	0	5	3	1	1	0	0	0	1	0	0	0	0	1
Bacteria|Firmicutes|Clostridia|Clostridiales|Clostridiaceae|SMB53		0	0	0	0	0	0	0	0	0	0	0	0	0	0	0	0
Bacteria|Firmicutes|Clostridia|Clostridiales|Clostridiaceae|Thermoa		0	0	0	0	0	0	0	0	0	0	0	0	0	0	0	0
Bacteria|Firmicutes|Clostridia|Clostridiales|Clostridiaceae_Other		0	0	0	0	0	0	0	0	0	0	0	0	0	0	0	0

Microbial Clade	Sample ID<br>Group<br>Body Site	C1.002<br>Oral Ca<br>Sa	C1.003<br>Oral Ca<br>Sa	C1.006<br>Oral Ca<br>Sa	C1.009<br>Oral Ca<br>Sa	C1.010<br>Oral Ca<br>Sa	C1.012<br>Oral Ca<br>Sa	C1.013<br>Oral Ca<br>Sa	C1.014<br>Oral Ca<br>Sa	C1.016<br>Oral Ca<br>Sa	C1.017<br>Oral Ca<br>Sa	C1.018<br>Oral Ca<br>Sa	C1.019<br>Oral Ca<br>Sa	C1.020<br>Oral Ca<br>Sa	C1.021<br>Oral Ca<br>Sa	C1.024<br>Oral Ca<br>Sa	C1.025<br>Oral Ca<br>Sa
Bacteria|Firmicutes|Clostridia|Clostridiales|Clostridiaceae_unclass		0	0	0	1	0	1	0	0	0	0	0	0	0	0	1	1
Bacteria|Firmicutes|Clostridia|Clostridiales|Dehalobacteriaceae|De		0	0	0	0	0	0	0	0	0	0	0	0	0	0	0	0
Bacteria|Firmicutes|Clostridia|Clostridiales|Dehalobacteriaceae_un		0	0	0	0	0	0	0	0	0	0	0	0	0	0	0	0
Bacteria|Firmicutes|Clostridia|Clostridiales|Eubacteriaceae|Anaero		0	0	0	0	0	0	0	0	0	0	0	0	0	0	0	0
Bacteria|Firmicutes|Clostridia|Clostridiales|Eubacteriaceae|Pseudo		0	1	0	1	0	0	16	1	0	1	1	0	0	0	1	0
Bacteria|Firmicutes|Clostridia|Clostridiales|Lachnospiraceae|[Rum		0	0	0	14	0	0	0	0	0	0	0	0	0	0	0	0
Bacteria|Firmicutes|Clostridia|Clostridiales|Lachnospiraceae|Anaer		0	0	0	2	0	0	0	0	0	0	0	0	0	0	0	0
Bacteria|Firmicutes|Clostridia|Clostridiales|Lachnospiraceae|Blauti		0	0	0	18	1	1	0	0	0	0	0	0	0	1	0	1
Bacteria|Firmicutes|Clostridia|Clostridiales|Lachnospiraceae|Butyr		10	7	7	6	1	9	9	6	19	6	9	22	2	27	17	9
Bacteria|Firmicutes|Clostridia|Clostridiales|Lachnospiraceae|Caton		0	3	3	3	4	0	3	8	0	3	23	6	1	2	21	2
Bacteria|Firmicutes|Clostridia|Clostridiales|Lachnospiraceae|Copro		0	0	0	29	1	3	0	0	0	0	0	0	0	1	0	0
Bacteria|Firmicutes|Clostridia|Clostridiales|Lachnospiraceae|Dorea		0	0	0	14	0	0	0	0	0	0	0	0	0	0	0	0
Bacteria|Firmicutes|Clostridia|Clostridiales|Lachnospiraceae|Epulo		0	0	0	0	0	0	0	0	0	0	0	0	0	0	0	0
Bacteria|Firmicutes|Clostridia|Clostridiales|Lachnospiraceae|Lachn		0	0	0	15	0	0	0	0	0	0	0	0	0	0	0	0
Bacteria|Firmicutes|Clostridia|Clostridiales|Lachnospiraceae|Lachn		0	0	0	19	0	1	0	0	0	0	0	0	0	0	0	0
Bacteria|Firmicutes|Clostridia|Clostridiales|Lachnospiraceae|Moryc		0	1	0	0	0	0	0	0	0	0	2	0	2	0	0	0
Bacteria|Firmicutes|Clostridia|Clostridiales|Lachnospiraceae|Oriba		87	39	72	29	9	24	191	101	55	137	59	148	504	72	10	20
Bacteria|Firmicutes|Clostridia|Clostridiales|Lachnospiraceae|Roseb		0	0	0	42	3	1	0	0	0	0	0	0	0	0	0	0
Bacteria|Firmicutes|Clostridia|Clostridiales|Lachnospiraceae|Shuttl		38	34	17	33	23	1	118	6	2	2	148	52	76	1	7	8
Bacteria|Firmicutes|Clostridia|Clostridiales|Lachnospiraceae_Othe		0	0	0	3	2	0	1	1	1	0	0	2	13	2	0	0
Bacteria|Firmicutes|Clostridia|Clostridiales|Lachnospiraceae_uncla		40	22	17	225	35	6	28	22	17	28	36	57	38	6	100	15
Bacteria|Firmicutes|Clostridia|Clostridiales|Peptococcaceae|Desulf		0	0	0	0	0	0	0	0	0	0	0	0	0	0	0	0
Bacteria|Firmicutes|Clostridia|Clostridiales|Peptococcaceae|Peptoc		4	3	4	2	2	0	2	0	2	0	4	4	4	0	5	0
Bacteria|Firmicutes|Clostridia|Clostridiales|Peptococcaceae|rc4-4		0	0	0	0	0	0	0	0	0	0	0	0	0	0	0	0
Bacteria|Firmicutes|Clostridia|Clostridiales|Peptococcaceae_unclas		0	0	0	2	0	0	0	0	0	0	0	0	0	0	0	0
Bacteria|Firmicutes|Clostridia|Clostridiales|Peptostreptococcaceae|		4	4	0	10	7	1	10	0	0	1	15	0	2	1	15	5
Bacteria|Firmicutes|Clostridia|Clostridiales|Peptostreptococcaceae|		4	2	9	7	24	3	1	8	5	1	16	8	0	2	21	8
Bacteria|Firmicutes|Clostridia|Clostridiales|Peptostreptococcaceae_		0	5	3	1	1	0	1	1	0	0	4	2	0	0	7	0
Bacteria|Firmicutes|Clostridia|Clostridiales|Ruminococcaceae|Ana		0	0	0	0	0	0	0	0	0	0	0	0	0	0	0	0
Bacteria|Firmicutes|Clostridia|Clostridiales|Ruminococcaceae|Etha		0	0	0	0	0	0	0	0	0	0	0	0	0	0	0	0
Bacteria|Firmicutes|Clostridia|Clostridiales|Ruminococcaceae|Faec		1	0	0	204	12	1	0	1	0	0	0	0	0	0	2	0
Bacteria|Firmicutes|Clostridia|Clostridiales|Ruminococcaceae|Osci		0	0	0	34	2	2	0	0	1	0	0	0	0	0	0	0
Bacteria|Firmicutes|Clostridia|Clostridiales|Ruminococcaceae|Rum		0	0	0	24	1	0	0	0	0	0	0	0	0	0	0	0
Bacteria|Firmicutes|Clostridia|Clostridiales|Ruminococcaceae_Oth		0	0	0	1	0	0	0	0	0	0	0	0	0	0	0	0
Bacteria|Firmicutes|Clostridia|Clostridiales|Ruminococcaceae_unc		2	0	2	208	14	5	2	2	0	1	1	1	3	0	1	2
Bacteria|Firmicutes|Clostridia|Clostridiales|Syntrophomonadaceae|		1	0	0	0	0	0	0	0	0	0	2	0	0	0	0	0
Bacteria|Firmicutes|Clostridia|Clostridiales|Veillonellaceae|Anaero		0	0	0	1	1	0	0	0	0	0	0	0	0	0	0	0

Microbial Clade	Sample ID	C1.002	C1.003	C1.006	C1.009	C1.010	C1.012	C1.013	C1.014	C1.016	C1.017	C1.018	C1.019	C1.020	C1.021	C1.024	C1.025
	Group	Oral Ca	Oral Ca	Oral Ca	Oral Ca	Oral Ca	Oral Ca	Oral Ca	Oral Ca	Oral Ca	Oral Ca	Oral Ca	Oral Ca	Oral Ca	Oral Ca	Oral Ca	Oral Ca
	Body Site	Sa	Sa	Sa	Sa	Sa	Sa	Sa	Sa	Sa	Sa	Sa	Sa	Sa	Sa	Sa	Sa
Bacteria|Firmicutes|Clostridia|Clostridiales|Veillonellaceae|Dialiste		15	26	11	57	25	4	207	15	6	4	73	19	15	19	25	10
Bacteria|Firmicutes|Clostridia|Clostridiales|Veillonellaceae|Megam		0	0	0	0	0	0	0	0	0	0	0	0	0	0	0	0
Bacteria|Firmicutes|Clostridia|Clostridiales|Veillonellaceae|Megasp		31	22	2	10	14	3	143	55	4	198	14	35	64	130	3	9
Bacteria|Firmicutes|Clostridia|Clostridiales|Veillonellaceae|Phasco		0	0	0	0	0	0	0	0	0	0	0	0	0	0	0	0
Bacteria|Firmicutes|Clostridia|Clostridiales|Veillonellaceae|Schwar		10	6	0	9	28	0	28	2	0	3	132	0	1	5	44	3
Bacteria|Firmicutes|Clostridia|Clostridiales|Veillonellaceae|Seleno		169	156	117	145	93	49	502	282	70	237	740	126	122	135	458	53
Bacteria|Firmicutes|Clostridia|Clostridiales|Veillonellaceae|Succin		0	0	0	0	0	0	0	0	0	0	0	0	0	0	0	0
Bacteria|Firmicutes|Clostridia|Clostridiales|Veillonellaceae|Thermo		0	0	0	0	0	0	0	0	0	0	0	0	0	0	0	0
Bacteria|Firmicutes|Clostridia|Clostridiales|Veillonellaceae|Veillor		59	17	11	18	10	1	24	84	8	129	70	33	88	50	16	17
Bacteria|Firmicutes|Clostridia|Clostridiales|Veillonellaceae_Other		0	0	0	0	0	0	0	0	0	0	1	0	0	1	0	0
Bacteria|Firmicutes|Clostridia|Clostridiales|Veillonellaceae_unclas		2	5	1	33	5	4	94	0	1	36	70	26	2	0	8	1
Bacteria|Firmicutes|Clostridia|Clostridiales_Other		0	0	0	22	2	3	1	0	0	0	0	1	0	0	1	0
Bacteria|Firmicutes|Clostridia|Thermoanaerobacterales|Thermoana		0	0	0	0	0	0	0	0	0	0	0	0	0	0	0	0
Bacteria|Firmicutes|Erysipelotrichi|Erysipelotrichales|Erysipelotric		0	0	0	6	1	0	0	0	0	0	0	0	0	0	0	0
Bacteria|Firmicutes|Erysipelotrichi|Erysipelotrichales|Erysipelotric		0	0	0	1	0	0	0	0	0	0	0	0	0	0	2	0
Bacteria|Firmicutes|Erysipelotrichi|Erysipelotrichales|Erysipelotric		46	30	13	43	6	4	43	79	4	70	34	83	67	23	26	120
Bacteria|Firmicutes|Erysipelotrichi|Erysipelotrichales|Erysipelotric		0	0	0	3	1	0	0	0	0	0	0	0	0	0	0	0
Bacteria|Firmicutes|Erysipelotrichi|Erysipelotrichales|Erysipelotric		0	0	0	0	0	0	0	0	0	0	0	0	0	0	0	0
Bacteria|Firmicutes|Erysipelotrichi|Erysipelotrichales|Erysipelotric		0	0	0	3	0	0	0	0	0	0	0	0	0	0	0	0
Bacteria|Firmicutes|Erysipelotrichi|Erysipelotrichales|Erysipelotric		0	0	0	2	1	0	0	0	0	0	0	0	0	0	0	0
Bacteria|Firmicutes|Erysipelotrichi|Erysipelotrichales|Erysipelotric		0	0	0	5	1	0	0	0	0	0	0	0	0	0	0	0
Bacteria|Firmicutes|Erysipelotrichi|Erysipelotrichales|Erysipelotric		0	0	0	0	1	0	0	0	0	0	3	0	0	0	1	1
Bacteria|Firmicutes|Erysipelotrichi|Erysipelotrichales|Erysipelotric		0	0	0	0	0	0	0	0	0	0	0	0	0	0	0	0
Bacteria|Firmicutes|Erysipelotrichi|Erysipelotrichales|Erysipelotric		0	0	0	9	1	0	0	0	0	0	81	0	0	0	0	0
Bacteria|Firmicutes_Other		0	0	0	0	0	0	0	0	0	0	0	0	0	0	0	0
Bacteria|Fusobacteria|Fusobacteriia|Fusobacteriales|Fusobacteriace		27	54	28	49	56	37	28	17	7	12	215	18	44	8	115	74
Bacteria|Fusobacteria|Fusobacteriia|Fusobacteriales|Leptotrichiace		5	21	30	23	29	7	12	10	2	15	57	14	5	5	117	21
Bacteria|Fusobacteria|Fusobacteriia|Fusobacteriales|Leptotrichiace		0	0	0	1	27	0	0	0	0	0	0	0	0	0	19	4
Bacteria|Fusobacteria|Fusobacteriia|Fusobacteriales|Leptotrichiace		0	0	0	0	0	0	0	0	0	0	0	0	0	0	0	0
Bacteria|Fusobacteria|Fusobacteriia|Fusobacteriales|Leptotrichiace		0	1	0	0	1	0	0	0	0	0	1	0	0	0	0	0
Bacteria|Lentisphaerae|[Lentisphaeria]|Z20|R4-45B_unclassified		0	0	0	1	0	0	0	0	0	0	0	0	0	0	0	0
Bacteria|Planctomycetes|Planctomycetia|Gemmatales|Gemmatacea		0	0	0	0	0	0	0	0	0	0	0	0	0	0	0	0
Bacteria|Proteobacteria|Alphaproteobacteria|Caulobacterales|Caulo		0	0	0	0	0	0	0	0	0	0	0	0	0	0	0	0
Bacteria|Proteobacteria|Alphaproteobacteria|Caulobacterales|Caulo		0	0	0	0	0	0	0	0	0	0	0	0	0	0	0	0
Bacteria|Proteobacteria|Alphaproteobacteria|Caulobacterales|Caulo		0	0	0	0	0	0	0	0	0	0	0	0	0	0	0	0
Bacteria|Proteobacteria|Alphaproteobacteria|Caulobacterales|Caulo		0	0	0	0	0	0	0	0	0	0	0	0	0	0	1	0
Bacteria|Proteobacteria|Alphaproteobacteria|Caulobacterales|Caulo		0	0	0	0	0	0	0	0	0	0	0	0	0	0	0	0

Microbial Clade	Sample ID<br>Group<br>Body Site	C1.002<br>Oral Ca<br>Sa	C1.003<br>Oral Ca<br>Sa	C1.006<br>Oral Ca<br>Sa	C1.009<br>Oral Ca<br>Sa	C1.010<br>Oral Ca<br>Sa	C1.012<br>Oral Ca<br>Sa	C1.013<br>Oral Ca<br>Sa	C1.014<br>Oral Ca<br>Sa	C1.016<br>Oral Ca<br>Sa	C1.017<br>Oral Ca<br>Sa	C1.018<br>Oral Ca<br>Sa	C1.019<br>Oral Ca<br>Sa	C1.020<br>Oral Ca<br>Sa	C1.021<br>Oral Ca<br>Sa	C1.024<br>Oral Ca<br>Sa	C1.025<br>Oral Ca<br>Sa
Bacteria|Proteobacteria|Alphaproteobacteria|Caulobacterales|Caulc		0	0	0	0	0	0	0	0	0	0	0	0	0	0	6	0
Bacteria|Proteobacteria|Alphaproteobacteria|Caulobacterales|Caulc		0	0	0	0	0	0	0	0	0	0	0	0	0	0	0	0
Bacteria|Proteobacteria|Alphaproteobacteria|Caulobacterales|Caulc		0	1	0	1	4	3	1	0	1	0	1	0	2	0	7	1
Bacteria|Proteobacteria|Alphaproteobacteria|Caulobacterales_Othe		0	0	0	0	0	0	0	0	0	0	0	0	0	0	0	0
Bacteria|Proteobacteria|Alphaproteobacteria|Rhizobiales|Aurantim		0	0	0	0	0	0	0	0	0	0	0	0	0	0	0	0
Bacteria|Proteobacteria|Alphaproteobacteria|Rhizobiales|Bartonell		0	0	0	0	0	0	0	0	0	0	0	0	0	0	0	0
Bacteria|Proteobacteria|Alphaproteobacteria|Rhizobiales|Beijerincl		0	0	0	0	0	0	0	0	0	0	0	0	0	0	0	0
Bacteria|Proteobacteria|Alphaproteobacteria|Rhizobiales|Beijerincl		0	0	0	0	0	0	0	0	0	0	0	0	0	0	0	0
Bacteria|Proteobacteria|Alphaproteobacteria|Rhizobiales|Beijerincl		0	0	0	0	0	0	0	0	0	0	0	0	0	0	0	0
Bacteria|Proteobacteria|Alphaproteobacteria|Rhizobiales|Bradyrhiz		0	0	0	0	0	0	0	0	0	0	0	0	0	0	0	0
Bacteria|Proteobacteria|Alphaproteobacteria|Rhizobiales|Bradyrhiz		0	0	0	0	0	0	0	0	0	0	0	0	0	0	0	0
Bacteria|Proteobacteria|Alphaproteobacteria|Rhizobiales|Bradyrhiz		0	1	0	0	0	0	0	0	0	0	0	0	1	0	2	0
Bacteria|Proteobacteria|Alphaproteobacteria|Rhizobiales|Bradyrhiz		0	0	0	0	0	0	0	0	0	0	0	0	0	0	0	0
Bacteria|Proteobacteria|Alphaproteobacteria|Rhizobiales|Bradyrhiz		0	0	0	0	0	0	0	0	0	0	0	0	0	0	0	0
Bacteria|Proteobacteria|Alphaproteobacteria|Rhizobiales|Brucellac		0	0	0	1	2	1	0	0	0	0	0	0	0	0	3	0
Bacteria|Proteobacteria|Alphaproteobacteria|Rhizobiales|Brucellac		0	0	0	0	0	0	0	0	0	0	0	0	0	0	2	0
Bacteria|Proteobacteria|Alphaproteobacteria|Rhizobiales|Hyphomi		0	0	0	0	0	0	0	0	0	0	0	0	0	0	1	0
Bacteria|Proteobacteria|Alphaproteobacteria|Rhizobiales|Hyphomi		0	0	1	0	0	0	0	0	0	0	0	0	0	0	0	1
Bacteria|Proteobacteria|Alphaproteobacteria|Rhizobiales|Hyphomi		0	0	0	0	0	0	0	0	0	0	0	0	0	0	2	0
Bacteria|Proteobacteria|Alphaproteobacteria|Rhizobiales|Hyphomi		0	0	0	0	0	0	0	0	0	0	0	0	0	0	0	0
Bacteria|Proteobacteria|Alphaproteobacteria|Rhizobiales|Hyphomi		0	0	0	0	0	0	0	0	0	0	0	0	0	0	0	0
Bacteria|Proteobacteria|Alphaproteobacteria|Rhizobiales|Hyphomi		0	0	0	0	0	0	0	0	0	0	0	0	0	0	0	2
Bacteria|Proteobacteria|Alphaproteobacteria|Rhizobiales|Methylob		0	0	0	0	1	0	0	0	0	0	0	1	0	0	2	0
Bacteria|Proteobacteria|Alphaproteobacteria|Rhizobiales|Methylob		0	0	0	0	0	0	0	0	0	0	0	0	0	0	1	0
Bacteria|Proteobacteria|Alphaproteobacteria|Rhizobiales|Methyloc		0	0	0	0	0	0	0	0	0	0	0	0	0	0	0	0
Bacteria|Proteobacteria|Alphaproteobacteria|Rhizobiales|Methyloc		0	0	0	0	0	0	0	0	0	0	0	0	0	0	0	0
Bacteria|Proteobacteria|Alphaproteobacteria|Rhizobiales|Methyloc		0	0	0	0	0	0	0	0	0	0	0	0	0	0	0	0
Bacteria|Proteobacteria|Alphaproteobacteria|Rhizobiales|Phyllobac		0	0	0	0	0	0	0	0	0	0	0	0	0	0	0	0
Bacteria|Proteobacteria|Alphaproteobacteria|Rhizobiales|Phyllobac		0	0	0	0	0	0	0	0	0	0	0	0	0	0	0	0
Bacteria|Proteobacteria|Alphaproteobacteria|Rhizobiales|Phyllobac		0	0	0	0	0	0	0	0	0	0	0	0	0	0	0	3
Bacteria|Proteobacteria|Alphaproteobacteria|Rhizobiales|Phyllobac		0	0	0	0	0	0	0	0	0	0	0	0	0	0	0	0
Bacteria|Proteobacteria|Alphaproteobacteria|Rhizobiales|Rhizobiac		0	0	0	0	0	0	0	0	0	0	0	0	0	0	0	1
Bacteria|Proteobacteria|Alphaproteobacteria|Rhizobiales|Rhizobiac		0	0	0	0	0	0	0	0	0	0	0	0	0	0	0	0
Bacteria|Proteobacteria|Alphaproteobacteria|Rhizobiales|Rhizobiac		0	0	0	0	0	0	0	0	0	0	0	0	0	0	0	1
Bacteria|Proteobacteria|Alphaproteobacteria|Rhizobiales|Rhizobiac		0	0	0	0	0	0	0	0	0	0	0	0	0	0	0	0
Bacteria|Proteobacteria|Alphaproteobacteria|Rhizobiales|Xanthoba		0	0	0	0	0	0	0	0	0	0	0	0	0	0	0	0
Bacteria|Proteobacteria|Alphaproteobacteria|Rhizobiales|Xanthoba		0	0	0	0	0	0	0	0	0	0	0	0	0	0	0	0

Microbial Clade	Sample ID<br>Group<br>Body Site	C1.002<br>Oral Ca<br>Sa	C1.003<br>Oral Ca<br>Sa	C1.006<br>Oral Ca<br>Sa	C1.009<br>Oral Ca<br>Sa	C1.010<br>Oral Ca<br>Sa	C1.012<br>Oral Ca<br>Sa	C1.013<br>Oral Ca<br>Sa	C1.014<br>Oral Ca<br>Sa	C1.016<br>Oral Ca<br>Sa	C1.017<br>Oral Ca<br>Sa	C1.018<br>Oral Ca<br>Sa	C1.019<br>Oral Ca<br>Sa	C1.020<br>Oral Ca<br>Sa	C1.021<br>Oral Ca<br>Sa	C1.024<br>Oral Ca<br>Sa	C1.025<br>Oral Ca<br>Sa
Bacteria|Proteobacteria|Alphaproteobacteria|Rhizobiales|Xanthoba		0	0	0	0	0	0	0	0	0	0	0	0	0	0	0	0
Bacteria|Proteobacteria|Alphaproteobacteria|Rhizobiales_Other		0	0	0	0	0	0	0	0	0	0	0	0	0	0	0	0
Bacteria|Proteobacteria|Alphaproteobacteria|Rhodobacterales|Hypl		0	0	0	0	0	0	0	0	0	0	0	0	0	0	0	0
Bacteria|Proteobacteria|Alphaproteobacteria|Rhodobacterales|Rhoc		0	0	0	0	0	0	0	0	0	0	0	0	0	0	0	0
Bacteria|Proteobacteria|Alphaproteobacteria|Rhodobacterales|Rhoc		0	0	0	0	0	0	0	0	0	0	0	0	0	0	0	0
Bacteria|Proteobacteria|Alphaproteobacteria|Rhodobacterales|Rhoc		0	0	0	0	0	0	0	0	0	0	0	0	0	0	1	0
Bacteria|Proteobacteria|Alphaproteobacteria|Rhodobacterales|Rhoc		0	0	0	0	0	0	0	0	0	0	0	0	0	0	0	0
Bacteria|Proteobacteria|Alphaproteobacteria|Rhodobacterales|Rhoc		0	0	0	0	0	0	0	0	0	0	0	0	0	0	0	0
Bacteria|Proteobacteria|Alphaproteobacteria|Rhodobacterales|Rhoc		0	0	1	0	0	0	0	0	0	0	0	0	0	0	0	0
Bacteria|Proteobacteria|Alphaproteobacteria|Rhodospirillales|Acet		0	0	0	0	1	31	0	0	0	0	0	0	0	0	0	0
Bacteria|Proteobacteria|Alphaproteobacteria|Rhodospirillales|Acet		0	0	0	0	0	0	0	0	0	0	0	0	0	0	0	0
Bacteria|Proteobacteria|Alphaproteobacteria|Rhodospirillales|Acet		0	0	0	0	0	0	0	0	0	0	0	0	0	0	0	0
Bacteria|Proteobacteria|Alphaproteobacteria|Rhodospirillales|Acet		0	0	0	0	0	0	0	0	0	0	0	0	0	0	0	0
Bacteria|Proteobacteria|Alphaproteobacteria|Rhodospirillales|Acet		0	0	0	0	0	0	0	0	0	0	0	0	0	0	0	0
Bacteria|Proteobacteria|Alphaproteobacteria|Rhodospirillales|Acet		0	0	0	0	0	1	0	0	0	0	0	0	0	0	0	0
Bacteria|Proteobacteria|Alphaproteobacteria|Rhodospirillales|Rhoc		0	0	0	0	0	0	0	0	0	0	0	0	0	0	0	0
Bacteria|Proteobacteria|Alphaproteobacteria|Rhodospirillales|Rhoc		0	0	0	0	0	0	0	0	0	0	0	0	0	0	0	0
Bacteria|Proteobacteria|Alphaproteobacteria|Rhodospirillales|Rhoc		0	0	0	0	0	0	0	0	0	0	0	0	0	0	0	0
Bacteria|Proteobacteria|Alphaproteobacteria|Rhodospirillales|Rhoc		0	0	0	1	0	0	0	0	0	0	0	0	0	0	0	0
Bacteria|Proteobacteria|Alphaproteobacteria|Rhodospirillales|Rhoc		0	0	0	0	0	0	0	0	0	0	0	0	0	0	0	0
Bacteria|Proteobacteria|Alphaproteobacteria|Rickettsiales|mitochor		0	0	0	0	0	0	0	0	0	0	0	0	0	0	0	0
Bacteria|Proteobacteria|Alphaproteobacteria|Rickettsiales|Pelagiba		0	0	0	0	0	0	0	0	0	0	0	0	0	0	0	0
Bacteria|Proteobacteria|Alphaproteobacteria|Rickettsiales|Rickettsi		0	0	0	0	0	0	0	0	0	0	0	0	0	0	0	0
Bacteria|Proteobacteria|Alphaproteobacteria|Sphingomonadales|Er		0	0	0	0	0	0	0	0	0	0	0	0	0	0	0	0
Bacteria|Proteobacteria|Alphaproteobacteria|Sphingomonadales|Er		0	0	0	0	1	0	0	0	0	0	0	0	0	0	0	0
Bacteria|Proteobacteria|Alphaproteobacteria|Sphingomonadales|Sp		0	0	0	0	0	0	0	0	0	0	0	0	0	0	0	0
Bacteria|Proteobacteria|Alphaproteobacteria|Sphingomonadales|Sp		0	0	0	0	0	0	0	0	0	0	0	0	0	0	0	1
Bacteria|Proteobacteria|Alphaproteobacteria|Sphingomonadales|Sp		0	0	0	0	3	1	0	0	0	0	0	0	0	0	13	0
Bacteria|Proteobacteria|Alphaproteobacteria|Sphingomonadales|Sp		0	0	0	0	4	0	0	0	0	0	0	0	0	0	1	0
Bacteria|Proteobacteria|Alphaproteobacteria|Sphingomonadales|Sp		0	1	0	0	11	0	0	1	0	0	0	0	0	0	613	7
Bacteria|Proteobacteria|Alphaproteobacteria|Sphingomonadales|Sp		0	0	0	0	0	0	0	0	0	0	0	0	0	0	0	0
Bacteria|Proteobacteria|Alphaproteobacteria|Sphingomonadales|Sp		0	0	0	0	0	0	0	0	0	0	0	0	0	0	1	0
Bacteria|Proteobacteria|Alphaproteobacteria|Sphingomonadales|Sp		0	0	0	0	0	0	0	0	0	0	0	0	0	0	0	0
Bacteria|Proteobacteria|Alphaproteobacteria|Sphingomonadales_C		0	0	0	0	0	0	0	0	0	0	0	0	0	0	0	0
Bacteria|Proteobacteria|Alphaproteobacteria_Other		0	0	0	0	0	0	0	0	0	0	0	0	0	0	0	0
Bacteria|Proteobacteria|Betaproteobacteria|Burkholderiales|Alcalig		0	0	0	0	0	0	0	0	0	0	0	0	0	0	2	0
Bacteria|Proteobacteria|Betaproteobacteria|Burkholderiales|Alcalig		0	0	0	32	0	1	0	0	0	0	0	0	0	0	0	0

Microbial Clade / Sample ID	C1.002	C1.003	C1.006	C1.009	C1.010	C1.012	C1.013	C1.014	C1.016	C1.017	C1.018	C1.019	C1.020	C1.021	C1.024	C1.025
Group	Oral Ca	Oral Ca	Oral Ca	Oral Ca	Oral Ca	Oral Ca	Oral Ca	Oral Ca	Oral Ca	Oral Ca	Oral Ca	Oral Ca	Oral Ca	Oral Ca	Oral Ca	Oral Ca
Body Site	Sa	Sa	Sa	Sa	Sa	Sa	Sa	Sa	Sa	Sa	Sa	Sa	Sa	Sa	Sa	Sa
Bacteria|Proteobacteria|Betaproteobacteria|Burkholderiales|Alcalig	0	0	0	0	0	0	0	0	0	0	0	0	0	0	0	0
Bacteria|Proteobacteria|Betaproteobacteria|Burkholderiales|Alcalig	0	0	0	0	0	0	0	0	0	0	0	0	0	0	0	0
Bacteria|Proteobacteria|Betaproteobacteria|Burkholderiales|Burkho	0	0	0	0	0	0	0	0	0	0	0	0	0	0	0	0
Bacteria|Proteobacteria|Betaproteobacteria|Burkholderiales|Burkho	79	521	411	362	292	81	7	1088	572	122	80	48	1264	243	1613	287
Bacteria|Proteobacteria|Betaproteobacteria|Burkholderiales|Burkho	0	0	0	0	0	0	0	0	0	0	0	0	0	0	0	0
Bacteria|Proteobacteria|Betaproteobacteria|Burkholderiales|Burkho	0	0	0	0	0	0	0	0	0	0	0	0	0	0	0	0
Bacteria|Proteobacteria|Betaproteobacteria|Burkholderiales|Coman	0	0	0	0	0	0	0	0	0	0	0	0	0	0	0	0
Bacteria|Proteobacteria|Betaproteobacteria|Burkholderiales|Coman	0	0	0	0	0	0	0	0	0	0	0	0	0	0	0	0
Bacteria|Proteobacteria|Betaproteobacteria|Burkholderiales|Coman	0	0	0	0	0	0	0	0	0	0	0	0	0	0	0	0
Bacteria|Proteobacteria|Betaproteobacteria|Burkholderiales|Coman	0	0	0	0	0	0	0	0	0	0	0	0	0	0	0	0
Bacteria|Proteobacteria|Betaproteobacteria|Burkholderiales|Coman	0	0	0	0	0	0	0	0	0	0	0	0	0	0	0	0
Bacteria|Proteobacteria|Betaproteobacteria|Burkholderiales|Coman	0	0	0	0	0	0	0	0	0	0	0	0	0	0	0	0
Bacteria|Proteobacteria|Betaproteobacteria|Burkholderiales|Coman	0	0	0	0	0	0	0	0	0	0	0	0	0	0	0	0
Bacteria|Proteobacteria|Betaproteobacteria|Burkholderiales|Coman	0	0	0	0	0	0	0	0	0	0	0	0	0	0	0	0
Bacteria|Proteobacteria|Betaproteobacteria|Burkholderiales|Coman	0	0	0	1	0	0	0	0	0	0	0	0	0	0	0	1
Bacteria|Proteobacteria|Betaproteobacteria|Burkholderiales|Coman	0	0	0	0	0	0	0	0	0	0	0	0	0	0	0	0
Bacteria|Proteobacteria|Betaproteobacteria|Burkholderiales|Coman	0	0	0	0	0	0	0	0	0	0	0	0	0	0	0	0
Bacteria|Proteobacteria|Betaproteobacteria|Burkholderiales|Coman	0	0	0	0	0	0	0	0	0	0	0	0	0	0	0	0
Bacteria|Proteobacteria|Betaproteobacteria|Burkholderiales|Coman	0	0	0	0	0	0	0	0	0	0	0	0	0	0	0	0
Bacteria|Proteobacteria|Betaproteobacteria|Burkholderiales|Coman	0	0	0	0	0	0	0	0	0	0	0	0	0	0	0	0
Bacteria|Proteobacteria|Betaproteobacteria|Burkholderiales|Coman	0	1	0	0	0	0	0	0	0	0	0	0	0	0	0	0
Bacteria|Proteobacteria|Betaproteobacteria|Burkholderiales|Coman	0	5	0	1	370	0	0	0	0	0	0	0	50	0	94	5
Bacteria|Proteobacteria|Betaproteobacteria|Burkholderiales|Coman	23	190	190	151	1065	96	3	44	379	144	26	10	192	220	957	60
Bacteria|Proteobacteria|Betaproteobacteria|Burkholderiales|Oxalob	0	0	0	0	1	0	0	0	0	0	0	0	1	0	2	0
Bacteria|Proteobacteria|Betaproteobacteria|Burkholderiales|Oxalob	0	0	0	0	0	0	0	0	0	0	0	0	0	0	0	0
Bacteria|Proteobacteria|Betaproteobacteria|Burkholderiales|Oxalob	0	0	0	0	0	0	0	0	0	0	0	0	0	0	0	0
Bacteria|Proteobacteria|Betaproteobacteria|Burkholderiales|Oxalob	0	0	0	0	0	0	0	0	0	0	0	0	0	0	0	1
Bacteria|Proteobacteria|Betaproteobacteria|Burkholderiales|Oxalob	0	0	0	1	1	0	0	0	0	0	0	0	0	0	2	0
Bacteria|Proteobacteria|Betaproteobacteria|Burkholderiales_Other	0	0	0	0	0	0	0	0	0	0	0	0	0	0	0	0
Bacteria|Proteobacteria|Betaproteobacteria|Methylophilales|Methy	0	0	0	0	0	0	0	0	0	0	0	0	0	0	0	0
Bacteria|Proteobacteria|Betaproteobacteria|Methylophilales|Methy	0	0	0	0	0	0	0	0	0	0	0	0	0	0	0	0
Bacteria|Proteobacteria|Betaproteobacteria|Methylophilales|Methy	0	0	0	0	0	0	0	0	0	0	0	0	0	0	0	0
Bacteria|Proteobacteria|Betaproteobacteria|Neisseriales|Neisseriace	30	79	402	153	97	116	17	16	86	76	18	38	219	121	250	83
Bacteria|Proteobacteria|Betaproteobacteria|Neisseriales|Neisseriace	43	16	17	25	2	15	27	66	10	16	3	11	0	0	21	20
Bacteria|Proteobacteria|Betaproteobacteria|Neisseriales|Neisseriace	8050	5272	12763	4594	2256	2885	1528	4837	9480	3041	4592	6588	2007	3345	4531	4217
Bacteria|Proteobacteria|Betaproteobacteria|Neisseriales|Neisseriace	8	8	72	30	4	32	1	1	15	4	7	6	66	10	34	46
Bacteria|Proteobacteria|Betaproteobacteria|Neisseriales|Neisseriace	841	878	588	518	360	57	79	719	542	298	160	63	23	446	1732	494

Microbial Clade																	
	Sample ID	C1.002	C1.003	C1.006	C1.009	C1.010	C1.012	C1.013	C1.014	C1.016	C1.017	C1.018	C1.019	C1.020	C1.021	C1.024	C1.025
	Group	Oral Ca	Oral Ca	Oral Ca	Oral Ca	Oral Ca	Oral Ca	Oral Ca	Oral Ca	Oral Ca	Oral Ca	Oral Ca	Oral Ca	Oral Ca	Oral Ca	Oral Ca	Oral Ca
	Body Site	Sa	Sa	Sa	Sa	Sa	Sa	Sa	Sa	Sa	Sa	Sa	Sa	Sa	Sa	Sa	Sa
Bacteria|Proteobacteria|Betaproteobacteria|Rhodocyclales|Rhodocy		0	0	0	0	0	0	0	0	0	0	0	0	0	0	0	0
Bacteria|Proteobacteria|Betaproteobacteria|Rhodocyclales|Rhodocy		0	0	0	0	0	0	0	0	0	0	0	0	0	0	0	0
Bacteria|Proteobacteria|Betaproteobacteria|Rhodocyclales|Rhodocy		0	0	0	0	0	0	0	0	0	0	0	0	0	0	1	0
Bacteria|Proteobacteria|Betaproteobacteria|Rhodocyclales|Rhodocy		0	4	17	2	2	0	1	0	0	0	0	0	4	1	13	0
Bacteria|Proteobacteria|Betaproteobacteria|Rhodocyclales|Rhodocy		0	0	0	0	0	0	0	0	0	0	0	0	0	0	0	0
Bacteria|Proteobacteria|Betaproteobacteria|Rhodocyclales|Rhodocy		0	0	0	0	0	0	0	0	0	0	0	0	0	0	0	0
Bacteria|Proteobacteria|Betaproteobacteria_Other		0	0	0	1	0	0	0	0	0	0	0	0	0	0	0	0
Bacteria|Proteobacteria|Deltaproteobacteria|Bdellovibrionales|Bact		0	0	0	0	0	0	0	0	0	0	0	0	0	0	0	0
Bacteria|Proteobacteria|Deltaproteobacteria|Bdellovibrionales|Bde		0	0	0	0	0	0	0	0	0	0	0	0	0	0	0	0
Bacteria|Proteobacteria|Deltaproteobacteria|Desulfobacterales|Desu		11	12	0	21	0	2	8	1	0	1	4	0	35	5	12	2
Bacteria|Proteobacteria|Deltaproteobacteria|Desulfovibrionales|De		0	0	0	0	0	0	0	0	0	2	21	0	0	0	0	0
Bacteria|Proteobacteria|Deltaproteobacteria|Desulfovibrionales|De		0	0	0	4	0	0	0	0	0	0	0	0	0	0	0	0
Bacteria|Proteobacteria|Deltaproteobacteria|Desulfovibrionales|De		0	5	0	8	61	0	74	0	5	0	38	0	11	18	0	3
Bacteria|Proteobacteria|Deltaproteobacteria|Desulfovibrionales|De		0	0	0	1	0	0	0	0	0	0	0	0	0	0	0	0
Bacteria|Proteobacteria|Deltaproteobacteria|Myxococcales|0319-6		0	0	0	0	0	0	0	0	0	0	0	0	0	0	0	0
Bacteria|Proteobacteria|Deltaproteobacteria|Myxococcales|Haliang		0	0	0	0	0	0	0	0	0	0	0	0	0	0	0	0
Bacteria|Proteobacteria|Deltaproteobacteria|Myxococcales|Myxocc		0	0	0	0	0	0	0	0	0	0	0	0	0	0	0	0
Bacteria|Proteobacteria|Deltaproteobacteria|Myxococcales_Other		0	0	0	0	0	0	0	0	0	0	0	0	0	0	0	0
Bacteria|Proteobacteria|Deltaproteobacteria|Syntrophobacterales|Sy		0	0	0	0	0	0	0	0	0	0	0	0	0	0	0	0
Bacteria|Proteobacteria|Epsilonproteobacteria|Campylobacterales|C		0	0	0	0	0	0	0	0	0	0	0	0	0	0	0	0
Bacteria|Proteobacteria|Epsilonproteobacteria|Campylobacterales|C		20	15	33	29	15	10	16	8	5	31	54	23	32	11	61	12
Bacteria|Proteobacteria|Epsilonproteobacteria|Campylobacterales|I		0	0	0	10	0	0	0	0	0	1	0	0	0	0	0	1
Bacteria|Proteobacteria|Epsilonproteobacteria|Campylobacterales|I		0	0	0	0	0	0	0	0	0	0	0	0	0	0	0	0
Bacteria|Proteobacteria|Epsilonproteobacteria|Campylobacterales|I		0	0	0	0	0	0	0	0	0	0	0	0	0	0	0	0
Bacteria|Proteobacteria|Gammaproteobacteria|Aeromonadales|Aer		0	0	0	0	0	0	0	0	0	0	0	0	0	0	0	0
Bacteria|Proteobacteria|Gammaproteobacteria|Aeromonadales|Aer		0	0	0	0	0	0	0	0	0	0	0	0	0	0	0	0
Bacteria|Proteobacteria|Gammaproteobacteria|Aeromonadales|Aer		0	0	0	0	0	1	0	0	0	0	0	0	0	0	0	1
Bacteria|Proteobacteria|Gammaproteobacteria|Aeromonadales|Suc		0	0	0	47	4	2	1	0	0	0	0	0	0	0	0	0
Bacteria|Proteobacteria|Gammaproteobacteria|Alteromonadales|[C		0	0	0	0	0	0	0	0	0	0	0	0	0	0	0	0
Bacteria|Proteobacteria|Gammaproteobacteria|Alteromonadales|[C		0	0	0	0	0	0	0	0	0	0	0	0	0	0	0	0
Bacteria|Proteobacteria|Gammaproteobacteria|Alteromonadales|[C		0	0	0	0	0	0	0	0	0	0	0	0	0	0	0	0
Bacteria|Proteobacteria|Gammaproteobacteria|Alteromonadales|Al		0	0	0	0	0	0	0	0	0	0	0	0	0	0	0	0
Bacteria|Proteobacteria|Gammaproteobacteria|Alteromonadales|Al		0	0	0	0	0	0	0	0	0	0	0	0	0	0	0	0
Bacteria|Proteobacteria|Gammaproteobacteria|Alteromonadales|Al		0	0	0	0	0	0	0	0	0	0	0	0	0	0	0	0
Bacteria|Proteobacteria|Gammaproteobacteria|Alteromonadales|Sh		0	0	0	0	0	0	0	0	0	0	0	0	0	0	0	0
Bacteria|Proteobacteria|Gammaproteobacteria|Cardiobacteriales|Ca		15	122	273	166	61	29	8	314	102	35	5	14	147	84	373	53
Bacteria|Proteobacteria|Gammaproteobacteria|Cardiobacteriales|Ca		4	0	0	58	19	0	0	0	0	0	0	0	0	2	106	41

Microbial Clade / Sample ID / Group / Body Site	C1.002	C1.003	C1.006	C1.009	C1.010	C1.012	C1.013	C1.014	C1.016	C1.017	C1.018	C1.019	C1.020	C1.021	C1.024	C1.025
Group	Oral Ca	Oral Ca	Oral Ca	Oral Ca	Oral Ca	Oral Ca	Oral Ca	Oral Ca	Oral Ca	Oral Ca	Oral Ca	Oral Ca	Oral Ca	Oral Ca	Oral Ca	Oral Ca
Body Site	Sa	Sa	Sa	Sa	Sa	Sa	Sa	Sa	Sa	Sa	Sa	Sa	Sa	Sa	Sa	Sa
Bacteria|Proteobacteria|Gammaproteobacteria|Cardiobacteriales_C	0	0	0	4	0	0	0	0	3	0	0	0	0	3	1	8
Bacteria|Proteobacteria|Gammaproteobacteria|Enterobacteriales|Er	0	0	0	0	0	0	0	0	0	0	0	0	0	0	0	0
Bacteria|Proteobacteria|Gammaproteobacteria|Enterobacteriales|Er	0	0	0	1	0	0	0	0	0	0	0	0	0	0	0	0
Bacteria|Proteobacteria|Gammaproteobacteria|Enterobacteriales|Er	0	0	0	0	0	0	0	0	0	0	0	0	0	0	1	1
Bacteria|Proteobacteria|Gammaproteobacteria|Enterobacteriales|Er	0	0	0	0	0	0	0	0	0	0	0	0	0	0	0	0
Bacteria|Proteobacteria|Gammaproteobacteria|Enterobacteriales|Er	0	0	0	0	0	0	0	0	0	0	0	0	0	0	0	0
Bacteria|Proteobacteria|Gammaproteobacteria|Enterobacteriales|Er	0	0	0	0	0	0	0	0	0	0	0	0	0	0	0	0
Bacteria|Proteobacteria|Gammaproteobacteria|Enterobacteriales|Er	0	0	0	1	0	0	0	0	0	0	0	0	0	0	0	0
Bacteria|Proteobacteria|Gammaproteobacteria|Enterobacteriales|Er	0	0	0	0	0	0	0	0	0	0	0	0	0	0	0	0
Bacteria|Proteobacteria|Gammaproteobacteria|Enterobacteriales|Er	0	0	0	0	0	0	0	0	0	0	0	0	8	0	0	0
Bacteria|Proteobacteria|Gammaproteobacteria|Enterobacteriales|Er	0	0	0	0	0	0	1	0	0	0	0	0	0	0	2	2
Bacteria|Proteobacteria|Gammaproteobacteria|Enterobacteriales|Er	0	3	0	32	8	56	2	0	0	1	7	0	6	0	46	8
Bacteria|Proteobacteria|Gammaproteobacteria|Legionellales|Coxiel	0	0	0	0	0	0	0	0	0	0	0	0	0	0	0	0
Bacteria|Proteobacteria|Gammaproteobacteria|Legionellales|Legion	0	0	0	0	0	0	0	0	0	0	0	0	0	0	0	0
Bacteria|Proteobacteria|Gammaproteobacteria|Legionellales|Legion	0	0	0	0	0	0	0	0	0	0	0	0	0	0	0	0
Bacteria|Proteobacteria|Gammaproteobacteria|Legionellales|Legion	0	0	0	0	0	0	0	0	0	0	0	0	0	0	0	0
Bacteria|Proteobacteria|Gammaproteobacteria|Oceanospirillales|Al	0	0	0	0	0	0	0	0	0	0	0	0	0	0	0	0
Bacteria|Proteobacteria|Gammaproteobacteria|Oceanospirillales|Ha	0	0	0	0	0	0	0	0	0	0	0	0	0	0	0	0
Bacteria|Proteobacteria|Gammaproteobacteria|Pasteurellales|Pasteu	28	37	12	18	14	1	11	30	3	165	25	22	23	17	62	5
Bacteria|Proteobacteria|Gammaproteobacteria|Pasteurellales|Pasteu	239	77	553	68	58	30	142	33	160	9	61	60	19	94	146	16
Bacteria|Proteobacteria|Gammaproteobacteria|Pasteurellales|Pasteu	419	453	704	120	194	20	94	449	177	310	139	286	1794	263	99	91
Bacteria|Proteobacteria|Gammaproteobacteria|Pasteurellales|Pasteu	0	0	0	0	0	0	0	0	0	0	0	0	0	0	0	0
Bacteria|Proteobacteria|Gammaproteobacteria|Pasteurellales|Pasteu	0	0	0	0	0	0	0	0	0	0	0	0	0	0	0	0
Bacteria|Proteobacteria|Gammaproteobacteria|Pasteurellales|Pasteu	0	1	0	2	1	1	0	0	0	0	1	0	0	0	0	1
Bacteria|Proteobacteria|Gammaproteobacteria|Pasteurellales|Pasteu	0	0	0	0	0	0	0	0	0	0	0	0	0	0	0	0
Bacteria|Proteobacteria|Gammaproteobacteria|Pseudomonadales|M	0	0	0	0	0	0	0	0	0	0	0	0	0	0	4	0
Bacteria|Proteobacteria|Gammaproteobacteria|Pseudomonadales|M	0	0	0	0	0	0	0	0	0	0	0	0	0	0	0	0
Bacteria|Proteobacteria|Gammaproteobacteria|Pseudomonadales|M	4	0	0	3	0	0	0	0	0	0	0	0	0	0	3	1
Bacteria|Proteobacteria|Gammaproteobacteria|Pseudomonadales|M	12	5	83	3	71	0	0	0	3	0	24	3	5	0	66	22
Bacteria|Proteobacteria|Gammaproteobacteria|Pseudomonadales|M	0	0	0	0	0	0	0	0	0	0	0	0	0	0	0	0
Bacteria|Proteobacteria|Gammaproteobacteria|Pseudomonadales|M	0	0	0	0	0	0	0	0	0	0	0	0	0	0	0	0
Bacteria|Proteobacteria|Gammaproteobacteria|Pseudomonadales|M	0	0	0	0	0	0	0	0	0	0	0	0	0	0	2	0
Bacteria|Proteobacteria|Gammaproteobacteria|Pseudomonadales|M	0	0	0	0	0	0	0	0	0	0	0	0	0	0	0	1
Bacteria|Proteobacteria|Gammaproteobacteria|Pseudomonadales|Ps	0	0	0	0	3	0	0	1	1	0	0	0	0	0	19	7
Bacteria|Proteobacteria|Gammaproteobacteria|Pseudomonadales|Ps	0	0	0	0	0	0	0	0	0	0	0	0	0	0	1	0
Bacteria|Proteobacteria|Gammaproteobacteria|Pseudomonadales|Ps	0	0	0	0	0	0	0	0	0	0	0	0	0	0	1	0
Bacteria|Proteobacteria|Gammaproteobacteria|Pseudomonadales_C	0	0	0	0	0	0	0	0	0	0	0	0	0	0	0	0

Microbial Clade	Sample ID	C1.002	C1.003	C1.006	C1.009	C1.010	C1.012	C1.013	C1.014	C1.016	C1.017	C1.018	C1.019	C1.020	C1.021	C1.024	C1.025
	Group	Oral Ca	Oral Ca	Oral Ca	Oral Ca	Oral Ca	Oral Ca	Oral Ca	Oral Ca	Oral Ca	Oral Ca	Oral Ca	Oral Ca	Oral Ca	Oral Ca	Oral Ca	Oral Ca
	Body Site	Sa	Sa	Sa	Sa	Sa	Sa	Sa	Sa	Sa	Sa	Sa	Sa	Sa	Sa	Sa	Sa
Bacteria|Proteobacteria|Gammaproteobacteria|Salinisphaerales|Sal		0	0	0	0	0	0	0	0	0	0	0	0	0	0	0	0
Bacteria|Proteobacteria|Gammaproteobacteria|Vibrionales|Pseudoa		0	0	0	0	0	0	0	0	0	0	0	0	0	0	0	0
Bacteria|Proteobacteria|Gammaproteobacteria|Vibrionales|Vibrion		0	0	0	0	0	0	0	0	0	0	0	0	0	0	0	0
Bacteria|Proteobacteria|Gammaproteobacteria|Xanthomonadales|S		0	0	0	0	0	0	0	0	0	0	0	0	0	0	0	0
Bacteria|Proteobacteria|Gammaproteobacteria|Xanthomonadales|S		0	0	0	0	0	0	0	0	0	0	0	0	0	0	0	0
Bacteria|Proteobacteria|Gammaproteobacteria|Xanthomonadales|S		0	0	0	1	0	0	0	0	0	0	0	0	0	0	0	0
Bacteria|Proteobacteria|Gammaproteobacteria|Xanthomonadales|X		0	0	0	0	0	0	0	0	0	0	0	0	0	0	0	0
Bacteria|Proteobacteria|Gammaproteobacteria|Xanthomonadales|X		0	0	0	0	0	0	0	0	0	0	0	0	0	0	0	0
Bacteria|Proteobacteria|Gammaproteobacteria|Xanthomonadales|X		0	0	0	0	0	0	0	0	0	0	0	0	0	0	0	0
Bacteria|Proteobacteria|Gammaproteobacteria|Xanthomonadales|X		0	0	0	0	0	0	0	0	0	0	0	0	0	0	0	0
Bacteria|Proteobacteria|Gammaproteobacteria|Xanthomonadales|X		0	0	0	0	0	0	0	0	0	0	0	0	0	0	0	0
Bacteria|Proteobacteria|Gammaproteobacteria|Xanthomonadales|X		0	0	1	1	0	2	0	0	0	0	2	0	0	0	10	0
Bacteria|Proteobacteria|Gammaproteobacteria|Xanthomonadales|X		0	0	0	0	0	0	0	0	0	0	0	0	0	0	0	0
Bacteria|Proteobacteria|Gammaproteobacteria|Xanthomonadales|X		0	0	0	0	0	0	0	0	0	0	0	0	0	0	0	0
Bacteria|Proteobacteria|Gammaproteobacteria|Xanthomonadales|X		0	0	0	0	0	0	0	0	0	0	0	0	0	0	0	1
Bacteria|Proteobacteria|Gammaproteobacteria_Other		0	0	0	0	0	0	0	0	0	0	0	0	0	0	0	0
Bacteria|Proteobacteria_Other		0	0	0	0	0	0	0	0	0	0	0	0	0	0	1	0
Bacteria|Spirochaetes|Spirochaetes|Sphaerochaetales|Sphaerochaet		0	0	0	0	0	0	2	0	0	0	5	0	0	2	0	0
Bacteria|Spirochaetes|Spirochaetes|Spirochaetales|Spirochaetaceae		100	156	59	114	131	0	215	5	15	36	254	2	89	21	163	21
Bacteria|Synergistetes|Synergistia|Synergistales|Dethiosulfovibrior		1	1	0	0	0	0	3	4	0	0	5	0	1	1	0	1
Bacteria|Synergistetes|Synergistia|Synergistales|Dethiosulfovibrior		21	42	15	51	167	2	74	0	6	3	103	0	23	14	70	10
Bacteria|Synergistetes|Synergistia|Synergistales|Synergistaceae|Car		0	0	0	0	0	0	0	0	0	0	0	0	0	0	0	0
Bacteria|Synergistetes|Synergistia|Synergistales|Synergistaceae_un		0	0	0	0	0	0	0	0	0	0	0	0	0	0	0	0
Bacteria|Tenericutes|Mollicutes|Acholeplasmatales|Acholeplasmata		0	0	0	6	0	0	0	0	0	1	0	0	0	0	0	0
Bacteria|Tenericutes|Mollicutes|Mycoplasmatales|Mycoplasmatace		4	0	0	1	26	0	4	1	0	0	29	0	1	0	32	6
Bacteria|Tenericutes|Mollicutes|Mycoplasmatales|Mycoplasmatace		0	0	0	0	0	0	0	0	0	0	0	0	0	0	2	2
Bacteria|Thermotogae|Thermotogae|Thermotogales|Thermotogacea		0	0	0	0	0	0	0	0	0	0	0	0	0	0	0	0
Bacteria|Thermotogae|Thermotogae|Thermotogales|Thermotogacea		0	0	0	0	0	0	0	0	0	0	0	0	0	0	0	0
Bacteria|TM7|TM7-3|CW040|F16_unclassified		1	2	1	0	1	1	1	1	0	4	3	4	1	2	0	0
Bacteria|TM7|TM7-3|I025|Rs-045_unclassified		5	15	13	55	147	15	105	59	1	31	242	0	24	17	191	30
Bacteria|Verrucomicrobia|Verruco-5|WCHB1-41|RFP12_unclassi		0	0	0	1	0	0	0	0	0	0	0	0	0	0	0	0
Bacteria|Verrucomicrobia|Verrucomicrobiae|Verrucomicrobiales|V		0	0	0	0	0	0	0	0	0	0	0	0	0	0	0	0

C1.026	C1.027	C1.029	C1.030	C1.031	C1.032	C1.033	C1.034	C1.036	C1.037	C1.038	C2.002	C2.003	C2.006	C2.009	C2.010	C2.012	C2.013	C2.014	C2.016	C2.017	C2.018	C2.019
Oral Ca	Oral Ca	Oral Ca	Oral Ca	Oral Ca	Oral Ca	Oral Ca	Oral Ca	Oral Ca	Oral Ca	Oral Ca	Oral Ca	Oral Ca	Oral Ca	Oral Ca	Oral Ca	Oral Ca	Oral Ca	Oral Ca	Oral Ca	Oral Ca	Oral Ca	Oral Ca
Sa	Sa	Sa	Sa	Sa	Sa	Sa	Sa	Sa	Sa	Sa	TD	TD	TD	TD	TD	TD	TD	TD	TD	TD	TD	TD
0	0	0	0	0	0	0	0	0	0	0	0	0	0	0	0	0	0	0	0	0	0	0
13137	19900	20917	18700	18462	14829	17895	17233	18961	11738	20254	20653	16786	22660	10202	16262	7464	9761	20464	17624	21293	20447	18945
32	211	124	1	1	0	6	3	8	2	294	4	2	1	12	367	42	1	1	3	1	0	0
0	0	1	0	0	0	0	0	0	0	0	0	0	0	0	0	0	0	0	0	0	0	0
0	0	3	0	0	0	0	0	0	0	0	0	0	0	1	0	0	0	0	0	0	0	0
389	803	3342	1344	3600	1523	5059	3159	863	3813	1791	835	1409	399	378	319	416	984	2485	446	3305	392	2259
0	0	1	0	0	0	0	0	0	0	0	0	0	0	0	0	0	0	0	0	0	0	0
6373	10540	4900	8203	6709	6023	4806	6378	5462	2752	7987	4427	5454	5924	1415	3318	1951	3131	8261	3161	3977	2228	6615
0	0	0	0	0	0	0	0	0	0	0	0	0	0	0	0	0	0	0	0	0	0	0
0	0	0	0	0	0	0	0	0	0	0	0	0	0	0	0	0	1	0	0	0	0	0
0	0	0	0	0	0	0	0	0	0	0	0	0	0	0	0	0	0	0	0	0	0	0
0	1	0	0	0	1	0	0	1	1	0	0	0	0	0	0	0	0	0	0	0	0	0
0	0	2	0	0	0	0	0	0	0	0	0	0	0	0	0	0	0	0	0	0	0	0
0	0	0	0	0	0	0	0	0	0	0	1	0	0	0	0	0	1	0	0	0	0	0
0	1	0	0	0	0	0	0	0	0	0	0	0	0	1	0	0	0	0	0	0	0	0
862	875	1292	966	1106	928	1544	1945	1451	1372	2298	3419	2849	2268	2676	1910	1650	4908	6352	3045	8353	3941	5525
126	223	49	28	24	21	79	44	47	11	76	655	1104	751	122	1314	396	206	276	659	373	889	693
0	0	0	0	0	0	0	0	0	0	0	0	0	0	0	0	0	0	0	0	0	0	0
7	7	1	1	1	0	1	1	4	0	0	2	1	5	5	20	7	1	0	0	1	0	0
0	0	0	0	0	0	0	0	0	0	0	0	0	0	0	0	0	0	0	0	0	0	0
0	0	0	0	0	0	0	0	0	0	0	0	0	0	0	0	0	0	0	0	0	0	0
5048	4913	10703	7802	6527	5967	5946	5314	10714	3395	7133	10552	5431	12861	5355	8503	2794	410	2964	8708	5227	12790	2759
70	289	1	121	187	122	169	144	212	89	177	22	16	6	23	31	0	34	2	11	10	48	3
0	1	0	5	4	1	1	3	8	0	8	20	30	131	0	43	0	8	0	606	0	2	6
17	124	0	12	87	52	46	36	31	39	26	0	2	0	0	0	0	3	0	1	1	0	0
9	13	3	4	4	7	1	3	0	3	0	17	12	7	27	19	4	19	2	1	5	5	8
0	0	0	0	0	0	0	0	0	0	0	0	0	0	0	0	0	0	0	0	0	0	0
0	0	0	0	0	0	0	0	0	0	0	0	0	0	0	0	0	0	0	0	0	0	0
204	1897	495	213	212	184	237	202	160	261	464	699	476	307	187	418	204	54	121	983	40	152	1077
0	0	0	0	0	0	0	0	0	0	0	0	0	0	0	0	0	0	0	0	0	0	0
0	0	0	0	0	0	0	0	0	0	0	0	0	0	0	0	0	0	0	0	0	0	0
0	2	0	0	0	0	0	1	0	0	0	0	0	0	0	0	0	0	0	0	0	0	0
0	0	1	0	0	0	0	0	0	0	0	0	0	0	0	0	0	0	0	0	0	0	0
0	0	0	0	0	0	0	0	0	0	0	0	0	0	0	0	0	0	0	0	0	0	0
0	0	0	0	0	0	0	0	0	0	0	0	0	0	0	0	0	0	0	0	0	0	0
0	0	0	0	0	0	0	0	0	0	0	0	0	0	0	0	0	0	0	0	0	0	0

C1.026	C1.027	C1.029	C1.030	C1.031	C1.032	C1.033	C1.034	C1.036	C1.037	C1.038	C2.002	C2.003	C2.006	C2.009	C2.010	C2.012	C2.013	C2.014	C2.016	C2.017	C2.018	C2.019
Oral Ca	Oral Ca	Oral Ca	Oral Ca	Oral Ca	Oral Ca	Oral Ca	Oral Ca	Oral Ca	Oral Ca	Oral Ca	Oral Ca	Oral Ca	Oral Ca	Oral Ca	Oral Ca	Oral Ca	Oral Ca	Oral Ca	Oral Ca	Oral Ca	Oral Ca	Oral Ca
Sa	Sa	Sa	Sa	Sa	Sa	Sa	Sa	Sa	Sa	Sa	TD	TD	TD	TD	TD	TD	TD	TD	TD	TD	TD	TD
0	0	0	0	0	0	0	0	0	0	0	0	0	0	0	0	0	0	0	0	0	0	0
0	0	3	0	0	0	0	0	0	0	0	0	0	0	1	0	0	0	0	0	0	0	0
0	0	0	0	0	0	0	0	0	0	0	0	0	0	0	0	0	0	0	0	0	0	0
0	0	0	0	0	0	0	0	0	0	0	0	0	0	0	0	0	0	0	0	0	0	0
382	761	3286	1314	3549	1485	4921	3015	839	3583	1710	792	1369	389	366	278	390	959	2251	390	3249	382	1987
7	42	56	30	51	38	138	144	24	230	81	43	40	10	12	41	26	25	234	56	56	10	272
0	0	0	0	0	0	0	0	0	0	0	0	0	0	0	0	0	0	0	0	0	0	0
0	0	0	0	0	0	0	0	0	0	0	0	0	0	0	0	0	0	0	0	0	0	0
0	0	0	0	0	0	0	0	0	0	0	0	0	0	0	0	0	0	0	0	0	0	0
0	0	1	0	0	0	0	0	0	0	0	0	0	0	0	0	0	0	0	0	0	0	0
0	0	0	0	0	0	0	0	0	0	0	0	0	0	0	0	0	0	0	0	0	0	0
0	0	39	0	0	0	0	0	0	0	0	0	0	0	1	0	0	0	0	0	0	0	0
6218	10270	4820	8161	6653	5963	4782	6351	5375	2725	7895	4320	5360	5740	1330	3212	1869	3079	8221	3097	3939	2079	6529
0	0	1	0	0	0	0	0	0	0	0	0	0	0	0	0	0	0	0	0	0	0	0
154	270	36	42	56	60	24	27	87	27	92	107	94	184	84	104	82	52	40	64	38	149	86
1	0	4	0	0	0	0	0	0	0	0	0	0	0	0	2	0	0	0	0	0	0	0
0	0	0	0	0	0	0	0	0	0	0	0	0	0	0	0	0	0	0	0	0	0	0
0	0	0	0	0	0	0	0	0	0	0	0	0	0	0	0	0	1	0	0	0	0	0
0	0	0	0	0	0	0	0	0	0	0	0	0	0	0	0	0	0	0	0	0	0	0
0	0	0	0	0	0	0	0	0	0	0	0	0	0	0	0	0	0	0	0	0	0	0
0	1	0	0	0	1	0	0	1	1	0	0	0	0	0	0	0	0	0	0	0	0	0
0	0	0	0	0	0	0	0	0	0	0	0	0	0	0	0	0	0	0	0	0	0	0
0	0	0	0	0	0	0	0	0	0	0	0	0	0	0	0	0	0	0	0	0	0	0
0	0	0	0	0	0	0	0	0	0	0	0	0	0	0	0	0	0	0	0	0	0	0
0	0	0	0	0	0	0	0	0	0	0	0	0	0	0	0	0	0	0	0	0	0	0
0	0	0	0	0	0	0	0	0	0	0	0	0	0	0	0	0	0	0	0	0	0	0
0	0	0	0	0	0	0	0	0	0	0	0	0	0	0	0	0	0	0	0	0	0	0
0	0	1	0	0	0	0	0	0	0	0	0	0	0	0	0	0	0	0	0	0	0	0
0	0	1	0	0	0	0	0	0	0	0	0	0	0	0	0	0	0	0	0	0	0	0
0	0	0	0	0	0	0	0	0	0	0	0	0	0	0	0	0	0	0	0	0	0	0
0	0	0	0	0	0	0	0	0	0	0	0	0	0	0	0	0	1	0	0	0	0	0
0	0	0	0	0	0	0	0	0	0	0	1	0	0	0	0	0	0	0	0	0	0	0
0	1	0	0	0	0	0	0	0	0	0	0	0	0	1	0	0	0	0	0	0	0	0
456	249	875	455	533	490	369	702	1119	639	946	1875	1061	1065	1689	1016	944	2671	2234	1484	5427	2250	2167
392	617	406	488	557	424	1134	1194	314	690	1254	1421	1674	1105	900	870	633	2071	3267	1475	2686	1559	3097
14	9	11	23	16	14	41	49	18	43	98	123	114	98	87	24	73	166	851	86	240	132	261
126	223	49	28	24	21	79	44	47	11	76	655	1104	751	122	1314	396	206	276	659	373	889	693

C1.026	C1.027	C1.029	C1.030	C1.031	C1.032	C1.033	C1.034	C1.036	C1.037	C1.038	C2.002	C2.003	C2.006	C2.009	C2.010	C2.012	C2.013	C2.014	C2.016	C2.017	C2.018	C2.019
Oral Ca	Oral Ca	Oral Ca	Oral Ca	Oral Ca	Oral Ca	Oral Ca	Oral Ca	Oral Ca	Oral Ca	Oral Ca	Oral Ca	Oral Ca	Oral Ca	Oral Ca	Oral Ca	Oral Ca	Oral Ca	Oral Ca	Oral Ca	Oral Ca	Oral Ca	Oral Ca
Sa	Sa	Sa	Sa	Sa	Sa	Sa	Sa	Sa	Sa	Sa	TD	TD	TD	TD	TD	TD	TD	TD	TD	TD	TD	TD
0	0	0	0	0	0	0	0	0	0	0	0	0	0	0	0	0	0	0	0	0	0	0
7	7	1	1	1	0	1	1	4	0	0	2	1	5	5	20	7	1	0	0	1	0	0
0	0	0	0	0	0	0	0	0	0	0	0	0	0	0	0	0	0	0	0	0	0	0
0	0	0	0	0	0	0	0	0	0	0	0	0	0	0	0	0	0	0	0	0	0	0
0	0	0	0	0	0	0	0	0	0	0	0	0	0	0	0	0	0	0	0	0	0	0
0	0	0	0	0	0	0	0	0	0	0	0	0	0	0	0	0	0	0	0	0	0	0
43	99	297	1	2	0	1	1	9	0	0	2	1	1	3	7	15	2	2	39	110	76	31
4631	4056	10005	7153	6141	5615	5594	4712	8990	3356	5715	9560	4391	10493	4261	3180	2454	178	2129	8143	3751	12030	1948
20	221	5	1	44	34	11	49	14	4	3	3	4	0	3	1	0	2	0	0	1	2	0
33	60	10	14	22	13	25	29	20	11	38	274	144	85	137	50	198	186	203	70	324	201	531
321	477	386	633	318	305	315	523	1681	24	1377	713	891	2282	951	5265	127	42	630	456	1041	481	249
0	0	0	0	0	0	0	0	0	0	0	0	0	0	0	0	0	0	0	0	0	0	0
0	0	0	0	0	0	0	1	0	0	0	0	0	0	0	0	0	0	0	0	0	0	0
70	289	1	121	187	122	169	143	212	89	177	22	16	6	23	31	0	34	2	11	10	48	3
17	124	0	12	87	52	46	36	31	39	26	0	2	0	0	0	0	3	0	1	1	0	0
0	0	0	0	0	0	0	0	0	0	0	0	0	0	0	0	0	0	0	0	0	0	0
8	13	3	4	4	7	0	2	0	3	0	17	12	7	26	19	4	19	2	1	5	4	8
1	0	0	0	0	0	1	1	0	0	0	0	0	0	1	0	0	0	0	0	0	1	0
0	0	0	0	0	0	0	0	0	0	0	0	0	0	0	0	0	0	0	0	0	0	0
0	0	0	0	0	0	0	0	0	0	0	0	0	0	0	0	0	0	0	0	0	0	0
0	0	0	0	0	0	0	0	0	0	0	0	0	0	0	0	0	0	0	0	0	0	0
0	0	0	0	0	0	0	0	0	0	0	0	0	0	0	0	0	0	0	0	0	0	0
0	0	0	0	0	0	0	0	0	0	0	0	0	0	0	0	0	0	0	0	0	0	0
204	1897	495	213	212	184	237	202	160	261	464	699	476	307	187	418	204	54	121	983	40	152	1077
0	0	0	0	0	0	0	0	0	0	0	0	0	0	0	0	0	0	0	0	0	0	0
0	0	0	0	0	0	0	0	0	0	0	0	0	0	0	0	0	0	0	0	0	0	0
0	2	0	0	0	0	0	1	0	0	0	0	0	0	0	0	0	0	0	0	0	0	0
0	0	1	0	0	0	0	0	0	0	0	0	0	0	0	0	0	0	0	0	0	0	0
0	0	0	0	0	0	0	0	0	0	0	0	0	0	0	0	0	0	0	0	0	0	0
0	0	0	0	0	0	0	0	0	0	0	0	0	0	0	0	0	0	0	0	0	0	0
0	0	0	0	0	0	0	0	0	0	0	0	0	0	0	0	0	0	0	0	0	0	0
0	0	0	0	0	0	0	0	0	0	0	0	0	0	0	0	0	0	0	0	0	0	0
0	0	0	0	0	0	0	0	0	0	0	0	0	0	0	0	0	0	0	0	0	0	0
0	0	3	0	0	0	0	0	0	0	0	0	0	0	1	0	0	0	0	0	0	0	0
0	0	0	0	0	0	0	0	0	0	0	0	0	0	0	0	0	0	0	0	0	0	0
0	0	0	0	0	0	0	0	0	0	0	0	0	0	0	0	0	0	0	0	0	0	0
373	558	3239	1314	3545	1481	4921	3002	823	3571	1707	778	1369	389	360	76	321	919	2249	389	3204	381	1979

C1.026	C1.027	C1.029	C1.030	C1.031	C1.032	C1.033	C1.034	C1.036	C1.037	C1.038	C2.002	C2.003	C2.006	C2.009	C2.010	C2.012	C2.013	C2.014	C2.016	C2.017	C2.018	C2.019
Oral Ca	Oral Ca	Oral Ca	Oral Ca	Oral Ca	Oral Ca	Oral Ca	Oral Ca	Oral Ca	Oral Ca	Oral Ca	Oral Ca	Oral Ca	Oral Ca	Oral Ca	Oral Ca	Oral Ca	Oral Ca	Oral Ca	Oral Ca	Oral Ca	Oral Ca	Oral Ca
Sa	Sa	Sa	Sa	Sa	Sa	Sa	Sa	Sa	Sa	Sa	TD	TD	TD	TD	TD	TD	TD	TD	TD	TD	TD	TD
9	203	47	0	4	4	0	13	16	12	3	14	0	0	6	202	69	40	2	1	45	1	8
0	0	0	0	0	0	0	0	0	0	0	0	0	0	0	0	0	0	0	0	0	0	0
7	42	56	30	51	38	138	144	24	230	81	43	40	10	12	41	26	25	234	56	56	10	272
0	0	0	0	0	0	0	0	0	0	0	0	0	0	0	0	0	0	0	0	0	0	0
0	0	0	0	0	0	0	0	0	0	0	0	0	0	0	0	0	0	0	0	0	0	0
0	0	0	0	0	0	0	0	0	0	0	0	0	0	0	0	0	0	0	0	0	0	0
0	0	0	0	0	0	0	0	0	0	0	0	0	0	0	0	0	0	0	0	0	0	0
0	0	1	0	0	0	0	0	0	0	0	0	0	0	0	0	0	0	0	0	0	0	0
0	0	0	0	0	0	0	0	0	0	0	0	0	0	0	0	0	0	0	0	0	0	0
0	0	39	0	0	0	0	0	0	0	0	0	0	0	1	0	0	0	0	0	0	0	0
6218	10270	4820	8161	6653	5963	4782	6351	5375	2725	7895	4320	5360	5740	1330	3212	1869	3079	8221	3097	3939	2079	6529
0	0	1	0	0	0	0	0	0	0	0	0	0	0	0	0	0	0	0	0	0	0	0
154	270	36	42	56	60	24	27	87	27	92	107	94	184	84	104	82	52	40	64	38	149	86
1	0	4	0	0	0	0	0	0	0	0	0	0	0	0	2	0	0	0	0	0	0	0
0	0	0	0	0	0	0	0	0	0	0	0	0	0	0	0	0	1	0	0	0	0	0
0	0	0	0	0	0	0	0	0	0	0	0	0	0	0	0	0	0	0	0	0	0	0
0	1	0	0	0	1	0	0	1	1	0	0	0	0	0	0	0	0	0	0	0	0	0
0	0	0	0	0	0	0	0	0	0	0	0	0	0	0	0	0	0	0	0	0	0	0
0	0	0	0	0	0	0	0	0	0	0	0	0	0	0	0	0	0	0	0	0	0	0
0	0	0	0	0	0	0	0	0	0	0	0	0	0	0	0	0	0	0	0	0	0	0
0	0	0	0	0	0	0	0	0	0	0	0	0	0	0	0	0	0	0	0	0	0	0
0	0	0	0	0	0	0	0	0	0	0	0	0	0	0	0	0	0	0	0	0	0	0
0	0	0	0	0	0	0	0	0	0	0	0	0	0	0	0	0	0	0	0	0	0	0
0	0	0	0	0	0	0	0	0	0	0	0	0	0	0	0	0	0	0	0	0	0	0
0	0	0	0	0	0	0	0	0	0	0	0	0	0	0	0	0	0	0	0	0	0	0
0	0	1	0	0	0	0	0	0	0	0	0	0	0	0	0	0	0	0	0	0	0	0
0	0	0	0	0	0	0	0	0	0	0	0	0	0	0	0	0	0	0	0	0	0	0
0	0	0	0	0	0	0	0	0	0	0	0	0	0	0	0	0	0	0	0	0	0	0
0	0	0	0	0	0	0	0	0	0	0	0	0	0	0	0	0	1	0	0	0	0	0
0	1	0	0	0	0	0	0	0	0	0	0	0	0	1	0	0	0	0	0	0	0	0
0	13	15	0	0	0	0	0	0	0	0	0	0	0	1	10	7	0	0	0	0	0	0
7	14	26	7	4	2	9	15	9	10	5	117	65	57	20	28	17	123	7	58	67	121	23
449	222	829	448	529	488	360	687	1110	629	940	1758	996	1008	1668	978	920	2548	2227	1426	5360	2129	2144
0	0	0	0	0	0	0	0	0	0	0	0	0	0	0	0	0	0	0	0	0	0	0
392	617	406	488	557	424	1134	1194	314	690	1254	1421	1674	1105	900	870	633	2071	3267	1475	2686	1559	3097
0	0	0	0	0	0	0	0	0	0	0	0	0	0	0	0	0	0	0	0	0	0	0
0	0	0	0	0	0	0	0	0	0	0	0	0	0	0	0	0	0	0	0	0	0	0

C1.026	C1.027	C1.029	C1.030	C1.031	C1.032	C1.033	C1.034	C1.036	C1.037	C1.038	C2.002	C2.003	C2.006	C2.009	C2.010	C2.012	C2.013	C2.014	C2.016	C2.017	C2.018	C2.019
Oral Ca	Oral Ca	Oral Ca	Oral Ca	Oral Ca	Oral Ca	Oral Ca	Oral Ca	Oral Ca	Oral Ca	Oral Ca	Oral Ca	Oral Ca	Oral Ca	Oral Ca	Oral Ca	Oral Ca	Oral Ca	Oral Ca	Oral Ca	Oral Ca	Oral Ca	Oral Ca
Sa	Sa	Sa	Sa	Sa	Sa	Sa	Sa	Sa	Sa	Sa	TD	TD	TD	TD	TD	TD	TD	TD	TD	TD	TD	TD
14	9	11	23	16	14	41	49	18	43	98	123	114	98	87	24	73	166	851	86	240	132	261
126	223	49	28	24	21	79	44	47	11	76	655	1104	751	122	1314	396	206	276	659	373	889	693
0	0	0	0	0	0	0	0	0	0	0	0	0	0	0	0	0	0	0	0	0	0	0
0	0	0	0	0	0	0	0	0	0	0	0	0	0	0	0	0	0	0	0	0	0	0
0	0	0	0	0	0	0	0	0	0	0	0	0	0	0	0	0	0	0	0	0	0	0
0	0	0	0	0	0	0	0	0	0	0	0	0	0	0	0	0	0	0	0	0	0	0
0	0	0	0	0	0	0	0	0	0	0	0	0	0	0	0	0	0	0	0	0	0	0
0	0	0	0	0	0	0	0	0	0	0	0	0	0	0	0	0	0	0	0	0	0	0
1	20	51	1	1	0	1	0	4	0	0	0	0	0	2	3	3	0	1	39	109	75	29
0	0	0	0	0	0	0	0	0	0	0	0	0	0	0	0	0	0	0	0	0	0	0
0	0	0	0	0	0	0	0	0	0	0	0	0	0	0	0	0	0	0	0	0	0	0
0	6	57	0	1	0	0	1	2	0	0	1	0	0	1	4	4	1	0	0	0	1	1
0	0	3	0	0	0	0	0	0	0	0	0	0	0	0	0	1	0	0	0	0	0	0
0	0	12	0	0	0	0	0	1	0	0	1	0	0	0	0	0	0	1	0	0	0	0
0	0	0	0	0	0	0	0	0	0	0	0	0	0	0	0	0	0	0	0	0	0	0
42	73	174	0	0	0	0	0	2	0	0	0	1	1	0	0	7	1	0	0	1	0	1
0	0	0	0	0	0	0	0	0	0	0	0	0	0	0	0	0	0	0	0	0	0	0
327	1673	682	338	254	242	544	335	990	240	181	8	12	9	13	37	35	1	65	9	77	8	11
0	0	0	0	0	0	0	0	0	0	0	0	0	0	0	0	0	0	0	0	0	0	0
0	0	0	0	0	0	0	0	0	0	0	0	0	0	0	0	0	0	0	0	0	0	0
4304	2344	9320	6814	5880	5370	5047	4377	7989	3116	5534	9552	4379	10484	4248	3143	2419	177	2064	8134	3674	12022	1937
0	35	0	1	6	3	3	0	11	0	0	0	0	0	0	0	0	0	0	0	0	0	0
0	0	1	0	0	0	0	0	0	0	0	0	0	0	0	0	0	0	0	0	0	0	0
0	0	0	0	0	0	0	0	0	0	0	0	0	0	0	0	0	0	0	0	0	0	0
0	0	3	0	0	0	0	0	0	0	0	0	0	0	0	0	0	0	0	0	0	0	0
1	1	0	0	11	11	11	21	14	4	3	3	4	0	0	0	0	1	0	0	0	0	0
19	219	0	1	31	23	0	21	0	0	0	0	0	0	0	1	0	1	0	0	1	2	0
0	0	0	0	0	0	0	0	0	0	0	0	0	0	0	0	0	0	0	0	0	0	0
0	1	0	0	2	0	0	1	0	0	0	0	0	0	3	0	0	0	0	0	0	0	0
0	0	0	0	0	0	0	0	0	0	0	0	0	0	0	0	0	0	0	0	0	0	0
0	0	2	0	0	0	0	6	0	0	0	0	0	0	0	0	0	0	0	0	0	0	0
0	0	0	0	0	0	0	0	0	0	0	0	0	0	0	0	0	0	0	0	0	0	0
0	0	0	0	0	0	0	0	0	0	0	0	0	0	0	0	0	0	0	0	0	0	0
33	60	10	14	22	13	25	29	20	11	38	274	144	85	137	50	198	186	203	70	324	201	531
4	1	1	0	0	0	0	0	0	0	0	0	0	0	1	0	0	0	0	0	0	0	0
0	0	0	0	0	1	0	0	0	0	0	0	0	0	0	0	0	0	0	0	0	0	0
76	103	15	83	44	30	153	54	80	9	57	0	1	2	6	2	7	0	4	0	2	1	5

C1.026	C1.027	C1.029	C1.030	C1.031	C1.032	C1.033	C1.034	C1.036	C1.037	C1.038	C2.002	C2.003	C2.006	C2.009	C2.010	C2.012	C2.013	C2.014	C2.016	C2.017	C2.018	C2.019
Oral Ca	Oral Ca	Oral Ca	Oral Ca	Oral Ca	Oral Ca	Oral Ca	Oral Ca	Oral Ca	Oral Ca	Oral Ca	Oral Ca	Oral Ca	Oral Ca	Oral Ca	Oral Ca	Oral Ca	Oral Ca	Oral Ca	Oral Ca	Oral Ca	Oral Ca	Oral Ca
Sa	Sa	Sa	Sa	Sa	Sa	Sa	Sa	Sa	Sa	Sa	TD	TD	TD	TD	TD	TD	TD	TD	TD	TD	TD	TD
5	95	160	7	1	0	0	2	2	2	2	3	1	0	13	11	8	1	1	0	2	0	7
0	0	0	0	0	0	0	0	0	0	0	0	0	0	0	0	0	0	0	0	0	0	0
0	0	0	0	0	0	0	0	0	0	0	0	0	0	0	0	0	0	0	0	0	0	0
230	234	79	536	255	264	161	465	1564	13	1268	665	885	1106	918	468	48	40	625	281	1037	397	231
5	33	97	7	17	10	1	2	34	0	50	45	4	1174	10	4783	60	1	0	175	0	83	6
0	0	0	0	0	0	0	0	0	0	0	0	0	0	0	0	0	0	0	0	0	0	0
0	0	0	0	0	0	0	0	0	0	0	0	0	0	0	0	0	0	0	0	0	0	0
1	11	34	0	1	0	0	0	1	0	0	0	0	0	3	1	4	0	0	0	0	0	0
0	0	0	0	0	0	0	0	0	0	0	0	0	0	0	0	0	0	0	0	0	0	0
0	0	0	0	0	0	0	1	0	0	0	0	0	0	0	0	0	0	0	0	0	0	0
0	0	0	0	0	1	0	0	0	2	0	0	0	0	0	0	0	1	0	0	0	0	0
70	289	1	120	187	121	169	143	212	87	177	22	16	6	23	31	0	33	2	11	10	48	3
17	124	0	12	87	52	46	36	31	39	26	0	2	0	0	0	0	3	0	1	1	0	0
0	3	0	4	3	2	0	2	0	0	0	0	0	0	17	0	0	0	0	0	5	0	0
5	9	3	0	1	5	0	0	0	3	0	2	3	2	2	11	4	9	2	1	0	2	0
3	1	0	0	0	0	0	0	0	0	0	15	9	5	7	8	0	10	0	0	0	2	8
1	0	0	0	0	0	1	1	0	0	0	0	0	0	1	0	0	0	0	0	0	1	0
0	0	0	0	0	0	0	0	0	0	0	0	0	0	0	0	0	0	0	0	0	0	0
0	0	0	0	0	0	0	0	0	0	0	0	0	0	0	0	0	0	0	0	0	0	0
6	9	0	0	0	4	0	7	12	0	9	16	16	77	29	91	7	9	0	13	9	4	91
0	0	2	0	0	0	0	0	0	0	0	0	0	0	0	0	0	0	0	0	0	0	0
13	49	7	75	70	74	123	100	23	46	33	1	0	0	0	180	62	1	0	0	8	3	1
0	0	0	0	0	0	0	0	0	0	0	0	0	0	0	0	0	0	0	0	0	0	0
0	0	0	0	0	0	0	0	0	0	0	0	0	0	0	0	0	0	0	0	0	0	0
0	2	0	0	0	0	0	1	0	0	0	0	0	0	0	0	0	0	0	0	0	0	0
0	0	1	0	0	0	0	0	0	0	0	0	0	0	0	0	0	0	0	0	0	0	0
0	0	0	0	0	0	0	0	0	0	0	0	0	0	0	0	0	0	0	0	0	0	0
0	0	0	0	0	0	0	0	0	0	0	0	0	0	0	0	0	0	0	0	0	0	0
0	0	0	0	0	0	0	0	0	0	0	0	0	0	0	0	0	0	0	0	0	0	0
0	0	0	0	0	0	0	0	0	0	0	0	0	0	0	0	0	0	0	0	0	0	0
0	0	0	0	0	0	0	0	0	0	0	0	0	0	0	0	0	0	0	0	0	0	0
0	0	0	0	0	0	0	0	0	0	0	0	0	0	0	0	0	0	0	0	0	0	0
0	0	0	0	0	0	0	0	0	0	0	0	0	0	0	0	0	0	0	0	0	0	0
0	0	0	0	0	0	0	0	0	0	0	0	0	0	0	0	0	0	0	0	0	0	0
148	275	803	471	402	354	1759	908	111	1178	469	612	1270	161	267	46	268	525	2153	280	1079	251	1730
0	0	0	0	0	0	0	0	0	0	0	0	0	0	0	0	0	0	0	0	0	0	0
0	0	0	0	0	0	0	0	0	0	0	0	0	0	0	0	0	0	0	0	0	0	0

C1.026	C1.027	C1.029	C1.030	C1.031	C1.032	C1.033	C1.034	C1.036	C1.037	C1.038	C2.002	C2.003	C2.006	C2.009	C2.010	C2.012	C2.013	C2.014	C2.016	C2.017	C2.018	C2.019
Oral Ca	Oral Ca	Oral Ca	Oral Ca	Oral Ca	Oral Ca	Oral Ca	Oral Ca	Oral Ca	Oral Ca	Oral Ca	Oral Ca	Oral Ca	Oral Ca	Oral Ca	Oral Ca	Oral Ca	Oral Ca	Oral Ca	Oral Ca	Oral Ca	Oral Ca	Oral Ca
Sa	Sa	Sa	Sa	Sa	Sa	Sa	Sa	Sa	Sa	Sa	TD	TD	TD	TD	TD	TD	TD	TD	TD	TD	TD	TD
7	173	42	45	106	69	141	55	90	7	69	1	2	2	3	0	2	0	8	3	5	2	30
0	0	0	0	0	0	0	0	0	0	0	0	0	0	0	0	0	0	0	0	0	0	0
0	0	0	0	0	0	0	0	0	0	0	0	0	0	0	0	0	0	0	0	0	0	0
0	0	0	0	0	0	0	0	0	0	0	0	0	0	0	0	0	0	0	0	0	0	0
0	0	0	0	0	0	0	0	0	0	0	0	0	0	0	0	0	0	0	0	0	0	0
0	0	0	0	0	0	0	0	0	0	0	0	0	0	0	0	0	0	0	0	0	0	0
0	0	1	0	0	0	0	0	0	0	0	0	0	0	0	0	0	0	0	0	0	0	0
0	0	0	0	0	0	0	0	0	0	0	0	0	0	0	0	0	0	0	0	0	0	0
0	0	1	0	0	0	0	0	0	0	0	0	0	0	0	1	0	0	0	0	0	0	0
0	0	0	0	0	0	0	0	0	0	0	0	0	0	0	0	0	0	0	0	0	0	0
0	0	0	0	0	0	0	0	0	0	0	0	0	0	0	0	0	0	0	0	0	0	0
0	0	1	0	0	0	0	0	0	0	2	0	0	0	0	0	2	0	0	0	0	0	0
218	82	2384	739	2993	1022	2958	1993	447	2364	1115	165	97	225	86	28	48	392	87	106	2118	128	215
0	0	1	0	0	0	0	0	0	0	0	0	0	0	0	0	0	0	0	0	0	0	0
0	0	0	0	0	0	0	0	0	0	0	0	0	0	0	0	0	0	0	0	0	0	0
0	1	1	0	0	0	0	0	0	0	0	0	0	0	1	1	0	0	0	0	0	0	0
0	0	0	3	0	0	0	0	0	0	0	0	0	0	0	0	0	0	0	0	0	0	0
0	0	0	0	0	0	0	0	0	0	0	0	0	0	0	0	0	0	0	0	0	0	0
0	0	0	0	0	0	0	0	0	0	0	0	0	0	0	0	0	0	0	0	0	0	0
0	26	4	56	44	36	63	46	175	22	52	0	0	1	3	0	0	2	1	0	2	0	4
0	0	1	0	0	0	0	0	0	0	0	0	0	0	0	0	1	0	0	0	0	0	0
0	0	0	0	0	0	0	0	0	0	0	0	0	0	0	0	0	0	0	0	0	0	0
0	0	0	0	0	0	0	0	0	0	0	0	0	0	0	0	0	0	0	0	0	0	0
0	0	0	0	0	0	0	0	0	0	0	0	0	0	0	0	0	0	0	0	0	0	0
0	0	0	0	0	0	0	0	0	0	0	0	0	0	0	0	0	0	0	0	0	0	0
0	0	0	0	0	0	0	0	0	0	0	0	0	0	0	0	0	0	0	0	0	0	0
9	203	47	0	4	4	0	13	16	12	3	14	0	0	6	202	69	40	2	1	45	1	8
7	42	56	30	51	38	138	144	24	230	81	43	40	10	12	41	26	25	234	56	56	10	272
0	0	0	0	0	0	0	0	0	0	0	0	0	0	0	0	0	0	0	0	0	0	0
0	0	0	0	0	0	0	0	0	0	0	0	0	0	0	0	0	0	0	0	0	0	0
0	0	0	0	0	0	0	0	0	0	0	0	0	0	0	0	0	0	0	0	0	0	0
0	0	0	0	0	0	0	0	0	0	0	0	0	0	0	0	0	0	0	0	0	0	0
0	0	1	0	0	0	0	0	0	0	0	0	0	0	0	0	0	0	0	0	0	0	0
0	0	0	0	0	0	0	0	0	0	0	0	0	0	0	0	0	0	0	0	0	0	0
0	0	39	0	0	0	0	0	0	0	0	0	0	0	1	0	0	0	0	0	0	0	0
0	0	0	0	0	0	0	0	0	0	0	0	0	0	0	0	0	0	0	0	0	0	0
0	0	0	0	0	0	0	0	0	0	0	0	0	0	0	0	0	0	0	0	0	0	0

C1.026	C1.027	C1.029	C1.030	C1.031	C1.032	C1.033	C1.034	C1.036	C1.037	C1.038	C2.002	C2.003	C2.006	C2.009	C2.010	C2.012	C2.013	C2.014	C2.016	C2.017	C2.018	C2.019
Oral Ca	Oral Ca	Oral Ca	Oral Ca	Oral Ca	Oral Ca	Oral Ca	Oral Ca	Oral Ca	Oral Ca	Oral Ca	Oral Ca	Oral Ca	Oral Ca	Oral Ca	Oral Ca	Oral Ca	Oral Ca	Oral Ca	Oral Ca	Oral Ca	Oral Ca	Oral Ca
Sa	Sa	Sa	Sa	Sa	Sa	Sa	Sa	Sa	Sa	Sa	TD	TD	TD	TD	TD	TD	TD	TD	TD	TD	TD	TD
0	0	0	0	0	0	0	4	0	0	0	0	0	0	0	0	0	0	0	0	0	0	0
1188	2764	2605	4263	2057	1388	2255	2384	1210	1313	4014	1183	1117	1806	486	1951	966	843	918	725	1259	633	1547
6	22	1	0	16	9	0	15	13	34	4	2	6	0	11	1	0	4	0	1	0	4	1
8	85	0	0	0	0	0	23	0	0	0	1	5	0	0	0	0	3	0	0	0	0	0
0	0	0	0	0	0	0	0	0	0	0	0	0	0	2	0	0	0	0	0	0	0	0
4404	5244	2052	3323	3967	4040	1677	3057	3229	734	2287	213	1351	1864	327	697	97	118	9	272	54	635	119
392	1113	160	431	442	364	833	784	505	617	1526	2918	2881	2070	490	560	804	2111	7292	2099	2622	806	4862
0	0	0	0	0	1	0	0	0	0	0	0	0	0	1	0	0	0	0	0	0	0	0
0	0	0	0	0	0	0	0	0	0	0	0	0	0	5	0	1	0	0	0	1	0	0
2	1	2	0	0	0	0	0	0	0	0	1	0	0	6	0	1	0	1	0	0	1	0
0	0	0	0	0	0	0	0	0	0	0	0	0	0	0	0	0	0	0	0	0	0	0
0	0	1	0	0	0	0	0	0	0	0	0	0	0	0	0	0	0	0	0	0	0	0
10	31	14	2	3	1	2	3	5	5	6	23	19	46	8	23	3	8	2	7	4	30	4
0	0	0	0	0	0	0	0	0	0	0	0	0	0	0	0	0	0	0	0	0	0	0
144	239	22	40	53	59	22	24	82	22	86	84	75	138	76	81	79	44	38	57	34	119	82
1	0	4	0	0	0	0	0	0	0	0	0	0	0	0	2	0	0	0	0	0	0	0
0	0	0	0	0	0	0	0	0	0	0	0	0	0	0	0	0	0	0	0	0	0	0
0	0	0	0	0	0	0	0	0	0	0	0	0	0	0	0	0	1	0	0	0	0	0
0	1	0	0	0	1	0	0	1	1	0	0	0	0	0	0	0	0	0	0	0	0	0
0	0	0	0	0	0	0	0	0	0	0	0	0	0	0	0	0	0	0	0	0	0	0
0	0	0	0	0	0	0	0	0	0	0	0	0	0	0	0	0	0	0	0	0	0	0
0	0	0	0	0	0	0	0	0	0	0	0	0	0	0	0	0	0	0	0	0	0	0
0	0	0	0	0	0	0	0	0	0	0	0	0	0	0	0	0	0	0	0	0	0	0
0	1	0	0	0	0	0	0	0	0	0	0	0	0	1	0	0	0	0	0	0	0	0
0	0	2	0	0	0	0	0	0	0	0	0	0	0	0	0	0	0	0	0	0	0	0
0	0	0	0	0	0	0	0	0	0	0	0	0	0	0	0	0	0	0	0	0	0	0
0	0	0	0	0	0	0	0	0	0	0	0	0	0	0	0	0	0	0	0	0	0	0
0	13	6	0	0	0	0	0	0	0	0	0	0	0	0	7	7	0	0	0	0	0	0
0	0	0	0	0	0	0	0	0	0	0	0	0	0	0	0	0	0	0	0	0	0	0
0	0	2	0	0	0	0	0	0	0	0	0	0	0	1	0	0	0	0	0	0	0	0
0	0	1	0	0	0	0	0	0	0	0	0	0	0	0	2	0	0	0	0	0	0	0
0	0	0	0	0	0	0	0	0	0	0	0	0	0	0	0	0	0	0	0	0	0	0
0	0	4	0	0	0	0	0	0	0	0	0	0	0	0	0	0	0	0	0	0	0	0
0	0	0	0	0	0	0	0	0	0	0	0	0	0	0	0	0	0	0	0	0	0	0
7	14	26	7	4	2	9	15	9	10	5	117	65	57	20	28	17	123	7	58	67	121	23
6	1	26	17	18	12	2	19	57	9	9	3	2	4	14	68	82	0	89	2	1	23	4
34	15	48	56	78	69	87	89	55	27	163	304	285	227	749	179	155	542	319	567	423	783	573

C1.026	C1.027	C1.029	C1.030	C1.031	C1.032	C1.033	C1.034	C1.036	C1.037	C1.038	C2.002	C2.003	C2.006	C2.009	C2.010	C2.012	C2.013	C2.014	C2.016	C2.017	C2.018	C2.019
Oral Ca	Oral Ca	Oral Ca	Oral Ca	Oral Ca	Oral Ca	Oral Ca	Oral Ca	Oral Ca	Oral Ca	Oral Ca	Oral Ca	Oral Ca	Oral Ca	Oral Ca	Oral Ca	Oral Ca	Oral Ca	Oral Ca	Oral Ca	Oral Ca	Oral Ca	Oral Ca
Sa	Sa	Sa	Sa	Sa	Sa	Sa	Sa	Sa	Sa	Sa	TD	TD	TD	TD	TD	TD	TD	TD	TD	TD	TD	TD
0	0	8	0	0	0	0	0	0	0	0	0	0	0	0	6	1	0	0	0	0	0	0
161	86	406	0	0	0	0	0	4	0	0	0	2	0	13	403	58	124	4	0	12	1	121
0	0	1	0	0	0	0	0	3	0	0	0	0	0	0	0	0	0	0	0	0	0	0
248	120	328	375	433	407	270	579	991	593	761	1451	707	777	888	311	618	1881	1815	857	4924	1321	1445
0	0	0	0	0	0	0	0	0	0	0	0	0	0	0	0	0	0	0	0	0	0	0
0	0	0	0	0	0	0	1	0	0	0	0	0	0	0	0	0	0	0	0	0	0	0
21	26	14	17	42	25	23	58	13	48	49	211	177	72	77	177	58	343	132	172	266	174	161
28	22	2	0	4	3	31	1	4	4	1	1	48	27	6	48	10	7	0	2	1	159	1
0	0	0	0	0	0	0	0	0	0	0	0	0	0	2	0	0	0	0	0	0	0	0
2	41	8	0	0	0	0	0	0	0	0	0	0	0	2	60	12	0	0	0	0	0	0
0	0	0	0	0	0	0	0	0	0	0	0	0	0	0	0	0	0	0	0	0	0	0
0	0	0	0	1	0	0	0	0	1	0	0	0	0	2	0	0	18	1	0	3	3	0
97	232	170	95	122	119	228	365	160	138	562	533	261	273	508	315	320	392	632	465	718	661	860
2	6	0	5	6	5	0	6	4	1	12	4	15	2	8	4	0	0	0	5	3	14	0
25	21	3	10	12	7	11	6	14	4	18	49	54	114	30	121	11	37	139	33	26	320	28
16	7	3	2	2	2	1	3	1	0	1	0	1	4	62	0	0	2	0	0	0	0	0
0	0	0	0	0	5	0	1	0	0	0	0	0	0	0	0	0	1	0	0	0	0	0
137	181	84	243	303	178	719	263	100	401	451	311	910	527	135	128	155	1171	2275	727	1468	175	1969
0	0	0	0	0	0	0	0	0	0	0	0	0	0	0	0	0	0	0	0	0	0	0
14	9	11	23	16	14	41	49	18	43	98	123	114	98	87	24	73	166	851	86	240	132	261
107	113	37	13	18	13	54	37	39	4	57	450	628	370	60	1241	265	122	193	522	115	787	303
19	110	12	15	6	8	25	7	8	7	19	205	476	381	62	73	131	84	83	137	258	102	390
0	0	0	0	0	0	0	0	0	0	0	0	0	0	0	0	0	0	0	0	0	0	0
0	0	0	0	0	0	0	0	0	0	0	0	0	0	0	0	0	0	0	0	0	0	0
1	20	51	1	1	0	1	0	4	0	0	0	0	0	2	3	3	0	1	39	109	75	29
0	0	0	0	0	0	0	0	0	0	0	0	0	0	0	0	0	0	0	0	0	0	0
0	0	0	0	0	0	0	0	0	0	0	0	0	0	0	0	0	0	0	0	0	0	0
0	0	0	0	0	0	0	0	0	0	0	0	0	0	0	0	0	0	0	0	0	0	0
0	0	14	0	1	0	0	0	0	0	0	0	0	0	0	1	1	0	0	0	0	1	0
0	3	27	0	0	0	0	1	0	0	0	0	0	0	1	3	1	1	0	0	0	0	0
0	0	3	0	0	0	0	0	0	0	0	0	0	0	0	0	0	0	0	0	0	0	0
0	2	3	0	0	0	0	0	2	0	0	1	0	0	0	0	1	0	0	0	0	0	0
0	0	0	0	0	0	0	0	0	0	0	0	0	0	0	0	0	0	0	0	0	0	0
0	0	5	0	0	0	0	0	0	0	0	0	0	0	0	0	0	0	0	0	0	0	1
0	0	3	0	0	0	0	0	0	0	0	0	0	0	0	0	0	0	0	0	0	0	0
0	0	0	0	0	0	0	0	0	0	0	0	0	0	0	0	0	0	0	0	0	0	0
0	0	2	0	0	0	0	0	0	0	0	0	0	0	0	0	0	0	0	0	0	0	0

C1.026	C1.027	C1.029	C1.030	C1.031	C1.032	C1.033	C1.034	C1.036	C1.037	C1.038	C2.002	C2.003	C2.006	C2.009	C2.010	C2.012	C2.013	C2.014	C2.016	C2.017	C2.018	C2.019
Oral Ca	Oral Ca	Oral Ca	Oral Ca	Oral Ca	Oral Ca	Oral Ca	Oral Ca	Oral Ca	Oral Ca	Oral Ca	Oral Ca	Oral Ca	Oral Ca	Oral Ca	Oral Ca	Oral Ca	Oral Ca	Oral Ca	Oral Ca	Oral Ca	Oral Ca	Oral Ca
Sa	Sa	Sa	Sa	Sa	Sa	Sa	Sa	Sa	Sa	Sa	TD	TD	TD	TD	TD	TD	TD	TD	TD	TD	TD	TD
0	0	1	0	0	0	0	0	0	0	0	0	0	0	0	0	1	0	0	0	0	0	0
0	0	3	0	0	0	0	0	0	0	0	0	0	0	0	0	0	0	0	0	0	0	0
0	0	9	0	0	0	0	0	1	0	0	1	0	0	0	0	0	0	1	0	0	0	0
0	0	0	0	0	0	0	0	0	0	0	0	0	0	0	0	0	0	0	0	0	0	0
0	0	0	0	0	0	0	0	0	0	0	0	0	0	0	0	0	0	0	0	0	0	0
0	0	0	0	0	0	0	0	0	0	0	0	0	0	0	0	0	0	0	0	0	0	0
0	0	2	0	0	0	0	0	0	0	0	0	0	0	0	0	0	0	0	0	0	0	0
42	73	172	0	0	0	0	0	2	0	0	0	1	1	0	0	7	1	0	0	1	0	1
3	1	3	0	1	0	0	2	0	0	0	0	0	0	7	0	1	0	0	0	0	0	0
216	301	637	262	95	84	243	248	581	89	125	7	10	9	1	33	21	0	65	9	71	7	5
108	1370	40	76	158	158	301	85	405	151	56	1	1	0	3	4	13	0	0	0	5	1	6
0	1	2	0	0	0	0	0	3	0	0	0	1	0	2	0	0	1	0	0	1	0	0
0	0	0	0	0	0	0	0	0	0	0	0	0	0	0	0	0	0	0	0	0	0	0
4304	2344	9320	6814	5880	5370	5047	4377	7989	3116	5534	9552	4379	10484	4248	3143	2419	177	2064	8134	3674	12022	1937
0	35	0	1	6	3	3	0	11	0	0	0	0	0	0	0	0	0	0	0	0	0	0
0	0	1	0	0	0	0	0	0	0	0	0	0	0	0	0	0	0	0	0	0	0	0
0	0	2	0	0	0	0	0	0	0	0	0	0	0	0	0	0	0	0	0	0	0	0
1	1	0	0	11	11	11	21	14	4	3	3	4	0	0	0	0	1	0	0	0	0	0
0	0	0	0	14	9	0	0	0	0	0	0	0	0	0	0	0	0	0	0	1	1	0
19	219	0	1	17	14	0	21	0	0	0	0	0	0	0	1	0	1	0	0	0	1	0
0	0	0	0	0	0	0	0	0	0	0	0	0	0	0	0	0	0	0	0	0	0	0
0	0	0	0	0	0	0	0	0	0	0	0	0	0	0	0	0	0	0	0	0	0	0
0	0	0	0	0	0	0	0	0	0	0	0	0	0	0	0	0	0	0	0	0	0	0
0	0	0	0	0	0	0	0	0	0	0	0	0	0	0	0	0	0	0	0	0	0	0
32	44	10	12	22	13	24	26	15	11	38	274	142	85	135	50	198	186	203	70	323	201	531
1	16	0	1	0	0	1	3	5	0	0	0	2	0	2	0	0	0	0	0	1	0	0
0	0	1	0	0	0	0	0	0	0	0	0	0	0	0	0	0	0	0	0	0	0	0
4	1	0	0	0	0	0	0	0	0	0	0	0	0	1	0	0	0	0	0	0	0	0
0	0	0	0	0	0	0	0	0	0	0	0	0	0	0	0	0	0	0	0	0	0	0
0	0	0	0	0	1	0	0	0	0	0	0	0	0	0	0	0	0	0	0	0	0	0
0	0	0	0	0	0	0	0	0	0	0	0	0	0	0	0	0	0	0	0	0	0	0
76	103	15	78	44	30	153	54	80	9	57	0	1	2	6	2	7	0	4	0	2	1	5
5	95	160	7	1	0	0	2	2	2	2	3	1	0	13	11	8	1	1	0	2	0	7
0	0	0	0	0	0	0	0	0	0	0	0	0	0	0	0	0	0	0	0	0	0	0
0	0	0	0	0	0	0	0	0	0	0	0	0	0	0	0	0	0	0	0	0	0	0
0	0	0	0	0	0	0	0	0	0	0	0	0	0	0	0	0	0	0	0	0	0	0
0	0	0	0	0	0	0	0	0	0	0	0	0	0	0	0	0	0	0	0	0	0	0

C1.026	C1.027	C1.029	C1.030	C1.031	C1.032	C1.033	C1.034	C1.036	C1.037	C1.038	C2.002	C2.003	C2.006	C2.009	C2.010	C2.012	C2.013	C2.014	C2.016	C2.017	C2.018	C2.019
Oral Ca	Oral Ca	Oral Ca	Oral Ca	Oral Ca	Oral Ca	Oral Ca	Oral Ca	Oral Ca	Oral Ca	Oral Ca	Oral Ca	Oral Ca	Oral Ca	Oral Ca	Oral Ca	Oral Ca	Oral Ca	Oral Ca	Oral Ca	Oral Ca	Oral Ca	Oral Ca
Sa	Sa	Sa	Sa	Sa	Sa	Sa	Sa	Sa	Sa	Sa	TD	TD	TD	TD	TD	TD	TD	TD	TD	TD	TD	TD
230	234	79	536	255	264	161	465	1564	13	1268	665	885	1106	918	468	48	40	625	281	1037	397	231
0	12	19	6	17	10	1	2	33	0	50	45	4	1174	7	4778	54	1	0	175	0	83	5
4	21	78	1	0	0	0	0	1	0	0	0	0	0	3	5	6	0	0	0	0	0	1
0	0	0	0	0	0	0	0	0	0	0	0	0	0	0	0	0	0	0	0	0	0	0
0	0	0	0	0	0	0	0	0	0	0	0	0	0	0	0	0	0	0	0	0	0	0
0	0	0	0	0	0	0	0	0	0	0	0	0	0	0	0	0	0	0	0	0	0	0
0	2	4	0	0	0	0	0	0	0	0	0	0	0	0	0	1	0	0	0	0	0	0
1	9	30	0	1	0	0	0	1	0	0	0	0	0	3	1	3	0	0	0	0	0	0
0	0	0	0	0	1	0	0	0	2	0	0	0	0	0	0	0	1	0	0	0	0	0
70	289	1	120	187	121	169	143	212	87	177	22	16	6	23	31	0	33	2	11	10	48	3
16	124	0	12	87	52	46	34	31	37	26	0	2	0	0	0	0	3	0	1	1	0	0
1	0	0	0	0	0	0	2	0	2	0	0	0	0	0	0	0	0	0	0	0	0	0
0	3	0	4	3	2	0	2	0	0	0	0	0	0	17	0	0	0	0	0	5	0	0
5	9	3	0	1	5	0	0	0	3	0	2	3	2	2	11	4	9	2	1	0	2	0
0	0	0	0	0	0	0	0	0	0	0	0	0	0	0	0	0	0	0	0	0	0	0
4	0	0	0	0	3	0	6	2	0	3	3	6	6	0	7	7	6	0	11	9	2	59
13	49	7	75	70	74	123	100	23	46	33	1	0	0	0	180	62	1	0	0	8	3	1
0	0	0	0	0	0	0	0	0	0	0	0	0	0	0	0	0	0	0	0	0	0	0
0	0	0	0	0	0	0	0	0	0	0	0	0	0	0	0	0	0	0	0	0	0	0
0	0	1	0	0	0	0	0	0	0	0	0	0	0	0	0	0	0	0	0	0	0	0
0	0	0	0	0	0	0	0	0	0	0	0	0	0	0	0	0	0	0	0	0	0	0
0	0	0	0	0	0	0	0	0	0	0	0	0	0	0	0	0	0	0	0	0	0	0
0	0	0	0	0	0	0	0	0	0	0	0	0	0	0	0	0	0	0	0	0	0	0
0	0	0	0	0	0	0	0	0	0	0	0	0	0	0	0	0	0	0	0	0	0	0
0	0	0	0	0	0	0	0	0	0	0	0	0	0	0	0	0	0	0	0	0	0	0
0	0	0	0	0	0	0	0	0	0	0	0	0	0	0	0	0	0	0	0	0	0	0
0	0	0	0	0	0	0	0	0	0	0	0	0	0	0	0	0	0	0	0	0	0	0
0	0	0	0	0	0	0	0	0	0	0	0	0	0	0	0	0	0	0	0	0	0	0
145	273	803	468	398	347	1750	895	110	1164	469	610	1270	160	267	45	268	524	2148	278	1070	251	1729
0	0	0	0	0	0	0	10	0	0	0	1	0	0	0	1	0	1	0	1	0	0	0
0	0	0	0	0	0	0	0	0	0	0	0	0	0	0	0	0	0	0	0	0	0	0
0	0	0	0	0	0	0	0	0	0	0	0	0	0	0	0	0	0	0	0	0	0	0
3	2	0	3	4	7	9	3	1	14	0	1	0	1	0	0	0	0	5	1	9	0	1
0	0	0	0	0	0	0	0	0	0	0	0	0	0	0	0	0	0	0	0	0	0	0
0	0	0	0	0	0	0	0	0	0	0	0	0	0	0	0	0	0	0	0	0	0	0
7	173	42	45	106	69	141	55	90	7	69	1	2	2	3	0	2	0	8	3	5	2	30
0	0	0	0	0	0	0	0	0	0	0	0	0	0	0	0	0	0	0	0	0	0	0

C1.026	C1.027	C1.029	C1.030	C1.031	C1.032	C1.033	C1.034	C1.036	C1.037	C1.038	C2.002	C2.003	C2.006	C2.009	C2.010	C2.012	C2.013	C2.014	C2.016	C2.017	C2.018	C2.019
Oral Ca	Oral Ca	Oral Ca	Oral Ca	Oral Ca	Oral Ca	Oral Ca	Oral Ca	Oral Ca	Oral Ca	Oral Ca	Oral Ca	Oral Ca	Oral Ca	Oral Ca	Oral Ca	Oral Ca	Oral Ca	Oral Ca	Oral Ca	Oral Ca	Oral Ca	Oral Ca
Sa	Sa	Sa	Sa	Sa	Sa	Sa	Sa	Sa	Sa	Sa	TD	TD	TD	TD	TD	TD	TD	TD	TD	TD	TD	TD
0	0	0	0	0	0	0	0	0	0	0	0	0	0	0	0	0	0	0	0	0	0	0
0	0	0	0	0	0	0	0	0	0	0	0	0	0	0	0	0	0	0	0	0	0	0
0	0	0	0	0	0	0	0	0	0	0	0	0	0	0	0	0	0	0	0	0	0	0
0	0	0	0	0	0	0	0	0	0	0	0	0	0	0	0	0	0	0	0	0	0	0
0	0	0	0	0	0	0	0	0	0	0	0	0	0	0	0	0	0	0	0	0	0	0
0	0	0	0	0	0	0	0	0	0	0	0	0	0	0	0	0	0	0	0	0	0	0
0	0	0	0	0	0	0	0	0	0	0	0	0	0	0	0	0	0	0	0	0	0	0
0	0	1	0	0	0	0	0	0	0	0	0	0	0	0	0	0	0	0	0	0	0	0
0	0	0	0	0	0	0	0	0	0	0	0	0	0	0	0	0	0	0	0	0	0	0
0	0	0	0	0	0	0	0	0	0	0	0	0	0	0	0	0	0	0	0	0	0	0
0	0	0	0	0	0	0	0	0	0	0	0	0	0	0	0	0	0	0	0	0	0	0
0	0	0	0	0	0	0	0	0	0	0	0	0	0	0	0	0	0	0	0	0	0	0
0	0	1	0	0	0	0	0	0	0	0	0	0	0	0	1	0	0	0	0	0	0	0
0	0	0	0	0	0	0	0	0	0	0	0	0	0	0	0	0	0	0	0	0	0	0
0	0	0	0	0	0	0	0	0	0	0	0	0	0	0	0	0	0	0	0	0	0	0
0	0	0	0	0	0	0	0	0	0	0	0	0	0	0	0	0	0	0	0	0	0	0
0	0	0	0	0	0	0	0	0	0	0	0	0	0	0	0	0	0	0	0	0	0	0
0	0	0	0	0	0	0	0	0	0	0	0	0	0	0	0	0	0	0	0	0	0	0
0	0	0	0	0	0	0	0	0	0	0	0	0	0	0	0	0	0	0	0	0	0	0
0	0	0	0	0	0	0	0	0	0	0	0	0	0	0	0	0	0	0	0	0	0	0
0	0	0	0	0	0	0	0	0	0	0	0	0	0	0	0	1	0	0	0	0	0	0
0	0	0	0	0	0	0	0	0	0	0	0	0	0	0	0	0	0	0	0	0	0	0
0	0	0	0	0	0	0	0	0	0	0	0	0	0	0	0	0	0	0	0	0	0	0
0	0	0	0	0	0	0	0	0	0	2	0	0	0	0	0	0	0	0	0	0	0	0
0	0	1	0	0	0	0	0	0	0	0	0	0	0	0	0	1	0	0	0	0	0	0
0	0	1	0	0	0	0	0	0	0	0	0	0	0	0	0	0	0	0	0	0	0	0
0	0	0	0	0	0	0	0	0	0	0	0	0	0	0	0	0	0	0	0	0	0	0
0	0	0	0	0	0	0	0	0	0	0	0	0	0	0	0	0	0	0	0	0	0	0
0	0	0	0	0	0	0	0	0	0	0	0	0	0	0	0	0	0	0	0	0	0	0
0	0	0	0	0	0	0	0	0	0	0	0	0	0	0	0	0	0	0	0	0	0	0
0	1	0	0	1	0	0	0	1	0	0	0	0	0	0	0	0	0	0	0	0	0	0
218	81	2383	739	2992	1022	2958	1993	446	2364	1115	165	97	224	86	28	48	392	87	106	2118	128	215
0	0	0	0	0	0	0	0	0	0	0	0	0	0	0	0	0	0	0	0	0	0	0
0	0	0	0	0	0	0	0	0	0	0	0	0	1	0	0	0	0	0	0	0	0	0
0	0	1	0	0	0	0	0	0	0	0	0	0	0	0	0	0	0	0	0	0	0	0
0	0	0	0	0	0	0	0	0	0	0	0	0	0	0	0	0	0	0	0	0	0	0
0	0	0	0	0	0	0	0	0	0	0	0	0	0	0	0	0	0	0	0	0	0	0

C1.026	C1.027	C1.029	C1.030	C1.031	C1.032	C1.033	C1.034	C1.036	C1.037	C1.038	C2.002	C2.003	C2.006	C2.009	C2.010	C2.012	C2.013	C2.014	C2.016	C2.017	C2.018	C2.019
Oral Ca	Oral Ca	Oral Ca	Oral Ca	Oral Ca	Oral Ca	Oral Ca	Oral Ca	Oral Ca	Oral Ca	Oral Ca	Oral Ca	Oral Ca	Oral Ca	Oral Ca	Oral Ca	Oral Ca	Oral Ca	Oral Ca	Oral Ca	Oral Ca	Oral Ca	Oral Ca
Sa	Sa	Sa	Sa	Sa	Sa	Sa	Sa	Sa	Sa	Sa	TD	TD	TD	TD	TD	TD	TD	TD	TD	TD	TD	TD
0	1	1	0	0	0	0	0	0	0	0	0	0	0	1	1	0	0	0	0	0	0	0
0	0	0	0	0	0	0	0	0	0	0	0	0	0	0	0	0	0	0	0	0	0	0
0	0	0	0	0	0	0	0	0	0	0	0	0	0	0	0	0	0	0	0	0	0	0
0	0	0	0	0	0	0	0	0	0	0	0	0	0	0	0	0	0	0	0	0	0	0
0	0	0	0	0	0	0	0	0	0	0	0	0	0	0	0	0	0	0	0	0	0	0
0	0	0	0	0	0	0	0	0	0	0	0	0	0	0	0	0	0	0	0	0	0	0
0	0	0	3	0	0	0	0	0	0	0	0	0	0	0	0	0	0	0	0	0	0	0
0	0	0	0	0	0	0	0	0	0	0	0	0	0	0	0	0	0	0	0	0	0	0
0	0	0	0	0	0	0	0	0	0	0	0	0	0	0	0	0	0	0	0	0	0	0
0	0	0	0	0	0	0	0	0	0	0	0	0	0	0	0	0	0	0	0	0	0	0
0	0	0	0	0	0	0	0	0	0	0	0	0	0	0	0	0	0	0	0	0	0	0
0	0	0	0	0	0	0	0	0	0	0	0	0	0	0	0	0	0	0	0	0	0	0
0	0	0	0	0	0	0	0	0	0	0	0	0	0	0	0	0	0	0	0	0	0	0
0	0	0	0	0	0	0	0	0	0	0	0	0	0	0	0	0	0	0	0	0	0	0
0	0	3	0	1	0	0	0	0	0	0	0	0	0	0	0	0	2	0	0	0	0	0
0	0	0	0	0	0	0	0	0	0	0	0	0	0	0	0	0	0	0	0	0	0	0
0	26	1	56	43	36	63	46	175	22	52	0	0	1	3	0	0	0	1	0	2	0	4
0	0	1	0	0	0	0	0	0	0	0	0	0	0	0	0	1	0	0	0	0	0	0
0	0	0	0	0	0	0	0	0	0	0	0	0	0	0	0	0	0	0	0	0	0	0
0	0	0	0	0	0	0	0	0	0	0	0	0	0	0	0	0	0	0	0	0	0	0
0	0	0	0	0	0	0	0	0	0	0	0	0	0	0	0	0	0	0	0	0	0	0
0	0	0	0	0	0	0	0	0	0	0	0	0	0	0	0	0	0	0	0	0	0	0
0	0	0	0	0	0	0	0	0	0	0	0	0	0	0	0	0	0	0	0	0	0	0
0	0	0	0	0	0	0	0	0	0	0	0	0	0	0	0	0	0	0	0	0	0	0
0	0	0	0	0	0	0	0	0	0	0	0	0	0	0	0	0	0	0	0	0	0	0
0	0	0	0	0	0	0	0	0	0	0	0	0	0	0	0	0	0	0	0	0	0	0
0	0	0	0	0	0	0	0	0	0	0	0	0	0	0	0	0	0	0	0	0	0	0
0	2	4	0	1	0	0	4	9	9	0	0	0	0	5	5	1	36	2	1	33	1	1
0	0	0	0	0	0	0	0	0	0	0	0	0	0	0	0	0	0	0	0	0	0	0
0	0	23	0	2	3	0	1	7	0	3	0	0	0	1	0	1	2	0	0	5	0	0
0	1	1	0	0	0	0	0	0	0	0	0	0	0	0	1	0	0	0	0	0	0	0
9	200	19	0	1	1	0	8	0	3	0	14	0	0	0	196	67	2	0	0	7	0	7
0	0	0	0	0	0	0	1	0	0	0	0	0	0	0	0	0	0	0	0	0	0	0
3	4	39	29	36	33	132	118	6	227	81	43	39	10	12	0	13	20	233	55	54	9	271
0	0	0	0	0	0	0	0	0	0	0	0	0	0	0	0	0	0	0	0	0	0	0
0	0	0	0	0	0	0	0	0	0	0	0	0	0	0	0	0	0	0	0	0	0	0
0	1	0	0	5	0	3	9	0	0	0	0	0	0	0	0	0	3	0	0	0	0	0

C1.026 Oral Ca Sa	C1.027 Oral Ca Sa	C1.029 Oral Ca Sa	C1.030 Oral Ca Sa	C1.031 Oral Ca Sa	C1.032 Oral Ca Sa	C1.033 Oral Ca Sa	C1.034 Oral Ca Sa	C1.036 Oral Ca Sa	C1.037 Oral Ca Sa	C1.038 Oral Ca Sa	C2.002 Oral Ca TD	C2.003 Oral Ca TD	C2.006 Oral Ca TD	C2.009 Oral Ca TD	C2.010 Oral Ca TD	C2.012 Oral Ca TD	C2.013 Oral Ca TD	C2.014 Oral Ca TD	C2.016 Oral Ca TD	C2.017 Oral Ca TD	C2.018 Oral Ca TD	C2.019 Oral Ca TD
1	0	5	0	2	1	0	2	0	0	0	0	0	0	0	0	0	1	0	1	0	0	1
3	37	12	1	8	4	3	14	18	3	0	0	1	0	0	41	13	1	1	0	2	1	0
0	0	0	0	0	0	0	0	0	0	0	0	0	0	0	0	0	0	0	0	0	0	0
0	0	0	0	0	0	0	0	0	0	0	0	0	0	0	0	0	0	0	0	0	0	0
0	0	0	0	0	0	0	0	0	0	0	0	0	0	0	0	0	0	0	0	0	0	0
0	0	0	0	0	0	0	0	0	0	0	0	0	0	0	0	0	0	0	0	0	0	0
0	0	1	0	0	0	0	0	0	0	0	0	0	0	0	0	0	0	0	0	0	0	0
0	0	0	0	0	0	0	0	0	0	0	0	0	0	0	0	0	0	0	0	0	0	0
0	0	0	0	0	0	0	0	0	0	0	0	0	0	0	0	0	0	0	0	0	0	0
0	0	0	0	0	0	0	0	0	0	0	0	0	0	0	0	0	0	0	0	0	0	0
0	0	0	0	0	0	0	0	0	0	0	0	0	0	0	0	0	0	0	0	0	0	0
0	0	39	0	0	0	0	0	0	0	0	0	0	0	1	0	0	0	0	0	0	0	0
0	0	0	0	0	0	0	0	0	0	0	0	0	0	0	0	0	0	0	0	0	0	0
0	0	0	0	0	0	0	0	0	0	0	0	0	0	0	0	0	0	0	0	0	0	0
0	0	0	0	0	0	0	0	0	0	0	0	0	0	0	0	0	0	0	0	0	0	0
0	0	0	0	0	0	0	0	0	0	0	0	0	0	0	0	0	0	0	0	0	0	0
0	0	0	0	0	0	0	0	0	0	0	0	0	0	0	0	0	0	0	0	0	0	0
0	0	0	0	0	0	0	4	0	0	0	0	0	0	0	0	0	0	0	0	0	0	0
1188	2764	2605	4263	2057	1388	2255	2384	1210	1311	4014	1183	1117	1806	486	1951	966	843	918	725	1259	633	1547
0	0	0	0	0	0	0	0	0	0	0	0	0	0	0	0	0	0	0	0	0	0	0
0	0	0	0	0	0	0	0	0	0	0	0	0	0	0	0	0	0	0	0	0	0	0
0	0	0	0	0	0	0	0	0	2	0	0	0	0	0	0	0	0	0	0	0	0	0
6	22	1	0	16	9	0	15	13	34	4	2	6	0	11	1	0	4	0	1	0	4	1
8	85	0	0	0	0	0	23	0	0	0	1	5	0	0	0	0	3	0	0	0	0	0
0	0	0	0	0	0	0	0	0	0	0	0	0	0	2	0	0	0	0	0	0	0	0
0	0	0	0	0	0	0	0	0	0	0	0	0	0	0	0	0	0	0	0	0	0	0
15	80	0	1	1	5	2	3	6	0	6	1	6	1	1	1	1	1	0	0	3	0	1
0	0	0	0	0	0	0	0	0	0	0	0	0	0	1	0	0	0	0	0	0	0	0
4258	4858	2042	3243	3913	3934	1563	3000	3124	711	2185	199	1250	1859	318	691	84	83	8	263	46	623	102
131	306	10	79	53	101	112	54	99	23	96	13	95	4	7	5	12	34	1	9	5	12	16
0	0	0	0	0	0	0	0	0	0	0	0	0	0	0	0	0	0	0	0	0	0	0
392	1113	160	431	442	364	833	784	505	617	1526	2918	2881	2070	490	560	804	2111	7292	2099	2622	806	4862
0	0	0	0	0	1	0	0	0	0	0	0	0	0	1	0	0	0	0	0	0	0	0
0	0	0	0	0	0	0	0	0	0	0	0	0	0	0	0	0	0	0	0	0	0	0
0	0	0	0	0	0	0	0	0	0	0	0	0	0	0	0	0	0	0	0	1	0	0
0	0	0	0	0	0	0	0	0	0	0	0	0	0	0	0	0	0	0	0	0	0	0
0	0	0	0	0	0	0	0	0	0	0	0	0	0	5	0	1	0	0	0	0	0	0

C1.026	C1.027	C1.029	C1.030	C1.031	C1.032	C1.033	C1.034	C1.036	C1.037	C1.038	C2.002	C2.003	C2.006	C2.009	C2.010	C2.012	C2.013	C2.014	C2.016	C2.017	C2.018	C2.019
Oral Ca	Oral Ca	Oral Ca	Oral Ca	Oral Ca	Oral Ca	Oral Ca	Oral Ca	Oral Ca	Oral Ca	Oral Ca	Oral Ca	Oral Ca	Oral Ca	Oral Ca	Oral Ca	Oral Ca	Oral Ca	Oral Ca	Oral Ca	Oral Ca	Oral Ca	Oral Ca
Sa	Sa	Sa	Sa	Sa	Sa	Sa	Sa	Sa	Sa	Sa	TD	TD	TD	TD	TD	TD	TD	TD	TD	TD	TD	TD
2	1	2	0	0	0	0	0	0	0	0	1	0	0	6	0	1	0	1	0	0	1	0
0	0	0	0	0	0	0	0	0	0	0	0	0	0	0	0	0	0	0	0	0	0	0
0	0	0	0	0	0	0	0	0	0	0	0	0	0	0	0	0	0	0	0	0	0	0
0	0	0	0	0	0	0	0	0	0	0	0	0	0	0	0	0	0	0	0	0	0	0
0	0	0	0	0	0	0	0	0	0	0	0	0	0	0	0	0	0	0	0	0	0	0
0	0	0	0	0	0	0	0	0	0	0	0	0	0	0	0	0	0	0	0	0	0	0
0	0	1	0	0	0	0	0	0	0	0	0	0	0	0	0	0	0	0	0	0	0	0
0	0	0	0	0	0	0	0	0	0	0	0	0	0	0	0	0	0	0	0	0	0	0
0	0	0	0	0	0	0	0	0	0	0	0	0	0	0	0	0	0	0	0	0	0	0
0	0	0	0	0	0	0	0	0	0	0	0	0	0	0	0	0	0	0	0	0	0	0
0	0	0	0	0	0	0	0	0	0	0	0	0	0	0	0	0	0	0	0	0	0	0
0	0	0	0	0	0	0	0	0	0	0	0	0	0	0	0	0	0	0	0	0	0	0
0	1	13	0	0	0	0	0	0	0	1	0	0	0	0	0	2	0	0	0	0	0	0
0	0	0	0	0	0	0	0	0	0	0	0	0	0	0	0	0	0	0	0	0	0	0
0	0	1	0	0	0	0	0	0	0	0	0	0	0	0	0	0	0	0	0	0	0	0
0	0	0	0	0	0	0	0	0	0	0	0	0	0	0	0	0	0	0	0	0	0	0
0	0	0	0	0	0	0	0	0	0	0	0	0	0	0	0	0	0	0	0	0	0	0
10	30	0	2	3	1	2	3	5	5	5	23	19	46	8	23	1	8	2	7	4	30	4
0	0	0	0	0	0	0	0	0	0	0	0	0	0	0	0	0	0	0	0	0	0	0
143	239	17	40	53	59	22	24	82	22	86	84	75	138	76	81	79	44	38	57	34	119	82
1	0	5	0	0	0	0	0	0	0	0	0	0	0	0	0	0	0	0	0	0	0	0
0	0	0	0	0	0	0	0	0	0	0	0	0	0	0	0	0	0	0	0	0	0	0
0	0	0	0	0	0	0	0	0	0	0	0	0	0	0	0	0	0	0	0	0	0	0
1	0	2	0	0	0	0	0	0	0	0	0	0	0	0	2	0	0	0	0	0	0	0
0	0	2	0	0	0	0	0	0	0	0	0	0	0	0	0	0	0	0	0	0	0	0
0	0	0	0	0	0	0	0	0	0	0	0	0	0	0	0	0	0	0	0	0	0	0
0	0	0	0	0	0	0	0	0	0	0	0	0	0	0	0	0	0	0	0	0	0	0
0	0	0	0	0	0	0	0	0	0	0	0	0	0	0	0	0	1	0	0	0	0	0
0	1	0	0	0	1	0	0	1	1	0	0	0	0	0	0	0	0	0	0	0	0	0
0	0	0	0	0	0	0	0	0	0	0	0	0	0	0	0	0	0	0	0	0	0	0
0	0	0	0	0	0	0	0	0	0	0	0	0	0	0	0	0	0	0	0	0	0	0
0	0	0	0	0	0	0	0	0	0	0	0	0	0	0	0	0	0	0	0	0	0	0
0	0	0	0	0	0	0	0	0	0	0	0	0	0	0	0	0	0	0	0	0	0	0
0	0	0	0	0	0	0	0	0	0	0	0	0	0	0	0	0	0	0	0	0	0	0
0	1	0	0	0	0	0	0	0	0	0	0	0	0	1	0	0	0	0	0	0	0	0
0	0	2	0	0	0	0	0	0	0	0	0	0	0	0	0	0	0	0	0	0	0	0
0	0	0	0	0	0	0	0	0	0	0	0	0	0	0	0	0	0	0	0	0	0	0

C1.026	C1.027	C1.029	C1.030	C1.031	C1.032	C1.033	C1.034	C1.036	C1.037	C1.038	C2.002	C2.003	C2.006	C2.009	C2.010	C2.012	C2.013	C2.014	C2.016	C2.017	C2.018	C2.019
Oral Ca	Oral Ca	Oral Ca	Oral Ca	Oral Ca	Oral Ca	Oral Ca	Oral Ca	Oral Ca	Oral Ca	Oral Ca	Oral Ca	Oral Ca	Oral Ca	Oral Ca	Oral Ca	Oral Ca	Oral Ca	Oral Ca	Oral Ca	Oral Ca	Oral Ca	Oral Ca
Sa	Sa	Sa	Sa	Sa	Sa	Sa	Sa	Sa	Sa	Sa	TD	TD	TD	TD	TD	TD	TD	TD	TD	TD	TD	TD
0	0	0	0	0	0	0	0	0	0	0	0	0	0	0	0	0	0	0	0	0	0	0
0	0	0	0	0	0	0	0	0	0	0	0	0	0	0	0	0	0	0	0	0	0	0
0	13	6	0	0	0	0	0	0	0	0	0	0	0	0	7	6	0	0	0	0	0	0
0	0	0	0	0	0	0	0	0	0	0	0	0	0	0	0	0	0	0	0	0	0	0
0	0	0	0	0	0	0	0	0	0	0	0	0	0	0	0	0	0	0	0	0	0	0
0	0	0	0	0	0	0	0	0	0	0	0	0	0	0	0	0	0	0	0	0	0	0
0	0	0	0	0	0	0	0	0	0	0	0	0	0	0	0	1	0	0	0	0	0	0
0	0	0	0	0	0	0	0	0	0	0	0	0	0	0	0	0	0	0	0	0	0	0
0	0	0	0	0	0	0	0	0	0	0	0	0	0	0	0	0	0	0	0	0	0	0
0	0	2	0	0	0	0	0	0	0	0	0	0	0	1	0	0	0	0	0	0	0	0
0	0	0	0	0	0	0	0	0	0	0	0	0	0	0	0	0	0	0	0	0	0	0
0	0	0	0	0	0	0	0	0	0	0	0	0	0	0	0	0	0	0	0	0	0	0
0	0	0	0	0	0	0	0	0	0	0	0	0	0	0	0	0	0	0	0	0	0	0
0	0	0	0	0	0	0	0	0	0	0	0	0	0	0	0	0	0	0	0	0	0	0
0	0	1	0	0	0	0	0	0	0	0	0	0	0	0	2	0	0	0	0	0	0	0
0	0	0	0	0	0	0	0	0	0	0	0	0	0	0	0	0	0	0	0	0	0	0
0	0	0	0	0	0	0	0	0	0	0	0	0	0	0	0	0	0	0	0	0	0	0
0	0	0	0	0	0	0	0	0	0	0	0	0	0	0	0	0	0	0	0	0	0	0
0	0	0	0	0	0	0	0	0	0	0	0	0	0	0	0	0	0	0	0	0	0	0
0	0	4	0	0	0	0	0	0	0	0	0	0	0	0	0	0	0	0	0	0	0	0
0	0	0	0	0	0	0	0	0	0	0	0	0	0	0	0	0	0	0	0	0	0	0
0	0	0	0	0	0	0	0	0	0	0	0	0	0	0	1	0	0	0	0	0	0	0
1	1	3	0	0	0	0	0	0	0	0	0	0	0	0	11	0	0	0	0	0	0	0
0	0	0	0	0	0	0	0	0	0	0	0	0	0	0	0	0	0	0	0	0	0	0
6	13	23	7	4	2	9	15	9	10	5	117	65	57	20	17	17	123	7	58	67	121	23
0	0	0	0	0	0	0	0	0	0	0	0	0	0	0	0	0	0	0	0	0	0	0
1	0	15	17	18	12	2	19	57	9	9	3	2	4	14	9	47	0	89	2	1	23	4
5	1	11	0	0	0	0	0	0	0	0	0	0	0	0	59	33	0	0	0	0	0	0
0	0	0	0	0	0	0	0	0	0	0	0	0	0	0	0	0	0	0	0	0	0	0
0	0	0	0	0	0	0	0	0	0	0	0	0	0	0	0	0	0	0	0	0	0	0
0	0	0	0	0	0	0	0	0	0	0	0	0	0	0	0	0	0	0	0	0	0	0
0	0	0	0	0	0	0	0	0	0	0	0	0	0	0	0	0	0	0	0	0	0	0
0	0	0	0	0	0	0	0	0	0	0	0	0	0	0	0	2	0	0	0	0	0	0
0	0	0	0	0	0	0	0	0	0	0	0	0	0	0	0	0	0	0	0	0	0	0
0	0	5	1	0	0	0	0	0	0	0	0	0	0	0	0	0	0	0	0	0	0	0
0	0	0	0	0	0	0	0	0	0	0	0	0	0	0	0	0	0	0	0	0	0	0
34	15	43	55	78	69	87	89	55	27	163	304	285	227	749	179	155	542	319	567	423	783	573

C1.026 Oral Ca Sa	C1.027 Oral Ca Sa	C1.029 Oral Ca Sa	C1.030 Oral Ca Sa	C1.031 Oral Ca Sa	C1.032 Oral Ca Sa	C1.033 Oral Ca Sa	C1.034 Oral Ca Sa	C1.036 Oral Ca Sa	C1.037 Oral Ca Sa	C1.038 Oral Ca Sa	C2.002 Oral Ca TD	C2.003 Oral Ca TD	C2.006 Oral Ca TD	C2.009 Oral Ca TD	C2.010 Oral Ca TD	C2.012 Oral Ca TD	C2.013 Oral Ca TD	C2.014 Oral Ca TD	C2.016 Oral Ca TD	C2.017 Oral Ca TD	C2.018 Oral Ca TD	C2.019 Oral Ca TD
0	0	0	0	0	0	0	0	0	0	0	0	0	0	0	0	0	0	0	0	0	0	0
0	0	0	0	0	0	0	0	0	0	0	0	0	0	0	0	0	0	0	0	0	0	0
0	0	0	0	0	0	0	0	0	0	0	0	0	0	0	0	0	0	0	0	0	0	0
0	0	0	0	0	0	0	0	0	0	0	0	0	0	0	5	1	0	0	0	0	0	0
0	0	0	0	0	0	0	0	0	0	0	0	0	0	0	0	0	0	0	0	0	0	0
0	0	8	0	0	0	0	0	0	0	0	0	0	0	0	0	0	0	0	0	0	0	0
0	0	0	0	0	0	0	0	0	0	0	0	0	0	0	1	0	0	0	0	0	0	0
161	86	406	0	0	0	0	0	4	0	0	0	2	0	13	403	58	124	4	0	12	1	121
0	0	1	0	0	0	0	0	3	0	0	0	0	0	0	0	0	0	0	0	0	0	0
0	0	0	0	0	0	0	0	0	0	0	0	0	0	0	0	0	0	0	0	0	0	0
248	120	328	375	433	407	270	579	991	593	761	1451	707	777	888	311	618	1881	1815	857	4924	1321	1445
0	0	0	0	0	0	0	0	0	0	0	0	0	0	0	0	0	0	0	0	0	0	0
0	0	12	0	0	0	1	0	0	0	7	0	0	0	4	11	6	1	0	0	0	1	1
0	0	0	0	0	0	0	0	0	0	0	0	0	0	0	0	0	0	0	0	0	0	0
0	0	5	0	0	0	0	0	0	0	1	0	0	0	0	0	0	0	0	0	0	0	0
0	0	0	0	0	0	0	1	0	0	0	0	0	0	0	0	0	0	0	0	0	0	0
2	1	0	3	1	0	0	2	0	0	0	3	8	40	2	149	3	0	0	0	0	0	0
0	0	0	0	0	1	0	5	0	2	0	75	75	12	50	0	9	310	8	70	140	141	81
0	3	0	0	1	0	0	0	0	0	1	2	0	0	0	1	0	1	0	0	2	1	0
19	22	14	14	40	24	23	51	13	46	48	131	94	20	25	27	46	32	124	102	124	32	80
0	0	0	0	0	0	0	0	0	0	0	0	0	0	0	0	0	0	0	0	0	0	0
0	4	2	0	0	0	0	0	0	0	0	0	0	0	0	8	2	0	0	0	0	0	0
13	2	0	0	0	0	0	0	0	0	0	0	0	0	0	0	0	0	0	0	0	0	0
0	0	0	0	0	0	0	0	0	0	0	0	0	0	0	0	0	0	0	0	0	0	0
12	13	0	0	2	1	30	0	4	3	1	1	45	27	5	35	7	7	0	2	1	159	1
3	2	0	0	0	0	0	0	0	0	0	0	0	0	0	5	1	0	0	0	0	0	0
0	0	0	0	0	0	0	0	0	0	0	0	0	0	0	0	0	0	0	0	0	0	0
0	0	0	0	0	0	0	0	0	0	0	0	0	0	0	0	0	0	0	0	0	0	0
0	0	0	0	0	0	0	0	0	0	0	0	0	0	0	0	0	0	0	0	0	0	0
0	1	0	0	2	2	1	1	0	1	0	0	3	0	1	0	0	0	0	0	0	0	0
0	0	0	0	0	0	0	0	0	0	0	0	0	0	1	0	0	0	0	0	0	0	0
0	0	0	0	0	0	0	0	0	0	0	0	0	0	1	0	0	0	0	0	0	0	0
0	0	0	0	0	0	0	0	0	0	0	0	0	0	0	0	0	0	0	0	0	0	0
1	40	7	0	0	0	0	0	0	0	0	0	0	0	2	60	12	0	0	0	0	0	0
0	0	0	0	0	0	0	0	0	0	0	0	0	0	0	0	0	0	0	0	0	0	0
0	0	0	0	0	0	0	0	0	0	0	0	0	0	0	0	0	0	0	0	0	0	0
0	0	0	0	0	0	0	0	0	0	0	0	0	0	0	0	0	0	0	0	0	0	0

C1.026	C1.027	C1.029	C1.030	C1.031	C1.032	C1.033	C1.034	C1.036	C1.037	C1.038	C2.002	C2.003	C2.006	C2.009	C2.010	C2.012	C2.013	C2.014	C2.016	C2.017	C2.018	C2.019
Oral Ca	Oral Ca	Oral Ca	Oral Ca	Oral Ca	Oral Ca	Oral Ca	Oral Ca	Oral Ca	Oral Ca	Oral Ca	Oral Ca	Oral Ca	Oral Ca	Oral Ca	Oral Ca	Oral Ca	Oral Ca	Oral Ca	Oral Ca	Oral Ca	Oral Ca	Oral Ca
Sa	Sa	Sa	Sa	Sa	Sa	Sa	Sa	Sa	Sa	Sa	TD	TD	TD	TD	TD	TD	TD	TD	TD	TD	TD	TD
1	1	1	0	0	0	0	0	0	0	0	0	0	0	0	0	0	0	0	0	0	0	0
0	0	0	0	0	0	0	0	0	0	0	0	0	0	0	0	0	0	0	0	0	0	0
0	0	0	0	0	0	0	0	0	0	0	0	0	0	0	0	0	0	0	0	0	0	0
0	0	0	0	0	0	0	0	0	0	0	0	0	0	0	0	0	0	0	0	0	0	0
0	0	0	0	1	0	0	0	0	1	0	0	0	0	2	0	0	18	1	0	3	3	0
0	0	0	0	0	0	0	0	0	0	0	1	0	0	0	0	0	0	0	0	0	0	0
0	0	0	0	0	1	0	0	0	0	0	0	0	0	0	0	0	0	0	0	0	0	0
1	1	1	0	0	0	0	1	0	0	0	0	0	0	4	1	0	1	0	0	0	0	0
1	17	69	15	29	22	35	15	4	22	14	10	6	14	9	0	5	5	7	37	1	1	18
10	7	2	2	2	2	3	4	7	0	5	38	38	15	28	65	26	27	99	132	16	75	47
2	1	3	0	0	0	0	0	0	0	0	0	0	0	6	1	0	0	0	0	0	0	0
0	0	0	0	0	0	0	0	0	0	0	0	0	0	3	0	0	0	0	0	0	0	0
0	0	0	0	0	0	0	0	0	0	0	0	0	0	1	0	0	0	0	0	0	0	0
0	0	0	0	0	0	0	0	0	0	0	0	0	0	3	0	0	0	0	0	0	0	0
1	0	0	0	0	0	0	0	0	0	0	0	0	0	2	0	0	0	0	0	0	0	0
0	10	1	0	0	1	0	1	0	0	1	0	0	0	0	0	0	1	0	0	1	0	0
24	6	70	44	40	41	106	77	20	66	408	195	76	98	83	4	140	97	183	122	267	90	459
2	2	0	0	0	0	0	0	0	0	0	0	0	0	4	0	0	1	0	0	0	0	0
19	121	15	0	19	20	67	203	81	27	51	40	2	0	7	54	4	63	1	0	2	8	93
0	1	1	0	0	0	1	1	0	3	3	1	0	0	2	0	0	1	0	0	0	0	0
37	66	8	34	32	32	16	63	48	20	80	248	139	146	356	190	145	196	342	174	431	487	243
0	0	0	0	0	0	0	0	0	0	0	0	0	0	0	0	0	0	0	0	0	0	0
2	6	0	5	6	5	0	6	4	1	12	4	15	2	8	4	0	0	0	5	3	14	0
0	0	0	0	0	0	0	0	0	0	0	0	0	0	0	0	0	0	0	0	0	0	0
0	0	0	0	0	0	0	0	0	0	0	0	0	0	0	0	0	0	0	0	0	0	0
9	10	0	3	6	4	3	4	11	1	1	14	15	0	4	66	0	23	2	0	15	189	2
12	8	3	6	5	3	6	1	2	3	14	34	36	97	26	40	11	14	137	31	8	46	25
4	3	0	1	1	0	2	1	1	0	3	1	3	17	0	15	0	0	0	2	3	85	1
0	0	0	0	0	0	0	0	0	0	0	0	0	0	0	0	0	0	0	0	0	0	0
0	0	0	0	0	0	0	0	0	0	0	0	0	0	0	0	0	0	0	0	0	0	0
4	1	0	0	0	0	0	0	1	0	0	0	1	0	28	0	0	0	0	0	0	0	0
2	3	1	0	0	0	0	3	0	0	0	0	0	0	10	0	0	1	0	0	0	0	0
0	0	0	0	0	0	0	0	0	0	0	0	0	0	2	0	0	1	0	0	0	0	0
0	0	0	0	0	0	0	0	0	0	0	0	0	0	0	0	0	0	0	0	0	0	0
10	3	2	2	2	2	1	0	0	0	1	0	0	4	22	0	0	0	0	0	0	0	0
0	0	0	0	0	5	0	1	0	0	0	0	0	0	0	0	0	1	0	0	0	0	0
0	0	0	0	0	0	0	0	0	0	0	0	0	0	0	0	0	0	0	0	0	0	0

C1.026	C1.027	C1.029	C1.030	C1.031	C1.032	C1.033	C1.034	C1.036	C1.037	C1.038	C2.002	C2.003	C2.006	C2.009	C2.010	C2.012	C2.013	C2.014	C2.016	C2.017	C2.018	C2.019
Oral Ca	Oral Ca	Oral Ca	Oral Ca	Oral Ca	Oral Ca	Oral Ca	Oral Ca	Oral Ca	Oral Ca	Oral Ca	Oral Ca	Oral Ca	Oral Ca	Oral Ca	Oral Ca	Oral Ca	Oral Ca	Oral Ca	Oral Ca	Oral Ca	Oral Ca	Oral Ca
Sa	Sa	Sa	Sa	Sa	Sa	Sa	Sa	Sa	Sa	Sa	TD	TD	TD	TD	TD	TD	TD	TD	TD	TD	TD	TD
14	32	8	14	23	21	32	68	9	5	14	26	11	0	8	8	1	152	22	0	5	14	97
0	0	0	0	0	0	0	0	0	0	0	0	0	0	0	0	0	0	0	0	0	0	0
2	26	21	11	14	7	18	14	3	92	107	17	175	0	3	56	12	203	265	20	272	0	265
0	0	0	0	0	0	0	0	0	0	0	0	0	0	0	0	0	0	0	0	0	0	0
2	18	0	12	28	10	38	12	8	19	12	0	0	0	1	2	0	1	0	0	1	0	1
112	103	40	187	223	128	553	135	68	252	226	19	19	13	2	19	7	35	23	80	17	2	162
0	0	0	0	0	0	0	0	0	0	0	0	0	0	0	0	0	0	0	0	0	0	0
0	0	0	0	0	0	0	0	0	0	0	0	0	0	0	0	0	0	0	0	0	0	0
7	2	15	19	8	6	27	32	10	33	86	249	697	514	121	39	134	777	1965	627	1165	159	1284
0	0	0	0	0	0	0	0	0	0	5	0	1	0	0	0	0	0	0	0	0	0	0
0	0	0	0	7	6	51	2	2	0	1	0	7	0	0	4	1	3	0	0	8	0	160
0	2	1	0	0	0	0	0	0	0	0	0	0	0	1	0	0	0	0	0	0	0	0
0	0	0	0	0	0	0	0	0	0	0	0	0	0	0	0	0	0	0	0	0	0	0
4	0	0	0	0	0	0	0	0	0	0	0	0	0	0	0	0	0	0	0	0	0	0
0	0	1	0	0	0	0	0	0	0	0	0	0	0	1	0	0	1	0	0	1	0	0
9	3	10	23	16	13	37	43	16	42	98	122	111	98	84	21	73	165	851	86	237	130	261
0	0	0	0	0	0	0	0	0	0	0	0	0	0	0	0	0	0	0	0	0	0	0
0	0	0	0	0	0	0	0	0	0	0	0	0	0	0	0	0	0	0	0	0	0	0
0	0	0	0	0	0	0	0	0	0	0	0	0	0	0	0	0	0	0	0	0	0	0
0	0	0	0	0	0	0	0	0	0	0	0	0	0	0	0	0	0	0	0	0	0	0
0	1	0	0	0	0	0	0	0	1	0	0	0	0	0	3	0	0	0	0	0	0	0
1	4	0	0	0	0	0	0	0	0	0	1	3	0	1	0	0	0	0	0	2	2	0
0	1	0	0	0	0	0	0	0	0	0	0	0	0	0	0	0	0	0	0	0	0	0
0	0	0	0	0	1	4	6	2	0	0	0	0	0	1	0	0	0	0	0	0	0	0
0	0	0	0	0	0	0	0	0	0	0	0	0	0	0	0	0	0	0	0	0	0	0
107	113	37	13	18	13	54	37	39	4	57	450	628	370	60	1241	265	122	193	522	115	787	303
19	106	9	15	6	8	22	7	8	7	19	203	475	381	62	52	128	83	83	137	258	102	390
0	4	3	0	0	0	0	0	0	0	0	0	0	0	0	18	3	0	0	0	0	0	0
0	0	0	0	0	0	0	0	0	0	0	0	0	0	0	0	0	0	0	0	0	0	0
0	0	0	0	0	0	3	0	0	0	0	2	1	0	0	3	0	1	0	0	0	0	0
0	0	0	0	0	0	0	0	0	0	0	0	0	0	0	0	0	0	0	0	0	0	0
0	0	0	0	0	0	0	0	0	0	0	0	0	0	0	0	0	0	0	0	0	0	0
0	0	0	0	0	0	0	0	0	0	0	0	0	0	0	0	0	0	0	0	0	0	0
0	0	0	0	0	0	0	0	0	0	0	0	0	0	0	0	0	0	0	0	0	0	0
0	1	2	0	0	0	0	0	0	0	0	0	0	0	0	0	0	0	0	0	4	0	0
1	0	2	0	0	0	0	0	0	0	0	0	0	0	0	0	0	0	0	0	0	0	0
0	0	0	0	0	0	0	0	0	0	0	0	0	0	0	0	0	0	0	0	0	0	0

C1.026	C1.027	C1.029	C1.030	C1.031	C1.032	C1.033	C1.034	C1.036	C1.037	C1.038	C2.002	C2.003	C2.006	C2.009	C2.010	C2.012	C2.013	C2.014	C2.016	C2.017	C2.018	C2.019
Oral Ca	Oral Ca	Oral Ca	Oral Ca	Oral Ca	Oral Ca	Oral Ca	Oral Ca	Oral Ca	Oral Ca	Oral Ca	Oral Ca	Oral Ca	Oral Ca	Oral Ca	Oral Ca	Oral Ca	Oral Ca	Oral Ca	Oral Ca	Oral Ca	Oral Ca	Oral Ca
Sa	Sa	Sa	Sa	Sa	Sa	Sa	Sa	Sa	Sa	Sa	TD	TD	TD	TD	TD	TD	TD	TD	TD	TD	TD	TD
0	10	9	1	0	0	0	0	0	0	0	0	0	0	0	0	1	0	0	0	0	0	0
0	0	0	0	0	0	0	0	0	0	0	0	0	0	0	0	0	0	0	0	0	0	0
0	9	38	0	1	0	1	0	4	0	0	0	0	0	2	3	2	0	1	39	105	75	29
0	0	0	0	0	0	0	0	0	0	0	0	0	0	0	0	0	0	0	0	0	0	0
0	0	0	0	0	0	0	0	0	0	0	0	0	0	0	0	0	0	0	0	0	0	0
0	0	0	0	0	0	0	0	0	0	0	0	0	0	0	0	0	0	0	0	0	0	0
0	0	0	0	0	0	0	0	0	0	0	0	0	0	0	0	0	0	0	0	0	0	0
0	0	0	0	0	0	0	0	0	0	0	0	0	0	0	0	0	0	0	0	0	0	0
0	0	0	0	0	0	0	0	0	0	0	0	0	0	0	0	0	0	0	0	0	0	0
0	0	0	0	0	0	0	0	0	0	0	0	0	0	0	1	0	0	0	0	0	0	0
0	0	0	0	0	0	0	0	0	0	0	0	0	0	0	0	0	0	0	0	0	0	0
0	0	14	0	1	0	0	0	0	0	0	0	0	0	0	0	1	0	0	0	0	1	0
0	0	0	0	0	0	0	0	0	0	0	0	0	0	0	0	0	0	0	0	0	0	0
0	0	0	0	0	0	0	0	0	0	0	0	0	0	0	0	0	0	0	0	0	0	0
0	3	26	0	0	0	0	1	0	0	0	0	0	0	1	3	1	1	0	0	0	0	0
0	0	1	0	0	0	0	0	0	0	0	0	0	0	0	0	0	0	0	0	0	0	0
0	0	1	0	0	0	0	0	0	0	0	0	0	0	0	0	0	0	0	0	0	0	0
0	0	0	0	0	0	0	0	0	0	0	0	0	0	0	0	0	0	0	0	0	0	0
0	0	2	0	0	0	0	0	0	0	0	0	0	0	0	0	0	0	0	0	0	0	0
0	0	0	0	0	0	0	0	0	0	0	0	0	0	0	0	0	0	0	0	0	0	0
0	0	0	0	0	0	0	0	0	0	0	0	0	0	0	0	0	0	0	0	0	0	0
0	0	0	0	0	0	0	0	0	0	0	0	0	0	0	0	0	0	0	0	0	0	0
0	1	1	0	0	0	0	0	2	0	0	1	0	0	0	0	1	0	0	0	0	0	0
0	1	2	0	0	0	0	0	0	0	0	0	0	0	0	0	0	0	0	0	0	0	0
0	0	0	0	0	0	0	0	0	0	0	0	0	0	0	0	0	0	0	0	0	0	0
0	0	0	0	0	0	0	0	0	0	0	0	0	0	0	0	0	0	0	0	0	0	0
0	0	0	0	0	0	0	0	0	0	0	0	0	0	0	0	0	0	0	0	0	0	0
0	0	1	0	0	0	0	0	0	0	0	0	0	0	0	0	0	0	0	0	0	0	0
0	0	0	0	0	0	0	0	0	0	0	0	0	0	0	0	0	0	0	0	0	0	0
0	0	3	0	0	0	0	0	0	0	0	0	0	0	0	0	0	0	0	0	0	0	0
0	0	1	0	0	0	0	0	0	0	0	0	0	0	0	0	0	0	0	0	0	0	1
0	0	0	0	0	0	0	0	0	0	0	0	0	0	0	0	0	0	0	0	0	0	0
0	0	0	0	0	0	0	0	0	0	0	0	0	0	0	0	0	0	0	0	0	0	0
0	0	0	0	0	0	0	0	0	0	0	0	0	0	0	0	0	0	0	0	0	0	0
0	0	3	0	0	0	0	0	0	0	0	0	0	0	0	0	0	0	0	0	0	0	0
0	0	0	0	0	0	0	0	0	0	0	0	0	0	0	0	0	0	0	0	0	0	0
0	0	0	0	0	0	0	0	0	0	0	0	0	0	0	0	0	0	0	0	0	0	0

C1.026	C1.027	C1.029	C1.030	C1.031	C1.032	C1.033	C1.034	C1.036	C1.037	C1.038	C2.002	C2.003	C2.006	C2.009	C2.010	C2.012	C2.013	C2.014	C2.016	C2.017	C2.018	C2.019
Oral Ca	Oral Ca	Oral Ca	Oral Ca	Oral Ca	Oral Ca	Oral Ca	Oral Ca	Oral Ca	Oral Ca	Oral Ca	Oral Ca	Oral Ca	Oral Ca	Oral Ca	Oral Ca	Oral Ca	Oral Ca	Oral Ca	Oral Ca	Oral Ca	Oral Ca	Oral Ca
Sa	Sa	Sa	Sa	Sa	Sa	Sa	Sa	Sa	Sa	Sa	TD	TD	TD	TD	TD	TD	TD	TD	TD	TD	TD	TD
0	0	0	0	0	0	0	0	0	0	0	0	0	0	0	0	0	0	0	0	0	0	0
0	0	0	0	0	0	0	0	0	0	0	0	0	0	0	0	0	0	0	0	0	0	0
0	0	2	0	0	0	0	0	0	0	0	0	0	0	0	0	0	0	0	0	0	0	0
0	0	0	0	0	0	0	0	0	0	0	0	0	0	0	0	0	0	0	0	0	0	0
0	0	0	0	0	0	0	0	0	0	0	0	0	0	0	0	1	0	0	0	0	0	0
0	0	0	0	0	0	0	0	0	0	0	0	0	0	0	0	0	0	0	0	0	0	0
0	0	0	0	0	0	0	0	0	0	0	0	0	0	0	0	0	0	0	0	0	0	0
0	0	0	0	0	0	0	0	0	0	0	0	0	0	0	0	0	0	0	0	0	0	0
0	0	1	0	0	0	0	0	0	0	0	0	0	0	0	0	0	0	0	0	0	0	0
0	0	0	0	0	0	0	0	0	0	0	0	0	0	0	0	0	0	0	0	0	0	0
0	0	0	0	0	0	0	0	0	0	0	0	0	0	0	0	0	0	0	0	0	0	0
0	0	0	0	0	0	0	0	0	0	0	0	0	0	0	0	0	0	0	0	0	0	0
0	0	0	0	0	0	0	0	0	0	0	0	0	0	0	0	0	0	0	0	0	0	0
0	0	3	0	0	0	0	0	0	0	0	0	0	0	0	0	0	0	0	0	0	0	0
0	0	0	0	0	0	0	0	0	0	0	0	0	0	0	0	0	0	0	0	0	0	0
0	0	3	0	0	0	0	0	0	0	0	0	0	0	0	0	0	0	0	0	0	0	0
0	0	1	0	0	0	0	0	0	0	0	0	0	0	0	0	0	0	0	0	0	0	0
0	0	0	0	0	0	0	0	0	0	0	0	0	0	0	0	0	0	0	0	0	0	0
0	0	0	0	0	0	0	0	0	0	0	0	0	0	0	0	0	0	1	0	0	0	0
0	0	5	0	0	0	0	0	1	0	0	1	0	0	0	0	0	0	0	0	0	0	0
0	0	0	0	0	0	0	0	0	0	0	0	0	0	0	0	0	0	0	0	0	0	0
0	0	0	0	0	0	0	0	0	0	0	0	0	0	0	0	0	0	0	0	0	0	0
0	0	0	0	0	0	0	0	0	0	0	0	0	0	0	0	0	0	0	0	0	0	0
0	0	0	0	0	0	0	0	0	0	0	0	0	0	0	0	0	0	0	0	0	0	0
0	0	2	0	0	0	0	0	0	0	0	0	0	0	0	0	0	0	0	0	0	0	0
0	0	0	0	0	0	0	0	0	0	0	0	0	0	0	0	0	0	0	0	0	0	0
0	0	0	0	0	0	0	0	0	0	0	0	0	0	0	0	0	0	0	0	0	0	0
0	3	16	0	0	0	0	0	1	0	0	0	1	0	0	0	0	0	0	0	0	0	1
0	0	3	0	0	0	0	0	0	0	0	0	0	0	0	0	0	0	0	0	0	0	0
42	70	149	0	0	0	0	0	1	0	0	0	0	1	0	0	7	1	0	0	1	0	0
0	0	0	0	0	0	0	0	0	0	0	0	0	0	0	0	0	0	0	0	0	0	0
0	0	3	0	0	0	0	0	0	0	0	0	0	0	0	0	0	0	0	0	0	0	0
0	0	1	0	0	0	0	0	0	0	0	0	0	0	0	0	0	0	0	0	0	0	0
0	0	0	0	0	0	0	0	0	0	0	0	0	0	0	0	0	0	0	0	0	0	0
0	0	0	0	0	0	0	0	0	0	0	0	0	0	0	0	0	0	0	0	0	0	0
0	0	1	0	0	0	0	0	0	0	0	0	0	0	0	0	0	0	0	0	0	0	0
3	1	0	0	1	0	0	2	0	0	0	0	0	0	7	0	0	0	0	0	0	0	0

C1.026	C1.027	C1.029	C1.030	C1.031	C1.032	C1.033	C1.034	C1.036	C1.037	C1.038	C2.002	C2.003	C2.006	C2.009	C2.010	C2.012	C2.013	C2.014	C2.016	C2.017	C2.018	C2.019
Oral Ca	Oral Ca	Oral Ca	Oral Ca	Oral Ca	Oral Ca	Oral Ca	Oral Ca	Oral Ca	Oral Ca	Oral Ca	Oral Ca	Oral Ca	Oral Ca	Oral Ca	Oral Ca	Oral Ca	Oral Ca	Oral Ca	Oral Ca	Oral Ca	Oral Ca	Oral Ca
Sa	Sa	Sa	Sa	Sa	Sa	Sa	Sa	Sa	Sa	Sa	TD	TD	TD	TD	TD	TD	TD	TD	TD	TD	TD	TD
0	0	1	0	0	0	0	0	0	0	0	0	0	0	0	0	0	0	0	0	0	0	0
0	0	1	0	0	0	0	0	0	0	0	0	0	0	0	0	1	0	0	0	0	0	0
0	0	0	0	0	0	0	0	0	0	0	0	0	0	0	0	0	0	0	0	0	0	0
216	301	637	262	95	84	243	248	581	89	125	7	10	9	1	33	21	0	65	9	71	7	5
0	0	0	0	0	0	0	0	0	0	0	0	0	0	0	0	0	0	0	0	0	0	0
0	0	0	0	0	0	0	0	0	0	0	0	0	0	0	0	0	0	0	0	0	0	0
0	1	3	0	0	0	0	0	0	0	0	0	0	0	0	0	0	0	0	0	0	0	0
0	0	1	0	0	0	0	0	0	0	0	0	0	0	0	0	0	0	0	0	0	0	0
0	0	0	0	0	0	0	0	0	0	0	0	0	0	0	0	0	0	0	0	0	0	0
0	1	0	0	0	0	0	0	0	0	0	0	0	0	0	0	0	0	0	0	0	0	0
0	0	0	0	0	0	0	0	0	0	0	0	0	0	0	0	0	0	0	0	0	0	0
1	0	0	0	0	0	0	0	1	0	0	0	0	0	0	0	0	0	0	0	0	0	0
0	0	1	0	0	0	0	0	0	0	0	0	0	0	0	0	0	0	0	0	0	0	0
0	0	1	0	0	0	0	0	0	0	0	0	0	0	0	0	0	0	0	0	0	0	0
0	0	2	0	0	0	0	0	1	0	1	0	0	0	0	0	0	0	0	0	0	0	0
0	0	0	0	0	0	0	0	0	0	0	0	0	0	0	0	0	0	0	0	0	0	0
0	0	0	0	0	0	0	0	0	0	0	0	0	0	0	0	0	0	0	0	0	0	0
0	0	0	0	0	0	0	0	0	0	0	0	0	0	0	0	0	0	0	0	0	0	0
0	0	0	0	0	0	0	0	0	0	0	0	0	0	0	0	0	0	0	0	0	0	0
0	0	0	0	0	0	0	0	0	0	0	0	0	0	0	0	0	0	0	0	0	0	0
0	0	0	0	0	0	0	0	0	0	0	0	0	0	0	0	0	0	0	0	0	0	0
8	211	3	12	0	0	0	6	119	0	7	0	0	0	0	0	2	0	0	0	0	0	0
99	1157	29	64	158	158	301	79	284	151	48	1	1	0	3	4	11	0	0	0	5	1	6
0	0	1	0	0	0	0	0	1	0	0	0	0	0	0	0	0	1	0	0	0	0	0
0	0	0	0	0	0	0	0	0	0	0	0	0	0	0	0	0	0	0	0	0	0	0
0	0	0	0	0	0	0	0	0	0	0	0	0	0	0	0	0	0	0	0	0	0	0
0	0	0	0	0	0	0	0	0	0	0	0	0	0	0	0	0	0	0	0	0	0	0
0	1	1	0	0	0	0	0	2	0	0	0	1	0	2	0	0	0	0	0	1	0	0
0	0	0	0	0	0	0	0	0	0	0	0	0	0	0	0	0	0	0	0	0	0	0
0	0	0	0	0	0	0	0	0	0	0	0	0	0	0	0	0	0	0	0	0	0	0
0	0	0	0	0	0	0	0	0	0	0	0	0	0	0	0	0	0	0	0	0	0	0
0	0	0	0	0	0	0	0	0	0	0	0	0	0	0	0	0	0	0	0	0	0	0
106	147	152	81	124	121	66	113	214	52	80	1	1	7	10	3	12	0	0	3	19	2	2
8	6	30	5	13	21	17	17	26	5	1	9	1	2	3	0	1	0	8	2	0	2	2
3684	1674	9023	6377	5042	4575	4716	3735	6636	2842	5031	9375	4327	10452	4199	3080	2369	171	1913	8117	3557	11984	1922
56	34	13	36	22	18	13	43	31	0	16	8	0	2	8	2	6	0	0	5	3	19	2
450	483	102	315	679	635	235	469	1082	217	406	159	50	21	28	58	31	6	143	7	95	15	9

C1.026 Oral Ca Sa	C1.027 Oral Ca Sa	C1.029 Oral Ca Sa	C1.030 Oral Ca Sa	C1.031 Oral Ca Sa	C1.032 Oral Ca Sa	C1.033 Oral Ca Sa	C1.034 Oral Ca Sa	C1.036 Oral Ca Sa	C1.037 Oral Ca Sa	C1.038 Oral Ca Sa	C2.002 Oral Ca TD	C2.003 Oral Ca TD	C2.006 Oral Ca TD	C2.009 Oral Ca TD	C2.010 Oral Ca TD	C2.012 Oral Ca TD	C2.013 Oral Ca TD	C2.014 Oral Ca TD	C2.016 Oral Ca TD	C2.017 Oral Ca TD	C2.018 Oral Ca TD	C2.019 Oral Ca TD
0	0	0	0	0	0	0	0	0	0	0	0	0	0	0	0	0	0	0	0	0	0	0
0	0	0	0	0	0	0	0	0	0	0	0	0	0	0	0	0	0	0	0	0	0	0
0	0	0	0	0	0	0	0	0	0	0	0	0	0	0	0	0	0	0	0	0	0	0
0	34	0	1	6	3	3	0	11	0	0	0	0	0	0	0	0	0	0	0	0	0	0
0	1	0	0	0	0	0	0	0	0	0	0	0	0	0	0	0	0	0	0	0	0	0
0	0	0	0	0	0	0	0	0	0	0	0	0	0	0	0	0	0	0	0	0	0	0
0	4	2	0	0	0	0	0	0	0	0	0	0	0	0	0	0	0	0	0	0	0	0
0	0	1	0	0	0	0	0	0	0	0	0	0	0	0	0	0	0	0	0	0	0	0
0	0	2	0	0	0	0	0	0	0	0	0	0	0	0	0	0	0	0	0	0	0	0
1	1	0	0	11	11	11	21	14	4	3	3	4	0	0	0	0	1	0	0	0	0	0
0	0	0	0	14	9	0	0	0	0	0	0	0	0	0	0	0	0	0	0	1	1	0
0	0	0	0	0	0	0	0	0	0	0	0	0	0	0	0	0	0	0	0	0	0	0
19	219	0	1	17	14	0	21	0	0	0	0	0	0	0	1	0	1	0	0	0	1	0
0	0	0	0	0	0	0	0	0	0	0	0	0	0	0	0	0	0	0	0	0	0	0
0	0	0	0	0	0	0	0	0	0	0	0	0	0	0	0	0	0	0	0	0	0	0
0	0	0	0	0	0	0	0	0	0	0	0	0	0	0	0	0	0	0	0	0	0	0
0	0	0	0	0	0	0	0	0	0	0	0	0	0	0	0	0	0	0	0	0	0	0
0	0	0	0	0	0	0	0	0	0	0	0	0	0	0	0	0	0	0	0	0	0	0
0	0	0	0	0	0	0	0	0	0	0	0	0	0	0	0	0	0	0	0	0	0	0
0	0	0	0	0	0	0	0	0	0	0	0	0	0	0	0	0	0	0	0	0	0	0
32	44	10	12	22	13	24	26	15	11	38	274	142	85	135	50	198	186	203	70	323	201	531
1	16	0	1	0	0	1	3	5	0	0	0	0	0	2	0	0	0	0	0	0	0	0
0	0	0	0	0	0	0	0	0	0	0	0	2	0	0	0	0	0	0	0	1	0	0
0	0	0	0	0	0	0	0	0	0	0	0	0	0	0	0	0	0	0	0	0	0	0
0	0	0	0	0	0	0	0	0	0	0	0	0	0	0	0	0	0	0	0	0	0	0
0	0	0	0	0	0	0	0	0	0	0	0	0	0	0	0	0	0	0	0	0	0	0
0	0	1	0	0	0	0	0	0	0	0	0	0	0	0	0	0	0	0	0	0	0	0
4	1	0	0	0	0	0	0	0	0	0	0	0	0	1	0	0	0	0	0	0	0	0
0	0	0	0	0	0	0	0	0	0	0	0	0	0	0	0	0	0	0	0	0	0	0
0	0	0	0	0	0	0	0	0	0	0	0	0	0	0	0	0	0	0	0	0	0	0
0	0	0	0	0	0	0	0	0	0	0	0	0	0	0	0	0	0	0	0	0	0	0
0	0	0	0	0	0	0	0	0	0	0	0	0	0	0	0	0	0	0	0	0	0	0
0	0	0	0	0	1	0	0	0	0	0	0	0	0	0	0	0	0	0	0	0	0	0
0	0	0	0	0	0	0	0	0	0	0	0	0	0	0	0	0	0	0	0	0	0	0
0	0	0	0	0	0	0	0	0	0	0	0	0	0	0	0	0	0	0	0	0	0	0
51	102	15	69	40	30	79	44	75	9	56	0	1	2	6	2	5	0	4	0	2	1	5
25	1	0	9	4	0	74	10	5	0	1	0	0	0	0	0	2	0	0	0	0	0	0

C1.026	C1.027	C1.029	C1.030	C1.031	C1.032	C1.033	C1.034	C1.036	C1.037	C1.038	C2.002	C2.003	C2.006	C2.009	C2.010	C2.012	C2.013	C2.014	C2.016	C2.017	C2.018	C2.019
Oral Ca	Oral Ca	Oral Ca	Oral Ca	Oral Ca	Oral Ca	Oral Ca	Oral Ca	Oral Ca	Oral Ca	Oral Ca	Oral Ca	Oral Ca	Oral Ca	Oral Ca	Oral Ca	Oral Ca	Oral Ca	Oral Ca	Oral Ca	Oral Ca	Oral Ca	Oral Ca
Sa	Sa	Sa	Sa	Sa	Sa	Sa	Sa	Sa	Sa	Sa	TD	TD	TD	TD	TD	TD	TD	TD	TD	TD	TD	TD
0	0	0	5	0	0	0	0	0	0	0	0	0	0	0	0	0	0	0	0	0	0	0
0	0	0	0	0	0	0	0	0	0	0	0	0	0	0	0	0	0	0	0	0	0	0
0	0	2	0	0	0	0	0	0	0	0	0	0	0	0	0	0	0	0	0	0	0	0
0	1	2	0	0	0	0	0	0	0	0	0	0	0	0	0	0	0	0	0	0	0	1
0	0	0	0	0	0	0	0	0	0	0	0	0	0	0	0	0	0	0	0	0	0	0
0	0	0	0	0	0	0	0	0	0	0	0	0	0	0	0	0	0	0	0	0	0	0
0	0	0	0	0	0	0	0	0	0	0	0	0	0	0	0	0	0	0	0	0	0	0
0	0	0	0	0	0	0	0	0	0	0	0	0	0	0	0	0	0	0	0	0	0	0
0	0	0	0	0	0	0	0	0	0	0	0	0	0	0	0	0	0	0	0	0	0	0
0	0	0	0	0	0	0	0	0	0	0	0	0	0	0	0	0	0	0	0	0	0	0
0	0	11	0	0	0	0	0	0	0	0	0	0	0	0	0	3	0	0	0	0	0	0
5	94	145	7	1	0	0	2	2	2	2	3	1	0	13	11	5	1	1	0	2	0	6
0	0	0	0	0	0	0	0	0	0	0	0	0	0	0	0	0	0	0	0	0	0	0
0	0	0	0	0	0	0	0	0	0	0	0	0	0	0	0	0	0	0	0	0	0	0
0	0	0	0	0	0	0	0	0	0	0	0	0	0	0	0	0	0	0	0	0	0	0
0	0	0	0	0	0	0	0	0	0	0	0	0	0	0	0	0	0	0	0	0	0	0
0	0	0	0	0	0	0	0	0	0	0	0	0	0	0	0	0	0	0	0	0	0	0
0	0	0	0	0	0	0	0	0	0	0	0	0	0	0	0	0	0	0	0	0	0	0
5	87	22	6	9	9	0	2	118	3	12	22	18	1	5	6	0	4	28	0	189	10	5
42	74	5	124	96	106	29	64	751	0	336	74	23	79	19	32	12	12	10	28	8	12	23
183	71	51	406	150	149	132	399	695	8	894	569	844	1026	893	422	36	24	587	253	840	375	203
0	0	0	0	0	0	0	0	0	0	0	0	0	0	0	0	0	0	0	0	0	0	0
0	0	0	0	0	0	0	0	0	2	0	0	0	0	0	0	0	0	0	0	0	0	0
0	2	1	0	0	0	0	0	0	0	26	0	0	0	1	8	0	0	0	0	0	0	0
0	0	0	0	0	0	0	0	0	0	0	0	0	0	0	0	0	0	0	0	0	0	0
0	1	9	0	0	0	0	0	1	0	0	0	0	0	0	0	1	0	0	0	0	0	0
0	0	0	0	0	0	0	0	0	0	0	0	0	0	0	0	0	0	0	0	0	0	0
0	2	8	0	9	6	0	0	0	0	0	2	0	0	3	0	0	0	0	0	0	0	0
0	9	0	6	8	4	1	2	32	0	50	43	4	1174	4	4776	52	1	0	175	0	83	5
0	0	0	0	0	0	0	0	0	0	0	0	0	0	0	0	0	0	0	0	0	0	0
0	0	0	0	0	0	0	0	0	0	0	0	0	0	0	0	0	0	0	0	0	0	0
0	0	0	0	0	0	0	0	0	0	0	0	0	0	0	2	0	0	0	0	0	0	0
0	0	2	0	0	0	0	0	0	0	0	0	0	0	0	0	1	0	0	0	0	0	0
4	20	72	1	0	0	0	0	1	0	0	0	0	0	3	5	6	0	0	0	0	0	1
0	1	1	0	0	0	0	0	0	0	0	0	0	0	0	0	0	0	0	0	0	0	0
0	0	5	0	0	0	0	0	0	0	0	0	0	0	0	0	0	0	0	0	0	0	0
1	0	0	0	0	0	0	0	0	0	0	0	0	0	0	0	0	0	0	0	0	0	0

C1.026	C1.027	C1.029	C1.030	C1.031	C1.032	C1.033	C1.034	C1.036	C1.037	C1.038	C2.002	C2.003	C2.006	C2.009	C2.010	C2.012	C2.013	C2.014	C2.016	C2.017	C2.018	C2.019
Oral Ca	Oral Ca	Oral Ca	Oral Ca	Oral Ca	Oral Ca	Oral Ca	Oral Ca	Oral Ca	Oral Ca	Oral Ca	Oral Ca	Oral Ca	Oral Ca	Oral Ca	Oral Ca	Oral Ca	Oral Ca	Oral Ca	Oral Ca	Oral Ca	Oral Ca	Oral Ca
Sa	Sa	Sa	Sa	Sa	Sa	Sa	Sa	Sa	Sa	Sa	TD	TD	TD	TD	TD	TD	TD	TD	TD	TD	TD	TD
0	0	0	0	0	0	0	0	0	0	0	0	0	0	0	0	0	0	0	0	0	0	0
0	0	0	0	0	0	0	0	0	0	0	0	0	0	0	0	0	0	0	0	0	0	0
0	0	0	0	0	0	0	0	0	0	0	0	0	0	0	0	0	0	0	0	0	0	0
0	0	0	0	0	0	0	0	0	0	0	0	0	0	0	0	0	0	0	0	0	0	0
0	1	1	0	0	0	0	0	0	0	0	0	0	0	0	0	0	0	0	0	0	0	0
0	1	3	0	0	0	0	0	0	0	0	0	0	0	0	0	1	0	0	0	0	0	0
0	0	0	0	0	0	0	0	0	0	0	0	0	0	0	0	0	0	0	0	0	0	0
0	0	0	0	0	0	0	0	0	0	0	0	0	0	0	0	0	0	0	0	0	0	0
0	0	0	0	0	0	0	0	0	0	0	0	0	0	0	0	0	0	0	0	0	0	0
0	0	0	0	0	0	0	0	0	0	0	0	0	0	0	0	0	0	0	0	0	0	0
0	0	0	0	0	0	0	0	0	0	0	0	0	0	0	0	0	0	0	0	0	0	0
1	9	24	0	1	0	0	0	1	0	0	0	0	0	3	1	3	0	0	0	0	0	0
0	0	0	0	0	0	0	0	0	0	0	0	0	0	0	0	0	0	0	0	0	0	0
0	0	2	0	0	0	0	0	0	0	0	0	0	0	0	0	0	0	0	0	0	0	0
0	0	4	0	0	0	0	0	0	0	0	0	0	0	0	0	0	0	0	0	0	0	0
0	0	0	0	0	0	0	0	0	0	0	0	0	0	0	0	0	0	0	0	0	0	0
0	0	0	0	0	0	0	0	0	0	0	0	0	0	0	0	0	0	0	0	0	0	0
0	0	0	0	0	1	0	0	0	2	0	0	0	0	0	0	0	1	0	0	0	0	0
70	289	1	120	187	121	169	143	212	87	177	22	16	6	23	31	0	33	2	11	10	48	3
0	2	0	0	0	0	0	0	1	3	0	0	0	0	0	0	0	0	0	0	0	0	0
16	122	0	12	87	52	46	34	30	34	26	0	2	0	0	0	0	3	0	1	1	0	0
1	0	0	0	0	0	0	0	0	2	0	0	0	0	0	0	0	0	0	0	0	0	0
0	0	0	0	0	0	0	2	0	0	0	0	0	0	0	0	0	0	0	0	0	0	0
0	3	0	4	3	2	0	2	0	0	0	0	0	0	17	0	0	0	0	0	5	0	0
5	9	3	0	1	5	0	0	0	3	0	2	3	2	2	7	1	9	2	1	0	2	0
0	0	0	0	0	0	0	0	0	0	0	0	0	0	0	4	3	0	0	0	0	0	0
0	0	0	0	0	0	0	0	0	0	0	0	0	0	0	0	0	0	0	0	0	0	0
0	0	0	0	0	0	0	0	0	0	0	0	0	0	0	0	0	0	0	0	0	0	0
4	0	0	0	0	3	0	6	2	0	3	3	6	6	0	7	7	6	0	11	9	2	59
13	49	7	75	70	74	123	100	23	46	33	1	0	0	0	180	62	1	0	0	8	3	1
0	0	0	0	0	0	0	0	0	0	0	0	0	0	0	0	0	0	0	0	0	0	0
0	0	0	0	0	0	0	0	0	0	0	0	0	0	0	0	0	0	0	0	0	0	0

C2.020	C2.021	C2.024	C2.025	C2.026	C2.027	C2.029	C2.030	C2.031	C2.032	C2.033	C2.034	C2.036	C2.037	C2.038	C3.002	C3.003	C3.006	C3.009	C3.010	C3.012	C3.013	C3.014
Oral Ca	Oral Ca	Oral Ca	Oral Ca	Oral Ca	Oral Ca	Oral Ca	Oral Ca	Oral Ca	Oral Ca	Oral Ca	Oral Ca	Oral Ca	Oral Ca	Oral Ca	Oral Ca	Oral Ca	Oral Ca	Oral Ca	Oral Ca	Oral Ca	Oral Ca	Oral Ca
TD	TD	TD	TD	TD	TD	TD	TD	TD	TD	TD	TD	TD	TD	TD	SP	SP	SP	SP	SP	SP	SP	SP
0	0	0	0	0	0	0	0	0	0	0	0	0	0	0	0	0	0	0	0	0	0	0
20961	25350	18240	13189	11315	11527	9523	24258	21518	26477	16752	26061	23209	23511	18712	20905	11291	22151	17428	5716	11834	20832	16683
1	1	143	131	66	19	22	5	0	0	2	10	2	0	0	0	0	14	25	39	38	116	49
0	0	0	0	0	0	0	0	0	0	0	0	0	0	0	0	0	0	0	0	0	0	0
0	0	0	0	0	0	0	0	0	0	0	0	0	0	0	0	0	0	0	0	1	0	0
501	2707	731	408	256	151	668	760	1167	381	718	794	289	3934	722	418	1031	303	1036	330	304	2451	1228
0	0	0	0	0	0	0	0	0	0	0	0	0	0	0	0	0	0	0	0	0	0	0
12454	7287	2699	3356	2721	4286	2067	6802	7822	4759	5190	5676	3379	5976	8128	10705	4547	9831	8239	1929	8082	7360	6868
0	0	0	0	0	0	0	0	0	0	0	0	0	0	0	0	0	0	0	0	0	0	0
0	0	0	0	0	0	0	0	0	0	0	0	0	0	0	0	0	0	0	0	0	0	0
0	0	0	0	0	0	0	0	0	0	0	0	0	0	0	0	0	0	0	0	0	0	0
0	0	0	0	0	0	0	0	0	0	0	1	0	0	0	0	0	0	0	0	0	0	0
0	2	0	0	0	0	0	0	0	0	0	0	0	0	0	4	4	12	0	0	1	0	0
0	1	0	0	0	0	0	0	0	0	0	0	0	4	0	21	0	0	0	0	0	15	0
0	0	0	0	0	0	0	0	0	0	0	0	0	0	0	0	0	0	0	0	0	0	0
6624	5722	2744	1649	2057	1063	4215	4773	6653	2280	4426	5428	2908	7668	4645	4980	716	3394	1530	706	728	6118	2085
645	280	1956	416	993	479	844	1298	1772	580	1243	2151	410	766	855	1095	574	1568	953	229	536	1321	1073
0	0	0	0	0	0	0	0	0	0	0	0	0	0	0	0	0	0	0	0	0	0	0
0	0	9	90	14	236	0	5	10	2	0	12	9	1	0	0	168	86	83	272	77	4	1
0	0	0	0	0	0	0	0	0	0	0	0	0	0	0	0	0	0	0	0	0	0	0
0	0	0	0	0	0	0	0	0	0	0	0	0	0	0	0	0	0	0	0	0	0	0
696	9283	6840	5812	4766	4786	1175	9987	3438	18253	4471	11521	15343	4387	3479	2388	4026	4899	4648	1704	2020	1169	3443
16	0	46	12	17	53	2	59	17	15	50	22	11	33	11	715	23	272	369	36	5	385	76
0	0	32	15	9	217	6	28	39	143	5	46	19	0	16	11	5	79	39	13	11	4	19
2	0	2	0	2	1	0	0	1	0	0	2	0	4	0	66	1	12	8	3	0	79	0
0	1	46	17	39	20	3	67	19	5	2	96	1	13	0	59	1	56	212	56	7	118	16
0	0	0	0	0	0	0	0	0	0	0	0	0	0	0	0	0	0	0	0	0	0	0
0	0	0	0	0	0	0	0	0	0	0	0	0	0	0	0	0	0	0	0	0	0	0
22	66	2992	1283	375	215	521	474	580	59	645	302	838	725	856	443	195	1625	286	399	24	1692	1825
0	0	0	0	0	0	0	0	0	0	0	0	0	0	0	0	0	0	0	0	0	0	0
0	0	0	0	0	0	0	0	0	0	0	0	0	0	0	0	0	0	0	0	0	0	0
0	0	0	0	0	1	0	0	0	0	0	0	0	0	0	0	0	0	0	0	0	0	0
0	0	0	0	0	0	0	0	0	0	0	0	0	0	0	0	0	0	0	0	0	0	0
0	0	0	0	0	0	0	0	0	0	0	0	0	0	0	0	0	0	0	0	0	0	0
0	0	0	0	0	0	0	0	0	0	0	0	0	0	0	0	0	0	0	0	0	0	0
0	0	0	0	0	0	0	0	0	0	0	0	0	0	0	0	0	0	0	0	0	0	0

C2.020	C2.021	C2.024	C2.025	C2.026	C2.027	C2.029	C2.030	C2.031	C2.032	C2.033	C2.034	C2.036	C2.037	C2.038	C3.002	C3.003	C3.006	C3.009	C3.010	C3.012	C3.013	C3.014
Oral Ca	Oral Ca	Oral Ca	Oral Ca	Oral Ca	Oral Ca	Oral Ca	Oral Ca	Oral Ca	Oral Ca	Oral Ca	Oral Ca	Oral Ca	Oral Ca	Oral Ca	Oral Ca	Oral Ca	Oral Ca	Oral Ca	Oral Ca	Oral Ca	Oral Ca	Oral Ca
TD	TD	TD	TD	TD	TD	TD	TD	TD	TD	TD	TD	TD	TD	TD	SP	SP	SP	SP	SP	SP	SP	SP
0	0	0	0	0	0	0	0	0	0	0	0	0	0	0	0	0	0	0	0	0	0	0
0	0	0	0	0	0	0	0	0	0	0	0	0	0	0	0	0	0	0	0	1	0	0
0	0	0	0	0	0	0	0	0	0	0	0	0	0	0	0	0	0	0	0	0	0	0
0	0	5	0	2	0	2	0	0	0	0	0	0	0	0	0	0	0	0	0	0	0	0
401	2583	702	403	239	148	630	737	1126	378	673	746	277	3724	676	397	1030	292	1034	329	281	2341	1225
100	124	22	5	15	3	33	23	41	3	45	48	12	210	46	21	1	11	2	1	23	110	3
0	0	0	0	0	0	0	0	0	0	0	0	0	0	0	0	0	0	0	0	0	0	0
0	0	0	0	0	0	0	0	0	0	0	0	0	0	0	0	0	0	0	0	0	0	0
0	0	2	0	0	0	3	0	0	0	0	0	0	0	0	0	0	0	0	0	0	0	0
0	0	0	0	0	0	0	0	0	0	0	0	0	0	0	0	0	0	0	0	0	0	0
0	0	0	0	0	0	0	0	0	0	0	0	0	0	0	0	0	0	0	0	0	0	0
0	0	2	0	0	3	7	0	0	1	0	0	0	0	0	0	0	0	0	2	1	0	0
12443	7101	2468	3190	2489	3889	2015	6626	7534	4521	5151	5558	3275	5900	8089	10256	999	5290	4283	581	800	5537	2337
0	0	2	0	1	0	1	0	0	0	0	0	0	0	0	0	0	0	0	0	0	0	0
11	186	182	166	224	394	25	176	288	237	39	118	104	76	38	449	3548	4541	3956	1346	7281	1823	4531
0	0	45	0	7	0	19	0	0	0	0	0	0	0	1	0	0	0	0	0	0	0	0
0	0	0	0	0	0	0	0	0	0	0	0	0	0	0	0	0	0	0	0	0	0	0
0	0	0	0	0	0	0	0	0	0	0	0	0	0	0	0	0	0	0	0	0	0	0
0	0	0	0	0	0	0	0	0	0	0	0	0	0	0	0	0	0	0	0	0	0	0
0	0	0	0	0	0	0	0	0	0	0	0	0	0	0	0	0	0	0	0	0	0	0
0	0	0	0	0	0	0	0	0	0	0	1	0	0	0	0	0	0	0	0	0	0	0
0	0	0	0	0	0	0	0	0	0	0	0	0	0	0	0	0	0	0	0	0	0	0
0	0	0	0	0	0	0	0	0	0	0	0	0	0	0	0	0	0	0	0	0	0	0
0	0	0	0	0	0	0	0	0	0	0	0	0	0	0	0	0	0	0	0	0	0	0
0	0	0	0	0	0	0	0	0	0	0	0	0	0	0	0	0	0	0	0	0	0	0
0	0	0	0	0	0	0	0	0	0	0	0	0	0	0	0	0	0	0	0	0	0	0
0	0	0	0	0	0	0	0	0	0	0	0	0	0	0	0	0	0	0	0	1	0	0
0	2	0	0	0	0	0	0	0	0	0	0	0	0	0	4	4	12	0	0	0	0	0
0	0	0	0	0	0	0	0	0	0	0	0	0	0	0	0	0	0	0	0	0	0	0
0	0	0	0	0	0	0	0	0	0	0	0	0	0	0	0	0	0	0	0	0	0	0
0	0	0	0	0	0	0	0	0	0	0	0	0	0	0	0	0	0	0	0	0	15	0
0	1	0	0	0	0	0	0	0	0	0	0	0	4	0	21	0	0	0	0	0	0	0
0	0	0	0	0	0	0	0	0	0	0	0	0	0	0	0	0	0	0	0	0	0	0
1219	2831	928	1183	1226	492	2801	2017	4247	1502	2195	2915	1954	3926	1226	197	340	1028	387	334	433	1318	409
5297	2704	1648	313	802	532	1224	2437	2284	715	1953	2269	717	3501	3179	4743	375	2363	1125	370	284	4717	1661
108	187	168	153	29	39	190	319	122	63	278	244	237	241	240	40	1	3	18	2	11	83	14
645	280	1956	416	993	479	844	1298	1772	580	1243	2151	410	766	855	1095	574	1568	953	229	536	1321	1073

C2.020 Oral Ca TD	C2.021 Oral Ca TD	C2.024 Oral Ca TD	C2.025 Oral Ca TD	C2.026 Oral Ca TD	C2.027 Oral Ca TD	C2.029 Oral Ca TD	C2.030 Oral Ca TD	C2.031 Oral Ca TD	C2.032 Oral Ca TD	C2.033 Oral Ca TD	C2.034 Oral Ca TD	C2.036 Oral Ca TD	C2.037 Oral Ca TD	C2.038 Oral Ca TD	C3.002 Oral Ca SP	C3.003 Oral Ca SP	C3.006 Oral Ca SP	C3.009 Oral Ca SP	C3.010 Oral Ca SP	C3.012 Oral Ca SP	C3.013 Oral Ca SP	C3.014 Oral Ca SP
0	0	0	0	0	0	0	0	0	0	0	0	0	0	0	0	0	0	0	0	0	0	0
0	0	9	90	14	236	0	5	10	2	0	12	9	1	0	0	168	86	83	272	77	4	1
0	0	0	0	0	0	0	0	0	0	0	0	0	0	0	0	0	0	0	0	0	0	0
0	0	0	0	0	0	0	0	0	0	0	0	0	0	0	0	0	0	0	0	0	0	0
0	0	0	0	0	0	0	0	0	0	0	0	0	0	0	0	0	0	0	0	0	0	0
0	0	0	0	0	0	0	0	0	0	0	0	0	0	0	0	0	0	0	0	0	0	0
62	69	758	119	460	88	77	53	26	70	0	66	116	72	49	32	111	103	3	30	131	114	4
70	7546	4626	4735	3050	3391	702	8845	2723	14626	3767	9486	12462	3312	2640	1214	2975	3288	3410	1269	1572	266	1686
1	0	3	1	1	3	1	0	1	0	1	3	1	2	0	88	1	51	13	5	1	147	1
211	213	1168	138	136	57	194	200	178	104	246	403	129	971	151	678	162	904	366	168	49	581	413
352	1455	285	819	1119	1247	201	889	510	3453	457	1563	2635	30	639	376	777	553	856	232	267	61	1337
0	0	0	0	0	0	0	0	0	0	0	0	0	0	0	0	0	0	0	0	0	0	0
0	0	0	0	0	0	0	0	0	0	0	0	0	0	0	0	0	2	0	0	0	0	0
16	0	46	12	17	53	2	59	17	15	50	22	11	33	11	715	23	270	369	36	5	385	76
2	0	2	0	2	1	0	0	1	0	0	2	0	4	0	66	1	12	8	3	0	79	0
0	0	0	0	0	0	0	0	0	0	0	0	0	0	0	0	0	0	0	0	0	0	0
0	1	46	16	36	20	2	67	18	5	2	96	1	13	0	45	1	54	190	17	7	14	16
0	0	0	1	3	0	1	0	1	0	0	0	0	0	0	14	0	2	22	39	0	104	0
0	0	0	0	0	0	0	0	0	0	0	0	0	0	0	0	0	0	0	0	0	0	0
0	0	0	0	0	0	0	0	0	0	0	0	0	0	0	0	0	0	0	0	0	0	0
0	0	0	0	0	0	0	0	0	0	0	0	0	0	0	0	0	0	0	0	0	0	0
0	0	0	0	0	0	0	0	0	0	0	0	0	0	0	0	0	0	0	0	0	0	0
0	0	0	0	0	0	0	0	0	0	0	0	0	0	0	0	0	0	0	0	0	0	0
22	66	2992	1283	375	215	521	474	580	59	645	302	838	725	856	443	195	1625	286	399	24	1692	1825
0	0	0	0	0	0	0	0	0	0	0	0	0	0	0	0	0	0	0	0	0	0	0
0	0	0	0	0	0	0	0	0	0	0	0	0	0	0	0	0	0	0	0	0	0	0
0	0	0	0	0	1	0	0	0	0	0	0	0	0	0	0	0	0	0	0	0	0	0
0	0	0	0	0	0	0	0	0	0	0	0	0	0	0	0	0	0	0	0	0	0	0
0	0	0	0	0	0	0	0	0	0	0	0	0	0	0	0	0	0	0	0	0	0	0
0	0	0	0	0	0	0	0	0	0	0	0	0	0	0	0	0	0	0	0	0	0	0
0	0	0	0	0	0	0	0	0	0	0	0	0	0	0	0	0	0	0	0	0	0	0
0	0	0	0	0	0	0	0	0	0	0	0	0	0	0	0	0	0	0	0	0	0	0
0	0	0	0	0	0	0	0	0	0	0	0	0	0	0	0	0	0	0	0	0	0	0
0	0	0	0	0	0	0	0	0	0	0	0	0	0	0	0	0	0	0	0	1	0	0
0	0	0	0	0	0	0	0	0	0	0	0	0	0	0	0	0	0	0	0	0	0	0
0	0	5	0	2	0	2	0	0	0	0	0	0	0	0	0	0	0	0	0	0	0	0
398	2582	499	398	125	129	594	737	1121	378	673	725	272	3695	675	397	1030	291	1014	232	125	1301	1225

C2.020	C2.021	C2.024	C2.025	C2.026	C2.027	C2.029	C2.030	C2.031	C2.032	C2.033	C2.034	C2.036	C2.037	C2.038	C3.002	C3.003	C3.006	C3.009	C3.010	C3.012	C3.013	C3.014
Oral Ca	Oral Ca	Oral Ca	Oral Ca	Oral Ca	Oral Ca	Oral Ca	Oral Ca	Oral Ca	Oral Ca	Oral Ca	Oral Ca	Oral Ca	Oral Ca	Oral Ca	Oral Ca	Oral Ca	Oral Ca	Oral Ca	Oral Ca	Oral Ca	Oral Ca	Oral Ca
TD	TD	TD	TD	TD	TD	TD	TD	TD	TD	TD	TD	TD	TD	TD	SP	SP	SP	SP	SP	SP	SP	SP
3	1	203	5	114	19	36	0	5	0	0	21	5	29	1	0	0	1	20	97	156	1040	0
0	0	0	0	0	0	0	0	0	0	0	0	0	0	0	0	0	0	0	0	0	0	0
100	124	22	5	15	3	33	23	41	3	45	48	12	210	46	21	1	11	2	1	23	110	3
0	0	0	0	0	0	0	0	0	0	0	0	0	0	0	0	0	0	0	0	0	0	0
0	0	0	0	0	0	0	0	0	0	0	0	0	0	0	0	0	0	0	0	0	0	0
0	0	0	0	0	0	0	0	0	0	0	0	0	0	0	0	0	0	0	0	0	0	0
0	0	2	0	0	0	3	0	0	0	0	0	0	0	0	0	0	0	0	0	0	0	0
0	0	0	0	0	0	0	0	0	0	0	0	0	0	0	0	0	0	0	0	0	0	0
0	0	0	0	0	0	0	0	0	0	0	0	0	0	0	0	0	0	0	0	0	0	0
0	0	2	0	0	3	7	0	0	1	0	0	0	0	0	0	0	0	0	2	1	0	0
12443	7101	2468	3190	2489	3889	2015	6626	7534	4521	5151	5558	3275	5900	8089	10256	999	5290	4283	581	800	5537	2337
0	0	2	0	1	0	1	0	0	0	0	0	0	0	0	0	0	0	0	0	0	0	0
11	186	182	166	224	394	25	176	288	237	39	118	104	76	38	449	3548	4541	3956	1346	7281	1823	4531
0	0	45	0	7	0	19	0	0	0	0	0	0	0	1	0	0	0	0	0	0	0	0
0	0	0	0	0	0	0	0	0	0	0	0	0	0	0	0	0	0	0	0	0	0	0
0	0	0	0	0	0	0	0	0	0	0	0	0	0	0	0	0	0	0	0	0	0	0
0	0	0	0	0	0	0	0	0	0	0	1	0	0	0	0	0	0	0	0	0	0	0
0	0	0	0	0	0	0	0	0	0	0	0	0	0	0	0	0	0	0	0	0	0	0
0	0	0	0	0	0	0	0	0	0	0	0	0	0	0	0	0	0	0	0	0	0	0
0	0	0	0	0	0	0	0	0	0	0	0	0	0	0	0	0	0	0	0	0	0	0
0	0	0	0	0	0	0	0	0	0	0	0	0	0	0	0	0	0	0	0	0	0	0
0	0	0	0	0	0	0	0	0	0	0	0	0	0	0	0	0	0	0	0	1	0	0
0	0	0	0	0	0	0	0	0	0	0	0	0	0	0	0	0	0	0	0	0	0	0
0	0	0	0	0	0	0	0	0	0	0	0	0	0	0	0	0	0	0	0	0	0	0
0	0	0	0	0	0	0	0	0	0	0	0	0	0	0	0	0	0	0	0	0	0	0
0	2	0	0	0	0	0	0	0	0	0	0	0	0	0	4	4	12	0	0	0	0	0
0	0	0	0	0	0	0	0	0	0	0	0	0	0	0	0	0	0	0	0	0	0	0
0	0	0	0	0	0	0	0	0	0	0	0	0	0	0	0	0	0	0	0	0	0	0
0	0	0	0	0	0	0	0	0	0	0	0	0	0	0	0	0	0	0	0	0	15	0
0	0	0	0	0	0	0	0	0	0	0	0	0	0	0	0	0	0	0	0	0	0	0
0	2	51	13	16	3	6	0	0	0	0	0	0	0	3	0	0	1	0	7	3	0	0
10	112	66	20	88	17	25	34	222	280	77	171	80	93	33	31	11	173	21	12	12	28	5
1209	2717	810	1150	1122	472	2769	1983	4025	1222	2118	2744	1874	3833	1190	166	329	854	365	315	418	1290	404
0	0	0	0	0	0	0	0	0	0	0	0	0	0	0	0	0	0	1	0	0	0	0
5297	2704	1648	313	802	532	1224	2437	2284	715	1953	2269	717	3501	3179	4743	375	2363	1125	370	284	4717	1661
0	0	0	0	0	0	0	0	0	0	0	0	0	0	0	0	0	0	0	0	0	0	0
0	0	0	0	0	0	0	0	0	0	0	0	0	0	0	0	0	0	0	0	0	0	0

C2.020	C2.021	C2.024	C2.025	C2.026	C2.027	C2.029	C2.030	C2.031	C2.032	C2.033	C2.034	C2.036	C2.037	C2.038	C3.002	C3.003	C3.006	C3.009	C3.010	C3.012	C3.013	C3.014
Oral Ca	Oral Ca	Oral Ca	Oral Ca	Oral Ca	Oral Ca	Oral Ca	Oral Ca	Oral Ca	Oral Ca	Oral Ca	Oral Ca	Oral Ca	Oral Ca	Oral Ca	Oral Ca	Oral Ca	Oral Ca	Oral Ca	Oral Ca	Oral Ca	Oral Ca	Oral Ca
TD	TD	TD	TD	TD	TD	TD	TD	TD	TD	TD	TD	TD	TD	TD	SP	SP	SP	SP	SP	SP	SP	SP
108	187	168	153	29	39	190	319	122	63	278	244	237	241	240	40	1	3	18	2	11	83	14
645	280	1956	416	993	479	844	1298	1772	580	1243	2151	410	766	855	1095	574	1568	953	229	536	1321	1073
0	0	0	0	0	0	0	0	0	0	0	0	0	0	0	0	0	0	0	0	0	0	0
0	0	0	0	0	0	0	0	0	0	0	0	0	0	0	0	0	0	0	0	0	0	0
0	0	0	0	0	0	0	0	0	0	0	0	0	0	0	0	0	0	0	0	0	0	0
0	0	0	0	0	0	0	0	0	0	0	0	0	0	0	0	0	0	0	0	0	0	0
0	0	0	0	0	0	0	0	0	0	0	0	0	0	0	0	0	0	0	0	0	0	0
0	0	0	0	0	0	1	0	0	0	0	0	0	0	0	0	0	0	0	0	0	0	0
61	67	13	3	4	6	10	52	25	68	0	65	116	71	49	31	111	94	0	15	15	113	3
0	0	0	0	0	0	0	0	0	0	0	0	0	0	0	0	0	0	0	0	0	0	0
0	0	0	0	0	1	0	0	0	0	0	0	0	0	0	0	0	0	0	0	0	0	0
1	1	69	3	35	7	47	1	0	1	0	0	0	1	0	1	0	2	1	5	14	0	1
0	0	12	0	0	0	5	0	0	0	0	0	0	0	0	0	0	0	0	1	0	0	0
0	0	3	112	0	2	1	0	0	0	0	0	0	0	0	0	0	0	1	0	2	0	0
0	0	0	0	0	0	0	0	0	0	0	0	0	0	0	0	0	0	0	0	0	0	0
0	1	661	1	421	72	13	0	1	1	0	0	0	0	0	0	0	1	1	9	100	1	0
0	0	0	0	0	0	0	0	0	0	0	0	0	0	0	0	0	0	0	0	0	0	0
25	70	102	51	92	54	45	16	25	3	10	27	14	18	13	151	543	1596	203	799	297	15	385
0	0	0	0	0	0	0	0	0	0	0	0	0	0	0	0	0	0	0	0	0	0	0
0	0	0	0	0	0	0	0	0	0	0	0	0	0	0	0	0	0	0	0	0	0	0
45	7476	4519	4678	2958	3337	657	8829	2698	14623	3756	9459	12448	3294	2627	1063	2429	1691	3205	469	1274	250	1301
0	0	1	0	0	0	0	0	0	0	1	0	0	0	0	0	3	1	2	1	0	1	0
0	0	1	0	0	0	0	0	0	0	0	0	0	0	0	0	0	0	0	0	0	0	0
0	0	0	0	0	0	0	0	0	0	0	0	0	0	0	0	0	0	0	0	0	0	0
0	0	0	0	0	0	0	0	0	0	0	0	0	0	0	0	0	0	0	0	0	0	0
1	0	0	0	0	0	0	0	0	0	1	3	0	2	0	88	0	0	8	0	0	6	1
0	0	0	1	0	3	0	0	1	0	0	0	1	0	0	0	1	0	4	5	1	141	0
0	0	0	0	0	0	0	0	0	0	0	0	0	0	0	0	0	0	0	0	0	0	0
0	0	1	0	1	0	1	0	0	0	0	0	0	0	0	0	0	50	1	0	0	0	0
0	0	0	0	0	0	0	0	0	0	0	0	0	0	0	0	0	0	0	0	0	0	0
0	0	2	0	0	0	0	0	0	0	0	0	0	0	0	0	0	1	0	0	0	0	0
0	0	0	0	0	0	0	0	0	0	0	0	0	0	0	0	0	0	0	0	0	0	0
0	0	0	0	0	0	0	0	0	0	0	0	0	0	0	0	0	0	0	0	0	0	0
211	213	1168	138	136	57	194	200	178	104	246	403	129	971	151	678	162	904	366	168	49	581	413
0	0	0	0	5	2	3	0	0	0	0	0	0	0	3	0	0	0	5	0	0	0	0
0	0	0	0	0	0	0	0	0	0	0	0	0	0	0	0	0	0	0	0	0	0	0
2	6	13	22	13	2	3	5	0	2	3	5	0	2	2	5	194	152	214	48	32	23	827

C2.020 Oral Ca TD	C2.021 Oral Ca TD	C2.024 Oral Ca TD	C2.025 Oral Ca TD	C2.026 Oral Ca TD	C2.027 Oral Ca TD	C2.029 Oral Ca TD	C2.030 Oral Ca TD	C2.031 Oral Ca TD	C2.032 Oral Ca TD	C2.033 Oral Ca TD	C2.034 Oral Ca TD	C2.036 Oral Ca TD	C2.037 Oral Ca TD	C2.038 Oral Ca TD	C3.002 Oral Ca SP	C3.003 Oral Ca SP	C3.006 Oral Ca SP	C3.009 Oral Ca SP	C3.010 Oral Ca SP	C3.012 Oral Ca SP	C3.013 Oral Ca SP	C3.014 Oral Ca SP
9	2	81	378	27	30	22	3	0	7	0	1	0	6	3	0	0	0	18	23	49	0	8
0	0	1	0	0	0	0	0	0	0	0	1	0	0	0	0	0	0	0	0	0	0	0
0	0	0	0	0	0	0	0	0	0	0	0	0	0	0	0	0	0	0	0	0	0	0
328	1447	156	381	1042	916	166	872	499	3362	453	1544	2526	22	559	353	278	398	583	90	145	37	502
13	0	31	38	27	294	6	9	9	81	1	11	109	0	62	18	305	3	36	63	35	1	0
0	0	0	0	0	0	0	0	0	0	0	0	0	0	0	0	0	0	0	0	0	0	0
0	0	1	0	0	0	0	0	0	0	0	0	0	0	0	0	0	0	0	0	0	0	0
0	0	2	0	5	3	1	0	2	1	0	1	0	0	10	0	0	0	0	8	6	0	0
0	0	0	0	0	0	0	0	0	0	0	0	0	0	0	0	0	0	0	0	0	0	0
0	0	0	0	0	0	0	0	0	0	0	0	0	0	0	0	0	2	0	0	0	0	0
0	0	0	0	0	0	0	0	0	0	0	0	0	0	0	2	0	0	0	0	0	5	0
16	0	46	12	17	53	2	59	17	15	50	22	11	33	11	713	23	267	369	36	5	380	76
2	0	2	0	2	1	0	0	1	0	0	2	0	4	0	66	1	12	8	3	0	79	0
0	0	0	4	0	1	0	1	8	0	0	2	0	6	0	0	0	6	187	1	0	0	0
0	1	20	8	26	7	2	1	2	4	2	3	1	7	0	45	1	1	3	15	5	14	6
0	0	26	4	10	12	0	65	8	1	0	91	0	0	0	0	0	47	0	1	2	0	10
0	0	0	1	3	0	1	0	1	0	0	0	0	0	0	14	0	2	22	39	0	104	0
0	0	0	0	0	0	0	0	0	0	0	0	0	0	0	0	0	0	0	0	0	0	0
0	0	0	0	0	0	0	0	0	0	0	0	0	0	0	0	0	0	0	0	0	0	0
15	52	15	140	41	73	1	5	12	8	13	53	114	6	34	8	8	59	1	9	1	26	1
0	0	0	0	0	0	0	0	0	0	0	0	0	0	0	0	0	0	0	0	0	0	0
2	1	4	6	5	2	6	5	0	5	3	2	0	6	4	101	0	13	2	2	1	157	256
0	0	0	0	0	0	0	0	0	0	0	0	0	0	0	0	0	0	0	0	0	0	0
0	0	0	0	0	0	0	0	0	0	0	0	0	0	0	0	0	0	0	0	0	0	0
0	0	0	0	0	1	0	0	0	0	0	0	0	0	0	0	0	0	0	0	0	0	0
0	0	0	0	0	0	0	0	0	0	0	0	0	0	0	0	0	0	0	0	0	0	0
0	0	0	0	0	0	0	0	0	0	0	0	0	0	0	0	0	0	0	0	0	0	0
0	0	0	0	0	0	0	0	0	0	0	0	0	0	0	0	0	0	0	0	0	0	0
0	0	0	0	0	0	0	0	0	0	0	0	0	0	0	0	0	0	0	0	0	0	0
0	0	0	0	0	0	0	0	0	0	0	0	0	0	0	0	0	0	0	0	0	0	0
0	0	0	0	0	0	0	0	0	0	0	0	0	0	0	0	0	0	0	0	0	0	0
0	0	0	0	0	0	0	0	0	0	0	0	0	0	0	0	0	0	0	0	0	0	0
0	0	3	0	1	0	2	0	0	0	0	0	0	0	0	0	0	0	0	0	0	0	0
0	0	0	0	0	0	0	0	0	0	0	0	0	0	0	0	0	0	0	0	0	0	0
178	2055	403	373	81	107	406	419	807	101	390	423	187	2424	453	200	253	249	345	58	40	766	452
0	0	0	0	0	0	0	0	0	0	0	0	0	0	0	0	0	0	0	0	0	0	0
0	0	0	0	0	0	0	0	0	0	0	0	0	0	0	0	0	0	0	0	0	0	0

C2.020	C2.021	C2.024	C2.025	C2.026	C2.027	C2.029	C2.030	C2.031	C2.032	C2.033	C2.034	C2.036	C2.037	C2.038	C3.002	C3.003	C3.006	C3.009	C3.010	C3.012	C3.013	C3.014
Oral Ca	Oral Ca	Oral Ca	Oral Ca	Oral Ca	Oral Ca	Oral Ca	Oral Ca	Oral Ca	Oral Ca	Oral Ca	Oral Ca	Oral Ca	Oral Ca	Oral Ca	Oral Ca	Oral Ca	Oral Ca	Oral Ca	Oral Ca	Oral Ca	Oral Ca	Oral Ca
TD	TD	TD	TD	TD	TD	TD	TD	TD	TD	TD	TD	TD	TD	TD	SP	SP	SP	SP	SP	SP	SP	SP
0	6	3	2	8	4	1	0	1	2	1	2	4	1	2	94	554	15	425	85	65	474	416
0	0	0	0	0	0	0	0	0	0	0	0	0	0	0	0	0	0	0	0	0	0	0
0	0	0	0	0	0	0	0	0	0	0	0	0	0	0	0	0	0	0	0	0	0	0
0	0	0	0	0	0	0	0	0	0	0	0	0	0	0	0	0	0	0	0	0	0	0
0	0	0	0	0	0	0	0	0	0	0	0	0	0	0	0	0	0	0	0	0	0	0
0	0	0	0	0	0	0	0	0	0	0	0	0	0	0	0	0	0	0	0	0	0	0
0	0	0	0	0	0	0	0	0	0	0	0	0	0	0	0	0	0	0	0	0	0	0
0	0	0	0	0	0	0	0	0	0	0	0	0	0	0	0	0	0	0	0	0	0	0
0	0	0	0	0	0	0	0	0	0	0	0	0	0	0	0	0	0	0	0	0	0	0
0	0	0	0	0	0	0	0	0	0	0	0	0	0	0	0	0	0	0	0	0	0	0
0	0	0	0	0	0	0	0	0	0	0	0	0	0	0	0	0	0	0	0	0	0	0
0	0	4	0	0	0	1	0	0	0	0	0	0	0	0	0	0	0	0	0	0	0	0
220	516	80	22	30	17	178	315	310	274	280	298	80	1269	220	79	33	1	19	1	0	21	10
0	0	1	0	0	0	1	0	0	0	0	0	0	0	0	0	0	0	0	0	0	0	0
0	0	0	0	0	0	2	0	0	0	0	0	0	0	0	0	0	0	0	1	0	0	0
0	0	1	0	0	0	0	0	0	0	0	0	0	0	0	0	0	0	0	0	0	0	0
0	0	3	0	3	0	3	0	0	0	0	0	0	0	0	0	0	0	9	0	0	0	0
0	0	0	0	0	0	0	0	0	0	0	0	0	0	0	0	0	0	0	0	0	0	0
0	0	0	0	0	0	0	0	0	0	0	0	0	0	0	0	0	0	0	0	0	0	0
0	5	2	1	2	1	0	3	3	1	2	2	1	1	0	24	190	26	216	86	20	40	347
0	0	1	0	0	0	2	0	0	0	0	0	0	0	0	0	0	0	0	0	0	0	0
0	0	0	0	0	0	0	0	0	0	0	0	0	0	0	0	0	0	0	0	0	0	0
0	0	0	0	0	0	0	0	0	0	0	0	0	0	0	0	0	0	0	0	0	0	0
0	0	0	0	0	0	0	0	0	0	0	0	0	0	0	0	0	0	0	0	0	0	0
0	0	0	0	0	0	0	0	0	0	0	0	0	0	0	0	0	0	0	0	0	0	0
0	0	0	0	0	0	0	0	0	0	0	0	0	0	0	0	0	0	0	0	0	0	0
3	1	203	5	114	19	36	0	5	0	0	21	5	29	1	0	0	1	20	97	156	1040	0
100	124	22	5	15	3	33	23	41	3	45	48	12	210	46	21	1	11	2	1	23	110	3
0	0	0	0	0	0	0	0	0	0	0	0	0	0	0	0	0	0	0	0	0	0	0
0	0	0	0	0	0	0	0	0	0	0	0	0	0	0	0	0	0	0	0	0	0	0
0	0	0	0	0	0	0	0	0	0	0	0	0	0	0	0	0	0	0	0	0	0	0
0	0	1	0	0	0	1	0	0	0	0	0	0	0	0	0	0	0	0	0	0	0	0
0	0	0	0	0	0	0	0	0	0	0	0	0	0	0	0	0	0	0	0	0	0	0
0	0	0	0	0	0	0	0	0	0	0	0	0	0	0	0	0	0	0	0	0	0	0
0	0	2	0	0	3	7	0	0	1	0	0	0	0	0	0	0	0	0	2	1	0	0
0	0	0	0	0	0	0	0	0	0	0	0	0	0	0	0	0	0	0	0	0	0	0
0	0	0	0	0	0	0	0	0	0	0	0	0	0	0	0	0	0	0	0	0	0	0

C2.020	C2.021	C2.024	C2.025	C2.026	C2.027	C2.029	C2.030	C2.031	C2.032	C2.033	C2.034	C2.036	C2.037	C2.038	C3.002	C3.003	C3.006	C3.009	C3.010	C3.012	C3.013	C3.014
Oral Ca	Oral Ca	Oral Ca	Oral Ca	Oral Ca	Oral Ca	Oral Ca	Oral Ca	Oral Ca	Oral Ca	Oral Ca	Oral Ca	Oral Ca	Oral Ca	Oral Ca	Oral Ca	Oral Ca	Oral Ca	Oral Ca	Oral Ca	Oral Ca	Oral Ca	Oral Ca
TD	TD	TD	TD	TD	TD	TD	TD	TD	TD	TD	TD	TD	TD	TD	SP	SP	SP	SP	SP	SP	SP	SP
0	0	0	0	0	0	0	0	0	0	0	3	0	0	0	0	0	0	3	0	0	0	0
1598	1366	471	726	443	1951	584	2278	890	1177	2056	1124	762	1312	2409	512	6	570	904	16	71	127	57
2	1	0	3	0	5	0	0	0	4	0	3	1	5	2	4	0	6	24	4	4	13	0
0	0	1	0	0	6	0	0	0	0	0	10	0	0	0	0	0	0	0	0	0	8	0
0	0	0	0	1	3	2	0	0	0	0	0	0	0	0	0	0	0	2	0	0	0	0
35	739	500	295	1333	1083	119	1094	3725	2321	197	1419	1624	140	233	1405	474	840	674	333	188	259	253
10808	4995	1495	2160	703	812	1310	3249	2910	1017	2895	2990	877	4438	5443	7983	519	3781	2581	217	534	4825	1908
0	0	0	0	0	0	0	0	0	0	0	0	0	0	0	0	0	0	0	0	0	2	0
0	0	0	0	0	0	0	0	0	0	0	0	0	0	0	0	0	0	11	0	0	0	0
0	0	0	0	1	4	0	0	0	0	0	0	2	0	0	0	0	0	6	1	3	0	0
0	0	0	0	0	0	0	0	0	0	0	0	0	0	0	0	0	0	0	0	0	0	0
0	0	2	0	1	0	1	0	0	0	0	0	0	0	0	0	0	0	0	0	0	0	0
0	82	14	20	64	22	7	25	23	32	4	12	29	26	10	11	164	1410	70	628	17	24	104
0	0	0	0	0	0	0	0	0	0	0	0	0	0	0	0	0	0	0	0	0	0	0
11	104	168	146	160	372	18	151	265	205	35	106	75	50	28	438	3384	3131	3885	718	7264	1799	4427
0	0	45	0	7	0	19	0	0	0	0	0	0	0	1	0	0	0	0	0	0	0	0
0	0	0	0	0	0	0	0	0	0	0	0	0	0	0	0	0	0	0	0	0	0	0
0	0	0	0	0	0	0	0	0	0	0	0	0	0	0	0	0	0	0	0	0	0	0
0	0	0	0	0	0	0	0	0	0	0	1	0	0	0	0	0	0	0	0	0	0	0
0	0	0	0	0	0	0	0	0	0	0	0	0	0	0	0	0	0	0	0	0	0	0
0	0	0	0	0	0	0	0	0	0	0	0	0	0	0	0	0	0	0	0	0	0	0
0	0	0	0	0	0	0	0	0	0	0	0	0	0	0	0	0	0	0	0	0	0	0
0	0	0	0	0	0	0	0	0	0	0	0	0	0	0	0	0	0	0	0	0	0	0
0	0	0	0	0	0	0	0	0	0	0	0	0	0	0	0	0	0	0	0	0	0	0
0	0	3	0	1	0	1	0	0	0	0	0	0	0	0	0	0	0	0	0	0	0	0
0	0	0	0	0	0	0	0	0	0	0	0	0	0	0	0	0	1	0	0	0	0	0
0	0	0	0	0	0	0	0	0	0	0	0	0	0	0	0	0	0	0	0	0	0	0
0	0	45	11	13	3	4	0	0	0	0	0	0	0	3	0	0	0	0	7	3	0	0
0	0	1	0	0	0	1	0	0	0	0	0	0	0	0	0	0	0	0	0	0	0	0
0	0	0	0	0	0	0	0	0	0	0	0	0	0	0	0	0	0	0	0	0	0	0
0	0	1	0	1	0	0	0	0	0	0	0	0	0	0	0	0	0	0	0	0	0	0
0	0	0	0	0	0	0	0	0	0	0	0	0	0	0	0	0	0	0	0	0	0	0
0	2	1	2	1	0	0	0	0	0	0	0	0	0	0	0	0	0	0	0	0	0	0
0	0	0	0	0	0	0	0	0	0	0	0	0	0	0	0	0	0	0	0	0	0	0
10	112	66	20	88	17	25	34	222	280	77	171	80	93	33	31	11	173	21	12	12	28	5
0	8	6	4	52	15	11	5	34	4	2	16	12	9	5	0	16	18	23	29	92	0	36
316	842	89	121	163	123	443	1080	1432	560	840	828	773	204	314	10	9	4	15	10	12	22	40

C2.020	C2.021	C2.024	C2.025	C2.026	C2.027	C2.029	C2.030	C2.031	C2.032	C2.033	C2.034	C2.036	C2.037	C2.038	C3.002	C3.003	C3.006	C3.009	C3.010	C3.012	C3.013	C3.014
Oral Ca	Oral Ca	Oral Ca	Oral Ca	Oral Ca	Oral Ca	Oral Ca	Oral Ca	Oral Ca	Oral Ca	Oral Ca	Oral Ca	Oral Ca	Oral Ca	Oral Ca	Oral Ca	Oral Ca	Oral Ca	Oral Ca	Oral Ca	Oral Ca	Oral Ca	Oral Ca
TD	TD	TD	TD	TD	TD	TD	TD	TD	TD	TD	TD	TD	TD	TD	SP	SP	SP	SP	SP	SP	SP	SP
0	0	1	0	0	0	0	0	0	0	0	0	0	0	2	0	0	0	0	0	1	0	1
0	0	131	25	428	111	416	0	4	0	2	0	2	6	0	0	0	0	8	201	145	178	0
0	0	0	0	0	0	0	0	0	0	0	0	7	0	0	0	0	0	0	0	0	0	0
891	1862	579	993	475	217	1896	896	2552	658	1273	1900	1080	3613	869	156	303	832	319	75	168	1090	325
0	0	0	0	0	0	0	0	0	0	0	0	0	0	0	0	0	0	1	0	0	0	0
0	0	0	0	0	0	0	0	0	0	0	0	0	0	0	0	0	0	0	0	0	0	0
93	154	390	78	55	84	141	222	280	70	261	292	48	252	129	515	6	37	61	22	3	275	68
0	21	265	18	212	31	18	13	54	73	5	29	1	19	2	59	1	69	4	87	17	16	1
0	0	0	0	1	0	0	0	0	0	0	0	0	0	0	0	0	0	0	0	0	4	0
0	0	25	1	12	2	5	0	0	0	0	0	0	0	0	0	1	0	1	2	37	0	0
0	0	0	0	0	0	0	0	0	0	0	0	0	0	0	0	0	0	0	0	0	0	0
0	0	0	1	0	0	0	0	5	1	0	2	1	3	0	1	0	0	3	0	1	677	2
2356	500	284	56	181	115	297	396	600	117	640	524	266	770	445	706	143	259	484	52	112	895	276
0	0	87	3	28	8	9	5	76	6	8	15	11	0	18	5	1	14	7	0	1	12	2
4	67	117	29	125	69	32	68	236	54	73	126	60	101	72	368	72	220	45	128	17	57	53
0	0	2	7	15	15	11	0	1	0	0	0	2	0	0	0	0	0	81	0	11	0	0
0	0	0	0	0	0	0	0	0	0	0	1	0	0	0	19	0	2	0	0	0	16	0
2720	1889	399	92	137	186	656	1620	869	366	907	1210	303	2183	2353	2910	137	1456	352	28	79	2416	1201
0	0	0	0	0	0	0	0	0	0	0	0	0	0	0	0	0	0	0	0	0	0	0
108	187	168	153	29	39	190	319	122	63	278	244	237	241	240	40	1	3	18	2	11	83	14
158	108	1693	352	870	443	674	973	1510	525	506	1823	321	100	489	837	344	1148	511	139	423	857	638
487	172	263	64	123	36	170	325	262	55	737	328	89	666	366	258	230	420	442	90	113	464	435
0	0	0	0	0	0	0	0	0	0	0	0	0	0	0	0	0	0	0	0	0	0	0
0	0	0	0	0	0	0	0	0	0	0	0	0	0	0	0	0	0	0	0	0	0	0
61	67	13	3	4	6	10	52	25	68	0	65	116	71	49	31	111	94	0	15	15	113	3
0	0	0	0	0	0	1	0	0	0	0	0	0	0	0	0	0	0	0	0	0	0	0
0	0	0	0	0	0	0	0	0	0	0	0	0	0	0	0	0	0	0	0	0	0	0
0	0	1	0	0	0	0	0	0	0	0	0	0	0	0	0	0	0	0	1	1	0	0
1	1	11	0	3	0	10	1	0	1	0	0	0	1	0	1	0	2	0	0	2	0	0
0	0	0	3	7	4	1	0	0	0	0	0	0	0	0	0	0	0	1	4	5	0	0
0	0	28	0	4	0	10	0	0	0	0	0	0	0	0	0	0	0	0	0	1	0	0
0	0	2	0	0	1	1	0	0	0	0	0	0	0	0	0	0	0	0	0	4	0	0
0	0	0	0	0	0	1	0	0	0	0	0	0	0	0	0	0	0	0	0	0	0	0
0	0	11	0	10	1	6	0	0	0	0	0	0	0	0	0	0	0	0	0	0	0	1
0	0	8	0	8	0	9	0	0	0	0	0	0	0	0	0	0	0	0	0	0	0	0
0	0	0	0	0	0	0	0	0	0	0	0	0	0	0	0	0	0	0	0	0	0	0
0	0	0	0	0	0	0	0	0	0	0	0	0	0	0	0	0	0	0	0	0	0	0

C2.020	C2.021	C2.024	C2.025	C2.026	C2.027	C2.029	C2.030	C2.031	C2.032	C2.033	C2.034	C2.036	C2.037	C2.038	C3.002	C3.003	C3.006	C3.009	C3.010	C3.012	C3.013	C3.014
Oral Ca	Oral Ca	Oral Ca	Oral Ca	Oral Ca	Oral Ca	Oral Ca	Oral Ca	Oral Ca	Oral Ca	Oral Ca	Oral Ca	Oral Ca	Oral Ca	Oral Ca	Oral Ca	Oral Ca	Oral Ca	Oral Ca	Oral Ca	Oral Ca	Oral Ca	Oral Ca
TD	TD	TD	TD	TD	TD	TD	TD	TD	TD	TD	TD	TD	TD	TD	SP	SP	SP	SP	SP	SP	SP	SP
0	0	12	0	0	0	5	0	0	0	0	0	0	0	0	0	0	0	0	1	0	0	0
0	0	0	112	0	2	1	0	0	0	0	0	0	0	0	0	0	0	0	0	2	0	0
0	0	3	0	0	0	0	0	0	0	0	0	0	0	0	0	0	0	1	0	0	0	0
0	0	0	0	0	0	0	0	0	0	0	0	0	0	0	0	0	0	0	0	0	0	0
0	0	0	0	0	0	0	0	0	0	0	0	0	0	0	0	0	0	0	0	0	0	0
0	0	0	0	0	0	0	0	0	0	0	0	0	0	0	0	0	0	0	0	0	0	0
0	0	0	0	0	0	0	0	0	0	0	0	0	0	0	0	0	0	0	0	0	0	0
0	1	661	1	419	72	13	0	1	1	0	0	0	0	0	0	0	1	1	9	100	1	0
0	0	4	1	3	1	1	0	0	0	0	0	0	0	0	0	0	0	6	0	0	0	0
25	36	36	39	24	11	24	7	4	1	7	20	9	13	9	74	284	5	123	136	26	1	320
0	34	55	10	59	41	16	9	21	2	3	7	5	5	3	77	259	1590	73	662	270	14	65
0	0	7	0	6	0	4	0	0	0	0	0	0	0	1	0	0	1	1	0	1	0	0
0	0	0	0	0	0	0	0	0	0	0	0	0	0	0	0	0	0	0	0	0	0	0
45	7476	4519	4678	2958	3337	657	8829	2698	14623	3756	9459	12448	3294	2627	1063	2429	1691	3205	469	1274	250	1301
0	0	1	0	0	0	0	0	0	0	1	0	0	0	0	0	3	1	2	1	0	1	0
0	0	0	0	0	0	0	0	0	0	0	0	0	0	0	0	0	0	0	0	0	0	0
0	0	0	0	0	0	0	0	0	0	0	0	0	0	0	0	0	0	0	0	0	0	0
1	0	0	0	0	0	0	0	0	0	1	3	0	2	0	88	0	0	8	0	0	6	1
0	0	0	0	0	0	0	0	1	0	0	0	0	0	0	0	0	0	0	0	0	0	0
0	0	0	1	0	3	0	0	0	0	0	0	1	0	0	0	1	0	4	5	1	141	0
0	0	0	0	0	0	0	0	0	0	0	0	0	0	0	0	0	0	0	0	0	0	0
0	0	2	0	0	0	0	0	0	0	0	0	0	0	0	0	0	0	0	0	0	0	0
0	0	0	0	0	0	0	0	0	0	0	0	0	0	0	0	0	0	0	0	0	0	0
0	0	0	0	0	0	0	0	0	0	0	0	0	0	0	0	0	0	0	0	0	0	0
211	213	1168	138	136	56	193	199	178	99	246	400	128	971	151	657	162	904	363	168	46	581	412
0	0	0	0	0	1	1	0	0	5	0	3	1	0	0	21	0	0	3	0	3	0	1
0	0	0	0	0	0	0	0	0	0	0	0	0	0	3	0	0	0	0	0	0	0	0
0	0	0	0	5	2	3	0	0	0	0	0	0	0	0	0	0	0	5	0	0	0	0
0	0	0	0	0	0	0	0	0	0	0	0	0	0	0	0	0	0	0	0	0	0	0
0	0	0	0	0	0	0	0	0	0	0	0	0	0	0	0	0	0	0	0	0	0	0
0	0	0	0	0	0	0	0	0	0	0	0	0	0	0	0	0	0	0	0	0	0	0
2	4	11	19	12	2	3	5	0	2	3	5	0	2	2	5	194	152	202	48	32	23	827
9	2	81	378	27	30	22	3	0	7	0	1	0	6	3	0	0	0	18	23	49	0	8
0	0	0	0	0	0	0	0	0	0	0	0	0	0	0	0	0	0	0	0	0	0	0
0	0	1	0	0	0	0	0	0	0	0	1	0	0	0	0	0	0	0	0	0	0	0
0	0	0	0	0	0	0	0	0	0	0	0	0	0	0	0	0	0	0	0	0	0	0
0	0	0	0	0	0	0	0	0	0	0	0	0	0	0	0	0	0	0	0	0	0	0

C2.020	C2.021	C2.024	C2.025	C2.026	C2.027	C2.029	C2.030	C2.031	C2.032	C2.033	C2.034	C2.036	C2.037	C2.038	C3.002	C3.003	C3.006	C3.009	C3.010	C3.012	C3.013	C3.014
Oral Ca	Oral Ca	Oral Ca	Oral Ca	Oral Ca	Oral Ca	Oral Ca	Oral Ca	Oral Ca	Oral Ca	Oral Ca	Oral Ca	Oral Ca	Oral Ca	Oral Ca	Oral Ca	Oral Ca	Oral Ca	Oral Ca	Oral Ca	Oral Ca	Oral Ca	Oral Ca
TD	TD	TD	TD	TD	TD	TD	TD	TD	TD	TD	TD	TD	TD	TD	SP	SP	SP	SP	SP	SP	SP	SP
328	1447	156	381	1042	916	166	872	499	3362	453	1544	2526	22	559	353	278	398	583	90	145	37	502
13	0	14	37	21	276	3	9	7	81	1	11	109	0	61	18	305	3	30	53	8	1	0
0	0	17	1	6	18	3	0	2	0	0	0	0	0	1	0	0	0	6	10	27	0	0
0	0	0	0	0	0	0	0	0	0	0	0	0	0	0	0	0	0	0	0	0	0	0
0	0	0	0	0	0	0	0	0	0	0	0	0	0	0	0	0	0	0	0	0	0	0
0	0	1	0	0	0	0	0	0	0	0	0	0	0	0	0	0	0	0	0	0	0	0
0	0	0	0	1	0	1	0	0	0	0	0	0	0	0	0	0	0	0	0	0	0	0
0	0	2	0	4	3	0	0	2	1	0	1	0	0	10	0	0	0	0	8	6	0	0
0	0	0	0	0	0	0	0	0	0	0	0	0	0	0	2	0	0	0	0	0	5	0
16	0	46	12	17	53	2	59	17	15	50	22	11	33	11	713	23	267	369	36	5	380	76
1	0	2	0	2	1	0	0	1	0	0	2	0	4	0	66	1	12	8	3	0	79	0
1	0	0	0	0	0	0	0	0	0	0	0	0	0	0	0	0	0	0	0	0	0	0
0	0	0	4	0	1	0	1	8	0	0	2	0	6	0	0	0	6	187	1	0	0	0
0	1	20	8	26	7	2	1	2	4	2	3	1	7	0	45	1	1	3	15	5	14	6
0	0	0	0	0	0	0	0	0	0	0	0	0	0	0	0	0	0	0	0	0	0	0
15	15	10	1	5	13	0	5	8	0	13	27	7	6	8	6	0	36	1	5	0	21	1
2	1	4	6	5	2	6	5	0	5	3	2	0	6	4	101	0	13	2	2	1	157	256
0	0	0	0	0	0	0	0	0	0	0	0	0	0	0	0	0	0	0	0	0	0	0
0	0	0	0	0	0	0	0	0	0	0	0	0	0	0	0	0	0	0	0	0	0	0
0	0	0	0	0	0	0	0	0	0	0	0	0	0	0	0	0	0	0	0	0	0	0
0	0	0	0	0	0	0	0	0	0	0	0	0	0	0	0	0	0	0	0	0	0	0
0	0	0	0	0	0	0	0	0	0	0	0	0	0	0	0	0	0	0	0	0	0	0
0	0	0	0	0	0	0	0	0	0	0	0	0	0	0	0	0	0	0	0	0	0	0
0	0	0	0	0	0	0	0	0	0	0	0	0	0	0	0	0	0	0	0	0	0	0
0	0	0	0	0	0	0	0	0	0	0	0	0	0	0	0	0	0	0	0	0	0	0
0	0	0	0	0	0	0	0	0	0	0	0	0	0	0	0	0	0	0	0	0	0	0
0	0	3	0	1	0	2	0	0	0	0	0	0	0	0	0	0	0	0	0	0	0	0
0	0	0	0	0	0	0	0	0	0	0	0	0	0	0	0	0	0	0	0	0	0	0
175	2052	403	373	80	107	406	418	805	100	390	418	187	2400	453	174	242	249	325	58	34	765	431
0	0	0	0	0	0	0	0	0	0	0	3	0	0	0	0	0	0	0	0	0	0	0
0	0	0	0	0	0	0	0	0	0	0	0	0	0	0	0	0	0	0	0	0	0	0
0	0	0	0	0	0	0	0	0	0	0	0	0	0	0	0	0	0	0	0	0	0	0
3	3	0	0	1	0	0	1	2	1	0	2	0	24	0	26	11	0	20	0	6	1	21
0	0	0	0	0	0	0	0	0	0	0	0	0	0	0	0	0	0	0	0	0	0	0
0	0	0	0	0	0	0	0	0	0	0	0	0	0	0	0	0	0	0	0	0	0	0
0	6	3	2	8	4	1	0	1	2	1	2	4	1	2	94	554	15	425	85	65	474	416
0	0	0	0	0	0	0	0	0	0	0	0	0	0	0	0	0	0	0	0	0	0	0

C2.020	C2.021	C2.024	C2.025	C2.026	C2.027	C2.029	C2.030	C2.031	C2.032	C2.033	C2.034	C2.036	C2.037	C2.038	C3.002	C3.003	C3.006	C3.009	C3.010	C3.012	C3.013	C3.014
Oral Ca	Oral Ca	Oral Ca	Oral Ca	Oral Ca	Oral Ca	Oral Ca	Oral Ca	Oral Ca	Oral Ca	Oral Ca	Oral Ca	Oral Ca	Oral Ca	Oral Ca	Oral Ca	Oral Ca	Oral Ca	Oral Ca	Oral Ca	Oral Ca	Oral Ca	Oral Ca
TD	TD	TD	TD	TD	TD	TD	TD	TD	TD	TD	TD	TD	TD	TD	SP	SP	SP	SP	SP	SP	SP	SP
0	0	0	0	0	0	0	0	0	0	0	0	0	0	0	0	0	0	0	0	0	0	0
0	0	0	0	0	0	0	0	0	0	0	0	0	0	0	0	0	0	0	0	0	0	0
0	0	0	0	0	0	0	0	0	0	0	0	0	0	0	0	0	0	0	0	0	0	0
0	0	0	0	0	0	0	0	0	0	0	0	0	0	0	0	0	0	0	0	0	0	0
0	0	0	0	0	0	0	0	0	0	0	0	0	0	0	0	0	0	0	0	0	0	0
0	0	0	0	0	0	0	0	0	0	0	0	0	0	0	0	0	0	0	0	0	0	0
0	0	0	0	0	0	0	0	0	0	0	0	0	0	0	0	0	0	0	0	0	0	0
0	0	0	0	0	0	0	0	0	0	0	0	0	0	0	0	0	0	0	0	0	0	0
0	0	0	0	0	0	0	0	0	0	0	0	0	0	0	0	0	0	0	0	0	0	0
0	0	0	0	0	0	0	0	0	0	0	0	0	0	0	0	0	0	0	0	0	0	0
0	0	0	0	0	0	0	0	0	0	0	0	0	0	0	0	0	0	0	0	0	0	0
0	0	0	0	0	0	0	0	0	0	0	0	0	0	0	0	0	0	0	0	0	0	0
0	0	0	0	0	0	0	0	0	0	0	0	0	0	0	0	0	0	0	0	0	0	0
0	0	0	0	0	0	0	0	0	0	0	0	0	0	0	0	0	0	0	0	0	0	0
0	0	0	0	0	0	0	0	0	0	0	0	0	0	0	0	0	0	0	0	0	0	0
0	0	0	0	0	0	0	0	0	0	0	0	0	0	0	0	0	0	0	0	0	0	0
0	0	1	0	0	0	0	0	0	0	0	0	0	0	0	0	0	0	0	0	0	0	0
0	0	1	0	0	0	0	0	0	0	0	0	0	0	0	0	0	0	0	0	0	0	0
0	0	0	0	0	0	0	0	0	0	0	0	0	0	0	0	0	0	0	0	0	0	0
0	0	0	0	0	0	0	0	0	0	0	0	0	0	0	0	0	0	0	0	0	0	0
0	0	0	0	0	0	0	0	0	0	0	0	0	0	0	0	0	0	0	0	0	0	0
0	0	0	0	0	0	0	0	0	0	0	0	0	0	0	0	0	0	0	0	0	0	0
0	0	1	0	0	0	1	0	0	0	0	0	0	0	0	0	0	0	0	0	0	0	0
0	0	0	0	0	0	0	0	0	0	0	0	0	0	0	0	0	0	0	0	0	0	0
0	0	1	0	0	0	0	0	0	0	0	0	0	0	0	0	0	0	0	0	0	0	0
0	0	1	0	0	0	0	0	0	0	0	0	0	0	0	0	0	0	0	0	0	0	0
0	0	0	0	0	0	0	0	0	0	0	0	0	0	0	0	0	0	0	0	0	0	0
0	0	0	0	0	0	0	0	0	0	0	0	0	0	0	0	0	0	0	0	0	0	0
0	0	0	0	0	0	0	0	0	0	0	0	0	0	0	0	0	0	0	0	0	0	0
0	0	0	0	0	0	0	0	0	0	0	0	0	0	0	0	0	0	0	0	0	0	0
0	0	0	0	0	0	0	0	0	0	0	0	0	0	0	0	0	0	0	0	0	0	0
220	516	77	22	30	17	178	315	310	274	280	298	80	1269	220	79	33	1	19	1	0	21	10
0	0	0	0	0	0	0	0	0	0	0	0	0	0	0	0	0	0	0	0	0	0	0
0	0	2	0	0	0	0	0	0	0	0	0	0	0	0	0	0	0	0	0	0	0	0
0	0	1	0	0	0	1	0	0	0	0	0	0	0	0	0	0	0	0	0	0	0	0
0	0	0	0	0	0	2	0	0	0	0	0	0	0	0	0	0	0	0	1	0	0	0
0	0	0	0	0	0	0	0	0	0	0	0	0	0	0	0	0	0	0	0	0	0	0

C2.020 Oral Ca TD	C2.021 Oral Ca TD	C2.024 Oral Ca TD	C2.025 Oral Ca TD	C2.026 Oral Ca TD	C2.027 Oral Ca TD	C2.029 Oral Ca TD	C2.030 Oral Ca TD	C2.031 Oral Ca TD	C2.032 Oral Ca TD	C2.033 Oral Ca TD	C2.034 Oral Ca TD	C2.036 Oral Ca TD	C2.037 Oral Ca TD	C2.038 Oral Ca TD	C3.002 Oral Ca SP	C3.003 Oral Ca SP	C3.006 Oral Ca SP	C3.009 Oral Ca SP	C3.010 Oral Ca SP	C3.012 Oral Ca SP	C3.013 Oral Ca SP	C3.014 Oral Ca SP
0	0	1	0	0	0	0	0	0	0	0	0	0	0	0	0	0	0	0	0	0	0	0
0	0	0	0	0	0	0	0	0	0	0	0	0	0	0	0	0	0	0	0	0	0	0
0	0	0	0	0	0	0	0	0	0	0	0	0	0	0	0	0	0	0	0	0	0	0
0	0	0	0	0	0	1	0	0	0	0	0	0	0	0	0	0	0	0	0	0	0	0
0	0	1	0	0	0	0	0	0	0	0	0	0	0	0	0	0	0	0	0	0	0	0
0	0	0	0	0	0	0	0	0	0	0	0	0	0	0	0	0	0	0	0	0	0	0
0	0	0	0	0	0	0	0	0	0	0	0	0	0	0	0	0	0	9	0	0	0	0
0	0	0	0	0	0	0	0	0	0	0	0	0	0	0	0	0	0	0	0	0	0	0
0	0	2	0	3	0	2	0	0	0	0	0	0	0	0	0	0	0	0	0	0	0	0
0	0	0	0	0	0	0	0	0	0	0	0	0	0	0	0	0	0	0	0	0	0	0
0	0	0	0	0	0	0	0	0	0	0	0	0	0	0	0	0	0	0	0	0	0	0
0	0	0	0	0	0	0	0	0	0	0	0	0	0	0	0	0	0	0	0	0	0	0
0	0	0	0	0	0	0	0	0	0	0	0	0	0	0	0	0	0	0	0	0	0	0
0	0	0	0	0	0	0	0	0	0	0	0	0	0	0	0	0	0	0	0	0	0	0
0	0	0	0	0	0	0	0	0	1	0	0	0	0	0	0	0	1	0	0	0	0	0
0	0	0	0	0	0	0	0	0	0	0	0	0	0	0	0	0	0	1	0	0	0	0
0	5	2	1	2	1	0	3	3	0	2	2	1	1	0	24	190	25	215	86	20	40	347
0	0	1	0	0	0	2	0	0	0	0	0	0	0	0	0	0	0	0	0	0	0	0
0	0	0	0	0	0	0	0	0	0	0	0	0	0	0	0	0	0	0	0	0	0	0
0	0	0	0	0	0	0	0	0	0	0	0	0	0	0	0	0	0	0	0	0	0	0
0	0	0	0	0	0	0	0	0	0	0	0	0	0	0	0	0	0	0	0	0	0	0
0	0	0	0	0	0	0	0	0	0	0	0	0	0	0	0	0	0	0	0	0	0	0
0	0	0	0	0	0	0	0	0	0	0	0	0	0	0	0	0	0	0	0	0	0	0
0	0	0	0	0	0	0	0	0	0	0	0	0	0	0	0	0	0	0	0	0	0	0
0	0	0	0	0	0	0	0	0	0	0	0	0	0	0	0	0	0	0	0	0	0	0
0	0	0	0	0	0	0	0	0	0	0	0	0	0	0	0	0	0	0	0	0	0	0
0	0	0	0	0	0	0	0	0	0	0	0	0	0	0	0	0	0	0	0	0	0	0
0	0	3	1	0	0	2	0	1	0	0	0	2	12	0	0	0	1	20	2	0	1038	0
0	0	0	0	0	0	0	0	0	0	0	0	0	0	0	0	0	0	0	0	0	0	0
3	1	0	0	0	0	6	0	4	0	0	0	3	0	1	0	0	0	0	0	0	2	0
0	0	5	0	0	0	0	0	0	0	0	0	0	0	0	0	0	0	0	2	1	0	0
0	0	195	4	114	19	28	0	0	0	0	21	0	17	0	0	0	0	0	93	155	0	0
0	0	0	0	0	0	0	0	0	0	0	0	0	0	0	0	0	0	0	0	0	0	0
99	123	15	4	4	1	31	22	38	3	43	47	8	210	46	8	1	5	0	0	1	10	0
0	0	0	0	0	0	0	0	0	0	0	0	0	0	0	0	0	0	0	0	0	0	0
0	0	0	0	0	0	0	0	0	0	0	0	0	0	0	0	0	0	1	0	0	0	0
0	0	0	0	0	0	0	0	0	0	2	0	0	0	0	0	0	0	1	0	0	3	1

C2.020	C2.021	C2.024	C2.025	C2.026	C2.027	C2.029	C2.030	C2.031	C2.032	C2.033	C2.034	C2.036	C2.037	C2.038	C3.002	C3.003	C3.006	C3.009	C3.010	C3.012	C3.013	C3.014
Oral Ca	Oral Ca	Oral Ca	Oral Ca	Oral Ca	Oral Ca	Oral Ca	Oral Ca	Oral Ca	Oral Ca	Oral Ca	Oral Ca	Oral Ca	Oral Ca	Oral Ca	Oral Ca	Oral Ca	Oral Ca	Oral Ca	Oral Ca	Oral Ca	Oral Ca	Oral Ca
TD	TD	TD	TD	TD	TD	TD	TD	TD	TD	TD	TD	TD	TD	TD	SP	SP	SP	SP	SP	SP	SP	SP
1	0	0	0	0	0	1	0	1	0	0	0	0	0	0	1	0	0	0	0	0	10	0
0	1	7	1	11	2	1	1	2	0	0	1	4	0	0	12	0	6	0	1	22	87	2
0	0	0	0	0	0	0	0	0	0	0	0	0	0	0	0	0	0	0	0	0	0	0
0	0	0	0	0	0	0	0	0	0	0	0	0	0	0	0	0	0	0	0	0	0	0
0	0	0	0	0	0	0	0	0	0	0	0	0	0	0	0	0	0	0	0	0	0	0
0	0	1	0	0	0	1	0	0	0	0	0	0	0	0	0	0	0	0	0	0	0	0
0	0	0	0	0	0	0	0	0	0	0	0	0	0	0	0	0	0	0	0	0	0	0
0	0	0	0	0	0	0	0	0	0	0	0	0	0	0	0	0	0	0	0	0	0	0
0	0	0	0	0	0	1	0	0	0	0	0	0	0	0	0	0	0	0	0	0	0	0
0	0	0	0	0	0	0	0	0	0	0	0	0	0	0	0	0	0	0	0	0	0	0
0	0	0	0	0	0	0	0	0	0	0	0	0	0	0	0	0	0	0	0	0	0	0
0	0	0	0	0	3	1	0	0	1	0	0	0	0	0	0	0	0	0	2	1	0	0
0	0	2	0	0	0	5	0	0	0	0	0	0	0	0	0	0	0	0	0	0	0	0
0	0	0	0	0	0	0	0	0	0	0	0	0	0	0	0	0	0	0	0	0	0	0
0	0	0	0	0	0	0	0	0	0	0	0	0	0	0	0	0	0	0	0	0	0	0
0	0	0	0	0	0	0	0	0	0	0	0	0	0	0	0	0	0	0	0	0	0	0
0	0	0	0	0	0	0	0	0	0	0	0	0	0	0	0	0	0	0	0	0	0	0
0	0	0	0	0	0	0	0	0	0	0	3	0	0	0	0	0	0	3	0	0	0	0
1598	1366	471	726	442	1951	583	2276	890	1177	2056	1124	762	1312	2409	500	6	568	903	16	71	80	57
0	0	0	0	1	0	1	0	0	0	0	0	0	0	0	0	0	0	1	0	0	0	0
0	0	0	0	0	0	0	0	0	0	0	0	0	0	0	0	0	0	0	0	0	0	0
0	0	0	0	0	0	0	2	0	0	0	0	0	0	0	12	0	2	0	0	0	47	0
2	1	0	3	0	5	0	0	0	4	0	3	1	5	2	4	0	6	24	4	4	13	0
0	0	1	0	0	6	0	0	0	0	0	10	0	0	0	0	0	0	0	0	0	8	0
0	0	0	0	1	3	2	0	0	0	0	0	0	0	0	0	0	0	2	0	0	0	0
0	0	0	0	0	0	0	0	0	0	0	0	0	0	0	0	0	0	0	0	0	0	0
2	4	6	1	7	10	2	1	3	1	0	3	1	3	0	188	148	211	143	108	14	52	31
0	0	0	0	1	0	1	0	0	0	0	0	0	0	0	0	0	0	6	0	1	0	0
29	733	400	290	1315	1061	114	1072	3707	2307	189	1405	1622	131	229	748	216	552	447	190	132	130	201
4	2	94	4	10	12	2	21	15	13	8	11	1	6	4	469	110	77	78	35	41	77	21
0	0	0	0	0	0	0	0	0	0	0	0	0	0	0	0	0	0	0	0	0	0	0
10808	4995	1495	2160	703	812	1310	3249	2910	1017	2895	2990	877	4438	5443	7983	519	3781	2581	217	534	4825	1908
0	0	0	0	0	0	0	0	0	0	0	0	0	0	0	0	0	0	0	0	0	2	0
0	0	0	0	0	0	0	0	0	0	0	0	0	0	0	0	0	0	0	0	0	0	0
0	0	0	0	0	0	0	0	0	0	0	0	0	0	0	0	0	0	0	0	0	0	0
0	0	0	0	0	0	0	0	0	0	0	0	0	0	0	0	0	0	0	0	0	0	0
0	0	0	0	0	0	0	0	0	0	0	0	0	0	0	0	0	0	11	0	0	0	0

C2.020	C2.021	C2.024	C2.025	C2.026	C2.027	C2.029	C2.030	C2.031	C2.032	C2.033	C2.034	C2.036	C2.037	C2.038	C3.002	C3.003	C3.006	C3.009	C3.010	C3.012	C3.013	C3.014
Oral Ca	Oral Ca	Oral Ca	Oral Ca	Oral Ca	Oral Ca	Oral Ca	Oral Ca	Oral Ca	Oral Ca	Oral Ca	Oral Ca	Oral Ca	Oral Ca	Oral Ca	Oral Ca	Oral Ca	Oral Ca	Oral Ca	Oral Ca	Oral Ca	Oral Ca	Oral Ca
TD	TD	TD	TD	TD	TD	TD	TD	TD	TD	TD	TD	TD	TD	TD	SP	SP	SP	SP	SP	SP	SP	SP
0	0	0	0	1	4	0	0	0	0	0	0	2	0	0	0	0	0	6	1	3	0	0
0	0	0	0	0	0	0	0	0	0	0	0	0	0	0	0	0	0	0	0	0	0	0
0	0	0	0	0	0	0	0	0	0	0	0	0	0	0	0	0	0	0	0	0	0	0
0	0	0	0	0	0	0	0	0	0	0	0	0	0	0	0	0	0	0	0	0	0	0
0	0	2	0	1	0	1	0	0	0	0	0	0	0	0	0	0	0	0	0	0	0	0
0	0	0	0	0	0	0	0	0	0	0	0	0	0	0	0	0	0	0	0	0	0	0
0	0	0	0	0	0	0	0	0	0	0	0	0	0	0	0	0	0	0	0	0	0	0
0	0	0	0	0	0	0	0	0	0	0	0	0	0	0	0	0	0	0	0	0	0	0
0	0	0	0	0	0	0	0	0	0	0	0	0	0	0	0	0	0	0	0	0	0	0
0	0	0	0	0	0	0	0	0	0	0	0	0	0	0	0	0	0	0	0	0	0	0
0	0	0	0	0	0	0	0	0	0	0	0	0	0	0	0	0	0	0	0	0	0	0
0	0	0	0	0	0	0	0	0	0	0	0	0	0	0	0	0	0	0	0	0	0	0
0	0	3	0	4	1	4	0	1	0	0	0	0	0	0	0	0	0	0	0	0	0	0
0	0	0	0	0	0	0	0	0	0	0	0	0	0	0	0	0	0	0	0	0	0	0
0	0	1	0	2	0	0	0	0	0	0	0	0	0	0	0	0	0	0	0	0	0	0
0	0	0	0	0	0	0	0	0	0	0	0	0	0	0	0	0	0	0	0	0	0	0
0	0	0	0	0	0	0	0	0	0	0	0	0	0	0	0	0	0	0	1	0	0	0
0	82	10	20	58	21	3	25	22	32	4	12	29	26	10	11	164	1410	70	627	17	24	104
0	0	0	0	0	0	0	0	0	0	0	0	0	0	0	0	0	0	0	0	0	0	0
11	104	165	146	156	372	17	151	265	205	35	106	75	50	28	438	3384	3131	3885	718	7263	1799	4427
0	0	3	0	4	0	1	0	0	0	0	0	0	0	0	0	0	0	0	0	0	0	0
0	0	0	0	0	0	0	0	0	0	0	0	0	0	0	0	0	0	0	0	1	0	0
0	0	0	0	0	0	0	0	0	0	0	0	0	0	0	0	0	0	1	0	0	0	0
0	0	42	0	3	0	18	0	0	0	0	0	0	0	1	0	0	0	0	0	0	0	0
0	0	0	0	0	0	0	0	0	0	0	0	0	0	0	0	0	0	0	0	0	0	0
0	0	3	0	4	0	1	0	0	0	0	0	0	0	0	0	0	0	0	0	0	0	0
0	0	0	0	0	0	0	0	0	0	0	0	0	0	0	0	0	0	0	0	0	0	0
0	0	0	0	0	0	0	0	0	0	0	0	0	0	0	0	0	0	0	0	0	0	0
0	0	0	0	0	0	0	0	0	0	0	1	0	0	0	0	0	0	0	0	0	0	0
0	0	0	0	0	0	0	0	0	0	0	0	0	0	0	0	0	0	0	0	0	0	0
0	0	0	0	0	0	0	0	0	0	0	0	0	0	0	0	0	0	0	0	0	0	0
0	0	0	0	0	0	0	0	0	0	0	0	0	0	0	0	0	0	0	0	0	0	0
0	0	0	0	0	0	0	0	0	0	0	0	0	0	0	0	0	0	0	0	0	0	0
0	0	0	0	0	0	0	0	0	0	0	0	0	0	0	0	0	0	0	0	0	0	0
0	0	0	0	0	0	0	0	0	0	0	0	0	0	0	0	0	0	0	0	0	0	0
0	0	3	0	1	0	1	0	0	0	0	0	0	0	0	0	0	0	0	0	0	0	0
0	0	0	0	0	0	0	0	0	0	0	0	0	0	0	0	0	1	0	0	0	0	0

C2.020	C2.021	C2.024	C2.025	C2.026	C2.027	C2.029	C2.030	C2.031	C2.032	C2.033	C2.034	C2.036	C2.037	C2.038	C3.002	C3.003	C3.006	C3.009	C3.010	C3.012	C3.013	C3.014
Oral Ca	Oral Ca	Oral Ca	Oral Ca	Oral Ca	Oral Ca	Oral Ca	Oral Ca	Oral Ca	Oral Ca	Oral Ca	Oral Ca	Oral Ca	Oral Ca	Oral Ca	Oral Ca	Oral Ca	Oral Ca	Oral Ca	Oral Ca	Oral Ca	Oral Ca	Oral Ca
TD	TD	TD	TD	TD	TD	TD	TD	TD	TD	TD	TD	TD	TD	TD	SP	SP	SP	SP	SP	SP	SP	SP
0	0	0	0	0	0	0	0	0	0	0	0	0	0	0	0	0	0	0	0	0	0	0
0	0	0	0	0	0	0	0	0	0	0	0	0	0	0	0	0	0	0	0	0	0	0
0	0	44	11	13	3	4	0	0	0	0	0	0	0	3	0	0	0	0	7	3	0	0
0	0	0	0	0	0	0	0	0	0	0	0	0	0	0	0	0	0	0	0	0	0	0
0	0	0	0	0	0	0	0	0	0	0	0	0	0	0	0	0	0	0	0	0	0	0
0	0	0	0	0	0	0	0	0	0	0	0	0	0	0	0	0	0	0	0	0	0	0
0	0	1	0	0	0	0	0	0	0	0	0	0	0	0	0	0	0	0	0	0	0	0
0	0	1	0	0	0	1	0	0	0	0	0	0	0	0	0	0	0	0	0	0	0	0
0	0	0	0	0	0	0	0	0	0	0	0	0	0	0	0	0	0	0	0	0	0	0
0	0	0	0	0	0	0	0	0	0	0	0	0	0	0	0	0	0	0	0	0	0	0
0	0	0	0	0	0	0	0	0	0	0	0	0	0	0	0	0	0	0	0	0	0	0
0	0	1	0	0	0	0	0	0	0	0	0	0	0	0	0	0	0	0	0	0	0	0
0	0	0	0	0	0	0	0	0	0	0	0	0	0	0	0	0	0	0	0	0	0	0
0	0	0	0	0	0	0	0	0	0	0	0	0	0	0	0	0	0	0	0	0	0	0
0	0	0	0	1	0	0	0	0	0	0	0	0	0	0	0	0	0	0	0	0	0	0
0	0	0	0	0	0	0	0	0	0	0	0	0	0	0	0	0	0	0	0	0	0	0
0	0	0	0	0	0	0	0	0	0	0	0	0	0	0	0	0	0	0	0	0	0	0
0	0	0	0	0	0	0	0	0	0	0	0	0	0	0	0	0	0	0	0	0	0	0
0	0	0	0	0	0	0	0	0	0	0	0	0	0	0	0	0	0	0	0	0	0	0
0	2	1	2	1	0	0	0	0	0	0	0	0	0	0	0	0	0	0	0	0	0	0
0	0	0	0	0	0	0	0	0	0	0	0	0	0	0	0	0	0	0	0	0	0	0
0	0	0	0	0	0	0	0	0	0	0	0	0	0	0	0	0	0	0	0	0	0	0
0	0	40	0	57	0	4	0	0	0	0	0	0	0	0	0	0	0	0	12	5	0	0
0	0	0	0	0	0	0	0	0	0	0	0	0	0	0	0	0	0	0	0	0	0	0
10	112	26	20	31	17	21	34	222	280	77	171	80	93	33	31	11	173	21	0	7	28	5
0	0	0	0	0	0	0	0	0	0	0	0	0	0	0	0	0	0	0	0	0	0	0
0	8	6	3	1	1	2	5	34	4	2	16	12	9	5	0	16	18	23	0	7	0	35
0	0	0	1	51	14	9	0	0	0	0	0	0	0	0	0	0	0	0	29	85	0	0
0	0	0	0	0	0	0	0	0	0	0	0	0	0	0	0	0	0	0	0	0	0	0
0	0	0	0	0	0	0	0	0	0	0	0	0	0	0	0	0	0	0	0	0	0	0
0	0	0	0	0	0	0	0	0	0	0	0	0	0	0	0	0	0	0	0	0	0	0
0	0	0	0	0	0	0	0	0	0	0	0	0	0	0	0	0	0	0	0	0	0	0
0	0	0	0	0	0	0	0	0	0	0	0	0	0	0	0	0	0	0	0	0	0	1
0	0	0	0	0	0	0	0	0	0	0	0	0	0	0	0	0	0	0	0	0	0	0
0	0	0	0	0	0	0	0	0	0	0	0	0	0	0	0	0	0	0	0	0	0	0
0	0	0	0	0	0	0	0	0	0	0	0	0	0	0	0	0	0	0	0	0	0	0
316	842	89	121	163	123	443	1080	1432	560	840	828	773	204	314	10	9	4	15	10	12	22	40

C2.020	C2.021	C2.024	C2.025	C2.026	C2.027	C2.029	C2.030	C2.031	C2.032	C2.033	C2.034	C2.036	C2.037	C2.038	C3.002	C3.003	C3.006	C3.009	C3.010	C3.012	C3.013	C3.014
Oral Ca	Oral Ca	Oral Ca	Oral Ca	Oral Ca	Oral Ca	Oral Ca	Oral Ca	Oral Ca	Oral Ca	Oral Ca	Oral Ca	Oral Ca	Oral Ca	Oral Ca	Oral Ca	Oral Ca	Oral Ca	Oral Ca	Oral Ca	Oral Ca	Oral Ca	Oral Ca
TD	TD	TD	TD	TD	TD	TD	TD	TD	TD	TD	TD	TD	TD	TD	SP	SP	SP	SP	SP	SP	SP	SP
0	0	0	0	0	0	0	0	0	0	0	0	0	0	0	0	0	0	0	0	0	0	0
0	0	0	0	0	0	0	0	0	0	0	0	0	0	0	0	0	0	0	0	0	0	0
0	0	0	0	0	0	0	0	0	0	0	0	0	0	0	0	0	0	0	0	0	0	0
0	0	0	0	0	0	0	0	0	0	0	0	0	0	2	0	0	0	0	0	0	0	1
0	0	0	0	0	0	0	0	0	0	0	0	0	0	0	0	0	0	0	0	0	0	0
0	0	1	0	0	0	0	0	0	0	0	0	0	0	0	0	0	0	0	0	1	0	0
0	0	0	0	0	0	0	0	0	0	0	0	0	0	0	0	0	0	0	0	0	0	0
0	0	131	25	428	111	416	0	4	0	2	0	2	6	0	0	0	0	8	201	145	178	0
0	0	0	0	0	0	0	0	0	0	0	0	7	0	0	0	0	0	0	0	0	0	0
0	0	0	0	0	0	0	0	0	0	0	0	0	0	0	0	0	0	0	0	0	0	0
891	1862	577	993	475	217	1896	896	2552	658	1273	1900	1080	3613	869	156	303	832	319	75	168	1090	325
0	0	2	0	0	0	0	0	0	0	0	0	0	0	0	0	0	0	0	0	0	0	0
2	5	4	7	4	6	3	2	3	0	1	0	0	1	0	0	1	0	0	0	0	0	2
0	0	0	0	0	0	0	0	0	0	0	0	0	0	0	0	0	0	1	0	0	0	0
0	0	1	0	0	0	1	0	0	0	0	0	0	0	0	0	0	0	0	0	0	0	0
0	0	0	0	0	0	0	0	0	0	0	0	0	0	0	0	0	0	0	0	0	0	0
0	0	79	3	23	65	0	80	0	4	18	11	9	0	0	8	0	2	5	0	3	0	0
2	47	23	6	5	5	64	53	129	4	29	68	12	71	34	9	2	1	2	1	0	30	1
0	0	0	1	0	0	0	0	1	3	0	2	0	3	0	69	0	17	5	2	0	10	5
91	107	288	68	27	14	77	89	150	59	214	211	27	178	95	429	4	17	49	19	0	235	62
0	0	0	0	0	0	0	0	0	0	0	0	0	0	0	0	0	0	0	0	0	0	0
0	0	3	0	11	0	5	0	0	0	0	0	0	0	0	0	0	0	0	13	12	0	0
0	0	1	0	2	1	1	0	0	0	0	0	0	0	0	0	0	0	0	6	1	0	0
0	0	0	0	0	0	0	0	0	0	0	0	0	0	0	0	0	0	0	0	0	0	0
0	21	257	17	190	27	11	13	54	72	5	29	1	17	2	25	0	69	2	61	4	11	1
0	0	4	0	9	2	1	0	0	0	0	0	0	0	0	0	0	0	0	7	0	0	0
0	0	0	0	0	0	0	0	0	0	0	0	0	0	0	0	0	0	0	0	0	0	0
0	0	0	0	0	0	0	0	0	0	0	0	0	0	0	0	0	0	0	0	0	0	0
0	0	0	0	0	0	0	0	0	0	0	0	0	0	0	0	0	0	0	0	0	0	0
0	0	0	1	0	1	0	0	0	1	0	0	0	2	0	34	1	0	2	0	0	5	0
0	0	0	0	0	0	0	0	0	0	0	0	0	0	0	0	0	0	0	0	0	4	0
0	0	0	0	1	0	0	0	0	0	0	0	0	0	0	0	0	0	0	0	0	0	0
0	0	0	0	0	0	0	0	0	0	0	0	0	0	0	0	0	0	0	0	0	0	0
0	0	23	1	9	2	5	0	0	0	0	0	0	0	0	0	0	0	0	2	36	0	0
0	0	0	0	0	0	0	0	0	0	0	0	0	0	0	0	0	0	0	0	0	0	0
0	0	0	0	0	0	0	0	0	0	0	0	0	0	0	0	0	0	0	0	0	0	0
0	0	0	0	0	0	0	0	0	0	0	0	0	0	0	0	0	0	0	0	0	0	0

C2.020	C2.021	C2.024	C2.025	C2.026	C2.027	C2.029	C2.030	C2.031	C2.032	C2.033	C2.034	C2.036	C2.037	C2.038	C3.002	C3.003	C3.006	C3.009	C3.010	C3.012	C3.013	C3.014
Oral Ca	Oral Ca	Oral Ca	Oral Ca	Oral Ca	Oral Ca	Oral Ca	Oral Ca	Oral Ca	Oral Ca	Oral Ca	Oral Ca	Oral Ca	Oral Ca	Oral Ca	Oral Ca	Oral Ca	Oral Ca	Oral Ca	Oral Ca	Oral Ca	Oral Ca	Oral Ca
TD	TD	TD	TD	TD	TD	TD	TD	TD	TD	TD	TD	TD	TD	TD	SP	SP	SP	SP	SP	SP	SP	SP
0	0	2	0	3	0	0	0	0	0	0	0	0	0	0	0	1	0	1	0	1	0	0
0	0	0	0	0	0	0	0	0	0	0	0	0	0	0	0	0	0	0	0	0	0	0
0	0	0	0	0	0	0	0	0	0	0	0	0	0	0	0	0	0	0	0	0	0	0
0	0	0	0	0	0	0	0	0	0	0	0	0	0	0	0	0	0	0	0	0	0	0
0	0	0	1	0	0	0	0	5	1	0	2	1	3	0	1	0	0	3	0	1	677	2
0	0	0	0	0	1	0	0	0	0	0	0	0	0	0	0	0	0	1	0	2	0	0
0	0	0	0	0	0	0	0	0	0	0	0	0	0	0	0	0	0	0	0	0	0	0
0	0	0	3	5	3	0	0	0	0	0	0	0	0	0	0	0	0	5	0	4	0	0
5	28	16	5	0	1	2	32	33	0	42	13	0	23	8	0	0	1	1	1	0	3	2
0	37	46	4	37	28	26	67	84	37	124	40	73	18	47	248	12	57	140	5	24	75	88
0	0	0	0	4	2	1	0	0	1	0	0	0	0	0	0	0	0	3	0	0	0	0
0	0	0	0	3	1	1	0	0	0	0	0	0	0	0	0	0	0	3	0	3	0	0
0	0	0	0	0	0	0	0	0	0	0	0	0	0	0	0	0	0	0	0	0	0	0
0	0	0	0	0	0	0	0	0	0	0	0	0	0	0	0	0	0	2	0	0	0	0
0	0	0	0	1	0	0	0	0	0	0	0	0	0	0	0	0	0	6	0	0	0	0
0	0	1	0	0	0	0	0	0	0	0	0	0	0	0	0	0	0	0	0	0	0	0
1818	220	34	14	50	20	181	112	114	18	305	195	82	158	107	109	11	3	3	0	2	169	10
0	0	0	1	6	2	3	0	0	0	0	0	0	0	0	0	0	0	9	0	3	1	0
2	0	5	3	2	3	1	0	19	0	5	51	4	2	20	40	1	0	0	3	40	328	0
1	0	1	0	0	0	1	0	0	0	0	0	0	2	0	0	0	0	1	0	2	4	0
530	215	181	26	73	54	81	185	350	61	164	225	107	567	263	309	119	198	310	43	32	315	176
0	0	0	0	0	0	0	0	0	0	0	0	0	0	0	0	0	0	0	0	0	0	0
0	0	87	3	28	8	9	5	76	6	8	15	11	0	18	5	1	14	7	0	1	12	2
0	0	0	0	0	0	0	0	0	0	0	0	0	0	0	0	0	0	0	0	0	0	0
0	0	0	0	0	0	0	0	0	0	0	0	0	0	0	0	0	0	0	0	0	0	0
4	4	18	8	16	22	1	4	12	18	5	36	11	33	1	280	3	37	31	1	1	51	43
0	61	97	21	64	43	29	56	224	31	58	76	45	67	67	68	1	32	2	123	9	2	7
0	2	2	0	45	4	2	8	0	5	10	14	4	1	4	20	68	151	12	4	7	4	3
0	0	0	0	0	0	0	0	0	0	0	0	0	0	0	0	0	0	1	0	0	0	0
0	0	0	0	0	0	0	0	0	0	0	0	0	0	0	0	0	0	0	0	0	0	0
0	0	1	0	5	8	4	0	0	0	0	0	0	0	0	0	0	0	26	0	2	0	0
0	0	0	0	0	1	1	0	0	0	0	0	2	0	0	0	0	0	10	0	2	0	0
0	0	0	1	1	1	0	0	0	0	0	0	0	0	0	0	0	0	4	0	0	0	0
0	0	0	0	0	0	0	0	0	0	0	0	0	0	0	0	0	0	0	0	0	0	0
0	0	1	6	9	5	6	0	1	0	0	0	0	0	0	0	0	0	40	0	7	0	0
0	0	0	0	0	0	0	0	0	0	0	1	0	0	0	19	0	2	0	0	0	16	0
0	0	0	0	0	1	0	0	0	0	0	0	0	0	0	0	0	0	0	0	0	0	0

C2.020	C2.021	C2.024	C2.025	C2.026	C2.027	C2.029	C2.030	C2.031	C2.032	C2.033	C2.034	C2.036	C2.037	C2.038	C3.002	C3.003	C3.006	C3.009	C3.010	C3.012	C3.013	C3.014
Oral Ca	Oral Ca	Oral Ca	Oral Ca	Oral Ca	Oral Ca	Oral Ca	Oral Ca	Oral Ca	Oral Ca	Oral Ca	Oral Ca	Oral Ca	Oral Ca	Oral Ca	Oral Ca	Oral Ca	Oral Ca	Oral Ca	Oral Ca	Oral Ca	Oral Ca	Oral Ca
TD	TD	TD	TD	TD	TD	TD	TD	TD	TD	TD	TD	TD	TD	TD	SP	SP	SP	SP	SP	SP	SP	SP
1	8	17	4	19	12	5	5	20	4	11	171	6	2	12	622	8	85	10	3	3	257	125
0	0	0	0	0	0	0	0	0	0	0	0	0	0	0	0	0	0	0	0	0	0	0
481	147	31	11	11	5	54	46	42	0	38	107	2	229	280	119	3	1	4	2	33	96	15
0	0	0	0	0	0	0	0	0	0	0	0	0	0	0	0	0	0	0	0	0	0	0
0	0	1	0	0	1	0	0	1	0	1	0	1	1	2	87	1	47	1	0	0	66	2
86	5	24	3	5	8	3	209	15	8	93	43	6	105	86	1583	82	1018	316	19	42	1384	959
0	0	0	0	0	0	0	0	0	0	0	0	0	0	0	0	0	0	0	0	0	0	0
0	0	0	0	0	0	0	0	0	0	0	0	0	0	0	0	0	0	0	0	0	0	0
2152	1729	314	73	102	159	594	1360	781	354	763	889	287	1846	1973	490	37	297	14	4	1	417	100
0	0	1	0	0	0	0	0	0	0	0	0	0	0	0	0	0	0	1	0	0	0	0
0	0	11	1	0	0	0	0	10	0	1	0	1	0	0	9	6	8	6	0	0	196	0
0	0	1	1	0	0	0	0	0	1	0	0	0	0	0	0	0	0	1	0	4	0	0
0	0	0	0	0	0	0	0	0	0	0	0	0	0	0	0	0	0	0	0	0	0	0
0	0	0	0	2	1	0	0	0	0	0	0	0	0	0	0	0	0	1	0	0	0	0
0	0	0	0	0	0	1	0	0	0	0	0	1	0	0	0	0	0	0	0	7	0	0
107	184	168	146	26	35	189	318	121	63	278	236	234	239	240	37	1	2	6	0	3	13	14
0	0	0	0	0	0	0	0	0	0	0	0	0	0	0	0	0	0	1	0	0	0	0
0	0	0	0	0	0	0	0	0	0	0	0	0	0	0	0	0	0	0	0	0	0	0
0	0	0	0	0	0	0	0	0	0	0	0	0	0	0	0	0	0	2	0	0	0	0
0	0	0	0	0	0	0	0	0	0	0	0	0	0	0	0	0	0	1	0	0	0	0
0	0	0	1	0	0	0	0	0	0	0	0	0	0	0	0	0	1	1	0	0	33	0
1	3	0	3	0	3	0	1	1	0	0	6	1	2	0	3	0	0	5	2	0	2	0
0	0	0	0	0	0	0	0	0	0	0	0	0	0	0	0	0	0	1	0	0	0	0
0	0	0	3	1	0	0	0	0	0	0	2	1	0	0	0	0	0	0	0	1	35	0
0	0	0	0	0	0	0	0	0	0	0	0	0	0	0	0	0	0	0	0	0	0	1
158	108	1693	352	870	443	674	973	1510	525	506	1823	321	100	489	837	344	1148	511	139	423	857	638
487	172	218	58	93	30	170	324	261	52	726	328	89	666	366	257	230	418	442	70	108	464	435
0	0	45	0	30	5	0	0	0	0	0	0	0	0	0	0	0	0	0	20	5	0	0
0	0	0	0	0	0	0	0	0	0	0	0	0	0	0	0	0	0	0	0	0	0	0
0	0	0	6	0	1	0	1	1	3	11	0	0	0	0	1	0	2	0	0	0	0	0
0	0	0	0	0	0	0	0	0	0	0	0	0	0	0	0	0	0	0	0	0	0	0
0	0	0	0	0	0	0	0	0	0	0	0	0	0	0	0	0	0	0	0	0	0	0
0	0	0	0	0	0	1	0	0	0	0	0	0	0	0	0	0	0	0	0	0	0	0
0	0	0	0	0	0	0	0	0	0	0	0	0	0	0	0	0	0	0	0	0	0	0
0	0	0	1	0	1	1	0	0	0	0	0	0	0	0	0	0	0	0	0	2	0	0
0	0	0	0	0	0	0	0	0	0	0	0	0	0	0	0	0	0	0	0	0	0	0
0	0	4	0	0	1	1	0	0	0	0	0	0	0	0	0	0	0	0	0	0	0	0

C2.020 Oral Ca TD	C2.021 Oral Ca TD	C2.024 Oral Ca TD	C2.025 Oral Ca TD	C2.026 Oral Ca TD	C2.027 Oral Ca TD	C2.029 Oral Ca TD	C2.030 Oral Ca TD	C2.031 Oral Ca TD	C2.032 Oral Ca TD	C2.033 Oral Ca TD	C2.034 Oral Ca TD	C2.036 Oral Ca TD	C2.037 Oral Ca TD	C2.038 Oral Ca TD	C3.002 Oral Ca SP	C3.003 Oral Ca SP	C3.006 Oral Ca SP	C3.009 Oral Ca SP	C3.010 Oral Ca SP	C3.012 Oral Ca SP	C3.013 Oral Ca SP	C3.014 Oral Ca SP
0	0	3	0	0	1	1	0	0	0	0	0	0	0	0	0	0	0	0	12	3	0	0
0	0	0	0	0	0	0	0	0	0	0	0	0	0	0	0	0	0	0	0	0	0	0
61	67	6	2	4	3	6	52	25	68	0	65	116	71	49	31	111	94	0	3	10	113	3
0	0	0	0	0	0	0	0	0	0	0	0	0	0	0	0	0	0	0	0	0	0	0
0	0	0	0	0	0	1	0	0	0	0	0	0	0	0	0	0	0	0	0	0	0	0
0	0	0	0	0	0	0	0	0	0	0	0	0	0	0	0	0	0	0	0	0	0	0
0	0	0	0	0	0	0	0	0	0	0	0	0	0	0	0	0	0	0	0	0	0	0
0	0	0	0	0	0	0	0	0	0	0	0	0	0	0	0	0	0	0	0	0	0	0
0	0	1	0	0	0	0	0	0	0	0	0	0	0	0	0	0	0	0	1	1	0	0
0	0	0	0	0	0	2	0	0	0	0	0	0	0	0	0	0	0	0	0	0	0	0
0	0	3	0	0	0	1	0	0	0	0	0	0	0	0	0	0	0	0	0	0	0	0
1	1	6	0	2	0	4	1	0	1	0	0	0	1	0	1	0	2	0	0	1	0	0
0	0	0	0	1	0	0	0	0	0	0	0	0	0	0	0	0	0	0	0	0	0	0
0	0	2	0	0	0	3	0	0	0	0	0	0	0	0	0	0	0	0	0	1	0	0
0	0	0	3	7	4	1	0	0	0	0	0	0	0	0	0	0	0	1	3	5	0	0
0	0	0	0	0	0	0	0	0	0	0	0	0	0	0	0	0	0	0	1	0	0	0
0	0	24	0	3	0	6	0	0	0	0	0	0	0	0	0	0	0	0	0	0	0	0
0	0	1	0	1	0	0	0	0	0	0	0	0	0	0	0	0	0	0	0	1	0	0
0	0	0	0	0	0	1	0	0	0	0	0	0	0	0	0	0	0	0	0	0	0	0
0	0	2	0	0	0	1	0	0	0	0	0	0	0	0	0	0	0	0	0	0	0	0
0	0	0	0	0	0	0	0	0	0	0	0	0	0	0	0	0	0	0	0	0	0	0
0	0	1	0	0	0	2	0	0	0	0	0	0	0	0	0	0	0	0	0	0	0	0
0	0	2	0	0	0	0	0	0	0	0	0	0	0	0	0	0	0	0	0	3	0	0
0	0	0	0	0	1	1	0	0	0	0	0	0	0	0	0	0	0	0	0	1	0	0
0	0	0	0	0	0	1	0	0	0	0	0	0	0	0	0	0	0	0	0	0	0	0
0	0	0	0	0	0	0	0	0	0	0	0	0	0	0	0	0	0	0	0	0	0	0
0	0	0	0	0	0	0	0	0	0	0	0	0	0	0	0	0	0	0	0	0	0	0
0	0	1	0	0	0	0	0	0	0	0	0	0	0	0	0	0	0	0	0	0	0	0
0	0	1	0	0	0	1	0	0	0	0	0	0	0	0	0	0	0	0	0	0	0	0
0	0	8	0	10	1	5	0	0	0	0	0	0	0	0	0	0	0	0	0	0	0	1
0	0	1	0	0	0	0	0	0	0	0	0	0	0	0	0	0	0	0	0	0	0	0
0	0	0	0	2	0	1	0	0	0	0	0	0	0	0	0	0	0	0	0	0	0	0
0	0	0	0	0	0	0	0	0	0	0	0	0	0	0	0	0	0	0	0	0	0	0
0	0	2	0	0	0	3	0	0	0	0	0	0	0	0	0	0	0	0	0	0	0	0
0	0	6	0	6	0	5	0	0	0	0	0	0	0	0	0	0	0	0	0	0	0	0
0	0	0	0	0	0	0	0	0	0	0	0	0	0	0	0	0	0	0	0	0	0	0
0	0	0	0	0	0	0	0	0	0	0	0	0	0	0	0	0	0	0	0	0	0	0

C2.020	C2.021	C2.024	C2.025	C2.026	C2.027	C2.029	C2.030	C2.031	C2.032	C2.033	C2.034	C2.036	C2.037	C2.038	C3.002	C3.003	C3.006	C3.009	C3.010	C3.012	C3.013	C3.014
Oral Ca	Oral Ca	Oral Ca	Oral Ca	Oral Ca	Oral Ca	Oral Ca	Oral Ca	Oral Ca	Oral Ca	Oral Ca	Oral Ca	Oral Ca	Oral Ca	Oral Ca	Oral Ca	Oral Ca	Oral Ca	Oral Ca	Oral Ca	Oral Ca	Oral Ca	Oral Ca
TD	TD	TD	TD	TD	TD	TD	TD	TD	TD	TD	TD	TD	TD	TD	SP	SP	SP	SP	SP	SP	SP	SP
0	0	0	0	0	0	0	0	0	0	0	0	0	0	0	0	0	0	0	0	0	0	0
0	0	0	0	0	0	0	0	0	0	0	0	0	0	0	0	0	0	0	0	1	0	0
0	0	0	0	0	0	0	0	0	0	0	0	0	0	0	0	0	0	0	0	0	0	0
0	0	0	0	0	0	0	0	0	0	0	0	0	0	0	0	0	0	0	0	0	0	0
0	0	0	0	0	0	0	0	0	0	0	0	0	0	0	0	0	0	0	0	0	0	0
0	0	9	0	0	0	4	0	0	0	0	0	0	0	0	0	0	0	0	1	0	0	0
0	0	0	0	0	0	0	0	0	0	0	0	0	0	0	0	0	0	0	0	0	0	0
0	0	2	0	0	0	0	0	0	0	0	0	0	0	0	0	0	0	0	0	0	0	0
0	0	1	0	0	0	1	0	0	0	0	0	0	0	0	0	0	0	0	0	0	0	0
0	0	0	94	0	0	0	0	0	0	0	0	0	0	0	0	0	0	0	0	0	0	0
0	0	0	0	0	0	0	0	0	0	0	0	0	0	0	0	0	0	0	0	0	0	0
0	0	0	0	0	0	0	0	0	0	0	0	0	0	0	0	0	0	0	0	0	0	0
0	0	0	0	0	0	0	0	0	0	0	0	0	0	0	0	0	0	0	0	0	0	0
0	0	0	15	0	0	0	0	0	0	0	0	0	0	0	0	0	0	0	0	0	0	0
0	0	0	3	0	2	1	0	0	0	0	0	0	0	0	0	0	0	0	0	2	0	0
0	0	0	0	0	0	0	0	0	0	0	0	0	0	0	0	0	0	0	0	0	0	0
0	0	0	0	0	0	0	0	0	0	0	0	0	0	0	0	0	0	0	0	0	0	0
0	0	0	0	0	0	0	0	0	0	0	0	0	0	0	0	0	0	0	0	0	0	0
0	0	0	0	0	0	0	0	0	0	0	0	0	0	0	0	0	0	0	0	0	0	0
0	0	3	0	0	0	0	0	0	0	0	0	0	0	0	0	0	0	1	0	0	0	0
0	0	0	0	0	0	0	0	0	0	0	0	0	0	0	0	0	0	0	0	0	0	0
0	0	0	0	0	0	0	0	0	0	0	0	0	0	0	0	0	0	0	0	0	0	0
0	0	0	0	0	0	0	0	0	0	0	0	0	0	0	0	0	0	0	0	0	0	0
0	0	0	0	0	0	0	0	0	0	0	0	0	0	0	0	0	0	0	0	0	0	0
0	0	0	0	0	0	0	0	0	0	0	0	0	0	0	0	0	0	0	0	0	0	0
0	0	0	0	0	0	0	0	0	0	0	0	0	0	0	0	0	0	0	0	0	0	0
0	0	7	0	0	0	4	0	0	0	0	0	0	0	0	0	0	0	0	0	0	0	0
0	0	2	0	2	4	1	0	1	1	0	0	0	0	0	0	0	0	0	1	3	0	0
0	0	0	1	0	3	0	0	0	0	0	0	0	0	0	0	0	1	1	0	4	0	0
0	1	651	0	416	65	6	0	0	0	0	0	0	0	0	0	0	0	0	8	92	1	0
0	0	1	0	0	0	1	0	0	0	0	0	0	0	0	0	0	0	0	0	0	0	0
0	0	0	0	0	0	0	0	0	0	0	0	0	0	0	0	0	0	0	0	0	0	0
0	0	0	0	1	0	1	0	0	0	0	0	0	0	0	0	0	0	0	0	1	0	0
0	0	0	0	0	0	0	0	0	0	0	0	0	0	0	0	0	0	0	0	0	0	0
0	0	0	0	0	0	0	0	0	0	0	0	0	0	0	0	0	0	0	0	0	0	0
0	0	1	0	0	0	0	0	0	0	0	0	0	0	0	0	0	0	0	0	0	0	0
0	0	1	0	3	0	0	0	0	0	0	0	0	0	0	0	0	0	6	0	0	0	0

C2.020	C2.021	C2.024	C2.025	C2.026	C2.027	C2.029	C2.030	C2.031	C2.032	C2.033	C2.034	C2.036	C2.037	C2.038	C3.002	C3.003	C3.006	C3.009	C3.010	C3.012	C3.013	C3.014
Oral Ca	Oral Ca	Oral Ca	Oral Ca	Oral Ca	Oral Ca	Oral Ca	Oral Ca	Oral Ca	Oral Ca	Oral Ca	Oral Ca	Oral Ca	Oral Ca	Oral Ca	Oral Ca	Oral Ca	Oral Ca	Oral Ca	Oral Ca	Oral Ca	Oral Ca	Oral Ca
TD	TD	TD	TD	TD	TD	TD	TD	TD	TD	TD	TD	TD	TD	TD	SP	SP	SP	SP	SP	SP	SP	SP
0	0	0	0	0	0	0	0	0	0	0	0	0	0	0	0	0	0	0	0	0	0	0
0	0	2	1	0	1	1	0	0	0	0	0	0	0	0	0	0	0	0	0	0	0	0
0	0	0	0	0	0	0	0	0	0	0	0	0	0	0	0	0	0	0	0	0	0	0
25	36	36	39	24	11	24	7	4	1	7	20	9	13	9	74	284	5	123	136	26	1	320
0	0	0	0	0	0	0	0	0	0	0	0	0	0	0	0	0	0	0	0	0	0	0
0	0	0	0	0	0	0	0	0	0	0	0	0	0	0	0	0	0	0	0	0	0	0
0	0	2	0	2	2	2	0	0	0	0	0	0	0	0	0	0	0	0	0	0	0	0
0	0	0	0	0	0	0	0	0	0	0	0	0	0	0	0	0	0	0	0	0	0	0
0	0	1	0	0	0	0	0	0	0	0	0	0	0	0	0	0	0	0	0	0	0	0
0	0	2	0	0	0	0	0	0	0	0	0	0	0	0	0	0	0	0	0	1	0	0
0	0	0	0	0	0	0	0	0	0	0	0	0	0	0	0	0	0	0	0	0	0	0
0	0	0	0	0	0	0	0	0	0	0	0	0	0	0	0	0	0	0	0	0	0	0
0	0	0	0	0	0	0	0	0	0	0	0	0	0	0	0	0	0	0	0	0	0	0
0	0	0	0	0	0	0	0	0	0	0	0	0	0	0	0	0	0	0	0	0	0	0
0	0	4	0	4	0	2	0	0	0	0	0	0	0	0	0	0	0	0	0	0	0	0
0	0	0	0	0	0	0	0	0	0	0	0	0	0	0	0	0	0	0	0	0	0	0
0	0	0	0	0	0	0	0	0	0	0	0	0	0	0	0	0	0	0	0	0	0	0
0	0	1	0	1	0	0	0	0	0	0	0	0	0	0	0	0	0	0	0	0	0	0
0	0	0	0	0	0	0	0	0	0	0	0	0	0	0	0	0	0	0	0	0	0	0
0	0	0	0	0	0	0	0	0	0	0	0	0	0	0	0	0	0	0	0	0	0	0
0	0	2	0	3	0	1	0	0	0	0	0	0	0	0	0	0	0	0	0	0	0	0
0	0	4	1	11	8	1	1	0	0	0	0	2	0	0	1	5	0	0	208	0	0	1
0	34	39	9	38	31	10	8	21	2	3	7	3	5	3	76	254	1590	73	454	269	14	64
0	0	0	0	1	0	0	0	0	0	0	0	0	0	1	0	0	0	0	0	0	0	0
0	0	4	0	2	0	2	0	0	0	0	0	0	0	0	0	0	0	0	0	0	0	0
0	0	0	0	0	0	0	0	0	0	0	0	0	0	0	0	0	0	0	0	0	0	0
0	0	1	0	1	0	2	0	0	0	0	0	0	0	0	0	0	1	0	0	0	0	0
0	0	2	0	2	0	0	0	0	0	0	0	0	0	0	0	0	0	1	0	1	0	0
0	0	0	0	0	0	0	0	0	0	0	0	0	0	0	0	0	0	0	0	0	0	0
0	0	0	0	0	0	0	0	0	0	0	0	0	0	0	0	0	0	0	0	0	0	0
0	0	0	0	0	0	0	0	0	0	0	0	0	0	0	0	0	0	0	0	0	0	0
0	0	0	0	0	0	0	0	0	0	0	0	0	0	0	0	0	0	0	0	0	0	0
3	24	9	25	39	50	9	5	17	18	4	23	50	7	3	6	60	218	171	20	355	35	30
0	0	5	13	6	4	3	5	5	26	0	21	18	3	0	1	2	0	9	0	9	100	121
40	7373	4402	4470	2789	3112	637	8776	2510	14495	3721	9301	12187	3261	2587	671	1298	913	2245	212	690	55	349
2	1	51	23	21	27	3	7	8	3	13	11	58	0	1	0	2	5	15	4	4	0	3
0	78	52	147	103	144	5	36	158	81	18	103	135	23	36	385	1067	555	765	233	216	60	798

C2.020	C2.021	C2.024	C2.025	C2.026	C2.027	C2.029	C2.030	C2.031	C2.032	C2.033	C2.034	C2.036	C2.037	C2.038	C3.002	C3.003	C3.006	C3.009	C3.010	C3.012	C3.013	C3.014
Oral Ca	Oral Ca	Oral Ca	Oral Ca	Oral Ca	Oral Ca	Oral Ca	Oral Ca	Oral Ca	Oral Ca	Oral Ca	Oral Ca	Oral Ca	Oral Ca	Oral Ca	Oral Ca	Oral Ca	Oral Ca	Oral Ca	Oral Ca	Oral Ca	Oral Ca	Oral Ca
TD	TD	TD	TD	TD	TD	TD	TD	TD	TD	TD	TD	TD	TD	TD	SP	SP	SP	SP	SP	SP	SP	SP
0	0	0	0	0	0	0	0	0	0	0	0	0	0	0	0	0	0	0	0	0	0	0
0	0	0	0	0	0	0	0	0	0	0	0	0	0	0	0	0	0	0	0	0	0	0
0	0	0	0	0	0	0	0	0	0	0	0	0	0	0	0	0	0	0	0	0	0	0
0	0	1	0	0	0	0	0	0	0	1	0	0	0	0	0	3	1	2	0	0	1	0
0	0	0	0	0	0	0	0	0	0	0	0	0	0	0	0	0	0	0	1	0	0	0
0	0	0	0	0	0	0	0	0	0	0	0	0	0	0	0	0	0	0	0	0	0	0
0	0	2	6	0	0	0	0	0	0	0	0	0	0	0	0	0	0	0	0	1	0	0
0	0	0	0	0	0	0	0	0	0	0	0	0	0	0	0	0	0	0	0	0	0	0
0	0	0	0	0	0	0	0	0	0	0	0	0	0	0	0	0	0	0	0	0	0	0
1	0	0	0	0	0	0	0	0	0	1	3	0	2	0	88	0	0	8	0	0	6	1
0	0	0	0	0	0	0	0	1	0	0	0	0	0	0	0	0	0	0	0	0	0	0
0	0	0	0	0	0	0	0	0	0	0	0	0	0	0	0	0	0	3	0	0	0	0
0	0	0	1	0	3	0	0	0	0	0	0	1	0	0	0	1	0	1	5	1	141	0
0	0	0	0	0	0	0	0	0	0	0	0	0	0	0	0	0	0	0	0	0	0	0
0	0	0	0	0	0	0	0	0	0	0	0	0	0	0	0	0	0	0	0	0	0	0
0	0	2	0	0	0	0	0	0	0	0	0	0	0	0	0	0	0	0	0	0	0	0
0	0	0	0	0	0	0	0	0	0	0	0	0	0	0	0	0	0	0	0	0	0	0
0	0	0	0	0	0	0	0	0	0	0	0	0	0	0	0	0	0	0	0	0	0	0
0	0	0	0	0	0	0	0	0	0	0	0	0	0	0	0	0	0	0	0	0	0	0
0	0	0	0	0	0	0	0	0	0	0	0	0	0	0	0	0	0	0	0	0	0	0
211	213	1168	138	136	56	193	199	178	99	246	400	128	971	151	657	162	904	363	168	46	581	412
0	0	0	0	0	1	1	0	0	0	0	3	1	0	0	21	0	0	3	0	0	0	1
0	0	0	0	0	0	0	0	0	5	0	0	0	0	0	0	0	0	0	0	3	0	0
0	0	0	0	0	0	0	0	0	0	0	0	0	0	0	0	0	0	0	0	0	0	0
0	0	0	0	0	0	0	0	0	0	0	0	0	0	0	0	0	0	0	0	0	0	0
0	0	0	0	0	0	0	0	0	0	0	0	0	0	0	0	0	0	0	0	0	0	0
0	0	0	0	0	0	0	0	0	0	0	0	0	0	3	0	0	0	0	0	0	0	0
0	0	0	0	5	2	3	0	0	0	0	0	0	0	0	0	0	0	5	0	0	0	0
0	0	0	0	0	0	0	0	0	0	0	0	0	0	0	0	0	0	0	0	0	0	0
0	0	0	0	0	0	0	0	0	0	0	0	0	0	0	0	0	0	0	0	0	0	0
0	0	0	0	0	0	0	0	0	0	0	0	0	0	0	0	0	0	0	0	0	0	0
0	0	0	0	0	0	0	0	0	0	0	0	0	0	0	0	0	0	0	0	0	0	0
0	0	0	0	0	0	0	0	0	0	0	0	0	0	0	0	0	0	0	0	0	0	0
0	0	0	0	0	0	0	0	0	0	0	0	0	0	0	0	0	0	0	0	0	0	0
0	0	0	0	0	0	0	0	0	0	0	0	0	0	0	0	0	0	0	0	0	0	0
2	4	10	6	6	2	3	1	0	2	3	5	0	2	2	5	194	142	130	39	32	23	827
0	0	1	13	6	0	0	4	0	0	0	0	0	0	0	0	0	10	72	9	0	0	0

C2.020	C2.021	C2.024	C2.025	C2.026	C2.027	C2.029	C2.030	C2.031	C2.032	C2.033	C2.034	C2.036	C2.037	C2.038	C3.002	C3.003	C3.006	C3.009	C3.010	C3.012	C3.013	C3.014
Oral Ca	Oral Ca	Oral Ca	Oral Ca	Oral Ca	Oral Ca	Oral Ca	Oral Ca	Oral Ca	Oral Ca	Oral Ca	Oral Ca	Oral Ca	Oral Ca	Oral Ca	Oral Ca	Oral Ca	Oral Ca	Oral Ca	Oral Ca	Oral Ca	Oral Ca	Oral Ca
TD	TD	TD	TD	TD	TD	TD	TD	TD	TD	TD	TD	TD	TD	TD	SP	SP	SP	SP	SP	SP	SP	SP
0	2	2	3	1	0	0	0	0	0	0	0	0	0	0	0	0	0	12	0	0	0	0
0	0	0	0	0	0	0	0	0	0	0	0	0	0	0	0	0	0	0	0	0	0	0
1	0	0	0	0	0	0	0	0	0	0	0	0	0	0	0	0	0	0	1	0	0	1
0	0	0	1	0	0	0	0	0	0	0	0	0	0	0	0	0	0	0	0	0	0	1
0	0	0	0	0	0	0	0	0	0	0	0	0	0	0	0	0	0	0	0	0	0	0
0	0	0	0	0	0	0	0	0	0	0	0	0	0	0	0	0	0	0	0	0	0	0
0	0	0	0	0	0	0	0	0	0	0	0	0	0	0	0	0	0	0	0	0	0	0
0	0	1	0	0	0	0	0	0	0	0	0	0	0	0	0	0	0	0	0	0	0	0
0	0	0	0	0	0	0	0	0	0	0	0	0	0	0	0	0	0	0	0	0	0	0
0	0	0	0	0	0	0	0	0	0	0	0	0	0	0	0	0	0	0	0	0	0	0
0	0	5	0	5	0	12	0	0	0	0	0	0	2	1	0	0	0	0	1	0	0	0
8	2	75	377	22	30	10	3	0	7	0	1	0	4	2	0	0	0	18	21	49	0	6
0	0	0	0	0	0	0	0	0	0	0	0	0	0	0	0	0	0	0	0	0	0	0
0	0	1	0	0	0	0	0	0	0	0	1	0	0	0	0	0	0	0	0	0	0	0
0	0	0	0	0	0	0	0	0	0	0	0	0	0	0	0	0	0	0	0	0	0	0
0	0	0	0	0	0	0	0	0	0	0	0	0	0	0	0	0	0	0	0	0	0	0
0	0	0	0	0	0	0	0	0	0	0	0	0	0	0	0	0	0	0	0	0	0	0
0	0	0	0	0	0	0	0	0	0	0	0	0	0	0	0	0	0	0	0	0	0	0
5	40	8	14	2	32	1	1	10	19	3	0	8	3	1	5	0	0	2	3	28	1	11
2	83	27	56	16	64	2	21	182	75	25	52	105	0	89	259	185	379	531	59	84	18	245
321	1324	120	304	1022	818	163	850	307	3266	425	1492	2413	19	469	89	93	19	49	28	33	18	245
0	0	0	0	0	0	0	0	0	0	0	0	0	0	0	0	0	0	0	0	0	0	0
0	0	0	0	0	0	0	0	0	0	0	0	0	0	0	0	0	0	0	0	0	0	0
0	0	1	7	2	2	0	0	0	1	0	0	0	0	0	0	0	0	1	0	0	0	1
0	0	0	0	0	0	0	0	0	1	0	0	0	0	0	0	0	0	0	0	0	0	0
0	0	12	0	3	0	1	0	0	0	0	0	0	0	0	0	0	0	1	0	1	0	0
0	0	0	0	0	0	0	0	0	0	0	0	0	0	0	0	0	0	0	0	0	0	0
0	0	0	0	0	3	0	0	0	25	0	0	1	0	0	0	0	0	0	0	0	0	0
13	0	2	37	18	273	2	9	7	56	1	11	108	0	61	18	305	3	29	53	7	1	0
0	0	0	0	0	0	0	0	0	0	0	0	0	0	0	0	0	0	0	0	0	0	0
0	0	0	0	0	0	0	0	0	0	0	0	0	0	0	0	0	0	0	0	0	0	0
0	0	0	0	0	0	0	0	0	0	0	0	0	0	0	0	0	0	0	0	0	0	0
0	0	0	0	0	0	0	0	0	0	0	0	0	0	0	0	0	0	0	0	0	0	0
0	0	17	1	6	17	3	0	1	0	0	0	0	0	1	0	0	0	6	10	26	0	0
0	0	0	0	0	1	0	0	0	0	0	0	0	0	0	0	0	0	0	0	0	0	0
0	0	0	0	0	0	0	0	1	0	0	0	0	0	0	0	0	0	0	0	1	0	0
0	0	0	0	0	0	0	0	0	0	0	0	0	0	0	0	0	0	0	0	0	0	0

C2.020	C2.021	C2.024	C2.025	C2.026	C2.027	C2.029	C2.030	C2.031	C2.032	C2.033	C2.034	C2.036	C2.037	C2.038	C3.002	C3.003	C3.006	C3.009	C3.010	C3.012	C3.013	C3.014
Oral Ca	Oral Ca	Oral Ca	Oral Ca	Oral Ca	Oral Ca	Oral Ca	Oral Ca	Oral Ca	Oral Ca	Oral Ca	Oral Ca	Oral Ca	Oral Ca	Oral Ca	Oral Ca	Oral Ca	Oral Ca	Oral Ca	Oral Ca	Oral Ca	Oral Ca	Oral Ca
TD	TD	TD	TD	TD	TD	TD	TD	TD	TD	TD	TD	TD	TD	TD	SP	SP	SP	SP	SP	SP	SP	SP
0	0	0	0	0	0	0	0	0	0	0	0	0	0	0	0	0	0	0	0	0	0	0
0	0	0	0	0	0	0	0	0	0	0	0	0	0	0	0	0	0	0	0	0	0	0
0	0	1	0	0	0	0	0	0	0	0	0	0	0	0	0	0	0	0	0	0	0	0
0	0	0	0	0	0	0	0	0	0	0	0	0	0	0	0	0	0	0	0	0	0	0
0	0	0	0	0	0	0	0	0	0	0	0	0	0	0	0	0	0	0	0	0	0	0
0	0	0	0	1	0	1	0	0	0	0	0	0	0	0	0	0	0	0	0	0	0	0
0	0	0	0	0	0	0	0	0	0	0	0	0	0	0	0	0	0	0	0	0	0	0
0	0	0	0	0	0	0	0	0	0	0	0	0	0	0	0	0	0	0	0	0	0	0
0	0	0	0	0	0	0	0	0	0	0	0	0	0	0	0	0	0	0	0	0	0	0
0	0	0	0	0	0	0	0	0	0	0	0	0	0	0	0	0	0	0	0	0	0	0
0	0	0	0	0	0	0	0	0	0	0	0	0	0	0	0	0	0	0	0	0	0	0
0	0	1	0	3	3	0	0	0	1	0	0	0	0	7	0	0	0	0	8	6	0	0
0	0	0	0	0	0	0	0	0	0	0	0	0	0	0	0	0	0	0	0	0	0	0
0	0	0	0	0	0	0	0	0	0	0	0	0	0	0	0	0	0	0	0	0	0	0
0	0	1	0	1	0	0	0	2	0	0	1	0	0	3	0	0	0	0	0	0	0	0
0	0	0	0	0	0	0	0	0	0	0	0	0	0	0	0	0	0	0	0	0	0	0
0	0	0	0	0	0	0	0	0	0	0	0	0	0	0	0	0	0	0	0	0	0	2
0	0	0	0	0	0	0	0	0	0	0	0	0	0	0	2	0	0	0	0	0	5	0
16	0	46	12	17	53	2	59	17	15	50	22	11	33	11	713	23	267	369	36	5	380	76
0	0	0	0	0	0	0	0	1	0	0	0	0	0	0	0	0	0	0	0	0	28	0
1	0	2	0	2	1	0	0	0	0	0	2	0	4	0	66	1	12	8	3	0	51	0
1	0	0	0	0	0	0	0	0	0	0	0	0	0	0	0	0	0	0	0	0	0	0
0	0	0	0	0	0	0	0	0	0	0	0	0	0	0	0	0	0	0	0	0	0	0
0	0	0	4	0	1	0	1	8	0	0	2	0	6	0	0	0	6	187	1	0	0	0
0	1	20	8	21	4	2	1	2	4	2	3	1	7	0	45	1	1	3	14	4	14	6
0	0	0	0	5	3	0	0	0	0	0	0	0	0	0	0	0	0	0	1	1	0	0
0	0	0	0	0	0	0	0	0	0	0	0	0	0	0	0	0	0	0	0	0	0	0
0	0	0	0	0	0	0	0	0	0	0	0	0	0	0	0	0	0	0	0	0	0	0
15	15	10	1	5	13	0	5	8	0	13	27	7	6	8	6	0	36	1	5	0	21	1
2	1	4	6	5	2	6	5	0	5	3	2	0	6	4	101	0	13	2	2	1	157	256
0	0	0	0	0	0	0	0	0	0	0	0	0	0	0	0	0	0	0	0	0	0	0
0	0	0	0	0	0	0	0	0	0	0	0	0	0	0	0	0	0	0	0	0	0	0

C3.016	C3.017	C3.018	C3.019	C3.020	C3.021	C3.024	C3.025	C3.026	C3.027	C3.030	C3.031	C3.032	C3.033	C3.034	C3.036	C3.037	C3.038	E1.006	E1.010	E1.012	E1.013	E1.014
Oral Ca	Oral Ca	Oral Ca	Oral Ca	Oral Ca	Oral Ca	Oral Ca	Oral Ca	Oral Ca	Oral Ca	Oral Ca	Oral Ca	Oral Ca	Oral Ca	Oral Ca	Oral Ca	Oral Ca	Oral Ca	Esophag	Esophag	Esophag	Esophag	Esophag
SP	SP	SP	SP	SP	SP	SP	SP	SP	SP	SP	SP	SP	SP	SP	SP	SP	SP	UE	UE	UE	UE	UE
0	0	0	0	0	0	0	0	0	0	0	0	0	0	0	0	0	0	0	0	0	0	0
20447	15960	21661	20771	7787	19656	10368	11742	15860	13916	23153	19434	9718	20086	16190	19806	17455	19117	11950	12271	5598	20951	11346
1	0	2	5	7	0	22	97	23	96	3	23	2	2	9	1	29	246	0	26	32	5	9
0	0	0	0	0	0	0	0	0	0	0	0	0	0	0	0	0	0	0	0	1	0	0
0	0	0	0	0	0	1	0	1	0	0	0	0	0	0	0	0	0	0	14	17	0	3
1916	655	910	1056	2273	1478	116	209	137	121	771	243	243	788	731	573	844	570	83	572	88	445	354
0	0	0	1	0	0	0	0	0	0	0	0	0	0	0	0	0	0	0	2	11	0	0
3872	3846	6182	2987	2159	7526	4805	5358	10251	6642	8136	5645	3420	9087	5208	5674	6278	7489	1189	176	430	3034	1158
0	0	0	0	0	0	0	0	0	0	0	0	0	0	0	0	0	0	0	0	0	0	0
0	0	0	0	0	0	0	0	0	0	0	0	0	0	0	0	0	0	0	0	0	0	0
0	0	0	0	0	0	0	0	0	0	0	0	0	0	13	0	0	0	0	0	0	0	0
0	0	0	0	0	1	0	0	0	0	0	4	0	0	1	0	1	0	0	0	0	0	0
8	1	0	0	3	0	0	0	0	0	0	4	0	0	0	0	4	0	0	0	2	0	0
0	0	34	0	0	12	0	0	0	0	0	0	0	0	8	0	4	0	0	0	0	0	0
0	0	0	0	0	0	0	0	0	0	0	0	0	0	0	0	0	0	0	0	0	0	0
2158	502	2126	8928	231	772	1133	952	1596	383	2361	3580	1021	2521	1876	1584	1891	1269	4185	998	700	14616	4286
1271	1810	601	263	251	1239	676	761	1484	1690	2759	962	833	1257	420	832	1030	1870	137	22	15	498	202
0	0	0	0	0	0	0	0	0	0	0	0	0	0	0	0	0	0	0	0	0	0	0
141	117	54	6	19	0	86	563	25	148	112	228	25	37	58	164	0	66	0	0	1	0	0
0	0	0	0	0	0	0	0	0	0	0	0	0	0	0	0	0	0	0	0	0	0	0
0	0	0	0	0	0	0	0	0	0	0	0	0	0	0	0	0	0	0	0	0	0	0
10903	8823	10692	6566	2092	8209	2998	3494	1390	3116	8003	5316	3809	5639	6513	10157	3075	6739	6287	10451	4277	1976	5171
13	18	481	4	54	84	85	140	305	59	85	1572	105	127	366	208	570	270	1	1	0	182	0
30	15	27	9	9	27	23	56	21	48	51	23	17	58	21	47	26	77	0	1	1	23	15
1	0	37	0	5	15	2	5	3	21	1	61	8	3	77	14	22	6	0	0	0	12	0
3	0	185	7	0	0	8	14	13	7	16	442	36	4	28	20	134	5	0	4	4	23	9
0	0	0	0	0	0	0	0	0	0	0	0	0	0	0	0	0	0	0	0	0	0	0
0	0	0	0	0	0	0	0	0	0	0	0	0	0	0	0	0	0	0	0	0	0	0
130	173	310	937	684	256	413	93	611	1580	854	1287	199	563	857	532	3547	510	68	4	18	117	102
0	0	0	2	0	0	0	0	0	0	0	0	0	0	0	0	0	0	0	0	0	20	37
0	0	0	0	0	0	0	0	0	0	0	0	0	0	0	0	0	0	0	0	1	0	0
0	0	20	0	0	37	0	0	0	5	1	44	0	0	4	0	0	0	0	0	0	0	0
0	0	0	0	0	0	0	0	0	0	0	0	0	0	0	0	0	0	0	0	1	0	0
0	0	0	0	0	0	0	0	0	0	0	0	0	0	0	0	0	0	0	0	0	0	0
0	0	0	0	0	0	0	0	0	0	0	0	0	0	0	0	0	0	0	0	0	0	3
0	0	0	0	0	0	0	0	0	0	0	0	0	0	0	0	0	0	0	1	0	0	0

C3.016	C3.017	C3.018	C3.019	C3.020	C3.021	C3.024	C3.025	C3.026	C3.027	C3.030	C3.031	C3.032	C3.033	C3.034	C3.036	C3.037	C3.038	E1.006	E1.010	E1.012	E1.013	E1.014
Oral Ca	Oral Ca	Oral Ca	Oral Ca	Oral Ca	Oral Ca	Oral Ca	Oral Ca	Oral Ca	Oral Ca	Oral Ca	Oral Ca	Oral Ca	Oral Ca	Oral Ca	Oral Ca	Oral Ca	Oral Ca	Esophag	Esophag	Esophag	Esophag	Esophag
SP	SP	SP	SP	SP	SP	SP	SP	SP	SP	SP	SP	SP	SP	SP	SP	SP	SP	UE	UE	UE	UE	UE
0	0	0	0	0	0	0	0	0	0	0	0	0	0	0	0	0	0	0	0	0	0	0
0	0	0	0	0	0	1	0	1	0	0	0	0	0	0	0	0	0	0	13	17	0	0
0	0	0	0	0	0	0	0	0	0	0	0	0	0	0	0	0	0	0	0	0	0	0
0	0	0	0	0	0	0	1	0	0	0	0	0	0	0	0	0	0	0	0	0	0	0
1916	655	905	1036	2272	1478	116	201	135	118	768	236	242	774	727	571	841	569	83	553	86	428	350
0	0	5	20	1	0	0	7	2	3	3	7	1	14	4	2	3	1	0	19	2	15	4
0	0	0	0	0	0	0	0	0	0	0	0	0	0	0	0	0	0	0	0	0	0	0
0	0	0	0	0	0	0	0	0	0	0	0	0	0	0	0	0	0	0	0	0	0	0
0	0	0	0	0	0	0	0	0	0	0	0	0	0	0	0	0	0	0	0	0	2	0
0	0	0	1	0	0	0	0	0	0	0	0	0	0	0	0	0	0	0	2	11	0	0
0	0	0	0	0	0	0	0	0	0	0	0	0	0	0	0	0	0	0	0	0	0	0
0	0	0	0	0	0	1	1	0	0	1	1	0	1	0	0	0	0	49	15	14	18	14
1417	658	3350	2854	468	1570	2855	977	8336	2157	4638	3454	1995	6230	4343	3523	2158	3928	667	158	378	2572	1087
7	0	0	0	0	0	0	0	0	0	0	0	0	0	0	0	0	0	0	0	2	2	0
2441	3188	2832	98	1691	5956	1949	4379	1915	4485	3497	2184	1425	2856	865	2151	4120	3561	211	3	35	438	54
7	0	0	35	0	0	0	1	0	0	0	6	0	0	0	0	0	0	262	0	1	4	3
0	0	0	0	0	0	0	0	0	0	0	0	0	0	0	0	0	0	0	0	0	0	0
0	0	0	0	0	0	0	0	0	0	0	0	0	0	0	0	0	0	0	0	0	0	0
0	0	0	0	0	0	0	0	0	0	0	0	0	0	0	0	0	0	0	0	0	0	0
0	0	0	0	0	0	0	0	0	0	0	0	0	0	13	0	0	0	0	0	0	0	0
0	0	0	0	0	1	0	0	0	0	0	4	0	0	1	0	1	0	0	0	0	0	0
0	0	0	0	0	0	0	0	0	0	0	0	0	0	0	0	0	0	0	0	0	0	0
0	0	0	0	0	0	0	0	0	0	0	0	0	0	0	0	0	0	0	0	0	0	0
0	0	0	0	0	0	0	0	0	0	0	0	0	0	0	0	0	0	0	0	0	0	0
0	0	0	0	0	0	0	0	0	0	0	0	0	0	0	0	0	0	0	0	0	0	0
0	0	0	0	0	0	0	0	0	0	0	0	0	0	0	0	0	0	0	0	0	0	0
0	1	0	0	0	0	0	0	0	0	0	1	0	0	0	0	0	0	0	0	0	0	0
8	0	0	0	3	0	0	0	0	0	0	3	0	0	0	0	4	0	0	0	2	0	0
0	0	0	0	0	0	0	0	0	0	0	0	0	0	0	0	0	0	0	0	0	0	0
0	0	0	0	0	0	0	0	0	0	0	0	0	0	0	0	0	0	0	0	0	0	0
0	0	22	0	0	0	0	0	0	0	0	0	0	0	8	0	0	0	0	0	0	0	0
0	0	12	0	0	12	0	0	0	0	0	0	0	0	0	0	4	0	0	0	0	0	0
0	0	0	0	0	0	0	0	0	0	0	0	0	0	0	0	0	0	0	0	0	0	0
1490	242	622	8349	94	363	439	482	641	112	742	1112	223	666	427	799	456	268	3805	544	428	13110	3049
667	260	1488	551	137	405	687	466	944	269	1607	2456	792	1817	1432	778	1401	999	376	410	243	1477	1119
1	0	16	28	0	4	7	4	11	2	12	12	6	38	17	7	34	2	4	44	29	29	118
1271	1810	601	263	251	1239	676	761	1484	1690	2759	962	833	1257	420	832	1030	1870	137	22	15	498	202

C3.016	C3.017	C3.018	C3.019	C3.020	C3.021	C3.024	C3.025	C3.026	C3.027	C3.030	C3.031	C3.032	C3.033	C3.034	C3.036	C3.037	C3.038	E1.006	E1.010	E1.012	E1.013	E1.014
Oral Ca	Oral Ca	Oral Ca	Oral Ca	Oral Ca	Oral Ca	Oral Ca	Oral Ca	Oral Ca	Oral Ca	Oral Ca	Oral Ca	Oral Ca	Oral Ca	Oral Ca	Oral Ca	Oral Ca	Oral Ca	Esophag	Esophag	Esophag	Esophag	Esophag
SP	SP	SP	SP	SP	SP	SP	SP	SP	SP	SP	SP	SP	SP	SP	SP	SP	SP	UE	UE	UE	UE	UE
0	0	0	0	0	0	0	0	0	0	0	0	0	0	0	0	0	0	0	0	0	0	0
141	117	54	6	19	0	86	563	25	148	112	228	25	37	58	164	0	66	0	0	1	0	0
0	0	0	0	0	0	0	0	0	0	0	0	0	0	0	0	0	0	0	0	0	0	0
0	0	0	0	0	0	0	0	0	0	0	0	0	0	0	0	0	0	0	0	0	0	0
0	0	0	0	0	0	0	0	0	0	0	0	0	0	0	0	0	0	0	0	0	0	0
0	0	0	0	0	0	0	0	0	0	0	0	0	0	0	0	0	0	0	0	0	0	0
40	1	1	42	13	2	292	9	144	1	3	11	0	1	1	0	2	0	580	9080	3154	478	1144
8962	8109	8117	4673	1620	7029	1860	2376	546	2409	5871	4459	2755	3856	4351	5883	2681	4424	1147	727	466	195	1126
0	0	97	0	31	30	1	2	9	15	14	91	6	1	151	29	0	1	0	6	3	46	22
132	296	343	112	91	415	412	275	304	171	510	271	137	347	643	499	375	213	1990	0	47	176	91
1769	417	2134	1739	336	733	433	831	387	520	1605	484	911	1434	1367	3746	17	2100	2570	638	607	1081	2788
0	0	0	0	0	0	0	0	0	0	0	0	0	0	0	0	0	0	0	0	0	0	0
0	0	0	0	0	0	0	0	0	0	0	0	0	0	6	0	0	0	0	0	0	0	0
13	18	481	4	54	84	85	140	305	59	85	1572	105	127	360	208	570	270	1	1	0	182	0
1	0	37	0	5	15	2	5	3	21	1	61	8	3	77	14	22	6	0	0	0	12	0
0	0	0	0	0	0	0	0	0	0	0	0	0	0	0	0	0	0	0	0	0	0	0
3	0	119	7	0	0	7	14	12	6	16	379	31	4	23	20	134	5	0	4	4	23	9
0	0	66	0	0	0	1	0	1	1	0	63	5	0	5	0	0	0	0	0	0	0	0
0	0	0	0	0	0	0	0	0	0	0	0	0	0	0	0	0	0	0	0	0	0	0
0	0	0	0	0	0	0	0	0	0	0	0	0	0	0	0	0	0	0	0	0	0	0
0	0	0	0	0	0	0	0	0	0	0	0	0	0	0	0	0	0	0	0	0	0	0
0	0	0	0	0	0	0	0	0	0	0	0	0	0	0	0	0	0	0	0	0	0	0
0	0	0	0	0	0	0	0	0	0	0	0	0	0	0	0	0	0	0	0	0	0	0
130	173	310	937	684	256	413	93	611	1580	854	1287	199	563	857	532	3547	510	68	4	18	117	102
0	0	0	0	0	0	0	0	0	0	0	0	0	0	0	0	0	0	0	0	0	0	0
0	0	0	2	0	0	0	0	0	0	0	0	0	0	0	0	0	0	0	0	0	20	37
0	0	20	0	0	37	0	0	0	5	1	44	0	0	4	0	0	0	0	0	0	0	0
0	0	0	0	0	0	0	0	0	0	0	0	0	0	0	0	0	0	0	0	1	0	0
0	0	0	0	0	0	0	0	0	0	0	0	0	0	0	0	0	0	0	0	0	0	0
0	0	0	0	0	0	0	0	0	0	0	0	0	0	0	0	0	0	0	0	0	0	0
0	0	0	0	0	0	0	0	0	0	0	0	0	0	0	0	0	0	0	0	0	0	3
0	0	0	0	0	0	0	0	0	0	0	0	0	0	0	0	0	0	0	1	0	0	0
0	0	0	0	0	0	0	0	0	0	0	0	0	0	0	0	0	0	0	0	0	0	0
0	0	0	0	0	0	1	0	1	0	0	0	0	0	0	0	0	0	0	13	17	0	0
0	0	0	0	0	0	0	0	0	0	0	0	0	0	0	0	0	0	0	0	0	0	0
0	0	0	0	0	0	0	1	0	0	0	0	0	0	0	0	0	0	0	0	0	0	0
1915	655	905	1008	2271	1478	111	157	87	107	768	223	242	774	727	570	841	569	83	32	46	415	350

C3.016	C3.017	C3.018	C3.019	C3.020	C3.021	C3.024	C3.025	C3.026	C3.027	C3.030	C3.031	C3.032	C3.033	C3.034	C3.036	C3.037	C3.038	E1.006	E1.010	E1.012	E1.013	E1.014
Oral Ca	Oral Ca	Oral Ca	Oral Ca	Oral Ca	Oral Ca	Oral Ca	Oral Ca	Oral Ca	Oral Ca	Oral Ca	Oral Ca	Oral Ca	Oral Ca	Oral Ca	Oral Ca	Oral Ca	Oral Ca	Esophag	Esophag	Esophag	Esophag	Esophag
SP	SP	SP	SP	SP	SP	SP	SP	SP	SP	SP	SP	SP	SP	SP	SP	SP	SP	UE	UE	UE	UE	UE
1	0	0	28	1	0	5	44	48	11	0	13	0	0	0	1	0	0	0	521	40	13	0
0	0	0	0	0	0	0	0	0	0	0	0	0	0	0	0	0	0	0	0	0	0	0
0	0	5	20	1	0	0	7	2	3	3	7	1	14	4	2	3	1	0	19	2	15	4
0	0	0	0	0	0	0	0	0	0	0	0	0	0	0	0	0	0	0	0	0	0	0
0	0	0	0	0	0	0	0	0	0	0	0	0	0	0	0	0	0	0	0	0	0	0
0	0	0	0	0	0	0	0	0	0	0	0	0	0	0	0	0	0	0	0	0	2	0
0	0	0	0	0	0	0	0	0	0	0	0	0	0	0	0	0	0	0	0	0	0	0
0	0	0	1	0	0	0	0	0	0	0	0	0	0	0	0	0	0	0	2	11	0	0
0	0	0	0	0	0	0	0	0	0	0	0	0	0	0	0	0	0	0	0	0	0	0
0	0	0	0	0	0	1	1	0	0	1	1	0	1	0	0	0	0	49	15	14	18	14
1417	658	3350	2854	468	1570	2855	977	8336	2157	4638	3454	1995	6230	4343	3523	2158	3928	667	158	378	2572	1087
7	0	0	0	0	0	0	0	0	0	0	0	0	0	0	0	0	0	0	0	2	2	0
2441	3188	2832	98	1691	5956	1949	4379	1915	4485	3497	2184	1425	2856	865	2151	4120	3561	211	3	35	438	54
7	0	0	35	0	0	0	1	0	0	0	6	0	0	0	0	0	0	262	0	1	4	3
0	0	0	0	0	0	0	0	0	0	0	0	0	0	0	0	0	0	0	0	0	0	0
0	0	0	0	0	0	0	0	0	0	0	0	0	0	0	0	0	0	0	0	0	0	0
0	0	0	0	0	1	0	0	0	0	0	4	0	0	1	0	1	0	0	0	0	0	0
0	0	0	0	0	0	0	0	0	0	0	0	0	0	0	0	0	0	0	0	0	0	0
0	0	0	0	0	0	0	0	0	0	0	0	0	0	0	0	0	0	0	0	0	0	0
0	0	0	0	0	0	0	0	0	0	0	0	0	0	0	0	0	0	0	0	0	0	0
0	0	0	0	0	0	0	0	0	0	0	0	0	0	0	0	0	0	0	0	0	0	0
0	1	0	0	0	0	0	0	0	0	0	1	0	0	0	0	0	0	0	0	0	0	0
0	0	0	0	0	0	0	0	0	0	0	0	0	0	0	0	0	0	0	0	0	0	0
0	0	0	0	0	0	0	0	0	0	0	0	0	0	0	0	0	0	0	0	0	0	0
0	0	0	0	0	0	0	0	0	0	0	0	0	0	0	0	0	0	0	0	0	0	0
8	0	0	0	3	0	0	0	0	0	0	3	0	0	0	0	4	0	0	0	2	0	0
0	0	0	0	0	0	0	0	0	0	0	0	0	0	0	0	0	0	0	0	0	0	0
0	0	0	0	0	0	0	0	0	0	0	0	0	0	0	0	0	0	0	0	0	0	0
0	0	22	0	0	0	0	0	0	0	0	0	0	0	8	0	0	0	0	0	0	0	0
0	0	0	0	0	0	0	0	0	0	0	0	0	0	0	0	0	0	0	0	0	0	0
134	4	1	19	0	0	0	4	10	4	0	15	0	0	0	0	0	0	79	78	34	34	122
9	10	18	441	1	31	34	26	31	13	30	27	4	44	31	16	14	11	220	24	38	659	229
1347	228	603	7889	93	332	405	452	600	95	712	1070	219	622	396	783	442	257	3506	442	355	12417	2685
0	0	0	0	0	0	0	0	0	0	0	0	0	0	0	0	0	0	0	0	1	0	13
667	260	1488	551	137	405	687	466	944	269	1607	2456	792	1817	1432	778	1401	999	376	410	243	1477	1119
0	0	0	0	0	0	0	0	0	0	0	0	0	0	0	0	0	0	0	0	0	0	0
0	0	0	0	0	0	0	0	0	0	0	0	0	0	0	0	0	0	0	0	0	0	0

C3.016	C3.017	C3.018	C3.019	C3.020	C3.021	C3.024	C3.025	C3.026	C3.027	C3.030	C3.031	C3.032	C3.033	C3.034	C3.036	C3.037	C3.038	E1.006	E1.010	E1.012	E1.013	E1.014
Oral Ca	Oral Ca	Oral Ca	Oral Ca	Oral Ca	Oral Ca	Oral Ca	Oral Ca	Oral Ca	Oral Ca	Oral Ca	Oral Ca	Oral Ca	Oral Ca	Oral Ca	Oral Ca	Oral Ca	Oral Ca	Esophag	Esophag	Esophag	Esophag	Esophag
SP	SP	SP	SP	SP	SP	SP	SP	SP	SP	SP	SP	SP	SP	SP	SP	SP	SP	UE	UE	UE	UE	UE
1	0	16	28	0	4	7	4	11	2	12	12	6	38	17	7	34	2	4	44	29	29	118
1271	1810	601	263	251	1239	676	761	1484	1690	2759	962	833	1257	420	832	1030	1870	137	22	15	498	202
0	0	0	0	0	0	0	0	0	0	0	0	0	0	0	0	0	0	0	0	0	0	0
0	0	0	0	0	0	0	0	0	0	0	0	0	0	0	0	0	0	0	0	0	0	0
0	0	0	0	0	0	0	0	0	0	0	0	0	0	0	0	0	0	0	0	0	0	0
0	0	0	0	0	0	0	0	0	0	0	0	0	0	0	0	0	0	0	0	0	0	0
0	0	0	0	0	0	0	0	0	0	0	0	0	0	0	0	0	0	0	0	0	0	0
0	0	0	0	0	0	1	0	0	0	0	0	0	0	0	0	0	0	51	0	0	0	0
24	1	0	13	8	1	3	2	4	0	2	7	0	1	1	0	1	0	238	291	118	282	478
0	0	0	0	0	0	0	0	0	0	0	0	0	0	0	0	0	0	0	0	0	0	8
0	0	0	0	0	0	0	0	0	0	0	0	0	0	0	0	0	0	0	0	0	0	0
7	0	1	20	4	1	1	2	5	0	0	3	0	0	0	0	0	0	195	753	193	37	298
0	0	0	0	0	0	0	0	0	0	0	0	0	0	0	0	0	0	0	0	14	0	23
2	0	0	0	0	0	0	0	1	1	0	0	0	0	0	0	0	0	7	86	14	5	128
1	0	0	0	0	0	0	0	0	0	0	0	0	0	0	0	0	0	0	0	0	3	0
6	0	0	9	1	0	287	5	134	0	1	1	0	0	0	0	1	0	89	7950	2815	151	209
0	0	0	0	0	0	0	0	0	0	0	0	0	0	0	0	0	0	0	0	0	0	0
2095	1690	1270	48	1401	2633	313	569	98	702	924	637	85	1223	1523	1368	1170	702	157	673	219	47	245
0	0	0	1	0	0	0	0	0	0	0	0	0	0	0	0	0	0	0	1	0	0	0
0	0	0	0	0	0	0	0	0	0	0	0	0	0	0	0	0	0	0	0	0	0	0
6867	6418	6846	4624	216	4390	1547	1805	446	1705	4913	3821	2662	2632	2824	4485	1511	3720	990	53	240	133	879
0	1	1	0	3	6	0	2	1	2	34	1	8	1	4	30	0	2	0	0	3	15	2
0	0	0	0	0	0	0	0	0	0	0	0	0	0	0	0	0	0	0	0	0	0	0
0	0	0	0	0	0	0	0	0	0	0	0	0	0	0	0	0	0	0	0	0	0	0
0	0	0	0	0	0	0	0	0	0	0	0	0	0	0	0	0	0	0	1	0	0	0
0	0	5	0	30	20	1	0	1	0	0	15	6	1	53	29	0	1	0	0	0	0	0
0	0	92	0	1	10	0	2	8	5	0	67	0	0	35	0	0	0	0	4	2	43	16
0	0	0	0	0	0	0	0	0	0	0	0	0	0	0	0	0	0	0	0	0	0	0
0	0	0	0	0	0	0	0	0	10	14	9	0	0	42	0	0	0	0	0	0	0	0
0	0	0	0	0	0	0	0	0	0	0	0	0	0	0	0	0	0	0	0	0	0	0
0	0	0	0	0	0	0	0	0	0	0	0	0	0	21	0	0	0	0	1	1	0	6
0	0	0	0	0	0	0	0	0	0	0	0	0	0	0	0	0	0	0	0	0	0	0
0	0	0	0	0	0	0	0	0	0	0	0	0	0	0	0	0	0	0	0	0	3	0
132	296	343	112	91	415	412	275	304	171	510	271	137	347	643	499	375	213	1990	0	47	176	91
0	0	0	0	0	0	3	0	0	0	0	0	0	0	0	0	0	0	0	0	0	0	28
0	0	0	0	0	0	0	0	0	0	0	0	0	0	0	0	0	0	0	0	0	0	0
450	170	70	1	309	516	112	354	54	127	372	18	51	594	166	96	2	224	0	0	2	0	0

C3.016	C3.017	C3.018	C3.019	C3.020	C3.021	C3.024	C3.025	C3.026	C3.027	C3.030	C3.031	C3.032	C3.033	C3.034	C3.036	C3.037	C3.038	E1.006	E1.010	E1.012	E1.013	E1.014
Oral Ca	Oral Ca	Oral Ca	Oral Ca	Oral Ca	Oral Ca	Oral Ca	Oral Ca	Oral Ca	Oral Ca	Oral Ca	Oral Ca	Oral Ca	Oral Ca	Oral Ca	Oral Ca	Oral Ca	Oral Ca	Esophag	Esophag	Esophag	Esophag	Esophag
SP	SP	SP	SP	SP	SP	SP	SP	SP	SP	SP	SP	SP	SP	SP	SP	SP	SP	UE	UE	UE	UE	UE
28	0	0	9	2	1	20	8	51	16	0	0	0	0	0	0	0	0	193	321	214	103	1399
0	0	0	0	0	0	0	0	0	0	0	0	0	0	0	0	0	0	0	0	0	0	0
0	0	0	0	0	0	0	0	0	0	0	0	0	0	0	0	0	0	0	0	0	0	0
898	247	578	1606	22	215	280	176	264	373	1077	430	422	835	1194	2974	15	1331	1677	262	34	902	1183
393	0	1486	44	3	1	16	293	16	4	156	8	438	5	7	676	0	542	364	40	309	11	154
0	0	0	0	0	0	0	0	0	0	0	0	0	0	0	0	0	0	0	0	0	0	0
0	0	0	0	0	0	0	0	0	0	0	0	0	0	0	0	0	0	0	0	0	0	0
0	0	0	79	0	0	2	0	2	0	0	28	0	0	0	0	0	0	336	15	48	65	24
0	0	0	0	0	0	0	0	0	0	0	0	0	0	0	0	0	0	0	0	0	0	0
0	0	0	0	0	0	0	0	0	0	0	0	0	0	6	0	0	0	0	0	0	0	0
0	0	14	0	0	2	0	0	0	0	0	5	1	0	2	0	14	0	0	0	0	0	0
13	18	467	4	54	82	85	140	305	59	85	1567	104	127	356	208	556	270	1	1	0	182	0
1	0	37	0	5	15	2	5	3	21	1	61	8	3	77	14	22	6	0	0	0	12	0
0	0	0	0	0	0	0	7	0	0	15	271	28	0	14	0	82	1	0	0	0	0	0
3	0	118	0	0	0	4	7	8	1	0	108	2	4	3	20	52	4	0	4	4	23	9
0	0	1	7	0	0	3	0	4	5	1	0	1	0	6	0	0	0	0	0	0	0	0
0	0	66	0	0	0	1	0	1	1	0	63	5	0	5	0	0	0	0	0	0	0	0
0	0	0	0	0	0	0	0	0	0	0	0	0	0	0	0	0	0	0	0	0	0	0
0	0	0	0	0	0	0	0	0	0	0	0	0	0	0	0	0	0	0	0	0	0	0
0	0	2	448	1	64	14	6	5	54	29	8	10	18	46	276	7	91	0	0	4	23	0
0	0	0	0	0	0	0	0	0	0	0	0	0	0	0	0	0	0	44	0	0	0	0
0	0	0	0	0	0	13	4	4	72	76	38	5	11	6	3	1	13	0	2	3	6	0
0	0	0	0	0	0	0	0	0	0	0	0	0	0	0	0	0	0	0	0	0	0	0
0	0	0	2	0	0	0	0	0	0	0	0	0	0	0	0	0	0	0	0	0	20	37
0	0	20	0	0	37	0	0	0	5	1	44	0	0	4	0	0	0	0	0	0	0	0
0	0	0	0	0	0	0	0	0	0	0	0	0	0	0	0	0	0	0	0	1	0	0
0	0	0	0	0	0	0	0	0	0	0	0	0	0	0	0	0	0	0	0	0	0	0
0	0	0	0	0	0	0	0	0	0	0	0	0	0	0	0	0	0	0	0	0	0	0
0	0	0	0	0	0	0	0	0	0	0	0	0	0	0	0	0	0	0	0	0	0	0
0	0	0	0	0	0	0	0	0	0	0	0	0	0	0	0	0	0	0	0	0	0	0
0	0	0	0	0	0	0	0	0	0	0	0	0	0	0	0	0	0	0	1	0	0	0
0	0	0	0	0	0	0	0	0	0	0	0	0	0	0	0	0	0	0	0	0	0	0
0	0	0	0	0	0	0	1	0	0	0	0	0	0	0	0	0	0	0	0	0	0	0
0	0	0	0	0	0	0	0	0	0	0	0	0	0	0	0	0	0	0	0	0	0	0
580	278	395	772	1223	499	51	58	32	36	317	84	92	291	370	195	421	133	3	10	10	171	157
0	0	0	0	0	0	0	0	0	0	0	0	0	0	0	0	0	0	0	0	0	0	0
0	0	0	0	0	0	0	0	0	0	0	0	0	0	0	0	0	0	0	0	1	0	4

C3.016	C3.017	C3.018	C3.019	C3.020	C3.021	C3.024	C3.025	C3.026	C3.027	C3.030	C3.031	C3.032	C3.033	C3.034	C3.036	C3.037	C3.038	E1.006	E1.010	E1.012	E1.013	E1.014
Oral Ca	Oral Ca	Oral Ca	Oral Ca	Oral Ca	Oral Ca	Oral Ca	Oral Ca	Oral Ca	Oral Ca	Oral Ca	Oral Ca	Oral Ca	Oral Ca	Oral Ca	Oral Ca	Oral Ca	Oral Ca	Esophag	Esophag	Esophag	Esophag	Esophag
SP	SP	SP	SP	SP	SP	SP	SP	SP	SP	SP	SP	SP	SP	SP	SP	SP	SP	UE	UE	UE	UE	UE
632	292	137	21	58	531	46	51	34	46	185	94	98	331	157	141	50	259	7	5	7	23	10
0	0	0	0	0	0	0	0	0	0	0	0	0	0	0	0	0	0	0	0	0	0	0
0	0	0	0	0	0	0	0	0	0	0	0	0	0	0	0	0	0	0	0	0	0	0
0	0	0	0	0	0	0	0	0	0	0	0	0	0	0	0	0	0	0	0	0	0	0
0	0	0	0	0	0	0	0	0	0	0	0	0	0	0	0	0	0	0	0	0	0	0
0	0	0	0	0	0	0	0	0	0	0	0	0	0	0	0	0	0	0	0	1	0	6
0	0	0	0	0	0	0	0	0	0	0	0	0	0	0	0	0	0	0	0	0	0	0
0	0	0	0	0	0	0	0	0	0	0	0	0	0	0	0	0	0	0	0	0	0	0
0	0	0	2	0	0	0	0	0	0	0	0	0	0	0	0	0	0	0	0	2	0	9
0	0	0	0	0	0	0	0	0	0	0	0	0	0	0	0	0	0	0	0	0	0	0
0	0	0	0	0	0	0	0	0	0	0	0	0	0	0	0	0	0	0	0	0	0	0
4	0	0	0	0	0	0	0	0	0	0	0	0	0	0	0	0	0	6	0	1	0	23
134	41	18	161	80	43	7	3	3	1	32	12	18	12	31	4	95	9	11	5	7	161	25
0	0	0	0	0	0	0	0	0	0	0	0	0	0	0	0	0	0	0	0	0	0	0
0	0	0	0	0	0	0	0	0	0	0	0	0	0	0	0	0	0	0	0	0	0	0
0	0	0	0	0	0	0	0	0	0	0	0	0	0	0	0	0	0	0	6	0	0	2
0	0	0	0	0	1	0	8	1	0	3	0	0	0	13	0	0	0	0	0	2	0	4
0	0	0	0	0	0	0	0	0	0	0	0	0	0	0	0	0	0	0	0	0	0	0
0	0	0	0	0	0	0	0	0	0	0	0	0	0	0	0	0	0	0	0	0	0	0
564	44	355	52	910	404	7	37	17	24	231	33	34	140	156	230	275	168	3	5	15	47	84
0	0	0	0	0	0	0	0	0	0	0	0	0	0	0	0	0	0	0	0	0	0	0
0	0	0	0	0	0	0	0	0	0	0	0	0	0	0	0	0	0	0	0	0	0	0
0	0	0	0	0	0	0	0	0	0	0	0	0	0	0	0	0	0	0	0	0	0	0
1	0	0	0	0	0	0	0	0	0	0	0	0	0	0	0	0	0	53	1	0	13	26
0	0	0	0	0	0	0	0	0	0	0	0	0	0	0	0	0	0	0	0	0	0	0
0	0	0	0	0	0	0	0	0	0	0	0	0	0	0	0	0	0	0	0	0	0	0
1	0	0	28	1	0	5	44	48	11	0	13	0	0	0	1	0	0	0	521	40	13	0
0	0	5	20	1	0	0	7	2	3	3	7	1	14	4	2	3	1	0	19	2	15	4
0	0	0	0	0	0	0	0	0	0	0	0	0	0	0	0	0	0	0	0	0	0	0
0	0	0	0	0	0	0	0	0	0	0	0	0	0	0	0	0	0	0	0	0	0	0
0	0	0	0	0	0	0	0	0	0	0	0	0	0	0	0	0	0	0	0	0	0	0
0	0	0	0	0	0	0	0	0	0	0	0	0	0	0	0	0	0	0	0	0	0	0
0	0	0	1	0	0	0	0	0	0	0	0	0	0	0	0	0	0	0	2	11	0	0
0	0	0	0	0	0	0	0	0	0	0	0	0	0	0	0	0	0	0	0	0	0	0
0	0	0	0	0	0	1	1	0	0	1	1	0	1	0	0	0	0	49	15	14	18	14
0	0	0	0	0	0	0	0	0	0	0	0	0	0	0	0	0	0	0	0	0	0	0
0	0	0	0	0	0	0	0	0	0	0	0	0	0	0	0	0	0	0	0	0	0	0

C3.016	C3.017	C3.018	C3.019	C3.020	C3.021	C3.024	C3.025	C3.026	C3.027	C3.030	C3.031	C3.032	C3.033	C3.034	C3.036	C3.037	C3.038	E1.006	E1.010	E1.012	E1.013	E1.014
Oral Ca	Oral Ca	Oral Ca	Oral Ca	Oral Ca	Oral Ca	Oral Ca	Oral Ca	Oral Ca	Oral Ca	Oral Ca	Oral Ca	Oral Ca	Oral Ca	Oral Ca	Oral Ca	Oral Ca	Oral Ca	Esophag	Esophag	Esophag	Esophag	Esophag
SP	SP	SP	SP	SP	SP	SP	SP	SP	SP	SP	SP	SP	SP	SP	SP	SP	SP	UE	UE	UE	UE	UE
0	0	0	0	0	0	0	0	0	0	0	0	0	0	59	0	0	0	0	1	0	0	1
41	24	278	739	25	34	119	101	639	270	316	567	269	918	399	382	47	654	182	25	126	481	135
3	1	151	0	0	15	4	0	10	5	0	79	1	1	20	4	61	0	0	7	6	27	186
0	0	0	0	0	0	0	0	2	7	0	1	0	0	75	0	0	0	0	0	0	3	0
0	0	0	0	0	0	1	0	0	0	0	0	0	0	0	0	0	0	0	0	0	0	0
1110	262	1603	455	356	1032	1170	330	1152	637	1436	1126	738	747	1003	1320	924	1532	403	5	46	230	131
251	366	1198	1659	82	430	1477	512	6464	1175	2837	1289	894	4534	2604	1697	1021	1659	66	23	190	1750	411
0	0	0	0	0	0	0	0	0	0	0	15	0	0	0	0	0	0	0	0	0	0	0
1	1	0	0	0	0	0	0	0	0	0	0	0	0	0	0	0	0	0	0	0	0	33
4	1	0	1	0	0	4	0	3	0	0	0	0	0	0	0	0	0	15	97	10	73	186
0	0	0	0	0	0	0	0	0	0	0	0	0	0	0	0	0	0	0	0	0	0	0
7	0	0	0	0	0	0	0	0	0	0	0	0	0	0	0	0	0	0	0	2	2	0
251	96	106	43	53	869	388	963	63	630	426	122	91	223	41	238	2050	320	210	2	10	313	50
0	0	0	0	0	0	0	0	0	0	0	0	0	0	0	0	0	0	0	0	0	0	0
2190	3092	2726	55	1638	5087	1561	3416	1852	3850	3071	2062	1334	2633	824	1913	2067	3236	1	1	25	125	4
7	0	0	35	0	0	0	1	0	0	0	6	0	0	0	0	0	0	262	0	1	4	3
0	0	0	0	0	0	0	0	0	0	0	0	0	0	0	0	0	0	0	0	0	0	0
0	0	0	0	0	0	0	0	0	0	0	0	0	0	0	0	0	0	0	0	0	0	0
0	0	0	0	0	1	0	0	0	0	0	4	0	0	1	0	1	0	0	0	0	0	0
0	0	0	0	0	0	0	0	0	0	0	0	0	0	0	0	0	0	0	0	0	0	0
0	0	0	0	0	0	0	0	0	0	0	0	0	0	0	0	0	0	0	0	0	0	0
0	0	0	0	0	0	0	0	0	0	0	0	0	0	0	0	0	0	0	0	0	0	0
0	0	0	0	0	0	0	0	0	0	0	0	0	0	0	0	0	0	0	0	0	0	0
0	0	0	0	0	0	0	0	0	0	0	0	0	0	0	0	0	0	0	0	0	0	0
0	0	0	2	0	0	0	0	0	0	0	0	0	0	0	0	0	0	0	0	1	0	0
0	0	0	0	0	0	0	0	0	0	0	0	0	0	0	0	0	0	0	0	0	0	0
0	0	0	0	0	0	0	0	0	0	0	0	0	0	0	0	0	0	0	0	0	0	0
38	0	0	12	0	0	0	4	10	4	0	12	0	0	0	0	0	0	45	56	24	0	29
0	0	0	0	0	0	0	0	0	0	0	0	0	0	0	0	0	0	0	1	0	0	0
0	0	0	2	0	0	0	0	0	0	0	2	0	0	0	0	0	0	0	20	3	0	0
0	0	0	1	0	0	0	0	0	0	0	0	0	0	0	0	0	0	0	1	0	0	0
0	0	0	0	0	0	0	0	0	0	0	0	0	0	0	0	0	0	0	0	0	0	0
96	4	1	2	0	0	0	0	0	0	0	1	0	0	0	0	0	0	34	0	4	34	93
0	0	0	0	0	0	0	0	0	0	0	0	0	0	0	0	0	0	0	0	0	0	0
9	10	18	441	1	31	34	26	31	13	30	27	4	44	31	16	14	11	220	24	38	659	229
59	1	101	27	4	2	17	28	7	2	48	28	22	0	15	60	16	7	0	3	15	0	153
179	13	23	535	7	119	9	10	1	11	66	117	24	41	54	74	1	27	60	3	32	63	36

C3.016	C3.017	C3.018	C3.019	C3.020	C3.021	C3.024	C3.025	C3.026	C3.027	C3.030	C3.031	C3.032	C3.033	C3.034	C3.036	C3.037	C3.038	E1.006	E1.010	E1.012	E1.013	E1.014
Oral Ca	Oral Ca	Oral Ca	Oral Ca	Oral Ca	Oral Ca	Oral Ca	Oral Ca	Oral Ca	Oral Ca	Oral Ca	Oral Ca	Oral Ca	Oral Ca	Oral Ca	Oral Ca	Oral Ca	Oral Ca	Esophag	Esophag	Esophag	Esophag	Esophag
SP	SP	SP	SP	SP	SP	SP	SP	SP	SP	SP	SP	SP	SP	SP	SP	SP	SP	UE	UE	UE	UE	UE
0	0	0	0	0	0	0	0	0	0	0	0	0	0	0	0	0	0	0	7	2	0	0
2	0	0	83	0	0	103	271	136	13	0	1	0	0	0	1	0	0	0	103	60	275	31
0	0	0	0	0	0	0	0	0	0	0	0	0	0	0	0	0	0	0	0	0	0	0
1107	213	479	7243	82	211	274	141	455	69	597	924	173	581	327	648	425	220	3446	325	246	12079	2465
0	0	0	0	0	0	0	0	0	0	0	0	0	0	0	0	0	0	0	0	1	0	13
0	0	0	0	0	0	0	0	0	0	0	0	0	0	1	0	0	0	0	0	0	0	0
4	10	308	47	42	66	9	73	25	32	29	613	57	52	185	81	168	19	0	4	16	251	54
0	4	46	2	2	23	6	96	24	2	18	18	16	1	25	2	58	2	0	32	3	11	11
0	0	0	0	0	0	1	0	0	0	0	0	0	0	0	0	0	0	3	0	0	0	52
0	0	2	0	0	0	3	6	10	11	0	1	0	0	2	0	0	0	1	12	10	7	36
0	0	0	0	0	0	0	0	0	0	0	0	0	0	0	0	0	0	0	0	0	0	0
0	0	17	0	0	0	0	0	0	0	0	11	0	0	1	0	5	1	0	0	0	10	0
191	71	123	185	48	71	342	42	199	55	220	462	249	306	190	277	342	193	131	102	125	216	226
1	0	2	0	2	0	7	4	8	3	3	4	0	1	2	6	5	1	4	0	0	0	0
12	19	478	19	24	116	69	41	83	16	76	261	103	30	182	116	119	39	88	59	8	50	71
3	0	5	1	0	0	3	0	0	0	0	0	0	0	0	0	1	0	55	137	19	75	283
0	0	25	0	0	0	0	0	0	0	0	17	0	0	15	0	0	0	0	0	0	0	0
387	143	257	239	17	112	202	61	392	136	1079	850	301	1393	782	233	491	629	44	23	36	564	110
0	0	0	0	0	0	0	0	0	0	0	0	0	0	0	0	0	0	0	0	0	0	0
1	0	16	28	0	4	7	4	11	2	12	12	6	38	17	7	34	2	4	44	29	29	118
741	420	499	176	195	854	554	579	537	1199	1451	566	767	535	340	609	684	1097	87	1	4	479	170
530	1390	102	87	56	385	122	182	947	491	1308	396	66	722	80	223	346	773	50	21	11	19	32
0	0	0	0	0	0	0	0	0	0	0	0	0	0	0	0	0	0	0	0	0	0	0
0	0	0	0	0	0	0	0	0	0	0	0	0	0	0	0	0	0	0	0	0	0	0
24	1	0	13	8	1	3	2	4	0	2	7	0	1	1	0	1	0	238	291	118	282	478
0	0	0	0	0	0	0	0	0	0	0	0	0	0	0	0	0	0	0	0	0	0	0
0	0	0	0	0	0	0	0	0	0	0	0	0	0	0	0	0	0	0	0	0	3	0
0	0	0	0	0	0	0	0	0	0	0	0	0	0	0	0	0	0	0	0	1	0	6
2	0	0	5	1	0	0	0	1	0	0	1	0	0	0	0	0	0	43	18	5	28	103
0	0	0	0	0	0	1	0	1	0	0	0	0	0	0	0	0	0	0	37	41	0	70
0	0	0	0	0	0	0	2	1	0	0	0	0	0	0	0	0	0	0	4	0	0	18
5	0	0	1	0	0	0	0	1	0	0	1	0	0	0	0	0	0	114	688	141	6	69
0	0	0	0	0	0	0	0	0	0	0	0	0	0	0	0	0	0	0	0	0	0	21
0	0	1	14	3	1	0	0	0	0	0	0	0	0	0	0	0	0	26	6	1	0	0
0	0	0	0	0	0	0	0	0	0	0	0	0	0	0	0	0	0	0	0	2	0	0
0	0	0	0	0	0	0	0	0	0	0	0	0	0	0	0	0	0	12	0	0	0	0
0	0	0	0	0	0	0	0	0	0	0	0	0	0	0	0	0	0	0	0	0	0	0

C3.016	C3.017	C3.018	C3.019	C3.020	C3.021	C3.024	C3.025	C3.026	C3.027	C3.030	C3.031	C3.032	C3.033	C3.034	C3.036	C3.037	C3.038	E1.006	E1.010	E1.012	E1.013	E1.014
Oral Ca	Oral Ca	Oral Ca	Oral Ca	Oral Ca	Oral Ca	Oral Ca	Oral Ca	Oral Ca	Oral Ca	Oral Ca	Oral Ca	Oral Ca	Oral Ca	Oral Ca	Oral Ca	Oral Ca	Oral Ca	Esophag	Esophag	Esophag	Esophag	Esophag
SP	SP	SP	SP	SP	SP	SP	SP	SP	SP	SP	SP	SP	SP	SP	SP	SP	SP	UE	UE	UE	UE	UE
0	0	0	0	0	0	0	0	0	0	0	0	0	0	0	0	0	0	0	0	14	0	23
2	0	0	0	0	0	0	0	0	1	0	0	0	0	0	0	0	0	7	72	4	5	101
0	0	0	0	0	0	0	0	1	0	0	0	0	0	0	0	0	0	0	14	10	0	27
1	0	0	0	0	0	0	0	0	0	0	0	0	0	0	0	0	0	0	0	0	0	0
0	0	0	0	0	0	0	0	0	0	0	0	0	0	0	0	0	0	0	0	0	3	0
0	0	0	0	0	0	0	0	0	0	0	0	0	0	0	0	0	0	0	0	0	0	0
0	0	0	0	0	0	0	0	0	0	0	0	0	0	0	0	0	0	0	4	0	0	0
6	0	0	9	1	0	287	5	134	0	1	1	0	0	0	0	1	0	89	7946	2815	136	209
0	0	0	2	0	0	3	0	1	0	0	0	0	0	0	0	0	0	14	2	3	0	63
1661	327	524	23	686	640	98	321	46	39	403	15	49	158	151	457	213	151	0	0	8	5	30
433	1363	746	16	715	1993	211	248	49	663	521	617	36	1064	1372	911	957	549	55	548	150	16	53
1	0	0	7	0	0	1	0	2	0	0	5	0	1	0	0	0	0	88	123	57	26	99
0	0	0	1	0	0	0	0	0	0	0	0	0	0	0	0	0	0	0	1	0	0	0
6867	6418	6846	4624	216	4390	1547	1805	446	1705	4913	3821	2662	2632	2824	4485	1511	3720	990	53	240	133	879
0	1	1	0	3	6	0	2	1	2	34	1	8	1	4	30	0	2	0	0	3	15	2
0	0	0	0	0	0	0	0	0	0	0	0	0	0	0	0	0	0	0	0	0	0	0
0	0	0	0	0	0	0	0	0	0	0	0	0	0	0	0	0	0	0	1	0	0	0
0	0	5	0	30	20	1	0	1	0	0	15	6	1	53	29	0	1	0	0	0	0	0
0	0	48	0	0	0	0	0	0	0	0	23	0	0	1	0	0	0	0	0	0	0	0
0	0	44	0	1	10	0	2	8	5	0	44	0	0	34	0	0	0	0	4	2	43	16
0	0	0	0	0	0	0	0	0	0	0	0	0	0	0	0	0	0	0	1	0	0	0
0	0	0	0	0	0	0	0	0	0	0	0	0	0	0	0	0	0	0	0	0	0	0
0	0	0	0	0	0	0	0	0	0	0	0	0	0	0	0	0	0	0	0	0	0	0
0	0	0	0	0	0	0	0	0	0	0	0	0	0	0	0	0	0	0	0	0	3	0
132	296	343	112	91	415	412	273	297	171	501	270	136	345	636	389	375	213	1	0	14	176	74
0	0	0	0	0	0	0	2	7	0	9	1	1	2	7	110	0	0	1989	0	33	0	17
0	0	0	0	0	0	0	0	0	0	0	0	0	0	0	0	0	0	0	0	0	0	28
0	0	0	0	0	0	3	0	0	0	0	0	0	0	0	0	0	0	0	0	0	0	0
0	0	0	0	0	0	0	0	0	0	0	0	0	0	0	0	0	0	0	0	0	0	0
0	0	0	0	0	0	0	0	0	0	0	0	0	0	0	0	0	0	0	0	0	0	0
0	0	0	0	0	0	0	0	0	0	0	0	0	0	0	0	0	0	0	0	0	0	0
308	170	70	1	309	462	112	306	54	127	357	18	51	594	166	96	2	224	0	0	2	0	0
28	0	0	9	2	1	20	8	51	16	0	0	0	0	0	0	0	0	193	321	214	103	1399
0	0	0	0	0	0	0	0	0	0	0	0	0	0	0	0	0	0	0	0	0	0	0
0	0	0	0	0	0	0	0	0	0	0	0	0	0	0	0	0	0	0	0	0	0	0
0	0	0	0	0	0	0	0	0	0	0	0	0	0	0	0	0	0	0	0	0	0	0
0	0	0	0	0	0	0	0	0	0	0	0	0	0	0	0	0	0	0	0	0	0	0

C3.016	C3.017	C3.018	C3.019	C3.020	C3.021	C3.024	C3.025	C3.026	C3.027	C3.030	C3.031	C3.032	C3.033	C3.034	C3.036	C3.037	C3.038	E1.006	E1.010	E1.012	E1.013	E1.014
Oral Ca	Oral Ca	Oral Ca	Oral Ca	Oral Ca	Oral Ca	Oral Ca	Oral Ca	Oral Ca	Oral Ca	Oral Ca	Oral Ca	Oral Ca	Oral Ca	Oral Ca	Oral Ca	Oral Ca	Oral Ca	Esophag	Esophag	Esophag	Esophag	Esophag
SP	SP	SP	SP	SP	SP	SP	SP	SP	SP	SP	SP	SP	SP	SP	SP	SP	SP	UE	UE	UE	UE	UE
898	247	578	1606	22	215	280	176	264	373	1077	430	422	835	1194	2974	15	1331	1677	262	34	902	1183
393	0	1486	11	0	0	0	289	1	3	156	5	438	5	7	676	0	542	95	3	60	10	54
0	0	0	33	3	1	16	4	15	1	0	3	0	0	0	0	0	0	269	37	248	1	100
0	0	0	0	0	0	0	0	0	0	0	0	0	0	0	0	0	0	0	0	0	0	0
0	0	0	0	0	0	0	0	0	0	0	0	0	0	0	0	0	0	0	0	0	0	0
0	0	0	0	0	0	0	0	0	0	0	0	0	0	0	0	0	0	0	0	0	0	0
0	0	0	2	0	0	0	0	0	0	0	0	0	0	0	0	0	0	0	2	6	22	2
0	0	0	77	0	0	2	0	2	0	0	28	0	0	0	0	0	0	336	13	42	43	22
0	0	14	0	0	2	0	0	0	0	0	5	1	0	2	0	14	0	0	0	0	0	0
13	18	467	4	54	82	85	140	305	59	85	1567	104	127	356	208	556	270	1	1	0	182	0
1	0	37	0	5	15	2	5	3	21	1	61	8	3	77	14	22	6	0	0	0	12	0
0	0	0	0	0	0	0	0	0	0	0	0	0	0	0	0	0	0	0	0	0	0	0
0	0	0	0	0	0	0	7	0	0	15	271	28	0	14	0	82	1	0	0	0	0	0
3	0	118	0	0	0	4	7	8	1	0	108	2	4	3	20	52	4	0	4	4	23	9
0	0	0	0	0	0	0	0	0	0	0	0	0	0	0	0	0	0	0	0	0	0	0
0	0	0	7	1	0	14	0	1	0	11	5	6	7	41	0	6	8	0	0	2	18	0
0	0	0	0	0	0	13	4	4	72	76	38	5	11	6	3	1	13	0	2	3	6	0
0	0	0	0	0	0	0	0	0	0	0	0	0	0	0	0	0	0	0	0	0	0	0
0	0	0	2	0	0	0	0	0	0	0	0	0	0	0	0	0	0	0	0	0	20	37
0	0	0	0	0	0	0	0	0	0	0	0	0	0	0	0	0	0	0	0	1	0	0
0	0	0	0	0	0	0	0	0	0	0	0	0	0	0	0	0	0	0	0	0	0	0
0	0	0	0	0	0	0	0	0	0	0	0	0	0	0	0	0	0	0	0	0	0	0
0	0	0	0	0	0	0	0	0	0	0	0	0	0	0	0	0	0	0	0	0	0	0
0	0	0	0	0	0	0	0	0	0	0	0	0	0	0	0	0	0	0	0	0	0	0
0	0	0	0	0	0	0	0	0	0	0	0	0	0	0	0	0	0	0	1	0	0	0
0	0	0	0	0	0	0	0	0	0	0	0	0	0	0	0	0	0	0	0	0	0	0
0	0	0	0	0	0	0	1	0	0	0	0	0	0	0	0	0	0	0	0	0	0	0
0	0	0	0	0	0	0	0	0	0	0	0	0	0	0	0	0	0	0	0	0	0	0
529	250	382	755	1091	498	50	56	29	36	304	69	89	275	352	189	380	132	3	9	10	171	157
0	0	0	3	0	0	0	0	0	0	0	0	0	0	0	0	0	0	0	1	0	0	0
0	0	0	0	0	0	0	0	0	0	0	0	0	0	0	0	0	0	0	0	0	0	0
0	0	0	0	0	0	0	0	0	0	0	0	0	0	0	0	0	0	0	0	0	0	0
51	28	13	14	132	1	1	2	3	0	13	15	3	16	18	6	41	1	0	0	0	0	0
0	0	0	0	0	0	0	0	0	0	0	0	0	0	0	0	0	0	0	0	0	0	0
0	0	0	0	0	0	0	0	0	0	0	0	0	0	0	0	0	0	0	0	1	0	4
632	292	137	21	58	531	46	51	34	46	185	94	98	331	157	141	50	259	7	5	7	23	10
0	0	0	0	0	0	0	0	0	0	0	0	0	0	0	0	0	0	0	0	0	0	0

C3.016	C3.017	C3.018	C3.019	C3.020	C3.021	C3.024	C3.025	C3.026	C3.027	C3.030	C3.031	C3.032	C3.033	C3.034	C3.036	C3.037	C3.038	E1.006	E1.010	E1.012	E1.013	E1.014
Oral Ca	Oral Ca	Oral Ca	Oral Ca	Oral Ca	Oral Ca	Oral Ca	Oral Ca	Oral Ca	Oral Ca	Oral Ca	Oral Ca	Oral Ca	Oral Ca	Oral Ca	Oral Ca	Oral Ca	Oral Ca	Esophag	Esophag	Esophag	Esophag	Esophag
SP	SP	SP	SP	SP	SP	SP	SP	SP	SP	SP	SP	SP	SP	SP	SP	SP	SP	UE	UE	UE	UE	UE
0	0	0	0	0	0	0	0	0	0	0	0	0	0	0	0	0	0	0	0	0	0	0
0	0	0	0	0	0	0	0	0	0	0	0	0	0	0	0	0	0	0	0	0	0	0
0	0	0	0	0	0	0	0	0	0	0	0	0	0	0	0	0	0	0	0	0	0	0
0	0	0	0	0	0	0	0	0	0	0	0	0	0	0	0	0	0	0	0	0	0	0
0	0	0	0	0	0	0	0	0	0	0	0	0	0	0	0	0	0	0	0	0	0	0
0	0	0	0	0	0	0	0	0	0	0	0	0	0	0	0	0	0	0	0	1	0	6
0	0	0	0	0	0	0	0	0	0	0	0	0	0	0	0	0	0	0	0	0	0	0
0	0	0	0	0	0	0	0	0	0	0	0	0	0	0	0	0	0	0	0	0	0	0
0	0	0	0	0	0	0	0	0	0	0	0	0	0	0	0	0	0	0	0	0	0	0
0	0	0	0	0	0	0	0	0	0	0	0	0	0	0	0	0	0	0	0	0	0	0
0	0	0	0	0	0	0	0	0	0	0	0	0	0	0	0	0	0	0	0	0	0	0
0	0	0	0	0	0	0	0	0	0	0	0	0	0	0	0	0	0	0	0	0	0	0
0	0	0	2	0	0	0	0	0	0	0	0	0	0	0	0	0	0	0	0	2	0	9
0	0	0	0	0	0	0	0	0	0	0	0	0	0	0	0	0	0	0	0	0	0	0
0	0	0	0	0	0	0	0	0	0	0	0	0	0	0	0	0	0	0	0	0	0	0
0	0	0	0	0	0	0	0	0	0	0	0	0	0	0	0	0	0	0	0	0	0	0
0	0	0	0	0	0	0	0	0	0	0	0	0	0	0	0	0	0	0	0	0	0	0
0	0	0	0	0	0	0	0	0	0	0	0	0	0	0	0	0	0	0	0	0	0	0
0	0	0	0	0	0	0	0	0	0	0	0	0	0	0	0	0	0	0	0	0	0	0
0	0	0	0	0	0	0	0	0	0	0	0	0	0	0	0	0	0	0	0	0	0	0
4	0	0	0	0	0	0	0	0	0	0	0	0	0	0	0	0	0	6	0	0	0	8
0	0	0	0	0	0	0	0	0	0	0	0	0	0	0	0	0	0	0	0	0	0	0
0	0	0	0	0	0	0	0	0	0	0	0	0	0	0	0	0	0	0	0	0	0	0
0	0	0	0	0	0	0	0	0	0	0	0	0	0	0	0	0	0	0	0	0	0	0
0	0	0	0	0	0	0	0	0	0	0	0	0	0	0	0	0	0	0	0	1	0	15
0	0	0	0	0	0	0	0	0	0	0	0	0	0	0	0	0	0	0	0	0	0	0
0	0	0	0	0	0	0	0	0	0	0	0	0	0	0	0	0	0	0	0	0	0	0
0	0	0	0	0	0	0	0	0	0	0	0	0	0	0	0	0	0	0	0	0	0	0
0	0	0	0	0	0	0	0	0	0	0	0	0	0	0	0	0	0	0	0	0	0	0
0	0	0	0	0	0	0	0	0	0	0	0	0	0	0	0	0	0	0	0	0	0	0
0	2	0	7	0	0	0	0	0	0	0	5	0	0	0	0	0	1	0	0	0	0	0
133	39	18	154	80	43	7	3	3	1	32	7	18	12	31	4	95	8	11	4	5	161	25
0	0	0	0	0	0	0	0	0	0	0	0	0	0	0	0	0	0	0	0	1	0	0
1	0	0	0	0	0	0	0	0	0	0	0	0	0	0	0	0	0	0	1	1	0	0
0	0	0	0	0	0	0	0	0	0	0	0	0	0	0	0	0	0	0	0	0	0	0
0	0	0	0	0	0	0	0	0	0	0	0	0	0	0	0	0	0	0	0	0	0	0
0	0	0	0	0	0	0	0	0	0	0	0	0	0	0	0	0	0	0	0	0	0	0

C3.016	C3.017	C3.018	C3.019	C3.020	C3.021	C3.024	C3.025	C3.026	C3.027	C3.030	C3.031	C3.032	C3.033	C3.034	C3.036	C3.037	C3.038	E1.006	E1.010	E1.012	E1.013	E1.014
Oral Ca	Oral Ca	Oral Ca	Oral Ca	Oral Ca	Oral Ca	Oral Ca	Oral Ca	Oral Ca	Oral Ca	Oral Ca	Oral Ca	Oral Ca	Oral Ca	Oral Ca	Oral Ca	Oral Ca	Oral Ca	Esophag	Esophag	Esophag	Esophag	Esophag
SP	SP	SP	SP	SP	SP	SP	SP	SP	SP	SP	SP	SP	SP	SP	SP	SP	SP	UE	UE	UE	UE	UE
0	0	0	0	0	0	0	0	0	0	0	0	0	0	0	0	0	0	0	6	0	0	2
0	0	0	0	0	0	0	0	0	0	0	0	0	0	0	0	0	0	0	0	0	0	0
0	0	0	0	0	0	0	0	0	0	0	0	0	0	0	0	0	0	0	0	0	0	0
0	0	0	0	0	0	0	0	0	0	0	0	0	0	0	0	0	0	0	0	0	0	0
0	0	0	0	0	0	0	0	0	0	0	0	0	0	0	0	0	0	0	0	0	0	0
0	0	0	0	0	0	0	0	0	0	0	0	0	0	0	0	0	0	0	0	0	0	4
0	0	0	0	0	1	0	8	0	0	3	0	0	0	13	0	0	0	0	0	0	0	0
0	0	0	0	0	0	0	0	0	0	0	0	0	0	0	0	0	0	0	0	0	0	0
0	0	0	0	0	0	0	0	1	0	0	0	0	0	0	0	0	0	0	0	2	0	0
0	0	0	0	0	0	0	0	0	0	0	0	0	0	0	0	0	0	0	0	0	0	0
0	0	0	0	0	0	0	0	0	0	0	0	0	0	0	0	0	0	0	0	0	0	0
0	0	0	0	0	0	0	0	0	0	0	0	0	0	0	0	0	0	0	0	0	0	0
0	0	0	0	0	0	0	0	0	0	0	0	0	0	0	0	0	0	0	0	0	0	0
0	0	0	0	0	0	0	0	0	0	0	0	0	0	0	0	0	0	0	0	0	0	0
0	0	0	0	0	0	0	0	0	0	0	0	0	0	0	0	0	0	3	4	15	25	79
0	0	0	0	0	0	0	0	0	0	2	0	0	3	0	0	0	0	0	0	0	0	0
564	44	355	52	910	404	7	37	17	24	229	33	34	137	156	230	275	168	0	1	0	22	5
0	0	0	0	0	0	0	0	0	0	0	0	0	0	0	0	0	0	0	0	0	0	0
0	0	0	0	0	0	0	0	0	0	0	0	0	0	0	0	0	0	0	0	0	0	0
0	0	0	0	0	0	0	0	0	0	0	0	0	0	0	0	0	0	0	0	0	0	0
0	0	0	0	0	0	0	0	0	0	0	0	0	0	0	0	0	0	0	0	0	0	0
0	0	0	0	0	0	0	0	0	0	0	0	0	0	0	0	0	0	0	0	0	0	0
0	0	0	0	0	0	0	0	0	0	0	0	0	0	0	0	0	0	0	0	0	0	0
1	0	0	0	0	0	0	0	0	0	0	0	0	0	0	0	0	0	53	1	0	13	26
0	0	0	0	0	0	0	0	0	0	0	0	0	0	0	0	0	0	0	0	0	0	0
0	0	0	0	0	0	0	0	0	0	0	0	0	0	0	0	0	0	0	0	0	0	0
0	0	0	0	0	0	0	0	0	0	0	0	0	0	0	0	0	0	0	0	0	0	0
0	0	0	5	0	0	0	0	0	0	0	0	0	0	0	0	0	0	0	359	13	10	0
0	0	0	0	0	0	0	0	1	0	0	0	0	0	0	0	0	0	0	1	0	0	0
0	0	0	21	1	0	0	0	0	0	0	13	0	0	0	1	0	0	0	0	0	3	0
0	0	0	0	0	0	0	0	0	0	0	0	0	0	0	0	0	0	0	1	1	0	0
1	0	0	2	0	0	5	44	47	11	0	0	0	0	0	0	0	0	0	160	26	0	0
0	0	0	0	0	0	0	0	0	0	0	0	0	0	0	0	0	0	0	0	0	0	3
0	0	0	19	0	0	0	2	1	0	1	2	0	13	0	0	0	0	0	0	0	6	1
0	0	0	0	0	0	0	0	0	0	0	0	0	0	0	0	0	0	0	1	0	0	0
0	0	0	0	0	0	0	0	0	0	0	0	0	0	0	0	0	0	0	0	0	0	0
0	0	4	0	1	0	0	0	0	0	0	0	0	0	1	0	0	0	0	0	0	0	0

C3.016	C3.017	C3.018	C3.019	C3.020	C3.021	C3.024	C3.025	C3.026	C3.027	C3.030	C3.031	C3.032	C3.033	C3.034	C3.036	C3.037	C3.038	E1.006	E1.010	E1.012	E1.013	E1.014
Oral Ca	Oral Ca	Oral Ca	Oral Ca	Oral Ca	Oral Ca	Oral Ca	Oral Ca	Oral Ca	Oral Ca	Oral Ca	Oral Ca	Oral Ca	Oral Ca	Oral Ca	Oral Ca	Oral Ca	Oral Ca	Esophag	Esophag	Esophag	Esophag	Esophag
SP	SP	SP	SP	SP	SP	SP	SP	SP	SP	SP	SP	SP	SP	SP	SP	SP	SP	UE	UE	UE	UE	UE
0	0	0	0	0	0	0	0	0	0	0	1	0	0	0	0	0	0	0	0	0	0	0
0	0	1	1	0	0	0	5	1	3	2	4	1	1	3	2	3	1	0	18	2	9	0
0	0	0	0	0	0	0	0	0	0	0	0	0	0	0	0	0	0	0	0	0	0	0
0	0	0	0	0	0	0	0	0	0	0	0	0	0	0	0	0	0	0	0	0	0	0
0	0	0	0	0	0	0	0	0	0	0	0	0	0	0	0	0	0	0	0	0	0	0
0	0	0	0	0	0	0	0	0	0	0	0	0	0	0	0	0	0	0	0	0	0	0
0	0	0	1	0	0	0	0	0	0	0	0	0	0	0	0	0	0	0	2	11	0	0
0	0	0	0	0	0	0	0	0	0	0	0	0	0	0	0	0	0	0	0	0	0	0
0	0	0	0	0	0	0	0	0	0	0	0	0	0	0	0	0	0	0	0	0	0	0
0	0	0	0	0	0	0	0	0	0	0	0	0	0	0	0	0	0	0	0	0	0	0
0	0	0	0	0	0	0	0	0	0	0	0	0	0	0	0	0	0	0	0	0	0	0
0	0	0	0	0	0	1	1	0	0	1	0	0	1	0	0	0	0	18	15	14	9	10
0	0	0	0	0	0	0	0	0	0	0	1	0	0	0	0	0	0	31	0	0	9	4
0	0	0	0	0	0	0	0	0	0	0	0	0	0	0	0	0	0	0	0	0	0	0
0	0	0	0	0	0	0	0	0	0	0	0	0	0	0	0	0	0	0	0	0	0	0
0	0	0	0	0	0	0	0	0	0	0	0	0	0	0	0	0	0	0	0	0	0	0
0	0	0	0	0	0	0	0	0	0	0	0	0	0	0	0	0	0	0	1	0	0	1
0	0	0	0	0	0	0	0	0	0	0	0	0	0	59	0	0	0	0	0	0	0	0
41	24	278	739	25	34	119	101	637	270	315	565	268	916	396	381	47	654	182	25	126	481	135
0	0	0	0	0	0	0	0	0	0	0	0	0	0	0	0	0	0	0	0	0	0	0
0	0	0	0	0	0	0	0	0	0	0	0	0	0	0	0	0	0	0	0	0	0	0
0	0	0	0	0	0	0	0	2	0	1	2	1	2	3	1	0	0	0	0	0	0	0
3	1	151	0	0	15	4	0	10	5	0	79	1	1	20	4	61	0	0	7	6	27	186
0	0	0	0	0	0	0	0	2	7	0	1	0	0	75	0	0	0	0	0	0	3	0
0	0	0	0	0	0	1	0	0	0	0	0	0	0	0	0	0	0	0	0	0	0	0
0	0	0	0	0	0	0	0	0	0	0	0	0	0	0	0	0	0	0	0	0	0	0
39	25	59	1	124	96	255	92	541	213	285	109	94	130	227	203	226	69	0	0	0	0	0
0	0	0	0	0	0	0	0	0	0	0	0	0	0	0	0	0	0	0	1	2	0	0
962	226	1459	450	185	818	735	192	330	351	720	973	575	353	696	1048	598	1308	403	4	43	194	124
109	11	85	4	47	118	180	46	281	73	431	44	69	264	80	69	100	155	0	0	1	36	0
0	0	0	0	0	0	0	0	0	0	0	0	0	0	0	0	0	0	0	0	0	0	7
251	366	1198	1659	82	430	1477	512	6464	1175	2837	1289	894	4534	2604	1697	1021	1659	66	23	190	1750	411
0	0	0	0	0	0	0	0	0	0	0	15	0	0	0	0	0	0	0	0	0	0	0
0	0	0	0	0	0	0	0	0	0	0	0	0	0	0	0	0	0	0	0	0	0	0
0	1	0	0	0	0	0	0	0	0	0	0	0	0	0	0	0	0	0	0	0	0	0
0	0	0	0	0	0	0	0	0	0	0	0	0	0	0	0	0	0	0	0	0	0	0
1	0	0	0	0	0	0	0	0	0	0	0	0	0	0	0	0	0	0	0	0	0	33

C3.016	C3.017	C3.018	C3.019	C3.020	C3.021	C3.024	C3.025	C3.026	C3.027	C3.030	C3.031	C3.032	C3.033	C3.034	C3.036	C3.037	C3.038	E1.006	E1.010	E1.012	E1.013	E1.014
Oral Ca	Oral Ca	Oral Ca	Oral Ca	Oral Ca	Oral Ca	Oral Ca	Oral Ca	Oral Ca	Oral Ca	Oral Ca	Oral Ca	Oral Ca	Oral Ca	Oral Ca	Oral Ca	Oral Ca	Oral Ca	Esophag	Esophag	Esophag	Esophag	Esophag
SP	SP	SP	SP	SP	SP	SP	SP	SP	SP	SP	SP	SP	SP	SP	SP	SP	SP	UE	UE	UE	UE	UE
4	1	0	1	0	0	4	0	3	0	0	0	0	0	0	0	0	0	15	97	10	73	186
0	0	0	0	0	0	0	0	0	0	0	0	0	0	0	0	0	0	0	0	0	0	0
0	0	0	0	0	0	0	0	0	0	0	0	0	0	0	0	0	0	0	0	0	0	0
0	0	0	0	0	0	0	0	0	0	0	0	0	0	0	0	0	0	0	0	0	0	0
0	0	0	0	0	0	0	0	0	0	0	0	0	0	0	0	0	0	0	0	1	2	0
0	0	0	0	0	0	0	0	0	0	0	0	0	0	0	0	0	0	0	0	0	0	0
7	0	0	0	0	0	0	0	0	0	0	0	0	0	0	0	0	0	0	0	1	0	0
0	0	0	0	0	0	0	0	0	0	0	0	0	0	0	0	0	0	0	0	0	0	0
0	0	0	0	0	0	0	0	0	0	0	0	0	0	0	0	0	0	0	0	0	0	0
0	0	0	0	0	0	0	0	0	0	0	0	0	0	0	0	0	0	0	0	0	0	0
0	0	0	0	0	0	0	0	0	0	0	0	0	0	0	0	0	0	0	0	0	0	0
0	0	0	0	0	0	0	0	0	0	0	0	0	0	0	0	0	0	0	0	0	0	0
0	0	0	4	0	0	0	0	1	0	1	2	0	0	0	0	0	0	210	1	5	14	50
0	0	0	0	0	0	0	0	0	0	0	0	0	0	1	0	0	0	0	0	0	0	0
0	0	0	0	0	0	0	0	1	0	0	0	0	0	0	0	0	0	0	1	1	0	0
0	0	0	0	0	0	0	0	0	0	0	0	0	0	0	0	0	0	0	0	1	0	0
0	0	0	0	0	0	0	0	0	2	0	0	0	0	0	0	0	0	0	0	0	0	0
251	96	106	39	53	869	388	963	61	628	425	120	91	223	40	238	2050	320	0	0	3	299	0
0	0	0	0	0	0	0	0	0	0	0	0	0	0	0	0	0	0	0	0	0	0	0
2190	3092	2726	55	1638	5087	1561	3416	1852	3850	3071	2062	1334	2633	824	1913	2067	3236	1	1	21	125	4
0	0	0	0	0	0	0	0	0	0	0	0	0	0	0	0	0	0	0	0	4	0	0
0	0	0	0	0	0	0	0	0	0	0	0	0	0	0	0	0	0	0	0	0	0	0
0	0	0	0	0	0	0	0	0	5	0	0	0	0	0	0	3	5	0	0	0	0	0
4	0	0	35	0	0	0	1	0	0	0	6	0	0	0	0	0	0	262	0	0	4	3
3	0	0	0	0	0	0	0	0	0	0	0	0	0	0	0	0	0	0	0	1	0	0
0	0	0	0	0	0	0	0	0	0	0	0	0	0	0	0	0	0	0	0	0	0	0
0	0	0	0	0	0	0	0	0	0	0	0	0	0	0	0	0	0	0	0	0	0	0
0	0	0	0	0	0	0	0	0	0	0	0	0	0	0	0	0	0	0	0	0	0	0
0	0	0	0	0	1	0	0	0	0	0	4	0	0	1	0	1	0	0	0	0	0	0
0	0	0	0	0	0	0	0	0	0	0	0	0	0	0	0	0	0	0	0	0	0	0
0	0	0	0	0	0	0	0	0	0	0	0	0	0	0	0	0	0	0	0	0	0	0
0	0	0	0	0	0	0	0	0	0	0	0	0	0	0	0	0	0	0	0	0	0	0
0	0	0	0	0	0	0	0	0	0	0	0	0	0	0	0	0	0	0	0	0	0	0
0	0	0	0	0	0	0	0	0	0	0	0	0	0	0	0	0	0	0	0	0	0	0
0	0	0	0	0	0	0	0	0	0	0	0	0	0	0	0	0	0	0	0	0	0	0
0	0	0	2	0	0	0	0	0	0	0	0	0	0	0	0	0	0	0	0	1	0	0
0	0	0	0	0	0	0	0	0	0	0	0	0	0	0	0	0	0	0	0	0	0	0

C3.016	C3.017	C3.018	C3.019	C3.020	C3.021	C3.024	C3.025	C3.026	C3.027	C3.030	C3.031	C3.032	C3.033	C3.034	C3.036	C3.037	C3.038	E1.006	E1.010	E1.012	E1.013	E1.014
Oral Ca	Oral Ca	Oral Ca	Oral Ca	Oral Ca	Oral Ca	Oral Ca	Oral Ca	Oral Ca	Oral Ca	Oral Ca	Oral Ca	Oral Ca	Oral Ca	Oral Ca	Oral Ca	Oral Ca	Oral Ca	Esophag	Esophag	Esophag	Esophag	Esophag
SP	SP	SP	SP	SP	SP	SP	SP	SP	SP	SP	SP	SP	SP	SP	SP	SP	SP	UE	UE	UE	UE	UE
0	0	0	0	0	0	0	0	0	0	0	0	0	0	0	0	0	0	0	0	0	0	0
0	0	0	0	0	0	0	0	0	0	0	0	0	0	0	0	0	0	0	0	0	0	4
38	0	0	12	0	0	0	4	10	4	0	12	0	0	0	0	0	0	45	56	24	0	25
0	0	0	0	0	0	0	0	0	0	0	0	0	0	0	0	0	0	0	0	0	0	0
0	0	0	0	0	0	0	0	0	0	0	0	0	0	0	0	0	0	0	0	0	0	0
0	0	0	0	0	0	0	0	0	0	0	0	0	0	0	0	0	0	0	0	0	0	0
0	0	0	0	0	0	0	0	0	0	0	0	0	0	0	0	0	0	0	0	0	0	0
0	0	0	0	0	0	0	0	0	0	0	0	0	0	0	0	0	0	0	1	0	0	0
0	0	0	0	0	0	0	0	0	0	0	0	0	0	0	0	0	0	0	0	0	0	0
0	0	0	2	0	0	0	0	0	0	0	2	0	0	0	0	0	0	0	18	3	0	0
0	0	0	0	0	0	0	0	0	0	0	0	0	0	0	0	0	0	0	2	0	0	0
0	0	0	0	0	0	0	0	0	0	0	0	0	0	0	0	0	0	0	0	0	0	0
0	0	0	0	0	0	0	0	0	0	0	0	0	0	0	0	0	0	0	0	0	0	0
0	0	0	0	0	0	0	0	0	0	0	0	0	0	0	0	0	0	0	0	0	0	0
0	0	0	1	0	0	0	0	0	0	0	0	0	0	0	0	0	0	0	1	0	0	0
0	0	0	0	0	0	0	0	0	0	0	0	0	0	0	0	0	0	0	0	0	0	0
0	0	0	0	0	0	0	0	0	0	0	0	0	0	0	0	0	0	0	0	0	0	0
0	0	0	0	0	0	0	0	0	0	0	0	0	0	0	0	0	0	0	0	0	0	0
0	0	0	0	0	0	0	0	0	0	0	0	0	0	0	0	0	0	0	0	0	0	0
96	4	1	2	0	0	0	0	0	0	0	1	0	0	0	0	0	0	34	0	4	34	93
0	0	0	0	0	0	0	0	0	0	0	0	0	0	0	0	0	0	0	0	0	0	0
0	0	0	0	0	0	0	0	0	0	0	0	0	0	0	0	0	0	0	0	2	0	0
0	0	0	0	0	0	0	25	1	1	0	0	0	0	0	0	0	0	0	19	0	0	0
0	0	0	0	0	0	0	0	0	0	0	0	0	0	0	0	0	1	0	0	0	0	0
9	10	18	441	1	31	34	1	30	12	30	27	4	44	31	16	14	10	220	5	38	659	229
0	0	0	0	0	0	0	0	0	0	0	0	0	0	0	0	0	0	0	0	0	0	0
59	1	100	27	4	2	17	0	7	0	48	28	22	0	15	60	16	6	0	0	10	0	0
0	0	0	0	0	0	0	28	0	2	0	0	0	0	0	0	0	0	0	3	5	0	71
0	0	0	0	0	0	0	0	0	0	0	0	0	0	0	0	0	0	0	0	0	0	0
0	0	0	0	0	0	0	0	0	0	0	0	0	0	0	0	0	0	0	0	0	0	0
0	0	0	0	0	0	0	0	0	0	0	0	0	0	0	0	0	0	0	0	0	0	63
0	0	0	0	0	0	0	0	0	0	0	0	0	0	0	0	0	0	0	0	0	0	0
0	0	1	0	0	0	0	0	0	0	0	0	0	0	0	0	0	1	0	0	0	0	0
0	0	0	0	0	0	0	0	0	0	0	0	0	0	0	0	0	0	0	0	0	0	19
1	0	0	0	0	0	0	0	0	0	0	0	0	0	0	0	0	0	0	0	1	0	0
0	0	0	0	0	0	0	0	0	0	0	0	0	0	0	0	0	0	0	0	0	0	0
178	13	23	535	7	119	9	10	1	11	66	117	24	41	54	74	1	27	60	3	31	63	36

C3.016	C3.017	C3.018	C3.019	C3.020	C3.021	C3.024	C3.025	C3.026	C3.027	C3.030	C3.031	C3.032	C3.033	C3.034	C3.036	C3.037	C3.038	E1.006	E1.010	E1.012	E1.013	E1.014
Oral Ca	Oral Ca	Oral Ca	Oral Ca	Oral Ca	Oral Ca	Oral Ca	Oral Ca	Oral Ca	Oral Ca	Oral Ca	Oral Ca	Oral Ca	Oral Ca	Oral Ca	Oral Ca	Oral Ca	Oral Ca	Esophag	Esophag	Esophag	Esophag	Esophag
SP	SP	SP	SP	SP	SP	SP	SP	SP	SP	SP	SP	SP	SP	SP	SP	SP	SP	UE	UE	UE	UE	UE
0	0	0	0	0	0	0	0	0	0	0	0	0	0	0	0	0	0	0	0	0	0	0
0	0	0	0	0	0	0	0	0	0	0	0	0	0	0	0	0	0	0	0	0	0	0
0	0	0	0	0	0	0	0	0	0	0	0	0	0	0	0	0	0	0	0	0	0	0
0	0	0	0	0	0	0	0	0	0	0	0	0	0	0	0	0	0	0	2	0	0	0
0	0	0	0	0	0	0	0	0	0	0	0	0	0	0	0	0	0	0	0	0	0	0
0	0	0	0	0	0	0	0	0	0	0	0	0	0	0	0	0	0	0	1	1	0	0
0	0	0	0	0	0	0	0	0	0	0	0	0	0	0	0	0	0	0	4	1	0	0
2	0	0	83	0	0	103	271	136	13	0	1	0	0	0	1	0	0	0	103	60	275	31
0	0	0	0	0	0	0	0	0	0	0	0	0	0	0	0	0	0	0	0	0	0	0
0	0	0	0	0	0	0	0	0	0	0	0	0	0	0	0	0	0	0	1	0	0	0
1107	213	479	7243	82	211	274	141	455	69	597	924	173	581	327	648	425	220	3446	324	246	12079	2465
0	0	0	0	0	0	0	0	0	0	0	0	0	0	0	0	0	0	0	0	0	0	0
0	1	0	1	0	0	2	2	1	0	1	0	0	0	0	0	0	3	0	1	0	0	0
0	0	0	0	0	0	0	0	0	0	0	0	0	0	0	0	0	0	0	0	1	0	13
0	0	0	0	0	0	0	0	0	0	0	0	0	0	0	0	0	0	0	0	0	0	0
0	0	0	0	0	0	0	0	0	0	0	0	0	0	1	0	0	0	0	0	0	0	0
0	0	6	2	0	0	0	4	18	24	3	0	0	12	18	3	0	1	0	0	0	0	0
0	0	5	34	1	3	2	2	1	0	3	10	2	16	18	3	16	2	0	0	5	58	10
0	1	63	0	3	1	1	6	0	2	5	49	12	4	13	10	20	3	0	0	0	23	0
4	9	234	11	38	62	6	61	6	6	18	554	43	20	136	65	132	13	0	4	11	170	44
0	0	0	0	0	0	0	0	0	0	0	0	0	0	0	0	0	0	0	0	0	0	0
0	0	0	0	0	0	0	5	3	0	0	0	0	0	0	0	0	0	0	17	1	0	0
0	0	0	0	0	0	1	1	1	0	0	0	0	0	0	0	0	0	0	6	1	0	0
0	0	0	0	0	0	0	0	0	0	0	0	0	0	0	0	0	0	0	0	0	0	0
0	4	40	2	0	7	4	84	18	2	18	9	6	1	13	2	56	2	0	6	1	11	11
0	0	0	0	0	0	1	6	2	0	0	0	0	0	0	0	0	0	0	3	0	0	0
0	0	0	0	0	0	0	0	0	0	0	0	0	0	0	0	0	0	0	0	0	0	0
0	0	0	0	0	0	0	0	0	0	0	0	0	0	0	0	0	0	0	0	0	0	0
0	0	0	0	0	0	0	0	0	0	0	0	0	0	0	0	0	0	0	0	0	0	0
0	0	6	0	2	16	0	0	0	0	0	9	10	0	12	0	2	0	0	0	0	0	0
0	0	0	0	0	0	0	0	0	0	0	0	0	0	0	0	0	0	0	0	0	0	0
0	0	0	0	0	0	1	0	0	0	0	0	0	0	0	0	0	0	3	0	0	0	52
0	0	0	0	0	0	0	0	0	0	0	0	0	0	0	0	0	0	0	0	0	0	0
0	0	2	0	0	0	3	6	10	11	0	1	0	0	0	0	0	0	0	9	7	0	0
0	0	0	0	0	0	0	0	0	0	0	0	0	0	0	0	0	0	0	0	0	0	0
0	0	0	0	0	0	0	0	0	0	0	0	0	0	0	0	0	0	0	0	0	0	0
0	0	0	0	0	0	0	0	0	0	0	0	0	0	0	0	0	0	0	0	0	0	0

C3.016	C3.017	C3.018	C3.019	C3.020	C3.021	C3.024	C3.025	C3.026	C3.027	C3.030	C3.031	C3.032	C3.033	C3.034	C3.036	C3.037	C3.038	E1.006	E1.010	E1.012	E1.013	E1.014
Oral Ca	Oral Ca	Oral Ca	Oral Ca	Oral Ca	Oral Ca	Oral Ca	Oral Ca	Oral Ca	Oral Ca	Oral Ca	Oral Ca	Oral Ca	Oral Ca	Oral Ca	Oral Ca	Oral Ca	Oral Ca	Esophag	Esophag	Esophag	Esophag	Esophag
SP	SP	SP	SP	SP	SP	SP	SP	SP	SP	SP	SP	SP	SP	SP	SP	SP	SP	UE	UE	UE	UE	UE
0	0	0	0	0	0	0	0	0	0	0	0	0	0	2	0	0	0	1	3	3	7	36
0	0	0	0	0	0	0	0	0	0	0	0	0	0	0	0	0	0	0	0	0	0	0
0	0	0	0	0	0	0	0	0	0	0	0	0	0	0	0	0	0	0	0	0	0	0
0	0	0	0	0	0	0	0	0	0	0	0	0	0	0	0	0	0	0	0	0	0	0
0	0	17	0	0	0	0	0	0	0	0	11	0	0	1	0	5	1	0	0	0	10	0
0	0	0	0	0	0	0	0	0	0	0	0	0	0	0	0	0	0	0	1	1	0	4
0	0	0	0	0	0	0	0	0	0	0	0	0	0	0	0	0	0	0	2	1	0	0
0	0	0	0	0	0	1	0	0	0	0	0	0	0	0	0	0	0	0	13	18	63	27
1	1	3	12	0	0	1	0	0	1	0	0	1	2	4	6	0	4	0	1	22	0	0
6	16	51	20	5	7	202	7	69	16	77	57	124	57	64	114	17	52	0	0	4	6	0
0	0	0	0	0	0	2	0	0	0	0	0	0	0	0	0	0	0	0	6	3	15	31
1	0	0	0	0	0	1	0	1	0	0	0	0	0	0	0	0	0	0	4	6	0	16
0	0	0	0	0	0	0	0	0	0	0	0	0	0	0	0	0	0	0	0	0	0	0
0	0	0	0	0	0	0	0	0	0	0	0	0	0	0	0	0	0	0	0	0	0	0
0	0	0	0	0	0	0	0	0	0	0	0	0	0	0	0	0	0	0	0	2	0	7
0	0	0	0	0	0	0	0	0	0	0	0	0	0	0	0	0	0	0	0	0	0	0
0	0	1	80	0	0	2	1	1	0	1	0	1	47	3	3	0	2	0	0	13	62	24
0	0	0	0	0	0	1	0	0	0	0	0	0	0	0	0	0	0	0	3	5	0	2
0	0	0	3	2	0	2	3	3	2	1	0	1	63	3	6	0	0	0	5	3	30	0
0	0	0	0	0	0	2	0	0	0	0	0	0	0	0	0	0	0	0	13	7	0	40
183	54	68	70	41	64	128	31	125	36	141	405	122	137	116	148	325	135	131	54	40	40	75
0	0	0	0	0	0	0	0	0	0	0	0	0	0	0	0	0	0	0	0	0	0	0
1	0	2	0	2	0	7	4	8	3	3	4	0	1	2	6	5	1	4	0	0	0	0
0	0	0	0	0	0	0	0	0	0	0	0	0	0	0	0	0	0	0	0	0	0	0
0	0	0	0	0	0	0	0	0	0	0	0	0	0	0	0	0	0	0	0	0	0	0
4	5	329	0	12	51	43	18	36	9	18	147	63	1	129	37	57	10	0	0	2	50	12
6	0	72	17	0	23	14	12	37	4	25	92	27	12	19	10	50	3	88	59	6	0	56
2	14	77	2	12	42	12	11	10	3	33	22	13	17	34	69	12	26	0	0	0	0	3
0	0	0	0	0	0	0	0	0	0	0	0	0	0	0	0	0	0	0	1	0	0	0
0	0	0	0	0	0	0	0	0	0	0	0	0	0	0	0	0	0	0	0	0	0	0
0	0	0	0	0	0	2	0	0	0	0	0	0	0	0	0	0	0	0	21	1	0	0
1	0	0	0	0	0	0	0	0	0	0	0	0	0	0	0	0	0	47	9	3	26	145
1	0	0	0	0	0	1	0	0	0	0	0	0	0	0	0	1	0	0	77	5	17	7
0	0	0	0	0	0	0	0	0	0	0	0	0	0	0	0	0	0	0	0	0	0	0
1	0	5	1	0	0	0	0	0	0	0	0	0	0	0	0	0	0	8	29	10	32	131
0	0	25	0	0	0	0	0	0	0	0	17	0	0	15	0	0	0	0	0	0	0	0
0	0	0	0	0	0	0	0	0	0	0	0	0	0	0	0	0	0	0	0	0	0	0

C3.016	C3.017	C3.018	C3.019	C3.020	C3.021	C3.024	C3.025	C3.026	C3.027	C3.030	C3.031	C3.032	C3.033	C3.034	C3.036	C3.037	C3.038	E1.006	E1.010	E1.012	E1.013	E1.014
Oral Ca	Oral Ca	Oral Ca	Oral Ca	Oral Ca	Oral Ca	Oral Ca	Oral Ca	Oral Ca	Oral Ca	Oral Ca	Oral Ca	Oral Ca	Oral Ca	Oral Ca	Oral Ca	Oral Ca	Oral Ca	Esophag	Esophag	Esophag	Esophag	Esophag
SP	SP	SP	SP	SP	SP	SP	SP	SP	SP	SP	SP	SP	SP	SP	SP	SP	SP	UE	UE	UE	UE	UE
4	0	85	17	3	90	28	7	36	12	89	20	178	40	138	17	17	4	2	8	1	48	8
0	0	0	0	0	0	0	0	0	0	0	0	0	0	0	0	0	0	0	0	1	0	0
0	2	4	14	0	2	0	2	6	6	16	4	5	24	3	0	1	2	0	11	8	34	14
0	0	0	0	0	0	0	0	0	0	0	0	0	0	0	0	0	0	0	0	0	0	0
1	0	42	0	0	5	5	0	2	5	24	48	20	16	20	2	35	20	0	0	0	0	0
66	54	106	6	7	12	163	42	297	107	494	553	84	802	503	189	434	528	0	0	1	80	2
0	0	6	0	0	0	0	0	0	0	0	0	0	0	0	0	0	0	0	0	0	0	0
0	0	0	0	0	0	0	0	0	0	0	0	0	0	0	0	0	0	0	0	0	0	0
316	86	14	195	7	3	6	9	49	6	456	215	11	369	118	25	3	74	42	4	24	395	86
0	0	0	0	0	0	0	0	0	0	0	0	0	0	0	0	0	1	0	0	0	0	0
0	1	0	7	0	0	0	1	2	0	0	10	3	142	0	0	1	0	0	0	1	7	0
0	0	0	0	0	0	0	0	0	0	0	0	0	0	0	0	1	0	0	11	5	16	56
0	0	0	0	0	0	0	0	0	0	0	0	0	0	0	0	0	0	0	0	0	0	0
0	0	0	0	0	0	1	0	0	0	0	0	0	0	0	0	0	0	0	1	1	0	0
1	0	0	1	0	0	1	0	0	0	0	0	0	0	0	0	0	0	4	34	15	7	71
0	0	9	27	0	0	3	4	6	2	11	9	3	38	6	6	3	2	0	2	13	22	47
0	0	0	0	0	0	0	0	0	0	0	0	0	0	0	0	0	0	0	1	0	0	0
0	0	0	0	0	0	0	0	0	0	0	0	0	0	0	0	0	0	0	2	0	0	0
0	0	0	0	0	0	0	0	0	0	0	0	0	0	0	0	0	0	0	0	0	0	0
0	0	0	0	0	0	0	0	0	0	0	0	0	0	0	0	0	0	0	0	0	0	0
0	0	3	0	0	0	0	0	1	0	0	1	0	0	0	0	1	0	0	0	0	0	0
0	0	3	0	0	4	2	0	4	0	1	2	3	0	3	1	28	0	0	1	0	0	0
0	0	0	0	0	0	0	0	0	0	0	0	0	0	0	0	0	0	0	0	0	0	0
0	0	1	0	0	0	0	0	0	0	0	0	0	0	8	0	2	0	0	3	0	0	0
0	0	0	0	0	0	0	0	0	0	0	0	0	0	0	0	0	0	0	0	0	0	0
741	420	499	176	195	854	554	579	537	1199	1451	566	767	535	340	609	684	1097	87	1	4	479	170
530	1390	102	84	56	385	122	178	946	491	1308	396	66	716	80	223	346	773	50	1	11	19	32
0	0	0	0	0	0	0	4	0	0	0	0	0	0	0	0	0	0	0	20	0	0	0
0	0	0	0	0	0	0	0	1	0	0	0	0	0	0	0	0	0	0	0	0	0	0
0	0	0	3	0	0	0	0	0	0	0	0	0	6	0	0	0	0	0	0	0	0	0
0	0	0	0	0	0	0	0	0	0	0	0	0	0	0	0	0	0	0	0	0	0	0
0	0	0	0	0	0	0	0	0	0	0	0	0	0	0	0	0	0	0	0	0	0	0
0	0	0	0	0	0	0	0	0	0	0	0	0	0	0	0	0	0	0	0	0	0	0
0	0	0	0	0	0	0	0	0	0	0	0	0	0	0	0	0	0	0	0	0	0	0
0	0	0	0	0	0	0	0	0	0	0	0	0	0	0	0	0	0	0	19	10	0	0
0	0	0	0	0	0	0	0	0	0	0	0	0	0	0	0	0	0	0	5	0	1	0
0	0	0	0	0	0	0	0	0	0	0	0	0	0	0	0	0	0	0	0	0	0	0

C3.016	C3.017	C3.018	C3.019	C3.020	C3.021	C3.024	C3.025	C3.026	C3.027	C3.030	C3.031	C3.032	C3.033	C3.034	C3.036	C3.037	C3.038	E1.006	E1.010	E1.012	E1.013	E1.014
Oral Ca	Oral Ca	Oral Ca	Oral Ca	Oral Ca	Oral Ca	Oral Ca	Oral Ca	Oral Ca	Oral Ca	Oral Ca	Oral Ca	Oral Ca	Oral Ca	Oral Ca	Oral Ca	Oral Ca	Oral Ca	Esophag	Esophag	Esophag	Esophag	Esophag
SP	SP	SP	SP	SP	SP	SP	SP	SP	SP	SP	SP	SP	SP	SP	SP	SP	SP	UE	UE	UE	UE	UE
1	0	0	2	0	0	0	0	3	0	0	0	0	0	0	0	0	0	0	76	34	19	14
0	0	0	0	0	0	0	0	0	0	0	0	0	0	0	0	0	0	0	1	0	0	0
23	1	0	11	8	1	3	2	1	0	2	7	0	1	1	0	1	0	238	190	74	262	464
0	0	0	0	0	0	0	0	0	0	0	0	0	0	0	0	0	0	0	0	0	0	0
0	0	0	0	0	0	0	0	0	0	0	0	0	0	0	0	0	0	0	0	0	0	0
0	0	0	0	0	0	0	0	0	0	0	0	0	0	0	0	0	0	0	0	0	3	0
0	0	0	0	0	0	0	0	0	0	0	0	0	0	0	0	0	0	0	0	0	0	6
0	0	0	0	0	0	0	0	0	0	0	0	0	0	0	0	0	0	0	0	0	0	0
0	0	0	0	0	0	0	0	0	0	0	0	0	0	0	0	0	0	0	0	1	0	0
0	0	0	0	0	0	0	0	0	0	0	0	0	0	0	0	0	0	0	0	0	0	2
0	0	0	0	0	0	0	0	0	0	0	0	0	0	0	0	0	0	0	0	0	0	0
2	0	0	2	1	0	0	0	0	0	0	1	0	0	0	0	0	0	43	16	3	28	69
0	0	0	0	0	0	0	0	0	0	0	0	0	0	0	0	0	0	0	0	0	0	0
0	0	0	3	0	0	0	0	1	0	0	0	0	0	0	0	0	0	0	2	2	0	32
0	0	0	0	0	0	0	0	1	0	0	0	0	0	0	0	0	0	0	36	35	0	70
0	0	0	0	0	0	1	0	0	0	0	0	0	0	0	0	0	0	0	1	6	0	0
0	0	0	0	0	0	0	2	1	0	0	0	0	0	0	0	0	0	0	2	0	0	0
0	0	0	0	0	0	0	0	0	0	0	0	0	0	0	0	0	0	0	0	0	0	0
0	0	0	0	0	0	0	0	0	0	0	0	0	0	0	0	0	0	0	2	0	0	0
0	0	0	0	0	0	0	0	0	0	0	0	0	0	0	0	0	0	0	0	0	0	18
0	0	0	0	0	0	0	0	0	0	0	0	0	0	0	0	0	0	0	0	0	0	0
0	0	0	0	0	0	0	0	0	0	0	0	0	0	0	0	0	0	0	0	0	0	0
1	0	0	1	0	0	0	0	1	0	0	1	0	0	0	0	0	0	1	256	49	6	54
4	0	0	0	0	0	0	0	0	0	0	0	0	0	0	0	0	0	113	432	92	0	15
0	0	0	0	0	0	0	0	0	0	0	0	0	0	0	0	0	0	0	0	0	0	0
0	0	0	0	0	0	0	0	0	0	0	0	0	0	0	0	0	0	0	0	0	0	0
0	0	0	0	0	0	0	0	0	0	0	0	0	0	0	0	0	0	0	0	0	0	21
0	0	0	0	0	0	0	0	0	0	0	0	0	0	0	0	0	0	0	0	0	0	0
0	0	0	0	0	0	0	0	0	0	0	0	0	0	0	0	0	0	0	0	0	0	0
0	0	1	14	3	1	0	0	0	0	0	0	0	0	0	0	0	0	26	6	1	0	0
0	0	0	0	0	0	0	0	0	0	0	0	0	0	0	0	0	0	0	0	0	0	0
0	0	0	0	0	0	0	0	0	0	0	0	0	0	0	0	0	0	0	0	1	0	0
0	0	0	0	0	0	0	0	0	0	0	0	0	0	0	0	0	0	0	0	0	0	0
0	0	0	0	0	0	0	0	0	0	0	0	0	0	0	0	0	0	0	0	0	0	0
0	0	0	0	0	0	0	0	0	0	0	0	0	0	0	0	0	0	0	0	1	0	0
0	0	0	0	0	0	0	0	0	0	0	0	0	0	0	0	0	0	0	0	0	0	0
0	0	0	0	0	0	0	0	0	0	0	0	0	0	0	0	0	0	12	0	0	0	0

C3.016	C3.017	C3.018	C3.019	C3.020	C3.021	C3.024	C3.025	C3.026	C3.027	C3.030	C3.031	C3.032	C3.033	C3.034	C3.036	C3.037	C3.038	E1.006	E1.010	E1.012	E1.013	E1.014
Oral Ca	Oral Ca	Oral Ca	Oral Ca	Oral Ca	Oral Ca	Oral Ca	Oral Ca	Oral Ca	Oral Ca	Oral Ca	Oral Ca	Oral Ca	Oral Ca	Oral Ca	Oral Ca	Oral Ca	Oral Ca	Esophag	Esophag	Esophag	Esophag	Esophag
SP	SP	SP	SP	SP	SP	SP	SP	SP	SP	SP	SP	SP	SP	SP	SP	SP	SP	UE	UE	UE	UE	UE
0	0	0	0	0	0	0	0	0	0	0	0	0	0	0	0	0	0	0	0	0	0	0
0	0	0	0	0	0	0	0	0	0	0	0	0	0	0	0	0	0	0	0	1	0	0
0	0	0	0	0	0	0	0	0	0	0	0	0	0	0	0	0	0	0	0	0	0	0
0	0	0	0	0	0	0	0	0	0	0	0	0	0	0	0	0	0	0	0	2	0	0
0	0	0	0	0	0	0	0	0	0	0	0	0	0	0	0	0	0	0	0	4	0	7
0	0	0	0	0	0	0	0	0	0	0	0	0	0	0	0	0	0	0	0	0	0	0
0	0	0	0	0	0	0	0	0	0	0	0	0	0	0	0	0	0	0	0	1	0	0
0	0	0	0	0	0	0	0	0	0	0	0	0	0	0	0	0	0	0	0	0	0	0
0	0	0	0	0	0	0	0	0	0	0	0	0	0	0	0	0	0	0	0	7	0	16
0	0	0	0	0	0	0	0	0	1	0	0	0	0	0	0	0	0	0	24	0	0	6
0	0	0	0	0	0	0	0	0	0	0	0	0	0	0	0	0	0	0	0	0	0	0
0	0	0	0	0	0	0	0	0	0	0	0	0	0	0	0	0	0	0	0	0	0	0
0	0	0	0	0	0	0	0	0	0	0	0	0	0	0	0	0	0	0	0	0	0	90
0	0	0	0	0	0	0	0	0	0	0	0	0	0	0	0	0	0	0	6	2	0	0
2	0	0	0	0	0	0	0	0	0	0	0	0	0	0	0	0	0	7	42	2	5	5
0	0	0	0	0	0	0	0	0	0	0	0	0	0	0	0	0	0	0	0	0	0	0
0	0	0	0	0	0	0	0	0	0	0	0	0	0	0	0	0	0	0	0	1	0	0
0	0	0	0	0	0	0	0	0	0	0	0	0	0	0	0	0	0	0	0	0	0	0
0	0	0	0	0	0	0	0	0	0	0	0	0	0	0	0	0	0	0	0	0	0	0
0	0	0	0	0	0	0	0	1	0	0	0	0	0	0	0	0	0	0	14	9	0	27
1	0	0	0	0	0	0	0	0	0	0	0	0	0	0	0	0	0	0	0	0	0	0
0	0	0	0	0	0	0	0	0	0	0	0	0	0	0	0	0	0	0	0	0	3	0
0	0	0	0	0	0	0	0	0	0	0	0	0	0	0	0	0	0	0	0	0	0	0
0	0	0	0	0	0	0	0	0	0	0	0	0	0	0	0	0	0	0	1	0	0	0
0	0	0	0	0	0	0	0	0	0	0	0	0	0	0	0	0	0	0	3	0	0	0
0	0	0	0	0	0	0	0	0	0	0	0	0	0	0	0	0	0	0	3	1	0	0
0	0	0	0	0	0	0	0	0	0	0	0	0	0	0	0	0	0	0	1	3	0	0
1	0	0	3	1	0	2	0	2	0	0	0	0	0	0	0	1	0	0	3807	655	58	34
0	0	0	2	0	0	0	0	0	0	0	0	0	0	0	0	0	0	17	3183	636	0	33
5	0	0	4	0	0	285	5	132	0	1	0	0	0	0	0	0	0	70	937	1504	78	132
0	0	0	0	0	0	0	0	0	0	0	0	0	0	0	0	0	0	0	0	0	0	0
0	0	0	0	0	0	0	0	0	0	0	0	0	0	0	0	0	0	0	14	14	0	0
0	0	0	0	0	0	0	0	0	0	0	1	0	0	0	0	0	0	2	1	2	0	10
0	0	0	0	0	0	0	0	0	0	0	0	0	0	0	0	0	0	0	0	0	0	0
0	0	0	0	0	0	0	0	0	0	0	0	0	0	0	0	0	0	0	0	0	0	0
0	0	0	0	0	0	0	0	0	0	0	0	0	0	0	0	0	0	0	1	0	0	11
0	0	0	2	0	0	1	0	1	0	0	0	0	0	0	0	0	0	14	1	0	0	52

C3.016	C3.017	C3.018	C3.019	C3.020	C3.021	C3.024	C3.025	C3.026	C3.027	C3.030	C3.031	C3.032	C3.033	C3.034	C3.036	C3.037	C3.038	E1.006	E1.010	E1.012	E1.013	E1.014
Oral Ca	Oral Ca	Oral Ca	Oral Ca	Oral Ca	Oral Ca	Oral Ca	Oral Ca	Oral Ca	Oral Ca	Oral Ca	Oral Ca	Oral Ca	Oral Ca	Oral Ca	Oral Ca	Oral Ca	Oral Ca	Esophag	Esophag	Esophag	Esophag	Esophag
SP	SP	SP	SP	SP	SP	SP	SP	SP	SP	SP	SP	SP	SP	SP	SP	SP	SP	UE	UE	UE	UE	UE
0	0	0	0	0	0	0	0	0	0	0	0	0	0	0	0	0	0	0	0	0	0	0
0	0	0	0	0	0	2	0	0	0	0	0	0	0	0	0	0	0	0	0	3	0	0
0	0	0	0	0	0	0	0	0	0	0	0	0	0	0	0	0	0	0	0	0	0	0
1661	327	524	22	686	640	98	321	46	39	403	15	49	158	151	457	213	151	0	0	8	5	30
0	0	0	0	0	0	0	0	0	0	0	0	0	0	0	0	0	0	0	0	0	0	0
0	0	0	1	0	0	0	0	0	0	0	0	0	0	0	0	0	0	0	0	0	0	0
0	0	0	0	0	0	1	0	1	1	0	0	0	0	0	0	0	0	0	4	3	0	0
0	0	0	0	0	0	0	0	0	0	0	0	0	0	0	0	0	0	0	7	5	0	6
0	0	0	0	0	0	0	0	0	0	0	0	0	0	0	0	0	0	0	0	0	0	0
0	0	0	0	0	0	0	0	0	0	0	0	0	0	0	0	0	0	0	1	0	0	0
0	0	0	0	0	0	0	0	0	0	0	0	0	0	0	0	0	0	0	0	0	0	0
0	0	0	0	0	0	0	0	0	0	0	0	0	0	0	4	0	1	0	0	0	0	0
0	0	0	0	0	0	0	0	0	0	0	0	0	0	0	0	0	0	0	0	0	0	0
0	0	0	0	0	0	0	0	0	0	0	0	0	0	0	0	0	0	0	0	0	0	0
0	0	0	0	0	0	0	0	0	0	0	0	0	0	0	0	0	0	24	0	0	0	0
0	0	0	0	0	0	0	0	0	0	0	0	0	0	0	0	0	0	1	0	0	0	4
0	0	0	0	0	0	0	0	0	0	0	0	0	0	0	0	0	0	0	0	0	0	0
0	0	0	0	0	0	0	0	0	0	0	0	0	0	0	0	0	0	0	0	0	0	0
0	0	0	0	0	0	0	0	0	0	0	0	0	0	0	0	0	0	0	1	0	0	0
0	0	0	0	0	0	0	0	0	0	0	0	0	0	0	0	0	0	0	0	0	0	0
0	0	0	0	0	0	0	0	0	0	0	0	0	0	0	0	0	0	0	0	0	0	0
0	0	0	0	134	1	11	5	5	229	5	1	1	1	9	289	0	186	0	285	70	1	0
433	1363	746	16	581	1992	199	243	43	433	516	616	35	1063	1363	618	957	362	30	250	72	15	43
0	0	0	2	0	0	1	0	1	0	0	2	0	1	0	0	0	0	5	113	43	9	21
0	0	0	0	0	0	0	0	0	0	0	0	0	0	0	0	0	0	0	0	0	8	0
0	0	0	1	0	0	0	0	0	0	0	0	0	0	0	0	0	0	48	0	1	3	20
1	0	0	1	0	0	0	0	0	0	0	1	0	0	0	0	0	0	8	0	2	6	15
0	0	0	3	0	0	0	0	1	0	0	2	0	0	0	0	0	0	27	10	11	0	43
0	0	0	0	0	0	0	0	0	0	0	0	0	0	0	0	0	0	0	0	0	0	0
0	0	0	0	0	0	0	0	0	0	0	0	0	0	0	0	0	0	0	0	0	0	0
0	0	0	0	0	0	0	0	0	0	0	0	0	0	0	0	0	0	0	0	0	0	0
0	0	0	1	0	0	0	0	0	0	0	0	0	0	0	0	0	0	0	1	0	0	0
196	355	136	38	138	375	49	103	15	315	344	304	78	243	140	345	224	341	0	0	3	0	0
33	14	4	4	0	0	5	5	2	0	9	1	2	41	2	1	45	5	3	0	0	0	0
4866	3909	5039	4481	10	2440	793	757	195	774	2364	2463	2088	1016	1160	1927	184	2593	987	49	229	133	798
42	12	11	12	45	10	26	48	2	15	83	31	3	19	8	20	5	79	0	0	1	0	0
1730	2128	1656	89	23	1565	674	892	232	601	2113	1022	491	1313	1514	2192	1053	702	0	4	7	0	81

C3.016	C3.017	C3.018	C3.019	C3.020	C3.021	C3.024	C3.025	C3.026	C3.027	C3.030	C3.031	C3.032	C3.033	C3.034	C3.036	C3.037	C3.038	E1.006	E1.010	E1.012	E1.013	E1.014
Oral Ca	Oral Ca	Oral Ca	Oral Ca	Oral Ca	Oral Ca	Oral Ca	Oral Ca	Oral Ca	Oral Ca	Oral Ca	Oral Ca	Oral Ca	Oral Ca	Oral Ca	Oral Ca	Oral Ca	Oral Ca	Esophag	Esophag	Esophag	Esophag	Esophag
SP	SP	SP	SP	SP	SP	SP	SP	SP	SP	SP	SP	SP	SP	SP	SP	SP	SP	UE	UE	UE	UE	UE
0	0	0	0	0	0	0	0	0	0	0	0	0	0	0	0	0	0	0	0	0	0	0
0	0	0	0	0	0	0	0	0	0	0	0	0	0	0	0	0	0	0	0	0	0	0
0	0	0	0	0	0	0	0	0	0	0	0	0	0	0	0	0	0	0	0	1	0	0
0	1	1	0	3	6	0	2	1	2	34	1	8	1	4	30	0	0	0	0	0	0	0
0	0	0	0	0	0	0	0	0	0	0	0	0	0	0	0	0	2	0	0	1	0	0
0	0	0	0	0	0	0	0	0	0	0	0	0	0	0	0	0	0	0	0	1	15	2
0	0	0	0	0	0	0	0	1	0	0	0	0	0	0	0	0	0	0	0	4	0	0
0	0	0	0	0	0	0	0	0	0	0	0	0	0	0	0	0	0	0	0	0	0	0
0	0	0	0	0	0	0	0	0	0	0	0	0	0	0	0	0	0	0	1	0	0	0
0	0	5	0	30	20	1	0	1	0	0	15	6	1	53	29	0	1	0	0	0	0	0
0	0	48	0	0	0	0	0	0	0	0	23	0	0	1	0	0	0	0	0	0	0	0
0	0	0	0	0	0	0	0	0	0	0	0	0	0	0	0	0	0	0	0	0	0	0
0	0	44	0	1	10	0	2	8	5	0	44	0	0	34	0	0	0	0	2	0	43	6
0	0	0	0	0	0	0	0	0	0	0	0	0	0	0	0	0	0	0	2	2	0	10
0	0	0	0	0	0	0	0	0	0	0	0	0	0	0	0	0	0	0	1	0	0	0
0	0	0	0	0	0	0	0	0	0	0	0	0	0	0	0	0	0	0	0	0	0	0
0	0	0	0	0	0	0	0	0	0	0	0	0	0	0	0	0	0	0	0	0	0	0
0	0	0	0	0	0	0	0	0	0	0	0	0	0	0	0	0	0	0	0	0	0	0
0	0	0	0	0	0	0	0	0	0	0	0	0	0	0	0	0	0	0	0	0	3	0
0	0	0	0	0	0	0	0	0	0	0	0	0	0	0	0	0	0	0	0	0	0	0
132	296	343	112	91	415	412	273	297	171	501	270	136	345	636	389	375	213	1	0	14	176	74
0	0	0	0	0	0	0	2	7	0	9	0	1	2	6	110	0	0	0	0	0	0	0
0	0	0	0	0	0	0	0	0	0	0	1	0	0	1	0	0	0	1989	0	33	0	16
0	0	0	0	0	0	0	0	0	0	0	0	0	0	0	0	0	0	0	0	0	0	1
0	0	0	0	0	0	0	0	0	0	0	0	0	0	0	0	0	0	0	0	0	0	0
0	0	0	0	0	0	0	0	0	0	0	0	0	0	0	0	0	0	0	0	0	0	1
0	0	0	0	0	0	0	0	0	0	0	0	0	0	0	0	0	0	0	0	0	0	27
0	0	0	0	0	0	3	0	0	0	0	0	0	0	0	0	0	0	0	0	0	0	0
0	0	0	0	0	0	0	0	0	0	0	0	0	0	0	0	0	0	0	0	0	0	0
0	0	0	0	0	0	0	0	0	0	0	0	0	0	0	0	0	0	0	0	0	0	0
0	0	0	0	0	0	0	0	0	0	0	0	0	0	0	0	0	0	0	0	0	0	0
0	0	0	0	0	0	0	0	0	0	0	0	0	0	0	0	0	0	0	0	0	0	0
0	0	0	0	0	0	0	0	0	0	0	0	0	0	0	0	0	0	0	0	0	0	0
0	0	0	0	0	0	0	0	0	0	0	0	0	0	0	0	0	0	0	0	0	0	0
0	0	0	0	0	0	0	0	0	0	0	0	0	0	0	0	0	0	0	0	0	0	0
308	170	70	1	309	454	86	230	41	127	251	18	49	322	166	94	2	208	0	0	2	0	0
0	0	0	0	0	8	26	76	13	0	106	0	2	272	0	2	0	16	0	0	0	0	0

C3.016	C3.017	C3.018	C3.019	C3.020	C3.021	C3.024	C3.025	C3.026	C3.027	C3.030	C3.031	C3.032	C3.033	C3.034	C3.036	C3.037	C3.038	E1.006	E1.010	E1.012	E1.013	E1.014
Oral Ca	Oral Ca	Oral Ca	Oral Ca	Oral Ca	Oral Ca	Oral Ca	Oral Ca	Oral Ca	Oral Ca	Oral Ca	Oral Ca	Oral Ca	Oral Ca	Oral Ca	Oral Ca	Oral Ca	Oral Ca	Esophag	Esophag	Esophag	Esophag	Esophag
SP	SP	SP	SP	SP	SP	SP	SP	SP	SP	SP	SP	SP	SP	SP	SP	SP	SP	UE	UE	UE	UE	UE
142	0	0	0	0	54	0	48	0	0	15	0	0	0	0	0	0	0	0	0	0	0	0
0	0	0	0	0	0	0	0	0	0	0	0	0	0	0	0	0	0	0	0	0	0	0
0	0	0	0	0	0	0	0	0	0	0	0	0	0	0	0	0	0	0	1	1	0	0
0	0	0	0	0	0	0	0	0	0	0	0	0	0	0	0	0	0	0	1	0	0	0
0	0	0	0	0	0	0	0	0	0	0	0	0	0	0	0	0	0	0	0	0	0	0
0	0	0	0	0	0	0	0	0	0	0	0	0	0	0	0	0	0	0	0	0	0	0
0	0	0	0	0	0	0	0	0	0	0	0	0	0	0	0	0	0	0	0	0	0	0
0	0	0	0	0	0	0	0	0	0	0	0	0	0	0	0	0	0	0	0	0	0	0
0	0	0	0	0	0	0	0	0	0	0	0	0	0	0	0	0	0	0	0	0	0	0
0	0	0	0	0	0	0	0	0	0	0	0	0	0	0	0	0	0	0	0	0	0	0
0	0	0	0	0	0	0	0	0	0	0	0	0	0	0	0	0	0	0	0	1	3	4
28	0	0	9	2	1	20	8	51	16	0	0	0	0	0	0	0	0	193	319	212	100	1395
0	0	0	0	0	0	0	0	0	0	0	0	0	0	0	0	0	0	0	0	0	0	0
0	0	0	0	0	0	0	0	0	0	0	0	0	0	0	0	0	0	0	0	0	0	0
0	0	0	0	0	0	0	0	0	0	0	0	0	0	0	0	0	0	0	0	0	0	0
0	0	0	0	0	0	0	0	0	0	0	0	0	0	0	0	0	0	0	0	0	0	0
0	0	0	0	0	0	0	0	0	0	0	0	0	0	0	0	0	0	0	0	0	0	0
0	0	0	0	0	0	0	0	0	0	0	0	0	0	0	0	0	0	0	0	0	0	0
0	11	83	672	0	6	14	2	19	0	1	9	3	1	0	34	0	4	561	202	14	496	893
264	139	412	186	11	180	245	130	223	350	847	410	343	757	1126	2579	11	1153	64	1	6	17	26
634	97	83	747	11	29	21	43	22	22	228	11	76	77	68	361	4	157	1052	59	14	389	264
0	0	0	0	0	0	0	0	0	0	0	0	0	0	0	0	0	4	0	0	0	0	0
0	0	0	0	0	0	0	0	0	0	0	0	0	0	0	0	0	0	0	0	0	0	0
0	0	0	1	0	0	0	1	0	1	1	0	0	0	0	0	0	13	0	0	0	0	0
0	0	0	0	0	0	0	0	0	0	0	0	0	0	0	0	0	0	0	0	0	0	0
1	0	0	1	0	0	0	0	0	0	0	0	0	1	0	0	0	0	60	1	23	10	19
0	0	0	0	0	0	0	0	0	0	0	0	0	0	0	0	0	0	0	0	0	0	1
0	0	0	1	0	0	0	0	0	0	0	0	5	0	0	0	0	0	0	1	32	0	6
392	0	1486	9	0	0	0	289	1	3	156	5	433	4	7	676	0	542	35	1	0	0	25
0	0	0	0	0	0	0	0	0	0	0	0	0	0	0	0	0	0	0	0	0	0	0
0	0	0	0	0	0	0	0	0	0	0	0	0	0	0	0	0	0	0	0	1	0	0
0	0	0	0	0	0	0	0	0	0	0	0	0	0	0	0	0	0	0	0	0	0	1
0	0	0	0	0	0	0	0	0	0	0	0	0	0	0	0	0	0	0	0	4	0	2
0	0	0	27	2	1	16	4	12	1	0	2	0	0	0	0	0	0	183	25	230	1	83
0	0	0	0	0	0	0	0	1	0	0	0	0	0	0	0	0	0	1	0	3	0	0
0	0	0	6	1	0	0	0	2	0	0	1	0	0	0	0	0	0	85	12	15	0	17
0	0	0	0	0	0	0	0	0	0	0	0	0	0	0	0	0	0	0	0	1	0	0

C3.016	C3.017	C3.018	C3.019	C3.020	C3.021	C3.024	C3.025	C3.026	C3.027	C3.030	C3.031	C3.032	C3.033	C3.034	C3.036	C3.037	C3.038	E1.006	E1.010	E1.012	E1.013	E1.014
Oral Ca	Oral Ca	Oral Ca	Oral Ca	Oral Ca	Oral Ca	Oral Ca	Oral Ca	Oral Ca	Oral Ca	Oral Ca	Oral Ca	Oral Ca	Oral Ca	Oral Ca	Oral Ca	Oral Ca	Oral Ca	Esophag	Esophag	Esophag	Esophag	Esophag
SP	SP	SP	SP	SP	SP	SP	SP	SP	SP	SP	SP	SP	SP	SP	SP	SP	SP	UE	UE	UE	UE	UE
0	0	0	0	0	0	0	0	0	0	0	0	0	0	0	0	0	0	0	0	0	0	0
0	0	0	0	0	0	0	0	0	0	0	0	0	0	0	0	0	0	0	0	0	0	0
0	0	0	0	0	0	0	0	0	0	0	0	0	0	0	0	0	0	0	0	0	0	0
0	0	0	0	0	0	0	0	0	0	0	0	0	0	0	0	0	0	0	0	0	0	0
0	0	0	0	0	0	0	0	0	0	0	0	0	0	0	0	0	0	0	0	1	22	0
0	0	0	2	0	0	0	0	0	0	0	0	0	0	0	0	0	0	0	2	5	0	2
0	0	0	0	0	0	0	0	0	0	0	0	0	0	0	0	0	0	0	0	0	0	0
0	0	0	1	0	0	0	0	0	0	0	0	0	0	0	0	0	0	0	0	0	0	0
0	0	0	0	0	0	0	0	0	0	0	0	0	0	0	0	0	0	0	0	1	0	0
0	0	0	0	0	0	0	0	0	0	0	0	0	0	0	0	0	0	0	0	0	0	0
0	0	0	0	0	0	0	0	0	0	0	0	0	0	0	0	0	0	0	0	0	0	0
0	0	0	59	0	0	2	0	2	0	0	21	0	0	0	0	0	0	228	10	39	43	17
0	0	0	0	0	0	0	0	0	0	0	0	0	0	0	0	0	0	0	0	0	0	0
0	0	0	0	0	0	0	0	0	0	0	0	0	0	0	0	0	0	0	2	2	0	0
0	0	0	17	0	0	0	0	0	0	0	7	0	0	0	0	0	0	108	1	0	0	5
0	0	0	0	0	0	0	0	0	0	0	0	0	0	0	0	0	3	0	0	0	0	0
0	0	0	0	1	0	0	1	0	0	0	0	0	0	0	0	0	1	0	0	0	0	0
0	0	14	0	0	2	0	0	0	0	0	5	1	0	2	0	14	0	0	0	0	0	0
13	18	467	4	54	82	85	140	305	59	85	1567	104	127	356	208	556	270	1	1	0	182	0
0	0	4	0	0	1	0	0	0	0	0	0	1	0	0	0	4	0	0	0	0	2	0
1	0	33	0	5	14	2	5	3	21	1	61	7	3	77	14	18	6	0	0	0	10	0
0	0	0	0	0	0	0	0	0	0	0	0	0	0	0	0	0	0	0	0	0	0	0
0	0	0	0	0	0	0	0	0	0	0	0	0	0	0	0	0	0	0	0	0	0	0
0	0	0	0	0	0	0	7	0	0	15	271	28	0	14	0	82	1	0	0	0	0	0
3	0	118	0	0	0	4	7	8	1	0	108	2	4	3	20	52	4	0	4	4	23	9
0	0	0	0	0	0	0	0	0	0	0	0	0	0	0	0	0	0	0	0	0	0	0
0	0	0	0	0	0	0	0	0	0	0	0	0	0	0	0	0	0	0	0	0	0	0
0	0	0	0	0	0	0	0	0	0	0	0	0	0	0	0	0	0	0	0	0	0	0
0	0	0	7	1	0	14	0	1	0	11	5	6	7	41	0	6	8	0	0	2	18	0
0	0	0	0	0	0	13	4	4	72	76	38	5	11	6	3	1	13	0	2	3	6	0
0	0	0	0	0	0	0	0	0	0	0	0	0	0	0	0	0	0	0	0	0	0	0
0	0	0	2	0	0	0	0	0	0	0	0	0	0	0	0	0	0	0	0	0	20	37

E1.016	E1.017	E1.018	E1.020	E1.021	E1.024	E1.025	E1.026	E1.027	E1.029	E1.030	E1.031	E1.032	E1.033	E1.034	E1.036	E1.037	E1.038	E2.002	E2.003	E2.009	E2.010	E2.012
Esophag	Esophag	Esophag	Esophag	Esophag	Esophag	Esophag	Esophag	Esophag	Esophag	Esophag	Esophag	Esophag	Esophag	Esophag	Esophag	Esophag	Esophag	Esophag	Esophag	Esophag	Esophag	Esophag
UE	UE	UE	UE	UE	UE	UE	UE	UE	UE	UE	UE	UE	UE	UE	UE	UE	UE	ME	ME	ME	ME	ME
0	0	0	0	0	0	0	0	0	0	0	0	0	0	0	0	0	0	0	0	0	0	0
12557	19181	26355	8885	24478	7528	16969	13777	9254	11530	18723	23496	9799	24353	22943	17291	19697	16704	13331	31346	12584	8396	17967
46	4	39	0	1	14	237	51	84	43	0	0	0	1	0	2	1	0	14	0	6	32	363
0	0	62	0	0	0	3	12	8	1	0	0	0	1	11	0	0	0	0	0	0	0	7
0	0	3	0	0	0	24	9	9	12	0	6	0	0	0	0	0	7	0	0	7	6	60
502	131	874	153	617	160	194	763	190	144	803	680	242	738	222	19	328	350	560	203	308	127	1025
0	7	2	0	0	3	1	1	4	7	0	2	0	0	18	0	1	4	10	0	1	0	20
2648	694	2910	2865	6485	1427	1182	487	1339	983	1570	5429	1730	2666	3489	910	872	2301	1831	4425	1049	345	2005
0	0	0	0	0	0	0	0	0	0	0	0	0	0	0	0	0	0	0	0	0	0	0
0	0	0	0	0	0	0	0	0	1	0	0	0	3	0	0	0	0	0	0	0	0	5
0	0	0	0	0	0	0	0	0	0	0	0	0	0	0	0	0	0	0	0	0	0	0
0	0	6	0	0	0	0	0	0	0	0	0	0	15	4	0	0	0	0	0	0	0	1
4	27	5	3	5	0	2	1	0	2	0	0	0	4	0	1	0	0	4	2	0	1	4
0	0	3	0	0	0	1	0	0	0	0	0	0	0	0	0	0	0	0	0	0	0	1
0	0	0	0	0	1	0	0	0	0	0	0	0	0	0	0	0	0	0	0	0	0	0
4194	3905	13303	4417	5271	2149	1615	2374	3634	6238	7152	12309	4007	12062	7173	1584	17392	6196	4073	18199	5391	537	3463
217	69	116	80	585	131	141	150	151	163	475	268	99	825	283	16	125	84	598	425	90	58	112
0	0	3	0	0	0	0	3	0	0	0	0	0	0	0	0	0	0	0	0	0	0	0
0	0	0	0	0	12	54	9	14	1	139	74	113	37	48	1	0	9	1	6	10	2	11
0	0	0	0	0	0	0	0	0	0	0	0	0	0	0	0	0	0	0	0	0	0	0
0	0	0	0	0	0	0	0	0	0	0	0	0	0	0	0	0	0	0	0	0	0	0
4847	14270	8545	1333	11360	3432	13214	9718	3110	3780	8552	4394	3468	7447	11543	14589	804	7604	6044	7699	5514	7235	10360
0	6	95	10	10	37	17	19	32	15	2	153	0	36	68	3	25	7	27	34	52	3	4
0	0	0	0	5	30	4	2	27	9	0	20	42	0	4	3	0	0	6	60	0	3	1
0	0	0	0	2	2	0	0	2	0	1	0	0	4	0	0	2	0	1	1	7	1	1
0	9	26	0	30	8	18	26	39	11	7	0	94	0	0	0	9	8	24	73	81	21	92
0	0	0	0	0	0	0	0	0	0	0	0	0	0	0	0	0	0	0	0	0	0	0
0	0	0	0	0	0	0	0	1	0	0	0	0	0	0	0	0	0	0	0	0	0	0
73	26	226	7	100	122	262	152	610	119	22	161	0	514	80	163	138	112	138	219	67	24	432
26	33	137	17	7	0	0	0	0	0	0	0	0	0	0	0	0	22	0	0	1	0	0
0	0	0	0	0	0	0	0	0	1	0	0	4	0	0	0	0	0	0	0	0	1	0
0	0	0	0	0	0	0	0	0	0	0	0	0	0	0	0	0	0	0	0	0	0	0
0	0	62	0	0	0	3	12	8	1	0	0	0	1	11	0	0	0	0	0	0	0	7
0	0	3	0	0	0	0	0	0	0	0	0	0	0	0	0	0	0	0	0	0	0	0
0	0	0	0	0	0	0	0	0	0	0	6	0	0	0	0	0	0	0	0	0	0	0
0	0	0	0	0	0	0	0	0	0	0	0	0	0	0	0	0	0	0	0	0	0	2

E1.016	E1.017	E1.018	E1.020	E1.021	E1.024	E1.025	E1.026	E1.027	E1.029	E1.030	E1.031	E1.032	E1.033	E1.034	E1.036	E1.037	E1.038	E2.002	E2.003	E2.009	E2.010	E2.012
Esophag	Esophag	Esophag	Esophag	Esophag	Esophag	Esophag	Esophag	Esophag	Esophag	Esophag	Esophag	Esophag	Esophag	Esophag	Esophag	Esophag	Esophag	Esophag	Esophag	Esophag	Esophag	Esophag
UE	UE	UE	UE	UE	UE	UE	UE	UE	UE	UE	UE	UE	UE	UE	UE	UE	UE	ME	ME	ME	ME	ME
0	0	0	0	0	0	0	0	0	0	0	0	0	0	0	0	0	0	0	0	0	0	0
0	0	0	0	0	0	24	9	9	12	0	0	0	0	0	0	0	7	0	0	7	6	58
0	0	0	0	0	0	0	0	0	0	0	0	0	0	0	0	0	0	0	0	0	0	0
0	0	3	0	0	0	0	0	0	0	0	0	0	0	0	0	0	0	0	0	0	0	0
495	125	858	153	538	157	182	671	168	131	803	680	234	724	222	18	320	349	537	198	298	124	912
7	6	9	0	79	3	12	92	22	13	0	0	8	14	0	1	8	1	23	5	10	3	113
0	0	0	0	0	0	0	0	0	0	0	0	0	0	0	0	0	0	0	0	0	0	0
0	0	4	0	0	0	0	0	0	0	0	0	0	0	0	0	0	0	0	0	0	0	0
0	0	0	0	0	0	0	0	0	0	0	0	0	0	0	0	0	0	0	0	0	0	0
0	7	2	0	0	3	1	1	4	7	0	2	0	0	0	0	0	4	10	0	1	0	20
0	0	0	0	0	0	0	0	0	0	0	0	0	0	18	0	1	0	0	0	0	0	0
48	27	79	25	14	23	55	15	18	38	6	60	0	45	165	0	6	91	65	8	41	17	121
2557	615	2581	2821	6197	1288	1024	390	1223	671	1359	5060	1620	1906	767	871	801	1498	1550	4163	761	260	1783
26	0	60	0	0	1	4	0	4	0	0	0	0	5	26	0	0	7	18	0	0	1	5
9	41	171	19	273	115	93	82	93	254	205	309	110	545	312	39	63	190	198	254	247	66	91
8	11	19	0	1	0	6	0	1	20	0	0	0	165	2219	0	2	515	0	0	0	1	5
0	0	0	0	0	0	0	0	0	0	0	0	0	0	0	0	0	0	0	0	0	0	0
0	0	0	0	0	0	0	0	0	1	0	0	0	3	0	0	0	0	0	0	0	0	5
0	0	0	0	0	0	0	0	0	0	0	0	0	0	0	0	0	0	0	0	0	0	0
0	0	0	0	0	0	0	0	0	0	0	0	0	0	0	0	0	0	0	0	0	0	0
0	0	0	0	0	0	0	0	0	0	0	0	0	0	0	0	0	0	0	0	0	0	1
0	0	0	0	0	0	0	0	0	0	0	0	0	0	0	0	0	0	0	0	0	0	0
0	0	6	0	0	0	0	0	0	0	0	0	0	0	0	0	0	0	0	0	0	0	0
0	0	0	0	0	0	0	0	0	0	0	0	0	0	0	0	0	0	0	0	0	0	0
0	0	0	0	0	0	0	0	0	0	0	0	0	15	4	0	0	0	0	0	0	0	0
0	0	0	0	0	0	0	0	0	0	0	0	0	0	0	0	0	0	0	0	0	0	0
4	0	2	0	0	0	1	0	0	0	0	0	0	0	0	1	0	0	1	0	0	0	1
0	27	3	3	5	0	1	0	0	2	0	0	0	4	0	0	0	0	3	2	0	1	3
0	0	0	0	0	0	0	0	0	0	0	0	0	0	0	0	0	0	0	0	0	0	0
0	0	0	0	0	0	0	1	0	0	0	0	0	0	0	0	0	0	0	0	0	0	0
0	0	3	0	0	0	1	0	0	0	0	0	0	0	0	0	0	0	0	0	0	0	1
0	0	0	0	0	0	0	0	0	0	0	0	0	0	0	0	0	0	0	0	0	0	0
0	0	0	0	0	1	0	0	0	0	0	0	0	0	0	0	0	0	0	0	0	0	0
2670	3317	11642	3132	3122	1724	1246	2078	3201	5832	6919	11211	3792	11219	6549	1501	16852	5497	3139	17499	4620	373	2069
1467	556	1515	1268	2120	408	314	271	402	391	233	1086	215	707	604	81	530	648	889	665	731	148	1302
57	32	146	17	29	17	54	25	31	15	0	12	0	136	20	2	10	51	45	35	40	16	91
217	69	116	80	585	131	141	150	151	163	475	268	99	825	283	16	125	84	598	425	90	58	112

E1.016	E1.017	E1.018	E1.020	E1.021	E1.024	E1.025	E1.026	E1.027	E1.029	E1.030	E1.031	E1.032	E1.033	E1.034	E1.036	E1.037	E1.038	E2.002	E2.003	E2.009	E2.010	E2.012
Esophag	Esophag	Esophag	Esophag	Esophag	Esophag	Esophag	Esophag	Esophag	Esophag	Esophag	Esophag	Esophag	Esophag	Esophag	Esophag	Esophag	Esophag	Esophag	Esophag	Esophag	Esophag	Esophag
UE	UE	UE	UE	UE	UE	UE	UE	UE	UE	UE	UE	UE	UE	UE	UE	UE	UE	ME	ME	ME	ME	ME
0	0	3	0	0	0	0	3	0	0	0	0	0	0	0	0	0	0	0	0	0	0	0
0	0	0	0	0	12	54	9	14	1	139	74	113	37	48	1	0	9	1	6	10	2	11
0	0	0	0	0	0	0	0	0	0	0	0	0	0	0	0	0	0	0	0	0	0	0
0	0	0	0	0	0	0	0	0	0	0	0	0	0	0	0	0	0	0	0	0	0	0
0	0	0	0	0	0	0	0	0	0	0	0	0	0	0	0	0	0	0	0	0	0	0
0	0	0	0	0	0	0	0	0	0	0	0	0	0	0	0	0	0	0	0	0	0	0
694	366	2937	433	84	338	10653	7621	1324	350	286	476	795	602	1428	64	126	955	861	250	366	5565	6965
1224	679	2660	195	1372	916	556	470	402	571	830	1573	486	2564	1566	331	229	1300	1731	1386	1550	534	1536
79	8	118	0	3	3	8	1	10	3	2	24	0	1	0	0	5	7	12	4	14	4	14
13	193	114	189	641	55	73	111	98	54	95	1140	216	95	199	0	51	37	80	155	57	7	150
2837	13024	2716	516	9260	2120	1923	1515	1276	2802	7339	1181	1971	4185	8350	14194	393	5305	3360	5904	3527	1125	1695
0	0	0	0	0	0	0	0	0	0	0	0	0	0	0	0	0	0	0	0	0	0	0
0	0	0	0	0	0	0	0	0	0	0	0	0	0	0	0	0	0	0	0	0	0	0
0	6	95	10	10	37	17	19	32	15	2	153	0	36	68	3	25	7	27	34	52	3	4
0	0	0	0	2	2	0	0	2	0	1	0	0	4	0	0	2	0	1	1	7	1	1
0	0	0	0	0	0	0	0	0	0	0	0	0	0	0	0	0	0	0	0	0	0	0
0	9	22	0	30	8	18	25	39	11	7	0	94	0	0	0	9	8	24	64	79	21	92
0	0	4	0	0	0	0	1	0	0	0	0	0	0	0	0	0	0	0	9	2	0	0
0	0	0	0	0	0	0	0	0	0	0	0	0	0	0	0	0	0	0	0	0	0	0
0	0	0	0	0	0	0	0	1	0	0	0	0	0	0	0	0	0	0	0	0	0	0
0	0	0	0	0	0	0	0	0	0	0	0	0	0	0	0	0	0	0	0	0	0	0
0	0	0	0	0	0	0	0	0	0	0	0	0	0	0	0	0	0	0	0	0	0	0
0	0	8	0	0	0	0	0	0	0	0	0	0	0	0	0	0	0	0	0	0	0	1
73	26	218	7	100	122	262	152	610	119	22	161	0	514	80	163	138	112	138	219	67	24	431
0	0	0	0	0	0	0	0	0	0	0	0	0	0	0	0	0	0	0	0	0	0	0
26	33	137	17	7	0	0	0	0	0	0	0	0	0	0	0	0	22	0	0	1	0	0
0	0	0	0	0	0	0	0	0	0	0	0	0	0	0	0	0	0	0	0	0	0	0
0	0	62	0	0	0	3	12	8	1	0	0	0	1	11	0	0	0	0	0	0	0	6
0	0	0	0	0	0	0	0	0	0	0	0	0	0	0	0	0	0	0	0	0	0	1
0	0	3	0	0	0	0	0	0	0	0	0	0	0	0	0	0	0	0	0	0	0	0
0	0	0	0	0	0	0	0	0	0	0	6	0	0	0	0	0	0	0	0	0	0	0
0	0	0	0	0	0	0	0	0	0	0	0	0	0	0	0	0	0	0	0	0	0	2
0	0	0	0	0	0	0	0	0	0	0	0	0	0	0	0	0	0	0	0	0	0	0
0	0	0	0	0	0	24	9	9	12	0	0	0	0	0	0	0	7	0	0	7	6	58
0	0	0	0	0	0	0	0	0	0	0	0	0	0	0	0	0	0	0	0	0	0	0
0	0	3	0	0	0	0	0	0	0	0	0	0	0	0	0	0	0	0	0	0	0	0
494	124	850	153	536	66	121	41	65	86	791	680	234	724	218	18	319	304	523	195	246	26	309

E1.016	E1.017	E1.018	E1.020	E1.021	E1.024	E1.025	E1.026	E1.027	E1.029	E1.030	E1.031	E1.032	E1.033	E1.034	E1.036	E1.037	E1.038	E2.002	E2.003	E2.009	E2.010	E2.012
Esophag	Esophag	Esophag	Esophag	Esophag	Esophag	Esophag	Esophag	Esophag	Esophag	Esophag	Esophag	Esophag	Esophag	Esophag	Esophag	Esophag	Esophag	Esophag	Esophag	Esophag	Esophag	Esophag
UE	UE	UE	UE	UE	UE	UE	UE	UE	UE	UE	UE	UE	UE	UE	UE	UE	UE	ME	ME	ME	ME	ME
1	1	8	0	2	91	61	630	103	45	12	0	0	0	4	0	1	45	14	3	52	98	603
0	0	0	0	0	0	0	0	0	0	0	0	0	0	0	0	0	0	0	0	0	0	0
7	6	9	0	79	3	12	92	22	13	0	0	8	14	0	1	8	1	23	5	10	3	113
0	0	0	0	0	0	0	0	0	0	0	0	0	0	0	0	0	0	0	0	0	0	0
0	0	4	0	0	0	0	0	0	0	0	0	0	0	0	0	0	0	0	0	0	0	0
0	0	0	0	0	0	0	0	0	0	0	0	0	0	0	0	0	0	0	0	0	0	0
0	0	0	0	0	0	0	0	0	0	0	0	0	0	0	0	0	0	0	0	0	0	0
0	7	2	0	0	3	1	1	4	7	0	2	0	0	0	0	0	4	10	0	1	0	20
0	0	0	0	0	0	0	0	0	0	0	0	0	0	18	0	1	0	0	0	0	0	0
48	27	79	25	14	23	55	15	18	38	6	60	0	45	165	0	6	91	65	8	41	17	121
2557	615	2581	2821	6197	1288	1024	390	1223	671	1359	5060	1620	1906	767	871	801	1498	1550	4163	761	260	1783
26	0	60	0	0	1	4	0	4	0	0	0	0	5	26	0	0	7	18	0	0	1	5
9	41	171	19	273	115	93	82	93	254	205	309	110	545	312	39	63	190	198	254	247	66	91
8	11	19	0	1	0	6	0	1	20	0	0	0	165	2219	0	2	515	0	0	0	1	5
0	0	0	0	0	0	0	0	0	1	0	0	0	3	0	0	0	0	0	0	0	0	5
0	0	0	0	0	0	0	0	0	0	0	0	0	0	0	0	0	0	0	0	0	0	0
0	0	0	0	0	0	0	0	0	0	0	0	0	0	0	0	0	0	0	0	0	0	0
0	0	0	0	0	0	0	0	0	0	0	0	0	0	0	0	0	0	0	0	0	0	1
0	0	0	0	0	0	0	0	0	0	0	0	0	0	0	0	0	0	0	0	0	0	0
0	0	0	0	0	0	0	0	0	0	0	0	0	15	4	0	0	0	0	0	0	0	0
0	0	0	0	0	0	0	0	0	0	0	0	0	0	0	0	0	0	0	0	0	0	0
4	0	2	0	0	0	1	0	0	0	0	0	0	0	0	1	0	0	1	0	0	0	1
0	0	0	0	0	0	0	0	0	0	0	0	0	0	0	0	0	0	0	0	0	0	0
0	0	0	0	0	0	0	0	0	0	0	0	0	0	0	0	0	0	0	0	0	0	0
0	0	0	0	3	0	0	0	0	0	0	0	0	0	0	0	0	0	0	0	0	0	0
0	27	3	3	2	0	1	0	0	2	0	0	0	4	0	0	0	0	3	2	0	1	3
0	0	0	0	0	0	0	1	0	0	0	0	0	0	0	0	0	0	0	0	0	0	0
0	0	0	0	0	0	0	0	0	0	0	0	0	0	0	0	0	0	0	0	0	0	0
0	0	3	0	0	0	1	0	0	0	0	0	0	0	0	0	0	0	0	0	0	0	1
0	0	0	0	0	1	0	0	0	0	0	0	0	0	0	0	0	0	0	0	0	0	0
62	67	317	72	0	12	53	18	52	15	1	103	85	58	1087	2	0	342	83	3	153	30	254
263	96	451	487	432	165	140	62	99	2559	1531	885	612	1014	706	570	930	529	187	1964	676	29	230
2345	3154	10874	2573	2690	1547	1051	1997	3050	3255	5387	10223	3095	10147	4756	929	15922	4626	2869	15532	3790	314	1584
0	0	0	0	0	0	1	1	0	3	0	0	0	0	0	0	0	0	0	0	1	0	1
1467	556	1515	1268	2120	408	314	271	402	391	233	1086	215	707	604	81	530	648	889	665	731	148	1302
0	0	0	0	0	0	0	0	0	0	0	0	0	0	0	0	0	0	0	0	0	0	0
0	0	0	0	0	0	0	0	0	0	0	0	0	0	0	0	0	0	0	0	0	0	0

E1.016	E1.017	E1.018	E1.020	E1.021	E1.024	E1.025	E1.026	E1.027	E1.029	E1.030	E1.031	E1.032	E1.033	E1.034	E1.036	E1.037	E1.038	E2.002	E2.003	E2.009	E2.010	E2.012
Esophag	Esophag	Esophag	Esophag	Esophag	Esophag	Esophag	Esophag	Esophag	Esophag	Esophag	Esophag	Esophag	Esophag	Esophag	Esophag	Esophag	Esophag	Esophag	Esophag	Esophag	Esophag	Esophag
UE	UE	UE	UE	UE	UE	UE	UE	UE	UE	UE	UE	UE	UE	UE	UE	UE	UE	ME	ME	ME	ME	ME
57	32	146	17	29	17	54	25	31	15	0	12	0	136	20	2	10	51	45	35	40	16	91
217	69	116	80	585	131	141	150	151	163	475	268	99	825	283	16	125	84	598	425	90	58	112
0	0	3	0	0	0	0	3	0	0	0	0	0	0	0	0	0	0	0	0	0	0	0
0	0	0	0	0	0	0	0	0	0	0	0	0	0	0	0	0	0	0	0	0	0	0
0	0	0	0	0	0	0	0	0	0	0	0	0	0	0	0	0	0	0	0	0	0	0
0	0	0	0	0	0	0	0	0	0	0	0	0	0	0	0	0	0	0	0	0	0	0
0	0	0	0	0	0	0	0	0	0	0	0	0	0	0	0	0	0	0	0	0	0	0
0	0	0	0	0	0	0	0	0	0	0	0	0	0	1	0	0	4	0	0	0	0	0
507	161	721	225	43	42	504	174	104	131	108	110	295	127	650	28	71	454	280	113	153	271	872
0	0	0	0	0	0	0	0	0	0	0	0	0	0	0	0	0	0	0	0	0	0	0
0	0	0	0	0	0	0	0	0	0	0	0	0	0	0	0	0	0	0	0	1	0	0
129	76	212	109	20	20	360	166	50	68	89	200	379	156	397	26	23	272	378	90	105	123	372
19	0	85	10	0	1	2	9	9	2	0	0	0	201	0	0	0	36	0	0	2	3	16
9	12	533	0	4	4	29	21	17	13	17	9	21	1	47	0	0	18	41	2	61	14	225
0	0	0	0	0	0	1	0	4	4	0	0	0	0	0	0	0	0	0	0	0	0	1
30	117	1384	89	17	271	9757	7251	1140	131	72	128	100	117	333	10	32	171	162	45	43	5154	5478
0	0	0	0	0	0	0	0	0	0	0	0	0	0	0	0	0	0	0	0	0	0	0
54	77	1024	96	51	110	114	114	87	126	108	203	223	264	1071	23	49	459	222	44	369	56	212
4	0	3	0	0	0	1	1	2	0	0	0	0	0	0	2	0	0	0	0	0	0	3
0	0	0	0	0	0	0	0	0	0	0	0	0	0	0	0	0	0	0	0	0	0	0
1166	602	1614	94	1320	806	435	354	313	444	722	1370	263	2300	480	303	179	829	1491	1342	1180	477	1300
0	0	19	5	1	0	3	0	0	1	0	0	0	0	15	3	1	12	18	0	1	1	15
0	0	0	0	0	0	0	0	0	0	0	0	0	0	0	0	0	0	0	0	0	0	0
0	0	0	0	0	0	0	0	0	0	0	0	0	0	0	0	0	0	0	0	0	0	0
0	0	66	0	0	0	3	0	0	0	2	0	0	0	0	0	1	0	0	0	0	0	3
0	0	0	0	3	0	0	0	0	0	0	0	0	0	0	0	0	0	7	3	6	0	0
79	8	31	0	0	2	1	1	3	2	0	0	0	0	0	0	4	7	5	1	6	4	3
0	0	0	0	0	0	0	0	0	0	0	0	0	0	0	0	0	0	0	0	0	0	0
0	0	0	0	0	1	0	0	6	0	0	24	0	0	0	0	0	0	0	0	1	0	0
0	0	0	0	0	0	0	0	0	0	0	0	0	0	0	0	0	0	0	0	0	0	2
0	0	21	0	0	0	4	0	1	1	0	0	0	1	0	0	0	0	0	0	1	0	6
0	0	0	0	0	0	0	0	0	0	0	0	0	0	0	0	0	0	0	0	0	0	0
0	0	0	0	0	0	0	0	0	0	0	0	0	0	0	0	0	0	0	0	0	0	0
13	193	114	189	641	55	73	111	98	54	95	1140	216	95	199	0	51	37	80	155	57	7	150
0	2	7	1	0	3	6	0	0	2	0	0	0	0	12	0	0	0	11	0	2	3	3
0	0	0	0	0	0	6	1	0	1	0	0	0	0	0	0	0	0	0	0	0	0	4
0	0	1	0	3	2	1	3	16	6	0	0	0	21	0	0	0	8	0	1	8	0	4

E1.016	E1.017	E1.018	E1.020	E1.021	E1.024	E1.025	E1.026	E1.027	E1.029	E1.030	E1.031	E1.032	E1.033	E1.034	E1.036	E1.037	E1.038	E2.002	E2.003	E2.009	E2.010	E2.012
Esophag	Esophag	Esophag	Esophag	Esophag	Esophag	Esophag	Esophag	Esophag	Esophag	Esophag	Esophag	Esophag	Esophag	Esophag	Esophag	Esophag	Esophag	Esophag	Esophag	Esophag	Esophag	Esophag
UE	UE	UE	UE	UE	UE	UE	UE	UE	UE	UE	UE	UE	UE	UE	UE	UE	UE	ME	ME	ME	ME	ME
1229	93	1109	114	26	67	710	557	190	217	71	29	595	108	210	16	198	248	2254	49	259	425	797
2	0	6	0	3	0	0	0	0	0	0	0	0	0	0	3	0	0	0	0	0	0	1
0	0	0	0	0	1	0	0	0	0	0	0	0	0	8	0	0	0	0	0	0	0	2
1382	12872	1402	304	9221	1984	532	562	876	2224	7222	1012	1184	3840	592	14123	177	2704	824	5707	3004	411	329
168	36	155	90	3	48	454	238	167	329	25	96	129	19	1164	42	7	631	229	140	148	205	371
0	0	0	0	0	0	0	0	0	0	0	0	0	0	0	0	0	0	0	0	0	0	0
0	0	0	0	0	0	0	0	0	0	0	0	0	0	0	0	0	0	0	0	0	0	0
56	21	36	7	4	15	211	154	27	23	21	44	63	197	6364	10	11	1714	42	7	106	80	183
0	0	0	0	0	0	0	0	0	0	0	0	0	0	0	0	0	0	0	0	0	0	0
0	0	0	0	0	0	0	0	0	0	0	0	0	0	0	0	0	0	0	0	0	0	0
0	0	0	0	0	0	0	0	0	0	0	0	0	0	0	0	0	0	0	0	0	0	0
0	6	95	10	10	37	17	19	32	15	2	153	0	36	68	3	25	7	27	34	52	3	4
0	0	0	0	2	2	0	0	2	0	1	0	0	4	0	0	2	0	1	1	7	1	1
0	9	0	0	0	0	3	0	0	0	5	0	0	0	0	0	4	8	0	5	68	0	0
0	0	4	0	30	7	4	22	36	11	0	0	94	0	0	0	5	0	24	33	7	17	91
0	0	18	0	0	1	11	3	3	0	2	0	0	0	0	0	0	0	0	26	4	4	1
0	0	4	0	0	0	0	1	0	0	0	0	0	0	0	0	0	0	0	9	2	0	0
0	0	0	0	0	0	0	0	0	0	0	0	0	0	0	0	0	0	0	0	0	0	0
0	0	0	0	0	0	0	0	0	0	0	0	0	0	0	0	0	0	0	0	0	0	0
26	0	0	3	73	3	79	9	23	2	0	54	0	4	43	39	1	27	0	112	28	2	12
0	0	9	0	0	0	0	0	0	0	0	0	0	32	0	0	0	0	12	0	0	1	1
23	0	31	0	0	6	27	0	141	1	5	48	0	1	0	0	7	0	0	0	2	1	343
0	0	0	0	0	0	0	0	0	0	0	0	0	0	0	0	0	0	0	0	0	0	0
26	33	137	17	7	0	0	0	0	0	0	0	0	0	0	0	0	22	0	0	1	0	0
0	0	0	0	0	0	0	0	0	0	0	0	0	0	0	0	0	0	0	0	0	0	0
0	0	38	0	0	0	3	4	8	1	0	0	0	1	11	0	0	0	0	0	0	0	3
0	0	24	0	0	0	0	8	0	0	0	0	0	0	0	0	0	0	0	0	0	0	3
0	0	0	0	0	0	0	0	0	0	0	0	0	0	0	0	0	0	0	0	0	0	1
0	0	3	0	0	0	0	0	0	0	0	0	0	0	0	0	0	0	0	0	0	0	0
0	0	0	0	0	0	0	0	0	0	0	6	0	0	0	0	0	0	0	0	0	0	0
0	0	0	0	0	0	0	0	0	0	0	0	0	0	0	0	0	0	0	0	0	0	2
0	0	0	0	0	0	0	0	0	0	0	0	0	0	0	0	0	0	0	0	0	0	0
0	0	3	0	0	0	0	0	0	0	0	0	0	0	0	0	0	0	0	0	0	0	0
0	0	0	0	0	0	0	0	0	0	0	0	0	0	0	0	0	0	0	0	0	0	3
190	20	223	33	409	28	25	13	34	26	1	9	36	87	16	6	136	58	156	105	119	5	128
0	0	0	0	0	0	0	0	0	0	0	0	0	9	0	0	0	0	0	0	0	0	0
5	0	3	0	0	0	0	0	0	0	0	0	0	0	0	0	0	0	0	0	0	0	0

E1.016	E1.017	E1.018	E1.020	E1.021	E1.024	E1.025	E1.026	E1.027	E1.029	E1.030	E1.031	E1.032	E1.033	E1.034	E1.036	E1.037	E1.038	E2.002	E2.003	E2.009	E2.010	E2.012
Esophag	Esophag	Esophag	Esophag	Esophag	Esophag	Esophag	Esophag	Esophag	Esophag	Esophag	Esophag	Esophag	Esophag	Esophag	Esophag	Esophag	Esophag	Esophag	Esophag	Esophag	Esophag	Esophag
UE	UE	UE	UE	UE	UE	UE	UE	UE	UE	UE	UE	UE	UE	UE	UE	UE	UE	ME	ME	ME	ME	ME
67	1	48	16	5	7	4	2	9	14	7	0	0	23	19	0	1	16	44	5	8	0	31
0	0	0	0	0	0	0	0	0	0	0	0	0	0	0	0	0	0	0	0	0	0	0
0	0	0	0	0	0	0	0	0	0	0	0	0	0	0	0	0	3	0	0	0	0	0
0	0	0	0	0	0	0	0	0	0	0	0	0	1	0	0	0	0	0	0	0	0	0
0	0	0	0	0	0	0	0	0	0	0	0	0	0	0	0	0	0	0	0	0	0	0
0	0	0	0	0	0	0	9	0	0	0	0	0	0	0	0	0	0	0	0	0	0	0
0	0	33	0	0	0	47	1	0	0	0	0	12	0	0	0	0	0	0	0	0	0	3
0	0	0	0	0	0	0	0	0	0	0	0	0	0	0	0	0	8	0	0	0	0	0
22	7	17	0	0	0	1	0	1	1	3	5	1	47	7	0	0	0	33	0	0	0	4
0	0	0	0	0	0	0	0	0	0	0	0	0	0	0	0	0	0	0	0	0	0	0
0	0	0	0	0	0	0	0	0	0	0	0	0	0	0	0	0	0	0	0	0	0	0
12	0	28	0	3	2	3	1	3	7	0	6	0	30	3	0	1	1	0	1	1	1	11
83	88	77	68	110	26	22	4	5	19	752	640	102	301	119	8	176	193	137	62	88	5	64
1	0	12	0	0	0	0	0	0	0	0	0	0	0	0	0	0	0	0	0	0	0	3
0	0	0	0	0	0	0	0	0	1	0	0	0	0	0	0	0	0	0	0	0	0	0
9	0	0	0	0	1	14	7	2	0	0	0	0	11	10	0	0	1	0	0	15	10	13
0	0	181	0	0	0	0	0	0	0	0	0	0	151	13	0	0	0	0	0	0	0	3
0	7	0	0	0	0	0	0	0	0	0	0	0	0	0	0	0	0	0	0	0	0	0
0	0	0	0	0	0	0	0	0	0	0	0	0	0	0	0	0	0	0	0	0	0	0
91	1	125	18	9	2	5	4	11	17	28	20	83	49	29	3	5	22	97	20	15	3	44
0	0	0	0	0	0	0	0	0	0	0	0	0	0	0	0	0	0	0	0	0	2	1
0	0	7	16	0	0	0	0	0	0	0	0	0	0	0	0	0	0	0	0	0	0	0
0	0	0	0	0	0	0	0	0	0	0	0	0	0	0	0	0	0	0	0	0	0	0
14	0	14	2	0	0	0	0	0	1	0	0	0	0	2	1	0	2	54	2	0	0	1
0	0	0	0	0	0	0	0	0	0	0	0	0	0	0	0	0	0	0	0	0	0	0
0	0	0	0	0	0	0	0	0	0	0	0	0	0	0	0	0	0	2	0	0	0	0
1	1	8	0	2	91	61	630	103	45	12	0	0	0	4	0	1	45	14	3	52	98	603
7	6	9	0	79	3	12	92	22	13	0	0	8	14	0	1	8	1	23	5	10	3	113
0	0	0	0	0	0	0	0	0	0	0	0	0	0	0	0	0	0	0	0	0	0	0
0	0	4	0	0	0	0	0	0	0	0	0	0	0	0	0	0	0	0	0	0	0	0
0	0	0	0	0	0	0	0	0	0	0	0	0	0	0	0	0	0	0	0	0	0	0
0	0	0	0	0	0	0	0	0	0	0	0	0	0	0	0	0	0	0	0	0	0	0
0	7	2	0	0	3	1	1	4	7	0	2	0	0	0	0	0	4	10	0	1	0	20
0	0	0	0	0	0	0	0	0	0	0	0	0	0	18	0	1	0	0	0	0	0	0
48	27	79	25	14	23	55	15	18	38	6	60	0	45	165	0	6	91	65	8	41	17	121
0	0	0	0	0	0	0	0	0	0	0	0	0	0	0	0	0	0	0	0	0	0	0
0	0	0	0	0	0	0	0	0	0	0	0	0	0	0	0	0	0	0	0	0	0	0

E1.016	E1.017	E1.018	E1.020	E1.021	E1.024	E1.025	E1.026	E1.027	E1.029	E1.030	E1.031	E1.032	E1.033	E1.034	E1.036	E1.037	E1.038	E2.002	E2.003	E2.009	E2.010	E2.012
Esophag	Esophag	Esophag	Esophag	Esophag	Esophag	Esophag	Esophag	Esophag	Esophag	Esophag	Esophag	Esophag	Esophag	Esophag	Esophag	Esophag	Esophag	Esophag	Esophag	Esophag	Esophag	Esophag
UE	UE	UE	UE	UE	UE	UE	UE	UE	UE	UE	UE	UE	UE	UE	UE	UE	UE	ME	ME	ME	ME	ME
0	0	4	0	0	0	4	0	0	1	0	0	0	0	0	0	0	0	0	0	4	0	0
1288	205	748	664	1660	682	601	89	640	147	229	2221	1222	488	205	618	183	650	382	3068	176	139	777
184	51	81	102	10	6	21	5	16	28	0	4	0	14	0	9	6	53	9	9	13	9	40
0	0	0	0	0	0	0	0	39	0	0	0	0	0	8	0	0	0	0	8	0	1	1
0	0	0	1	0	0	0	0	1	0	0	0	0	0	0	0	0	0	0	0	0	0	0
343	56	670	23	711	192	119	175	247	194	775	1859	254	340	83	147	108	281	495	481	308	68	178
382	181	810	1853	3803	396	259	107	238	277	337	864	144	977	441	89	488	400	647	586	229	32	744
0	0	0	0	0	0	0	0	0	0	0	0	0	0	0	0	0	0	0	0	0	0	0
22	11	14	0	3	0	0	0	0	0	0	0	0	0	0	0	0	13	0	0	2	0	1
336	111	254	178	7	8	15	14	8	16	18	112	0	71	14	8	3	98	17	11	23	9	41
0	0	0	0	0	0	0	0	0	0	0	0	0	0	0	0	0	0	0	0	0	0	0
26	0	60	0	0	1	0	0	4	0	0	0	0	5	26	0	0	7	18	0	0	1	5
9	28	39	19	146	25	46	25	45	176	126	77	55	238	251	30	41	133	58	167	168	53	33
0	0	3	0	0	0	0	0	0	0	0	0	0	0	0	0	0	0	0	0	0	0	0
0	13	129	0	127	90	47	57	48	78	79	232	55	307	61	9	22	57	140	87	79	13	58
8	11	19	0	1	0	6	0	1	20	0	0	0	165	2219	0	2	515	0	0	0	1	5
0	0	0	0	0	0	0	0	0	1	0	0	0	0	0	0	0	0	0	0	0	0	5
0	0	0	0	0	0	0	0	0	0	0	0	0	3	0	0	0	0	0	0	0	0	0
0	0	0	0	0	0	0	0	0	0	0	0	0	0	0	0	0	0	0	0	0	0	0
0	0	0	0	0	0	0	0	0	0	0	0	0	0	0	0	0	0	0	0	0	0	1
0	0	0	0	0	0	0	0	0	0	0	0	0	0	0	0	0	0	0	0	0	0	0
0	0	0	0	0	0	0	1	0	0	0	0	0	0	0	0	0	0	0	0	0	0	0
0	0	0	0	0	0	0	0	0	0	0	0	0	0	0	0	0	0	0	0	0	0	0
0	0	0	0	0	1	0	0	0	0	0	0	0	0	0	0	0	0	0	0	0	0	0
0	0	0	0	0	0	0	0	0	0	0	0	7	0	0	0	0	0	8	0	0	0	0
0	0	0	0	0	0	0	0	0	0	0	0	0	0	0	0	0	0	0	0	0	0	0
0	0	0	0	0	0	0	0	0	0	0	0	0	0	0	0	0	0	0	0	3	0	0
12	0	10	0	0	8	25	9	26	7	0	0	0	57	949	0	0	269	0	0	34	10	180
0	0	0	0	0	0	2	0	0	0	0	0	0	0	0	0	0	0	0	0	0	0	7
0	0	0	0	0	1	10	3	3	0	0	0	0	0	126	0	0	16	0	0	97	2	14
0	0	2	0	0	1	3	3	3	1	0	0	0	0	0	0	0	4	0	0	0	5	12
0	0	0	0	0	0	0	0	0	0	0	0	0	0	0	0	0	0	0	0	0	0	1
50	67	300	72	0	1	12	2	20	6	1	103	78	1	12	2	0	52	71	3	15	13	37
0	0	0	0	0	0	0	0	0	0	0	0	0	0	0	0	0	0	0	0	0	0	0
263	96	451	487	432	165	140	61	98	2559	1531	885	612	1014	706	570	930	529	187	1964	676	29	228
0	11	38	0	4	11	25	3	17	4	30	48	0	26	12	3	2	36	7	31	10	2	113
70	16	202	168	193	136	67	26	48	44	467	424	290	476	222	62	42	275	138	168	125	15	145

E1.016	E1.017	E1.018	E1.020	E1.021	E1.024	E1.025	E1.026	E1.027	E1.029	E1.030	E1.031	E1.032	E1.033	E1.034	E1.036	E1.037	E1.038	E2.002	E2.003	E2.009	E2.010	E2.012
Esophag	Esophag	Esophag	Esophag	Esophag	Esophag	Esophag	Esophag	Esophag	Esophag	Esophag	Esophag	Esophag	Esophag	Esophag	Esophag	Esophag	Esophag	Esophag	Esophag	Esophag	Esophag	Esophag
UE	UE	UE	UE	UE	UE	UE	UE	UE	UE	UE	UE	UE	UE	UE	UE	UE	UE	ME	ME	ME	ME	ME
0	0	4	0	0	1	24	5	3	1	0	0	0	0	0	0	1	7	0	1	21	10	15
64	0	30	7	0	40	81	82	708	450	0	15	156	40	2	3	203	233	38	0	20	49	172
0	0	0	0	0	0	0	0	1	0	0	0	0	0	0	0	0	1	0	0	0	0	0
2211	3127	10600	2398	2491	1358	851	1881	2270	2755	4890	9735	2649	9605	4520	861	15674	4074	2686	15332	3613	235	1121
0	0	0	0	0	0	1	1	0	3	0	0	0	0	0	0	0	0	0	0	1	0	1
0	0	0	0	0	0	0	0	0	0	0	0	0	0	0	0	0	0	0	0	0	0	0
39	0	90	50	61	45	25	21	45	22	15	69	35	101	34	15	52	13	151	96	70	3	45
14	0	19	0	56	25	12	14	36	52	0	34	0	10	0	1	19	0	12	29	3	6	90
97	5	30	11	7	1	2	1	0	2	0	0	0	0	1	0	0	11	0	7	0	0	1
24	28	18	0	0	1	15	31	5	16	9	0	0	0	1	0	1	28	0	0	19	9	62
7	0	0	0	0	0	0	0	0	0	0	0	0	0	0	0	0	0	0	0	0	0	0
0	0	7	0	0	0	1	0	0	0	0	0	0	0	16	0	0	0	0	0	2	0	1
277	103	352	317	264	107	122	96	128	95	66	189	28	49	163	9	22	290	334	90	285	56	710
14	0	9	0	0	9	4	0	5	0	0	0	0	9	16	0	3	0	0	5	0	4	0
8	5	106	0	104	71	25	28	27	26	15	16	0	85	58	8	33	21	133	71	31	13	35
556	202	328	171	28	24	38	15	23	21	0	68	0	14	6	0	3	111	21	17	128	20	110
0	0	0	0	0	0	0	0	0	0	0	0	0	0	0	0	0	0	0	0	0	0	0
185	126	320	625	1504	101	38	35	107	129	114	633	114	422	309	32	385	114	121	246	114	15	170
0	0	0	0	0	0	0	0	0	0	0	0	0	0	0	0	0	0	0	0	0	0	0
57	32	146	17	29	17	54	25	31	15	0	12	0	136	20	2	10	51	45	35	40	16	91
144	57	47	35	554	100	110	137	119	140	396	213	99	376	256	12	114	70	532	341	82	25	53
73	12	69	45	31	31	31	13	32	23	79	55	0	449	27	4	11	14	66	84	8	33	59
0	0	0	0	0	0	0	0	0	0	0	0	0	0	0	0	0	0	0	0	0	0	0
0	0	0	0	0	0	0	0	0	0	0	0	0	0	0	0	0	0	0	0	0	0	0
507	161	721	225	43	42	504	174	104	131	108	110	295	127	650	28	71	454	280	113	153	271	872
0	0	0	0	0	0	0	0	0	0	0	0	0	0	0	0	0	0	0	0	0	0	2
0	0	0	0	0	0	0	0	0	0	0	0	0	0	0	0	0	0	0	0	0	0	0
0	0	0	0	0	0	1	0	0	1	0	0	0	0	0	0	0	0	0	0	1	0	0
80	34	69	37	13	4	41	19	4	11	33	32	26	55	63	7	5	76	92	18	3	15	66
29	11	12	56	0	8	242	106	24	20	18	56	178	0	42	0	4	41	102	18	91	90	198
6	0	5	13	0	0	10	6	1	3	0	0	0	0	21	0	0	0	0	0	1	3	12
14	13	56	3	1	7	49	26	19	10	6	95	173	66	108	6	7	39	81	21	6	11	41
0	0	0	0	0	0	0	0	0	2	0	0	0	0	0	0	0	0	0	0	0	0	0
0	0	0	0	0	1	12	8	1	8	32	17	0	19	103	13	7	83	88	33	3	0	47
0	0	38	0	6	0	2	1	1	8	0	0	0	0	0	0	0	0	15	0	0	2	3
0	0	2	0	0	0	0	0	0	0	0	0	0	0	0	0	0	0	0	0	0	0	0
0	0	0	0	0	0	0	1	0	1	0	0	0	0	0	0	0	0	0	0	0	0	1

E1.016	E1.017	E1.018	E1.020	E1.021	E1.024	E1.025	E1.026	E1.027	E1.029	E1.030	E1.031	E1.032	E1.033	E1.034	E1.036	E1.037	E1.038	E2.002	E2.003	E2.009	E2.010	E2.012
Esophag	Esophag	Esophag	Esophag	Esophag	Esophag	Esophag	Esophag	Esophag	Esophag	Esophag	Esophag	Esophag	Esophag	Esophag	Esophag	Esophag	Esophag	Esophag	Esophag	Esophag	Esophag	Esophag
UE	UE	UE	UE	UE	UE	UE	UE	UE	UE	UE	UE	UE	UE	UE	UE	UE	UE	ME	ME	ME	ME	ME
19	0	85	10	0	1	2	8	9	1	0	0	0	201	0	0	0	36	0	0	2	3	15
9	2	152	0	1	2	12	6	1	0	5	9	21	0	22	0	0	13	9	2	56	0	121
0	10	381	0	3	2	17	15	16	13	12	0	0	1	25	0	0	5	32	0	5	14	104
0	0	0	0	0	0	0	0	0	0	0	0	0	0	0	0	0	0	0	0	0	0	0
0	0	0	0	0	0	0	0	0	0	0	0	0	0	0	0	0	0	0	0	0	0	0
0	0	0	0	0	0	0	0	0	0	0	0	0	0	0	0	0	0	0	0	0	0	0
0	0	31	0	0	1	2	3	9	7	8	0	0	6	0	0	4	0	0	0	1	0	4
30	117	1353	89	17	270	9753	7248	1129	124	64	128	100	111	333	10	28	171	162	45	41	5154	5471
30	2	11	1	0	3	24	25	9	1	0	0	0	0	1	0	0	0	7	0	16	11	19
0	14	18	34	28	39	17	27	25	40	9	30	0	70	0	0	16	6	23	34	19	12	17
17	12	212	17	20	56	51	45	33	55	55	3	15	101	214	15	29	146	93	6	30	19	140
7	49	699	44	3	12	18	17	20	30	44	170	208	93	856	7	4	307	99	3	304	14	34
4	0	3	0	0	0	1	1	2	0	0	0	0	0	0	2	0	0	0	0	0	0	3
1166	602	1614	94	1320	806	435	354	313	444	722	1370	263	2300	480	303	179	829	1491	1342	1180	477	1300
0	0	19	5	1	0	3	0	0	1	0	0	0	0	15	3	1	12	18	0	1	1	15
0	0	12	0	0	0	1	0	0	0	2	0	0	0	0	0	0	0	0	0	0	0	0
0	0	54	0	0	0	2	0	0	0	0	0	0	0	0	0	1	0	0	0	0	0	3
0	0	0	0	3	0	0	0	0	0	0	0	0	0	0	0	0	0	7	3	6	0	0
0	0	0	0	0	0	0	0	0	0	0	0	0	0	0	0	0	0	0	0	0	0	0
79	8	31	0	0	2	1	1	3	2	0	0	0	0	0	0	4	7	5	1	6	4	3
0	0	0	0	0	0	0	0	0	1	0	0	0	0	0	0	0	0	0	0	1	0	1
0	0	0	0	0	0	0	0	0	0	0	0	0	0	0	0	0	0	0	0	0	0	0
0	0	0	0	0	0	0	0	0	0	0	0	0	0	0	0	0	0	0	0	0	0	0
0	0	0	0	0	0	0	0	0	0	0	0	0	0	0	0	0	0	0	0	0	0	0
0	14	72	189	294	55	43	44	98	51	75	1140	216	95	196	0	51	37	56	155	52	6	58
13	179	42	0	347	0	30	67	0	3	20	0	0	0	3	0	0	0	24	0	5	1	92
0	0	7	0	0	0	0	0	0	0	0	0	0	0	12	0	0	0	11	0	1	0	0
0	2	0	1	0	3	6	0	0	2	0	0	0	0	0	0	0	0	0	0	1	3	3
0	0	0	0	0	0	0	0	0	0	0	0	0	0	0	0	0	0	0	0	0	0	2
0	0	0	0	0	0	3	0	0	1	0	0	0	0	0	0	0	0	0	0	0	0	1
0	0	0	0	0	0	3	1	0	0	0	0	0	0	0	0	0	0	0	0	0	0	1
0	0	1	0	3	2	1	3	14	6	0	0	0	21	0	0	0	8	0	1	8	0	4
1229	93	1109	114	26	67	710	557	190	217	71	29	595	108	210	16	198	248	2254	49	259	425	797
0	0	0	0	0	0	0	0	0	0	0	0	0	0	0	0	0	0	0	0	0	0	1
2	0	6	0	3	0	0	0	0	0	0	0	0	0	0	3	0	0	0	0	0	0	0
0	0	0	0	0	0	0	0	0	0	0	0	0	0	8	0	0	0	0	0	0	0	0
0	0	0	0	0	1	0	0	0	0	0	0	0	0	0	0	0	0	0	0	0	0	2

E1.016	E1.017	E1.018	E1.020	E1.021	E1.024	E1.025	E1.026	E1.027	E1.029	E1.030	E1.031	E1.032	E1.033	E1.034	E1.036	E1.037	E1.038	E2.002	E2.003	E2.009	E2.010	E2.012
Esophag	Esophag	Esophag	Esophag	Esophag	Esophag	Esophag	Esophag	Esophag	Esophag	Esophag	Esophag	Esophag	Esophag	Esophag	Esophag	Esophag	Esophag	Esophag	Esophag	Esophag	Esophag	Esophag
UE	UE	UE	UE	UE	UE	UE	UE	UE	UE	UE	UE	UE	UE	UE	UE	UE	UE	ME	ME	ME	ME	ME
1382	12872	1397	304	9221	1984	532	562	876	2224	7222	1012	1184	3840	592	14123	177	2704	824	5707	3004	411	329
134	15	126	62	2	13	76	10	44	42	25	44	129	14	51	39	5	358	202	140	24	42	104
34	21	29	28	1	35	378	228	123	287	0	52	0	5	1113	3	2	273	27	0	124	163	267
0	0	0	0	0	0	0	0	0	0	0	0	0	0	0	0	0	0	0	0	0	0	0
0	0	0	0	0	0	0	0	0	0	0	0	0	0	0	0	0	0	0	0	0	0	0
0	0	0	0	0	0	0	0	0	0	0	0	0	0	0	0	0	0	0	0	0	0	0
11	0	0	0	1	2	9	8	6	6	0	0	0	0	0	0	2	0	0	0	3	0	22
45	21	36	7	3	13	202	146	21	17	21	44	63	197	6364	10	9	1714	42	7	103	80	161
0	0	0	0	0	0	0	0	0	0	0	0	0	0	0	0	0	0	0	0	0	0	0
0	6	95	10	10	37	17	19	32	15	2	153	0	36	68	3	25	7	27	34	52	3	4
0	0	0	0	2	2	0	0	2	0	1	0	0	4	0	0	2	0	1	1	7	1	1
0	0	0	0	0	0	0	0	0	0	0	0	0	0	0	0	0	0	0	0	0	0	0
0	9	0	0	0	0	3	0	0	0	5	0	0	0	0	0	4	8	0	5	68	0	0
0	0	4	0	30	7	4	22	36	11	0	0	94	0	0	0	5	0	24	33	7	17	91
0	0	0	0	0	0	0	0	0	0	0	0	0	0	0	0	0	0	0	0	0	0	0
0	0	0	3	24	0	2	3	6	1	0	0	0	4	0	2	1	16	0	5	4	0	4
23	0	6	0	0	6	27	0	141	1	5	48	0	1	0	0	7	0	0	0	2	1	343
0	0	0	0	0	0	0	0	0	0	0	0	0	0	0	0	0	0	0	0	0	0	0
26	33	137	17	7	0	0	0	0	0	0	0	0	0	0	0	0	22	0	0	1	0	0
0	0	38	0	0	0	3	4	8	1	0	0	0	1	11	0	0	0	0	0	0	0	3
0	0	24	0	0	0	0	8	0	0	0	0	0	0	0	0	0	0	0	0	0	0	3
0	0	0	0	0	0	0	0	0	0	0	0	0	0	0	0	0	0	0	0	0	0	1
0	0	3	0	0	0	0	0	0	0	0	0	0	0	0	0	0	0	0	0	0	0	0
0	0	0	0	0	0	0	0	0	0	0	6	0	0	0	0	0	0	0	0	0	0	0
0	0	0	0	0	0	0	0	0	0	0	0	0	0	0	0	0	0	0	0	0	0	2
0	0	0	0	0	0	0	0	0	0	0	0	0	0	0	0	0	0	0	0	0	0	0
0	0	3	0	0	0	0	0	0	0	0	0	0	0	0	0	0	0	0	0	0	0	0
0	0	0	0	0	0	0	0	0	0	0	0	0	0	0	0	0	0	0	0	0	0	3
190	20	223	33	408	28	25	13	30	26	1	9	36	87	16	6	131	58	156	105	117	4	127
0	0	0	0	0	0	0	0	0	0	0	0	0	0	0	0	0	0	0	0	0	0	1
0	0	0	0	0	0	0	0	0	0	0	0	0	0	0	0	0	0	0	0	0	0	0
0	0	0	0	0	0	0	0	1	0	0	0	0	0	0	0	0	0	0	0	0	1	0
0	0	0	0	1	0	0	0	3	0	0	0	0	0	0	0	5	0	0	0	2	0	0
0	0	0	0	0	0	0	0	0	0	0	0	0	9	0	0	0	0	0	0	0	0	0
5	0	3	0	0	0	0	0	0	0	0	0	0	0	0	0	0	0	0	0	0	0	0
67	1	48	16	5	7	4	2	9	14	7	0	0	23	19	0	1	16	44	5	8	0	31
0	0	0	0	0	0	0	0	0	0	0	0	0	0	0	0	0	0	0	0	0	0	0

E1.016	E1.017	E1.018	E1.020	E1.021	E1.024	E1.025	E1.026	E1.027	E1.029	E1.030	E1.031	E1.032	E1.033	E1.034	E1.036	E1.037	E1.038	E2.002	E2.003	E2.009	E2.010	E2.012
Esophag	Esophag	Esophag	Esophag	Esophag	Esophag	Esophag	Esophag	Esophag	Esophag	Esophag	Esophag	Esophag	Esophag	Esophag	Esophag	Esophag	Esophag	Esophag	Esophag	Esophag	Esophag	Esophag
UE	UE	UE	UE	UE	UE	UE	UE	UE	UE	UE	UE	UE	UE	UE	UE	UE	UE	ME	ME	ME	ME	ME
0	0	0	0	0	0	0	0	0	0	0	0	0	0	0	0	0	3	0	0	0	0	0
0	0	0	0	0	0	0	0	0	0	0	0	0	1	0	0	0	0	0	0	0	0	0
0	0	0	0	0	0	0	0	0	0	0	0	0	0	0	0	0	0	0	0	0	0	0
0	0	0	0	0	0	0	9	0	0	0	0	0	0	0	0	0	0	0	0	0	0	0
0	0	0	0	0	0	0	0	0	0	0	0	0	0	0	0	0	0	0	0	0	0	0
0	0	0	0	0	0	0	0	0	0	0	0	0	0	0	0	0	0	0	0	0	0	0
0	0	20	0	0	0	0	0	0	0	0	0	12	0	0	0	0	0	0	0	0	0	0
0	0	0	0	0	0	45	1	0	0	0	0	0	0	0	0	0	0	0	0	0	0	3
0	0	13	0	0	0	2	0	0	0	0	0	0	0	0	0	0	0	0	0	0	0	0
0	0	0	0	0	0	0	0	0	0	0	0	0	0	0	0	0	0	0	0	0	0	0
0	0	0	0	0	0	0	0	0	0	0	0	0	0	0	0	0	8	0	0	0	0	0
1	0	0	0	0	0	0	0	0	0	0	0	0	1	0	0	0	0	0	0	0	0	1
17	7	17	0	0	0	0	0	1	1	3	5	1	0	7	0	0	0	33	0	0	0	2
4	0	0	0	0	0	1	0	0	0	0	0	0	46	0	0	0	0	0	0	0	0	1
0	0	0	0	0	0	0	0	0	0	0	0	0	0	0	0	0	0	0	0	0	0	0
0	0	0	0	0	0	0	0	0	0	0	0	0	0	0	0	0	0	0	0	0	0	0
5	0	0	0	0	0	0	0	0	0	0	0	0	1	0	0	1	0	0	0	0	0	1
0	0	1	0	0	0	0	0	0	1	0	0	0	0	0	0	0	0	0	0	0	0	0
0	0	1	0	0	0	0	0	0	0	0	0	0	0	0	0	0	0	0	0	0	0	0
0	0	0	0	0	0	0	0	0	0	0	0	0	0	0	0	0	0	0	0	0	0	0
7	0	8	0	3	2	1	0	0	1	0	6	0	15	3	0	0	1	0	1	0	0	5
0	0	0	0	0	0	0	0	0	0	0	0	0	5	0	0	0	0	0	0	0	0	0
0	0	2	0	0	0	0	0	0	0	0	0	0	0	0	0	0	0	0	0	0	0	0
0	0	0	0	0	0	0	0	0	0	0	0	0	0	0	0	0	0	0	0	0	0	0
0	0	16	0	0	0	2	1	3	5	0	0	0	9	0	0	0	0	0	0	1	1	5
0	0	0	0	0	0	1	0	0	2	0	0	0	0	0	0	0	0	0	0	0	0	0
0	0	0	0	0	0	0	0	0	0	0	0	0	1	0	0	0	2	3	0	1	0	0
0	0	0	0	0	0	0	0	0	0	0	0	0	0	0	0	0	0	0	0	0	0	0
5	0	1	0	0	0	0	0	1	0	0	0	0	56	0	0	0	0	0	0	0	0	1
0	0	0	0	0	0	0	0	0	0	0	0	0	0	0	0	0	0	0	0	0	0	0
0	0	0	0	1	0	0	0	0	0	0	0	0	19	62	4	16	85	30	6	0	1	5
68	88	73	64	109	26	20	4	4	17	752	610	102	222	57	4	160	106	97	56	87	3	56
0	0	0	0	0	0	0	0	0	0	0	0	0	0	0	0	0	0	0	0	0	0	0
10	0	3	4	0	0	1	0	0	0	0	30	0	3	0	0	0	0	7	0	0	1	2
1	0	12	0	0	0	0	0	0	0	0	0	0	0	0	0	0	0	0	0	0	0	3
0	0	0	0	0	0	0	0	0	1	0	0	0	0	0	0	0	0	0	0	0	0	0
0	0	0	0	0	0	1	0	0	0	0	0	0	0	0	0	0	0	0	0	0	0	1

E1.016	E1.017	E1.018	E1.020	E1.021	E1.024	E1.025	E1.026	E1.027	E1.029	E1.030	E1.031	E1.032	E1.033	E1.034	E1.036	E1.037	E1.038	E2.002	E2.003	E2.009	E2.010	E2.012
Esophag	Esophag	Esophag	Esophag	Esophag	Esophag	Esophag	Esophag	Esophag	Esophag	Esophag	Esophag	Esophag	Esophag	Esophag	Esophag	Esophag	Esophag	Esophag	Esophag	Esophag	Esophag	Esophag
UE	UE	UE	UE	UE	UE	UE	UE	UE	UE	UE	UE	UE	UE	UE	UE	UE	UE	ME	ME	ME	ME	ME
9	0	0	0	0	1	13	7	2	0	0	0	0	11	10	0	0	1	0	0	15	10	12
0	0	0	0	0	0	0	0	0	0	0	0	0	0	0	0	0	0	0	0	0	0	0
0	0	0	0	0	0	0	0	0	0	0	0	0	0	0	0	0	0	0	0	0	0	0
0	0	0	0	0	0	0	0	0	0	0	0	0	0	0	0	0	0	0	0	0	0	0
0	0	22	0	0	0	0	0	0	0	0	0	0	0	0	0	0	0	0	0	0	0	0
0	0	0	0	0	0	0	0	0	0	0	0	0	0	0	0	0	0	0	0	0	0	1
0	0	3	0	0	0	0	0	0	0	0	0	0	121	13	0	0	0	0	0	0	0	0
0	0	0	0	0	0	0	0	0	0	0	0	0	0	0	0	0	0	0	0	0	0	0
0	0	156	0	0	0	0	0	0	0	0	0	0	30	0	0	0	0	0	0	0	0	2
0	4	0	0	0	0	0	0	0	0	0	0	0	0	0	0	0	0	0	0	0	0	0
0	3	0	0	0	0	0	0	0	0	0	0	0	0	0	0	0	0	0	0	0	0	0
0	0	0	0	0	0	0	0	0	0	0	0	0	0	0	0	0	0	0	0	0	0	0
0	0	0	0	0	0	0	0	0	0	0	0	0	0	0	0	0	0	0	0	0	0	0
0	0	31	0	0	0	0	0	0	0	0	0	0	0	0	0	0	0	0	0	0	0	0
89	1	70	18	0	2	5	3	8	15	28	6	83	17	10	0	5	17	92	19	4	3	43
0	0	0	0	0	0	0	0	0	0	0	0	0	0	0	0	0	0	0	0	0	0	0
2	0	24	0	9	0	0	1	3	2	0	14	0	32	19	3	0	5	5	1	11	0	1
0	0	0	0	0	0	0	0	0	0	0	0	0	0	0	0	0	0	0	0	0	2	1
0	0	0	0	0	0	0	0	0	0	0	0	0	0	0	0	0	0	0	0	0	0	0
0	0	0	0	0	0	0	0	0	0	0	0	0	0	0	0	0	0	0	0	0	0	0
0	0	0	0	0	0	0	0	0	0	0	0	0	0	0	0	0	0	0	0	0	0	0
0	0	7	16	0	0	0	0	0	0	0	0	0	0	0	0	0	0	0	0	0	0	0
0	0	0	0	0	0	0	0	0	0	0	0	0	0	0	0	0	0	0	0	0	0	0
14	0	14	2	0	0	0	0	0	1	0	0	0	0	2	1	0	2	54	2	0	0	1
0	0	0	0	0	0	0	0	0	0	0	0	0	0	0	0	0	0	0	0	0	0	0
0	0	0	0	0	0	0	0	0	0	0	0	0	0	0	0	0	0	2	0	0	0	0
0	0	0	0	0	0	0	0	0	0	0	0	0	0	0	0	0	0	0	0	0	0	0
1	0	0	0	0	2	3	7	4	3	12	0	0	0	0	0	1	4	0	3	48	6	33
0	0	0	0	0	0	0	0	0	0	0	0	0	0	0	0	0	0	0	0	0	0	0
0	1	6	0	2	0	0	0	0	3	0	0	0	0	0	0	0	0	0	0	0	0	1
0	0	0	0	0	0	2	5	0	1	0	0	0	0	0	0	0	0	0	0	0	0	6
0	0	2	0	0	89	56	618	99	38	0	0	0	0	4	0	0	41	14	0	4	92	563
0	0	0	0	0	0	0	0	0	0	0	0	0	0	0	0	0	0	0	0	0	0	0
0	0	2	0	76	0	4	6	9	3	0	0	8	14	0	1	8	0	22	5	6	0	9
0	0	0	0	0	0	0	0	0	0	0	0	0	0	0	0	0	0	0	0	0	0	0
0	0	0	0	0	0	0	0	0	0	0	0	0	0	0	0	0	0	0	0	2	0	0
0	0	0	0	1	0	0	0	0	0	0	0	0	0	0	0	0	0	0	0	0	0	0

E1.016	E1.017	E1.018	E1.020	E1.021	E1.024	E1.025	E1.026	E1.027	E1.029	E1.030	E1.031	E1.032	E1.033	E1.034	E1.036	E1.037	E1.038	E2.002	E2.003	E2.009	E2.010	E2.012
Esophag	Esophag	Esophag	Esophag	Esophag	Esophag	Esophag	Esophag	Esophag	Esophag	Esophag	Esophag	Esophag	Esophag	Esophag	Esophag	Esophag	Esophag	Esophag	Esophag	Esophag	Esophag	Esophag
UE	UE	UE	UE	UE	UE	UE	UE	UE	UE	UE	UE	UE	UE	UE	UE	UE	UE	ME	ME	ME	ME	ME
0	0	3	0	2	0	0	0	0	0	0	0	0	0	0	0	0	0	1	0	0	0	0
7	6	4	0	0	3	8	86	13	10	0	0	0	0	0	0	0	1	0	0	2	3	104
0	0	0	0	0	0	0	0	0	0	0	0	0	0	0	0	0	0	0	0	0	0	0
0	0	4	0	0	0	0	0	0	0	0	0	0	0	0	0	0	0	0	0	0	0	0
0	0	0	0	0	0	0	0	0	0	0	0	0	0	0	0	0	0	0	0	0	0	0
0	0	0	0	0	0	0	0	0	0	0	0	0	0	0	0	0	0	0	0	0	0	0
0	7	2	0	0	3	1	1	4	7	0	2	0	0	0	0	0	4	10	0	1	0	20
0	0	0	0	0	0	0	0	0	0	0	0	0	0	18	0	1	0	0	0	0	0	0
0	0	0	0	0	0	0	0	0	0	0	0	0	0	0	0	0	0	0	0	0	0	0
0	0	11	0	0	0	0	0	6	0	0	0	0	0	0	0	0	0	0	0	0	1	0
0	0	0	0	0	0	0	0	0	0	0	0	0	0	0	0	0	0	0	0	0	0	0
29	27	45	25	14	23	54	15	11	34	6	60	0	45	25	0	4	39	65	8	40	16	115
19	0	23	0	0	0	1	0	1	4	0	0	0	0	140	0	2	52	0	0	1	0	6
0	0	0	0	0	0	0	0	0	0	0	0	0	0	0	0	0	0	0	0	0	0	0
0	0	0	0	0	0	0	0	0	0	0	0	0	0	0	0	0	0	0	0	0	0	0
0	0	0	0	0	0	0	0	0	0	0	0	0	0	0	0	0	0	0	0	0	0	0
0	0	4	0	0	0	4	0	0	0	0	0	0	0	0	0	0	0	0	0	0	0	0
0	0	0	0	0	0	0	0	0	1	0	0	0	0	0	0	0	0	0	0	4	0	0
1288	205	748	664	1660	681	601	89	640	147	229	2221	1222	488	205	618	182	650	382	3068	175	139	777
0	0	0	0	0	1	0	0	0	0	0	0	0	0	0	0	1	0	0	0	0	0	0
0	0	0	0	0	0	0	0	0	0	0	0	0	0	0	0	0	0	0	0	1	0	0
0	0	0	0	0	0	0	0	0	0	0	0	0	0	0	0	0	0	0	0	0	0	0
184	51	81	102	10	6	21	5	16	28	0	4	0	14	0	9	6	53	9	9	13	9	40
0	0	0	0	0	0	0	0	39	0	0	0	0	0	8	0	0	0	0	8	0	1	1
0	0	0	1	0	0	0	0	1	0	0	0	0	0	0	0	0	0	0	0	0	0	0
0	0	0	0	0	0	1	0	0	0	0	0	0	0	0	0	0	0	0	0	0	0	0
0	0	0	0	2	5	4	10	40	20	0	0	0	1	0	6	3	0	0	5	5	0	2
0	0	8	0	0	0	4	0	0	1	0	0	0	4	0	0	0	5	0	7	10	0	1
337	56	650	23	698	173	110	158	179	167	775	1859	254	305	77	139	104	247	453	458	267	65	174
6	0	12	0	11	14	0	5	28	6	0	0	0	30	6	2	1	29	42	11	26	3	1
0	0	0	0	0	0	0	2	0	0	0	0	0	0	0	0	0	0	0	0	0	0	0
382	181	810	1853	3803	396	259	107	238	277	337	864	144	977	441	89	488	400	647	586	229	32	744
0	0	0	0	0	0	0	0	0	0	0	0	0	0	0	0	0	0	0	0	0	0	0
0	0	0	0	0	0	0	0	0	0	0	0	0	0	0	0	0	0	0	0	0	0	0
0	0	0	0	0	0	0	0	0	0	0	0	0	0	0	0	0	0	0	0	0	0	0
19	0	1	0	0	0	0	0	0	0	0	0	0	0	0	0	0	0	0	0	0	0	0
3	11	13	0	3	0	0	0	0	0	0	0	0	0	0	0	0	13	0	0	2	0	1

E1.016	E1.017	E1.018	E1.020	E1.021	E1.024	E1.025	E1.026	E1.027	E1.029	E1.030	E1.031	E1.032	E1.033	E1.034	E1.036	E1.037	E1.038	E2.002	E2.003	E2.009	E2.010	E2.012
Esophag	Esophag	Esophag	Esophag	Esophag	Esophag	Esophag	Esophag	Esophag	Esophag	Esophag	Esophag	Esophag	Esophag	Esophag	Esophag	Esophag	Esophag	Esophag	Esophag	Esophag	Esophag	Esophag
UE	UE	UE	UE	UE	UE	UE	UE	UE	UE	UE	UE	UE	UE	UE	UE	UE	UE	ME	ME	ME	ME	ME
336	111	254	178	7	8	15	14	8	16	18	112	0	71	14	8	3	98	17	11	23	9	41
0	0	0	0	0	0	0	0	0	0	0	0	0	0	0	0	0	0	0	0	0	0	0
0	0	5	0	0	0	0	0	0	0	0	0	0	0	0	0	0	0	0	0	0	0	0
0	0	0	0	0	0	0	0	0	0	0	0	0	0	0	0	0	0	0	0	0	0	0
0	0	24	0	0	0	0	0	0	0	0	0	0	5	26	0	0	7	0	0	0	0	0
0	0	0	0	0	0	0	0	0	0	0	0	0	0	0	0	0	0	0	0	0	0	0
0	0	12	0	0	0	0	0	3	0	0	0	0	0	0	0	0	0	18	0	0	1	5
26	0	0	0	0	0	0	0	0	0	0	0	0	0	0	0	0	0	0	0	0	0	0
0	0	19	0	0	0	0	0	0	0	0	0	0	0	0	0	0	0	0	0	0	0	0
0	0	0	0	0	0	0	0	0	0	0	0	0	0	0	0	0	0	0	0	0	0	0
0	0	0	0	0	0	0	0	0	0	0	0	0	0	0	0	0	0	0	0	0	0	0
0	0	0	0	0	1	0	0	1	0	0	0	0	0	0	0	0	0	0	0	0	0	0
0	12	14	4	0	2	11	6	4	12	0	0	0	43	251	0	0	47	0	0	132	13	11
3	0	0	0	0	0	0	1	2	0	0	0	0	0	0	0	0	0	0	0	0	1	0
0	0	0	0	0	0	28	14	3	3	0	0	0	0	0	0	1	0	0	0	8	31	12
0	0	0	0	0	0	0	0	0	0	0	0	0	0	0	0	0	0	0	0	0	1	0
0	0	0	0	0	0	0	0	0	0	0	0	0	0	0	0	0	0	0	0	0	0	0
6	16	25	15	146	23	7	4	36	161	126	77	55	195	0	30	40	86	58	167	28	7	10
0	0	3	0	0	0	0	0	0	0	0	0	0	0	0	0	0	0	0	0	0	0	0
0	13	61	0	127	90	45	55	46	72	79	214	55	307	61	9	22	57	140	78	78	11	48
0	0	68	0	0	0	2	2	2	6	0	18	0	0	0	0	0	0	0	9	1	2	10
0	0	0	0	0	0	0	0	0	0	0	0	0	0	0	0	0	0	0	0	0	0	0
0	0	0	0	0	0	0	0	0	0	0	0	0	0	0	0	0	0	0	0	0	0	0
8	11	19	0	1	0	1	0	1	17	0	0	0	165	2198	0	2	512	0	0	0	0	2
0	0	0	0	0	0	5	0	0	2	0	0	0	0	21	0	0	3	0	0	0	1	2
0	0	0	0	0	0	0	0	0	1	0	0	0	0	0	0	0	0	0	0	0	0	1
0	0	0	0	0	0	0	0	0	1	0	0	0	0	0	0	0	0	0	0	0	0	5
0	0	0	0	0	0	0	0	0	0	0	0	0	3	0	0	0	0	0	0	0	0	0
0	0	0	0	0	0	0	0	0	0	0	0	0	0	0	0	0	0	0	0	0	0	0
0	0	0	0	0	0	0	0	0	0	0	0	0	0	0	0	0	0	0	0	0	0	1
0	0	0	0	0	0	0	0	0	0	0	0	0	0	0	0	0	0	0	0	0	0	0
0	0	0	0	0	0	0	0	0	0	0	0	0	0	0	0	0	0	0	0	0	0	0
0	0	0	0	0	0	0	1	0	0	0	0	0	0	0	0	0	0	0	0	0	0	0
0	0	0	0	0	0	0	0	0	0	0	0	0	0	0	0	0	0	0	0	0	0	0
0	0	0	0	0	1	0	0	0	0	0	0	0	0	0	0	0	0	0	0	0	0	0
0	0	0	0	0	0	0	0	0	0	0	0	7	0	0	0	0	0	8	0	0	0	0
0	0	0	0	0	0	0	0	0	0	0	0	0	0	0	0	0	0	0	0	0	0	0

E1.016	E1.017	E1.018	E1.020	E1.021	E1.024	E1.025	E1.026	E1.027	E1.029	E1.030	E1.031	E1.032	E1.033	E1.034	E1.036	E1.037	E1.038	E2.002	E2.003	E2.009	E2.010	E2.012
Esophag	Esophag	Esophag	Esophag	Esophag	Esophag	Esophag	Esophag	Esophag	Esophag	Esophag	Esophag	Esophag	Esophag	Esophag	Esophag	Esophag	Esophag	Esophag	Esophag	Esophag	Esophag	Esophag
UE	UE	UE	UE	UE	UE	UE	UE	UE	UE	UE	UE	UE	UE	UE	UE	UE	UE	ME	ME	ME	ME	ME
0	0	0	0	0	0	0	0	0	0	0	0	0	0	0	0	0	0	0	0	3	0	0
0	0	0	0	0	0	0	0	0	0	0	0	0	0	0	0	0	0	0	0	0	0	0
12	0	6	0	0	8	24	9	26	7	0	0	0	57	949	0	0	269	0	0	34	10	178
0	0	0	0	0	0	0	0	0	0	0	0	0	0	0	0	0	0	0	0	0	0	0
0	0	0	0	0	0	0	0	0	0	0	0	0	0	0	0	0	0	0	0	0	0	0
0	0	0	0	0	0	1	0	0	0	0	0	0	0	0	0	0	0	0	0	0	0	1
0	0	4	0	0	0	0	0	0	0	0	0	0	0	0	0	0	0	0	0	0	0	1
0	0	0	0	0	0	2	0	0	0	0	0	0	0	0	0	0	0	0	0	0	0	7
0	0	0	0	0	0	0	0	0	0	0	0	0	0	0	0	0	0	0	0	0	0	0
0	0	0	0	0	1	10	3	3	0	0	0	0	0	126	0	0	16	0	0	95	0	11
0	0	0	0	0	0	0	0	0	0	0	0	0	0	0	0	0	0	0	0	2	2	3
0	0	0	0	0	0	2	0	0	0	0	0	0	0	0	0	0	0	0	0	0	2	0
0	0	2	0	0	0	1	0	0	0	0	0	0	0	0	0	0	0	0	0	0	0	0
0	0	0	0	0	0	0	0	0	0	0	0	0	0	0	0	0	0	0	0	0	0	0
0	0	0	0	0	1	0	3	3	1	0	0	0	0	0	0	0	4	0	0	0	3	12
0	0	0	0	0	0	0	0	0	0	0	0	0	0	0	0	0	0	0	0	0	0	1
0	0	0	0	0	0	0	0	0	0	0	0	0	0	0	0	0	0	0	0	1	1	0
0	0	0	0	0	0	0	0	0	0	0	0	0	0	0	0	0	0	0	0	0	0	0
0	0	0	0	0	0	0	0	0	0	0	0	0	0	0	0	0	0	0	0	0	0	0
50	67	300	72	0	1	12	2	20	6	1	103	78	1	12	2	0	52	71	3	14	12	37
0	0	0	0	0	0	0	0	0	0	0	0	0	0	0	0	0	0	0	0	0	0	0
0	0	5	0	0	1	1	1	0	1	0	0	0	0	0	0	0	1	4	0	4	0	3
0	0	0	0	0	1	11	4	28	4	0	0	0	0	0	0	0	0	0	0	0	3	111
0	0	0	0	0	0	0	0	0	3	0	0	0	0	0	0	0	0	0	0	0	0	0
263	96	451	487	432	164	129	57	70	2552	1531	885	612	1014	706	570	930	529	187	1964	676	26	117
0	0	0	0	0	0	0	0	1	0	0	0	0	0	0	0	0	0	0	0	0	0	1
0	0	8	0	4	4	3	0	2	0	6	48	0	26	12	3	2	3	0	31	9	0	64
0	11	0	0	0	7	21	3	12	2	0	0	0	0	0	0	0	33	0	0	1	2	46
0	0	0	0	0	0	0	0	0	0	0	0	0	0	0	0	0	0	0	0	0	0	0
0	0	0	0	0	0	0	0	0	0	0	0	0	0	0	0	0	0	0	0	0	0	0
0	0	0	0	0	0	0	0	0	0	24	0	0	0	0	0	0	0	0	0	0	0	2
0	0	8	0	0	0	0	0	0	0	0	0	0	0	0	0	0	0	0	0	0	0	0
0	0	0	0	0	0	0	0	0	0	0	0	0	0	0	0	0	0	0	0	0	0	0
0	0	22	0	0	0	1	0	3	2	0	0	0	0	0	0	0	0	7	0	0	0	1
0	0	6	0	0	0	0	0	0	0	0	0	0	0	0	0	0	0	0	0	0	0	4
0	0	0	0	0	0	0	0	0	0	0	0	0	0	0	0	0	0	0	0	0	0	0
70	16	196	168	193	136	67	26	48	44	467	424	290	476	222	62	42	275	138	168	125	15	140

E1.016	E1.017	E1.018	E1.020	E1.021	E1.024	E1.025	E1.026	E1.027	E1.029	E1.030	E1.031	E1.032	E1.033	E1.034	E1.036	E1.037	E1.038	E2.002	E2.003	E2.009	E2.010	E2.012
Esophag	Esophag	Esophag	Esophag	Esophag	Esophag	Esophag	Esophag	Esophag	Esophag	Esophag	Esophag	Esophag	Esophag	Esophag	Esophag	Esophag	Esophag	Esophag	Esophag	Esophag	Esophag	Esophag
UE	UE	UE	UE	UE	UE	UE	UE	UE	UE	UE	UE	UE	UE	UE	UE	UE	UE	ME	ME	ME	ME	ME
0	0	0	0	0	0	0	0	0	0	0	0	0	0	0	0	0	0	0	0	0	0	0
0	0	0	0	0	0	0	0	0	0	0	0	0	0	0	0	0	0	0	0	0	0	1
0	0	0	0	0	0	0	0	0	0	0	0	0	0	0	0	0	0	0	0	0	0	0
0	0	4	0	0	0	3	0	1	0	0	0	0	0	0	0	1	7	0	0	12	1	2
0	0	0	0	0	0	0	0	0	0	0	0	0	0	0	0	0	0	0	0	0	0	0
0	0	0	0	0	1	9	2	2	1	0	0	0	0	0	0	0	0	0	1	1	2	5
0	0	0	0	0	0	12	3	0	0	0	0	0	0	0	0	0	0	0	0	8	7	8
64	0	30	7	0	40	81	82	708	450	0	15	156	40	2	3	203	233	38	0	20	49	172
0	0	0	0	0	0	0	0	1	0	0	0	0	0	0	0	0	1	0	0	0	0	0
0	0	0	0	0	0	0	0	0	0	0	0	0	0	0	2	0	0	0	0	0	0	1
2211	3127	10600	2398	2491	1358	851	1881	2270	2755	4890	9735	2649	9605	4520	859	15674	4074	2686	15332	3613	235	1118
0	0	0	0	0	0	0	0	0	0	0	0	0	0	0	0	0	0	0	0	0	0	2
0	0	0	0	2	1	3	0	3	1	0	1	0	0	0	0	0	0	0	0	0	3	18
0	0	0	0	0	0	1	1	0	3	0	0	0	0	0	0	0	0	0	0	1	0	1
0	0	0	0	0	0	1	0	0	0	0	0	0	0	0	0	0	0	0	0	0	0	0
0	0	0	0	0	0	0	0	0	0	0	0	0	0	0	0	0	0	0	0	0	0	0
0	0	7	0	0	9	3	1	18	1	14	0	0	0	0	0	0	0	2	0	1	0	0
5	0	17	11	41	7	3	0	8	14	0	54	35	10	10	5	15	0	30	48	6	0	28
0	0	0	23	0	2	0	1	0	0	0	0	0	0	11	0	1	0	8	8	2	0	0
34	0	66	16	20	27	19	19	19	7	1	15	0	91	13	10	36	13	111	40	61	3	17
0	0	0	0	0	0	0	0	0	0	0	0	0	0	0	0	0	0	0	0	0	0	2
0	0	0	0	0	8	2	3	2	15	0	0	0	0	0	0	0	0	0	0	0	4	45
0	0	0	0	0	3	1	2	9	3	0	0	0	0	0	0	0	0	0	0	0	1	12
0	0	0	0	0	0	0	0	0	0	0	0	0	0	0	0	0	0	0	0	0	0	0
14	0	19	0	48	13	9	7	6	23	0	34	0	10	0	1	19	0	12	29	3	0	14
0	0	0	0	8	1	0	2	19	11	0	0	0	0	0	0	0	0	0	0	0	1	17
0	0	0	0	0	0	0	0	0	0	0	0	0	0	0	0	0	0	0	0	0	0	0
0	0	0	0	0	0	0	0	0	0	0	0	0	0	0	0	0	0	0	0	0	0	0
0	0	0	0	0	0	0	0	0	0	0	0	0	0	0	0	0	0	0	0	0	0	0
0	0	0	0	0	0	0	0	0	0	0	0	0	0	0	0	0	0	0	0	0	0	0
0	0	0	0	0	0	0	0	0	0	0	0	0	0	0	0	0	0	0	0	0	0	0
97	5	30	11	7	1	2	1	0	2	0	0	0	0	1	0	0	11	0	7	0	0	1
0	0	0	0	0	0	0	0	0	0	0	0	0	0	0	0	0	0	0	0	7	0	0
0	0	0	0	0	1	6	28	3	7	6	0	0	0	0	0	1	0	0	0	7	4	37
0	0	0	0	0	0	0	0	0	0	0	0	0	0	0	0	0	0	0	0	0	0	4
0	0	0	0	0	0	0	0	0	0	0	0	0	0	0	0	0	0	0	0	1	0	0
0	0	0	0	0	0	0	0	0	3	0	0	0	0	0	0	0	0	0	0	0	0	1

E1.016	E1.017	E1.018	E1.020	E1.021	E1.024	E1.025	E1.026	E1.027	E1.029	E1.030	E1.031	E1.032	E1.033	E1.034	E1.036	E1.037	E1.038	E2.002	E2.003	E2.009	E2.010	E2.012
Esophag	Esophag	Esophag	Esophag	Esophag	Esophag	Esophag	Esophag	Esophag	Esophag	Esophag	Esophag	Esophag	Esophag	Esophag	Esophag	Esophag	Esophag	Esophag	Esophag	Esophag	Esophag	Esophag
UE	UE	UE	UE	UE	UE	UE	UE	UE	UE	UE	UE	UE	UE	UE	UE	UE	UE	ME	ME	ME	ME	ME
24	28	18	0	0	0	9	3	2	6	3	0	0	0	1	0	0	28	0	0	4	5	20
7	0	0	0	0	0	0	0	0	0	0	0	0	0	0	0	0	0	0	0	0	0	0
0	0	0	0	0	0	0	0	0	0	0	0	0	0	0	0	0	0	0	0	0	0	0
0	0	0	0	0	0	0	0	0	0	0	0	0	0	0	0	0	0	0	0	0	0	0
0	0	7	0	0	0	1	0	0	0	0	0	0	0	16	0	0	0	0	0	2	0	1
0	0	7	3	0	0	0	0	2	0	0	0	0	0	0	0	0	0	0	0	6	1	14
0	0	0	0	0	0	0	0	0	0	0	0	0	0	0	0	0	0	0	0	3	2	1
14	22	56	28	0	7	17	8	12	11	0	58	0	0	0	0	0	36	0	0	33	14	45
0	0	0	1	19	4	4	0	1	1	0	0	0	0	9	4	3	12	0	2	4	0	66
12	0	4	0	6	16	3	9	10	11	0	0	0	21	21	0	3	0	15	3	26	0	20
104	0	38	35	0	2	3	1	5	0	0	0	0	0	0	1	1	10	0	0	15	3	10
0	0	6	7	0	4	4	6	4	6	0	0	0	0	42	0	0	1	0	1	4	4	19
0	0	0	0	0	0	0	0	0	0	0	0	0	0	0	0	0	0	0	0	0	0	0
0	0	0	0	1	0	0	0	0	0	0	0	0	0	0	0	0	0	0	0	2	0	0
5	0	3	0	0	1	5	2	3	1	0	0	0	0	0	0	0	13	0	0	4	2	6
0	0	0	0	0	0	1	0	0	0	0	15	0	0	0	0	1	0	1	0	0	0	27
15	35	43	175	121	6	0	4	8	11	20	1	0	13	0	3	4	83	53	41	35	1	34
0	0	0	0	0	3	1	2	1	2	0	0	0	0	0	0	0	2	0	0	7	2	14
0	0	4	0	0	6	29	8	3	12	0	0	0	0	25	0	3	1	46	0	7	0	330
0	0	24	14	0	2	4	3	11	7	0	0	0	0	0	1	0	1	0	0	9	1	12
127	46	167	54	117	56	51	53	68	33	46	115	28	15	66	0	7	131	219	43	130	26	112
0	0	0	0	0	0	0	0	0	0	0	0	0	0	0	0	0	0	0	0	0	1	0
14	0	9	0	0	9	4	0	5	0	0	0	0	9	16	0	3	0	0	5	0	3	0
0	0	0	0	0	0	0	0	0	0	0	0	0	0	0	0	0	0	0	0	0	0	0
0	0	0	0	0	0	0	0	0	0	0	0	0	0	0	0	0	0	0	0	0	0	0
5	4	42	0	16	22	4	5	5	3	0	0	0	10	22	8	17	0	42	38	21	3	4
3	0	64	0	82	36	20	22	17	22	0	16	0	68	36	0	16	21	91	33	10	10	31
0	1	0	0	6	13	1	1	5	1	15	0	0	7	0	0	0	0	0	0	0	0	0
0	0	0	0	0	0	0	0	0	0	0	0	0	0	0	0	0	0	0	0	0	0	0
0	0	0	0	0	0	0	0	0	0	0	0	0	0	0	0	0	0	0	0	0	0	0
0	0	0	0	0	9	6	2	3	6	0	0	0	0	0	0	0	7	0	0	37	1	23
247	125	144	87	12	4	11	4	8	6	0	29	0	13	1	0	0	33	3	6	19	7	16
28	0	13	48	0	1	12	2	3	2	0	0	0	0	0	0	0	8	3	6	16	1	9
0	0	0	0	0	0	0	0	0	0	0	0	0	0	0	0	0	0	0	0	0	0	0
281	77	171	36	16	10	9	7	9	7	0	39	0	1	5	0	3	63	15	5	56	11	62
0	0	0	0	0	0	0	0	0	0	0	0	0	0	0	0	0	0	0	0	0	0	0
0	0	0	0	0	1	0	0	0	0	0	0	0	0	0	0	0	0	0	0	0	0	0

E1.016	E1.017	E1.018	E1.020	E1.021	E1.024	E1.025	E1.026	E1.027	E1.029	E1.030	E1.031	E1.032	E1.033	E1.034	E1.036	E1.037	E1.038	E2.002	E2.003	E2.009	E2.010	E2.012
Esophag	Esophag	Esophag	Esophag	Esophag	Esophag	Esophag	Esophag	Esophag	Esophag	Esophag	Esophag	Esophag	Esophag	Esophag	Esophag	Esophag	Esophag	Esophag	Esophag	Esophag	Esophag	Esophag
UE	UE	UE	UE	UE	UE	UE	UE	UE	UE	UE	UE	UE	UE	UE	UE	UE	UE	ME	ME	ME	ME	ME
0	1	22	3	53	11	5	12	32	15	0	0	0	38	1	0	9	0	7	13	5	4	12
0	0	0	0	0	0	0	0	0	0	0	0	0	0	0	0	0	0	0	0	0	0	0
30	8	3	17	66	11	4	14	4	32	0	1	0	3	25	0	4	5	16	15	5	0	43
0	0	0	1	0	0	0	0	0	0	0	0	0	0	0	0	0	0	0	0	0	0	0
0	0	2	0	0	1	0	0	6	0	0	0	0	0	1	0	1	0	1	0	2	0	0
13	0	5	0	10	13	4	6	15	11	3	231	7	4	12	0	9	8	4	14	10	2	16
0	0	0	0	0	0	0	0	0	0	0	0	0	0	0	0	0	0	0	0	0	0	0
0	0	0	0	0	0	0	0	0	0	0	0	0	0	0	0	0	0	0	0	0	0	0
142	117	278	587	1375	64	25	3	50	71	111	384	107	377	270	32	362	101	93	200	89	8	97
0	0	0	0	0	0	0	0	0	0	0	0	0	0	0	0	0	0	0	0	0	0	0
0	0	10	17	0	0	0	0	0	0	0	17	0	0	0	0	0	0	0	4	3	1	2
145	18	72	68	0	0	5	4	7	4	0	0	0	0	0	1	0	16	0	0	4	5	11
0	0	0	0	0	0	0	0	0	0	0	0	0	0	0	0	0	0	0	0	0	0	0
0	0	0	0	0	2	0	0	4	0	0	0	0	0	0	0	0	0	0	0	1	0	3
29	17	74	17	0	7	33	10	15	10	0	0	0	0	15	0	1	22	16	4	20	14	43
28	15	67	0	27	7	20	13	11	4	0	12	0	136	5	2	9	29	29	31	15	1	29
0	0	0	0	0	0	0	0	1	0	0	0	0	0	0	0	0	0	0	0	0	1	1
0	0	0	0	0	0	0	0	0	0	0	0	0	0	0	0	0	0	0	0	0	0	0
0	0	0	0	0	0	0	0	0	0	0	0	0	0	0	0	0	0	0	0	1	0	0
0	0	0	0	0	1	0	0	0	0	0	0	0	0	0	0	0	0	0	0	0	0	0
0	0	0	0	0	0	0	0	0	0	0	0	0	0	0	0	0	0	0	0	1	0	0
0	0	0	0	2	0	0	2	0	1	0	0	0	0	0	0	0	0	0	0	0	0	1
0	0	0	0	0	0	0	0	0	0	0	0	0	0	0	0	0	0	0	0	0	0	0
0	0	5	0	0	0	1	0	0	0	0	0	0	0	0	0	0	0	0	0	2	0	14
0	0	0	0	0	0	1	0	0	0	0	0	0	0	0	0	0	0	0	0	0	0	1
144	57	47	35	554	100	110	137	119	140	396	213	99	376	256	12	114	70	532	341	82	25	53
73	12	68	45	27	26	22	9	20	17	72	55	0	132	27	4	11	12	66	55	8	3	22
0	0	0	0	0	3	6	4	12	6	0	0	0	0	0	0	0	0	0	0	0	29	36
0	0	0	0	0	0	0	0	0	0	0	0	0	0	0	0	0	0	0	0	0	0	1
0	0	1	0	4	2	3	0	0	0	7	0	0	317	0	0	0	2	0	29	0	1	0
0	0	0	0	0	0	0	0	0	0	0	0	0	0	0	0	0	0	0	0	0	0	0
0	0	0	0	0	0	0	0	0	0	0	0	0	0	0	0	0	0	0	0	0	0	0
0	0	0	0	0	0	0	0	0	0	0	0	0	0	0	0	0	0	0	0	0	0	2
0	0	0	0	0	0	0	0	0	0	0	0	0	0	0	0	0	0	0	0	0	0	0
0	0	0	0	0	3	19	4	9	19	0	0	0	0	12	0	0	10	7	0	7	12	44
4	0	0	0	0	0	8	20	1	0	0	0	0	0	10	0	0	5	0	0	7	2	12
0	0	1	0	0	1	7	0	1	0	0	0	0	0	0	0	0	0	0	0	0	0	5

E1.016	E1.017	E1.018	E1.020	E1.021	E1.024	E1.025	E1.026	E1.027	E1.029	E1.030	E1.031	E1.032	E1.033	E1.034	E1.036	E1.037	E1.038	E2.002	E2.003	E2.009	E2.010	E2.012
Esophag	Esophag	Esophag	Esophag	Esophag	Esophag	Esophag	Esophag	Esophag	Esophag	Esophag	Esophag	Esophag	Esophag	Esophag	Esophag	Esophag	Esophag	Esophag	Esophag	Esophag	Esophag	Esophag
UE	UE	UE	UE	UE	UE	UE	UE	UE	UE	UE	UE	UE	UE	UE	UE	UE	UE	ME	ME	ME	ME	ME
9	1	19	0	0	14	127	13	31	19	0	0	0	0	0	0	0	11	0	0	83	87	156
0	0	0	0	0	0	1	0	1	0	0	0	0	0	0	0	0	0	0	0	0	0	7
494	160	701	225	43	24	342	137	61	93	108	110	295	127	628	28	71	428	273	113	56	170	646
0	0	0	0	0	0	0	0	0	0	0	0	0	0	0	0	0	0	0	0	0	0	0
0	0	0	0	0	0	0	0	0	0	0	0	0	0	0	0	0	0	0	0	0	0	2
0	0	0	0	0	0	0	0	0	0	0	0	0	0	0	0	0	0	0	0	0	0	0
0	0	0	0	0	0	0	0	0	0	0	0	0	0	0	0	0	0	0	0	0	0	0
0	0	0	0	0	0	0	0	0	0	0	0	0	0	0	0	0	0	0	0	0	0	0
0	0	0	0	0	0	1	0	0	1	0	0	0	0	0	0	0	0	0	0	1	0	0
0	0	0	0	0	0	0	0	0	0	0	0	0	14	0	0	0	0	0	0	0	0	1
0	0	0	0	0	0	0	0	0	0	0	0	0	0	0	0	0	0	0	0	0	0	1
68	29	69	36	13	4	32	17	4	7	33	24	26	24	62	5	5	76	88	14	3	12	54
0	0	0	1	0	0	0	0	0	0	0	0	0	0	0	1	0	0	0	0	0	0	0
12	5	0	0	0	0	9	2	0	4	0	8	0	17	1	1	0	0	4	4	0	3	10
29	11	12	56	0	8	236	101	23	19	18	56	178	0	42	0	4	41	102	18	89	89	186
0	0	0	0	0	0	6	5	1	1	0	0	0	0	0	0	0	0	0	0	2	1	12
6	0	5	0	0	0	3	6	1	3	0	0	0	0	0	0	0	0	0	0	0	2	4
0	0	0	13	0	0	0	0	0	0	0	0	0	0	0	0	0	0	0	0	1	0	1
0	0	0	0	0	0	0	0	0	0	0	0	0	0	0	0	0	0	0	0	0	1	0
0	0	0	0	0	0	7	0	0	0	0	0	0	0	21	0	0	0	0	0	0	0	6
0	0	0	0	0	0	0	0	0	0	0	0	0	0	0	0	0	0	0	0	0	0	0
0	0	0	0	0	0	0	0	0	0	0	0	0	0	0	0	0	0	0	0	0	0	1
14	13	55	3	1	4	33	17	9	8	6	90	142	66	0	4	2	26	66	16	2	3	20
0	0	1	0	0	3	16	9	10	2	0	5	31	0	108	2	5	13	15	5	4	8	21
0	0	0	0	0	0	0	0	0	0	0	0	0	0	0	0	0	0	0	0	0	0	0
0	0	0	0	0	0	0	0	0	2	0	0	0	0	0	0	0	0	0	0	0	0	0
0	0	0	0	0	0	0	0	0	0	0	0	0	0	0	0	0	0	0	0	0	0	0
0	0	0	0	0	0	0	0	0	0	0	0	0	0	0	0	0	0	0	0	0	0	0
0	0	0	0	0	0	0	0	0	1	0	0	0	0	0	0	0	0	0	0	0	0	0
0	0	0	0	0	1	12	8	1	7	32	17	0	19	103	13	7	83	88	33	3	0	47
0	0	0	0	0	0	0	0	0	0	0	0	0	0	0	0	0	0	0	0	0	0	0
0	0	32	0	0	0	1	1	0	3	0	0	0	0	0	0	0	0	0	0	0	2	1
0	0	0	0	0	0	0	0	0	0	0	0	0	0	0	0	0	0	0	0	0	0	0
0	0	6	0	0	0	0	0	0	4	0	0	0	0	0	0	0	0	0	0	0	0	0
0	0	0	0	6	0	1	0	1	1	0	0	0	0	0	0	0	0	15	0	0	0	2
0	0	0	0	0	0	0	0	0	0	0	0	0	0	0	0	0	0	0	0	0	0	0
0	0	0	0	0	0	0	0	0	0	0	0	0	0	0	0	0	0	0	0	0	0	0

E1.016	E1.017	E1.018	E1.020	E1.021	E1.024	E1.025	E1.026	E1.027	E1.029	E1.030	E1.031	E1.032	E1.033	E1.034	E1.036	E1.037	E1.038	E2.002	E2.003	E2.009	E2.010	E2.012
Esophag	Esophag	Esophag	Esophag	Esophag	Esophag	Esophag	Esophag	Esophag	Esophag	Esophag	Esophag	Esophag	Esophag	Esophag	Esophag	Esophag	Esophag	Esophag	Esophag	Esophag	Esophag	Esophag
UE	UE	UE	UE	UE	UE	UE	UE	UE	UE	UE	UE	UE	UE	UE	UE	UE	UE	ME	ME	ME	ME	ME
0	0	2	0	0	0	0	0	0	0	0	0	0	0	0	0	0	0	0	0	0	0	0
0	0	4	0	0	0	2	0	0	0	0	0	0	0	0	0	0	0	0	0	0	0	1
0	0	0	0	0	0	0	1	0	1	0	0	0	0	0	0	0	0	0	0	0	0	1
0	0	0	0	0	0	0	0	0	0	0	0	0	1	0	0	0	0	0	0	0	0	0
7	0	3	10	0	0	0	3	2	0	0	0	0	113	0	0	0	15	0	0	0	1	8
0	0	0	0	0	0	0	0	0	1	0	0	0	0	0	0	0	18	0	0	0	0	3
0	0	74	0	0	0	1	5	3	0	0	0	0	0	0	0	0	0	0	0	0	0	1
0	0	0	0	0	0	0	0	0	0	0	0	0	0	0	0	0	0	0	0	0	0	0
12	0	8	0	0	1	1	0	4	0	0	0	0	87	0	0	0	3	0	0	2	2	3
4	0	1	0	0	1	0	0	0	0	0	0	0	0	0	0	0	0	0	0	55	0	5
1	0	0	0	0	0	2	2	0	0	0	0	0	0	0	0	0	0	0	0	0	0	16
0	0	0	0	0	0	0	0	0	0	0	0	0	0	19	0	0	0	0	0	0	0	0
1	0	0	0	0	0	0	0	0	0	0	0	0	0	0	0	0	0	0	0	0	0	0
0	0	0	0	0	0	8	1	0	0	0	0	0	0	0	0	0	0	0	0	1	0	95
3	2	151	0	1	1	2	3	1	0	5	9	21	0	3	0	0	13	9	2	0	0	5
0	0	0	0	0	0	0	0	0	0	0	0	0	0	0	0	0	0	0	0	0	0	3
0	0	0	0	0	0	0	0	1	0	0	0	0	0	0	0	0	0	0	0	0	0	0
0	0	0	0	0	0	0	0	0	0	0	0	0	0	0	0	0	0	0	0	0	0	0
0	0	367	0	0	0	1	0	0	0	0	0	0	0	0	0	0	0	0	0	0	0	1
0	10	14	0	3	2	16	15	15	13	12	0	0	1	25	0	0	5	32	0	5	14	100
0	0	0	0	0	0	0	0	0	0	0	0	0	0	0	0	0	0	0	0	0	0	0
0	0	0	0	0	0	0	0	0	0	0	0	0	0	0	0	0	0	0	0	0	0	0
0	0	0	0	0	0	0	0	0	0	0	0	0	0	0	0	0	0	0	0	0	0	0
0	0	0	0	0	0	0	0	0	0	0	0	0	0	0	0	0	0	0	0	0	0	0
0	0	31	0	0	1	2	3	9	7	8	0	0	6	0	0	4	0	0	0	1	0	4
0	0	0	0	0	0	0	3	0	1	0	0	0	0	1	2	0	0	0	0	0	1	4
0	0	211	0	0	0	0	20	0	2	0	0	0	0	7	0	0	10	0	0	0	1	1
19	22	52	41	1	30	128	84	38	46	0	27	0	16	81	0	8	58	70	22	6	62	214
1	0	0	0	2	19	55	50	18	2	6	0	0	0	129	0	2	16	3	6	28	18	153
10	94	1073	48	14	219	9533	7083	1065	71	58	101	75	63	113	8	18	86	89	17	7	5066	5073
0	0	0	0	0	1	3	0	1	2	0	0	0	0	0	0	0	0	0	0	0	0	6
0	0	2	0	0	1	22	5	4	0	0	0	0	12	0	0	0	0	0	0	0	4	13
0	1	15	0	0	0	12	3	3	0	0	0	25	20	2	0	0	1	0	0	0	2	7
0	0	0	0	0	0	1	0	0	0	0	0	0	0	0	0	0	0	0	0	0	0	0
0	0	0	0	0	0	0	0	0	0	0	0	0	0	0	0	0	0	0	0	0	0	0
0	0	0	0	0	1	0	8	3	0	0	0	0	0	0	0	0	0	0	0	0	0	5
30	2	11	1	0	0	0	2	2	0	0	0	0	0	1	0	0	0	7	0	5	0	3

E1.016	E1.017	E1.018	E1.020	E1.021	E1.024	E1.025	E1.026	E1.027	E1.029	E1.030	E1.031	E1.032	E1.033	E1.034	E1.036	E1.037	E1.038	E2.002	E2.003	E2.009	E2.010	E2.012
Esophag	Esophag	Esophag	Esophag	Esophag	Esophag	Esophag	Esophag	Esophag	Esophag	Esophag	Esophag	Esophag	Esophag	Esophag	Esophag	Esophag	Esophag	Esophag	Esophag	Esophag	Esophag	Esophag
UE	UE	UE	UE	UE	UE	UE	UE	UE	UE	UE	UE	UE	UE	UE	UE	UE	UE	ME	ME	ME	ME	ME
0	0	0	0	0	0	0	0	0	0	0	0	0	0	0	0	0	0	0	0	0	0	0
0	0	0	0	0	2	24	15	4	1	0	0	0	0	0	0	0	0	0	0	11	11	11
0	0	1	0	0	0	0	0	0	0	0	0	0	0	0	0	0	0	0	0	0	0	0
0	14	17	34	28	39	17	27	25	40	9	18	0	70	0	0	16	6	23	34	19	12	16
0	0	0	0	0	0	0	0	0	0	0	0	0	0	0	0	0	0	0	0	0	0	1
0	0	0	0	0	0	0	0	0	0	0	12	0	0	0	0	0	0	0	0	0	0	0
0	0	126	0	0	1	12	7	4	7	2	0	0	0	10	0	2	19	0	0	5	2	12
1	0	2	0	0	0	5	3	2	6	0	0	0	0	0	6	2	13	4	2	0	0	13
0	0	0	0	0	0	0	0	0	0	0	0	0	0	0	0	0	0	0	0	0	0	3
0	0	0	0	0	0	5	3	3	1	0	0	0	0	25	0	0	0	0	0	7	5	10
0	0	0	0	0	0	0	0	0	0	0	0	0	0	0	0	0	0	0	0	0	0	0
0	0	0	0	0	0	1	0	0	0	0	0	0	0	0	0	0	0	0	0	0	0	0
0	0	0	0	0	0	0	0	0	0	0	0	0	0	0	0	0	0	28	0	0	0	4
0	0	0	0	0	0	0	0	0	0	0	0	0	0	0	0	0	0	0	0	0	0	0
0	0	1	0	0	0	0	0	0	2	0	0	0	0	2	0	1	6	0	0	0	2	3
0	0	2	0	0	0	0	0	0	1	17	2	10	6	24	0	1	0	0	0	0	0	0
0	0	0	0	0	0	0	0	0	0	0	0	0	0	0	0	0	0	0	0	0	0	0
0	0	0	0	0	0	0	0	0	0	0	0	0	0	1	0	0	0	0	0	0	0	0
0	0	0	0	0	0	1	0	0	0	0	0	0	0	0	0	0	0	0	0	0	0	0
0	0	4	0	0	0	0	0	0	0	0	0	0	0	0	0	1	0	0	0	0	0	0
0	0	0	0	0	0	3	0	0	1	0	0	0	7	0	0	0	3	7	0	1	0	2
0	0	7	0	0	10	3	13	7	8	0	0	0	36	3	0	0	12	0	0	0	2	25
16	12	70	17	20	45	21	19	17	29	36	1	5	52	149	9	22	93	54	4	17	8	68
0	0	2	2	0	7	13	10	7	17	0	0	0	22	329	3	2	127	2	1	12	6	8
0	0	29	0	0	0	0	0	1	0	0	0	0	0	0	0	0	0	0	0	0	1	2
6	28	11	3	0	0	1	0	1	0	44	165	157	0	27	0	0	17	50	2	0	0	0
0	19	55	39	0	0	2	1	0	5	0	5	25	6	43	2	0	15	17	0	1	0	6
1	2	602	0	3	5	2	6	11	8	0	0	26	65	457	2	2	148	30	0	291	7	18
0	0	28	0	0	0	3	0	0	0	0	0	0	0	0	0	0	0	0	0	0	0	2
4	0	3	0	0	0	0	0	1	0	0	0	0	0	0	1	0	0	0	0	0	0	0
0	0	0	0	0	0	0	0	0	0	0	0	0	0	0	0	0	0	0	0	0	0	0
0	0	0	0	0	0	1	1	1	0	0	0	0	0	0	1	0	0	0	0	0	0	3
0	7	4	4	13	10	2	7	4	8	8	40	0	24	22	1	0	4	11	0	13	2	16
0	0	11	0	0	2	10	0	5	2	0	0	0	5	0	0	0	8	50	0	2	12	4
1133	574	1562	88	1268	755	392	310	278	394	634	1144	263	2123	453	300	165	767	1264	1198	1103	458	1239
0	0	0	0	1	4	3	7	5	2	1	5	0	30	0	0	0	0	10	0	5	2	3
33	21	37	2	38	35	28	30	21	38	79	181	0	118	5	2	14	50	156	144	57	3	38

E1.016	E1.017	E1.018	E1.020	E1.021	E1.024	E1.025	E1.026	E1.027	E1.029	E1.030	E1.031	E1.032	E1.033	E1.034	E1.036	E1.037	E1.038	E2.002	E2.003	E2.009	E2.010	E2.012
Esophag	Esophag	Esophag	Esophag	Esophag	Esophag	Esophag	Esophag	Esophag	Esophag	Esophag	Esophag	Esophag	Esophag	Esophag	Esophag	Esophag	Esophag	Esophag	Esophag	Esophag	Esophag	Esophag
UE	UE	UE	UE	UE	UE	UE	UE	UE	UE	UE	UE	UE	UE	UE	UE	UE	UE	ME	ME	ME	ME	ME
0	0	0	0	0	0	0	0	0	0	0	0	0	0	1	0	0	0	0	0	0	0	0
0	0	0	0	0	0	0	0	0	0	0	0	0	0	0	0	0	0	0	0	0	0	0
0	0	7	0	1	0	2	0	0	1	0	0	0	0	14	0	0	0	0	0	0	1	12
0	0	0	0	0	0	0	0	0	0	0	0	0	0	0	0	0	0	0	0	1	0	0
0	0	0	0	0	0	0	0	0	0	0	0	0	0	0	0	0	0	0	0	0	0	0
0	0	12	5	0	0	1	0	0	0	0	0	0	0	0	3	1	12	18	0	0	0	3
0	0	0	0	0	0	3	1	0	0	0	0	0	0	0	0	0	0	0	0	0	0	4
0	0	12	0	0	0	1	0	0	0	2	0	0	0	0	0	0	0	0	0	0	0	0
0	0	54	0	0	0	2	0	0	0	0	0	0	0	0	0	1	0	0	0	0	0	3
0	0	0	0	3	0	0	0	0	0	0	0	0	0	0	0	0	0	7	3	6	0	0
0	0	0	0	0	0	0	0	0	0	0	0	0	0	0	0	0	0	0	0	0	0	0
0	0	0	0	0	0	0	0	0	0	0	0	0	0	0	0	0	0	0	0	2	0	3
15	8	12	0	0	1	1	1	3	0	0	0	0	0	0	0	0	2	0	1	3	0	0
64	0	19	0	0	1	0	0	0	2	0	0	0	0	0	0	4	5	5	0	1	4	0
0	0	0	0	0	0	0	0	0	1	0	0	0	0	0	0	0	0	0	0	1	0	1
0	0	0	0	0	0	0	0	0	0	0	0	0	0	0	0	0	0	0	0	0	0	0
0	0	0	0	0	0	0	0	0	0	0	0	0	0	0	0	0	0	0	0	0	0	0
0	0	6	0	0	0	0	0	0	0	0	0	0	0	0	0	0	0	0	0	0	0	0
0	0	0	0	0	0	0	0	0	0	0	0	0	0	0	0	0	0	0	0	0	0	0
0	0	0	0	0	0	0	0	0	0	0	0	0	0	0	0	0	0	0	0	0	0	0
0	14	72	189	294	55	43	44	98	51	75	1140	216	95	196	0	51	37	56	155	52	6	58
0	0	0	0	0	0	1	0	0	3	0	0	0	0	0	0	0	0	15	0	5	0	0
13	179	40	0	347	0	29	67	0	0	20	0	0	0	3	0	0	0	9	0	0	1	92
0	0	2	0	0	0	0	0	0	0	0	0	0	0	0	0	0	0	0	0	0	0	0
0	0	0	0	0	0	0	0	0	0	0	0	0	0	12	0	0	0	0	0	0	0	0
0	0	0	0	0	0	0	0	0	0	0	0	0	0	0	0	0	0	0	0	0	0	0
0	0	7	0	0	0	0	0	0	0	0	0	0	0	0	0	0	0	11	0	1	0	0
0	2	0	1	0	3	6	0	0	2	0	0	0	0	0	0	0	0	0	0	1	3	3
0	0	0	0	0	0	0	0	0	0	0	0	0	0	0	0	0	0	0	0	0	0	2
0	0	0	0	0	0	0	0	0	0	0	0	0	0	0	0	0	0	0	0	0	0	0
0	0	0	0	0	0	0	0	0	0	0	0	0	0	0	0	0	0	0	0	0	0	0
0	0	0	0	0	0	3	0	0	1	0	0	0	0	0	0	0	0	0	0	0	0	1
0	0	0	0	0	0	0	0	0	0	0	0	0	0	0	0	0	0	0	0	0	0	0
0	0	0	0	0	0	0	0	0	0	0	0	0	0	0	0	0	0	0	0	0	0	0
0	0	0	0	0	0	3	1	0	0	0	0	0	0	0	0	0	0	0	0	0	0	1
0	0	1	0	3	2	1	3	14	6	0	0	0	21	0	0	0	8	0	1	6	0	4
0	0	0	0	0	0	0	0	0	0	0	0	0	0	0	0	0	0	0	0	2	0	0

E1.016	E1.017	E1.018	E1.020	E1.021	E1.024	E1.025	E1.026	E1.027	E1.029	E1.030	E1.031	E1.032	E1.033	E1.034	E1.036	E1.037	E1.038	E2.002	E2.003	E2.009	E2.010	E2.012
Esophag	Esophag	Esophag	Esophag	Esophag	Esophag	Esophag	Esophag	Esophag	Esophag	Esophag	Esophag	Esophag	Esophag	Esophag	Esophag	Esophag	Esophag	Esophag	Esophag	Esophag	Esophag	Esophag
UE	UE	UE	UE	UE	UE	UE	UE	UE	UE	UE	UE	UE	UE	UE	UE	UE	UE	ME	ME	ME	ME	ME
0	0	0	0	0	0	0	0	2	0	0	0	0	0	0	0	0	0	0	0	0	0	0
0	0	8	0	0	0	0	0	0	0	0	0	0	0	0	0	0	0	0	0	0	0	0
0	0	2	0	0	0	8	4	0	1	0	0	0	0	0	3	0	0	0	0	2	1	6
0	0	0	0	0	1	4	0	0	3	0	0	0	0	0	0	0	0	0	0	1	3	4
0	0	0	0	0	0	0	0	0	0	0	0	0	0	0	0	0	0	0	0	0	0	1
0	0	0	0	0	0	0	0	0	0	0	0	0	0	0	0	0	0	0	0	0	0	0
0	0	0	0	0	0	0	0	0	0	0	0	0	0	0	0	0	0	0	0	0	0	0
0	0	0	0	0	1	1	0	0	0	0	0	0	0	0	0	0	0	0	0	0	0	1
0	0	0	0	0	0	0	0	0	0	0	0	0	0	4	0	0	0	0	0	0	0	0
0	0	0	0	0	0	0	0	1	1	0	0	0	0	0	0	0	0	0	0	0	0	0
1	0	5	0	0	0	2	3	4	1	0	0	0	0	0	4	1	0	1	0	2	0	13
1228	93	1094	114	26	65	695	550	185	211	71	29	595	108	206	9	197	248	2253	49	254	421	772
0	0	0	0	0	0	0	0	0	0	0	0	0	0	0	0	0	0	0	0	0	0	1
2	0	6	0	3	0	0	0	0	0	0	0	0	0	0	3	0	0	0	0	0	0	0
0	0	0	0	0	0	0	0	0	0	0	0	0	0	0	0	0	0	0	0	0	0	0
0	0	0	0	0	0	0	0	0	0	0	0	0	0	0	0	0	0	0	0	0	0	0
0	0	0	0	0	0	0	0	0	0	0	0	0	0	8	0	0	0	0	0	0	0	0
0	0	0	0	0	1	0	0	0	0	0	0	0	0	0	0	0	0	0	0	0	0	2
1043	12364	324	0	8253	187	376	393	90	2072	6103	285	675	67	3	13672	5	1911	171	3701	2412	306	140
11	4	35	5	86	39	20	20	108	27	50	245	1	96	26	19	11	93	96	143	39	12	46
328	503	1038	299	881	1758	135	147	676	122	1069	482	508	3677	563	432	117	700	557	1862	553	91	134
0	0	0	0	0	0	0	0	0	0	0	0	0	0	0	0	0	0	0	0	0	0	0
0	0	0	0	0	0	0	0	0	0	0	0	0	0	0	0	44	0	0	0	0	0	0
0	1	0	0	1	0	1	2	2	3	0	0	0	0	0	0	0	0	0	1	0	2	9
0	0	0	0	0	0	0	0	0	0	0	0	0	0	0	0	0	0	0	0	0	0	0
97	9	36	0	2	2	32	5	8	11	0	3	32	14	10	2	3	305	77	5	4	15	44
0	0	19	0	0	0	0	0	1	0	0	0	0	0	0	0	0	0	0	0	0	0	0
31	6	10	0	0	2	30	3	20	25	0	5	97	0	8	0	0	10	18	100	18	2	47
0	0	51	62	0	8	7	2	14	1	25	36	0	0	33	37	2	43	107	35	1	25	8
0	0	0	0	0	0	0	0	1	0	0	0	0	0	0	0	0	0	0	0	0	0	0
0	0	0	0	0	0	3	0	0	0	0	0	0	0	0	0	0	0	0	0	0	0	1
0	0	0	0	0	0	0	0	0	0	0	0	0	0	0	0	0	0	0	0	0	0	0
6	0	10	0	0	1	4	0	0	5	0	0	0	0	0	0	0	0	0	0	1	0	4
34	21	29	28	0	30	344	217	122	283	0	52	0	1	566	3	2	147	27	0	124	160	248
0	0	0	0	0	0	13	2	0	2	0	0	0	0	1	0	0	0	0	0	0	0	7
0	0	0	0	1	5	21	9	1	2	0	0	0	4	546	0	0	126	0	0	0	3	12
0	0	0	0	0	0	0	0	0	0	0	0	0	0	0	0	0	0	0	0	0	0	0

E1.016	E1.017	E1.018	E1.020	E1.021	E1.024	E1.025	E1.026	E1.027	E1.029	E1.030	E1.031	E1.032	E1.033	E1.034	E1.036	E1.037	E1.038	E2.002	E2.003	E2.009	E2.010	E2.012
Esophag	Esophag	Esophag	Esophag	Esophag	Esophag	Esophag	Esophag	Esophag	Esophag	Esophag	Esophag	Esophag	Esophag	Esophag	Esophag	Esophag	Esophag	Esophag	Esophag	Esophag	Esophag	Esophag
UE	UE	UE	UE	UE	UE	UE	UE	UE	UE	UE	UE	UE	UE	UE	UE	UE	UE	ME	ME	ME	ME	ME
0	0	0	0	0	0	0	0	0	0	0	0	0	0	0	0	0	0	0	0	0	0	0
0	0	0	0	0	0	0	0	0	0	0	0	0	0	0	0	0	0	0	0	0	0	0
0	0	0	0	0	0	0	0	0	0	0	0	0	0	0	0	0	0	0	0	0	0	0
0	0	0	0	0	0	0	0	0	0	0	0	0	0	0	0	0	0	0	0	0	0	0
11	0	0	0	1	1	0	1	3	6	0	0	0	0	0	0	2	0	0	0	0	0	14
0	0	0	0	0	1	9	7	3	0	0	0	0	0	0	0	0	0	0	0	3	0	8
0	0	15	0	0	0	0	0	0	0	0	0	0	0	0	0	0	0	0	0	0	0	0
0	0	0	0	0	0	2	1	0	0	11	21	0	0	0	0	0	4	0	2	0	0	3
0	0	0	0	0	0	0	0	0	0	0	0	0	0	0	0	0	0	0	0	0	0	0
0	0	0	0	0	0	0	0	0	0	0	0	0	0	0	0	0	9	0	0	0	0	0
0	0	0	0	0	0	0	0	1	0	0	0	0	0	23	0	0	0	0	0	0	0	1
45	15	0	7	2	12	181	142	16	8	10	12	63	133	4620	10	7	1167	36	5	99	77	141
0	0	0	0	0	0	0	0	0	0	0	0	0	0	0	0	0	0	0	0	0	0	0
0	0	0	0	0	1	7	1	2	7	0	0	0	0	0	0	0	2	0	0	1	1	9
0	6	21	0	1	0	12	2	2	2	0	11	0	64	1721	0	2	532	6	0	3	2	7
0	0	0	0	0	0	3	0	0	0	0	0	0	0	0	0	0	0	0	0	0	1	1
0	0	0	0	0	0	1	0	0	0	0	0	0	0	0	0	0	0	0	0	0	0	0
0	0	0	0	0	0	0	0	0	0	0	0	0	0	0	0	0	0	0	0	0	0	0
0	6	95	10	10	37	17	19	32	15	2	153	0	36	68	3	25	7	27	34	52	3	4
0	0	0	0	0	0	0	0	0	0	0	0	0	0	0	0	0	0	0	0	0	0	0
0	0	0	0	2	2	0	0	2	0	1	0	0	4	0	0	2	0	1	1	7	1	1
0	0	0	0	0	0	0	0	0	0	0	0	0	0	0	0	0	0	0	0	0	0	0
0	0	0	0	0	0	0	0	0	0	0	0	0	0	0	0	0	0	0	0	0	0	0
0	9	0	0	0	0	3	0	0	0	5	0	0	0	0	0	4	8	0	5	68	0	0
0	0	4	0	30	6	4	16	34	11	0	0	94	0	0	0	5	0	24	33	7	14	84
0	0	0	0	0	1	0	6	2	0	0	0	0	0	0	0	0	0	0	0	0	3	7
0	0	0	0	0	0	0	0	0	0	0	0	0	0	0	0	0	0	0	0	0	0	0
0	0	0	0	0	0	0	0	0	0	0	0	0	0	0	0	0	0	0	0	0	0	0
0	0	0	3	24	0	2	3	6	1	0	0	0	4	0	2	1	16	0	5	4	0	4
23	0	6	0	0	6	27	0	141	1	5	48	0	1	0	0	7	0	0	0	2	1	343
0	0	0	0	0	0	0	0	0	0	0	0	0	0	0	0	0	0	0	0	0	0	0
26	33	137	17	7	0	0	0	0	0	0	0	0	0	0	0	0	22	0	0	1	0	0

E2.013	E2.014	E2.016	E2.017	E2.018	E2.019	E2.020	E2.021	E2.024	E2.025	E2.026	E2.027	E2.029	E2.030	E2.031	E2.032	E2.033	E2.034	E2.036	E2.037	E2.038	E3.006	E3.009
Esophag	Esophag	Esophag	Esophag	Esophag	Esophag	Esophag	Esophag	Esophag	Esophag	Esophag	Esophag	Esophag	Esophag	Esophag	Esophag	Esophag	Esophag	Esophag	Esophag	Esophag	Esophag	Esophag
ME	ME	ME	ME	ME	ME	ME	ME	ME	ME	ME	ME	ME	ME	ME	ME	ME	ME	ME	ME	ME	LE	LE
0	0	0	0	0	0	0	0	0	0	0	0	0	0	0	0	0	0	0	0	0	0	0
7230	14232	17304	7783	17838	13426	15530	11840	11934	14474	7351	19942	14365	29345	9545	31262	12839	11263	25800	18397	19564	19963	7107
1	2	0	21	17	42	2	0	6	202	26	99	236	1	1	1	0	0	4	1	2	0	9
0	0	0	0	39	0	0	0	0	2	1	0	1	4	0	0	0	82	0	0	27	0	0
0	0	0	0	0	0	0	0	2	23	3	10	7	0	0	0	0	0	0	0	0	0	4
87	466	45	282	132	567	79	138	108	162	395	246	571	528	149	828	161	370	89	662	711	564	159
0	0	0	0	0	0	0	0	2	8	3	2	1	0	0	0	0	0	0	1	0	5	1
647	2098	8618	865	4093	1298	4201	2914	491	2052	1031	1781	3110	1596	1557	3217	1282	2566	1220	2017	1399	1700	944
0	0	0	0	0	0	0	0	0	0	0	0	0	0	0	0	0	0	0	0	0	0	0
0	0	0	0	0	0	0	0	1	1	0	0	0	0	0	0	0	0	0	0	0	0	0
0	0	0	0	0	0	0	0	0	0	0	0	0	0	0	0	0	0	0	0	0	0	0
0	0	0	0	0	0	0	0	0	0	0	0	0	0	0	0	0	64	1	0	0	0	0
0	14	0	0	0	0	2	0	0	0	1	1	0	3	0	0	0	15	1	0	0	5	0
0	0	0	0	0	0	0	0	0	0	0	0	0	0	0	0	0	0	0	0	0	0	0
0	0	0	0	0	0	0	0	0	0	0	0	0	0	0	0	0	0	0	0	0	0	0
5744	5471	2899	3620	8682	4367	10339	2012	2289	2892	3021	3741	4939	11920	6299	9831	7104	4548	8061	14851	4133	8487	3963
96	485	573	0	430	211	47	206	103	153	96	248	487	70	104	197	325	118	68	59	233	249	29
0	0	0	0	0	0	0	0	0	0	0	0	0	0	0	0	0	0	0	0	0	0	0
0	0	0	0	0	0	0	0	2	55	3	23	2	3	0	0	0	0	2	0	47	37	4
0	0	0	0	0	0	0	0	0	0	0	0	0	0	0	0	0	0	0	0	0	0	0
0	0	0	0	0	0	0	0	0	0	0	0	0	0	0	0	0	0	0	0	0	0	0
544	5469	4880	2665	4238	6704	846	6473	8826	8394	2689	13020	4259	15121	1331	16993	3748	2890	16046	604	12695	8787	1815
29	15	0	0	91	0	10	7	2	8	9	68	31	21	62	78	53	55	12	32	1	46	29
33	0	0	0	0	7	0	0	2	6	3	22	8	1	0	64	0	0	4	0	0	24	1
2	0	0	0	0	0	1	0	0	0	0	2	0	0	0	0	0	0	5	9	0	1	3
19	27	0	184	71	0	0	35	22	42	22	29	6	17	0	0	0	56	1	3	0	7	63
0	0	0	0	0	0	0	0	0	0	0	0	0	0	0	0	0	0	0	0	0	0	0
0	0	0	0	0	0	0	0	0	0	0	1	0	0	0	0	0	0	0	0	0	0	0
28	162	289	17	40	199	3	55	78	472	47	649	707	51	42	53	132	232	266	155	316	51	81
0	23	0	46	5	31	0	0	0	0	0	0	0	8	0	0	34	267	20	3	0	0	2
0	0	0	83	0	0	0	0	0	2	1	0	0	1	0	0	0	0	0	0	0	0	0
0	0	0	0	0	0	0	0	0	0	0	0	0	0	0	0	0	0	0	0	0	0	0
0	0	0	0	39	0	0	0	0	2	1	0	1	4	0	0	0	82	0	0	27	0	0
0	0	0	0	0	0	0	0	0	0	0	0	0	0	0	0	0	0	0	0	0	0	0
0	0	0	0	0	0	0	0	0	0	0	0	0	0	0	0	0	0	0	0	0	0	0
0	0	0	0	0	0	0	0	0	0	0	5	0	0	0	0	0	0	0	0	0	0	0

E2.013	E2.014	E2.016	E2.017	E2.018	E2.019	E2.020	E2.021	E2.024	E2.025	E2.026	E2.027	E2.029	E2.030	E2.031	E2.032	E2.033	E2.034	E2.036	E2.037	E2.038	E3.006	E3.009
Esophag	Esophag	Esophag	Esophag	Esophag	Esophag	Esophag	Esophag	Esophag	Esophag	Esophag	Esophag	Esophag	Esophag	Esophag	Esophag	Esophag	Esophag	Esophag	Esophag	Esophag	Esophag	Esophag
ME	ME	ME	ME	ME	ME	ME	ME	ME	ME	ME	ME	ME	ME	ME	ME	ME	ME	ME	ME	ME	LE	LE
0	0	0	0	0	0	0	0	0	0	0	0	0	0	0	0	0	0	0	0	0	0	0
0	0	0	0	0	0	0	0	2	23	3	5	7	0	0	0	0	0	0	0	0	0	4
0	0	0	0	0	0	0	0	0	0	0	0	0	0	0	0	0	0	0	0	0	0	0
0	0	0	0	0	50	0	0	1	0	0	0	0	0	0	0	0	0	5	0	0	0	0
86	465	45	282	131	517	70	123	103	135	370	186	545	526	149	828	161	370	79	659	711	564	152
1	1	0	0	1	0	9	15	4	27	25	60	26	2	0	0	0	0	2	3	0	0	7
0	0	0	0	0	0	0	0	0	0	0	0	0	0	0	0	0	0	0	0	0	0	0
0	0	0	0	0	0	0	0	0	0	0	0	0	0	0	0	0	0	0	0	0	0	0
0	0	0	0	0	0	0	0	0	0	0	0	0	0	0	0	0	0	3	0	0	0	0
0	0	0	0	0	0	0	0	2	8	3	2	1	0	0	0	0	0	0	1	0	5	1
0	0	0	0	0	0	0	0	0	0	0	0	0	0	0	0	0	0	0	0	0	0	0
37	45	14	0	56	0	2	0	3	129	5	29	152	26	0	90	0	102	5	6	21	4	23
517	1927	8504	814	3855	1291	4188	2784	402	1785	827	1541	2694	1381	1413	2682	1086	2464	1178	1934	1141	1481	577
0	0	0	0	0	0	0	0	0	1	0	0	0	3	12	0	0	0	0	0	0	0	0
93	126	100	31	182	7	11	130	85	135	199	204	261	180	132	445	196	0	37	77	237	209	344
0	0	0	20	0	0	0	0	1	2	0	7	3	6	0	0	0	0	0	0	0	6	0
0	0	0	0	0	0	0	0	0	0	0	0	0	0	0	0	0	0	0	0	0	0	0
0	0	0	0	0	0	0	0	1	1	0	0	0	0	0	0	0	0	0	0	0	0	0
0	0	0	0	0	0	0	0	0	0	0	0	0	0	0	0	0	0	0	0	0	0	0
0	0	0	0	0	0	0	0	0	0	0	0	0	0	0	0	0	0	0	0	0	0	0
0	0	0	0	0	0	0	0	0	0	0	0	0	0	0	0	0	0	1	0	0	0	0
0	0	0	0	0	0	0	0	0	0	0	0	0	0	0	0	0	64	0	0	0	0	0
0	0	0	0	0	0	0	0	0	0	0	0	0	0	0	0	0	0	0	0	0	0	0
0	0	0	0	0	0	0	0	0	0	0	0	0	0	0	0	0	0	0	0	0	0	0
0	0	0	0	0	0	0	0	0	0	0	0	0	0	0	0	0	0	0	0	0	0	0
0	0	0	0	0	0	0	0	0	0	0	0	0	0	0	0	0	0	0	0	0	0	0
0	4	0	0	0	0	2	0	0	0	1	0	0	3	0	0	0	0	0	0	0	0	0
0	0	0	0	0	0	0	0	0	0	0	1	0	0	0	0	0	0	0	0	0	5	0
0	0	0	0	0	0	0	0	0	0	0	0	0	0	0	0	0	0	0	0	0	0	0
0	10	0	0	0	0	0	0	0	0	0	0	0	0	0	0	0	15	1	0	0	0	0
0	0	0	0	0	0	0	0	0	0	0	0	0	0	0	0	0	0	0	0	0	0	0
0	0	0	0	0	0	0	0	0	0	0	0	0	0	0	0	0	0	0	0	0	0	0
0	0	0	0	0	0	0	0	0	0	0	0	0	0	0	0	0	0	0	0	0	0	0
5442	4420	2241	2007	7863	3713	2681	1334	2009	2388	1405	3189	4367	11491	6110	9492	6646	2938	7711	13710	3361	7903	3337
302	993	603	1375	819	528	7654	677	256	406	1379	502	517	381	189	339	434	1555	335	1114	738	580	590
0	58	55	238	0	126	4	1	24	98	237	50	55	48	0	0	24	55	15	27	34	4	36
96	485	573	0	430	211	47	206	103	153	96	248	487	70	104	197	325	118	68	59	233	249	29

E2.013	E2.014	E2.016	E2.017	E2.018	E2.019	E2.020	E2.021	E2.024	E2.025	E2.026	E2.027	E2.029	E2.030	E2.031	E2.032	E2.033	E2.034	E2.036	E2.037	E2.038	E3.006	E3.009
Esophag	Esophag	Esophag	Esophag	Esophag	Esophag	Esophag	Esophag	Esophag	Esophag	Esophag	Esophag	Esophag	Esophag	Esophag	Esophag	Esophag	Esophag	Esophag	Esophag	Esophag	Esophag	Esophag
ME	ME	ME	ME	ME	ME	ME	ME	ME	ME	ME	ME	ME	ME	ME	ME	ME	ME	ME	ME	ME	LE	LE
0	0	0	0	0	0	0	0	0	0	0	0	0	0	0	0	0	0	0	0	0	0	0
0	0	0	0	0	0	0	0	2	55	3	23	2	3	0	0	0	0	2	0	47	37	4
0	0	0	0	0	0	0	0	0	0	0	0	0	0	0	0	0	0	0	0	0	0	0
0	0	0	0	0	0	0	0	0	0	0	0	0	0	0	0	0	0	0	0	0	0	0
0	0	0	0	0	0	0	0	0	0	0	0	0	0	0	0	0	0	0	0	0	0	0
0	0	0	0	0	0	0	0	0	0	0	0	0	0	0	0	0	0	0	0	0	0	0
124	722	673	313	408	873	48	69	6819	4733	696	10112	486	540	520	2726	300	1499	320	38	1437	151	248
105	1259	676	431	1191	1561	90	930	636	1008	636	605	989	1222	264	1963	800	571	410	150	1902	1511	514
4	0	1	25	23	32	3	0	3	4	7	8	2	1	0	0	2	0	4	1	31	0	7
36	20	9	137	189	44	328	476	37	85	180	101	112	168	33	51	191	0	4	137	99	5004	37
275	3468	3521	1759	2427	4194	377	4998	1331	2564	1170	2194	2670	13190	514	12253	2455	820	15308	278	9226	2117	1009
0	0	0	0	0	0	0	0	0	0	0	0	0	0	0	0	0	0	0	0	0	4	0
0	0	0	0	0	0	0	0	0	0	0	0	0	0	0	0	0	0	0	0	0	0	0
29	15	0	0	91	0	10	7	2	8	9	68	31	21	62	78	53	55	12	32	1	46	29
2	0	0	0	0	0	1	0	0	0	0	2	0	0	0	0	0	0	5	9	0	1	3
0	0	0	184	0	0	0	0	0	0	0	0	0	0	0	0	0	0	0	0	0	0	0
19	27	0	0	71	0	0	35	21	42	20	28	3	17	0	0	0	56	1	3	0	7	61
0	0	0	0	0	0	0	0	1	0	2	1	3	0	0	0	0	0	0	0	0	0	2
0	0	0	0	0	0	0	0	0	0	0	0	0	0	0	0	0	0	0	0	0	0	0
0	0	0	0	0	0	0	0	0	0	0	1	0	0	0	0	0	0	0	0	0	0	0
0	0	0	0	0	0	0	0	0	0	0	0	0	0	0	0	0	0	0	0	0	0	0
0	0	0	0	0	0	0	0	0	0	0	0	0	0	0	0	0	0	0	0	0	0	0
0	0	0	0	0	0	0	0	0	0	0	0	0	0	0	0	0	0	0	0	0	0	0
28	162	289	17	40	199	3	55	78	472	47	649	707	51	42	53	132	232	266	155	316	51	81
0	0	0	0	0	0	0	0	0	0	0	0	0	0	0	0	0	0	0	0	0	0	0
0	23	0	46	5	31	0	0	0	0	0	0	0	8	0	0	34	267	20	3	0	0	2
0	0	0	0	0	0	0	0	0	0	0	0	0	0	0	0	0	0	0	0	0	0	0
0	0	0	0	39	0	0	0	0	2	1	0	1	3	0	0	0	82	0	0	27	0	0
0	0	0	0	0	0	0	0	0	0	0	0	0	1	0	0	0	0	0	0	0	0	0
0	0	0	0	0	0	0	0	0	0	0	0	0	0	0	0	0	0	0	0	0	0	0
0	0	0	0	0	0	0	0	0	0	0	0	0	0	0	0	0	0	0	0	0	0	0
0	0	0	0	0	0	0	0	0	0	0	5	0	0	0	0	0	0	0	0	0	0	0
0	0	0	0	0	0	0	0	0	0	0	0	0	0	0	0	0	0	0	0	0	0	0
0	0	0	0	0	0	0	0	2	23	3	5	7	0	0	0	0	0	0	0	0	0	4
0	0	0	0	0	0	0	0	0	0	0	0	0	0	0	0	0	0	0	0	0	0	0
0	0	0	0	0	50	0	0	1	0	0	0	0	0	0	0	0	0	5	0	0	0	0
83	465	45	258	131	517	70	121	31	80	82	82	251	526	149	828	161	370	79	648	711	562	118

E2.013	E2.014	E2.016	E2.017	E2.018	E2.019	E2.020	E2.021	E2.024	E2.025	E2.026	E2.027	E2.029	E2.030	E2.031	E2.032	E2.033	E2.034	E2.036	E2.037	E2.038	E3.006	E3.009
Esophag	Esophag	Esophag	Esophag	Esophag	Esophag	Esophag	Esophag	Esophag	Esophag	Esophag	Esophag	Esophag	Esophag	Esophag	Esophag	Esophag	Esophag	Esophag	Esophag	Esophag	Esophag	Esophag
ME	ME	ME	ME	ME	ME	ME	ME	ME	ME	ME	ME	ME	ME	ME	ME	ME	ME	ME	ME	ME	LE	LE
3	0	0	24	0	0	0	2	72	55	288	104	294	0	0	0	0	0	0	11	0	0	34
0	0	0	0	0	0	0	0	0	0	0	0	0	0	0	0	0	0	0	0	0	2	0
1	1	0	0	1	0	9	15	4	27	25	60	26	2	0	0	0	0	2	3	0	0	7
0	0	0	0	0	0	0	0	0	0	0	0	0	0	0	0	0	0	0	0	0	0	0
0	0	0	0	0	0	0	0	0	0	0	0	0	0	0	0	0	0	0	0	0	0	0
0	0	0	0	0	0	0	0	0	0	0	0	0	0	0	0	0	0	3	0	0	0	0
0	0	0	0	0	0	0	0	0	0	0	0	0	0	0	0	0	0	0	0	0	0	0
0	0	0	0	0	0	0	0	2	8	3	2	1	0	0	0	0	0	0	1	0	5	1
0	0	0	0	0	0	0	0	0	0	0	0	0	0	0	0	0	0	0	0	0	0	0
37	45	14	0	56	0	2	0	3	129	5	29	152	26	0	90	0	102	5	6	21	4	23
517	1927	8504	814	3855	1291	4188	2784	402	1785	827	1541	2694	1381	1413	2682	1086	2464	1178	1934	1141	1481	577
0	0	0	0	0	0	0	0	0	1	0	0	0	3	12	0	0	0	0	0	0	0	0
93	126	100	31	182	7	11	130	85	135	199	204	261	180	132	445	196	0	37	77	237	209	344
0	0	0	20	0	0	0	0	1	2	0	7	3	6	0	0	0	0	0	0	0	6	0
0	0	0	0	0	0	0	0	1	1	0	0	0	0	0	0	0	0	0	0	0	0	0
0	0	0	0	0	0	0	0	0	0	0	0	0	0	0	0	0	0	0	0	0	0	0
0	0	0	0	0	0	0	0	0	0	0	0	0	0	0	0	0	0	0	0	0	0	0
0	0	0	0	0	0	0	0	0	0	0	0	0	0	0	0	0	0	1	0	0	0	0
0	0	0	0	0	0	0	0	0	0	0	0	0	0	0	0	0	0	0	0	0	0	0
0	0	0	0	0	0	0	0	0	0	0	0	0	0	0	0	0	0	0	0	0	0	0
0	0	0	0	0	0	0	0	0	0	0	0	0	0	0	0	0	0	0	0	0	0	0
0	4	0	0	0	0	2	0	0	0	0	0	0	3	0	0	0	0	0	0	0	0	0
0	0	0	0	0	0	0	0	0	0	0	0	0	0	0	0	0	0	0	0	0	0	0
0	0	0	0	0	0	0	0	0	0	1	0	0	0	0	0	0	0	0	0	0	0	0
0	0	0	0	0	0	0	0	0	0	0	0	0	0	0	0	0	0	0	0	0	0	0
0	0	0	0	0	0	0	0	0	0	0	1	0	0	0	0	0	0	0	0	0	5	0
0	10	0	0	0	0	0	0	0	0	0	0	0	0	0	0	0	0	0	0	0	0	0
0	0	0	0	0	0	0	0	0	0	0	0	0	0	0	0	0	15	1	0	0	0	0
0	0	0	0	0	0	0	0	0	0	0	0	0	0	0	0	0	0	0	0	0	0	0
0	0	0	0	0	0	0	0	0	0	0	0	0	0	0	0	0	0	0	0	0	0	0
7	233	88	31	10	109	1	4	33	129	56	27	64	127	78	3644	0	0	39	0	75	21	57
89	180	224	31	402	428	313	192	319	368	40	144	1577	1935	373	841	419	275	3242	322	245	202	540
5346	4007	1929	1945	7451	3176	2367	1138	1657	1891	1308	3017	2726	9429	5659	4983	6227	2663	4426	13388	3041	7680	2737
0	0	0	0	0	0	0	0	0	0	1	1	0	0	0	24	0	0	4	0	0	0	3
302	993	603	1375	819	528	7654	677	256	406	1379	502	517	381	189	339	434	1555	335	1114	738	580	582
0	0	0	0	0	0	0	0	0	0	0	0	0	0	0	0	0	0	0	0	0	0	0
0	0	0	0	0	0	0	0	0	0	0	0	0	0	0	0	0	0	0	0	0	0	8

E2.013	E2.014	E2.016	E2.017	E2.018	E2.019	E2.020	E2.021	E2.024	E2.025	E2.026	E2.027	E2.029	E2.030	E2.031	E2.032	E2.033	E2.034	E2.036	E2.037	E2.038	E3.006	E3.009
Esophag	Esophag	Esophag	Esophag	Esophag	Esophag	Esophag	Esophag	Esophag	Esophag	Esophag	Esophag	Esophag	Esophag	Esophag	Esophag	Esophag	Esophag	Esophag	Esophag	Esophag	Esophag	Esophag
ME	ME	ME	ME	ME	ME	ME	ME	ME	ME	ME	ME	ME	ME	ME	ME	ME	ME	ME	ME	ME	LE	LE
0	58	55	238	0	126	4	1	24	98	237	50	55	48	0	0	24	55	15	27	34	4	36
96	485	573	0	430	211	47	206	103	153	96	248	487	70	104	197	325	118	68	59	233	249	29
0	0	0	0	0	0	0	0	0	0	0	0	0	0	0	0	0	0	0	0	0	0	0
0	0	0	0	0	0	0	0	0	0	0	0	0	0	0	0	0	0	0	0	0	0	0
0	0	0	0	0	0	0	0	0	0	0	0	0	0	0	0	0	0	0	0	0	0	0
0	0	0	0	0	0	0	0	0	0	0	0	0	0	0	0	0	0	0	0	0	0	0
0	0	0	0	0	0	0	0	0	0	0	0	0	0	0	0	0	0	0	0	0	0	0
0	0	0	0	0	0	0	0	0	0	0	0	0	2	0	0	0	0	0	0	0	0	0
57	454	299	52	292	542	21	11	457	173	94	322	204	294	133	632	84	375	179	17	587	50	114
0	0	0	0	0	0	0	0	0	0	0	0	0	0	0	0	0	0	0	0	0	0	0
0	0	0	0	0	0	0	0	0	0	1	0	0	0	0	0	0	0	0	0	0	0	0
56	194	272	137	79	208	7	23	192	262	54	224	81	108	224	1450	129	351	74	14	520	62	80
0	0	0	26	0	24	0	3	1	3	0	7	3	3	0	98	0	0	11	0	53	0	1
2	14	6	19	27	5	0	7	8	24	11	27	25	19	10	23	5	122	4	1	16	7	8
0	0	0	0	0	0	0	3	0	0	1	0	0	0	0	0	0	0	0	0	0	0	0
9	60	96	79	10	94	20	22	6161	4268	535	9531	173	111	153	523	82	651	52	6	246	32	45
0	0	0	0	0	0	0	0	0	0	0	0	0	0	0	0	0	0	0	0	0	0	0
33	282	61	276	40	61	43	54	117	323	75	114	108	91	94	499	177	95	51	16	184	21	216
0	0	0	36	1	0	0	3	0	1	2	0	0	0	15	0	0	0	0	0	0	0	0
0	0	0	0	0	0	0	0	0	0	0	0	0	0	0	0	0	0	0	0	0	0	0
72	975	599	119	1148	1500	42	872	519	679	558	488	874	1126	155	1460	616	476	359	134	1718	1490	294
0	2	16	0	2	0	5	1	0	4	1	3	5	3	0	4	7	0	0	0	0	0	4
0	0	0	0	0	0	0	0	0	0	0	0	0	0	0	0	0	0	0	0	0	0	0
0	0	0	0	0	0	0	0	0	0	0	0	0	0	0	0	0	0	0	0	0	0	0
0	0	0	0	0	0	0	0	1	0	1	1	0	1	0	0	0	0	0	0	0	0	0
0	0	0	0	0	0	3	0	0	0	0	2	0	0	0	0	0	0	0	0	0	0	2
0	0	1	0	23	32	0	0	1	4	6	1	0	0	0	0	0	0	4	1	0	0	5
0	0	0	0	0	0	0	0	0	0	0	0	0	0	0	0	0	0	0	0	0	0	0
1	0	0	0	0	0	0	0	0	0	0	3	0	0	0	0	0	0	0	0	31	0	0
0	0	0	0	0	0	0	0	0	0	0	0	0	0	0	0	0	0	0	0	0	0	0
3	0	0	25	0	0	0	0	1	0	0	1	2	0	0	0	0	0	0	0	0	0	0
0	0	0	0	0	0	0	0	0	0	0	0	0	0	0	0	0	0	0	0	0	0	0
0	0	0	0	0	0	0	0	0	0	0	0	0	0	0	0	2	0	0	0	0	0	0
36	20	9	137	189	44	328	476	37	85	180	101	112	168	33	51	191	0	4	137	99	5004	37
0	0	0	0	2	0	0	2	0	0	0	0	5	0	0	0	4	0	0	0	0	0	7
0	0	0	31	0	0	0	0	3	1	0	1	0	1	0	0	0	0	0	1	0	0	0
0	0	0	0	0	0	0	10	3	4	0	9	2	1	0	0	0	0	0	3	0	2	2

E2.013	E2.014	E2.016	E2.017	E2.018	E2.019	E2.020	E2.021	E2.024	E2.025	E2.026	E2.027	E2.029	E2.030	E2.031	E2.032	E2.033	E2.034	E2.036	E2.037	E2.038	E3.006	E3.009
Esophag	Esophag	Esophag	Esophag	Esophag	Esophag	Esophag	Esophag	Esophag	Esophag	Esophag	Esophag	Esophag	Esophag	Esophag	Esophag	Esophag	Esophag	Esophag	Esophag	Esophag	Esophag	Esophag
ME	ME	ME	ME	ME	ME	ME	ME	ME	ME	ME	ME	ME	ME	ME	ME	ME	ME	ME	ME	ME	LE	LE
35	1342	106	143	102	1389	14	34	405	196	612	627	233	341	108	7264	108	282	230	164	8036	47	209
0	18	36	7	25	6	0	0	0	0	0	0	0	7	0	0	0	0	2	0	0	0	0
0	0	0	75	0	0	0	0	0	0	0	0	2	0	0	0	0	0	0	0	0	0	0
216	1977	3207	1359	2120	2601	339	4943	620	2038	419	867	2178	12698	348	3592	2284	94	15010	107	514	1549	644
14	129	108	101	151	140	20	6	227	291	127	469	213	105	50	1241	53	328	48	2	554	502	86
0	0	0	0	0	0	0	0	0	0	0	0	0	0	0	5	0	0	0	0	0	0	0
0	0	0	0	0	0	0	0	0	0	0	0	0	0	0	0	0	0	0	0	0	0	0
10	2	64	43	27	58	4	3	73	34	12	221	37	37	8	151	6	116	18	1	122	17	61
0	0	0	0	0	0	0	0	0	0	0	0	0	0	0	0	0	0	0	0	0	4	0
0	0	0	0	0	0	0	0	0	0	0	0	0	0	0	0	0	0	0	0	0	0	0
0	0	0	0	0	0	0	0	0	0	0	0	0	0	0	0	0	0	0	1	0	0	0
29	15	0	0	91	0	10	7	2	8	9	68	31	21	62	78	53	55	12	31	1	46	29
2	0	0	0	0	0	1	0	0	0	0	2	0	0	0	0	0	0	5	9	0	1	3
0	0	0	0	0	0	0	0	0	6	0	7	0	16	0	0	0	56	0	1	0	0	57
19	0	0	0	43	0	0	35	20	32	15	9	3	1	0	0	0	0	1	2	0	7	2
0	27	0	0	28	0	0	0	1	4	5	12	0	0	0	0	0	0	0	0	0	0	2
0	0	0	0	0	0	0	0	1	0	2	1	3	0	0	0	0	0	0	0	0	0	2
0	0	0	0	0	0	0	0	0	0	0	0	0	0	0	0	0	0	0	0	0	0	0
0	0	0	0	0	0	0	0	0	0	0	0	0	0	0	0	0	0	0	0	0	0	0
11	0	241	0	6	23	3	37	1	154	2	17	6	0	0	0	13	65	29	1	19	10	17
0	0	0	0	0	0	0	0	0	0	0	0	0	4	0	53	0	0	0	0	0	0	1
0	0	0	0	0	0	0	4	0	21	3	81	360	5	0	0	17	0	0	4	0	0	4
0	0	0	0	0	0	0	0	0	0	0	0	0	0	0	0	0	0	0	0	0	0	0
0	23	0	46	5	31	0	0	0	0	0	0	0	8	0	0	34	267	20	3	0	0	2
0	0	0	0	0	0	0	0	0	0	0	0	0	0	0	0	0	0	0	0	0	0	0
0	0	0	0	39	0	0	0	0	2	1	0	1	3	0	0	0	82	0	0	27	0	0
0	0	0	0	0	0	0	0	0	0	0	0	0	0	0	0	0	0	0	0	0	0	0
0	0	0	0	0	0	0	0	0	0	0	0	0	1	0	0	0	0	0	0	0	0	0
0	0	0	0	0	0	0	0	0	0	0	0	0	0	0	0	0	0	0	0	0	0	0
0	0	0	0	0	0	0	0	0	0	0	0	0	0	0	0	0	0	0	0	0	0	0
0	0	0	0	0	0	0	0	0	0	0	5	0	0	0	0	0	0	0	0	0	0	0
0	0	0	0	0	0	0	0	0	0	0	0	0	0	0	0	0	0	0	0	0	0	0
0	0	0	0	0	0	0	0	0	0	0	0	0	0	0	0	0	0	0	0	0	0	0
0	0	0	0	0	0	0	0	0	0	0	0	0	0	0	0	0	0	0	0	0	0	0
30	140	17	14	0	188	19	89	5	37	7	42	84	59	18	29	35	21	26	316	110	108	83
0	0	0	0	0	0	0	0	0	0	0	0	0	0	0	0	0	0	0	0	0	0	0
0	0	0	0	24	0	0	0	0	0	0	0	0	0	0	0	0	0	0	0	0	0	0

E2.013	E2.014	E2.016	E2.017	E2.018	E2.019	E2.020	E2.021	E2.024	E2.025	E2.026	E2.027	E2.029	E2.030	E2.031	E2.032	E2.033	E2.034	E2.036	E2.037	E2.038	E3.006	E3.009
Esophag	Esophag	Esophag	Esophag	Esophag	Esophag	Esophag	Esophag	Esophag	Esophag	Esophag	Esophag	Esophag	Esophag	Esophag	Esophag	Esophag	Esophag	Esophag	Esophag	Esophag	Esophag	Esophag
ME	ME	ME	ME	ME	ME	ME	ME	ME	ME	ME	ME	ME	ME	ME	ME	ME	ME	ME	ME	ME	LE	LE
0	14	0	3	0	17	1	0	4	4	59	15	4	8	13	2	2	83	0	3	28	27	0
0	0	0	0	0	0	0	0	0	1	0	0	0	0	0	0	0	0	0	0	0	0	1
0	0	0	0	0	0	0	0	0	0	0	0	0	0	0	0	0	0	0	0	0	0	0
0	0	0	0	0	0	0	0	0	0	0	0	0	0	0	0	0	0	0	0	0	0	0
0	0	0	0	0	0	0	0	0	0	0	0	0	3	0	0	0	0	0	0	0	0	0
0	0	0	0	0	0	0	0	0	0	0	1	0	0	0	0	0	0	0	0	0	0	0
0	0	0	0	0	24	0	0	0	2	0	0	0	0	0	0	0	0	0	0	0	0	0
0	0	0	0	0	0	0	0	0	0	0	0	0	0	0	0	0	0	0	0	0	0	0
0	17	0	14	22	11	0	0	1	0	1	0	2	10	12	164	5	1	7	2	155	2	0
0	0	0	0	0	0	0	0	0	0	0	0	0	0	0	0	0	0	0	0	0	0	0
0	0	0	0	0	0	0	0	0	0	0	0	0	0	0	0	0	0	0	0	0	0	0
0	0	15	0	13	0	0	0	0	6	0	0	3	0	0	35	7	0	0	0	0	1	2
51	135	2	0	58	107	45	26	4	8	7	3	133	394	99	194	76	97	16	319	60	386	12
0	0	0	0	0	0	0	0	0	2	0	0	0	0	0	0	0	0	3	1	0	0	0
0	16	0	0	0	0	0	0	0	0	0	0	0	0	0	0	0	0	0	0	0	0	0
0	0	0	0	0	0	0	0	13	4	3	15	0	0	0	0	0	0	0	0	0	0	7
0	0	0	0	0	14	0	0	0	1	0	1	0	12	0	0	0	0	2	0	0	0	2
0	0	0	5	0	0	0	0	0	0	0	0	0	0	0	0	0	0	0	0	0	0	0
0	0	0	0	0	0	0	0	0	0	0	0	0	0	0	0	0	0	0	0	0	0	1
2	121	11	156	14	100	3	5	2	15	3	5	24	29	7	86	33	157	17	7	175	24	9
0	0	0	23	0	0	0	0	0	0	0	0	0	0	0	0	1	11	0	0	0	0	0
0	0	0	0	0	0	0	0	0	0	0	0	0	0	0	0	0	0	0	0	0	0	0
0	0	0	0	0	0	0	0	0	0	0	0	0	0	0	0	0	0	0	0	0	0	0
0	22	0	43	0	56	2	1	0	0	2	0	1	11	0	318	2	0	8	0	183	14	0
0	0	0	0	0	0	0	0	0	0	0	0	0	0	0	0	0	0	0	0	0	0	0
0	0	0	0	0	0	0	0	0	0	0	0	0	0	0	0	0	0	0	0	0	0	0
3	0	0	24	0	0	0	2	72	55	288	104	294	0	0	0	0	0	0	11	0	0	34
1	1	0	0	1	0	9	15	4	27	25	60	26	2	0	0	0	0	2	3	0	0	7
0	0	0	0	0	0	0	0	0	0	0	0	0	0	0	0	0	0	0	0	0	0	0
0	0	0	0	0	0	0	0	0	0	0	0	0	0	0	0	0	0	0	0	0	0	0
0	0	0	0	0	0	0	0	0	0	0	0	0	0	0	0	0	0	0	0	0	0	0
0	0	0	0	0	0	0	0	0	0	0	0	0	0	0	0	0	0	0	0	0	0	0
0	0	0	0	0	0	0	0	2	8	3	2	1	0	0	0	0	0	0	1	0	5	1
0	0	0	0	0	0	0	0	0	0	0	0	0	0	0	0	0	0	0	0	0	0	0
37	45	14	0	56	0	2	0	3	128	5	25	152	26	0	90	0	102	5	6	21	4	23
0	0	0	0	0	0	0	0	0	1	0	4	0	0	0	0	0	0	0	0	0	0	0
0	0	0	0	0	0	0	0	0	0	0	0	0	0	0	0	0	0	0	0	0	0	0

E2.013	E2.014	E2.016	E2.017	E2.018	E2.019	E2.020	E2.021	E2.024	E2.025	E2.026	E2.027	E2.029	E2.030	E2.031	E2.032	E2.033	E2.034	E2.036	E2.037	E2.038	E3.006	E3.009
Esophag	Esophag	Esophag	Esophag	Esophag	Esophag	Esophag	Esophag	Esophag	Esophag	Esophag	Esophag	Esophag	Esophag	Esophag	Esophag	Esophag	Esophag	Esophag	Esophag	Esophag	Esophag	Esophag
ME	ME	ME	ME	ME	ME	ME	ME	ME	ME	ME	ME	ME	ME	ME	ME	ME	ME	ME	ME	ME	LE	LE
0	0	0	29	0	0	0	0	3	1	0	0	0	0	0	0	0	0	0	0	0	0	6
127	325	6273	52	1542	222	289	644	191	807	149	863	712	365	616	1588	370	216	859	478	459	670	180
5	100	46	235	76	83	2	3	2	89	98	8	40	20	0	0	0	221	13	15	52	8	36
0	0	0	0	0	0	0	0	0	0	0	91	0	0	0	0	0	0	0	0	0	0	0
0	0	0	2	0	0	0	0	0	0	0	0	0	0	0	0	0	0	0	0	0	0	1
87	256	1537	17	1148	138	55	510	96	320	101	281	546	650	522	495	241	230	128	110	187	414	88
290	969	535	134	1079	557	3841	1624	108	520	289	189	1368	298	239	555	443	270	92	1308	418	354	242
5	0	0	0	0	0	0	0	0	0	0	0	0	0	0	0	0	0	0	0	0	0	0
0	69	33	0	0	0	0	0	0	0	3	1	0	5	0	0	0	0	11	0	0	0	5
2	200	79	345	10	290	0	0	2	41	179	23	12	38	36	44	22	1527	64	17	3	22	16
0	0	0	0	0	0	0	0	0	0	0	0	0	0	0	0	0	0	0	0	0	0	0
0	0	0	0	0	0	0	0	0	1	0	0	0	3	12	0	0	0	0	0	0	0	0
48	90	92	31	72	2	0	92	38	67	9	101	163	81	6	287	145	0	31	52	0	36	320
0	0	0	0	0	0	0	0	0	0	0	0	0	0	0	0	0	0	0	0	0	0	0
45	36	8	0	110	5	11	38	47	68	190	103	98	99	126	158	51	0	6	25	237	173	24
0	0	0	0	0	0	0	0	1	2	0	7	3	6	0	0	0	0	0	0	0	6	0
0	0	0	0	0	0	0	0	1	1	0	0	0	0	0	0	0	0	0	0	0	0	0
0	0	0	0	0	0	0	0	0	0	0	0	0	0	0	0	0	0	0	0	0	0	0
0	0	0	0	0	0	0	0	0	0	0	0	0	0	0	0	0	0	0	0	0	0	0
0	0	0	0	0	0	0	0	0	0	0	0	0	0	0	0	0	0	1	0	0	0	0
0	0	0	0	0	0	0	0	0	0	0	0	0	0	0	0	0	0	0	0	0	0	0
0	10	0	0	0	0	0	0	0	0	0	0	0	0	0	0	0	0	0	0	0	0	0
0	0	0	0	0	0	0	0	0	0	0	0	0	0	0	0	0	15	1	0	0	0	0
0	0	0	0	0	0	0	0	0	0	0	0	0	0	0	0	0	0	0	0	0	0	0
0	0	0	0	0	0	0	0	0	0	0	0	0	7	0	0	0	0	0	0	0	0	0
0	0	0	0	0	0	0	0	0	1	0	0	0	0	0	0	0	0	0	0	0	0	0
0	0	0	0	0	0	0	0	0	0	0	0	0	0	0	0	0	0	0	0	0	0	0
0	131	0	13	0	16	0	0	10	110	31	17	30	18	0	46	0	0	1	0	44	3	15
0	0	0	0	0	0	0	0	0	0	2	0	0	0	0	0	0	0	0	0	0	0	0
0	2	0	0	10	0	0	0	16	1	0	1	0	0	0	0	0	0	0	0	0	0	34
0	0	0	0	0	0	0	0	0	2	0	5	6	1	0	9	0	0	0	0	0	0	0
0	0	0	0	0	0	0	0	0	0	0	0	0	0	0	0	0	0	0	0	0	0	0
7	100	88	18	0	93	1	4	5	15	22	4	24	101	78	3587	0	0	38	0	31	18	7
0	0	0	0	0	0	0	0	0	0	0	0	0	0	0	0	0	0	0	0	0	0	0
89	180	224	31	402	428	313	192	319	359	40	143	1570	1935	373	841	419	275	3242	322	245	202	540
0	6	0	225	5	0	2	0	35	23	79	13	21	37	0	162	30	0	15	2	0	52	0
47	125	257	0	118	27	81	49	149	102	17	27	113	849	198	170	155	68	248	61	324	283	51

E2.013	E2.014	E2.016	E2.017	E2.018	E2.019	E2.020	E2.021	E2.024	E2.025	E2.026	E2.027	E2.029	E2.030	E2.031	E2.032	E2.033	E2.034	E2.036	E2.037	E2.038	E3.006	E3.009
Esophag	Esophag	Esophag	Esophag	Esophag	Esophag	Esophag	Esophag	Esophag	Esophag	Esophag	Esophag	Esophag	Esophag	Esophag	Esophag	Esophag	Esophag	Esophag	Esophag	Esophag	Esophag	Esophag
ME	ME	ME	ME	ME	ME	ME	ME	ME	ME	ME	ME	ME	ME	ME	ME	ME	ME	ME	ME	ME	LE	LE
0	16	0	51	0	0	0	0	10	4	14	19	0	5	0	0	0	0	0	0	0	0	31
130	64	0	1	0	18	0	1	220	575	312	1106	225	43	7	0	19	0	24	1198	0	0	26
0	8	0	0	0	0	0	0	0	1	20	0	4	0	0	0	0	0	0	0	0	0	0
5169	3788	1672	1668	7328	3131	2284	1088	1241	1183	866	1847	2351	8495	5454	4651	6023	2595	4139	12127	2717	7344	2629
0	0	0	0	0	0	0	0	0	0	1	1	0	0	0	24	0	0	4	0	0	0	3
0	0	0	0	0	0	0	0	0	0	0	0	0	0	0	0	0	0	0	0	0	0	0
71	120	0	0	62	19	16	41	16	26	14	22	25	32	13	46	125	79	30	45	57	23	50
8	0	0	0	48	46	0	11	31	45	30	73	24	1	0	0	0	0	8	6	0	29	5
0	0	0	66	22	0	0	0	0	0	15	0	1	0	0	0	0	254	1	1	0	0	4
0	8	0	57	0	1	0	0	4	16	55	8	23	0	0	0	0	0	0	0	44	0	6
0	0	0	0	0	0	0	0	0	0	0	1	0	0	0	0	0	0	0	0	0	0	0
1	0	0	0	0	0	0	0	0	2	0	0	0	0	0	0	0	0	0	1	0	0	3
59	206	70	843	62	129	905	129	74	123	717	110	114	81	0	21	102	427	88	54	108	310	180
0	0	0	0	0	0	0	0	5	0	4	1	1	14	0	0	16	0	0	1	0	8	0
7	46	0	1	154	0	5	50	43	25	134	74	42	25	14	50	33	0	1	41	0	54	18
6	280	53	261	32	220	0	2	17	44	269	19	34	27	0	0	15	545	49	12	0	1	180
0	0	0	0	0	0	0	0	0	0	0	0	0	0	0	0	0	0	0	0	0	0	0
85	208	444	58	260	5	6713	415	45	72	44	138	187	187	162	130	104	67	103	940	506	113	66
0	0	0	0	0	0	0	0	0	0	0	0	0	0	0	0	0	0	0	0	0	0	8
0	58	55	238	0	126	4	1	24	98	237	50	55	48	0	0	24	55	15	27	34	4	36
55	347	535	0	376	184	36	197	84	139	71	219	442	34	58	185	142	51	54	49	220	180	23
41	138	38	0	54	27	11	9	19	14	25	29	45	36	46	12	183	67	14	10	13	69	6
0	0	0	0	0	0	0	0	0	0	0	0	0	0	0	0	0	0	0	0	0	0	0
0	0	0	0	0	0	0	0	0	0	0	0	0	0	0	0	0	0	0	0	0	0	0
57	454	299	52	292	542	21	11	457	172	94	322	204	294	133	632	84	375	179	17	587	50	114
0	0	0	0	0	0	0	0	0	0	0	0	0	0	0	0	0	0	0	0	0	0	0
0	0	0	0	0	0	0	0	0	0	0	0	0	0	0	0	0	0	0	0	0	0	0
0	0	0	0	0	0	0	0	0	0	1	0	0	0	0	0	0	0	0	0	0	0	0
16	96	98	28	77	23	5	20	11	34	1	30	39	56	105	346	75	122	38	8	109	0	1
15	44	129	71	0	107	1	1	128	31	15	158	20	9	81	556	11	6	16	2	263	51	70
0	0	0	0	0	0	0	0	3	0	4	0	0	18	0	0	0	0	2	0	0	1	0
24	50	45	38	2	45	0	2	41	192	30	27	16	19	38	501	43	223	14	2	111	9	7
0	0	0	0	0	0	0	0	2	0	1	0	0	0	0	0	0	0	0	0	0	0	0
1	1	0	0	0	3	0	0	7	2	1	9	5	0	0	0	0	0	0	0	37	0	2
0	3	0	0	0	1	1	0	0	3	0	0	1	6	0	46	0	0	0	2	0	0	0
0	0	0	0	0	0	0	0	0	0	0	0	0	0	0	0	0	0	0	0	0	0	0
0	0	0	0	0	0	0	0	0	0	0	0	0	0	0	0	0	0	0	0	0	0	0

E2.013	E2.014	E2.016	E2.017	E2.018	E2.019	E2.020	E2.021	E2.024	E2.025	E2.026	E2.027	E2.029	E2.030	E2.031	E2.032	E2.033	E2.034	E2.036	E2.037	E2.038	E3.006	E3.009
Esophag	Esophag	Esophag	Esophag	Esophag	Esophag	Esophag	Esophag	Esophag	Esophag	Esophag	Esophag	Esophag	Esophag	Esophag	Esophag	Esophag	Esophag	Esophag	Esophag	Esophag	Esophag	Esophag
ME	ME	ME	ME	ME	ME	ME	ME	ME	ME	ME	ME	ME	ME	ME	ME	ME	ME	ME	ME	ME	LE	LE
0	0	0	26	0	24	0	3	1	3	0	7	3	3	0	98	0	0	11	0	53	0	1
2	2	6	19	0	0	0	0	2	4	3	8	6	3	10	23	5	56	4	1	16	0	4
0	12	0	0	27	5	0	7	6	20	8	19	19	16	0	0	0	66	0	0	0	7	4
0	0	0	0	0	0	0	0	0	0	0	0	0	0	0	0	0	0	0	0	0	0	0
0	0	0	0	0	0	0	0	0	0	0	0	0	0	0	0	0	0	0	0	0	0	0
0	0	0	0	0	0	0	3	0	0	0	0	0	0	0	0	0	0	0	0	0	0	0
0	1	16	2	0	0	0	1	0	4	1	1	0	0	0	0	0	0	0	0	0	0	0
9	59	80	77	10	94	20	21	6161	4263	534	9530	173	111	153	523	82	576	52	6	246	32	45
0	4	0	178	1	0	0	0	8	5	10	16	3	10	0	0	0	0	0	0	0	0	15
0	88	24	25	0	0	42	27	17	43	23	45	58	43	0	24	19	0	7	10	18	14	6
6	134	33	15	9	13	0	23	14	194	30	41	27	29	61	306	36	73	31	5	43	1	13
27	56	4	58	30	48	1	4	78	78	12	12	19	9	33	169	122	22	13	1	123	6	182
0	0	0	36	1	0	0	3	0	1	2	0	0	0	15	0	0	0	0	0	0	0	0
72	975	599	119	1148	1500	42	872	519	679	558	488	874	1126	155	1460	616	476	359	134	1718	1490	294
0	2	16	0	2	0	5	1	0	4	1	3	5	3	0	4	7	0	0	0	0	0	4
0	0	0	0	0	0	0	0	0	0	0	1	0	0	0	0	0	0	0	0	0	0	0
0	0	0	0	0	0	0	0	1	0	1	0	0	1	0	0	0	0	0	0	0	0	0
0	0	0	0	0	0	3	0	0	0	0	2	0	0	0	0	0	0	0	0	0	0	2
0	0	0	0	23	0	0	0	0	0	0	0	0	0	0	0	0	0	0	0	0	0	0
0	0	1	0	0	32	0	0	1	4	6	1	0	0	0	0	0	0	4	1	0	0	5
0	0	0	0	0	0	0	0	0	0	0	1	1	0	0	0	0	0	0	0	0	0	0
0	0	0	25	0	0	0	0	0	0	0	0	0	0	0	0	0	0	0	0	0	0	0
0	0	0	0	0	0	0	0	0	0	0	0	0	0	0	0	0	0	0	0	0	0	0
0	0	0	0	0	0	0	0	0	0	0	0	0	0	0	0	2	0	0	0	0	0	0
36	20	9	11	43	44	328	193	37	32	18	99	77	78	33	51	191	0	4	135	99	28	36
0	0	0	126	146	0	0	283	0	53	162	2	35	90	0	0	0	0	0	2	0	4976	1
0	0	0	0	2	0	0	2	0	0	0	0	0	0	0	0	4	0	0	0	0	0	0
0	0	0	0	0	0	0	0	0	0	0	0	5	0	0	0	0	0	0	0	0	0	7
0	0	0	0	0	0	0	0	1	0	0	0	0	0	0	0	0	0	0	0	0	0	0
0	0	0	31	0	0	0	0	0	1	0	0	0	1	0	0	0	0	0	0	0	0	0
0	0	0	0	0	0	0	0	2	0	0	1	0	0	0	0	0	0	0	1	0	0	0
0	0	0	0	0	0	0	9	2	3	0	9	2	1	0	0	0	0	0	3	0	2	2
35	1342	106	143	102	1389	14	34	405	196	612	627	233	341	108	7264	108	282	230	164	8036	47	209
0	0	0	0	0	0	0	0	0	0	0	0	0	0	0	0	0	0	0	0	0	0	0
0	18	36	7	25	6	0	0	0	0	0	0	0	7	0	0	0	0	2	0	0	0	0
0	0	0	75	0	0	0	0	0	0	0	0	0	0	0	0	0	0	0	0	0	0	0
0	0	0	0	0	0	0	0	0	0	0	0	2	0	0	0	0	0	0	0	0	0	0

E2.013	E2.014	E2.016	E2.017	E2.018	E2.019	E2.020	E2.021	E2.024	E2.025	E2.026	E2.027	E2.029	E2.030	E2.031	E2.032	E2.033	E2.034	E2.036	E2.037	E2.038	E3.006	E3.009
Esophag	Esophag	Esophag	Esophag	Esophag	Esophag	Esophag	Esophag	Esophag	Esophag	Esophag	Esophag	Esophag	Esophag	Esophag	Esophag	Esophag	Esophag	Esophag	Esophag	Esophag	Esophag	Esophag
ME	ME	ME	ME	ME	ME	ME	ME	ME	ME	ME	ME	ME	ME	ME	ME	ME	ME	ME	ME	ME	LE	LE
216	1977	3207	1359	2120	2601	339	4943	620	2038	419	867	2178	12698	348	3592	2284	94	15010	107	514	1549	644
6	125	33	101	130	130	15	1	25	57	22	110	60	73	11	1004	10	328	37	0	287	487	9
8	4	75	0	21	10	5	5	202	234	105	359	153	32	39	237	43	0	11	2	267	15	77
0	0	0	0	0	0	0	0	0	0	0	0	0	0	0	5	0	0	0	0	0	0	0
0	0	0	0	0	0	0	0	0	0	0	0	0	0	0	0	0	0	0	0	0	0	0
0	0	0	0	0	0	0	0	0	0	0	0	0	0	0	0	0	0	0	0	0	0	0
0	0	0	0	0	24	0	1	5	3	2	6	3	0	0	0	0	0	0	0	0	0	1
10	2	64	43	27	34	4	2	68	31	10	215	34	37	8	151	6	116	18	1	122	17	60
0	0	0	0	0	0	0	0	0	0	0	0	0	0	0	0	0	0	0	1	0	0	0
29	15	0	0	91	0	10	7	2	8	9	68	31	21	62	78	53	55	12	31	1	46	29
2	0	0	0	0	0	1	0	0	0	0	2	0	0	0	0	0	0	5	9	0	1	3
0	0	0	0	0	0	0	0	0	0	0	0	0	0	0	0	0	0	0	0	0	0	0
0	0	0	0	0	0	0	0	0	6	0	7	0	16	0	0	0	56	0	1	0	0	57
19	0	0	0	43	0	0	35	20	32	15	9	3	1	0	0	0	0	1	2	0	7	2
0	0	0	0	0	0	0	0	0	0	0	0	0	0	0	0	0	0	0	0	0	0	0
11	0	0	0	6	0	3	15	1	0	0	1	1	0	0	0	13	65	0	0	0	0	0
0	0	0	0	0	0	0	4	0	21	3	81	360	5	0	0	17	0	0	4	0	0	4
0	0	0	0	0	0	0	0	0	0	0	0	0	0	0	0	0	0	0	0	0	0	0
0	23	0	46	5	31	0	0	0	0	0	0	0	8	0	0	34	267	20	3	0	0	2
0	0	0	0	39	0	0	0	0	2	1	0	1	3	0	0	0	82	0	0	27	0	0
0	0	0	0	0	0	0	0	0	0	0	0	0	0	0	0	0	0	0	0	0	0	0
0	0	0	0	0	0	0	0	0	0	0	0	0	1	0	0	0	0	0	0	0	0	0
0	0	0	0	0	0	0	0	0	0	0	0	0	0	0	0	0	0	0	0	0	0	0
0	0	0	0	0	0	0	0	0	0	0	0	0	0	0	0	0	0	0	0	0	0	0
0	0	0	0	0	0	0	0	0	0	0	5	0	0	0	0	0	0	0	0	0	0	0
0	0	0	0	0	0	0	0	0	0	0	0	0	0	0	0	0	0	0	0	0	0	0
0	0	0	0	0	0	0	0	0	0	0	0	0	0	0	0	0	0	0	0	0	0	0
0	0	0	0	0	0	0	0	0	0	0	0	0	0	0	0	0	0	0	0	0	0	0
30	140	17	14	0	188	17	89	5	36	7	40	84	59	18	29	35	21	26	293	110	106	83
0	0	0	0	0	0	0	0	0	1	0	0	0	0	0	0	0	0	0	0	0	0	0
0	0	0	0	0	0	0	0	0	0	0	0	0	0	0	0	0	0	0	0	0	0	0
0	0	0	0	0	0	0	0	0	0	0	0	0	0	0	0	0	0	0	0	0	0	0
0	0	0	0	0	0	2	0	0	0	0	2	0	0	0	0	0	0	0	23	0	2	0
0	0	0	0	0	0	0	0	0	0	0	0	0	0	0	0	0	0	0	0	0	0	0
0	0	0	0	24	0	0	0	0	0	0	0	0	0	0	0	0	0	0	0	0	0	0
0	14	0	3	0	17	1	0	4	4	59	15	4	8	13	2	2	83	0	3	28	27	0
0	0	0	0	0	0	0	0	0	1	0	0	0	0	0	0	0	0	0	0	0	0	1

E2.013	E2.014	E2.016	E2.017	E2.018	E2.019	E2.020	E2.021	E2.024	E2.025	E2.026	E2.027	E2.029	E2.030	E2.031	E2.032	E2.033	E2.034	E2.036	E2.037	E2.038	E3.006	E3.009
Esophag	Esophag	Esophag	Esophag	Esophag	Esophag	Esophag	Esophag	Esophag	Esophag	Esophag	Esophag	Esophag	Esophag	Esophag	Esophag	Esophag	Esophag	Esophag	Esophag	Esophag	Esophag	Esophag
ME	ME	ME	ME	ME	ME	ME	ME	ME	ME	ME	ME	ME	ME	ME	ME	ME	ME	ME	ME	ME	LE	LE
0	0	0	0	0	0	0	0	0	0	0	0	0	0	0	0	0	0	0	0	0	0	0
0	0	0	0	0	0	0	0	0	0	0	0	0	0	0	0	0	0	0	0	0	0	0
0	0	0	0	0	0	0	0	0	0	0	0	0	3	0	0	0	0	0	0	0	0	0
0	0	0	0	0	0	0	0	0	0	0	1	0	0	0	0	0	0	0	0	0	0	0
0	0	0	0	0	0	0	0	0	0	0	0	0	0	0	0	0	0	0	0	0	0	0
0	0	0	0	0	0	0	0	0	0	0	0	0	0	0	0	0	0	0	0	0	0	0
0	0	0	0	0	24	0	0	0	0	0	0	0	0	0	0	0	0	0	0	0	0	0
0	0	0	0	0	0	0	0	0	0	0	0	0	0	0	0	0	0	0	0	0	0	0
0	0	0	0	0	0	0	0	0	2	0	0	0	0	0	0	0	0	0	0	0	0	0
0	0	0	0	0	0	0	0	0	0	0	0	0	0	0	0	0	0	0	0	0	0	0
0	0	0	0	0	0	0	0	0	0	0	0	0	0	0	0	0	0	0	0	0	0	0
0	0	0	0	0	0	0	0	1	0	0	0	1	0	0	0	0	0	0	0	0	0	0
0	17	0	14	3	11	0	0	0	0	1	0	1	10	12	164	5	1	7	2	155	2	0
0	0	0	0	19	0	0	0	0	0	0	0	0	0	0	0	0	0	0	0	0	0	0
0	0	0	0	0	0	0	0	0	0	0	0	0	0	0	0	0	0	0	0	0	0	0
0	0	0	0	0	0	0	0	0	0	0	0	0	0	0	0	0	0	0	0	0	0	0
0	0	0	0	4	0	0	0	0	0	0	0	0	0	0	0	5	0	0	0	0	0	0
0	0	0	0	0	0	0	0	0	0	0	0	0	0	0	0	0	0	0	0	0	0	0
0	0	0	0	0	0	0	0	0	0	0	0	0	0	0	0	0	0	0	0	0	0	0
0	0	0	0	0	0	0	0	0	0	0	0	0	0	0	35	0	0	0	0	0	0	0
0	0	14	0	8	0	0	0	0	1	0	0	0	0	0	0	0	0	0	0	0	0	0
0	0	0	0	0	0	0	0	0	0	0	0	0	0	0	0	0	0	0	0	0	0	0
0	0	0	0	0	0	0	0	0	0	0	0	0	0	0	0	0	0	0	0	0	1	0
0	0	0	0	0	0	0	0	0	0	0	0	0	0	0	0	0	0	0	0	0	0	0
0	0	1	0	1	0	0	0	0	5	0	0	3	0	0	0	2	0	0	0	0	0	2
0	0	0	0	0	0	0	0	0	0	0	0	0	0	0	0	0	0	0	0	0	0	0
0	0	0	0	0	0	0	0	0	0	0	0	1	0	0	1	0	0	0	0	0	0	0
0	0	0	0	0	0	0	0	0	0	0	0	0	0	0	0	0	0	0	0	0	0	0
0	0	0	0	0	0	0	0	0	0	0	0	1	0	0	0	21	0	0	0	0	0	1
0	0	0	0	0	0	0	0	0	0	0	0	0	0	0	26	0	0	0	0	0	0	0
0	0	0	0	0	0	0	0	0	0	0	0	2	0	0	0	0	0	0	0	0	1	0
51	135	2	0	58	107	45	26	4	8	6	3	129	394	99	165	55	97	15	319	40	385	11
0	0	0	0	0	0	0	0	0	0	0	0	0	0	0	2	0	0	0	0	0	0	0
0	0	0	0	0	0	0	0	0	0	1	0	0	0	0	0	0	0	1	0	20	0	0
0	0	0	0	0	0	0	0	0	2	0	0	0	0	0	0	0	0	3	1	0	0	0
0	16	0	0	0	0	0	0	0	0	0	0	0	0	0	0	0	0	0	0	0	0	0
0	0	0	0	0	0	0	0	0	0	0	0	0	0	0	0	0	0	0	0	0	0	0

E2.013	E2.014	E2.016	E2.017	E2.018	E2.019	E2.020	E2.021	E2.024	E2.025	E2.026	E2.027	E2.029	E2.030	E2.031	E2.032	E2.033	E2.034	E2.036	E2.037	E2.038	E3.006	E3.009
Esophag	Esophag	Esophag	Esophag	Esophag	Esophag	Esophag	Esophag	Esophag	Esophag	Esophag	Esophag	Esophag	Esophag	Esophag	Esophag	Esophag	Esophag	Esophag	Esophag	Esophag	Esophag	Esophag
ME	ME	ME	ME	ME	ME	ME	ME	ME	ME	ME	ME	ME	ME	ME	ME	ME	ME	ME	ME	ME	LE	LE
0	0	0	0	0	0	0	0	13	4	3	15	0	0	0	0	0	0	0	0	0	0	7
0	0	0	0	0	0	0	0	0	0	0	0	0	0	0	0	0	0	0	0	0	0	0
0	0	0	0	0	0	0	0	0	0	0	0	0	0	0	0	0	0	0	0	0	0	0
0	0	0	0	0	0	0	0	0	0	0	0	0	0	0	0	0	0	0	0	0	0	1
0	0	0	0	0	0	0	0	0	0	0	0	0	0	0	0	0	0	0	0	0	0	0
0	0	0	0	0	0	0	0	0	0	0	0	0	3	0	0	0	0	2	0	0	0	0
0	0	0	0	0	0	0	0	0	1	0	0	0	0	0	0	0	0	0	0	0	0	0
0	0	0	0	0	0	0	0	0	0	0	0	0	2	0	0	0	0	0	0	0	0	0
0	0	0	0	0	14	0	0	0	0	0	1	0	7	0	0	0	0	0	0	0	0	1
0	0	0	5	0	0	0	0	0	0	0	0	0	0	0	0	0	0	0	0	0	0	0
0	0	0	0	0	0	0	0	0	0	0	0	0	0	0	0	0	0	0	0	0	0	0
0	0	0	0	0	0	0	0	0	0	0	0	0	0	0	0	0	0	0	0	0	0	0
0	0	0	0	0	0	0	0	0	0	0	0	0	0	0	0	0	0	0	0	0	0	1
0	0	0	0	0	0	0	0	0	0	0	0	0	0	0	0	0	0	0	0	0	0	0
2	121	11	156	14	99	0	1	1	14	3	1	24	29	7	86	0	101	17	2	175	0	2
0	0	0	0	0	0	0	0	0	0	0	0	0	0	0	0	0	0	0	0	0	0	0
0	0	0	0	0	1	3	4	1	1	0	4	0	0	0	0	33	56	0	5	0	24	7
0	0	0	16	0	0	0	0	0	0	0	0	0	0	0	0	1	0	0	0	0	0	0
0	0	0	0	0	0	0	0	0	0	0	0	0	0	0	0	0	11	0	0	0	0	0
0	0	0	7	0	0	0	0	0	0	0	0	0	0	0	0	0	0	0	0	0	0	0
0	0	0	0	0	0	0	0	0	0	0	0	0	0	0	0	0	0	0	0	0	0	0
0	0	0	0	0	0	0	0	0	0	0	0	0	0	0	0	0	0	0	0	0	0	0
0	0	0	0	0	0	0	0	0	0	0	0	0	0	0	0	0	0	0	0	0	0	0
0	22	0	43	0	56	2	1	0	0	2	0	1	11	0	318	2	0	8	0	183	14	0
0	0	0	0	0	0	0	0	0	0	0	0	0	0	0	0	0	0	0	0	0	0	0
0	0	0	0	0	0	0	0	0	0	0	0	0	0	0	0	0	0	0	0	0	0	0
0	0	0	0	0	0	0	0	0	0	0	0	0	0	0	0	0	0	0	0	0	0	0
1	0	0	24	0	0	0	0	7	13	6	4	9	0	0	0	0	0	0	5	0	0	34
0	0	0	0	0	0	0	0	0	0	0	0	0	0	0	0	0	0	0	0	0	0	0
2	0	0	0	0	0	0	2	0	1	0	0	2	0	0	0	0	0	0	0	0	0	0
0	0	0	0	0	0	0	0	0	2	1	2	7	0	0	0	0	0	0	0	0	0	0
0	0	0	0	0	0	0	0	65	39	281	98	276	0	0	0	0	0	0	6	0	0	0
0	0	0	0	0	0	0	0	0	0	0	0	0	0	0	0	0	0	0	0	0	0	0
1	1	0	0	1	0	9	15	0	8	3	3	3	2	0	0	0	0	1	2	0	0	3
0	0	0	0	0	0	0	0	0	0	0	0	1	0	0	0	0	0	0	0	0	0	1
0	0	0	0	0	0	0	0	0	0	1	0	0	0	0	0	0	0	0	0	0	0	1
0	0	0	0	0	0	0	0	0	0	0	0	0	0	0	0	0	0	0	0	0	0	0

E2.013	E2.014	E2.016	E2.017	E2.018	E2.019	E2.020	E2.021	E2.024	E2.025	E2.026	E2.027	E2.029	E2.030	E2.031	E2.032	E2.033	E2.034	E2.036	E2.037	E2.038	E3.006	E3.009
Esophag	Esophag	Esophag	Esophag	Esophag	Esophag	Esophag	Esophag	Esophag	Esophag	Esophag	Esophag	Esophag	Esophag	Esophag	Esophag	Esophag	Esophag	Esophag	Esophag	Esophag	Esophag	Esophag
ME	ME	ME	ME	ME	ME	ME	ME	ME	ME	ME	ME	ME	ME	ME	ME	ME	ME	ME	ME	ME	LE	LE
0	0	0	0	0	0	0	0	0	0	0	0	0	0	0	0	0	0	0	0	0	0	0
0	0	0	0	0	0	0	0	4	19	21	57	22	0	0	0	0	0	1	1	0	0	2
0	0	0	0	0	0	0	0	0	0	0	0	0	0	0	0	0	0	0	0	0	0	0
0	0	0	0	0	0	0	0	0	0	0	0	0	0	0	0	0	0	0	0	0	0	0
0	0	0	0	0	0	0	0	0	0	0	0	0	0	0	0	0	0	0	0	0	0	0
0	0	0	0	0	0	0	0	0	0	0	0	0	0	0	0	0	0	0	0	0	0	0
0	0	0	0	0	0	0	0	2	8	3	2	1	0	0	0	0	0	0	1	0	5	1
0	0	0	0	0	0	0	0	0	0	0	0	0	0	0	0	0	0	0	0	0	0	0
0	0	0	0	0	0	0	0	0	0	0	0	0	0	0	0	0	0	0	0	0	0	0
0	0	0	0	0	0	0	0	0	0	0	0	0	0	0	0	0	0	0	0	0	0	0
0	0	0	0	0	0	0	0	0	0	0	0	0	0	0	0	0	0	0	0	0	0	0
37	45	14	0	56	0	2	0	3	127	5	25	151	21	0	90	0	102	5	6	21	4	23
0	0	0	0	0	0	0	0	0	1	0	0	1	5	0	0	0	0	0	0	0	0	0
0	0	0	0	0	0	0	0	0	0	0	0	0	0	0	0	0	0	0	0	0	0	0
0	0	0	0	0	0	0	0	0	1	0	4	0	0	0	0	0	0	0	0	0	0	0
0	0	0	0	0	0	0	0	0	0	0	0	0	0	0	0	0	0	0	0	0	0	0
0	0	0	29	0	0	0	0	3	1	0	0	0	0	0	0	0	0	0	0	0	0	0
0	0	0	0	0	0	0	0	0	0	0	0	0	0	0	0	0	0	0	0	0	0	6
127	325	6271	52	1542	222	289	644	191	807	149	860	712	365	616	1588	370	216	859	477	459	670	179
0	0	2	0	0	0	0	0	0	0	0	0	0	0	0	0	0	0	0	0	0	0	0
0	0	0	0	0	0	0	0	0	0	0	0	0	0	0	0	0	0	0	0	0	0	1
0	0	0	0	0	0	0	0	0	0	0	3	0	0	0	0	0	0	0	1	0	0	0
5	100	46	235	76	83	2	3	2	89	98	8	40	20	0	0	0	221	13	15	52	8	36
0	0	0	0	0	0	0	0	0	0	0	91	0	0	0	0	0	0	0	0	0	0	0
0	0	0	2	0	0	0	0	0	0	0	0	0	0	0	0	0	0	0	0	0	0	1
0	0	0	0	0	0	0	0	2	0	0	0	2	0	0	0	0	0	0	0	0	0	1
0	0	0	0	0	0	8	2	0	2	0	36	0	0	11	0	0	0	1	3	0	0	0
1	27	0	13	0	0	0	0	0	5	9	3	0	0	0	0	0	0	0	0	0	0	2
84	223	1537	4	1136	129	41	504	93	311	86	185	544	645	511	495	215	230	125	104	187	395	83
2	6	0	0	12	9	6	4	1	2	6	57	0	5	0	0	26	0	2	3	0	19	2
0	0	0	0	0	0	0	0	0	0	0	0	0	0	0	0	0	0	0	0	0	0	0
290	969	535	134	1079	557	3841	1624	108	520	289	189	1368	298	239	555	443	270	92	1308	418	354	242
5	0	0	0	0	0	0	0	0	0	0	0	0	0	0	0	0	0	0	0	0	0	0
0	0	0	0	0	0	0	0	0	0	0	0	0	0	0	0	0	0	0	0	0	0	0
0	0	0	0	0	0	0	0	0	0	0	0	0	0	0	0	0	0	0	0	0	0	0
0	0	0	0	0	0	0	0	0	0	0	0	0	0	0	0	0	0	0	0	0	0	0
0	69	33	0	0	0	0	0	0	0	3	1	0	5	0	0	0	0	11	0	0	0	5

E2.013	E2.014	E2.016	E2.017	E2.018	E2.019	E2.020	E2.021	E2.024	E2.025	E2.026	E2.027	E2.029	E2.030	E2.031	E2.032	E2.033	E2.034	E2.036	E2.037	E2.038	E3.006	E3.009
Esophag	Esophag	Esophag	Esophag	Esophag	Esophag	Esophag	Esophag	Esophag	Esophag	Esophag	Esophag	Esophag	Esophag	Esophag	Esophag	Esophag	Esophag	Esophag	Esophag	Esophag	Esophag	Esophag
ME	ME	ME	ME	ME	ME	ME	ME	ME	ME	ME	ME	ME	ME	ME	ME	ME	ME	ME	ME	ME	LE	LE
2	200	79	345	10	290	0	0	2	41	179	23	12	38	36	44	22	1527	64	17	3	22	16
0	0	0	0	0	0	0	0	0	0	0	0	0	0	0	0	0	0	0	0	0	0	0
0	0	0	0	0	0	0	0	0	0	0	0	0	0	0	0	0	0	0	0	0	0	0
0	0	0	0	0	0	0	0	0	0	0	0	0	3	0	0	0	0	0	0	0	0	0
0	0	0	0	0	0	0	0	0	1	0	0	0	0	0	0	0	0	0	0	0	0	0
0	0	0	0	0	0	0	0	0	0	0	0	0	0	0	0	0	0	0	0	0	0	0
0	0	0	0	0	0	0	0	0	0	0	0	0	0	12	0	0	0	0	0	0	0	0
0	0	0	0	0	0	0	0	0	0	0	0	0	0	0	0	0	0	0	0	0	0	0
0	0	0	0	0	0	0	0	0	0	0	0	0	0	0	0	0	0	0	0	0	0	0
0	0	0	0	0	0	0	0	0	0	0	0	0	0	0	0	0	0	0	0	0	0	0
0	0	0	0	0	0	0	0	0	0	0	0	0	0	0	0	0	0	0	0	0	0	0
0	0	0	0	0	0	0	0	0	0	0	0	0	0	0	0	0	0	0	0	0	0	0
0	0	0	0	0	2	0	0	12	5	4	5	3	4	0	132	3	0	2	1	0	3	302
0	0	0	0	0	0	0	0	0	0	0	0	1	0	0	117	0	0	0	1	0	0	4
0	0	0	0	0	0	0	0	11	4	2	49	1	0	0	0	0	0	0	0	0	0	11
0	0	0	0	0	0	0	0	0	0	0	0	0	0	0	0	0	0	0	0	0	0	0
0	0	0	0	0	0	0	0	0	0	0	0	0	0	0	0	0	0	0	0	0	0	0
48	90	92	31	72	0	0	92	15	58	3	47	158	77	6	38	142	0	29	50	0	33	3
0	0	0	0	0	0	0	0	0	0	0	0	0	0	0	0	0	0	0	0	0	0	0
45	34	8	0	110	5	11	38	46	65	189	99	90	99	126	158	51	0	6	25	199	173	24
0	2	0	0	0	0	0	0	0	3	1	4	8	0	0	0	0	0	0	0	38	0	0
0	0	0	0	0	0	0	0	1	0	0	0	0	0	0	0	0	0	0	0	0	0	0
0	0	0	0	0	0	0	0	0	0	0	0	0	0	0	0	0	0	0	0	0	0	0
0	0	0	0	0	0	0	0	0	0	0	0	2	1	0	0	0	0	0	0	0	6	0
0	0	0	0	0	0	0	0	1	2	0	2	1	5	0	0	0	0	0	0	0	0	0
0	0	0	0	0	0	0	0	0	0	0	5	0	0	0	0	0	0	0	0	0	0	0
0	0	0	0	0	0	0	0	1	1	0	0	0	0	0	0	0	0	0	0	0	0	0
0	0	0	0	0	0	0	0	0	0	0	0	0	0	0	0	0	0	0	0	0	0	0
0	0	0	0	0	0	0	0	0	0	0	0	0	0	0	0	0	0	0	0	0	0	0
0	0	0	0	0	0	0	0	0	0	0	0	0	0	0	0	0	0	1	0	0	0	0
0	0	0	0	0	0	0	0	0	0	0	0	0	0	0	0	0	0	0	0	0	0	0
0	0	0	0	0	0	0	0	0	0	0	0	0	0	0	0	0	0	0	0	0	0	0
0	10	0	0	0	0	0	0	0	0	0	0	0	0	0	0	0	0	0	0	0	0	0
0	0	0	0	0	0	0	0	0	0	0	0	0	0	0	0	0	15	1	0	0	0	0
0	0	0	0	0	0	0	0	0	0	0	0	0	0	0	0	0	0	0	0	0	0	0
0	0	0	0	0	0	0	0	0	0	0	0	0	7	0	0	0	0	0	0	0	0	0
0	0	0	0	0	0	0	0	0	1	0	0	0	0	0	0	0	0	0	0	0	0	0

E2.013	E2.014	E2.016	E2.017	E2.018	E2.019	E2.020	E2.021	E2.024	E2.025	E2.026	E2.027	E2.029	E2.030	E2.031	E2.032	E2.033	E2.034	E2.036	E2.037	E2.038	E3.006	E3.009
Esophag	Esophag	Esophag	Esophag	Esophag	Esophag	Esophag	Esophag	Esophag	Esophag	Esophag	Esophag	Esophag	Esophag	Esophag	Esophag	Esophag	Esophag	Esophag	Esophag	Esophag	Esophag	Esophag
ME	ME	ME	ME	ME	ME	ME	ME	ME	ME	ME	ME	ME	ME	ME	ME	ME	ME	ME	ME	ME	LE	LE
0	0	0	0	0	0	0	0	0	0	0	0	0	0	0	0	0	0	0	0	0	0	0
0	115	0	0	0	0	0	0	0	0	0	0	0	0	0	0	0	0	0	0	0	0	0
0	14	0	0	0	0	0	0	10	110	31	17	30	13	0	46	0	0	0	0	23	3	15
0	0	0	13	0	16	0	0	0	0	0	0	0	0	0	0	0	0	1	0	0	0	0
0	0	0	0	0	0	0	0	0	0	0	0	0	0	0	0	0	0	0	0	0	0	0
0	0	0	0	0	0	0	0	0	0	0	0	0	0	0	0	0	0	0	0	0	0	0
0	2	0	0	0	0	0	0	0	0	0	0	0	5	0	0	0	0	0	0	21	0	0
0	0	0	0	0	0	0	0	0	0	2	0	0	0	0	0	0	0	0	0	0	0	0
0	0	0	0	10	0	0	0	0	0	0	0	0	0	0	0	0	0	0	0	0	0	0
0	2	0	0	0	0	0	0	16	1	0	1	0	0	0	0	0	0	0	0	0	0	34
0	0	0	0	0	0	0	0	0	0	0	0	0	0	0	0	0	0	0	0	0	0	0
0	0	0	0	0	0	0	0	0	0	0	0	0	0	0	0	0	0	0	0	0	0	0
0	0	0	0	0	0	0	0	0	0	0	0	0	0	0	0	0	0	0	0	0	0	0
0	0	0	0	0	0	0	0	0	0	0	0	0	0	0	0	0	0	0	0	0	0	0
0	0	0	0	0	0	0	0	0	2	0	5	6	1	0	9	0	0	0	0	0	0	0
0	0	0	0	0	0	0	0	0	0	0	0	0	0	0	0	0	0	0	0	0	0	0
0	0	0	0	0	0	0	0	0	0	2	0	0	0	0	0	0	0	0	0	0	0	0
0	0	12	0	0	0	0	0	0	0	0	0	0	0	0	0	0	0	0	0	0	0	0
0	0	0	0	0	0	0	0	0	0	0	0	0	0	0	0	0	0	0	0	0	0	0
7	100	76	18	0	93	1	4	5	15	20	4	24	101	78	3587	0	0	38	0	31	18	7
0	0	0	0	0	0	0	0	0	0	0	0	0	0	0	0	0	0	0	0	0	0	0
0	0	0	0	0	0	0	0	2	0	1	0	4	0	0	2	0	0	0	0	0	0	1
0	0	0	0	0	0	0	0	15	75	4	41	5	0	0	0	0	0	0	0	0	0	0
0	0	0	0	0	0	0	0	0	2	0	2	1	0	0	0	0	0	0	0	0	0	0
89	180	224	31	402	428	313	192	304	282	36	100	1564	1935	373	841	419	275	3242	322	245	202	540
0	0	0	0	0	0	0	0	0	2	0	0	1	0	0	0	0	0	0	0	0	0	0
0	1	0	0	5	0	2	0	2	3	2	1	3	27	0	0	30	0	15	1	0	46	0
0	0	0	217	0	0	0	0	33	19	73	12	13	5	0	162	0	0	0	0	0	0	0
0	0	0	0	0	0	0	0	0	0	0	0	0	0	0	0	0	0	0	0	0	0	0
0	0	0	8	0	0	0	0	0	0	0	0	0	5	0	0	0	0	0	1	0	6	0
0	0	0	0	0	0	0	0	0	0	2	0	0	0	0	0	0	0	0	0	0	0	0
0	0	0	0	0	0	0	0	0	0	0	0	0	0	0	0	0	0	0	0	0	0	0
0	0	0	0	0	0	0	0	0	0	0	0	0	0	0	0	0	0	0	0	0	0	0
0	5	0	0	0	0	0	0	0	1	2	0	5	0	0	0	0	0	0	0	0	0	0
0	0	0	0	0	0	0	0	0	0	0	0	0	7	0	0	0	0	0	0	0	0	0
0	0	0	0	0	0	0	0	0	0	0	0	0	0	0	0	0	0	0	0	0	0	0
47	125	257	0	118	27	81	49	149	102	17	26	113	842	198	170	155	68	248	61	324	283	51

E2.013	E2.014	E2.016	E2.017	E2.018	E2.019	E2.020	E2.021	E2.024	E2.025	E2.026	E2.027	E2.029	E2.030	E2.031	E2.032	E2.033	E2.034	E2.036	E2.037	E2.038	E3.006	E3.009
Esophag	Esophag	Esophag	Esophag	Esophag	Esophag	Esophag	Esophag	Esophag	Esophag	Esophag	Esophag	Esophag	Esophag	Esophag	Esophag	Esophag	Esophag	Esophag	Esophag	Esophag	Esophag	Esophag
ME	ME	ME	ME	ME	ME	ME	ME	ME	ME	ME	ME	ME	ME	ME	ME	ME	ME	ME	ME	ME	LE	LE
0	0	0	0	0	0	0	0	0	0	0	1	0	0	0	0	0	0	0	0	0	0	0
0	0	0	0	0	0	0	0	0	0	0	0	0	0	0	0	0	0	0	0	0	0	0
0	0	0	0	0	0	0	0	0	0	0	0	0	0	0	0	0	0	0	0	0	0	0
0	15	0	47	0	0	0	0	1	1	6	6	0	1	0	0	0	0	0	0	0	0	0
0	0	0	0	0	0	0	0	0	0	0	0	0	0	0	0	0	0	0	0	0	0	0
0	0	0	3	0	0	0	0	2	2	3	6	0	4	0	0	0	0	0	0	0	0	2
0	1	0	1	0	0	0	0	7	1	5	7	0	0	0	0	0	0	0	0	0	0	29
130	64	0	1	0	18	0	1	220	575	312	1106	225	43	7	0	19	0	24	1198	0	0	26
0	8	0	0	0	0	0	0	0	1	20	0	4	0	0	0	0	0	0	0	0	0	0
0	0	0	0	0	0	0	0	0	0	0	0	0	0	0	0	0	0	0	0	0	0	0
5169	3788	1672	1668	7328	3131	2284	1088	1241	1183	866	1847	2351	8495	5454	4651	6023	2595	4139	12127	2717	7344	2629
0	0	0	0	0	0	0	0	0	0	0	0	0	0	0	0	0	0	0	0	0	0	0
0	0	0	0	0	0	0	0	2	3	0	4	12	0	0	0	0	0	0	0	0	1	0
0	0	0	0	0	0	0	0	0	0	1	1	0	0	0	24	0	0	4	0	0	0	3
0	0	0	0	0	0	0	0	0	0	0	0	0	0	0	0	0	0	0	0	0	0	0
0	0	0	0	0	0	0	0	0	0	0	0	0	0	0	0	0	0	0	0	0	0	0
0	1	0	0	0	18	0	0	2	3	0	5	0	0	0	0	0	31	0	0	0	0	0
26	42	0	0	11	1	3	37	0	1	1	0	15	3	0	0	73	22	5	12	57	5	9
0	0	0	0	0	0	0	0	1	1	2	2	3	0	0	0	0	0	4	0	0	0	2
45	77	0	0	51	0	13	4	13	21	11	15	7	29	13	46	52	26	21	33	0	18	39
0	0	0	0	0	0	0	0	0	0	0	0	0	0	0	0	0	0	0	0	0	0	0
0	0	0	0	0	0	0	0	5	23	12	6	4	0	0	0	0	0	1	0	0	0	0
0	0	0	0	0	0	0	0	1	7	3	18	3	0	0	0	0	0	0	0	0	0	2
0	0	0	0	0	0	0	0	0	0	0	0	0	0	0	0	0	0	0	0	0	0	0
8	0	0	0	48	46	0	11	13	12	6	10	14	1	0	0	0	0	5	6	0	29	2
0	0	0	0	0	0	0	0	12	3	9	37	3	0	0	0	0	0	2	0	0	0	0
0	0	0	0	0	0	0	0	0	0	0	0	0	0	0	0	0	0	0	0	0	0	0
0	0	0	0	0	0	0	0	0	0	0	0	0	0	0	0	0	0	0	0	0	0	1
0	0	0	0	0	0	0	0	0	0	0	0	0	0	0	0	0	0	0	0	0	0	0
0	0	0	0	0	0	0	0	0	0	0	2	0	0	0	0	0	0	0	0	0	0	0
0	0	0	0	0	0	0	0	0	0	0	0	0	0	0	0	0	0	0	0	0	0	0
0	0	0	66	22	0	0	0	0	0	15	0	1	0	0	0	0	254	1	1	0	0	4
0	0	0	0	0	0	0	0	0	0	0	0	0	0	0	0	0	0	0	0	0	0	0
0	0	0	0	0	0	0	0	2	10	33	6	22	0	0	0	0	0	0	0	0	0	0
0	0	0	1	0	0	0	0	0	0	0	0	0	0	0	0	0	0	0	0	0	0	0
0	0	0	0	0	0	0	0	0	0	0	0	0	0	0	0	0	0	0	0	0	0	1
0	0	0	0	0	0	0	0	0	4	0	0	0	0	0	0	0	0	0	0	0	0	0

E2.013	E2.014	E2.016	E2.017	E2.018	E2.019	E2.020	E2.021	E2.024	E2.025	E2.026	E2.027	E2.029	E2.030	E2.031	E2.032	E2.033	E2.034	E2.036	E2.037	E2.038	E3.006	E3.009
Esophag	Esophag	Esophag	Esophag	Esophag	Esophag	Esophag	Esophag	Esophag	Esophag	Esophag	Esophag	Esophag	Esophag	Esophag	Esophag	Esophag	Esophag	Esophag	Esophag	Esophag	Esophag	Esophag
ME	ME	ME	ME	ME	ME	ME	ME	ME	ME	ME	ME	ME	ME	ME	ME	ME	ME	ME	ME	ME	LE	LE
0	8	0	56	0	1	0	0	2	2	22	2	1	0	0	0	0	0	0	0	44	0	5
0	0	0	0	0	0	0	0	0	0	0	1	0	0	0	0	0	0	0	0	0	0	0
0	0	0	0	0	0	0	0	0	0	0	0	0	0	0	0	0	0	0	0	0	0	0
0	0	0	0	0	0	0	0	0	0	0	0	0	0	0	0	0	0	0	0	0	0	0
1	0	0	0	0	0	0	0	0	2	0	0	0	0	0	0	0	0	0	1	0	0	3
0	0	0	6	0	0	0	0	0	1	14	0	0	0	0	0	0	0	5	1	0	0	6
0	0	0	37	0	0	0	0	0	0	4	1	0	0	0	0	0	0	0	0	0	0	2
0	17	19	203	0	90	0	0	11	26	193	16	23	11	0	0	0	88	8	0	0	0	24
0	0	0	0	0	0	0	36	12	5	0	1	3	2	0	0	0	0	0	10	0	5	2
26	13	0	0	10	0	5	3	10	7	1	5	7	7	0	0	0	0	0	2	0	7	6
0	34	31	46	0	2	0	0	2	10	54	2	3	0	0	0	0	100	20	0	0	14	9
0	0	0	19	0	0	0	0	4	9	213	3	8	0	0	0	0	0	3	0	0	0	8
0	0	0	0	0	0	0	0	0	0	0	0	0	0	0	0	0	0	0	0	0	0	0
0	0	0	0	0	0	0	0	0	0	3	0	0	0	0	0	0	138	0	0	0	0	6
0	22	0	0	0	0	0	0	0	0	18	4	6	0	0	0	0	0	1	0	0	0	4
0	0	0	0	0	0	0	0	0	0	1	0	0	0	0	0	0	0	0	0	0	0	0
22	0	0	0	0	1	831	62	7	1	0	3	21	6	0	20	0	0	0	15	0	50	9
0	0	0	67	0	0	0	0	0	2	23	0	5	0	0	0	0	0	2	0	0	0	24
0	0	0	0	11	0	10	0	4	6	51	4	7	0	0	0	0	0	0	4	0	0	5
0	6	0	39	0	2	0	8	1	12	19	6	5	0	0	1	0	0	8	0	0	1	5
11	114	20	426	41	34	59	20	23	44	123	65	26	55	0	0	102	101	41	22	108	233	70
0	0	0	0	0	0	0	0	0	0	0	0	0	0	0	0	0	0	0	0	0	0	0
0	0	0	0	0	0	0	0	5	0	3	1	1	14	0	0	16	0	0	1	0	8	0
0	0	0	0	0	0	0	0	0	0	0	0	0	0	0	0	0	0	0	0	0	0	0
0	0	0	0	0	0	0	0	0	0	1	0	0	0	0	0	0	0	0	0	0	0	0
7	10	0	0	109	0	4	16	19	6	2	3	7	3	0	0	0	0	1	9	0	20	8
0	36	0	0	34	0	0	34	18	16	131	25	34	18	14	50	33	0	0	32	0	23	10
0	0	0	1	11	0	1	0	6	3	1	46	1	4	0	0	0	0	0	0	0	11	0
0	0	0	0	0	0	0	0	0	0	0	0	0	0	0	0	0	0	0	0	0	0	1
0	0	0	0	0	0	0	0	0	0	0	0	0	0	0	0	0	0	0	0	0	0	0
0	31	0	0	0	0	0	2	1	11	25	8	7	0	0	0	0	0	0	0	0	1	63
0	111	0	158	1	74	0	0	4	6	70	7	16	17	0	0	0	403	33	5	0	0	14
0	2	0	0	0	70	0	0	0	9	9	1	3	0	0	0	0	0	14	5	0	0	20
0	0	0	0	0	0	0	0	0	0	0	0	0	0	0	0	0	0	0	0	0	0	0
6	136	53	103	31	76	0	0	12	18	165	3	8	10	0	0	15	142	2	2	0	0	82
0	0	0	0	0	0	0	0	0	0	0	0	0	0	0	0	0	0	0	0	0	0	0
0	0	0	0	0	0	0	0	0	0	0	0	0	0	0	0	0	0	0	0	0	0	0

E2.013	E2.014	E2.016	E2.017	E2.018	E2.019	E2.020	E2.021	E2.024	E2.025	E2.026	E2.027	E2.029	E2.030	E2.031	E2.032	E2.033	E2.034	E2.036	E2.037	E2.038	E3.006	E3.009
Esophag	Esophag	Esophag	Esophag	Esophag	Esophag	Esophag	Esophag	Esophag	Esophag	Esophag	Esophag	Esophag	Esophag	Esophag	Esophag	Esophag	Esophag	Esophag	Esophag	Esophag	Esophag	Esophag
ME	ME	ME	ME	ME	ME	ME	ME	ME	ME	ME	ME	ME	ME	ME	ME	ME	ME	ME	ME	ME	LE	LE
14	1	0	0	22	0	7	19	13	12	9	28	6	2	0	0	0	0	10	0	6	6	9
0	0	0	0	0	0	0	0	0	0	0	0	0	0	0	0	0	0	0	0	0	0	0
10	21	0	0	0	0	74	26	8	5	24	7	30	2	0	0	0	2	0	7	23	1	0
0	0	0	0	0	0	0	0	0	0	0	0	0	0	0	0	0	0	0	0	0	0	0
0	12	0	0	0	0	0	0	1	1	0	8	0	2	0	4	0	0	0	1	0	0	1
14	0	0	0	35	0	48	1	1	1	2	31	3	10	27	8	0	0	1	21	59	7	7
0	0	0	0	0	0	0	0	0	0	0	0	0	0	0	0	0	0	0	0	0	0	0
0	0	0	0	0	0	0	0	0	0	0	0	0	0	0	0	0	0	0	0	0	0	7
47	174	444	58	203	5	6581	369	22	53	9	63	148	171	127	118	104	65	92	911	418	99	39
0	0	0	0	0	0	0	0	0	0	0	0	0	0	0	0	0	0	0	0	0	0	0
0	0	0	0	0	0	3	0	0	0	0	1	0	0	8	0	0	0	0	0	0	0	3
0	29	0	34	20	9	0	0	1	8	7	2	2	2	0	0	0	0	8	1	0	2	6
0	0	0	0	0	0	0	0	0	0	0	0	0	0	0	0	0	0	0	0	0	0	8
0	6	0	0	0	0	0	0	1	2	0	0	2	0	0	0	0	0	0	0	0	0	4
0	25	55	238	0	120	0	0	3	63	227	32	21	9	0	0	7	55	8	0	34	0	15
0	27	0	0	0	5	4	1	19	30	4	12	30	28	0	0	17	0	6	25	0	4	10
0	0	0	0	0	0	0	0	0	0	0	0	0	0	0	0	0	0	0	0	0	0	5
0	0	0	0	0	0	0	0	0	0	0	0	0	0	0	0	0	0	0	0	0	0	0
0	0	0	0	0	0	0	0	0	0	0	0	0	0	0	0	0	0	0	0	0	0	0
0	0	0	0	0	0	0	0	0	0	0	0	0	0	0	0	0	0	0	0	0	0	1
0	0	0	0	0	0	0	0	0	0	0	0	0	0	0	0	0	0	0	0	0	0	0
0	0	0	0	0	0	0	0	1	0	0	6	0	0	0	0	0	0	0	2	0	0	0
0	0	0	0	0	0	0	0	0	0	0	0	0	0	0	0	0	0	0	0	0	0	0
0	0	0	0	0	1	0	0	0	3	6	0	2	11	0	0	0	0	1	0	0	0	1
0	0	0	0	0	0	0	0	0	0	0	0	0	0	0	0	0	0	0	0	0	0	0
55	347	535	0	376	184	36	197	84	139	71	219	442	34	58	185	142	51	54	49	220	180	23
28	138	38	0	54	27	11	9	17	8	13	28	34	33	13	12	65	67	13	10	13	67	6
0	0	0	0	0	0	0	0	2	1	12	1	11	0	0	0	0	0	0	0	0	0	0
0	0	0	0	0	0	0	0	0	0	0	0	0	0	0	0	0	0	0	0	0	0	0
13	0	0	0	0	0	0	0	0	5	0	0	0	3	33	0	118	0	1	0	0	2	0
0	0	0	0	0	0	0	0	0	0	0	0	0	0	0	0	0	0	0	0	0	0	0
0	0	0	0	0	0	0	0	0	0	0	0	0	0	0	0	0	0	0	0	0	0	0
0	0	0	0	0	0	0	0	0	0	0	0	0	0	0	0	0	0	0	0	0	0	0
0	0	0	0	0	0	0	0	0	1	0	0	0	0	0	0	0	0	0	0	0	0	0
0	0	0	0	0	0	0	0	10	3	11	3	32	16	0	77	0	0	0	0	55	0	7
0	0	0	0	0	0	0	0	11	5	1	17	1	0	0	0	0	0	0	0	7	0	4
0	0	0	0	0	0	0	0	1	0	4	0	1	6	0	0	0	0	0	0	0	0	2

E2.013	E2.014	E2.016	E2.017	E2.018	E2.019	E2.020	E2.021	E2.024	E2.025	E2.026	E2.027	E2.029	E2.030	E2.031	E2.032	E2.033	E2.034	E2.036	E2.037	E2.038	E3.006	E3.009
Esophag	Esophag	Esophag	Esophag	Esophag	Esophag	Esophag	Esophag	Esophag	Esophag	Esophag	Esophag	Esophag	Esophag	Esophag	Esophag	Esophag	Esophag	Esophag	Esophag	Esophag	Esophag	Esophag
ME	ME	ME	ME	ME	ME	ME	ME	ME	ME	ME	ME	ME	ME	ME	ME	ME	ME	ME	ME	ME	LE	LE
0	17	0	0	0	1	0	0	271	28	26	28	20	1	0	0	0	1	1	0	0	0	55
0	0	0	0	0	0	0	0	0	0	0	1	1	0	0	0	0	0	0	0	0	0	0
57	437	299	52	292	541	21	11	164	135	52	273	149	271	133	555	84	374	178	17	525	50	46
0	0	0	0	0	0	0	0	0	1	0	0	0	0	0	0	0	0	0	0	0	0	0
0	0	0	0	0	0	0	0	0	0	0	0	0	0	0	0	0	0	0	0	0	0	0
0	0	0	0	0	0	0	0	0	0	0	0	0	0	0	0	0	0	0	0	0	0	0
0	0	0	0	0	0	0	0	0	0	0	0	0	0	0	0	0	0	0	0	0	0	0
0	0	0	0	0	0	0	0	0	0	0	0	0	0	0	0	0	0	0	0	0	0	0
0	0	0	0	0	0	0	0	0	0	1	0	0	0	0	0	0	0	0	0	0	0	0
0	0	0	0	0	0	0	0	0	0	0	0	2	0	0	0	0	0	0	0	0	0	0
0	0	0	0	0	0	0	0	0	0	0	0	0	0	0	0	0	0	0	0	0	0	0
16	96	95	13	65	14	4	18	11	31	1	28	37	55	104	306	68	107	32	8	101	0	0
0	0	0	0	0	2	0	0	0	0	0	0	0	0	1	0	0	0	0	0	1	0	0
0	0	3	15	12	7	1	2	0	3	0	2	0	1	0	40	7	15	6	0	7	0	1
15	44	129	71	0	107	1	1	125	31	15	155	20	9	81	556	11	6	16	2	263	51	70
0	0	0	0	0	0	0	0	3	0	0	3	0	0	0	0	0	0	0	0	0	0	0
0	0	0	0	0	0	0	0	2	0	4	0	0	8	0	0	0	0	0	0	0	0	0
0	0	0	0	0	0	0	0	0	0	0	0	0	10	0	0	0	0	2	0	0	1	0
0	0	0	0	0	0	0	0	1	0	0	0	0	0	0	0	0	0	0	0	0	0	0
0	0	0	0	0	0	0	0	0	0	0	0	0	0	0	0	0	0	0	0	0	0	0
0	0	0	0	0	0	0	0	0	0	0	0	0	0	0	0	0	0	0	0	0	0	0
0	0	0	0	0	0	0	0	0	0	0	0	0	0	0	0	0	0	0	0	0	0	0
24	44	45	38	2	0	0	2	11	77	19	16	9	19	32	417	25	223	6	2	99	6	4
0	6	0	0	0	45	0	0	30	115	11	11	7	0	6	84	18	0	8	0	12	3	3
0	0	0	0	0	0	0	0	2	0	0	0	0	0	0	0	0	0	0	0	0	0	0
0	0	0	0	0	0	0	0	0	0	1	0	0	0	0	0	0	0	0	0	0	0	0
0	0	0	0	0	0	0	0	0	0	0	0	0	0	0	0	0	0	0	0	0	0	0
0	0	0	0	0	0	0	0	0	0	0	0	0	0	0	0	0	0	0	0	0	0	0
0	0	0	0	0	0	0	0	0	0	0	1	0	0	0	0	0	0	0	0	0	0	0
1	1	0	0	0	0	0	0	7	2	1	8	5	0	0	0	0	0	0	0	37	0	2
0	0	0	0	0	3	0	0	0	0	0	0	0	0	0	0	0	0	0	0	0	0	0
0	0	0	0	0	1	1	0	0	2	0	0	1	3	0	0	0	0	0	2	0	0	0
0	0	0	0	0	0	0	0	0	0	0	0	0	0	0	46	0	0	0	0	0	0	0
0	0	0	0	0	0	0	0	0	0	0	0	0	3	0	0	0	0	0	0	0	0	0
0	3	0	0	0	0	0	0	0	1	0	0	0	0	0	0	0	0	0	0	0	0	0
0	0	0	0	0	0	0	0	0	0	0	0	0	0	0	0	0	0	0	0	0	0	0
0	0	0	0	0	0	0	0	0	0	0	0	0	0	0	0	0	0	0	0	0	0	0

E2.013	E2.014	E2.016	E2.017	E2.018	E2.019	E2.020	E2.021	E2.024	E2.025	E2.026	E2.027	E2.029	E2.030	E2.031	E2.032	E2.033	E2.034	E2.036	E2.037	E2.038	E3.006	E3.009
Esophag	Esophag	Esophag	Esophag	Esophag	Esophag	Esophag	Esophag	Esophag	Esophag	Esophag	Esophag	Esophag	Esophag	Esophag	Esophag	Esophag	Esophag	Esophag	Esophag	Esophag	Esophag	Esophag
ME	ME	ME	ME	ME	ME	ME	ME	ME	ME	ME	ME	ME	ME	ME	ME	ME	ME	ME	ME	ME	LE	LE
0	0	0	0	0	0	0	0	0	0	0	0	0	0	0	0	0	0	0	0	0	0	0
0	0	0	0	0	0	0	0	0	0	0	0	0	0	0	0	0	0	0	0	0	0	0
0	0	0	0	0	0	0	0	0	0	0	0	0	0	0	0	0	0	0	0	0	0	0
0	0	0	0	0	0	0	0	0	0	0	0	0	0	0	0	0	0	0	0	0	0	0
0	0	0	0	0	0	0	0	0	2	0	2	1	1	0	0	0	0	4	0	32	0	0
0	0	0	0	0	0	0	3	1	0	0	1	2	0	0	0	0	0	0	0	0	0	0
0	0	0	26	0	0	0	0	0	0	0	2	0	0	0	0	0	0	0	0	0	0	1
0	0	0	0	0	0	0	0	0	0	0	0	0	0	0	0	0	0	0	0	0	0	0
0	0	0	0	0	24	0	0	0	1	0	2	0	2	0	98	0	0	7	0	21	0	0
0	0	0	0	0	0	0	0	2	0	1	0	0	0	0	0	0	0	0	0	0	0	2
0	0	0	0	0	0	0	0	0	0	0	3	0	0	0	0	0	0	0	0	0	0	0
0	0	0	0	0	0	0	0	0	0	0	0	0	0	0	0	0	0	2	0	0	0	0
0	0	0	0	0	0	0	0	0	0	0	0	0	0	0	0	0	0	2	0	0	0	0
0	0	0	0	0	0	0	0	0	1	0	2	5	0	0	0	0	0	0	0	0	0	0
2	2	6	19	0	0	0	0	0	3	2	3	1	3	10	23	5	56	0	1	16	0	2
0	0	0	0	0	0	0	0	0	0	0	0	0	0	0	0	0	0	0	0	0	0	0
0	0	0	0	0	5	0	0	0	0	1	0	0	0	0	0	0	0	0	0	0	0	0
0	0	0	0	0	0	0	0	0	0	0	0	0	3	0	0	0	0	0	0	0	0	0
0	0	0	0	0	0	0	0	0	0	0	0	0	0	0	0	0	0	0	0	0	0	0
0	12	0	0	27	0	0	7	6	20	7	19	19	13	0	0	0	66	0	0	0	7	4
0	0	0	0	0	0	0	0	0	0	0	0	0	0	0	0	0	0	0	0	0	0	0
0	0	0	0	0	0	0	0	0	0	0	0	0	0	0	0	0	0	0	0	0	0	0
0	0	0	0	0	0	0	3	0	0	0	0	0	0	0	0	0	0	0	0	0	0	0
0	0	0	0	0	0	0	0	0	0	0	0	0	0	0	0	0	0	0	0	0	0	0
0	1	16	2	0	0	0	1	0	4	1	1	0	0	0	0	0	0	0	0	0	0	0
0	0	0	0	0	0	0	0	1	7	0	0	0	4	0	0	0	0	0	0	0	0	0
0	0	0	0	0	0	0	0	0	0	0	0	0	0	0	0	0	0	0	0	0	0	0
1	12	23	8	0	10	5	0	270	2365	62	116	36	39	37	134	4	24	21	2	1	0	7
0	2	0	9	0	2	0	0	31	1082	40	53	20	3	0	0	20	82	0	0	0	1	31
8	41	57	44	10	82	15	20	5856	745	431	9354	113	61	116	290	58	469	31	4	245	30	7
0	0	0	0	0	0	0	0	1	6	0	0	0	4	0	0	0	0	0	0	0	1	0
0	0	0	0	0	0	0	0	1	53	1	3	1	0	0	0	0	0	0	0	0	0	0
0	4	0	16	0	0	0	1	1	5	0	4	3	0	0	99	0	1	0	0	0	0	0
0	0	0	0	0	0	0	0	0	0	0	0	0	0	0	0	0	0	0	0	0	0	0
0	0	0	0	0	0	0	0	0	0	0	0	0	0	0	0	0	0	0	0	0	0	0
0	4	0	0	1	0	0	0	0	0	0	0	1	0	0	0	0	0	0	0	0	0	2
0	0	0	178	0	0	0	0	1	3	6	5	2	10	0	0	0	0	0	0	0	0	9

E2.013	E2.014	E2.016	E2.017	E2.018	E2.019	E2.020	E2.021	E2.024	E2.025	E2.026	E2.027	E2.029	E2.030	E2.031	E2.032	E2.033	E2.034	E2.036	E2.037	E2.038	E3.006	E3.009
Esophag	Esophag	Esophag	Esophag	Esophag	Esophag	Esophag	Esophag	Esophag	Esophag	Esophag	Esophag	Esophag	Esophag	Esophag	Esophag	Esophag	Esophag	Esophag	Esophag	Esophag	Esophag	Esophag
ME	ME	ME	ME	ME	ME	ME	ME	ME	ME	ME	ME	ME	ME	ME	ME	ME	ME	ME	ME	ME	LE	LE
0	0	0	0	0	0	0	0	0	0	0	0	0	0	0	0	0	0	0	0	0	0	0
0	0	0	0	0	0	0	0	7	2	4	11	0	0	0	0	0	0	0	0	0	0	4
0	0	0	0	0	0	0	0	0	0	0	0	0	0	0	9	0	0	0	0	0	0	0
0	87	24	25	0	0	42	27	17	43	23	45	58	37	0	0	19	0	7	10	18	14	6
0	0	0	0	0	0	0	0	0	0	0	0	0	0	0	0	0	0	0	0	0	0	0
0	1	0	0	0	0	0	0	0	0	0	0	0	6	0	15	0	0	0	0	0	0	0
0	5	11	0	0	0	0	0	5	2	3	9	8	7	0	0	0	0	7	2	0	0	3
0	8	0	0	0	0	0	0	0	6	2	1	2	4	0	78	0	0	4	0	24	0	1
0	0	0	0	0	0	0	0	0	1	0	0	0	0	0	0	0	0	0	0	0	0	0
0	0	0	0	0	0	0	0	1	1	0	7	0	0	0	4	0	0	0	0	0	0	4
0	0	0	0	0	0	0	0	0	0	0	0	0	0	0	0	0	0	0	0	0	0	1
0	0	0	0	0	0	0	0	0	0	0	0	0	0	0	0	0	0	0	0	0	0	0
0	0	0	0	0	0	0	0	0	1	0	3	0	0	0	0	0	0	3	0	0	0	0
0	0	0	0	0	0	0	0	0	0	0	0	0	0	0	0	0	0	0	0	0	0	0
0	6	0	0	0	0	0	0	0	0	1	0	0	0	0	0	0	0	0	0	0	0	0
0	9	0	0	3	0	0	0	0	0	0	0	0	0	8	0	0	0	1	0	0	0	0
0	0	0	0	0	0	0	0	0	0	0	0	0	0	0	0	0	0	0	0	0	0	0
0	0	0	0	0	0	0	0	0	0	0	0	0	0	0	0	0	0	0	0	0	0	0
0	0	0	0	0	0	0	0	0	0	0	0	0	0	0	0	0	0	0	0	0	0	0
0	0	0	0	0	0	0	0	0	0	0	0	0	0	0	0	0	0	0	0	0	0	0
0	0	0	0	0	8	0	0	0	0	0	0	0	0	0	14	0	0	0	0	0	0	0
0	0	0	0	0	0	0	0	0	100	10	13	0	0	0	2	1	0	2	0	19	0	0
6	106	22	15	6	5	0	23	8	83	14	8	17	18	53	208	35	73	14	3	0	1	4
0	0	0	0	0	0	0	0	18	68	7	8	12	2	0	0	10	0	0	0	14	0	1
0	0	0	0	4	0	0	0	0	0	0	1	0	0	0	0	0	0	0	0	0	0	0
26	17	0	0	26	4	1	0	0	2	0	0	0	4	8	82	108	22	3	1	39	5	0
1	3	4	58	0	18	0	4	0	0	0	2	4	2	25	87	4	0	2	0	70	1	0
0	36	0	0	0	26	0	0	60	8	5	1	3	1	0	0	0	0	8	0	0	0	181
0	0	0	0	0	0	0	0	0	1	0	0	0	0	0	0	0	0	0	0	0	0	0
0	0	0	28	1	0	0	3	0	1	2	0	0	0	15	0	0	0	0	0	0	0	0
0	0	0	0	0	0	0	0	0	0	0	0	0	0	0	0	0	0	0	0	0	0	0
0	0	0	8	0	0	0	0	0	0	0	0	0	0	0	0	0	0	0	0	0	0	0
0	0	0	0	0	0	5	11	2	5	11	8	15	34	0	0	4	0	9	0	0	14	5
0	0	0	0	0	0	0	0	0	5	2	1	4	0	0	0	0	0	0	0	0	3	0
71	941	599	117	1148	1434	33	834	503	627	508	413	835	1010	136	1385	552	425	333	124	1622	1445	249
0	0	0	0	0	0	2	2	1	4	5	7	1	3	0	0	0	0	2	0	0	8	1
1	34	0	2	0	66	2	25	13	38	32	59	19	79	19	75	60	51	15	10	96	20	39

E2.013	E2.014	E2.016	E2.017	E2.018	E2.019	E2.020	E2.021	E2.024	E2.025	E2.026	E2.027	E2.029	E2.030	E2.031	E2.032	E2.033	E2.034	E2.036	E2.037	E2.038	E3.006	E3.009
Esophag	Esophag	Esophag	Esophag	Esophag	Esophag	Esophag	Esophag	Esophag	Esophag	Esophag	Esophag	Esophag	Esophag	Esophag	Esophag	Esophag	Esophag	Esophag	Esophag	Esophag	Esophag	Esophag
ME	ME	ME	ME	ME	ME	ME	ME	ME	ME	ME	ME	ME	ME	ME	ME	ME	ME	ME	ME	ME	LE	LE
0	0	0	0	0	0	0	0	0	1	0	0	0	0	0	0	0	0	0	0	0	0	0
0	0	0	0	0	0	0	0	0	0	0	0	0	0	0	4	0	0	0	0	0	0	0
0	0	16	0	2	0	0	0	0	3	0	3	3	0	0	0	0	0	0	0	0	0	0
0	0	0	0	0	0	0	0	0	0	0	0	0	0	0	0	0	0	0	0	0	0	0
0	0	0	0	0	0	0	0	0	0	0	0	1	0	0	0	0	0	0	0	0	0	4
0	2	0	0	0	0	5	1	0	0	1	0	1	3	0	0	7	0	0	0	0	0	0
0	0	0	0	0	0	0	0	0	1	0	0	2	0	0	0	0	0	0	0	0	0	0
0	0	0	0	0	0	0	0	0	0	0	1	0	0	0	0	0	0	0	0	0	0	0
0	0	0	0	0	0	0	0	1	0	1	0	0	1	0	0	0	0	0	0	0	0	0
0	0	0	0	0	0	3	0	0	0	0	2	0	0	0	0	0	0	0	0	0	0	2
0	0	0	0	23	0	0	0	0	0	0	0	0	0	0	0	0	0	0	0	0	0	0
0	0	0	0	0	0	0	0	0	0	0	0	0	0	0	0	0	0	0	0	0	0	2
0	0	1	0	0	0	0	0	1	4	4	0	0	0	0	0	0	0	4	0	0	0	1
0	0	0	0	0	32	0	0	0	0	2	1	0	0	0	0	0	0	0	1	0	0	2
0	0	0	0	0	0	0	0	0	0	0	1	1	0	0	0	0	0	0	0	0	0	0
0	0	0	25	0	0	0	0	0	0	0	0	0	0	0	0	0	0	0	0	0	0	0
0	0	0	0	0	0	0	0	0	0	0	0	0	0	0	0	0	0	0	0	0	0	0
0	0	0	0	0	0	0	0	0	0	0	0	0	0	0	0	0	0	0	0	0	0	0
0	0	0	0	0	0	0	0	0	0	0	0	0	0	0	0	2	0	0	0	0	0	0
0	0	0	0	0	0	0	0	0	0	0	0	0	0	0	0	0	0	0	0	0	0	0
36	20	9	11	43	44	328	193	37	32	18	99	77	78	33	51	191	0	4	135	99	28	36
0	0	0	0	0	0	0	0	0	1	1	2	2	0	0	0	0	0	0	0	0	0	1
0	0	0	126	146	0	0	283	0	52	161	0	33	90	0	0	0	0	0	2	0	4976	0
0	0	0	0	0	0	0	0	0	0	0	0	0	0	0	0	0	0	0	0	0	0	0
0	0	0	0	0	0	0	0	0	0	0	0	0	0	0	0	0	0	0	0	0	0	0
0	0	0	0	0	0	0	0	0	0	0	0	0	0	0	0	0	0	0	0	0	0	0
0	0	0	0	2	0	0	2	0	0	0	0	0	0	0	0	4	0	0	0	0	0	0
0	0	0	0	0	0	0	0	0	0	0	0	5	0	0	0	0	0	0	0	0	0	7
0	0	0	0	0	0	0	0	1	0	0	0	0	0	0	0	0	0	0	0	0	0	0
0	0	0	0	0	0	0	0	0	0	0	0	0	0	0	0	0	0	0	0	0	0	0
0	0	0	0	0	0	0	0	0	0	0	0	0	0	0	0	0	0	0	0	0	0	0
0	0	0	0	0	0	0	0	0	1	0	0	0	1	0	0	0	0	0	0	0	0	0
0	0	0	31	0	0	0	0	0	0	0	0	0	0	0	0	0	0	0	0	0	0	0
0	0	0	0	0	0	0	0	0	0	0	0	0	0	0	0	0	0	0	0	0	0	0
0	0	0	0	0	0	0	0	2	0	0	1	0	0	0	0	0	0	0	1	0	0	0
0	0	0	0	0	0	0	9	2	3	0	9	2	1	0	0	0	0	0	3	0	2	2
0	0	0	0	0	0	0	0	0	0	0	0	0	0	0	0	0	0	0	0	0	0	0

E2.013	E2.014	E2.016	E2.017	E2.018	E2.019	E2.020	E2.021	E2.024	E2.025	E2.026	E2.027	E2.029	E2.030	E2.031	E2.032	E2.033	E2.034	E2.036	E2.037	E2.038	E3.006	E3.009
Esophag	Esophag	Esophag	Esophag	Esophag	Esophag	Esophag	Esophag	Esophag	Esophag	Esophag	Esophag	Esophag	Esophag	Esophag	Esophag	Esophag	Esophag	Esophag	Esophag	Esophag	Esophag	Esophag
ME	ME	ME	ME	ME	ME	ME	ME	ME	ME	ME	ME	ME	ME	ME	ME	ME	ME	ME	ME	ME	LE	LE
0	0	0	0	0	0	0	1	1	1	0	0	0	0	0	0	0	0	0	0	0	0	0
0	0	0	0	0	0	0	0	0	0	0	0	0	0	0	0	0	0	0	0	0	0	0
5	0	0	0	0	0	0	0	1	2	2	1	3	0	13	0	0	0	0	1	0	0	0
0	0	0	0	0	6	0	0	0	2	1	0	3	0	0	0	0	0	0	1	0	0	1
0	0	0	0	0	0	0	0	0	0	0	0	0	0	0	0	0	0	0	0	0	0	0
0	0	0	0	0	0	0	0	0	0	0	0	0	0	0	0	0	0	0	0	0	0	0
0	0	0	0	0	0	0	0	0	0	0	0	0	0	0	0	0	0	0	0	0	0	0
0	0	0	31	0	0	0	0	0	0	0	2	0	0	0	0	0	0	0	0	0	0	0
0	0	0	0	0	0	0	0	0	0	0	0	0	0	0	0	0	0	0	0	0	0	0
0	0	0	0	0	0	0	0	0	0	0	0	1	0	0	0	0	0	0	0	0	0	0
0	13	0	0	10	12	0	0	0	0	1	8	0	0	11	7	0	0	0	3	4	0	0
30	1329	106	112	92	1371	14	34	404	192	608	616	226	341	84	7257	108	282	230	159	8032	47	208
0	0	0	0	0	0	0	0	0	0	0	0	0	0	0	0	0	0	0	0	0	0	0
0	18	36	7	25	6	0	0	0	0	0	0	0	7	0	0	0	0	2	0	0	0	0
0	0	0	0	0	0	0	0	0	0	0	0	0	0	0	0	0	0	0	0	0	0	0
0	0	0	0	0	0	0	0	0	0	0	0	0	0	0	0	0	0	0	0	0	0	0
0	0	0	75	0	0	0	0	0	0	0	0	0	0	0	0	0	0	0	0	0	0	0
0	0	0	0	0	0	0	0	0	0	0	0	2	0	0	0	0	0	0	0	0	0	0
95	1169	2257	1320	713	655	1	4398	46	1496	266	174	1835	10727	218	2856	3	0	14217	6	21	241	496
12	53	0	0	20	18	3	37	12	40	12	123	19	66	0	0	43	1	11	0	178	354	21
109	755	950	39	1387	1928	335	508	562	499	141	568	313	1905	130	736	2238	93	782	66	315	954	127
0	0	0	0	0	0	0	0	0	0	0	0	0	0	0	0	0	0	0	0	0	0	0
0	0	0	0	0	0	0	0	0	0	0	0	0	0	0	0	0	0	0	35	0	0	0
0	0	0	0	0	0	0	0	0	2	0	2	11	0	0	0	0	0	0	0	0	0	0
0	0	0	0	0	0	0	0	0	1	0	0	0	0	0	0	0	0	0	0	0	0	0
6	105	33	78	2	74	2	0	22	16	10	18	13	42	0	748	0	231	31	0	273	0	5
0	0	0	0	0	0	0	0	0	0	0	0	0	0	0	0	0	0	0	0	0	0	0
0	3	0	23	0	10	5	1	0	31	11	4	36	1	11	223	10	97	1	0	14	0	3
0	17	0	0	128	20	8	0	1	10	0	88	8	30	0	0	0	0	5	0	0	487	1
0	0	0	0	0	0	0	0	0	0	1	0	0	0	0	0	0	0	0	0	0	0	0
0	0	0	0	0	0	0	0	2	0	0	0	0	0	0	0	0	0	0	0	0	0	0
0	0	0	0	0	0	0	0	0	0	0	0	0	0	0	0	0	0	0	0	0	0	0
0	0	0	0	0	26	0	0	0	0	0	0	3	0	0	33	0	0	0	0	0	0	0
8	2	74	0	18	10	5	5	199	165	104	353	150	32	39	234	38	0	11	2	267	12	77
0	2	0	0	0	0	0	0	0	2	0	2	1	0	0	0	0	0	0	0	0	0	0
0	0	1	0	3	0	0	0	3	67	1	4	2	0	0	3	5	0	0	0	0	3	0
0	0	0	0	0	0	0	0	0	0	0	0	0	0	0	0	0	0	0	0	0	0	0

E2.013	E2.014	E2.016	E2.017	E2.018	E2.019	E2.020	E2.021	E2.024	E2.025	E2.026	E2.027	E2.029	E2.030	E2.031	E2.032	E2.033	E2.034	E2.036	E2.037	E2.038	E3.006	E3.009
Esophag	Esophag	Esophag	Esophag	Esophag	Esophag	Esophag	Esophag	Esophag	Esophag	Esophag	Esophag	Esophag	Esophag	Esophag	Esophag	Esophag	Esophag	Esophag	Esophag	Esophag	Esophag	Esophag
ME	ME	ME	ME	ME	ME	ME	ME	ME	ME	ME	ME	ME	ME	ME	ME	ME	ME	ME	ME	ME	LE	LE
0	0	0	0	0	0	0	0	0	0	0	0	0	0	0	5	0	0	0	0	0	0	0
0	0	0	0	0	0	0	0	0	0	0	0	0	0	0	0	0	0	0	0	0	0	0
0	0	0	0	0	0	0	0	0	0	0	0	0	0	0	0	0	0	0	0	0	0	0
0	0	0	0	0	0	0	0	0	1	0	0	0	0	0	0	0	0	0	0	0	0	0
0	0	0	0	0	24	0	0	0	1	0	1	2	0	0	0	0	0	0	0	0	0	0
0	0	0	0	0	0	0	1	5	1	2	5	1	0	0	0	0	0	0	0	0	0	1
0	0	0	0	0	0	0	0	0	0	0	0	0	0	0	0	0	0	0	0	0	0	0
0	0	0	0	2	4	0	0	2	1	0	1	0	0	0	4	0	66	0	0	21	0	0
0	0	0	0	0	0	0	0	0	0	1	1	0	0	0	0	0	0	0	0	0	0	1
0	0	0	0	12	0	0	0	0	1	0	0	3	0	0	0	0	0	0	0	0	0	0
0	0	0	0	0	0	0	0	0	0	0	0	0	0	0	0	0	0	0	0	0	0	0
10	2	64	43	2	30	4	2	64	25	7	210	14	37	8	146	6	49	10	1	101	16	57
0	0	0	0	0	0	0	0	0	0	0	0	0	0	0	0	0	0	0	0	0	0	0
0	0	0	0	0	0	0	0	0	3	2	2	13	0	0	0	0	0	0	0	0	0	0
0	0	0	0	11	0	0	0	2	1	0	1	4	0	0	1	0	1	8	0	0	1	2
0	0	0	0	0	0	0	0	0	0	0	0	0	0	0	0	0	0	0	0	0	0	0
0	0	0	0	0	0	0	0	0	0	0	0	0	0	0	0	0	0	0	0	0	0	0
0	0	0	0	0	0	0	0	0	0	0	0	0	0	0	0	0	0	0	1	0	0	0
29	15	0	0	91	0	10	7	2	8	9	68	31	21	62	78	53	55	12	31	1	46	29
0	0	0	0	0	0	0	0	0	0	0	0	0	0	0	0	0	0	5	0	0	0	0
2	0	0	0	0	0	1	0	0	0	0	2	0	0	0	0	0	0	0	9	0	1	3
0	0	0	0	0	0	0	0	0	0	0	0	0	0	0	0	0	0	0	0	0	0	0
0	0	0	0	0	0	0	0	0	0	0	0	0	0	0	0	0	0	0	0	0	0	0
0	0	0	0	0	0	0	0	0	6	0	7	0	16	0	0	0	56	0	1	0	0	57
19	0	0	0	43	0	0	35	20	30	13	5	3	1	0	0	0	0	1	2	0	7	2
0	0	0	0	0	0	0	0	0	2	2	4	0	0	0	0	0	0	0	0	0	0	0
0	0	0	0	0	0	0	0	0	0	0	0	0	0	0	0	0	0	0	0	0	0	0
0	0	0	0	0	0	0	0	0	0	0	0	0	0	0	0	0	0	0	0	0	0	0
11	0	0	0	6	0	3	15	1	0	0	1	1	0	0	0	13	65	0	0	0	0	0
0	0	0	0	0	0	0	4	0	21	3	81	360	5	0	0	17	0	0	4	0	0	4
0	0	0	0	0	0	0	0	0	0	0	0	0	0	0	0	0	0	0	0	0	0	0
0	23	0	46	5	31	0	0	0	0	0	0	0	8	0	0	34	267	20	3	0	0	2

E3.010	E3.012	E3.013	E3.016	E3.017	E3.018	E3.020	E3.021	E3.024	E3.025	E3.026	E3.027	E3.029	E3.030	E3.031	E3.032	E3.033	E3.034	E3.036	E3.037	E3.038
Esophag	Esophag	Esophag	Esophag	Esophag	Esophag	Esophag	Esophag	Esophag	Esophag	Esophag	Esophag	Esophag	Esophag	Esophag	Esophag	Esophag	Esophag	Esophag	Esophag	Esophagus
LE	LE	LE	LE	LE	LE	LE	LE	LE	LE	LE	LE	LE	LE	LE	LE	LE	LE	LE	LE	LE
0	0	0	0	0	0	0	0	0	0	0	0	0	0	0	0	0	0	0	0	0
6930	7422	10597	16674	17308	22949	10205	12234	11223	12007	21222	9374	10555	20084	23497	13948	13046	16351	27281	22916	10750
35	30	0	1	0	1	0	21	9	192	43	110	146	0	2	0	0	1	101	1	59
1	2	10	0	0	0	0	0	0	5	0	6	7	0	10	0	0	0	14	0	10
11	9	0	0	0	0	0	0	6	15	0	18	41	5	0	0	0	0	0	0	0
860	348	163	30	202	58	134	84	288	336	400	181	714	107	347	168	193	888	921	748	875
4	4	0	0	0	0	0	0	0	6	8	8	20	0	0	8	0	2	0	0	0
658	2109	561	4942	796	1824	2656	662	593	2195	913	899	1680	1324	3618	3215	2088	830	876	1276	2241
0	0	0	0	0	0	0	0	0	0	0	0	0	0	0	0	0	0	0	0	0
0	1	0	0	0	0	0	0	0	0	0	1	1	0	0	0	0	0	0	0	0
0	0	0	0	0	0	0	0	0	0	0	0	0	0	0	0	0	0	0	0	0
0	0	0	0	0	0	0	0	0	0	1	0	0	0	0	0	0	0	26	0	0
3	1	0	0	0	16	6	0	0	0	0	1	6	12	1	0	0	0	10	10	0
0	0	0	0	0	0	0	0	0	0	1	0	1	0	0	0	0	0	0	0	0
0	0	0	0	0	0	0	0	0	0	0	0	0	0	0	0	0	0	0	0	0
1286	1340	8952	2141	5392	14156	5759	1482	1446	2084	1426	2647	1891	8668	17113	3170	6627	5334	6140	19027	5637
47	41	155	134	35	358	106	86	152	102	262	69	140	169	36	208	245	247	75	147	89
0	0	0	0	0	0	0	0	0	0	0	0	0	0	0	0	0	0	0	0	0
6	5	0	0	0	0	12	0	12	29	3	9	2	13	2	37	0	0	0	0	6
1	0	0	0	0	0	0	0	0	0	0	0	0	0	0	0	0	0	0	0	0
0	0	0	0	0	0	0	0	0	0	0	0	0	0	0	0	0	0	0	0	0
3941	3408	697	9269	10863	6281	1507	9863	8545	6060	17988	5146	5756	9613	2156	6716	3740	8997	18839	1404	1675
3	1	0	0	0	75	25	0	12	14	20	22	1	26	74	6	68	13	3	28	23
2	1	1	95	0	15	0	0	7	4	1	9	4	10	1	261	0	0	0	0	0
0	0	0	0	0	12	0	0	1	0	3	1	0	4	6	5	0	0	0	4	0
27	39	0	0	0	0	0	0	13	17	21	15	13	0	29	1	19	0	0	14	0
0	0	0	0	0	0	0	0	0	0	0	0	2	0	0	0	0	0	0	0	10
0	0	0	0	0	0	0	0	0	5	0	0	3	0	0	0	0	0	0	0	0
44	83	58	62	20	153	0	36	137	943	132	227	127	133	100	153	66	39	276	257	63
0	0	0	0	0	0	0	0	0	0	0	0	0	0	2	0	0	0	0	0	62
1	0	0	0	0	0	0	0	2	0	0	0	0	0	0	0	0	0	0	0	0
0	0	0	0	0	0	0	0	0	0	0	5	0	0	0	0	0	0	0	0	0
1	2	10	0	0	0	0	0	0	5	0	6	7	0	10	0	0	0	14	0	10
0	0	0	0	0	0	0	0	0	0	0	0	0	0	0	0	0	0	0	0	0
0	0	0	0	0	0	0	0	0	0	0	0	0	5	0	0	0	0	0	0	0
0	0	0	0	0	0	0	0	0	0	0	1	3	0	0	0	0	0	0	0	0

E3.010	E3.012	E3.013	E3.016	E3.017	E3.018	E3.020	E3.021	E3.024	E3.025	E3.026	E3.027	E3.029	E3.030	E3.031	E3.032	E3.033	E3.034	E3.036	E3.037	E3.038
Esophag	Esophag	Esophag	Esophag	Esophag	Esophag	Esophag	Esophag	Esophag	Esophag	Esophag	Esophag	Esophag	Esophag	Esophag	Esophag	Esophag	Esophag	Esophag	Esophag	Esophagus
LE	LE	LE	LE	LE	LE	LE	LE	LE	LE	LE	LE	LE	LE	LE	LE	LE	LE	LE	LE	LE
0	0	0	0	0	0	0	0	0	0	0	0	0	0	0	0	0	0	0	0	0
11	9	0	0	0	0	0	0	6	15	0	17	38	0	0	0	0	0	0	0	0
0	0	0	0	0	0	0	0	0	0	0	0	0	0	0	0	0	0	0	0	0
0	0	0	0	0	0	0	0	0	1	0	0	0	0	0	0	0	0	0	0	0
792	332	163	30	201	58	134	84	277	307	379	166	697	107	346	168	193	887	903	735	784
68	16	0	0	1	0	0	0	11	28	21	15	17	0	1	0	0	1	0	13	91
0	0	0	0	0	0	0	0	0	0	0	0	0	0	0	0	0	0	0	0	0
0	0	0	0	0	0	0	0	0	0	0	0	0	0	0	0	0	0	0	0	0
0	0	0	0	0	0	0	0	0	0	0	0	0	0	0	0	0	0	18	0	0
4	4	0	0	0	0	0	0	0	6	8	8	20	0	0	8	0	2	0	0	0
0	0	0	0	0	0	0	0	0	0	0	0	0	0	0	0	0	0	0	0	0
54	19	50	14	27	19	19	6	5	90	21	45	832	32	13	8	0	24	9	0	9
576	2045	330	4520	744	1749	2631	581	442	1969	817	790	634	1089	3450	2876	2028	739	842	1198	2174
1	0	0	0	0	0	0	0	0	1	1	1	6	0	0	0	0	0	0	0	13
24	44	181	408	25	55	6	75	146	132	69	63	191	203	153	331	60	67	25	78	45
3	1	0	0	0	1	0	0	0	3	5	0	17	0	2	0	0	0	0	0	0
0	0	0	0	0	0	0	0	0	0	0	0	0	0	0	0	0	0	0	0	0
0	1	0	0	0	0	0	0	0	0	0	1	1	0	0	0	0	0	0	0	0
0	0	0	0	0	0	0	0	0	0	0	0	0	0	0	0	0	0	0	0	0
0	0	0	0	0	0	0	0	0	0	0	0	0	0	0	0	0	0	0	0	0
0	0	0	0	0	0	0	0	0	0	1	0	0	0	0	0	0	0	26	0	0
0	0	0	0	0	0	0	0	0	0	0	0	0	0	0	0	0	0	0	0	0
0	0	0	0	0	0	0	0	0	0	0	0	0	0	0	0	0	0	0	0	0
0	0	0	0	0	0	0	0	0	0	0	0	0	0	0	0	0	0	0	0	0
0	0	0	0	0	0	0	0	0	0	0	0	0	0	0	0	0	0	0	0	0
0	0	0	0	0	0	0	0	0	0	0	0	0	0	0	0	0	0	0	0	0
0	1	0	0	0	0	0	0	0	0	0	0	1	12	0	0	0	0	10	0	0
2	0	0	0	0	0	6	0	0	0	0	1	5	0	1	0	0	0	0	10	0
0	0	0	0	0	0	0	0	0	0	0	0	0	0	0	0	0	0	0	0	0
1	0	0	0	0	16	0	0	0	0	0	0	0	0	0	0	0	0	0	0	0
0	0	0	0	0	0	0	0	0	0	1	0	1	0	0	0	0	0	0	0	0
0	0	0	0	0	0	0	0	0	0	0	0	0	0	0	0	0	0	0	0	0
0	0	0	0	0	0	0	0	0	0	0	0	0	0	0	0	0	0	0	0	0
537	1054	8667	1733	5004	13247	2230	1099	1121	1504	865	1901	1486	8467	16409	2772	3782	4732	5838	18345	299
604	271	285	408	376	863	3479	365	278	537	540	694	382	201	695	386	2259	567	302	643	4490
145	15	0	0	11	46	50	18	47	42	21	52	23	0	9	12	586	35	0	39	848
47	41	155	134	35	358	106	86	152	102	262	69	140	169	36	208	245	247	75	147	89

E3.010	E3.012	E3.013	E3.016	E3.017	E3.018	E3.020	E3.021	E3.024	E3.025	E3.026	E3.027	E3.029	E3.030	E3.031	E3.032	E3.033	E3.034	E3.036	E3.037	E3.038
Esophag	Esophag	Esophag	Esophag	Esophag	Esophag	Esophag	Esophag	Esophag	Esophag	Esophag	Esophag	Esophag	Esophag	Esophag	Esophag	Esophag	Esophag	Esophag	Esophag	Esophagus
LE	LE	LE	LE	LE	LE	LE	LE	LE	LE	LE	LE	LE	LE	LE	LE	LE	LE	LE	LE	LE
0	0	0	0	0	0	0	0	0	0	0	0	0	0	0	0	0	0	0	0	0
6	5	0	0	0	0	12	0	12	29	3	9	2	13	2	37	0	0	0	0	6
1	0	0	0	0	0	0	0	0	0	0	0	0	0	0	0	0	0	0	0	0
0	0	0	0	0	0	0	0	0	0	0	0	0	0	0	0	0	0	0	0	0
0	0	0	0	0	0	0	0	0	0	0	0	0	0	0	0	0	0	0	0	0
0	0	0	0	0	0	0	0	0	0	0	0	0	0	0	0	0	0	0	0	0
1347	638	403	443	276	1033	424	5704	6914	1196	15364	3713	1582	497	345	205	428	2091	2059	206	294
485	1865	53	2269	816	1139	126	692	422	1533	772	295	706	1842	726	3367	853	1338	5890	339	740
9	3	0	0	0	0	11	0	1	12	0	10	7	0	6	18	96	0	0	4	29
4	49	0	0	4716	183	162	588	34	915	351	31	42	388	129	289	65	116	0	186	20
2096	853	241	6557	5055	3926	784	2879	1174	2404	1501	1097	3419	6886	950	2837	2298	5452	10890	669	592
0	0	0	0	0	0	0	0	0	0	0	0	0	0	0	0	0	0	0	0	0
0	0	0	0	0	0	0	0	0	0	0	0	0	0	0	0	0	0	0	0	0
3	1	0	0	0	75	25	0	12	14	20	22	1	26	74	6	68	13	3	28	23
0	0	0	0	0	12	0	0	1	0	3	1	0	4	6	5	0	0	0	4	0
0	0	0	0	0	0	0	0	0	0	0	0	0	0	0	0	0	0	0	0	0
26	39	0	0	0	0	0	0	13	17	21	15	13	0	22	1	19	0	0	14	0
1	0	0	0	0	0	0	0	0	0	0	0	0	0	7	0	0	0	0	0	0
0	0	0	0	0	0	0	0	0	0	0	0	2	0	0	0	0	0	0	0	10
0	0	0	0	0	0	0	0	0	5	0	0	0	0	0	0	0	0	0	0	0
0	0	0	0	0	0	0	0	0	0	0	0	3	0	0	0	0	0	0	0	0
0	0	0	0	0	0	0	0	0	0	0	0	0	0	0	0	0	0	0	0	0
0	0	0	0	0	0	0	0	0	0	0	0	0	0	0	0	0	0	0	0	0
44	83	58	62	20	153	0	36	137	943	132	227	127	133	100	153	66	39	276	257	63
0	0	0	0	0	0	0	0	0	0	0	0	0	0	0	0	0	0	0	0	0
0	0	0	0	0	0	0	0	0	0	0	0	0	0	2	0	0	0	0	0	62
0	0	0	0	0	0	0	0	0	0	0	5	0	0	0	0	0	0	0	0	0
1	2	0	0	0	0	0	0	0	4	0	5	7	0	10	0	0	0	14	0	10
0	0	10	0	0	0	0	0	0	1	0	1	0	0	0	0	0	0	0	0	0
0	0	0	0	0	0	0	0	0	0	0	0	0	0	0	0	0	0	0	0	0
0	0	0	0	0	0	0	0	0	0	0	0	0	5	0	0	0	0	0	0	0
0	0	0	0	0	0	0	0	0	0	0	1	3	0	0	0	0	0	0	0	0
0	0	0	0	0	0	0	0	0	0	0	0	0	0	0	0	0	0	0	0	0
11	9	0	0	0	0	0	0	6	15	0	17	38	0	0	0	0	0	0	0	0
0	0	0	0	0	0	0	0	0	0	0	0	0	0	0	0	0	0	0	0	0
0	0	0	0	0	0	0	0	0	1	0	0	0	0	0	0	0	0	0	0	0
66	153	162	30	201	58	134	84	50	156	54	59	393	107	342	168	193	887	903	728	246

E3.010	E3.012	E3.013	E3.016	E3.017	E3.018	E3.020	E3.021	E3.024	E3.025	E3.026	E3.027	E3.029	E3.030	E3.031	E3.032	E3.033	E3.034	E3.036	E3.037	E3.038
Esophag	Esophag	Esophag	Esophag	Esophag	Esophag	Esophag	Esophag	Esophag	Esophag	Esophag	Esophag	Esophag	Esophag	Esophag	Esophag	Esophag	Esophag	Esophag	Esophag	Esophagus
LE	LE	LE	LE	LE	LE	LE	LE	LE	LE	LE	LE	LE	LE	LE	LE	LE	LE	LE	LE	LE
726	179	1	0	0	0	0	0	227	151	325	107	304	0	4	0	0	0	0	7	538
0	0	0	0	0	0	0	0	0	0	0	0	0	0	0	0	0	0	0	0	0
68	16	0	0	1	0	0	0	11	28	21	15	17	0	1	0	0	1	0	13	91
0	0	0	0	0	0	0	0	0	0	0	0	0	0	0	0	0	0	0	0	0
0	0	0	0	0	0	0	0	0	0	0	0	0	0	0	0	0	0	0	0	0
0	0	0	0	0	0	0	0	0	0	0	0	0	0	0	0	0	0	0	0	0
0	0	0	0	0	0	0	0	0	0	0	0	0	0	0	0	0	0	18	0	0
4	4	0	0	0	0	0	0	0	6	8	8	20	0	0	8	0	2	0	0	0
0	0	0	0	0	0	0	0	0	0	0	0	0	0	0	0	0	0	0	0	0
54	19	50	14	27	19	19	6	5	90	21	45	832	32	13	8	0	24	9	0	9
576	2045	330	4520	744	1749	2631	581	442	1969	817	790	634	1089	3450	2876	2028	739	842	1198	2174
1	0	0	0	0	0	0	0	0	1	1	1	6	0	0	0	0	0	0	0	13
24	44	181	408	25	55	6	75	146	132	69	63	191	203	153	331	60	67	25	78	45
3	1	0	0	0	1	0	0	0	3	5	0	17	0	2	0	0	0	0	0	0
0	1	0	0	0	0	0	0	0	0	0	1	1	0	0	0	0	0	0	0	0
0	0	0	0	0	0	0	0	0	0	0	0	0	0	0	0	0	0	0	0	0
0	0	0	0	0	0	0	0	0	0	0	0	0	0	0	0	0	0	0	0	0
0	0	0	0	0	0	0	0	0	0	1	0	0	0	0	0	0	0	0	0	0
0	0	0	0	0	0	0	0	0	0	0	0	0	0	0	0	0	0	26	0	0
0	0	0	0	0	0	0	0	0	0	0	0	0	0	0	0	0	0	0	0	0
0	0	0	0	0	0	0	0	0	0	0	0	0	0	0	0	0	0	0	0	0
0	1	0	0	0	0	0	0	0	0	0	0	1	12	0	0	0	0	10	0	0
0	0	0	0	0	0	0	0	0	0	0	0	0	0	0	0	0	0	0	0	0
0	0	0	0	0	0	0	0	0	0	0	0	0	0	0	0	0	0	0	0	0
0	0	0	0	0	0	0	0	0	0	0	0	0	0	0	0	0	0	0	0	0
2	0	0	0	0	0	6	0	0	0	0	1	5	0	1	0	0	0	0	10	0
1	0	0	0	0	0	0	0	0	0	0	0	0	0	0	0	0	0	0	0	0
0	0	0	0	0	16	0	0	0	0	0	0	0	0	0	0	0	0	0	0	0
0	0	0	0	0	0	0	0	0	0	1	0	1	0	0	0	0	0	0	0	0
0	0	0	0	0	0	0	0	0	0	0	0	0	0	0	0	0	0	0	0	0
59	173	0	149	31	80	32	343	35	43	32	97	520	19	57	3	93	2891	227	3	123
18	124	123	90	145	275	335	91	131	183	118	127	213	1627	757	291	105	197	818	1965	0
460	757	8543	1494	4828	12892	1863	665	955	1278	715	1675	753	6821	15595	2478	3584	1629	4793	16377	164
0	0	0	0	0	0	0	0	0	0	0	2	0	0	0	0	0	15	0	0	12
604	271	285	408	376	863	3479	365	278	537	540	694	382	201	695	386	2259	567	302	643	4490
0	0	0	0	0	0	0	0	0	0	0	0	0	0	0	0	0	0	0	0	0
0	0	0	0	0	0	0	0	0	0	0	0	0	0	0	0	0	0	0	0	0

E3.010	E3.012	E3.013	E3.016	E3.017	E3.018	E3.020	E3.021	E3.024	E3.025	E3.026	E3.027	E3.029	E3.030	E3.031	E3.032	E3.033	E3.034	E3.036	E3.037	E3.038
Esophag	Esophag	Esophag	Esophag	Esophag	Esophag	Esophag	Esophag	Esophag	Esophag	Esophag	Esophag	Esophag	Esophag	Esophag	Esophag	Esophag	Esophag	Esophag	Esophag	Esophagus
LE	LE	LE	LE	LE	LE	LE	LE	LE	LE	LE	LE	LE	LE	LE	LE	LE	LE	LE	LE	LE
145	15	0	0	11	46	50	18	47	42	21	52	23	0	9	12	586	35	0	39	848
47	41	155	134	35	358	106	86	152	102	262	69	140	169	36	208	245	247	75	147	89
0	0	0	0	0	0	0	0	0	0	0	0	0	0	0	0	0	0	0	0	0
1	0	0	0	0	0	0	0	0	0	0	0	0	0	0	0	0	0	0	0	0
0	0	0	0	0	0	0	0	0	0	0	0	0	0	0	0	0	0	0	0	0
0	0	0	0	0	0	0	0	0	0	0	0	0	0	0	0	0	0	0	0	0
0	0	0	0	0	0	0	0	0	0	0	0	0	0	0	0	0	0	0	0	0
0	0	0	0	0	0	25	0	0	0	0	0	1	0	0	0	0	0	0	5	0
162	64	270	238	119	536	196	3682	237	146	241	342	663	195	181	83	120	808	638	101	87
0	0	0	0	0	0	0	0	0	0	0	0	0	0	0	0	0	0	0	0	0
0	0	0	0	0	0	0	0	0	0	0	0	0	0	0	0	0	0	0	0	0
95	49	105	71	123	159	115	729	99	127	260	178	231	179	53	64	142	928	989	34	186
10	3	0	0	0	0	0	0	0	9	0	5	15	0	12	0	0	0	0	0	0
12	5	27	11	3	51	39	25	8	10	11	20	71	21	6	8	70	15	6	2	9
1	2	0	0	0	0	0	0	0	1	0	2	1	0	0	0	0	0	0	10	0
1066	515	1	123	31	287	49	1268	6569	903	14844	3165	600	102	93	50	96	340	426	54	12
0	0	0	0	0	0	0	0	0	0	0	0	0	0	0	0	0	0	0	0	0
83	57	21	100	64	45	73	370	122	141	83	103	230	85	82	54	556	458	5193	120	558
3	0	0	0	0	0	0	0	0	1	0	1	4	0	0	0	0	0	0	0	12
0	0	0	0	0	0	0	0	0	0	0	0	0	0	0	0	0	0	0	0	0
396	1807	32	2169	750	1066	53	294	299	1389	686	172	451	1754	641	3313	270	880	680	217	169
1	1	0	0	2	28	0	28	1	1	3	15	21	3	3	0	27	0	17	2	1
0	0	0	0	0	0	0	0	0	0	0	0	0	0	0	0	0	0	0	0	0
0	0	0	0	0	0	0	0	0	0	0	0	0	0	0	0	0	0	0	0	0
1	1	0	0	0	0	0	0	0	0	0	2	0	0	0	0	0	0	0	0	0
0	0	0	0	0	0	0	0	1	0	0	0	0	0	1	18	0	0	0	4	0
5	1	0	0	0	0	11	0	0	6	0	6	0	0	5	0	96	0	0	0	29
0	0	0	0	0	0	0	0	0	0	0	0	0	0	0	0	0	0	0	0	0
0	0	0	0	0	0	0	0	0	0	0	0	7	0	0	0	0	0	0	0	0
0	0	0	0	0	0	0	0	0	0	0	0	0	0	0	0	0	0	0	0	0
3	1	0	0	0	0	0	0	0	6	0	2	0	0	0	0	0	0	0	0	0
0	0	0	0	0	0	0	0	0	0	0	0	0	0	0	0	0	0	0	0	0
0	0	0	0	0	0	0	0	0	0	0	0	0	0	0	0	0	0	0	0	0
4	49	0	0	4716	183	162	588	34	915	351	31	42	388	129	289	65	116	0	186	20
2	0	0	0	0	0	0	0	0	1	0	4	1	0	0	0	0	37	0	0	0
0	0	0	0	7	0	18	0	0	0	0	2	1	1	0	0	25	0	0	0	10
0	2	0	0	0	0	0	0	7	13	4	0	0	0	0	0	57	0	0	0	9

E3.010	E3.012	E3.013	E3.016	E3.017	E3.018	E3.020	E3.021	E3.024	E3.025	E3.026	E3.027	E3.029	E3.030	E3.031	E3.032	E3.033	E3.034	E3.036	E3.037	E3.038
Esophag	Esophag	Esophag	Esophag	Esophag	Esophag	Esophag	Esophag	Esophag	Esophag	Esophag	Esophag	Esophag	Esophag	Esophag	Esophag	Esophag	Esophag	Esophag	Esophag	Esophagus
LE	LE	LE	LE	LE	LE	LE	LE	LE	LE	LE	LE	LE	LE	LE	LE	LE	LE	LE	LE	LE
1483	208	142	101	70	2835	163	368	284	228	799	365	1381	87	192	94	189	4707	4724	497	402
0	0	0	0	0	0	15	21	0	0	0	0	0	2	12	0	0	0	0	0	0
0	0	0	0	0	0	0	0	0	0	0	1	2	0	0	0	52	0	0	0	16
330	334	69	6285	4966	909	533	1958	680	1843	396	425	1080	6662	681	2466	1652	347	2542	135	57
228	285	8	98	11	133	45	322	154	291	131	245	861	117	50	276	245	301	3289	17	69
0	0	0	0	0	0	0	0	0	0	0	0	0	0	0	0	0	0	0	0	0
0	0	0	0	0	0	0	0	0	0	0	0	0	0	0	0	0	0	0	0	0
53	24	22	73	1	49	10	210	49	28	171	55	93	17	15	1	78	60	335	20	29
0	0	0	0	0	0	0	0	0	0	0	0	0	0	0	0	0	0	0	0	0
0	0	0	0	0	0	0	0	0	0	0	0	0	0	0	0	0	0	0	0	0
0	0	0	0	0	0	0	0	0	0	0	0	0	0	0	0	0	0	0	3	0
3	1	0	0	0	75	25	0	12	14	20	22	1	26	74	6	68	13	3	25	23
0	0	0	0	0	12	0	0	1	0	3	1	0	4	6	5	0	0	0	4	0
0	0	0	0	0	0	0	0	0	8	0	2	1	0	20	0	0	0	0	11	0
26	39	0	0	0	0	0	0	11	4	19	5	5	0	0	1	19	0	0	3	0
0	0	0	0	0	0	0	0	2	5	2	8	7	0	2	0	0	0	0	0	0
1	0	0	0	0	0	0	0	0	0	0	0	0	0	7	0	0	0	0	0	0
0	0	0	0	0	0	0	0	0	0	0	0	2	0	0	0	0	0	0	0	10
0	0	0	0	0	0	0	0	0	0	0	0	0	0	0	0	0	0	0	0	0
3	0	24	62	2	1	0	0	1	205	5	12	3	25	24	48	0	17	0	0	5
0	0	0	0	0	0	0	0	0	0	0	1	4	0	0	0	33	0	8	0	0
21	9	34	0	0	12	0	0	25	399	4	34	16	0	3	28	0	0	51	14	0
0	0	0	0	0	0	0	0	0	0	0	0	0	0	0	0	0	0	0	0	0
0	0	0	0	0	0	0	0	0	0	0	0	0	0	2	0	0	0	0	0	62
0	0	0	0	0	0	0	0	0	0	0	5	0	0	0	0	0	0	0	0	0
1	2	0	0	0	0	0	0	0	4	0	5	7	0	0	0	0	0	14	0	10
0	0	0	0	0	0	0	0	0	0	0	0	0	0	10	0	0	0	0	0	0
0	0	10	0	0	0	0	0	0	1	0	1	0	0	0	0	0	0	0	0	0
0	0	0	0	0	0	0	0	0	0	0	0	0	0	0	0	0	0	0	0	0
0	0	0	0	0	0	0	0	0	0	0	0	0	0	0	0	0	0	0	0	0
0	0	0	0	0	0	0	0	0	0	0	1	3	0	0	0	0	0	0	0	0
0	0	0	0	0	0	0	0	0	0	0	0	0	0	0	0	0	0	0	0	0
0	0	0	0	0	0	0	0	0	1	0	0	0	0	0	0	0	0	0	0	0
0	0	0	0	5	0	0	0	0	0	0	0	1	0	0	0	0	0	0	0	0
9	128	51	2	64	0	14	14	18	89	6	10	24	23	82	29	75	185	29	332	39
0	0	0	0	0	0	0	0	0	0	0	0	0	0	0	0	0	0	0	0	0
0	0	0	0	0	0	0	0	0	1	0	1	0	0	0	0	0	0	0	0	0

E3.010	E3.012	E3.013	E3.016	E3.017	E3.018	E3.020	E3.021	E3.024	E3.025	E3.026	E3.027	E3.029	E3.030	E3.031	E3.032	E3.033	E3.034	E3.036	E3.037	E3.038
Esophag	Esophag	Esophag	Esophag	Esophag	Esophag	Esophag	Esophag	Esophag	Esophag	Esophag	Esophag	Esophag	Esophag	Esophag	Esophag	Esophag	Esophag	Esophag	Esophag	Esophagus
LE	LE	LE	LE	LE	LE	LE	LE	LE	LE	LE	LE	LE	LE	LE	LE	LE	LE	LE	LE	LE
11	7	0	0	16	1	43	0	4	10	4	7	49	15	9	0	29	15	14	3	64
0	0	0	0	0	0	0	0	0	0	0	1	0	0	1	0	0	0	0	0	0
1	0	0	0	0	0	0	0	0	0	0	0	0	0	0	0	0	0	0	0	0
0	0	0	0	0	0	0	0	0	0	0	0	0	0	0	0	0	0	0	0	0
0	0	0	0	0	0	0	0	0	0	0	0	0	0	0	0	0	0	0	0	0
0	0	0	0	0	0	0	0	0	0	0	0	0	0	0	0	0	0	0	0	0
0	0	0	0	0	0	0	0	0	2	0	0	4	0	1	0	0	0	0	0	0
0	0	0	0	0	0	0	0	0	0	0	0	0	0	0	0	0	0	0	0	0
1	1	0	0	0	18	0	0	0	3	2	0	10	9	1	0	0	147	29	0	19
0	0	0	0	0	0	0	0	0	0	0	0	0	0	0	0	0	0	0	0	0
1	0	0	0	0	0	0	0	0	0	0	1	0	0	0	0	0	0	0	0	0
1	0	0	0	0	0	10	18	1	3	2	2	6	0	6	0	0	0	29	1	0
5	11	111	8	86	5	28	39	12	21	20	7	14	41	203	139	20	105	118	378	16
3	0	0	0	0	10	0	0	0	1	0	3	4	0	3	0	0	32	0	0	8
0	0	0	0	0	0	0	0	0	0	0	0	0	0	0	0	0	0	0	0	0
5	0	0	8	0	0	0	0	10	1	9	8	4	0	0	0	0	0	102	7	0
2	0	0	0	0	0	15	0	0	0	1	0	1	9	0	0	0	0	1	0	0
0	0	0	0	0	0	0	0	0	0	0	0	0	0	0	0	6	0	0	0	7
0	0	0	0	0	0	0	0	0	0	0	0	3	0	6	0	0	0	0	0	0
25	6	0	0	30	0	0	0	4	21	8	16	267	8	20	0	24	204	210	7	80
0	0	0	0	0	0	0	0	0	0	0	1	0	2	0	0	14	0	0	0	10
0	0	0	0	0	0	0	0	0	0	0	0	0	0	3	0	0	0	0	0	0
0	0	0	0	0	0	0	0	0	0	2	1	0	0	0	0	0	0	0	0	0
2	0	0	12	0	24	24	13	1	4	0	0	6	0	7	0	25	188	371	0	0
0	0	0	0	0	0	0	0	0	0	0	0	0	0	0	0	0	0	0	0	0
0	0	0	0	0	0	0	0	0	0	0	0	0	0	0	0	0	7	0	0	3
726	179	1	0	0	0	0	0	227	151	325	107	304	0	4	0	0	0	0	7	538
68	16	0	0	1	0	0	0	11	28	21	15	17	0	1	0	0	1	0	13	91
0	0	0	0	0	0	0	0	0	0	0	0	0	0	0	0	0	0	0	0	0
0	0	0	0	0	0	0	0	0	0	0	0	0	0	0	0	0	0	0	0	0
0	0	0	0	0	0	0	0	0	0	0	0	0	0	0	0	0	0	18	0	0
0	0	0	0	0	0	0	0	0	0	0	0	0	0	0	0	0	0	0	0	0
4	4	0	0	0	0	0	0	0	6	8	8	20	0	0	8	0	2	0	0	0
0	0	0	0	0	0	0	0	0	0	0	0	0	0	0	0	0	0	0	0	0
54	19	50	14	20	19	19	6	5	88	21	41	816	32	13	8	0	24	9	0	9
0	0	0	0	7	0	0	0	0	2	0	4	16	0	0	0	0	0	0	0	0
0	0	0	0	0	0	0	0	0	0	0	0	0	0	0	0	0	0	0	0	0

E3.010	E3.012	E3.013	E3.016	E3.017	E3.018	E3.020	E3.021	E3.024	E3.025	E3.026	E3.027	E3.029	E3.030	E3.031	E3.032	E3.033	E3.034	E3.036	E3.037	E3.038
Esophag	Esophag	Esophag	Esophag	Esophag	Esophag	Esophag	Esophag	Esophag	Esophag	Esophag	Esophag	Esophag	Esophag	Esophag	Esophag	Esophag	Esophag	Esophag	Esophag	Esophagus
LE	LE	LE	LE	LE	LE	LE	LE	LE	LE	LE	LE	LE	LE	LE	LE	LE	LE	LE	LE	LE
1	0	0	0	0	0	0	0	0	0	0	0	0	0	0	0	41	10	0	0	0
267	991	105	3355	296	580	498	59	256	850	241	363	161	424	1810	1709	258	212	508	280	33
36	10	0	0	0	21	0	0	25	136	14	144	43	0	22	18	436	34	1	0	482
0	1	0	0	0	0	0	0	0	0	0	11	0	0	0	0	0	0	0	0	0
0	0	0	0	0	0	0	0	0	0	0	0	0	0	0	0	0	0	0	0	0
138	167	104	997	26	491	175	117	42	366	321	105	133	385	1018	701	192	195	100	216	141
44	867	95	157	419	582	1956	397	110	576	221	109	278	247	567	429	598	214	168	700	669
0	0	0	0	0	0	0	0	0	0	0	0	0	0	0	0	0	0	0	0	0
1	0	25	0	0	0	0	0	0	9	0	4	0	0	5	0	17	0	0	0	24
87	9	1	11	3	38	2	8	7	23	7	44	18	0	8	0	486	74	33	0	825
0	0	0	0	0	0	0	0	0	0	0	0	0	0	0	0	0	0	0	0	0
1	0	0	0	0	0	0	0	0	1	1	1	6	0	0	0	0	0	0	0	0
16	8	180	390	0	23	6	11	111	64	28	39	125	173	33	150	25	35	0	66	21
0	0	0	0	0	0	0	0	0	0	0	0	0	0	0	0	0	0	0	0	0
8	36	1	18	25	32	0	64	35	68	41	24	66	30	120	181	35	32	25	12	24
3	1	0	0	0	1	0	0	0	3	5	0	17	0	2	0	0	0	0	0	0
0	1	0	0	0	0	0	0	0	0	0	1	1	0	0	0	0	0	0	0	0
0	0	0	0	0	0	0	0	0	0	0	0	0	0	0	0	0	0	0	0	0
0	0	0	0	0	0	0	0	0	0	0	0	0	0	0	0	0	0	0	0	0
0	0	0	0	0	0	0	0	0	0	1	0	0	0	0	0	0	0	0	0	0
0	0	0	0	0	0	0	0	0	0	0	0	0	0	0	0	0	0	26	0	0
1	0	0	0	0	0	0	0	0	0	0	0	0	0	0	0	0	0	0	0	0
0	0	0	0	0	16	0	0	0	0	0	0	0	0	0	0	0	0	0	0	0
0	0	0	0	0	0	0	0	0	0	0	0	0	0	0	0	0	0	0	0	0
0	0	0	0	0	0	0	0	0	0	0	0	0	0	0	0	0	0	0	0	0
0	0	0	0	0	0	0	0	0	0	0	0	0	0	0	0	0	0	0	0	0
0	0	0	0	0	0	0	0	0	0	0	0	0	0	0	0	0	0	0	0	0
42	165	0	0	0	0	0	0	14	27	30	77	79	0	11	0	27	0	158	0	95
0	0	0	0	0	0	0	0	0	0	0	1	2	0	0	0	0	0	0	0	0
1	0	0	0	0	0	0	0	15	4	0	2	3	0	0	0	0	2716	0	0	0
0	2	0	0	0	0	0	2	0	2	1	1	5	0	2	0	0	0	0	0	13
0	0	0	0	0	0	0	0	0	0	0	0	0	0	0	0	0	0	0	0	0
16	6	0	148	31	80	32	339	6	10	1	15	430	19	44	3	66	161	69	3	15
0	0	0	0	0	0	0	0	0	0	0	0	0	0	0	0	0	0	0	0	0
18	121	123	90	145	275	335	91	131	171	118	117	209	1627	757	291	105	197	818	1965	0
24	60	0	0	0	0	25	0	11	12	7	13	19	13	23	0	18	0	46	1	6
11	68	45	104	14	68	18	0	69	116	26	31	35	465	359	160	240	81	66	49	13

E3.010	E3.012	E3.013	E3.016	E3.017	E3.018	E3.020	E3.021	E3.024	E3.025	E3.026	E3.027	E3.029	E3.030	E3.031	E3.032	E3.033	E3.034	E3.036	E3.037	E3.038
Esophag	Esophag	Esophag	Esophag	Esophag	Esophag	Esophag	Esophag	Esophag	Esophag	Esophag	Esophag	Esophag	Esophag	Esophag	Esophag	Esophag	Esophag	Esophag	Esophag	Esophagus
LE	LE	LE	LE	LE	LE	LE	LE	LE	LE	LE	LE	LE	LE	LE	LE	LE	LE	LE	LE	LE
11	0	0	0	0	0	0	0	4	3	6	3	21	0	2	0	54	0	0	0	35
157	138	491	0	1	80	10	0	24	90	76	93	200	0	17	0	102	0	0	170	27
3	0	0	0	0	0	0	0	0	2	0	2	1	0	0	0	0	0	0	0	0
251	489	8007	1390	4813	12744	1810	665	847	1049	600	1529	468	6343	15194	2315	3170	1548	4681	16157	83
0	0	0	0	0	0	0	0	0	0	0	2	0	0	0	0	0	15	0	0	12
0	0	0	0	0	0	0	0	0	0	0	0	0	0	0	0	0	0	0	0	0
10	17	79	0	15	49	19	0	14	31	18	14	18	52	57	73	86	17	24	107	107
17	10	0	0	0	0	0	83	14	13	8	5	51	0	7	0	0	0	37	44	126
4	0	2	0	0	78	0	0	2	1	0	0	0	0	0	0	207	0	0	0	109
27	7	0	0	0	86	0	0	4	27	110	18	23	0	1	0	95	0	0	4	332
0	0	0	0	0	0	0	0	0	0	0	1	0	0	0	0	0	0	0	0	0
0	0	0	0	0	0	0	0	0	0	0	1	0	0	0	0	0	0	0	0	0
283	115	83	89	83	74	382	0	114	201	231	366	112	0	75	14	1102	170	1	57	2281
1	1	0	0	0	0	0	0	4	0	13	2	0	0	1	0	6	0	0	7	1
43	21	0	0	14	171	0	0	24	22	69	27	18	45	41	100	3	169	74	96	10
115	25	3	62	7	194	0	64	14	101	18	146	79	0	11	0	449	91	105	12	891
0	0	0	0	0	0	0	0	0	0	0	0	0	0	0	0	0	0	0	0	0
42	61	115	218	255	93	3037	125	64	77	61	47	48	94	453	170	48	75	61	306	385
0	0	0	0	0	0	0	0	0	0	0	0	0	0	0	0	0	0	0	0	0
145	15	0	0	11	46	50	18	47	42	21	52	23	0	9	12	586	35	0	39	848
14	13	150	64	9	302	38	86	92	83	239	51	130	169	15	201	196	247	55	141	27
33	28	5	70	26	56	68	0	60	19	23	18	10	0	21	7	49	0	20	6	62
1	0	0	0	0	0	0	0	0	0	0	0	0	0	0	0	0	0	0	0	0
0	0	0	0	0	0	0	0	0	0	0	0	0	0	0	0	0	0	0	0	0
162	64	270	238	119	536	196	3682	237	146	241	342	663	195	181	83	120	808	638	101	87
0	0	0	0	0	0	0	0	0	0	0	0	0	0	0	0	0	0	0	0	0
0	0	0	0	0	0	0	0	0	0	0	0	0	0	0	0	0	0	62	0	0
1	0	0	0	0	0	0	0	0	0	0	3	0	0	0	0	0	0	0	0	0
20	5	90	9	57	44	77	77	3	21	33	29	150	62	26	16	9	219	268	24	23
28	24	2	2	0	24	1	306	69	46	214	56	40	8	10	20	81	536	168	1	18
2	5	2	28	0	0	0	0	3	0	0	1	3	0	5	0	0	23	0	0	0
34	11	11	32	52	49	36	197	21	56	8	78	29	109	9	4	42	149	79	3	137
1	0	0	0	0	0	0	0	0	0	3	0	0	0	0	0	0	0	0	0	0
1	2	0	0	2	0	1	1	1	3	2	9	3	0	0	0	10	0	27	0	0
6	1	0	0	0	42	0	148	1	1	0	0	6	0	2	24	0	0	262	0	1
0	0	0	0	0	0	0	0	0	0	0	0	0	0	0	0	0	0	50	0	0
0	0	0	0	0	0	0	0	0	0	0	1	0	0	0	0	0	0	0	0	0

E3.010	E3.012	E3.013	E3.016	E3.017	E3.018	E3.020	E3.021	E3.024	E3.025	E3.026	E3.027	E3.029	E3.030	E3.031	E3.032	E3.033	E3.034	E3.036	E3.037	E3.038
Esophag	Esophag	Esophag	Esophag	Esophag	Esophag	Esophag	Esophag	Esophag	Esophag	Esophag	Esophag	Esophag	Esophag	Esophag	Esophag	Esophag	Esophag	Esophag	Esophag	Esophagus
LE	LE	LE	LE	LE	LE	LE	LE	LE	LE	LE	LE	LE	LE	LE	LE	LE	LE	LE	LE	LE
10	3	0	0	0	0	0	0	0	9	0	4	15	0	12	0	0	0	0	0	0
4	1	0	11	2	0	2	25	5	2	1	3	2	17	0	3	63	15	6	2	9
8	4	27	0	1	51	37	0	3	8	10	17	69	4	6	5	7	0	0	0	0
0	0	0	0	0	0	0	0	0	0	0	0	0	0	0	0	0	0	0	10	0
0	0	0	0	0	0	0	0	0	0	0	0	0	0	0	0	0	0	0	0	0
0	0	0	0	0	0	0	0	0	0	0	0	0	0	0	0	0	0	0	0	0
2	1	0	0	0	2	0	0	0	2	0	2	7	6	10	0	0	0	0	0	0
1064	511	1	123	31	285	49	1268	6569	900	14844	3163	593	96	83	50	96	340	424	54	12
10	8	0	0	0	0	0	0	6	1	20	4	12	0	0	0	418	177	0	0	499
4	21	0	12	23	0	35	0	15	54	40	15	59	43	15	32	18	78	1	78	0
23	15	20	36	12	34	0	84	26	54	23	50	107	20	53	1	36	128	5003	32	29
45	12	1	52	29	11	38	286	75	30	0	34	52	22	13	21	84	75	189	10	30
3	0	0	0	0	0	0	0	0	1	0	1	4	0	0	0	0	0	0	0	12
396	1807	32	2169	750	1066	53	294	299	1389	686	172	451	1754	641	3313	270	880	680	217	169
1	1	0	0	2	28	0	28	1	1	3	15	21	3	3	0	27	0	17	2	1
1	1	0	0	0	0	0	0	0	0	0	0	0	0	0	0	0	0	0	0	0
0	0	0	0	0	0	0	0	0	0	0	2	0	0	0	0	0	0	0	0	0
0	0	0	0	0	0	0	0	1	0	0	0	0	0	1	18	0	0	0	4	0
0	0	0	0	0	0	0	0	0	0	0	0	0	0	4	0	0	0	0	0	0
5	1	0	0	0	0	11	0	0	6	0	6	0	0	1	0	96	0	0	0	29
0	0	0	0	0	0	0	0	0	5	0	0	0	0	0	0	0	0	0	0	0
0	0	0	0	0	0	0	0	0	0	0	0	0	0	0	0	0	0	0	0	0
0	0	0	0	0	0	0	0	0	0	0	0	0	0	0	0	0	0	0	0	0
0	0	0	0	0	0	0	0	0	0	0	0	0	0	0	0	0	0	0	0	0
3	14	0	0	34	44	162	2	32	18	59	19	41	11	110	239	65	116	0	186	20
1	35	0	0	4682	139	0	586	2	897	292	12	1	377	19	0	0	0	0	0	0
1	0	0	0	0	0	0	0	0	1	0	0	0	0	0	0	0	37	0	0	0
1	0	0	0	0	0	0	0	0	0	0	4	1	0	0	0	0	0	0	0	0
0	0	0	0	7	0	0	0	0	0	0	0	1	1	0	0	0	0	0	0	0
0	0	0	0	0	0	0	0	0	0	0	2	0	0	0	0	25	0	0	0	10
0	0	0	0	0	0	18	0	0	0	0	0	0	0	0	0	0	0	0	0	0
0	2	0	0	0	0	0	0	7	7	4	0	0	0	0	0	57	0	0	0	9
1483	208	142	101	70	2835	163	368	284	228	799	365	1381	87	192	94	189	4707	4724	497	402
0	0	0	0	0	0	0	0	0	0	0	0	0	0	0	0	0	0	0	0	0
0	0	0	0	0	0	15	21	0	0	0	0	0	2	12	0	0	0	0	0	0
0	0	0	0	0	0	0	0	0	0	0	0	0	0	0	0	52	0	0	0	16
0	0	0	0	0	0	0	0	0	0	0	1	2	0	0	0	0	0	0	0	0

E3.010	E3.012	E3.013	E3.016	E3.017	E3.018	E3.020	E3.021	E3.024	E3.025	E3.026	E3.027	E3.029	E3.030	E3.031	E3.032	E3.033	E3.034	E3.036	E3.037	E3.038
Esophag	Esophag	Esophag	Esophag	Esophag	Esophag	Esophag	Esophag	Esophag	Esophag	Esophag	Esophag	Esophag	Esophag	Esophag	Esophag	Esophag	Esophag	Esophag	Esophag	Esophagus
LE	LE	LE	LE	LE	LE	LE	LE	LE	LE	LE	LE	LE	LE	LE	LE	LE	LE	LE	LE	LE
330	334	69	6285	4966	909	533	1958	680	1843	396	425	1080	6662	681	2466	1652	347	2542	135	57
46	41	8	19	11	109	16	69	8	99	24	61	114	111	43	276	216	179	3179	11	46
182	244	0	79	0	24	29	253	146	191	107	184	746	6	7	0	29	122	110	6	23
0	0	0	0	0	0	0	0	0	0	0	0	0	0	0	0	0	0	0	0	0
0	0	0	0	0	0	0	0	0	0	0	0	0	0	0	0	0	0	0	0	0
0	0	0	0	0	0	0	0	0	0	0	0	0	0	0	0	0	0	0	0	0
4	3	0	9	1	0	0	48	0	4	7	7	17	0	8	1	0	29	0	11	0
49	21	22	64	0	49	10	162	49	24	164	48	76	17	7	0	78	31	335	9	29
0	0	0	0	0	0	0	0	0	0	0	0	0	0	0	0	0	0	0	3	0
3	1	0	0	0	75	25	0	12	14	20	22	1	26	74	6	68	13	3	25	23
0	0	0	0	0	12	0	0	1	0	3	1	0	4	6	5	0	0	0	4	0
0	0	0	0	0	0	0	0	0	0	0	0	0	0	0	0	0	0	0	0	0
0	0	0	0	0	0	0	0	0	8	0	2	1	0	20	0	0	0	0	11	0
26	39	0	0	0	0	0	0	11	4	19	5	5	0	0	1	19	0	0	3	0
0	0	0	0	0	0	0	0	0	0	0	0	2	0	0	0	0	0	0	0	10
0	0	24	0	2	0	0	0	1	0	3	1	2	1	0	6	0	17	0	0	5
21	9	34	0	0	12	0	0	25	399	4	34	16	0	3	28	0	0	51	14	0
0	0	0	0	0	0	0	0	0	0	0	0	0	0	0	0	0	0	0	0	0
0	0	0	0	0	0	0	0	0	0	0	0	0	0	2	0	0	0	0	0	62
1	2	0	0	0	0	0	0	0	4	0	5	7	0	0	0	0	0	14	0	10
0	0	0	0	0	0	0	0	0	0	0	0	0	0	10	0	0	0	0	0	0
0	0	10	0	0	0	0	0	0	1	0	1	0	0	0	0	0	0	0	0	0
0	0	0	0	0	0	0	0	0	0	0	0	0	0	0	0	0	0	0	0	0
0	0	0	0	0	0	0	0	0	0	0	0	0	0	0	0	0	0	0	0	0
0	0	0	0	0	0	0	0	0	0	0	1	3	0	0	0	0	0	0	0	0
0	0	0	0	0	0	0	0	0	0	0	0	0	0	0	0	0	0	0	0	0
0	0	0	0	0	0	0	0	0	1	0	0	0	0	0	0	0	0	0	0	0
0	0	0	0	5	0	0	0	0	0	0	0	1	0	0	0	0	0	0	0	0
7	126	51	2	64	0	13	14	18	89	6	10	24	18	81	29	75	152	29	284	29
0	0	0	0	0	0	0	0	0	0	0	0	0	0	0	0	0	0	0	0	10
0	0	0	0	0	0	0	0	0	0	0	0	0	0	1	0	0	0	0	0	0
1	2	0	0	0	0	0	0	0	0	0	0	0	0	0	0	0	0	0	0	0
1	0	0	0	0	0	1	0	0	0	0	0	0	5	0	0	0	33	0	48	0
0	0	0	0	0	0	0	0	0	0	0	0	0	0	0	0	0	0	0	0	0
0	0	0	0	0	0	0	0	0	1	0	1	0	0	0	0	0	0	0	0	0
11	7	0	0	16	1	43	0	4	10	4	7	49	15	9	0	29	15	14	3	64
0	0	0	0	0	0	0	0	0	0	0	1	0	0	1	0	0	0	0	0	0

E3.010	E3.012	E3.013	E3.016	E3.017	E3.018	E3.020	E3.021	E3.024	E3.025	E3.026	E3.027	E3.029	E3.030	E3.031	E3.032	E3.033	E3.034	E3.036	E3.037	E3.038
Esophag	Esophag	Esophag	Esophag	Esophag	Esophag	Esophag	Esophag	Esophag	Esophag	Esophag	Esophag	Esophag	Esophag	Esophag	Esophag	Esophag	Esophag	Esophag	Esophag	Esophagus
LE	LE	LE	LE	LE	LE	LE	LE	LE	LE	LE	LE	LE	LE	LE	LE	LE	LE	LE	LE	LE
1	0	0	0	0	0	0	0	0	0	0	0	0	0	0	0	0	0	0	0	0
0	0	0	0	0	0	0	0	0	0	0	0	0	0	0	0	0	0	0	0	0
0	0	0	0	0	0	0	0	0	0	0	0	0	0	0	0	0	0	0	0	0
0	0	0	0	0	0	0	0	0	0	0	0	0	0	0	0	0	0	0	0	0
0	0	0	0	0	0	0	0	0	0	0	0	0	0	0	0	0	0	0	0	0
0	0	0	0	0	0	0	0	0	0	0	0	0	0	0	0	0	0	0	0	0
0	0	0	0	0	0	0	0	0	1	0	0	0	0	0	0	0	0	0	0	0
0	0	0	0	0	0	0	0	0	0	0	0	2	0	0	0	0	0	0	0	0
0	0	0	0	0	0	0	0	0	1	0	0	2	0	1	0	0	0	0	0	0
0	0	0	0	0	0	0	0	0	0	0	0	0	0	0	0	0	0	0	0	0
0	0	0	0	0	0	0	0	0	0	0	0	0	0	0	0	0	0	0	0	0
1	0	0	0	0	0	0	0	0	0	0	0	0	0	0	0	0	0	0	0	0
0	1	0	0	0	18	0	0	0	3	2	0	10	9	1	0	0	147	29	0	19
0	0	0	0	0	0	0	0	0	0	0	0	0	0	0	0	0	0	0	0	0
0	0	0	0	0	0	0	0	0	0	0	0	0	0	0	0	0	0	0	0	0
1	0	0	0	0	0	0	0	0	0	0	1	0	0	0	0	0	0	0	0	0
0	0	0	0	0	0	0	0	1	0	0	0	0	0	0	0	0	0	1	0	0
0	0	0	0	0	0	0	0	0	0	0	0	0	0	0	0	0	0	0	0	0
0	0	0	0	0	0	0	0	0	0	0	0	0	0	0	0	0	0	0	0	0
0	0	0	0	0	0	0	0	0	0	0	0	0	0	0	0	0	0	0	0	0
1	0	0	0	0	0	8	18	0	2	2	0	1	0	3	0	0	0	22	0	0
0	0	0	0	0	0	0	0	0	0	0	0	0	0	0	0	0	0	1	0	0
0	0	0	0	0	0	0	0	0	0	0	0	0	0	0	0	0	0	0	1	0
0	0	0	0	0	0	0	0	0	0	0	0	0	0	0	0	0	0	0	0	0
0	0	0	0	0	0	2	0	0	1	0	2	5	0	3	0	0	0	5	0	0
0	0	0	0	0	0	0	0	0	0	0	0	0	0	1	0	0	0	22	0	3
0	0	0	0	0	0	0	0	0	1	0	1	1	0	0	0	0	3	52	0	9
0	0	0	0	0	0	0	0	0	1	0	0	0	0	0	0	0	0	0	0	0
0	1	0	0	0	0	0	24	0	0	2	2	2	0	0	0	0	0	0	0	1
0	0	0	0	0	0	0	0	0	0	0	0	0	0	0	0	0	0	0	0	0
0	1	0	0	0	0	0	0	0	0	0	1	0	0	1	0	0	0	0	0	0
5	9	111	8	86	5	28	15	12	18	18	2	11	41	201	139	20	102	25	377	0
0	0	0	0	0	0	0	0	0	0	0	1	0	0	0	0	0	0	0	0	0
0	0	0	0	0	0	0	0	0	1	0	0	0	0	0	0	0	0	19	1	3
3	0	0	0	0	10	0	0	0	1	0	3	4	0	3	0	0	32	0	0	8
0	0	0	0	0	0	0	0	0	0	0	0	0	0	0	0	0	0	0	0	0
0	0	0	0	0	0	0	0	0	0	0	0	2	0	0	0	0	0	0	0	0

E3.010	E3.012	E3.013	E3.016	E3.017	E3.018	E3.020	E3.021	E3.024	E3.025	E3.026	E3.027	E3.029	E3.030	E3.031	E3.032	E3.033	E3.034	E3.036	E3.037	E3.038
Esophag	Esophag	Esophag	Esophag	Esophag	Esophag	Esophag	Esophag	Esophag	Esophag	Esophag	Esophag	Esophag	Esophag	Esophag	Esophag	Esophag	Esophag	Esophag	Esophag	Esophagus
LE	LE	LE	LE	LE	LE	LE	LE	LE	LE	LE	LE	LE	LE	LE	LE	LE	LE	LE	LE	LE
3	0	0	0	0	0	0	0	10	1	9	8	2	0	0	0	0	0	1	0	0
0	0	0	0	0	0	0	0	0	0	0	0	0	0	0	0	0	0	101	0	0
2	0	0	8	0	0	0	0	0	0	0	0	0	0	0	0	0	0	0	7	0
0	0	0	0	0	0	0	0	0	0	0	0	0	0	0	0	0	0	0	0	0
1	0	0	0	0	0	15	0	0	0	0	0	0	0	0	0	0	0	0	0	0
0	0	0	0	0	0	0	0	0	0	0	0	0	0	0	0	0	0	0	0	0
0	0	0	0	0	0	0	0	0	0	0	0	0	0	0	0	0	0	1	0	0
0	0	0	0	0	0	0	0	0	0	0	0	0	2	0	0	0	0	0	0	0
1	0	0	0	0	0	0	0	0	0	1	0	1	7	0	0	0	0	0	0	0
0	0	0	0	0	0	0	0	0	0	0	0	0	0	0	0	6	0	0	0	7
0	0	0	0	0	0	0	0	0	0	0	0	0	0	0	0	0	0	0	0	0
0	0	0	0	0	0	0	0	0	0	0	0	0	0	0	0	0	0	0	0	0
0	0	0	0	0	0	0	0	0	0	0	0	3	0	6	0	0	0	0	0	0
0	0	0	0	0	0	0	0	0	0	0	0	0	0	0	0	0	0	0	0	0
24	6	0	0	27	0	0	0	3	17	8	16	266	5	19	0	24	167	210	0	80
0	0	0	0	0	0	0	0	0	0	0	0	0	0	0	0	0	0	0	0	0
1	0	0	0	3	0	0	0	1	4	0	0	1	3	1	0	0	37	0	7	0
0	0	0	0	0	0	0	0	0	0	0	1	0	0	0	0	14	0	0	0	8
0	0	0	0	0	0	0	0	0	0	0	0	0	0	0	0	0	0	0	0	0
0	0	0	0	0	0	0	0	0	0	0	0	0	0	0	0	0	0	0	0	2
0	0	0	0	0	0	0	0	0	0	0	0	0	2	0	0	0	0	0	0	0
0	0	0	0	0	0	0	0	0	0	0	0	0	0	3	0	0	0	0	0	0
0	0	0	0	0	0	0	0	0	0	2	1	0	0	0	0	0	0	0	0	0
2	0	0	12	0	24	24	13	1	4	0	0	6	0	7	0	25	188	371	0	0
0	0	0	0	0	0	0	0	0	0	0	0	0	0	0	0	0	0	0	0	0
0	0	0	0	0	0	0	0	0	0	0	0	0	0	0	0	0	7	0	0	3
0	0	0	0	0	0	0	0	0	0	0	0	0	0	0	0	0	0	0	0	0
22	9	1	0	0	0	0	0	11	9	2	14	14	0	3	0	0	0	0	6	22
1	0	0	0	0	0	0	0	1	2	0	0	0	0	0	0	0	0	0	0	0
0	0	0	0	0	0	0	0	0	1	0	0	2	0	1	0	0	0	0	0	0
0	2	0	0	0	0	0	0	4	11	1	1	4	0	0	0	0	0	0	0	0
703	168	0	0	0	0	0	0	211	128	322	92	284	0	0	0	0	0	0	1	516
0	0	0	0	0	0	0	0	1	0	0	0	0	0	0	0	0	0	0	0	18
1	5	0	0	1	0	0	0	1	1	2	2	1	0	1	0	0	1	0	13	0
0	0	0	0	0	0	0	0	0	0	0	1	0	0	0	0	0	0	0	0	0
0	0	0	0	0	0	0	0	0	0	0	0	0	0	0	0	0	0	0	0	0
0	0	0	0	0	0	0	0	0	0	0	0	0	0	0	0	0	0	0	0	0

E3.010	E3.012	E3.013	E3.016	E3.017	E3.018	E3.020	E3.021	E3.024	E3.025	E3.026	E3.027	E3.029	E3.030	E3.031	E3.032	E3.033	E3.034	E3.036	E3.037	E3.038
Esophag	Esophag	Esophag	Esophag	Esophag	Esophag	Esophag	Esophag	Esophag	Esophag	Esophag	Esophag	Esophag	Esophag	Esophag	Esophag	Esophag	Esophag	Esophag	Esophag	Esophagus
LE	LE	LE	LE	LE	LE	LE	LE	LE	LE	LE	LE	LE	LE	LE	LE	LE	LE	LE	LE	LE
0	1	0	0	0	0	0	0	0	0	0	0	1	0	0	0	0	0	0	0	0
67	10	0	0	0	0	0	0	9	27	19	12	15	0	0	0	0	0	0	0	73
0	0	0	0	0	0	0	0	0	0	0	0	0	0	0	0	0	0	0	0	0
0	0	0	0	0	0	0	0	0	0	0	0	0	0	0	0	0	0	0	0	0
0	0	0	0	0	0	0	0	0	0	0	0	0	0	0	0	0	0	18	0	0
0	0	0	0	0	0	0	0	0	0	0	0	0	0	0	0	0	0	0	0	0
4	4	0	0	0	0	0	0	0	6	8	8	20	0	0	8	0	2	0	0	0
0	0	0	0	0	0	0	0	0	0	0	0	0	0	0	0	0	0	0	0	0
0	0	0	0	0	0	0	0	0	0	0	0	0	0	0	0	0	0	0	0	0
0	0	0	0	0	0	0	0	0	0	0	0	0	0	0	0	0	0	0	0	0
0	0	0	0	0	0	0	0	0	0	0	0	0	0	0	0	0	0	0	0	0
51	16	50	14	20	19	19	6	5	85	21	41	810	32	13	8	0	24	9	0	0
3	3	0	0	0	0	0	0	0	3	0	0	6	0	0	0	0	0	0	0	9
0	0	0	0	0	0	0	0	0	2	0	0	0	0	0	0	0	0	0	0	0
0	0	0	0	7	0	0	0	0	0	0	4	16	0	0	0	0	0	0	0	0
0	0	0	0	0	0	0	0	0	0	0	0	0	0	0	0	0	0	0	0	0
1	0	0	0	0	0	0	0	0	0	0	0	0	0	0	0	41	0	0	0	0
0	0	0	0	0	0	0	0	0	0	0	0	0	0	0	0	0	10	0	0	0
267	991	105	3355	295	580	498	59	256	850	241	363	161	424	1810	1709	258	212	508	280	33
0	0	0	0	0	0	0	0	0	0	0	0	0	0	0	0	0	0	0	0	0
0	0	0	0	0	0	0	0	0	0	0	0	0	0	0	0	0	0	0	0	0
0	0	0	0	1	0	0	0	0	0	0	0	0	0	0	0	0	0	0	0	0
36	10	0	0	0	21	0	0	25	136	14	144	43	0	22	18	436	34	1	0	482
0	1	0	0	0	0	0	0	0	0	0	11	0	0	0	0	0	0	0	0	0
0	0	0	0	0	0	0	0	0	0	0	0	0	0	0	0	0	0	0	0	0
0	0	0	0	0	0	0	0	0	1	0	1	3	0	0	0	0	0	0	0	0
0	1	0	0	0	0	14	0	1	0	1	3	0	0	2	8	0	0	0	0	0
1	0	30	0	0	0	0	0	0	4	3	9	1	0	0	0	65	0	0	0	89
137	163	0	997	26	481	124	117	37	361	303	86	122	383	1007	684	127	154	100	204	36
0	3	74	0	0	10	37	0	4	0	14	6	4	2	5	9	0	41	0	12	16
0	0	0	0	0	0	0	0	0	0	0	0	3	0	4	0	0	0	0	0	0
44	867	95	157	419	582	1956	397	110	576	221	109	278	247	567	429	598	214	168	700	669
0	0	0	0	0	0	0	0	0	0	0	0	0	0	0	0	0	0	0	0	0
0	0	0	0	0	0	0	0	0	0	0	0	0	0	0	0	0	0	0	0	0
0	0	0	0	0	0	0	0	0	0	0	0	0	0	0	0	0	0	0	0	0
0	0	0	0	0	0	0	0	0	0	0	0	0	0	1	0	0	0	0	0	0
1	0	25	0	0	0	0	0	0	9	0	4	0	0	4	0	17	0	0	0	24

E3.010	E3.012	E3.013	E3.016	E3.017	E3.018	E3.020	E3.021	E3.024	E3.025	E3.026	E3.027	E3.029	E3.030	E3.031	E3.032	E3.033	E3.034	E3.036	E3.037	E3.038
Esophag	Esophag	Esophag	Esophag	Esophag	Esophag	Esophag	Esophag	Esophag	Esophag	Esophag	Esophag	Esophag	Esophag	Esophag	Esophag	Esophag	Esophag	Esophag	Esophag	Esophagus
LE	LE	LE	LE	LE	LE	LE	LE	LE	LE	LE	LE	LE	LE	LE	LE	LE	LE	LE	LE	LE
87	9	1	11	3	38	2	8	7	23	7	44	18	0	8	0	486	74	33	0	825
0	0	0	0	0	0	0	0	0	0	0	0	0	0	0	0	0	0	0	0	0
0	0	0	0	0	0	0	0	0	0	0	0	0	0	0	0	0	0	0	0	0
0	0	0	0	0	0	0	0	0	0	0	1	0	0	0	0	0	0	0	0	0
0	0	0	0	0	0	0	0	0	0	0	0	1	0	0	0	0	0	0	0	0
0	0	0	0	0	0	0	0	0	0	0	0	0	0	0	0	0	0	0	0	0
1	0	0	0	0	0	0	0	0	0	0	0	5	0	0	0	0	0	0	0	0
0	0	0	0	0	0	0	0	0	0	0	0	0	0	0	0	0	0	0	0	0
0	0	0	0	0	0	0	0	0	0	0	0	0	0	0	0	0	0	0	0	0
0	0	0	0	0	0	0	0	0	1	0	0	0	0	0	0	0	0	0	0	0
0	0	0	0	0	0	0	0	0	0	1	0	0	0	0	0	0	0	0	0	0
0	0	0	0	0	0	0	0	0	0	0	0	0	0	0	0	0	0	0	0	0
12	3	0	147	0	0	0	0	87	9	14	6	44	13	7	0	0	35	0	0	0
0	0	0	0	0	0	0	0	0	0	0	2	9	91	0	0	0	0	0	0	0
1	3	0	0	0	0	0	0	9	4	12	18	8	0	0	0	0	0	0	6	0
2	0	0	0	0	0	0	0	0	0	0	0	0	0	0	0	0	0	0	0	0
0	0	0	0	0	0	0	0	0	0	0	0	0	0	0	0	0	0	0	0	0
1	2	180	243	0	23	6	11	15	51	2	13	64	69	26	150	25	0	0	60	21
0	0	0	0	0	0	0	0	0	0	0	0	0	0	0	0	0	0	0	0	0
6	35	1	18	25	0	0	64	33	66	39	20	27	30	120	181	35	0	25	12	24
2	1	0	0	0	32	0	0	1	2	2	4	39	0	0	0	0	32	0	0	0
0	0	0	0	0	0	0	0	1	0	0	0	0	0	0	0	0	0	0	0	0
0	0	0	0	0	0	0	0	0	0	0	0	0	0	0	0	0	0	0	0	0
1	1	0	0	0	0	0	0	0	2	1	0	5	0	2	0	0	0	0	0	0
2	0	0	0	0	0	0	0	0	1	4	0	6	0	0	0	0	0	0	0	0
0	0	0	0	0	1	0	0	0	0	0	0	6	0	0	0	0	0	0	0	0
0	1	0	0	0	0	0	0	0	0	0	1	1	0	0	0	0	0	0	0	0
0	0	0	0	0	0	0	0	0	0	0	0	0	0	0	0	0	0	0	0	0
0	0	0	0	0	0	0	0	0	0	0	0	0	0	0	0	0	0	0	0	0
0	0	0	0	0	0	0	0	0	0	1	0	0	0	0	0	0	0	0	0	0
0	0	0	0	0	0	0	0	0	0	0	0	0	0	0	0	0	0	0	0	0
0	0	0	0	0	0	0	0	0	0	0	0	0	0	0	0	0	0	26	0	0
1	0	0	0	0	0	0	0	0	0	0	0	0	0	0	0	0	0	0	0	0
0	0	0	0	0	16	0	0	0	0	0	0	0	0	0	0	0	0	0	0	0
0	0	0	0	0	0	0	0	0	0	0	0	0	0	0	0	0	0	0	0	0
0	0	0	0	0	0	0	0	0	0	0	0	0	0	0	0	0	0	0	0	0
0	0	0	0	0	0	0	0	0	0	0	0	0	0	0	0	0	0	0	0	0

E3.010	E3.012	E3.013	E3.016	E3.017	E3.018	E3.020	E3.021	E3.024	E3.025	E3.026	E3.027	E3.029	E3.030	E3.031	E3.032	E3.033	E3.034	E3.036	E3.037	E3.038
Esophag	Esophag	Esophag	Esophag	Esophag	Esophag	Esophag	Esophag	Esophag	Esophag	Esophag	Esophag	Esophag	Esophag	Esophag	Esophag	Esophag	Esophag	Esophag	Esophag	Esophagus
LE	LE	LE	LE	LE	LE	LE	LE	LE	LE	LE	LE	LE	LE	LE	LE	LE	LE	LE	LE	LE
0	0	0	0	0	0	0	0	0	0	0	0	0	0	0	0	0	0	0	0	0
0	0	0	0	0	0	0	0	0	0	0	0	1	0	2	0	27	0	0	0	0
42	165	0	0	0	0	0	0	14	27	30	76	78	0	8	0	0	0	91	0	0
0	0	0	0	0	0	0	0	0	0	0	0	0	0	1	0	0	0	18	0	95
0	0	0	0	0	0	0	0	0	0	0	0	0	0	0	0	0	0	0	0	0
0	0	0	0	0	0	0	0	0	0	0	0	0	0	0	0	0	0	0	0	0
0	0	0	0	0	0	0	0	0	0	0	1	0	0	0	0	0	0	49	0	0
0	0	0	0	0	0	0	0	0	0	0	1	2	0	0	0	0	0	0	0	0
0	0	0	0	0	0	0	0	0	0	0	0	0	0	0	0	0	0	0	0	0
1	0	0	0	0	0	0	0	15	3	0	2	3	0	0	0	0	2716	0	0	0
0	0	0	0	0	0	0	0	0	1	0	0	0	0	0	0	0	0	0	0	0
0	0	0	0	0	0	0	0	0	0	0	0	2	0	0	0	0	0	0	0	0
0	0	0	0	0	0	0	0	0	0	0	0	1	0	0	0	0	0	0	0	0
0	0	0	0	0	0	0	0	0	0	0	0	0	0	0	0	0	0	0	0	0
0	2	0	0	0	0	0	2	0	2	1	1	2	0	2	0	0	0	0	0	13
0	0	0	0	0	0	0	0	0	0	0	0	0	0	0	0	0	0	0	0	0
0	0	0	0	0	0	0	0	1	0	0	0	5	0	0	0	0	0	0	0	0
0	0	0	0	0	0	0	0	0	0	0	0	2	0	0	0	0	0	0	0	0
0	0	0	0	0	0	0	0	0	0	0	0	0	0	0	0	0	0	0	0	0
16	6	0	148	31	80	32	339	5	10	1	15	423	19	44	3	66	161	69	3	15
0	0	0	0	0	0	0	0	0	0	0	0	0	0	0	0	0	0	0	0	0
0	0	0	1	0	0	0	2	0	0	0	1	1	0	0	0	0	14	0	0	0
13	4	0	0	0	0	0	0	2	8	4	8	22	0	0	0	0	0	0	0	0
0	1	0	0	0	0	0	0	0	0	0	0	0	0	0	0	0	0	0	0	0
5	116	123	90	145	275	335	91	129	163	114	109	187	1627	757	291	105	197	818	1965	0
0	0	0	0	0	0	0	0	0	1	0	1	0	0	0	0	0	0	0	0	0
0	42	0	0	0	0	25	0	7	2	3	2	7	0	14	0	0	0	1	1	0
21	17	0	0	0	0	0	0	4	6	4	11	10	0	4	0	0	0	32	0	0
0	0	0	0	0	0	0	0	0	0	0	0	0	0	0	0	0	0	0	0	0
0	0	0	0	0	0	0	0	0	1	0	0	0	0	5	0	5	0	13	0	6
1	0	0	0	0	0	0	0	0	0	0	0	0	0	0	0	0	0	0	0	0
0	0	0	0	0	0	0	0	0	0	0	0	0	0	0	0	0	0	0	0	0
0	0	0	0	0	0	0	0	0	0	0	0	0	0	0	0	0	0	0	0	0
2	1	0	0	0	0	0	0	0	3	0	0	2	13	0	0	13	0	0	0	0
0	0	0	0	0	0	0	0	0	0	0	0	0	0	0	9	0	0	0	0	0
0	0	0	0	0	0	0	0	0	0	0	0	0	0	0	0	0	0	0	0	0
10	68	45	104	14	68	18	0	69	116	26	31	35	465	315	151	240	81	66	49	13

E3.010	E3.012	E3.013	E3.016	E3.017	E3.018	E3.020	E3.021	E3.024	E3.025	E3.026	E3.027	E3.029	E3.030	E3.031	E3.032	E3.033	E3.034	E3.036	E3.037	E3.038
Esophag	Esophag	Esophag	Esophag	Esophag	Esophag	Esophag	Esophag	Esophag	Esophag	Esophag	Esophag	Esophag	Esophag	Esophag	Esophag	Esophag	Esophag	Esophag	Esophag	Esophagus
LE	LE	LE	LE	LE	LE	LE	LE	LE	LE	LE	LE	LE	LE	LE	LE	LE	LE	LE	LE	LE
1	0	0	0	0	0	0	0	0	0	0	0	0	0	18	0	0	0	0	0	0
0	0	0	0	0	0	0	0	0	0	0	0	0	0	0	0	0	0	0	0	0
0	0	0	0	0	0	0	0	0	0	0	0	0	0	26	0	0	0	0	0	0
5	0	0	0	0	0	0	0	0	0	0	0	5	0	0	0	2	0	0	0	17
0	0	0	0	0	0	0	0	0	0	0	0	0	0	0	0	0	0	0	0	0
1	0	0	0	0	0	0	0	2	1	2	0	2	0	2	0	30	0	0	0	13
5	0	0	0	0	0	0	0	2	2	4	3	14	0	0	0	22	0	0	0	5
157	138	491	0	1	80	10	0	24	90	76	93	200	0	17	0	102	0	0	170	27
3	0	0	0	0	0	0	0	0	2	0	2	1	0	0	0	0	0	0	0	0
0	0	0	0	0	5	0	0	1	0	0	0	0	0	2	0	0	0	0	0	0
251	489	8007	1390	4813	12739	1810	665	846	1049	600	1529	468	6343	15192	2315	3170	1548	4681	16157	83
0	0	0	0	0	0	0	0	0	0	0	0	0	0	0	0	0	0	0	0	0
3	2	0	0	0	0	0	0	0	6	0	4	9	0	0	3	0	0	0	0	0
0	0	0	0	0	0	0	0	0	0	0	2	0	0	0	0	0	15	0	0	12
0	0	1	0	0	0	0	0	0	0	0	0	0	0	0	0	0	0	0	0	0
0	0	0	0	0	0	0	0	0	0	0	0	0	0	0	0	0	0	0	0	0
3	0	0	0	0	0	0	0	1	0	2	5	0	0	0	0	0	0	0	0	0
0	14	29	0	0	11	0	0	2	8	11	1	3	12	8	17	0	17	0	33	0
2	0	0	0	0	0	0	0	1	0	0	1	0	0	0	0	0	0	0	0	0
5	3	50	0	15	38	19	0	10	23	5	7	15	40	49	56	86	0	24	74	107
0	0	0	0	0	0	0	0	0	0	0	0	0	0	0	0	0	0	0	0	0
6	4	0	0	0	0	0	0	3	4	0	1	24	0	0	0	0	0	0	0	0
2	2	0	0	0	0	0	0	1	1	1	2	6	0	0	0	0	0	0	0	27
0	0	0	0	0	0	0	0	0	0	0	0	0	0	0	0	0	0	0	0	0
1	2	0	0	0	0	0	83	6	6	7	2	8	0	5	0	0	0	37	44	12
8	2	0	0	0	0	0	0	4	2	0	0	12	0	2	0	0	0	0	0	87
0	0	0	0	0	0	0	0	0	0	0	0	0	0	0	0	0	0	0	0	0
0	0	0	0	0	0	0	0	0	0	0	0	0	0	0	0	0	0	0	0	0
0	0	0	0	0	0	0	0	0	0	0	0	1	0	0	0	0	0	0	0	0
0	0	0	0	0	0	0	0	0	0	0	0	0	0	0	0	0	0	0	0	0
0	0	0	0	0	0	0	0	0	0	0	0	0	0	0	0	0	0	0	0	0
4	0	2	0	0	78	0	0	2	1	0	0	0	0	0	0	207	0	0	0	109
0	0	0	0	0	0	0	0	0	0	0	0	0	0	0	0	0	0	0	0	0
19	6	0	0	0	0	0	0	2	24	109	9	17	0	0	0	0	0	0	4	198
0	0	0	0	0	0	0	0	0	0	0	1	1	0	0	0	0	0	0	0	0
0	0	0	0	0	0	0	0	0	0	0	0	0	0	0	0	0	0	0	0	0
0	0	0	0	0	1	0	0	0	0	0	0	1	0	0	0	0	0	0	0	0

E3.010	E3.012	E3.013	E3.016	E3.017	E3.018	E3.020	E3.021	E3.024	E3.025	E3.026	E3.027	E3.029	E3.030	E3.031	E3.032	E3.033	E3.034	E3.036	E3.037	E3.038
Esophag	Esophag	Esophag	Esophag	Esophag	Esophag	Esophag	Esophag	Esophag	Esophag	Esophag	Esophag	Esophag	Esophag	Esophag	Esophag	Esophag	Esophag	Esophag	Esophag	Esophagus
LE	LE	LE	LE	LE	LE	LE	LE	LE	LE	LE	LE	LE	LE	LE	LE	LE	LE	LE	LE	LE
8	1	0	0	0	85	0	0	2	3	1	8	4	0	1	0	95	0	0	0	134
0	0	0	0	0	0	0	0	0	0	0	1	0	0	0	0	0	0	0	0	0
0	0	0	0	0	0	0	0	0	0	0	0	0	0	0	0	0	0	0	0	0
0	0	0	0	0	0	0	0	0	0	0	1	0	0	0	0	0	0	0	0	0
0	0	0	0	0	0	0	0	0	0	0	0	0	0	0	0	0	0	0	0	0
8	1	0	0	0	0	0	0	4	6	0	12	2	0	0	0	4	0	0	0	10
7	0	0	0	0	0	0	0	0	1	0	3	0	0	0	0	0	0	0	0	0
78	3	21	0	14	0	28	0	38	30	4	85	19	0	0	0	275	0	0	0	566
0	40	0	0	0	0	0	0	1	2	0	0	0	0	8	0	0	0	0	6	0
0	2	0	0	0	0	0	0	1	5	11	4	0	0	15	3	0	0	0	3	13
12	2	0	10	0	0	0	0	1	4	3	11	5	0	0	0	95	18	0	0	196
52	3	0	0	4	0	0	0	11	6	0	33	26	0	0	0	44	0	0	0	41
0	0	0	0	0	0	0	0	0	0	0	0	0	0	0	0	0	0	0	0	0
1	0	0	0	7	0	0	0	0	0	0	1	0	0	0	0	0	0	0	0	4
9	3	0	0	0	0	0	0	1	4	0	4	2	0	0	0	23	0	0	0	0
0	3	0	0	0	0	0	0	0	2	0	0	0	0	0	0	0	0	0	0	241
5	35	0	1	10	0	300	0	1	6	18	6	3	0	3	5	30	0	0	19	8
14	2	0	0	0	0	11	0	1	7	0	20	0	0	0	0	67	0	0	0	113
11	8	0	0	0	54	0	0	6	8	149	11	15	0	10	0	0	96	0	3	180
19	1	0	0	6	3	0	0	15	18	2	24	10	0	1	0	122	0	0	0	104
67	12	62	78	42	17	43	0	34	102	44	152	30	0	38	6	442	56	1	26	805
0	1	0	0	0	0	0	0	0	0	0	0	0	0	0	0	0	0	0	0	0
0	0	0	0	0	0	0	0	4	0	13	0	0	0	1	0	0	0	0	7	0
0	0	0	0	0	0	0	0	0	0	0	1	0	0	0	0	0	0	0	0	1
1	0	0	0	0	0	0	0	0	0	0	1	0	0	0	0	6	0	0	0	0
0	1	0	0	0	47	0	0	11	2	17	0	0	12	10	17	0	47	0	55	0
42	20	0	0	8	83	0	0	13	20	44	23	17	33	28	52	0	122	59	41	7
1	0	0	0	6	41	0	0	0	0	8	4	1	0	3	31	3	0	15	0	3
0	0	0	0	0	0	0	0	0	0	0	0	0	0	0	0	0	0	0	0	0
0	0	0	0	0	0	0	0	0	0	0	0	0	0	0	0	0	0	0	0	0
5	7	0	0	0	0	0	0	4	45	8	52	5	0	0	0	0	0	0	0	0
35	5	0	52	1	157	0	64	7	6	2	14	40	0	8	0	198	30	82	0	466
10	4	0	0	6	0	0	0	0	20	0	25	0	0	0	0	0	0	0	0	29
0	0	0	0	0	0	0	0	0	0	0	0	0	0	0	0	0	0	0	0	0
65	9	3	10	0	37	0	0	3	30	8	55	34	0	3	0	251	61	23	12	396
0	0	0	0	0	0	0	0	0	0	0	0	0	0	0	0	0	0	0	0	0
0	0	0	0	0	0	0	0	0	0	0	0	0	0	0	0	0	0	0	0	0

E3.010	E3.012	E3.013	E3.016	E3.017	E3.018	E3.020	E3.021	E3.024	E3.025	E3.026	E3.027	E3.029	E3.030	E3.031	E3.032	E3.033	E3.034	E3.036	E3.037	E3.038
Esophag	Esophag	Esophag	Esophag	Esophag	Esophag	Esophag	Esophag	Esophag	Esophag	Esophag	Esophag	Esophag	Esophag	Esophag	Esophag	Esophag	Esophag	Esophag	Esophag	Esophagus
LE	LE	LE	LE	LE	LE	LE	LE	LE	LE	LE	LE	LE	LE	LE	LE	LE	LE	LE	LE	LE
7	6	25	0	0	2	0	0	3	7	7	2	7	0	2	4	0	14	0	7	0
0	0	0	0	0	0	0	0	0	0	0	0	0	0	0	0	0	0	0	0	0
22	11	23	1	9	0	54	2	19	25	37	6	12	0	22	0	0	0	0	9	362
0	0	0	0	0	0	0	0	0	0	0	1	0	0	0	0	0	0	0	0	0
0	0	0	0	0	1	0	0	0	0	0	1	0	0	0	0	0	0	0	0	0
1	15	15	3	2	0	29	12	0	1	5	11	4	25	165	1	8	4	0	20	3
0	0	0	0	0	0	0	0	0	0	0	0	0	0	0	0	0	0	0	0	0
0	0	0	0	0	0	0	0	0	0	0	0	0	0	0	0	0	0	0	0	0
12	29	52	214	244	90	2954	111	42	43	12	26	24	69	245	165	40	57	61	270	20
0	0	0	0	0	0	0	0	0	0	0	0	0	0	0	0	0	0	0	0	0
0	0	0	0	0	0	0	0	0	1	0	0	1	0	19	0	0	0	0	0	0
17	2	0	0	0	41	0	0	5	11	1	18	6	0	0	0	37	2	0	0	24
0	0	0	0	0	0	0	0	0	0	0	0	0	0	0	0	0	0	0	0	0
2	0	0	0	0	0	35	0	1	1	0	3	9	0	0	0	0	0	0	0	13
140	5	0	0	1	31	15	0	34	26	11	46	12	0	2	0	586	0	0	0	834
2	10	0	0	10	15	0	18	9	8	10	2	2	0	6	12	0	35	0	38	1
1	0	0	0	0	0	0	0	0	0	0	0	0	0	0	0	0	0	0	0	0
0	0	0	0	0	0	0	0	0	0	0	0	0	0	0	0	0	0	0	0	0
0	0	0	0	0	0	0	0	0	2	0	0	0	0	0	0	0	0	0	0	0
0	0	0	0	0	0	0	0	0	0	0	0	0	0	0	0	0	0	0	0	0
0	0	0	0	0	0	0	0	0	0	0	0	0	0	0	0	0	0	0	0	0
0	0	0	0	0	0	0	0	0	1	0	0	0	0	1	0	0	0	0	1	0
0	0	0	0	0	0	0	0	0	0	0	0	0	0	0	0	0	0	0	0	0
0	0	0	0	0	0	0	0	3	4	0	1	0	0	0	0	0	0	0	0	0
0	0	0	0	1	0	0	0	0	1	0	0	0	0	0	0	0	0	0	0	0
14	13	150	64	9	302	38	86	92	83	239	51	130	169	15	201	196	247	55	141	27
1	3	5	49	26	56	68	0	52	18	19	18	7	0	18	7	7	0	20	6	44
32	25	0	0	0	0	0	0	7	0	3	0	3	0	0	0	0	0	0	0	18
0	0	0	0	0	0	0	0	0	0	0	0	0	0	0	0	0	0	0	0	0
0	0	0	21	0	0	0	0	1	1	1	0	0	0	3	0	42	0	0	0	0
1	0	0	0	0	0	0	0	0	0	0	0	0	0	0	0	0	0	0	0	0
0	0	0	0	0	0	0	0	0	0	0	0	0	0	0	0	0	0	0	0	0
0	0	0	0	0	0	0	0	0	1	0	0	0	0	0	0	0	0	0	0	0
0	0	0	0	0	0	0	0	0	0	0	0	0	0	0	0	0	0	0	0	0
9	4	0	24	0	0	0	2094	13	7	16	26	53	0	1	0	0	23	20	1	0
4	0	0	5	11	0	0	0	2	2	16	3	6	1	0	0	0	0	38	0	0
2	0	0	0	0	0	0	0	1	6	0	3	4	0	0	0	0	29	0	0	0

E3.010	E3.012	E3.013	E3.016	E3.017	E3.018	E3.020	E3.021	E3.024	E3.025	E3.026	E3.027	E3.029	E3.030	E3.031	E3.032	E3.033	E3.034	E3.036	E3.037	E3.038
Esophag	Esophag	Esophag	Esophag	Esophag	Esophag	Esophag	Esophag	Esophag	Esophag	Esophag	Esophag	Esophag	Esophag	Esophag	Esophag	Esophag	Esophag	Esophag	Esophag	Esophagus
LE	LE	LE	LE	LE	LE	LE	LE	LE	LE	LE	LE	LE	LE	LE	LE	LE	LE	LE	LE	LE
29	9	1	0	1	0	0	0	116	15	14	123	33	0	1	0	0	0	0	1	0
0	0	0	0	0	0	0	0	0	0	0	0	0	0	0	0	0	0	0	0	0
118	51	269	209	107	536	196	1588	105	115	195	187	567	194	179	83	120	756	580	99	87
0	0	0	0	0	0	0	0	0	0	0	0	0	0	0	0	0	0	0	0	0
0	0	0	0	0	0	0	0	0	0	0	0	0	0	0	0	0	0	0	0	0
0	0	0	0	0	0	0	0	0	0	0	0	0	0	0	0	0	0	62	0	0
0	0	0	0	0	0	0	0	0	0	0	0	0	0	0	0	0	0	0	0	0
0	0	0	0	0	0	0	0	0	0	0	0	0	0	0	0	0	0	0	0	0
1	0	0	0	0	0	0	0	0	0	0	3	0	0	0	0	0	0	0	0	0
0	0	0	0	0	0	0	0	0	0	0	0	0	0	0	0	0	0	0	0	0
0	0	0	0	19	0	0	0	0	0	2	1	0	0	0	0	0	0	0	0	0
19	4	90	3	30	44	67	55	2	20	26	13	145	62	25	13	9	171	234	24	23
0	0	0	0	0	0	0	0	0	0	0	0	0	0	0	0	0	0	0	0	0
1	1	0	6	8	0	10	22	1	1	5	15	5	0	1	3	0	48	34	0	0
23	22	2	2	0	24	1	306	66	45	213	53	37	8	10	20	81	536	168	1	18
5	2	0	0	0	0	0	0	3	1	1	3	3	0	0	0	0	0	0	0	0
2	3	0	28	0	0	0	0	2	0	0	1	2	0	1	0	0	0	0	0	0
0	0	0	0	0	0	0	0	0	0	0	0	0	0	4	0	0	23	0	0	0
0	1	0	0	0	0	0	0	0	0	0	0	0	0	0	0	0	0	0	0	0
0	0	2	0	0	0	0	0	1	0	0	0	1	0	0	0	0	0	0	0	0
0	0	0	0	0	0	0	0	0	0	0	0	0	0	0	0	0	0	0	0	0
0	1	0	0	0	0	0	0	0	0	0	0	0	0	0	0	0	0	0	0	0
20	6	1	26	15	39	31	182	11	25	4	46	17	89	8	4	28	125	79	3	32
14	5	10	6	37	10	5	15	10	31	4	32	12	20	1	0	14	24	0	0	105
1	0	0	0	0	0	0	0	0	0	3	0	0	0	0	0	0	0	0	0	0
0	0	0	0	0	0	0	0	0	0	0	0	0	0	0	0	0	0	0	0	0
0	0	0	0	0	0	0	0	0	0	0	0	0	0	0	0	0	0	0	0	0
0	0	0	0	0	0	0	0	0	0	0	0	0	0	0	0	0	0	0	0	0
0	0	0	0	0	0	0	0	0	0	0	0	0	0	0	0	0	0	0	0	0
1	2	0	0	2	0	1	1	1	3	2	9	3	0	0	0	10	0	6	0	0
0	0	0	0	0	0	0	0	0	0	0	0	0	0	0	0	0	0	21	0	0
0	0	0	0	0	42	0	92	1	1	0	0	3	0	2	24	0	0	262	0	0
0	0	0	0	0	0	0	0	0	0	0	0	0	0	0	0	0	0	0	0	0
0	0	0	0	0	0	0	0	0	0	0	0	0	0	0	0	0	0	0	0	1
6	1	0	0	0	0	0	56	0	0	0	0	3	0	0	0	0	0	0	0	0
0	0	0	0	0	0	0	0	0	0	0	0	0	0	0	0	0	0	0	0	0
0	0	0	0	0	0	0	0	0	0	0	0	0	0	0	0	0	0	50	0	0

E3.010	E3.012	E3.013	E3.016	E3.017	E3.018	E3.020	E3.021	E3.024	E3.025	E3.026	E3.027	E3.029	E3.030	E3.031	E3.032	E3.033	E3.034	E3.036	E3.037	E3.038
Esophag	Esophag	Esophag	Esophag	Esophag	Esophag	Esophag	Esophag	Esophag	Esophag	Esophag	Esophag	Esophag	Esophag	Esophag	Esophag	Esophag	Esophag	Esophag	Esophag	Esophagus
LE	LE	LE	LE	LE	LE	LE	LE	LE	LE	LE	LE	LE	LE	LE	LE	LE	LE	LE	LE	LE
0	0	0	0	0	0	0	0	0	0	0	0	0	0	0	0	0	0	0	0	0
0	0	0	0	0	0	0	0	1	0	0	1	0	0	0	0	0	0	0	0	0
0	0	0	0	0	0	0	0	0	0	0	1	0	0	0	0	0	0	0	0	0
0	0	0	0	0	0	0	0	0	0	0	0	0	0	0	0	0	0	0	0	0
4	1	0	0	0	0	0	0	0	4	0	2	9	0	5	0	0	0	0	0	0
0	0	0	0	0	0	0	0	0	1	0	0	1	0	0	0	0	0	0	0	0
2	0	0	0	0	0	0	0	0	3	0	0	0	0	2	0	0	0	0	0	0
0	1	0	0	0	0	0	0	0	0	0	0	0	0	0	0	0	0	0	0	0
4	1	0	0	0	0	0	0	0	1	0	2	5	0	5	0	0	0	0	0	0
1	0	0	0	0	0	0	0	1	0	0	1	0	0	0	0	0	0	0	0	0
0	0	0	0	0	0	0	0	0	0	0	0	0	0	0	0	0	0	0	0	0
0	0	0	0	0	0	0	0	0	0	0	0	0	0	0	0	0	0	0	0	0
1	0	0	0	0	0	0	0	1	0	0	0	0	0	0	0	52	0	0	0	0
1	0	0	0	0	0	0	0	1	2	0	1	1	0	0	0	0	0	0	0	0
1	1	0	11	2	0	2	25	2	0	1	1	1	17	0	3	11	15	6	2	9
0	0	0	0	0	0	0	0	0	0	0	0	0	0	0	0	0	0	0	0	0
0	0	0	0	0	0	0	0	0	0	0	2	5	0	0	0	0	0	0	0	0
0	0	0	0	0	0	0	0	0	0	0	0	0	0	0	0	0	0	0	0	0
0	0	0	0	0	0	0	0	0	0	0	0	0	0	0	0	0	0	0	0	0
8	4	27	0	1	51	37	0	3	8	10	15	64	4	6	5	7	0	0	0	0
0	0	0	0	0	0	0	0	0	0	0	0	0	0	0	0	0	0	0	10	0
0	0	0	0	0	0	0	0	0	0	0	0	0	0	0	0	0	0	0	0	0
0	0	0	0	0	0	0	0	0	0	0	0	0	0	0	0	0	0	0	0	0
0	0	0	0	0	0	0	0	0	0	0	0	0	0	0	0	0	0	0	0	0
2	1	0	0	0	2	0	0	0	2	0	2	7	6	10	0	0	0	0	0	0
1	0	0	0	0	63	0	0	0	4	0	3	7	0	0	0	0	0	0	0	0
1	0	0	0	0	0	0	0	0	1	1	1	1	0	0	0	0	0	2	0	0
38	14	0	17	13	1	31	0	145	128	31	196	113	22	30	28	0	54	7	10	0
33	34	0	0	0	46	0	438	94	153	1	69	34	0	3	0	0	0	0	0	0
989	454	1	96	18	174	18	830	6330	597	14807	2857	428	74	49	22	96	286	390	44	12
0	5	0	10	0	0	0	0	0	4	0	3	2	0	0	0	0	0	25	0	0
1	2	0	0	0	1	0	0	0	10	1	19	2	0	0	0	0	0	0	0	0
1	2	0	0	0	0	0	0	0	3	3	15	6	0	1	0	0	0	0	0	0
0	0	0	0	0	0	0	0	0	0	0	0	0	0	0	0	0	0	0	0	0
0	0	0	0	0	0	0	0	0	0	0	0	0	0	0	0	0	0	0	0	0
2	3	0	0	0	0	0	0	0	0	0	1	6	0	0	0	0	0	0	0	0
6	2	0	0	0	0	0	0	1	0	5	0	4	0	0	0	418	177	0	0	499

E3.010	E3.012	E3.013	E3.016	E3.017	E3.018	E3.020	E3.021	E3.024	E3.025	E3.026	E3.027	E3.029	E3.030	E3.031	E3.032	E3.033	E3.034	E3.036	E3.037	E3.038
Esophag	Esophag	Esophag	Esophag	Esophag	Esophag	Esophag	Esophag	Esophag	Esophag	Esophag	Esophag	Esophag	Esophag	Esophag	Esophag	Esophag	Esophag	Esophag	Esophag	Esophagus
LE	LE	LE	LE	LE	LE	LE	LE	LE	LE	LE	LE	LE	LE	LE	LE	LE	LE	LE	LE	LE
0	0	0	0	0	0	0	0	0	0	1	0	0	0	0	0	0	0	0	0	0
2	3	0	0	0	0	0	0	5	1	14	3	2	0	0	0	0	0	0	0	0
0	0	0	0	0	0	0	0	0	0	0	0	0	0	0	0	0	0	1	0	0
4	21	0	12	23	0	35	0	15	54	40	15	55	43	15	21	16	65	0	78	0
0	0	0	0	0	0	0	0	0	0	0	0	1	0	0	0	0	0	0	0	0
0	0	0	0	0	0	0	0	0	0	0	0	3	0	0	11	2	13	0	0	0
1	2	1	13	0	0	0	0	2	5	6	11	19	0	0	0	0	0	0	0	0
0	1	0	0	0	0	0	0	0	6	2	5	24	0	3	0	0	94	4609	0	9
0	0	0	0	0	0	0	0	0	0	0	0	0	0	0	0	0	0	0	0	0
3	0	0	0	0	0	0	0	2	1	2	5	2	0	0	0	0	0	11	0	0
0	0	0	0	0	0	0	0	0	0	0	0	2	0	0	0	0	0	0	0	0
0	0	0	0	0	0	0	0	0	0	0	0	1	0	0	0	0	0	0	0	0
0	1	0	0	0	0	0	0	0	1	0	0	0	0	0	0	0	0	16	0	0
0	0	0	0	0	0	0	0	0	0	0	0	0	0	0	0	0	0	0	0	0
0	0	0	2	0	0	0	0	0	0	0	1	1	0	5	0	4	0	0	0	0
0	0	0	0	2	0	0	0	0	0	0	0	0	0	0	0	0	5	11	3	0
0	0	0	0	0	0	0	0	0	0	0	0	0	0	0	0	0	0	0	0	0
0	0	0	0	0	0	0	0	0	0	0	0	0	0	0	0	9	0	0	0	0
0	0	0	0	0	0	0	0	0	0	0	0	0	0	0	0	0	0	0	0	0
0	0	0	0	0	0	0	0	0	0	0	0	0	0	0	0	0	0	0	0	0
1	0	0	0	0	0	0	0	1	0	0	1	1	0	0	0	0	0	0	0	0
3	1	0	0	0	0	0	0	8	24	0	7	11	3	2	0	23	0	117	0	0
15	10	19	21	10	34	0	84	13	17	13	20	46	17	43	1	0	29	239	29	20
21	2	0	6	0	0	0	0	30	16	0	18	26	0	0	0	0	0	36	0	0
1	1	0	0	0	0	0	0	0	0	0	0	0	0	0	0	0	0	0	0	0
1	0	1	23	0	10	29	169	0	1	0	0	0	0	6	12	46	12	60	10	19
9	1	0	0	23	1	9	18	1	3	0	2	12	14	1	9	12	30	26	0	11
13	8	0	23	6	0	0	99	44	10	0	14	14	8	6	0	26	33	67	0	0
0	1	0	0	0	0	0	0	0	1	0	0	0	0	0	0	0	0	0	0	0
1	0	0	0	0	0	0	0	0	1	0	0	1	0	0	0	0	0	0	0	0
0	0	0	0	0	0	0	0	0	0	0	0	0	0	0	0	0	0	0	0	0
2	0	0	0	0	0	0	0	0	0	0	1	3	0	0	0	0	0	0	0	12
1	43	0	0	8	0	28	0	9	6	9	1	8	19	6	23	0	0	0	0	0
0	4	0	0	20	75	0	0	0	25	0	0	4	0	0	128	0	0	0	0	0
384	1711	32	2169	714	991	25	294	280	1279	624	148	416	1681	578	2885	244	677	678	205	169
0	2	0	0	0	0	0	0	0	8	0	2	3	9	2	14	0	0	0	0	0
11	47	0	0	8	0	0	0	10	71	53	21	20	45	55	263	26	203	2	12	0

E3.010	E3.012	E3.013	E3.016	E3.017	E3.018	E3.020	E3.021	E3.024	E3.025	E3.026	E3.027	E3.029	E3.030	E3.031	E3.032	E3.033	E3.034	E3.036	E3.037	E3.038
Esophag	Esophag	Esophag	Esophag	Esophag	Esophag	Esophag	Esophag	Esophag	Esophag	Esophag	Esophag	Esophag	Esophag	Esophag	Esophag	Esophag	Esophag	Esophag	Esophag	Esophagus
LE	LE	LE	LE	LE	LE	LE	LE	LE	LE	LE	LE	LE	LE	LE	LE	LE	LE	LE	LE	LE
0	0	0	0	0	0	0	0	0	0	0	0	0	0	0	0	0	0	0	0	0
0	0	0	0	0	0	0	0	0	0	0	0	1	0	0	0	0	0	2	0	0
1	1	0	0	2	12	0	28	1	1	3	13	20	3	0	0	0	0	0	0	0
0	0	0	0	0	0	0	0	0	0	0	0	0	0	0	0	0	0	0	0	0
0	0	0	0	0	0	0	0	0	0	0	2	0	0	0	0	0	0	0	0	0
0	0	0	0	0	16	0	0	0	0	0	0	0	0	3	0	27	0	15	2	1
2	0	0	0	0	0	0	0	0	1	0	4	0	0	0	0	0	0	0	0	0
1	1	0	0	0	0	0	0	0	0	0	0	0	0	0	0	0	0	0	0	0
0	0	0	0	0	0	0	0	0	0	0	2	0	0	0	0	0	0	0	0	0
0	0	0	0	0	0	0	0	1	0	0	0	0	0	1	18	0	0	0	4	0
0	0	0	0	0	0	0	0	0	0	0	0	0	0	4	0	0	0	0	0	0
0	0	0	0	0	0	0	0	0	0	0	0	0	0	0	0	0	0	0	0	0
4	0	0	0	0	0	11	0	0	2	0	5	0	0	1	0	38	0	0	0	20
1	1	0	0	0	0	0	0	0	4	0	1	0	0	0	0	58	0	0	0	9
0	0	0	0	0	0	0	0	0	5	0	0	0	0	0	0	0	0	0	0	0
0	0	0	0	0	0	0	0	0	0	0	0	0	0	0	0	0	0	0	0	0
0	0	0	0	0	0	0	0	0	0	0	0	0	0	0	0	0	0	0	0	0
0	0	0	0	0	0	0	0	0	0	0	0	0	0	0	0	0	0	0	0	0
0	0	0	0	0	0	0	0	0	0	0	0	0	0	0	0	0	0	0	0	0
0	0	0	0	0	0	0	0	0	0	0	0	0	0	0	0	0	0	0	0	0
3	14	0	0	34	44	162	2	32	18	59	19	41	11	110	239	65	116	0	186	20
0	0	0	0	0	0	0	0	0	0	0	0	1	0	0	0	0	0	0	0	0
1	35	0	0	4682	139	0	586	2	897	292	12	0	377	19	0	0	0	0	0	0
0	0	0	0	0	0	0	0	0	0	0	0	0	0	0	0	0	0	0	0	0
0	0	0	0	0	0	0	0	0	0	0	0	0	0	0	0	0	0	0	0	0
0	0	0	0	0	0	0	0	0	0	0	0	0	0	0	0	0	3	0	0	0
1	0	0	0	0	0	0	0	0	1	0	0	0	0	0	0	0	34	0	0	0
1	0	0	0	0	0	0	0	0	0	0	4	1	0	0	0	0	0	0	0	0
0	0	0	0	0	0	0	0	0	0	0	0	1	0	0	0	0	0	0	0	0
0	0	0	0	0	0	0	0	0	0	0	0	0	0	0	0	0	0	0	0	0
0	0	0	0	7	0	0	0	0	0	0	0	0	1	0	0	0	0	0	0	0
0	0	0	0	0	0	0	0	0	0	0	0	0	0	0	0	0	0	0	0	0
0	0	0	0	0	0	0	0	0	0	0	0	0	0	0	0	25	0	0	0	10
0	0	0	0	0	0	0	0	0	0	0	2	0	0	0	0	0	0	0	0	0
0	0	0	0	0	0	18	0	0	0	0	0	0	0	0	0	0	0	0	0	0
0	0	0	0	0	0	0	0	2	0	0	0	0	0	0	0	57	0	0	0	9
0	2	0	0	0	0	0	0	5	7	4	0	0	0	0	0	0	0	0	0	0

E3.010	E3.012	E3.013	E3.016	E3.017	E3.018	E3.020	E3.021	E3.024	E3.025	E3.026	E3.027	E3.029	E3.030	E3.031	E3.032	E3.033	E3.034	E3.036	E3.037	E3.038
Esophag	Esophag	Esophag	Esophag	Esophag	Esophag	Esophag	Esophag	Esophag	Esophag	Esophag	Esophag	Esophag	Esophag	Esophag	Esophag	Esophag	Esophag	Esophag	Esophag	Esophagus
LE	LE	LE	LE	LE	LE	LE	LE	LE	LE	LE	LE	LE	LE	LE	LE	LE	LE	LE	LE	LE
0	0	0	0	0	0	0	0	0	6	0	0	0	0	0	0	0	0	0	0	0
0	0	0	0	0	0	0	0	0	0	0	0	0	0	0	0	0	0	0	0	0
2	2	0	0	0	0	0	0	1	4	2	0	7	0	2	0	0	0	0	0	5
3	0	0	0	0	0	0	0	0	5	0	1	4	0	4	0	0	2	0	0	0
0	0	0	0	0	0	0	0	0	0	0	0	1	0	0	0	0	0	0	0	0
0	0	0	0	0	0	0	0	0	0	0	0	0	0	0	0	0	0	0	0	0
0	0	0	0	0	0	0	0	0	0	0	0	0	0	0	0	0	0	0	0	0
5	1	0	0	0	0	0	0	0	0	1	0	0	0	2	0	0	0	0	0	0
0	0	0	0	0	0	0	0	0	0	0	0	0	0	0	0	0	0	0	0	0
0	0	0	0	0	0	0	0	0	0	0	0	1	0	0	0	0	14	5	0	0
3	1	0	26	0	2	38	1	0	1	1	1	3	0	10	9	0	43	26	0	0
1470	204	142	75	70	2833	125	367	283	218	795	363	1365	87	174	85	189	4648	4693	497	397
0	0	0	0	0	0	0	0	0	0	0	0	0	0	0	0	0	0	0	0	0
0	0	0	0	0	0	15	21	0	0	0	0	0	2	12	0	0	0	0	0	0
0	0	0	0	0	0	0	0	0	0	0	0	0	0	0	0	0	0	0	0	0
0	0	0	0	0	0	0	0	0	0	0	0	0	0	0	0	0	0	0	0	0
0	0	0	0	0	0	0	0	0	0	0	0	0	0	0	0	52	0	0	0	16
0	0	0	0	0	0	0	0	0	0	0	1	2	0	0	0	0	0	0	0	0
157	126	25	5394	4693	72	4	1655	45	1129	168	148	893	4425	242	1275	0	0	2293	8	0
13	95	0	0	0	30	0	2	19	76	27	30	10	135	97	119	105	27	0	0	8
159	111	44	891	273	807	529	301	614	636	201	246	175	2102	342	1071	1547	320	249	63	49
0	0	0	0	0	0	0	0	0	0	0	0	0	0	0	0	0	0	0	0	0
0	0	0	0	0	0	0	0	0	0	0	0	0	0	0	0	0	0	0	64	0
1	2	0	0	0	0	0	0	2	2	0	1	2	0	0	0	0	0	0	0	0
0	0	0	0	0	0	0	0	0	0	0	0	0	0	0	1	0	0	0	0	0
6	19	8	19	11	48	6	69	6	13	19	16	17	77	7	0	209	173	3017	11	29
0	0	0	0	0	0	0	0	0	0	0	0	0	0	0	0	0	0	0	0	0
14	15	0	0	0	8	0	0	2	66	1	35	75	15	1	198	0	0	4	0	3
22	3	0	0	0	53	10	0	0	19	1	7	1	0	35	78	0	0	1	0	14
0	0	0	0	0	0	0	0	0	0	0	0	3	0	0	0	7	0	0	0	0
0	2	0	0	0	0	0	0	0	0	0	2	5	0	0	0	0	0	0	0	0
0	0	0	0	0	0	0	0	0	0	0	0	0	0	0	0	0	0	0	0	0
4	2	0	0	0	0	0	0	0	1	3	1	13	19	0	0	0	6	157	0	0
176	238	0	63	0	23	28	252	144	180	106	173	729	6	7	0	28	122	108	5	15
0	5	0	1	0	0	1	0	2	3	0	2	4	0	0	0	1	0	2	0	0
6	1	0	15	0	1	0	1	0	8	1	9	13	0	0	0	0	0	0	1	8
0	0	0	0	0	0	0	0	0	1	0	0	1	0	0	0	0	0	0	0	0

E3.010	E3.012	E3.013	E3.016	E3.017	E3.018	E3.020	E3.021	E3.024	E3.025	E3.026	E3.027	E3.029	E3.030	E3.031	E3.032	E3.033	E3.034	E3.036	E3.037	E3.038
Esophag	Esophag	Esophag	Esophag	Esophag	Esophag	Esophag	Esophag	Esophag	Esophag	Esophag	Esophag	Esophag	Esophag	Esophag	Esophag	Esophag	Esophag	Esophag	Esophag	Esophagus
LE	LE	LE	LE	LE	LE	LE	LE	LE	LE	LE	LE	LE	LE	LE	LE	LE	LE	LE	LE	LE
0	0	0	0	0	0	0	0	0	0	0	0	0	0	0	0	0	0	0	0	0
0	0	0	0	0	0	0	0	0	0	0	0	0	0	0	0	0	0	0	0	0
0	0	0	0	0	0	0	0	0	0	0	0	0	0	0	0	0	0	0	0	0
0	0	0	0	0	0	0	0	0	0	0	0	1	0	0	0	0	0	0	0	0
3	0	0	9	1	0	0	0	0	1	0	4	11	0	7	1	0	29	0	10	0
1	3	0	0	0	0	0	48	0	3	7	3	5	0	1	0	0	0	0	1	0
0	0	0	0	0	0	0	0	0	0	0	0	0	0	0	0	0	0	0	0	0
0	0	0	0	0	0	0	27	0	0	0	1	0	0	1	0	0	0	54	6	5
0	0	0	0	0	0	0	0	0	0	0	0	0	0	0	0	0	0	0	0	0
0	0	0	0	0	0	0	0	0	0	0	2	1	0	0	0	0	0	0	0	0
1	0	0	0	0	0	0	0	0	0	0	1	0	0	0	0	0	0	0	0	0
34	18	22	34	0	49	10	135	49	22	158	41	40	17	6	0	77	31	61	3	24
0	0	0	0	0	0	0	0	0	0	0	0	0	0	0	0	0	0	32	0	0
9	2	0	0	0	0	0	0	0	1	3	3	26	0	0	0	0	0	47	0	0
5	1	0	30	0	0	0	0	0	1	3	0	9	0	0	0	1	0	141	0	0
0	0	0	0	0	0	0	0	0	0	0	0	0	0	0	0	0	0	0	0	0
0	0	0	0	0	0	0	0	0	0	0	0	0	0	0	0	0	0	0	0	0
0	0	0	0	0	0	0	0	0	0	0	0	0	0	0	0	0	0	0	3	0
3	1	0	0	0	75	25	0	12	14	20	22	1	26	74	6	68	13	3	25	23
0	0	0	0	0	0	0	0	0	0	0	0	0	0	0	0	0	0	0	0	0
0	0	0	0	0	12	0	0	1	0	3	1	0	4	6	5	0	0	0	4	0
0	0	0	0	0	0	0	0	0	0	0	0	0	0	0	0	0	0	0	0	0
0	0	0	0	0	0	0	0	0	0	0	0	0	0	0	0	0	0	0	0	0
0	0	0	0	0	0	0	0	0	8	0	2	1	0	20	0	0	0	0	11	0
24	38	0	0	0	0	0	0	10	3	18	3	2	0	0	1	19	0	0	3	0
2	1	0	0	0	0	0	0	1	1	1	2	3	0	0	0	0	0	0	0	0
0	0	0	0	0	0	0	0	0	0	0	0	2	0	0	0	0	0	0	0	0
0	0	0	0	0	0	0	0	0	0	0	0	0	0	0	0	0	0	0	0	10
0	0	24	0	2	0	0	0	1	0	3	1	2	1	0	6	0	17	0	0	5
21	9	34	0	0	12	0	0	25	399	4	34	16	0	3	28	0	0	51	14	0
0	0	0	0	0	0	0	0	0	0	0	0	0	0	0	0	0	0	0	0	0
0	0	0	0	0	0	0	0	0	0	0	0	0	0	2	0	0	0	0	0	62
