# Supplementary material for: Microbial Similarity and Preference for Specific Sites in Healthy Oral Cavity and Esophagus
Source: Front Microbiol. 2018 Jul 17;9:1603. doi: 10.3389/fmicb.2018.01603 (PMC6056649; doi:10.3389/fmicb.2018.01603)
Supplement: Supplementary file 5 [file Image_1.PDF]

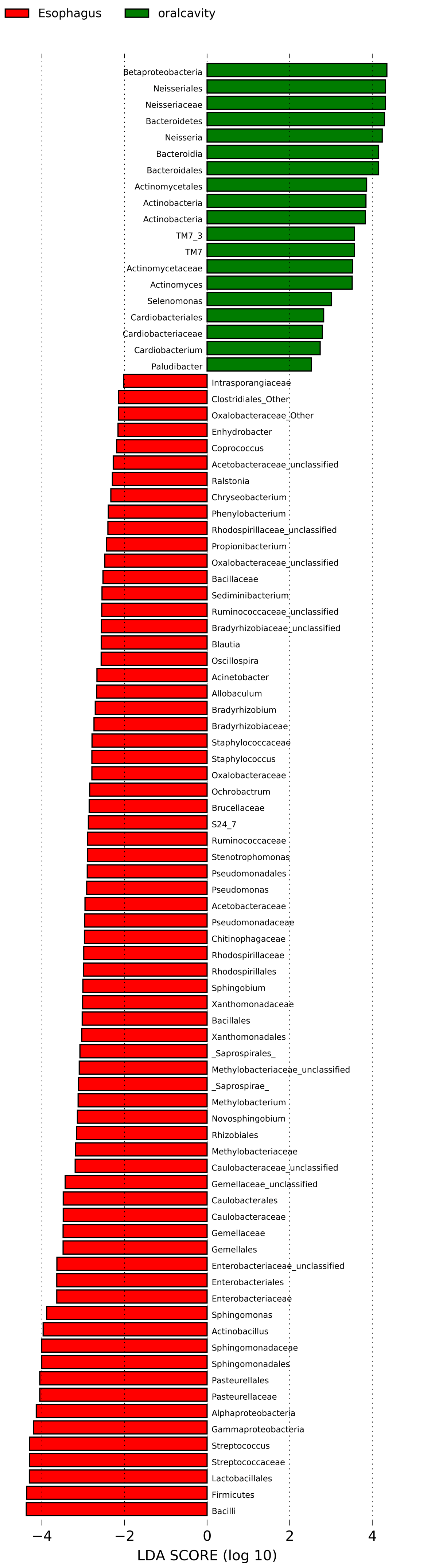

Figure Sl. LDA score for the beta diversity between the oral cavity and esophagus
